# Global burden of major gastrointestinal cancers and its association with socioeconomics, 1990–2019

**DOI:** 10.3389/fonc.2022.942035

**Published:** 2022-11-01

**Authors:** Mei-Zhu Hong, Jing-Mao Li, Zhi-Jian Chen, Xiao-Yun Lin, Jin-Shui Pan, Li-Li Gong

**Affiliations:** ^1^ Department of Traditional Chinese Medicine, Mengchao Hepatobiliary Hospital of Fujian Medical University, Fuzhou, Fujian, China; ^2^ Department of Statistics, School of Economics, Xiamen University, Xiamen, Fujian, China; ^3^ Department of Hepatology, First Affiliated Hospital of Fujian Medical University, Fuzhou, Fujian, China; ^4^ Department of Health Care, Zhongshan Hospital, Xiamen University, Xiamen, Fujian, China

**Keywords:** epidemiology, public health, socioeconomics, gastrointestinal oncology, colorectal cancer

## Abstract

**Background:**

To understand the impact of common cancers of the gastrointestinal tract and help to formulate evidence-based policy, we evaluate the relationship between the burden of GI tract cancers and socioeconomics.

**Methods:**

Data on GI tract cancer burden were obtained from the Global Burden of Disease (GBD) 2019 including mortality and incidence rates. According to the Socio-demographic Index (SDI) level, country and territory, and sex, *etc.*, the data were further stratified. The association between the burden of GI tract cancer and socioeconomics, indicated by SDI, was described. Uncertainty analysis was estimated using bootstrap draw.

**Results:**

In 2019, five major cancers of the gastrointestinal tract led to an age-standardized incidence rate (ASIR) of 61.9 (95% CI 56.1–67.6) per 100 000 person-years. From 1990 to 2019, five common tumors of the gastrointestinal tract related age-standardized death rates (ASDRs) decreased by −22.7% (−31.1 to −13.5). For the five common tumors, ASIRs and ASDRs were both higher in males than those in females. Globally, Mongolia, and several East Asia countries exhibited the highest ASIRs in 2019. The high SDI, and high-middle SDI locations recorded the highest incidence rate and death rate of colon and rectum cancer and pancreatic cancer. On the contrary, the low-middle SDI, and low SDI locations possessed the highest incidence rate and death rate of stomach cancer and esophageal cancer.

**Conclusion:**

There is a profound association between socioeconomics and burden of common cancers of the gastrointestinal tract. It would be helpful for the high SDI, and high-middle SDI locations to pay special attention to the screening of colon and rectum cancer and pancreatic cancer while the low-middle SDI, and low SDI locations should pay more attention to the screening of stomach cancer and esophageal cancer.

## Introduction

According to the report from Global Burden of Diseases, Injuries, and Risk Factors Study (GBD) 2017, global estimates of all causes-related deaths are 55.9 million with an age-standardized death rate (ASDR) of 737.7 per 100 000 for 2017. Among these causes, neoplasms contribute to 9.6 million deaths and 121.2 per 100 000 of ASDRs (1). Besides, common gastrointestinal tract cancers are major contributors of the top seven causes of cancer-related death, including colon and rectum cancer (CRC), stomach cancer, liver cancer, pancreatic cancer, and esophageal cancer. Overall, common gastrointestinal tract cancers accounted for 36.2% of neoplasms-related deaths (1). The situation is similar in China. According to the Cancer Statistics in China 2015, the gastrointestinal tract cancers occupy five out of the six leading causes of cancer-related death except for lung cancer (2). Given the background exposed above, the proper handle of gastrointestinal tract tumors would significantly reduce the overall burden of tumor-related death.

Gastrointestinal tract cancers have some unique characteristics. First, it shows substantial geographical and temporal heterogeneity. For example, the ASIR of CRC in the Netherlands is 50.9 per 100 000 person-years, which is nine times more compared to that of Iraq in 2017 (3). Second, most common gastrointestinal tract cancers can be detected by routine examination in clinical practice. Third, risk or environmental factors play a crucial role in the tumorigenesis of gastrointestinal tract cancers such as *Helicobacter pylori* for stomach cancer, Hepatitis B virus for liver cancer.

Cumulating pieces of evidence have indicated that further intervention on related environmental factors should be incorporated into the prevention, diagnosis, and treatment of gastrointestinal tract cancers. Highlighting global and geographical trends can help to generate specialized local and global interventions to reduce disease burden of common gastrointestinal tract cancers, and curtail the increasing of incident case numbers. Given the heavy burden of gastrointestinal tract cancers, several studies have focused on individual type of common cancer of the gastrointestinal tract (3–7). Recently, Arnold et al. (8) described the global burden due to common gastrointestinal tract cancers. Here, we further revealed the association between socioeconomics and the global burden of common cancers from the gastrointestinal tract.

## Methods

### Data sources

Age-standardized incidence rate (ASIR) and ASDR of common cancer of the gastrointestinal tract in this study were acquired from the Global Burden of Disease Study 2019, which covers 204 countries and territories (9). The International Classification of Diseases (the 10th revision) was adopted. Mortality and non-fatal estimates were described detailed in the previous studies (3, 7). Supplementary information was provided in **Supplementary Methods**.

### Uncertainty analysis

The 95% confidence interval (CI) of ASIR and ASDR of a specific tumor at a specific time point was extracted directly from GBD 2019. Uncertainty analysis on changing rate of ASIR or ASDR at a specific time point compared to another time point was calculated using the following method: 1) we assumed that the ASIR and ASDR in each year followed a log-normal distribution, and also the rates in different years were independent with each other. 2) Upon these assumptions, we performed uncertainty analysis. Based on the 25th and 975th ranked values in all 1000 draws of a round of bootstrap draw, we measured the increase rate and the 95% CIs.

### Sociodemographic Index

Sociodemographic Index (SDI) incorporates lag-distributed income per person, mean education for individuals aged ≥15 years, and the total fertility rate in women under the age of 25 years. SDI was generated according to the report by GBD 2016 Mortality Collaborators (10). The values of SDI ranges from 0 to 1, which reflects the development level of a country from worst to best. Low SDI, low-middle SDI, middle SDI, high-middle SDI, and high SDI, were set by < 20^th^, 20^th^–39^th^, 40^st^–59^th^, 60^th^–79^th^, ≥ 80^th^ percent of SDI values.

### Statistical analysis

Both ASIR and ASDR of common cancer of the gastrointestinal tract were further stratified by sex or SDI, which were also obtained from GBD 2019 (https://www.healthdata.org/). The obtained data were shown in appendix. Python 3.7 (https://www.python.org/) was employed to generate the figures, which were drawn based on the obtained data shown in the appendix.

## Results

### The burden of common gastrointestinal tract cancers

**Figure 1:**
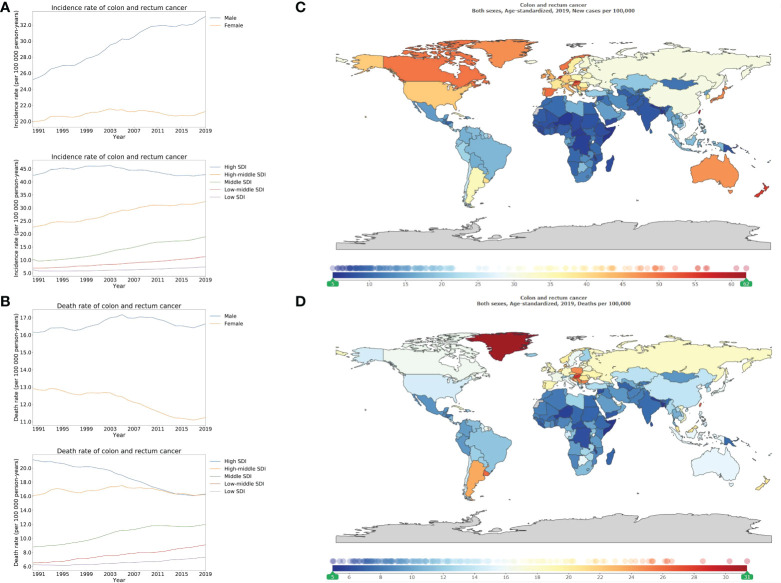
Burden of colorectal cancer for 204 countries and territories. Age-standardized incidence **(A)** and death **(B)** rate per 100000 population of colorectal cancer from 1990 through 2019 by country and territory, stratified by sex or SDI; age-standardized incidence **(C)** and death **(D)** rate of colorectal cancer per 100000 person-years by country and territory, in 2019. The maps in **(C)** and **(D)** were generated by GBD 2019 tool.

According to the ASIR, colon and rectum cancer ranked the first out of the five major tumors, followed by stomach cancer, liver cancer, esophageal cancer, and pancreatic cancer. After age-standardization, there was an incidence rate of 61.9 (95% UI 56.1–67.6) and an death rate of 44.2 (40.9–47.6) per 100 000 person-years, in terms of 5 common gastrointestinal tract cancers in 2019 (**S.Figure 1A**, appendix p 14). From 1990 to 2019, ASIRs of stomach cancer, esophageal cancer, and liver cancer, depicted a decreasing trend per annum (**S.Figure 1A**). In the same period, there was a decline in ASDRs due to the five common tumors (−22.7% [−31.1 to −13.5]; **S.Figure 1B**; appendix p 8). Furthermore, in 2019, several East Asia countries or regions such as Mongolia, Taiwan (Province of China), Japan, Korea, and China, suffered the highest burden of the five common cancers evaluated by incidence rate (**S.Figure 1C**; appendix pp 19–34). Besides, the following countries or regions namely Mongolia, Greenland, North Korea, China, and Guinea carried the greatest burden in 2019, measured by the overall death rate associated with the five common cancers (**S.Figure 1D**; appendix pp 35–50).

### The burden of colon and rectum cancer

Stratified using SDI, the ASIR of colon and rectum cancer was higher for high SDI locations compared to those that were lower in the high-middle, middle, low-middle, and low SDI locations (**Figure 1A**; appendix pp 53–61). In the past 30 years, a decreasing pattern was revealed in the ASDR of colon and rectum cancer in females compared with that remained stable in males (**Figure 1B**). The percentage change in ASIRs from 1990 to 2019 differed significantly between the SDI regions, with high-middle SDI locations (143.4% [129.6–158.8]), middle SDI locations (184.5% [162.3–209.6]) showing increases (**Figure 1A**; appendix p 10). On the other hand, death rates of high SDI locations (76.9% [71.0–83.3]) significantly declined during the same period (appendix p 9). Regarding the ASIR and ASDR, the males exhibited higher rates for colon and rectum cancers compared to the females (**Figures 1A, B**). In 2019, the ASIR of colon and rectum cancer also differed dramatically across the countries or regions. Specifically, the highest ASIR was recorded in Taiwan (Province of China) with 62.1 (48.9–80.1) per 100 000 person-years, then Monaco, Andorra, Slovakia, and Netherlands (**Figure 1C**; appendix pp 73–76). Greenland had the highest-burden of ASDR of colon and rectum cancer (**Figure 1D**).

### The burden of liver cancer

**Figure 2:**
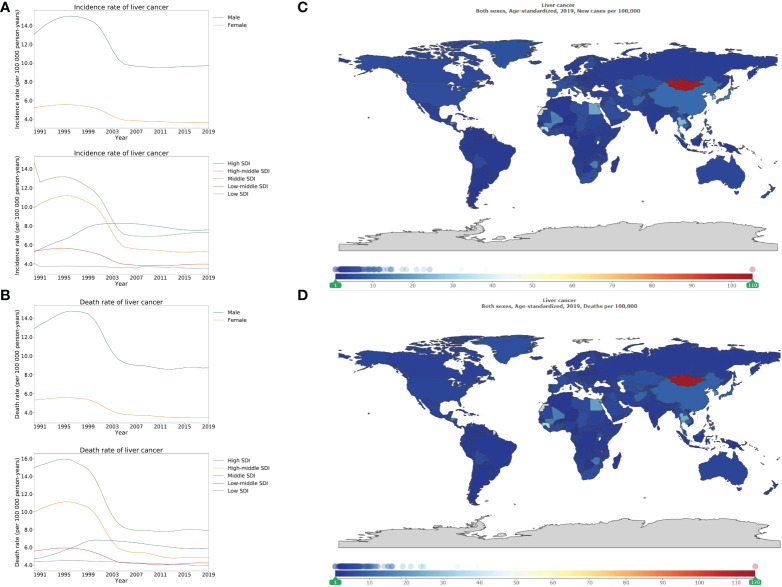
Burden of liver cancer for 204 countries and territories. Age-standardized incidence **(A)** and death **(B)** rate per 100000 population of liver cancer from 1990 through 2019 by country and territory, stratified by sex or SDI; age-standardized incidence **(C)** and death **(D)** rate of liver cancer per 100000 person-years by country and territory, in 2019. The maps in **(C)** and **(D)** were generated by GBD 2019 tool.

Stratified using SDI, the ASIR and ASDR of liver cancer were highest in high, and middle SDI locations compared to those that were lower in high-middle, low-middle, and low SDI locations. There was a declining pattern in the ASIR of liver cancer in high-middle (53.8% [45.1–64.3]), and middle SDI locations (49.7% [41.0–60.3]) compared to the upward trend in high SDI locations (144.5% [130.3–159.8]; **Figure 2A**; appendix p 10). Between 1990 and 2019, the high-middle, middle, and low-middle SDI locations recorded a decreasing trend of the ASDR of liver cancer (**Figure 2B**). In comparison, the highest burden of liver cancer was over 150 times, that of the lowest burden (**Figure 2C**). The countries that demonstrated the highest-burden of ASIR of liver cancer also had the highest burden of ASDR of liver cancer (**Figure 2D**). Finally, for the ASDR of liver cancer, Mongolia possessed the highest-burden (115.2 [91.5–142.5] per 100 000 person-years), followed by Gambia, and Guinea (**Figure 2D**; appendix pp 77–80).

### The burden of stomach cancer

**Figure 3:**
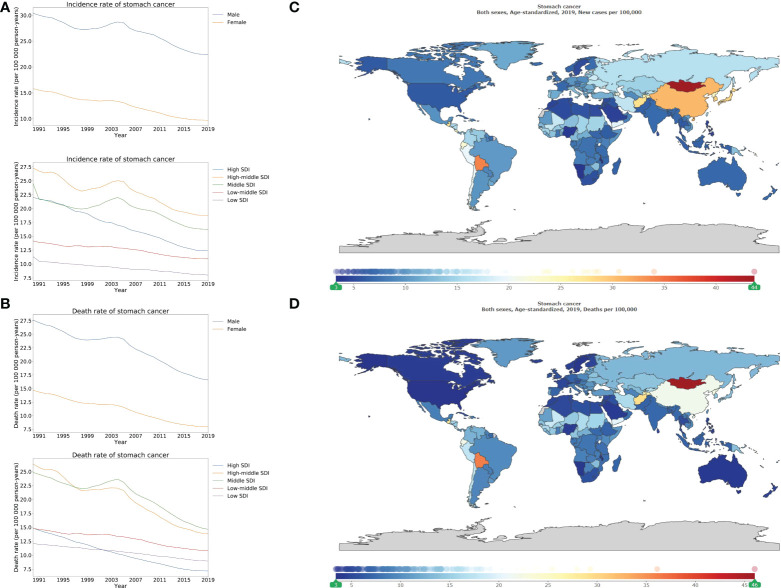
Burden of stomach cancer for 204 countries and territories. Age-standardized incidence **(A)** and death **(B)** rate per 100000 population of stomach cancer from 1990 through 2019 by country and territory, stratified by sex or SDI; age-standardized incidence **(C)** and death **(D)** rate of stomach cancer per 100000 person-years by country and territory, in 2019. The maps in **(C)** and **(D)** were generated by GBD 2019 tool.

In 2019, the ASIR of stomach cancer was 22.4 [19.8–25.3], and 9.7 [8.7–10.7] per 100 000 person-years in male and female, respectively (appendix p 112). Regarding the ASDR of stomach cancer, there were 16.6 [14.8–18.3], and 7.9 [7.1–8.8] per 100 000 person-years in male and female, respectively (appendix p 123). The highest ASIR and ASDR were noted in the high-middle, and middle SDI locations compared to those that were lower in high, low-middle, and low SDI locations (**Figures 3A, B**; appendix pp 113–121; 124–132). In 2019, Mongolia, Bolivia (Plurinational State of), and several East Asia countries such as China, Republic of Korea, and Japan outlined the highest ASIR of stomach cancer, whereas Maldives, Namibia, and Malawi elucidated the lowest ASIR (**Figure 3C**; appendix pp 168–171). Additionally, Mongolia exhibited the highest ASDR (46.4 [36.3–57.5]), followed by Bolivia (36.1 [28.8–44.3]), and Afghanistan (29.3 [21.3–36.5]). Lastly, the lowest ASDRs were recorded in Maldives, Malawi, Kuwait, and United States of America (**Figure 3D**; appendix pp 141–144).

### The burden of esophageal cancer

**Figure 4:**
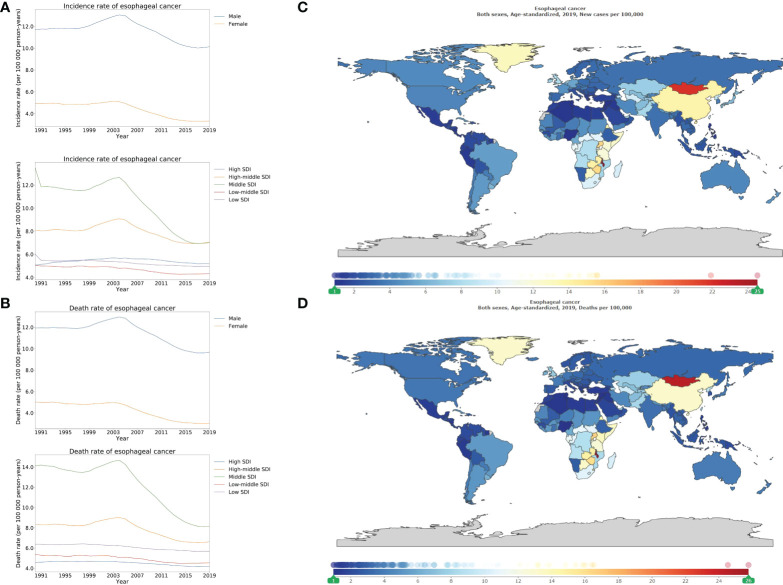
Burden of esophageal cancer for 204 countries and territories. Age-standardized incidence **(A)** and death **(B)** rate per 100000 population of esophageal cancer from 1990 through 2019 by country and territory, stratified by sex or SDI; age-standardized incidence **(C)** and death **(D)** rate of esophageal cancer per 100000 person-years by country and territory, in 2019. The maps in **(C)** and **(D)** were generated by GBD 2019 tool.

Stratified using SDI, the higher ASIRs were revealed in the high-middle, and middle SDI locations compared to those that were lower in high, low-middle and low SDI locations (**Figure 4A**). A similar pattern was also observed for the ASDR (**Figure 4B**). Geographical variance by SDI locations of esophageal cancer corresponded to that of stomach cancer. In 2019, the highest ASIR was 7.1 (5.5–8.2) per 100 000 person-years for high-middle SDI locations while that of high SDI locations was 5.2 (4.7–5.7) per 100 000 person-years. For the ASDR, there were 6.6 (5.3–7.6) per 100 000 person-years for high-middle SDI locations while that of high SDI locations was 4.2 (3.9–4.4) per 100 000 person-years in 2019 (**Figure 4B**; appendix pp 154–162). Remarkably, the highest incidence rates came from Malawi (24.5 [18.7–32.5] per 100 000 person-years), then Mongolia and Uganda. Furthermore, Malawi (25.8 [19.8–33.9] per 100 000 person-years), Mongolia, and Uganda exhibited the highest mortality rates (**Figures 4C, D**; appendix pp 163–166, 167–170).

### The burden of pancreatic cancer

**Figure 5:**
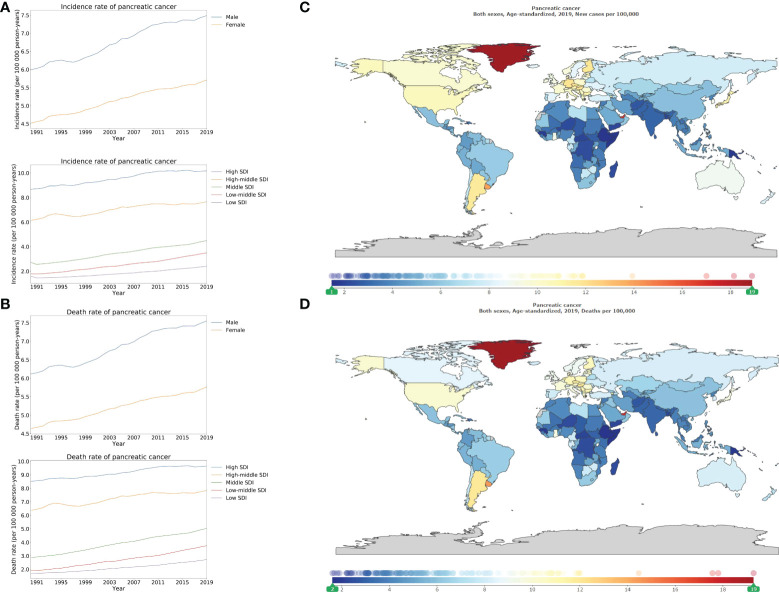
Burden of pancreatic cancer for 204 countries and territories. Age-standardized incidence **(A)** and death **(B)** rate per 100000 population of pancreatic cancer from 1990 through 2019 by country and territory, stratified by sex or SDI; age-standardized incidence **(C)** and death **(D)** rate of pancreatic cancer per 100000 person-years by country and territory, in 2019. The maps in **(C)** and **(D)** were generated by GBD 2019 tool.

Similar to the geographical variance in colon and rectum cancer, the ASIR and ASDR of pancreatic cancer increased along with increases in SDI values. Both the highest ASIR and ASDR were reported in the high SDI locations, followed by the high-middle, middle, low-middle, and low SDI locations, as the decreasing order of SDI (**Figures 5A, B**). In high SDI locations, the ASIR and ASDR were 10.2 (9.1–11.1), and 9.6 (8.8–10.2), per 100 000 person-years, respectively, in 2019. Whilst in low SDI locations, ASIR and ASDR were 2.4 (2.1–2.7), and 2.7 (2.4–3.1), per 100 000 person-years, respectively, in 2019 (appendix pp 173–181, 184–192). The ASIR and ASDR in high SDI locations tripled the rates in low SDI locations. Greenland (18.9 [15.5–22.3]) depicted the highest ASIR, followed by Monaco, United Arab Emirates (**Figure 5C**; appendix pp 193–196). Similar to the order of ASIR, Greenland (19.3 [15.7–22.8]), Monaco, and United Arab Emirates enumerated the highest ASDR (**Figure 5D**; appendix pp 197–200).

## Discussion

### Colon and rectum cancer

According to GLOBOCAN 2018, for both sexes combined, colon and rectum cancer (9.2%) ranks the first leading cause of mortality except lung cancer due to tumors (11). However, the morbidity and mortality of colon and rectum cancer vary greatly between different countries or regions. The incidence rates of colon and rectum cancer in highly developed countries such as North America (12) and Europe (13) is much higher compared to low-developed countries such as Africa and South-Central Asia (14).

According to the data from GBD 2019, high SDI locations exhibited the greatest burden of colon and rectum cancer in the world. By contrast, this study demonstrated that the ASDR of colon and rectum cancer has declined in the past three decades, which agrees with the findings of Arnold et al, which were based on several high HDI countries such as Canada, Denmark, UK, and Singapore (15). Another study by Doubeni et al. (16) noted that a higher prevalence of adverse health behaviors in the populations with low socioeconomic status would further contribute to the socioeconomics related-disparity in the risk of new-onset colon and rectum cancer. Therefore, given the economic resources and healthcare structure, relatively cheaper screening measures such as fecal occult blood tests in these areas may be a feasible strategy to reduce the burden of colon and rectum cancer. Lastly, organized screening and early detection programs, followed by the removal of precancerous polyps, may help to curb the mortality rate of colon and rectum cancer (17).

### Liver cancer

Liver cancer related-risk factors include hepatitis B virus (HBV), hepatitis C virus (HCV), metabolic syndrome, alcohol consumption, diabetes, and so on (18). Globally, HBV accounts for 33% of liver cancer deaths, while alcohol accounts for 30%, HCV for 21%, and other causes for 16% of liver cancer deaths (6). Should be noted, there is profound association between socioeconomics and attributable etiology of liver cancer (19). Unfortunately, treatment options for advanced stages of hepatocellular carcinoma, the most common form of liver cancer, remain scarce (20).

According to the SDI quintile, in all SDI locations except high SDI locations, over 35% burden of the ASDR of liver cancer was caused by hepatitis B virus infection between 1990 and 2015 (6). Considering the heavy burden of liver cancer in middle SDI, and high SDI locations, universal vaccination against the hepatitis B virus in these areas is a feasible strategy to reduce the liver cancer burden. In high SDI, high-middle SDI, and middle SDI locations, which also report the highest-burden of colon and rectum cancer, over 30% burden of liver cancer in terms of ASDR can be attributed to alcohol consumption (6).

### Stomach cancer

Overall, the ASIR and ASDR of stomach cancer have been gradually decreasing in both males and females in the past three decades. Unlike colon and rectum cancer, ASIR and ASDR of stomach cancer in high-middle SDI, and middle SDI locations surpass those of high SDI locations. The reported ASIR is lowest in the low-middle, and low SDI locations. However, this may be caused by inadequate screening since the socioeconomic factor has a critical influence on feasibility on upper endoscopy (21, 22). Population-based upper endoscopy screening, eradication of *Helicobacter pylori* infection, and improvements in food preservation, access to clean water, and household hygiene, would help reduce the global incidence of noncardia stomach cancer, especially in countries with a higher burden, e.g. high-middle SDI, and middle SDI locations.

### Esophageal cancer

Similar to the geographical variance observed in stomach cancer, the ASIR of esophageal cancer, or to be more exact, esophageal squamous cell carcinoma, tends to be higher in the middle, and high-middle SDI rather than high SDI locations, which is consistent with several other studies. Lower socioeconomic status has consistently linked to increased risk of esophageal squamous cell carcinoma (23, 24). Although there is an overall inverse association between socioeconomic status and ASIR of esophageal squamous cell carcinoma (4, 25–27), the higher socioeconomic status has a positive association with esophageal adenocarcinoma (28). It is important to note that the reported ASIR of esophageal cancer is lowest in the low-middle, and low SDI locations, which is similar to that of stomach cancer. Overtly underestimated incidence and mortality of esophageal cancer can be attributed to inadequate upper endoscopy-based screening. Further, an investigation from China has shown that lower socioeconomic status has strong association with elevated risks of esophageal cancer-related deaths (29). In countries with a high incidence of both stomach and esophageal cancer, for example, China and Mongolia, efforts of mass endoscopic screening in the population at high risk might be beneficial for early detection of both cancers.

### Pancreatic cancer

Globally, high-income North America, e.g. Greenland, and the United States, Central Europe, and Eastern Europe have the highest incidence rates of pancreatic cancer. The risk of pancreatic cancer is positively associated with socioeconomic status (11, 30) and resembles the geographical variance in colon and rectum cancer. The high SDI locations have the highest incidence of pancreatic cancer, which gradually decreases with a decrease of SDI stratification. The remarkable geographical variance can be attributed to higher exposure to the well-known or suspected risk factors of pancreatic cancer in high-income countries as well as the scarcity of feasible diagnostic tools in low-income, and middle-income countries. The ageing population and several potential risk factors, including smoking, high fasting plasma glucose, high BMI, may contribute to the higher incidence of pancreatic cancer in high SDI countries (5). Like other major cancers of the gastrointestinal tract, pancreatic cancer also has a predilection for males. However, the ratio of male to female in the incidence of pancreatic cancer is nearly 1.3, which is much lower than those in other major cancers of the gastrointestinal tract, such as 2.8 in liver cancer. Typically, pancreatic cancer is a disease in older people, with age being the most critical risk factor in developing pancreatic cancer. Usually, the actual or projected female life expectancy advantage over men is significant (31, 32). The higher proportion of aging women partially counteracts the impact of other risk factors such as smoking, on the development of pancreatic cancer. As a result, gender disparity in the incidence of pancreatic cancer shrinks. Although pancreatic cancer is one of the deadliest cancers, unfortunately, no data have the benefits of pancreatic cancer screening in asymptomatic adults (33, 34). It is still in need of effective serum or image-based screening for pancreatic cancer.

### Major cancers of the gastrointestinal tract

An understanding of the temporal and geographical trends in five major cancers originating from the gastrointestinal tract is important since these account for over 35% of neoplasms-related deaths. Trends in the burden of major gastrointestinal tract cancers have underwent substantial changes across the world because of the expanding screening programs, including ultrasound, gastroscopy, and colonoscopy, as well as changes in the related risk factors associated with the major gastrointestinal tract cancers.

Overall, four patterns of change arise in the five types of cancers: firstly, both ASIR and ASDR decrease, e.g. in the stomach and esophageal cancer; secondly, ASDR decrease, while ASIR increase, e.g. colon and rectum cancer; thirdly, both ASIR and ASDR remain stable, e.g. liver cancer; fourthly, both ASIR and ASDR increase, e.g. pancreatic cancer (**S. Figures 1A, 1B**).

Strong correlation, positive or negative, exists between socioeconomic status and burden of major gastrointestinal tract cancers. Stratified by SDI locations, significant binary polarization of the tumor burden related to major gastrointestinal tract cancers can be observed. The high SDI and high-middle SDI locations tend to have the highest ASIR and ASDR of colon and rectum cancer, and pancreatic cancer compared with that in low-middle SDI, and low SDI locations have the highest ASIR and ASDR of stomach cancer, and esophageal cancer (**S. Figure 2**).

In clinical practice, colorectal and pancreatic cancer represents a kind of “disease of richness” whereas stomach cancer and esophageal cancer are associated with poverty. Further, it should be noted that almost all the major gastrointestinal tract cancers related to incidence and mortality from low-middle SDI, and low SDI locations have been overtly underestimated. Since lower socioeconomic status remarkably hinders the accessibility and compliance to cancer screening, it is not surprising that the burden of tumors from these areas is seriously underestimated.

Significant gender variance exists in the burden of major gastrointestinal tract cancers. Regarding the incidence of major gastrointestinal tract cancers, males have a higher burden in all these cancers, with the ratios of male to female ranging from 1.3 in pancreatic cancer to 2.8 in liver cancer. Although women have a longer life expectancy, men may have a higher prevalence of cancer-related risk factors such as smoking and alcohol consumption, which leads to a higher burden of cancers in men. This indicates the importance of controlling cancer-related risk factors.

### Limitations

There are several limitations, including the possible underestimation of cancer burden in low-middle SDI, and low SDI locations due to inadequate cancer screening. Second, insufficient revelation about geographical variance in large countries such as USA and China. Cancer burden was reported by country or region in the GBD. However, a large country usually has a significant geographical variance of cancer burden in the urban or rural region. Third, no finer data is available for complex cancer. For example, stomach cancer can be divided into cardia cancer and noncardia cancer whereas esophageal cancer includes adenocarcinoma and squamous cell carcinoma. These subgroups of cancer tend to exhibit different features in terms of ASIR and ASDR. Fourth, racial disparities and inequities in the medical system are not rarely seen worldwide. Colored races may suffer insufficient medical care (35). However, GBD 2019 has no race-related information. Despite these limitations, data of GBD 2019 are valuable to implement cost-effective interventions, address modifiable risks, carry out efficient prevention for gastrointestinal tract cancers.

## Data availability statement

The original contributions presented in the study are included in the article/**Supplementary Material**. Further inquiries can be directed to the corresponding authors.

## Author contributions

J-SP and L-LG had full access to all the data in the study and takes responsibility for the integrity of the data and the accuracy of the data analysis; J-SP and L-LG were responsible for its conception and design; M-ZH, L-LG, J-ML, Z-JC, and X-YL were responsible for the acquisition, analysis, or interpretation of data; J-SP drafted the manuscript; M-ZH and L-LG made critical revision of the manuscript for important intellectual content; J-ML and J-SP conducted the data analysis. All authors contributed to the article and approved the submitted version.

## Funding

Supported by the National Natural Science Foundation of China No. 81871645 (to J-SP), and the Foundation of Department of Science and Technology of Fujian Province No. 2020J011218 (to M-ZH) and No. 2022J02030 (to J-SP).

## Acknowledgments

Editage provided language editing assistance for our manuscript.

## Conflict of interest

The authors declare that the research was conducted in the absence of any commercial or financial relationships that could be construed as a potential conflict of interest.

## Publisher’s note

All claims expressed in this article are solely those of the authors and do not necessarily represent those of their affiliated organizations, or those of the publisher, the editors and the reviewers. Any product that may be evaluated in this article, or claim that may be made by its manufacturer, is not guaranteed or endorsed by the publisher.
