# Supplementary material for: Global burden of major gastrointestinal cancers and its association with socioeconomics, 1990–2019
Source: Front Oncol. 2022 Nov 1;12:942035. doi: 10.3389/fonc.2022.942035 (PMC9664003; doi:10.3389/fonc.2022.942035)
Supplement: Supplementary file 1 [file DataSheet_1.pdf]

GBD_2019_append x

1

Socio-Demographic Index values for all estimated GBD 2019 locations, 1990-2019

Location	1990	1991	1992	1993	1994	1995	1996	1997	1998	1999	2000	2001	2002	2003	2004	2005	2006	2007	2008	2009	2010	2011	2012	2013	2014	2015	2016	2017	2018	2019
Global	0.511	0.516	0.521	0.525	0.529	0.534	0.538	0.542	0.547	0.551	0.556	0.561	0.566	0.571	0.576	0.581	0.586	0.591	0.596	0.601	0.607	0.612	0.616	0.621	0.626	0.631	0.635	0.641	0.647	0.651
Central Europe, eastern Europe, and central Asia	0.648	0.654	0.662	0.666	0.669	0.672	0.675	0.678	0.681	0.684	0.687	0.690	0.694	0.698	0.704	0.710	0.714	0.719	0.724	0.728	0.732	0.735	0.738	0.742	0.745	0.748	0.751	0.754	0.758	0.760
Central Asia	0.551	0.555	0.557	0.558	0.559	0.559	0.560	0.560	0.561	0.563	0.566	0.569	0.574	0.579	0.585	0.591	0.598	0.605	0.611	0.617	0.622	0.627	0.632	0.637	0.642	0.647	0.651	0.655	0.659	0.663
Armenia	0.536	0.541	0.541	0.542	0.544	0.546	0.550	0.554	0.559	0.564	0.570	0.577	0.586	0.596	0.606	0.616	0.626	0.637	0.647	0.652	0.658	0.662	0.666	0.670	0.673	0.676	0.679	0.682	0.686	0.689
Azerbaijan	0.576	0.578	0.579	0.578	0.576	0.573	0.569	0.565	0.561	0.559	0.559	0.561	0.564	0.569	0.575	0.583	0.594	0.607	0.619	0.628	0.637	0.645	0.652	0.658	0.664	0.669	0.673	0.677	0.680	0.683
Georgia	0.654	0.658	0.657	0.654	0.650	0.644	0.638	0.633	0.628	0.625	0.624	0.626	0.630	0.636	0.641	0.646	0.651	0.656	0.660	0.663	0.665	0.668	0.672	0.676	0.680	0.684	0.688	0.693	0.697	0.702
Kazakhstan	0.602	0.606	0.611	0.615	0.619	0.622	0.625	0.628	0.630	0.632	0.635	0.639	0.644	0.649	0.655	0.661	0.667	0.674	0.679	0.683	0.688	0.692	0.696	0.700	0.704	0.708	0.712	0.716	0.720	0.723
Kyrgyzstan	0.532	0.537	0.541	0.543	0.542	0.541	0.539	0.537	0.536	0.534	0.534	0.535	0.537	0.540	0.544	0.546	0.549	0.552	0.555	0.558	0.560	0.563	0.565	0.569	0.574	0.578	0.583	0.588	0.592	0.596
Mongolia	0.465	0.470	0.475	0.480	0.484	0.490	0.495	0.501	0.506	0.512	0.517	0.523	0.528	0.534	0.539	0.545	0.550	0.555	0.560	0.563	0.566	0.570	0.575	0.579	0.584	0.588	0.592	0.597	0.601	0.606
Tajikistan	0.468	0.473	0.474	0.474	0.472	0.468	0.462	0.457	0.451	0.445	0.441	0.440	0.443	0.448	0.456	0.463	0.470	0.477	0.483	0.489	0.495	0.500	0.505	0.511	0.516	0.521	0.526	0.531	0.535	0.539
Turkmenistan	0.548	0.551	0.554	0.557	0.557	0.558	0.558	0.557	0.556	0.557	0.561	0.565	0.570	0.576	0.582	0.588	0.595	0.601	0.606	0.611	0.616	0.622	0.628	0.635	0.642	0.648	0.654	0.660	0.666	0.670
Uzbekistan	0.490	0.492	0.494	0.496	0.498	0.501	0.505	0.510	0.515	0.520	0.525	0.531	0.536	0.541	0.546	0.551	0.556	0.561	0.567	0.572	0.578	0.584	0.590	0.597	0.603	0.609	0.616	0.622	0.627	0.631
Central Europe	0.641	0.647	0.652	0.658	0.665	0.672	0.678	0.683	0.689	0.695	0.702	0.709	0.715	0.720	0.726	0.731	0.736	0.740	0.745	0.750	0.756	0.760	0.764	0.768	0.771	0.775	0.778	0.781	0.785	0.788
Albania	0.540	0.537	0.534	0.533	0.535	0.538	0.544	0.549	0.555	0.561	0.569	0.577	0.585	0.593	0.601	0.608	0.615	0.621	0.627	0.631	0.636	0.640	0.645	0.651	0.658	0.664	0.669	0.674	0.678	0.681
Bosnia and Herzegovina	0.533	0.534	0.532	0.529	0.527	0.528	0.540	0.558	0.576	0.591	0.604	0.616	0.626	0.636	0.644	0.651	0.658	0.665	0.671	0.677	0.682	0.686	0.691	0.695	0.698	0.702	0.706	0.710	0.714	0.718
Bulgaria	0.631	0.641	0.648	0.656	0.666	0.671	0.676	0.681	0.677	0.675	0.680	0.688	0.693	0.697	0.701	0.706	0.710	0.715	0.718	0.724	0.733	0.737	0.740	0.743	0.746	0.750	0.752	0.755	0.760	0.764
Croatia	0.680	0.688	0.692	0.692	0.692	0.691	0.691	0.697	0.703	0.707	0.713	0.719	0.725	0.730	0.734	0.739	0.745	0.748	0.753	0.758	0.763	0.767	0.770	0.774	0.777	0.781	0.784	0.788	0.791	0.794
Czech Republic	0.688	0.696	0.705	0.718	0.736	0.748	0.755	0.760	0.765	0.771	0.776	0.782	0.786	0.790	0.794	0.798	0.801	0.804	0.807	0.810	0.813	0.816	0.818	0.819	0.820	0.820	0.820	0.822	0.825	0.828
Hungary	0.659	0.663	0.671	0.678	0.685	0.693	0.700	0.707	0.713	0.718	0.724	0.730	0.735	0.741	0.746	0.751	0.756	0.760	0.763	0.768	0.772	0.773	0.774	0.774	0.775	0.778	0.781	0.784	0.788	0.791
Montenegro	0.701	0.701	0.699	0.695	0.690	0.687	0.686	0.687	0.690	0.692	0.696	0.701	0.706	0.712	0.717	0.723	0.729	0.736	0.743	0.749	0.754	0.759	0.764	0.768	0.773	0.777	0.780	0.784	0.788	0.791
North Macedonia	0.618	0.620	0.623	0.625	0.627	0.631	0.635	0.640	0.646	0.651	0.656	0.662	0.668	0.674	0.679	0.684	0.689	0.694	0.700	0.704	0.709	0.713	0.717	0.722	0.726	0.730	0.734	0.738	0.741	0.744
Poland	0.632	0.637	0.644	0.653	0.661	0.670	0.677	0.685	0.693	0.701	0.709	0.717	0.724	0.730	0.735	0.740	0.743	0.747	0.752	0.757	0.763	0.770	0.775	0.780	0.784	0.788	0.791	0.795	0.798	0.802
Romania	0.625	0.632	0.635	0.638	0.643	0.649	0.653	0.655	0.659	0.664	0.669	0.677	0.682	0.686	0.693	0.698	0.702	0.707	0.711	0.718	0.726	0.729	0.734	0.740	0.741	0.744	0.747	0.752	0.756	0.760
Serbia	0.626	0.635	0.639	0.639	0.640	0.640	0.644	0.647	0.651	0.657	0.661	0.665	0.670	0.676	0.685	0.694	0.702	0.709	0.716	0.723	0.729	0.735	0.739	0.744	0.748	0.753	0.756	0.760	0.763	0.767
Slovakia	0.656	0.662	0.668	0.679	0.693	0.702	0.709	0.716	0.724	0.731	0.739	0.746	0.752	0.756	0.760	0.766	0.772	0.777	0.781	0.784	0.789	0.794	0.798	0.801	0.803	0.803	0.804	0.805	0.808	0.812
Slovenia	0.726	0.731	0.736	0.741	0.746	0.751	0.756	0.762	0.768	0.774	0.780	0.787	0.793	0.797	0.802	0.807	0.811	0.814	0.818	0.820	0.822	0.824	0.825	0.827	0.829	0.831	0.833	0.835	0.838	0.840
Eastern Europe	0.680	0.687	0.697	0.702	0.702	0.705	0.707	0.708	0.709	0.711	0.711	0.713	0.716	0.720	0.727	0.734	0.740	0.745	0.751	0.757	0.762	0.765	0.768	0.772	0.777	0.781	0.785	0.788	0.791	0.793
Belarus	0.591	0.595	0.600	0.606	0.611	0.614	0.618	0.620	0.621	0.624	0.629	0.635	0.642	0.650	0.658	0.665	0.671	0.678	0.687	0.695	0.703	0.709	0.713	0.719	0.725	0.730	0.734	0.738	0.742	0.745
Estonia	0.665	0.676	0.687	0.696	0.700	0.705	0.711	0.717	0.721	0.726	0.733	0.741	0.748	0.753	0.759	0.765	0.771	0.777	0.785	0.792	0.798	0.804	0.809	0.813	0.817	0.821	0.825	0.829	0.833	0.835
Latvia	0.675	0.682	0.691	0.700	0.708	0.713	0.716	0.719	0.721	0.723	0.727	0.733	0.739	0.745	0.753	0.760	0.766	0.774	0.784	0.793	0.797	0.798	0.801	0.803	0.804	0.805	0.809	0.813	0.817	0.820
Lithuania	0.670	0.672	0.682	0.691	0.694	0.696	0.700	0.705	0.709	0.714	0.723	0.730	0.736	0.743	0.752	0.760	0.765	0.771	0.782	0.792	0.797	0.801	0.808	0.813	0.817	0.822	0.829	0.835	0.839	0.843
Moldova	0.585	0.589	0.591	0.594	0.594	0.595	0.594	0.593	0.591	0.587	0.585	0.585	0.588	0.593	0.600	0.607	0.615	0.622	0.630	0.637	0.644	0.651	0.658	0.665	0.672	0.677	0.683	0.688	0.693	0.696

Socio-Demographic Index values for all estimated GBD 2019 locations, 1990-2019

Location	1990	1991	1992	1993	1994	1995	1996	1997	1998	1999	2000	2001	2002	2003	2004	2005	2006	2007	2008	2009	2010	2011	2012	2013	2014	2015	2016	2017	2018	2019
Russia	0.695	0.703	0.716	0.720	0.719	0.722	0.724	0.725	0.726	0.728	0.728	0.728	0.730	0.734	0.741	0.749	0.754	0.759	0.764	0.770	0.775	0.777	0.779	0.784	0.788	0.793	0.797	0.801	0.803	0.805
Ukraine	0.653	0.657	0.661	0.665	0.666	0.667	0.667	0.667	0.666	0.665	0.664	0.665	0.668	0.672	0.679	0.685	0.692	0.699	0.706	0.710	0.713	0.718	0.721	0.725	0.727	0.729	0.730	0.732	0.734	0.736
High-income	0.755	0.760	0.765	0.769	0.773	0.777	0.780	0.783	0.786	0.788	0.791	0.795	0.799	0.801	0.804	0.806	0.807	0.809	0.812	0.816	0.820	0.823	0.826	0.829	0.832	0.835	0.839	0.842	0.845	0.847
Australasia	0.742	0.746	0.749	0.753	0.757	0.761	0.765	0.769	0.773	0.777	0.781	0.785	0.789	0.793	0.797	0.799	0.799	0.800	0.803	0.807	0.810	0.812	0.816	0.821	0.825	0.828	0.832	0.835	0.837	0.840
Australia	0.738	0.741	0.745	0.749	0.753	0.757	0.761	0.766	0.770	0.774	0.778	0.782	0.787	0.791	0.795	0.797	0.798	0.799	0.802	0.806	0.809	0.812	0.815	0.820	0.824	0.828	0.832	0.834	0.837	0.839
New Zealand	0.757	0.762	0.765	0.769	0.772	0.774	0.778	0.782	0.785	0.787	0.790	0.794	0.796	0.798	0.802	0.803	0.800	0.800	0.803	0.807	0.809	0.812	0.816	0.821	0.825	0.828	0.832	0.835	0.838	0.840
High-income Asia Pacific	0.767	0.773	0.779	0.785	0.790	0.796	0.801	0.805	0.809	0.813	0.816	0.819	0.823	0.826	0.830	0.833	0.836	0.839	0.842	0.844	0.847	0.850	0.853	0.856	0.859	0.862	0.865	0.868	0.871	0.873
Brunei	0.676	0.682	0.688	0.694	0.700	0.706	0.712	0.717	0.723	0.729	0.735	0.741	0.747	0.753	0.758	0.764	0.769	0.774	0.779	0.784	0.789	0.793	0.797	0.801	0.806	0.809	0.813	0.817	0.820	0.823
Japan	0.791	0.796	0.801	0.805	0.809	0.813	0.817	0.820	0.822	0.824	0.826	0.828	0.830	0.833	0.836	0.838	0.840	0.842	0.844	0.846	0.848	0.850	0.853	0.855	0.857	0.860	0.862	0.865	0.867	0.870
South Korea	0.686	0.697	0.708	0.719	0.730	0.740	0.750	0.760	0.767	0.776	0.784	0.791	0.799	0.805	0.811	0.817	0.823	0.828	0.833	0.837	0.842	0.846	0.851	0.855	0.859	0.863	0.867	0.871	0.875	0.878
Singapore	0.688	0.697	0.705	0.714	0.723	0.731	0.740	0.749	0.756	0.762	0.769	0.776	0.783	0.789	0.794	0.801	0.808	0.814	0.822	0.828	0.835	0.839	0.843	0.847	0.850	0.852	0.855	0.858	0.860	0.861
High-income North America	0.771	0.773	0.777	0.780	0.784	0.787	0.790	0.792	0.794	0.797	0.800	0.805	0.809	0.811	0.814	0.815	0.814	0.817	0.822	0.828	0.834	0.837	0.841	0.844	0.847	0.850	0.854	0.857	0.859	0.860
Canada	0.790	0.792	0.795	0.797	0.800	0.804	0.809	0.812	0.815	0.819	0.824	0.828	0.832	0.835	0.838	0.840	0.842	0.843	0.845	0.848	0.851	0.853	0.856	0.859	0.861	0.864	0.867	0.869	0.871	0.873
Greenland	0.655	0.652	0.651	0.651	0.653	0.653	0.653	0.655	0.661	0.664	0.667	0.671	0.678	0.684	0.689	0.696	0.704	0.710	0.715	0.721	0.728	0.734	0.737	0.740	0.743	0.747	0.751	0.756	0.759	0.761
USA	0.768	0.771	0.775	0.778	0.782	0.785	0.788	0.789	0.791	0.794	0.797	0.802	0.806	0.809	0.811	0.812	0.811	0.814	0.819	0.826	0.832	0.835	0.839	0.842	0.845	0.849	0.853	0.856	0.858	0.859
Southern Latin America	0.584	0.589	0.597	0.602	0.608	0.614	0.620	0.625	0.630	0.634	0.640	0.644	0.648	0.651	0.653	0.658	0.663	0.664	0.667	0.671	0.676	0.681	0.686	0.689	0.692	0.701	0.710	0.716	0.719	0.721
Argentina	0.581	0.585	0.593	0.599	0.605	0.611	0.617	0.622	0.625	0.628	0.634	0.637	0.640	0.641	0.642	0.649	0.653	0.655	0.657	0.661	0.665	0.670	0.674	0.677	0.679	0.687	0.696	0.702	0.706	0.708
Chile	0.592	0.600	0.606	0.611	0.617	0.624	0.630	0.637	0.644	0.651	0.657	0.663	0.671	0.678	0.683	0.686	0.689	0.692	0.695	0.700	0.706	0.712	0.719	0.724	0.728	0.738	0.747	0.753	0.756	0.759
Uruguay	0.581	0.584	0.588	0.591	0.594	0.597	0.600	0.606	0.612	0.618	0.622	0.626	0.628	0.631	0.633	0.636	0.639	0.642	0.645	0.649	0.653	0.658	0.663	0.668	0.673	0.678	0.684	0.688	0.693	0.697
Western Europe	0.750	0.756	0.762	0.767	0.772	0.775	0.779	0.782	0.784	0.787	0.790	0.794	0.797	0.800	0.802	0.805	0.807	0.810	0.812	0.815	0.817	0.821	0.824	0.827	0.830	0.832	0.835	0.838	0.841	0.843
Andorra	0.834	0.838	0.840	0.841	0.841	0.841	0.843	0.845	0.847	0.849	0.851	0.854	0.855	0.859	0.862	0.865	0.867	0.869	0.872	0.874	0.876	0.879	0.881	0.883	0.885	0.887	0.889	0.891	0.892	0.894
Austria	0.753	0.754	0.757	0.761	0.768	0.773	0.778	0.783	0.787	0.791	0.795	0.799	0.803	0.805	0.808	0.811	0.815	0.818	0.821	0.824	0.826	0.830	0.833	0.835	0.838	0.839	0.841	0.844	0.847	0.849
Belgium	0.746	0.750	0.756	0.762	0.767	0.771	0.775	0.779	0.782	0.784	0.787	0.792	0.796	0.799	0.802	0.805	0.808	0.810	0.813	0.816	0.820	0.824	0.829	0.834	0.837	0.841	0.843	0.846	0.849	0.851
Cyprus	0.662	0.670	0.680	0.691	0.702	0.713	0.723	0.732	0.741	0.750	0.758	0.767	0.774	0.780	0.786	0.791	0.797	0.804	0.810	0.816	0.820	0.824	0.827	0.829	0.831	0.832	0.834	0.836	0.838	0.841
Denmark	0.806	0.809	0.813	0.816	0.820	0.824	0.828	0.833	0.836	0.840	0.844	0.848	0.852	0.855	0.858	0.860	0.862	0.864	0.865	0.867	0.870	0.873	0.875	0.878	0.880	0.882	0.884	0.886	0.888	0.890
Finland	0.757	0.759	0.762	0.765	0.769	0.773	0.777	0.782	0.785	0.788	0.792	0.797	0.802	0.805	0.808	0.812	0.815	0.818	0.821	0.824	0.828	0.831	0.834	0.837	0.840	0.844	0.848	0.851	0.853	0.856
France	0.738	0.743	0.750	0.755	0.759	0.763	0.767	0.770	0.773	0.775	0.777	0.781	0.785	0.787	0.790	0.793	0.796	0.799	0.801	0.803	0.806	0.809	0.812	0.815	0.819	0.822	0.826	0.829	0.832	0.834
Germany	0.819	0.830	0.834	0.838	0.841	0.843	0.844	0.844	0.844	0.844	0.847	0.853	0.856	0.858	0.861	0.863	0.866	0.869	0.873	0.875	0.878	0.881	0.883	0.886	0.888	0.890	0.892	0.894	0.896	0.898
Greece	0.682	0.688	0.695	0.702	0.707	0.713	0.717	0.723	0.729	0.735	0.740	0.743	0.748	0.753	0.758	0.761	0.765	0.768	0.771	0.775	0.779	0.782	0.785	0.786	0.786	0.787	0.788	0.790	0.792	0.794
Iceland	0.764	0.770	0.774	0.778	0.782	0.785	0.788	0.790	0.794	0.799	0.806	0.813	0.818	0.822	0.824	0.827	0.830	0.834	0.838	0.842	0.846	0.847	0.847	0.848	0.850	0.854	0.858	0.863	0.866	0.869
Ireland	0.730	0.735	0.741	0.747	0.753	0.758	0.763	0.768	0.774	0.780	0.786	0.793	0.799	0.806	0.812	0.816	0.819	0.821	0.824	0.827	0.831	0.835	0.839	0.842	0.845	0.850	0.854	0.859	0.864	0.867
Israel	0.717	0.721	0.726	0.730	0.734	0.738	0.742	0.745	0.749	0.752	0.756	0.760	0.762	0.765	0.769	0.773	0.776	0.778	0.778	0.779	0.781	0.784	0.787	0.790	0.792	0.794	0.796	0.798	0.800	0.803
Italy	0.712	0.717	0.722	0.727	0.732	0.737	0.740	0.744	0.747	0.750	0.753	0.757	0.761	0.763	0.766	0.768	0.771	0.773	0.775	0.777	0.780	0.782	0.784	0.787	0.789	0.791	0.794	0.796	0.798	0.801
Luxembourg	0.815	0.818	0.820	0.823	0.828	0.833	0.836	0.839	0.842	0.844	0.847	0.850	0.853	0.855	0.857	0.858	0.862	0.866	0.869	0.871	0.872	0.874	0.877	0.880	0.883	0.886	0.889	0.892	0.894	0.895

GBD_2019_appendix

Socio-Demographic Index values for all estimated GBD 2019 locations, 1990-2019

Location	1990	1991	1992	1993	1994	1995	1996	1997	1998	1999	2000	2001	2002	2003	2004	2005	2006	2007	2008	2009	2010	2011	2012	2013	2014	2015	2016	2017	2018	2019
Malta	0.666	0.670	0.675	0.682	0.690	0.695	0.696	0.700	0.708	0.715	0.722	0.729	0.733	0.737	0.741	0.745	0.749	0.753	0.757	0.761	0.764	0.768	0.772	0.775	0.779	0.784	0.788	0.793	0.797	0.801
Monaco	0.834	0.837	0.840	0.843	0.846	0.849	0.852	0.855	0.857	0.860	0.862	0.865	0.867	0.870	0.872	0.875	0.877	0.879	0.881	0.883	0.886	0.888	0.890	0.892	0.893	0.895	0.897	0.899	0.901	0.902
Netherlands	0.796	0.801	0.806	0.810	0.814	0.818	0.821	0.824	0.827	0.830	0.832	0.836	0.839	0.842	0.845	0.848	0.851	0.853	0.856	0.858	0.861	0.864	0.866	0.869	0.871	0.874	0.876	0.878	0.881	0.883
Norway	0.807	0.812	0.818	0.823	0.828	0.832	0.837	0.843	0.847	0.851	0.856	0.861	0.866	0.869	0.872	0.874	0.876	0.877	0.879	0.881	0.885	0.889	0.893	0.896	0.900	0.903	0.907	0.910	0.912	0.913
Portugal	0.607	0.615	0.622	0.629	0.636	0.641	0.647	0.651	0.656	0.661	0.666	0.671	0.676	0.681	0.686	0.690	0.694	0.698	0.701	0.705	0.709	0.714	0.718	0.722	0.726	0.729	0.732	0.736	0.739	0.743
San Marino	0.814	0.817	0.820	0.824	0.828	0.832	0.837	0.841	0.846	0.850	0.852	0.854	0.856	0.859	0.861	0.862	0.864	0.866	0.868	0.870	0.872	0.874	0.876	0.877	0.879	0.881	0.882	0.882	0.883	0.884
Spain	0.647	0.655	0.662	0.669	0.675	0.681	0.686	0.691	0.696	0.700	0.705	0.709	0.713	0.717	0.721	0.725	0.728	0.730	0.734	0.739	0.743	0.746	0.749	0.752	0.754	0.756	0.759	0.761	0.764	0.767
Sweden	0.769	0.775	0.782	0.788	0.794	0.801	0.806	0.811	0.815	0.819	0.823	0.826	0.829	0.832	0.835	0.838	0.840	0.842	0.844	0.846	0.849	0.852	0.855	0.858	0.860	0.863	0.865	0.868	0.870	0.872
Switzerland	0.868	0.869	0.873	0.876	0.878	0.880	0.881	0.883	0.884	0.886	0.889	0.893	0.894	0.896	0.898	0.900	0.902	0.905	0.908	0.909	0.912	0.914	0.917	0.919	0.921	0.922	0.924	0.926	0.928	0.929
UK	0.745	0.749	0.755	0.761	0.766	0.769	0.771	0.774	0.778	0.784	0.789	0.793	0.797	0.799	0.802	0.804	0.806	0.808	0.811	0.813	0.816	0.820	0.825	0.830	0.834	0.837	0.839	0.842	0.845	0.847
Latin America and Caribbean	0.491	0.496	0.501	0.506	0.511	0.516	0.521	0.526	0.531	0.536	0.541	0.546	0.550	0.554	0.558	0.563	0.568	0.573	0.578	0.583	0.589	0.594	0.600	0.605	0.611	0.616	0.620	0.625	0.629	0.633
Andean Latin America	0.489	0.492	0.496	0.500	0.504	0.509	0.514	0.519	0.524	0.529	0.534	0.538	0.543	0.548	0.553	0.558	0.564	0.569	0.575	0.580	0.585	0.591	0.596	0.602	0.608	0.613	0.618	0.624	0.628	0.632
Bolivia	0.412	0.417	0.422	0.428	0.434	0.441	0.448	0.455	0.462	0.469	0.474	0.480	0.485	0.491	0.496	0.501	0.506	0.510	0.514	0.518	0.521	0.525	0.528	0.533	0.538	0.544	0.550	0.556	0.562	0.566
Ecuador	0.503	0.506	0.510	0.514	0.517	0.521	0.524	0.528	0.532	0.535	0.539	0.544	0.549	0.554	0.559	0.565	0.570	0.575	0.581	0.586	0.591	0.597	0.603	0.609	0.615	0.621	0.626	0.632	0.636	0.640
Peru	0.501	0.505	0.508	0.511	0.516	0.521	0.526	0.532	0.537	0.543	0.548	0.552	0.557	0.562	0.566	0.571	0.577	0.583	0.589	0.595	0.601	0.608	0.614	0.620	0.625	0.630	0.635	0.640	0.645	0.648
Caribbean	0.517	0.522	0.525	0.528	0.531	0.533	0.536	0.539	0.542	0.547	0.552	0.557	0.563	0.569	0.573	0.579	0.585	0.588	0.591	0.594	0.598	0.601	0.605	0.609	0.612	0.616	0.621	0.625	0.628	0.631
Antigua and Barbuda	0.579	0.586	0.592	0.598	0.604	0.610	0.616	0.623	0.631	0.638	0.645	0.652	0.659	0.665	0.672	0.679	0.686	0.694	0.700	0.705	0.709	0.713	0.716	0.719	0.723	0.727	0.731	0.735	0.739	0.743
The Bahamas	0.692	0.684	0.676	0.679	0.686	0.692	0.697	0.702	0.710	0.721	0.731	0.735	0.737	0.739	0.742	0.749	0.755	0.759	0.762	0.767	0.774	0.779	0.780	0.782	0.784	0.786	0.789	0.791	0.794	0.796
Barbados	0.649	0.653	0.658	0.663	0.667	0.670	0.672	0.674	0.676	0.677	0.680	0.683	0.687	0.691	0.695	0.699	0.703	0.707	0.711	0.714	0.718	0.721	0.725	0.728	0.730	0.733	0.735	0.737	0.740	0.742
Belize	0.428	0.437	0.447	0.457	0.466	0.475	0.482	0.488	0.494	0.499	0.505	0.510	0.515	0.521	0.528	0.534	0.541	0.547	0.553	0.559	0.564	0.569	0.574	0.579	0.583	0.588	0.592	0.596	0.600	0.603
Bermuda	0.685	0.689	0.693	0.697	0.701	0.704	0.707	0.710	0.714	0.719	0.724	0.731	0.737	0.743	0.749	0.755	0.760	0.766	0.773	0.779	0.785	0.790	0.795	0.799	0.802	0.805	0.807	0.809	0.811	0.813
Cuba	0.578	0.586	0.589	0.588	0.586	0.583	0.583	0.582	0.579	0.581	0.586	0.588	0.595	0.602	0.605	0.611	0.618	0.618	0.616	0.618	0.620	0.624	0.631	0.636	0.640	0.645	0.653	0.660	0.665	0.668
Dominica	0.579	0.585	0.591	0.597	0.604	0.610	0.617	0.623	0.630	0.637	0.643	0.649	0.655	0.660	0.666	0.672	0.677	0.683	0.689	0.695	0.700	0.705	0.709	0.713	0.717	0.721	0.724	0.727	0.728	0.729
Dominican Republic	0.425	0.427	0.430	0.434	0.437	0.442	0.446	0.452	0.458	0.465	0.471	0.478	0.485	0.492	0.499	0.508	0.516	0.524	0.532	0.538	0.544	0.549	0.554	0.559	0.563	0.569	0.574	0.580	0.587	0.592
Grenada	0.463	0.474	0.485	0.495	0.505	0.515	0.524	0.533	0.543	0.553	0.562	0.570	0.577	0.585	0.592	0.600	0.606	0.613	0.619	0.624	0.628	0.633	0.637	0.641	0.645	0.650	0.654	0.659	0.664	0.669
Guyana	0.452	0.456	0.461	0.467	0.474	0.481	0.488	0.496	0.503	0.510	0.516	0.522	0.527	0.532	0.536	0.540	0.544	0.549	0.554	0.559	0.565	0.571	0.577	0.583	0.590	0.596	0.602	0.608	0.614	0.618
Haiti	0.307	0.311	0.314	0.317	0.320	0.323	0.328	0.333	0.338	0.344	0.350	0.356	0.361	0.366	0.371	0.375	0.379	0.383	0.387	0.392	0.395	0.399	0.403	0.407	0.412	0.416	0.420	0.424	0.428	0.432
Jamaica	0.542	0.547	0.553	0.560	0.566	0.573	0.580	0.586	0.592	0.598	0.603	0.609	0.614	0.620	0.625	0.630	0.635	0.640	0.645	0.649	0.653	0.657	0.661	0.664	0.668	0.671	0.675	0.678	0.681	0.684
Puerto Rico	0.670	0.676	0.679	0.684	0.689	0.693	0.699	0.705	0.711	0.714	0.720	0.731	0.738	0.742	0.744	0.748	0.752	0.756	0.760	0.764	0.769	0.774	0.779	0.785	0.793	0.802	0.808	0.811	0.813	0.814
Saint Kitts and Nevis	0.583	0.592	0.599	0.607	0.614	0.620	0.626	0.633	0.638	0.644	0.650	0.657	0.663	0.668	0.673	0.679	0.685	0.690	0.696	0.701	0.706	0.710	0.714	0.719	0.724	0.728	0.733	0.738	0.742	0.746
Saint Lucia	0.483	0.493	0.504	0.514	0.524	0.534	0.543	0.551	0.560	0.568	0.575	0.581	0.587	0.593	0.600	0.606	0.612	0.617	0.623	0.629	0.634	0.639	0.643	0.648	0.652	0.656	0.659	0.663	0.667	0.670
Saint Vincent and the Grenadines	0.462	0.471	0.479	0.487	0.495	0.502	0.509	0.515	0.522	0.528	0.534	0.539	0.545	0.551	0.557	0.562	0.568	0.574	0.580	0.585	0.589	0.593	0.598	0.602	0.606	0.610	0.615	0.619	0.623	0.627
Suriname	0.498	0.503	0.507	0.511	0.513	0.515	0.518	0.523	0.528	0.533	0.538	0.545	0.552	0.560	0.568	0.574	0.579	0.584	0.588	0.593	0.598	0.602	0.607	0.611	0.616	0.620	0.625	0.629	0.633	0.636
Trinidad and Tobago	0.618	0.622	0.626	0.630	0.634	0.639	0.644	0.650	0.656	0.662	0.669	0.675	0.682	0.689	0.696	0.704	0.711	0.718	0.724	0.728	0.732	0.736	0.739	0.742	0.745	0.748	0.751	0.753	0.755	0.757

Socio-Demographic Index values for all estimated GBD 2019 locations, 1990-2019

Location	1990	1991	1992	1993	1994	1995	1996	1997	1998	1999	2000	2001	2002	2003	2004	2005	2006	2007	2008	2009	2010	2011	2012	2013	2014	2015	2016	2017	2018	2019
Virgin Islands	0.667	0.680	0.689	0.697	0.704	0.710	0.715	0.720	0.724	0.728	0.731	0.734	0.742	0.749	0.756	0.762	0.768	0.773	0.778	0.782	0.785	0.788	0.790	0.791	0.792	0.794	0.795	0.796	0.798	0.799
Central Latin America	0.485	0.490	0.495	0.501	0.507	0.512	0.517	0.523	0.528	0.533	0.538	0.543	0.547	0.551	0.554	0.559	0.564	0.569	0.575	0.579	0.584	0.589	0.594	0.599	0.604	0.609	0.614	0.618	0.623	0.626
Colombia	0.478	0.480	0.482	0.486	0.492	0.498	0.503	0.509	0.514	0.518	0.522	0.526	0.529	0.533	0.536	0.540	0.546	0.552	0.559	0.567	0.574	0.582	0.589	0.596	0.603	0.609	0.616	0.622	0.628	0.633
Costa Rica	0.532	0.538	0.544	0.550	0.555	0.560	0.565	0.571	0.577	0.584	0.592	0.598	0.603	0.607	0.611	0.615	0.619	0.623	0.627	0.632	0.637	0.642	0.647	0.652	0.657	0.662	0.667	0.672	0.676	0.680
El Salvador	0.390	0.393	0.397	0.402	0.408	0.415	0.423	0.431	0.441	0.450	0.460	0.469	0.478	0.486	0.493	0.499	0.505	0.511	0.517	0.521	0.526	0.531	0.536	0.542	0.547	0.552	0.558	0.563	0.568	0.573
Guatemala	0.315	0.320	0.328	0.336	0.345	0.352	0.360	0.369	0.378	0.387	0.395	0.403	0.412	0.421	0.430	0.439	0.446	0.453	0.459	0.465	0.472	0.478	0.485	0.491	0.498	0.504	0.510	0.516	0.522	0.526
Honduras	0.330	0.335	0.340	0.345	0.351	0.357	0.363	0.370	0.377	0.384	0.391	0.397	0.404	0.411	0.417	0.424	0.430	0.437	0.443	0.448	0.454	0.459	0.464	0.469	0.473	0.478	0.482	0.487	0.492	0.496
Mexico	0.507	0.514	0.520	0.526	0.532	0.537	0.542	0.547	0.553	0.558	0.563	0.569	0.574	0.578	0.583	0.588	0.592	0.597	0.601	0.605	0.608	0.613	0.617	0.621	0.626	0.631	0.636	0.640	0.645	0.649
Nicaragua	0.338	0.345	0.353	0.360	0.368	0.376	0.385	0.393	0.402	0.411	0.419	0.426	0.433	0.438	0.444	0.449	0.453	0.458	0.462	0.466	0.470	0.474	0.479	0.484	0.489	0.495	0.500	0.506	0.512	0.517
Panama	0.544	0.549	0.555	0.559	0.562	0.565	0.568	0.573	0.579	0.586	0.592	0.597	0.602	0.605	0.607	0.610	0.613	0.616	0.620	0.623	0.627	0.630	0.635	0.642	0.650	0.658	0.666	0.674	0.680	0.686
Venezuela	0.509	0.514	0.522	0.530	0.537	0.542	0.546	0.551	0.554	0.557	0.559	0.560	0.558	0.552	0.548	0.550	0.557	0.566	0.575	0.582	0.586	0.591	0.596	0.600	0.604	0.607	0.608	0.608	0.608	0.607
Tropical Latin America	0.487	0.492	0.497	0.503	0.508	0.513	0.519	0.524	0.529	0.533	0.538	0.543	0.547	0.552	0.556	0.561	0.566	0.572	0.578	0.584	0.590	0.597	0.604	0.610	0.617	0.622	0.627	0.632	0.636	0.640
Brazil	0.487	0.492	0.498	0.503	0.508	0.513	0.519	0.524	0.529	0.533	0.538	0.543	0.547	0.551	0.556	0.561	0.566	0.572	0.577	0.583	0.590	0.597	0.603	0.610	0.616	0.622	0.627	0.632	0.636	0.640
Paraguay	0.465	0.471	0.477	0.484	0.491	0.498	0.505	0.512	0.519	0.524	0.529	0.534	0.539	0.544	0.549	0.554	0.559	0.564	0.570	0.575	0.582	0.588	0.594	0.601	0.608	0.615	0.621	0.627	0.633	0.638
North Africa and Middle East	0.414	0.423	0.432	0.441	0.450	0.460	0.469	0.478	0.487	0.497	0.506	0.515	0.524	0.532	0.541	0.550	0.560	0.569	0.578	0.587	0.595	0.603	0.611	0.619	0.626	0.633	0.640	0.647	0.654	0.660
North Africa and Middle East	0.414	0.423	0.432	0.441	0.450	0.460	0.469	0.478	0.487	0.497	0.506	0.515	0.524	0.532	0.541	0.550	0.560	0.569	0.578	0.587	0.595	0.603	0.611	0.619	0.626	0.633	0.640	0.647	0.654	0.660
Afghanistan	0.187	0.191	0.195	0.196	0.194	0.194	0.193	0.192	0.190	0.189	0.188	0.188	0.194	0.202	0.209	0.216	0.224	0.234	0.243	0.253	0.264	0.274	0.285	0.295	0.304	0.313	0.321	0.329	0.337	0.343
Algeria	0.436	0.446	0.456	0.465	0.474	0.483	0.492	0.500	0.509	0.518	0.526	0.534	0.542	0.550	0.558	0.566	0.573	0.580	0.587	0.593	0.599	0.605	0.611	0.617	0.623	0.628	0.634	0.640	0.646	0.652
Bahrain	0.553	0.560	0.566	0.573	0.580	0.587	0.595	0.602	0.610	0.618	0.626	0.634	0.643	0.653	0.662	0.672	0.680	0.688	0.697	0.705	0.711	0.715	0.720	0.726	0.731	0.735	0.739	0.743	0.747	0.751
Egypt	0.403	0.415	0.426	0.437	0.448	0.459	0.469	0.478	0.487	0.496	0.504	0.512	0.520	0.527	0.535	0.542	0.549	0.557	0.565	0.573	0.582	0.591	0.600	0.609	0.617	0.626	0.635	0.643	0.651	0.658
Iran	0.404	0.419	0.433	0.446	0.457	0.468	0.477	0.486	0.496	0.506	0.517	0.529	0.541	0.552	0.562	0.571	0.581	0.593	0.603	0.613	0.622	0.630	0.635	0.640	0.645	0.649	0.654	0.659	0.665	0.670
Iraq	0.392	0.397	0.402	0.407	0.412	0.417	0.422	0.429	0.439	0.451	0.462	0.475	0.485	0.493	0.504	0.514	0.525	0.535	0.547	0.558	0.570	0.583	0.597	0.610	0.622	0.632	0.644	0.654	0.663	0.671
Jordan	0.520	0.529	0.537	0.546	0.554	0.562	0.570	0.577	0.585	0.592	0.600	0.607	0.614	0.621	0.630	0.639	0.648	0.656	0.664	0.673	0.681	0.688	0.695	0.702	0.707	0.713	0.718	0.723	0.727	0.731
Kuwait	0.655	0.659	0.662	0.667	0.673	0.680	0.689	0.699	0.709	0.717	0.724	0.729	0.735	0.742	0.750	0.760	0.769	0.777	0.785	0.793	0.801	0.808	0.815	0.822	0.828	0.834	0.839	0.844	0.848	0.851
Lebanon	0.462	0.470	0.477	0.485	0.493	0.502	0.511	0.520	0.530	0.540	0.548	0.557	0.565	0.574	0.582	0.591	0.600	0.609	0.618	0.628	0.639	0.649	0.660	0.670	0.677	0.685	0.691	0.698	0.704	0.708
Libya	0.405	0.422	0.438	0.455	0.472	0.489	0.506	0.522	0.538	0.552	0.566	0.580	0.593	0.606	0.619	0.632	0.645	0.658	0.670	0.681	0.691	0.695	0.703	0.707	0.707	0.707	0.705	0.705	0.707	0.709
Morocco	0.347	0.354	0.361	0.367	0.374	0.380	0.386	0.392	0.398	0.403	0.409	0.414	0.420	0.426	0.432	0.439	0.445	0.452	0.460	0.467	0.475	0.483	0.491	0.499	0.508	0.516	0.524	0.533	0.541	0.548
Oman	0.441	0.455	0.469	0.485	0.500	0.514	0.528	0.543	0.558	0.573	0.588	0.601	0.615	0.628	0.641	0.653	0.664	0.678	0.692	0.704	0.715	0.726	0.737	0.747	0.754	0.760	0.767	0.773	0.778	0.783
Palestine	0.314	0.320	0.329	0.337	0.347	0.356	0.365	0.375	0.386	0.397	0.407	0.415	0.422	0.430	0.439	0.449	0.458	0.467	0.476	0.486	0.497	0.509	0.521	0.533	0.543	0.553	0.564	0.573	0.582	0.588
Qatar	0.585	0.598	0.610	0.621	0.632	0.643	0.654	0.666	0.676	0.685	0.694	0.703	0.711	0.719	0.727	0.735	0.743	0.750	0.757	0.764	0.772	0.779	0.786	0.793	0.799	0.806	0.812	0.818	0.825	0.830
Saudi Arabia	0.480	0.491	0.504	0.516	0.529	0.541	0.554	0.566	0.578	0.590	0.602	0.614	0.625	0.637	0.650	0.664	0.677	0.690	0.703	0.715	0.726	0.738	0.750	0.760	0.769	0.778	0.786	0.793	0.800	0.805
Sudan	0.227	0.234	0.241	0.248	0.256	0.263	0.271	0.280	0.289	0.298	0.308	0.318	0.327	0.337	0.348	0.358	0.370	0.381	0.393	0.404	0.416	0.428	0.440	0.451	0.462	0.474	0.485	0.497	0.507	0.515
Syria	0.367	0.376	0.387	0.398	0.409	0.421	0.432	0.443	0.454	0.465	0.476	0.486	0.497	0.508	0.521	0.535	0.548	0.560	0.572	0.583	0.594	0.601	0.605	0.606	0.607	0.608	0.610	0.613	0.616	0.619
Tunisia	0.434	0.444	0.455	0.466	0.476	0.487	0.498	0.508	0.518	0.528	0.538	0.548	0.556	0.565	0.574	0.582	0.591	0.599	0.607	0.614	0.622	0.628	0.634	0.640	0.646	0.651	0.657	0.662	0.667	0.672

GBD_2019_append.x

GBD_2019_append x

Socio-Demographic Index values for all estimated GBD 2019 locations, 1990-2019

Location	1990	1991	1992	1993	1994	1995	1996	1997	1998	1999	2000	2001	2002	2003	2004	2005	2006	2007	2008	2009	2010	2011	2012	2013	2014	2015	2016	2017	2018	2019
Turkey	0.473	0.483	0.493	0.504	0.513	0.523	0.534	0.545	0.556	0.566	0.577	0.587	0.597	0.607	0.619	0.630	0.641	0.652	0.663	0.671	0.680	0.689	0.698	0.707	0.715	0.723	0.729	0.736	0.743	0.748
United Arab Emirates	0.621	0.637	0.653	0.669	0.685	0.700	0.714	0.727	0.740	0.751	0.762	0.773	0.783	0.792	0.801	0.810	0.819	0.829	0.838	0.846	0.853	0.859	0.863	0.868	0.870	0.872	0.874	0.876	0.879	0.880
Yemen	0.176	0.183	0.191	0.198	0.207	0.215	0.224	0.234	0.243	0.253	0.263	0.273	0.283	0.293	0.303	0.314	0.325	0.335	0.346	0.356	0.366	0.375	0.384	0.393	0.402	0.407	0.410	0.412	0.413	0.412
South Asia	0.313	0.319	0.325	0.331	0.337	0.344	0.351	0.357	0.364	0.371	0.378	0.385	0.391	0.398	0.405	0.413	0.421	0.430	0.438	0.447	0.456	0.465	0.475	0.485	0.495	0.505	0.515	0.525	0.535	0.543
South Asia	0.313	0.319	0.325	0.331	0.337	0.344	0.351	0.357	0.364	0.371	0.378	0.385	0.391	0.398	0.405	0.413	0.421	0.430	0.438	0.447	0.456	0.465	0.475	0.485	0.495	0.505	0.515	0.525	0.535	0.543
Bangladesh	0.267	0.275	0.283	0.290	0.297	0.304	0.311	0.317	0.324	0.330	0.336	0.343	0.349	0.355	0.362	0.369	0.376	0.384	0.392	0.400	0.408	0.416	0.425	0.433	0.441	0.449	0.457	0.466	0.475	0.483
Bhutan	0.228	0.232	0.237	0.243	0.251	0.258	0.266	0.275	0.282	0.290	0.298	0.306	0.314	0.322	0.330	0.338	0.347	0.356	0.365	0.375	0.384	0.394	0.403	0.411	0.419	0.426	0.434	0.442	0.449	0.455
India	0.327	0.333	0.339	0.345	0.351	0.358	0.364	0.371	0.378	0.386	0.393	0.400	0.407	0.414	0.421	0.429	0.437	0.446	0.455	0.463	0.473	0.483	0.493	0.504	0.515	0.526	0.537	0.547	0.558	0.566
Nepal	0.198	0.203	0.208	0.215	0.221	0.228	0.236	0.244	0.251	0.259	0.267	0.276	0.284	0.291	0.299	0.307	0.315	0.322	0.330	0.339	0.347	0.356	0.365	0.373	0.382	0.391	0.399	0.408	0.416	0.422
Pakistan	0.247	0.253	0.259	0.265	0.271	0.277	0.283	0.289	0.295	0.301	0.307	0.313	0.320	0.326	0.333	0.340	0.347	0.355	0.363	0.371	0.379	0.387	0.394	0.402	0.410	0.418	0.426	0.434	0.442	0.449
Southeast Asia, east Asia, and Oceania	0.452	0.460	0.468	0.476	0.485	0.494	0.503	0.511	0.519	0.526	0.533	0.541	0.548	0.556	0.563	0.571	0.580	0.589	0.597	0.605	0.614	0.622	0.629	0.636	0.643	0.647	0.651	0.659	0.667	0.673
East Asia	0.447	0.456	0.464	0.473	0.483	0.492	0.502	0.511	0.520	0.528	0.537	0.545	0.554	0.562	0.571	0.580	0.590	0.600	0.609	0.618	0.628	0.637	0.644	0.652	0.660	0.662	0.665	0.675	0.684	0.691
China	0.433	0.441	0.450	0.459	0.469	0.479	0.489	0.499	0.508	0.516	0.525	0.534	0.543	0.552	0.561	0.571	0.581	0.591	0.601	0.611	0.621	0.631	0.638	0.646	0.654	0.657	0.659	0.669	0.679	0.686
North Korea	0.431	0.436	0.439	0.442	0.445	0.447	0.450	0.451	0.452	0.455	0.458	0.463	0.467	0.473	0.478	0.485	0.490	0.496	0.502	0.507	0.513	0.518	0.523	0.528	0.534	0.538	0.543	0.548	0.553	0.558
Taiwan (province of China)	0.667	0.678	0.685	0.694	0.702	0.711	0.719	0.731	0.743	0.747	0.754	0.763	0.772	0.779	0.787	0.795	0.802	0.810	0.817	0.824	0.830	0.833	0.838	0.843	0.848	0.852	0.856	0.860	0.865	0.868
Oceania	0.383	0.385	0.388	0.391	0.394	0.397	0.400	0.403	0.405	0.408	0.410	0.412	0.413	0.414	0.416	0.417	0.419	0.421	0.422	0.424	0.426	0.428	0.431	0.433	0.436	0.440	0.443	0.446	0.449	0.452
American Samoa	0.606	0.609	0.613	0.616	0.619	0.623	0.627	0.630	0.634	0.637	0.641	0.645	0.649	0.652	0.656	0.660	0.663	0.667	0.671	0.674	0.678	0.682	0.686	0.690	0.694	0.698	0.702	0.706	0.709	0.712
Cook Islands	0.625	0.631	0.637	0.643	0.649	0.654	0.659	0.663	0.667	0.671	0.676	0.682	0.687	0.692	0.697	0.701	0.706	0.711	0.715	0.719	0.722	0.726	0.730	0.734	0.739	0.744	0.750	0.755	0.760	0.764
Fiji	0.527	0.532	0.538	0.544	0.550	0.555	0.561	0.567	0.572	0.577	0.582	0.587	0.592	0.596	0.601	0.605	0.609	0.612	0.616	0.619	0.622	0.626	0.630	0.635	0.639	0.644	0.649	0.654	0.659	0.664
Guam	0.693	0.688	0.684	0.684	0.688	0.695	0.704	0.715	0.728	0.741	0.753	0.762	0.767	0.768	0.770	0.771	0.773	0.775	0.778	0.782	0.785	0.789	0.792	0.795	0.797	0.799	0.803	0.807	0.810	0.813
Kiribati	0.425	0.427	0.429	0.432	0.435	0.438	0.441	0.444	0.447	0.451	0.455	0.459	0.463	0.468	0.472	0.476	0.480	0.484	0.487	0.490	0.492	0.495	0.497	0.501	0.504	0.509	0.514	0.518	0.523	0.527
Marshall Islands	0.398	0.404	0.410	0.416	0.423	0.430	0.435	0.439	0.442	0.446	0.450	0.454	0.458	0.463	0.467	0.472	0.477	0.482	0.487	0.493	0.498	0.504	0.509	0.515	0.520	0.525	0.531	0.536	0.541	0.544
Federated States of Micronesia	0.447	0.453	0.459	0.465	0.471	0.478	0.483	0.488	0.492	0.497	0.502	0.507	0.511	0.516	0.521	0.525	0.530	0.534	0.538	0.542	0.546	0.550	0.554	0.558	0.561	0.565	0.569	0.573	0.577	0.580
Nauru	0.499	0.501	0.503	0.504	0.504	0.505	0.505	0.504	0.504	0.503	0.503	0.503	0.503	0.503	0.504	0.506	0.509	0.510	0.515	0.521	0.529	0.538	0.547	0.559	0.573	0.585	0.595	0.605	0.613	0.618
Niue	0.566	0.571	0.576	0.581	0.586	0.591	0.595	0.600	0.605	0.609	0.614	0.618	0.622	0.626	0.631	0.638	0.645	0.651	0.657	0.663	0.669	0.675	0.681	0.685	0.690	0.695	0.699	0.703	0.707	0.711
Northern Mariana Islands	0.692	0.698	0.704	0.709	0.714	0.718	0.722	0.725	0.727	0.731	0.738	0.744	0.748	0.750	0.751	0.752	0.752	0.753	0.753	0.752	0.751	0.750	0.750	0.750	0.750	0.751	0.756	0.761	0.767	0.771
Palau	0.621	0.629	0.636	0.642	0.647	0.652	0.658	0.663	0.668	0.672	0.676	0.680	0.683	0.686	0.690	0.693	0.697	0.700	0.703	0.705	0.707	0.710	0.714	0.717	0.720	0.725	0.729	0.732	0.735	0.738
Papua New Guinea	0.292	0.295	0.299	0.304	0.309	0.313	0.317	0.320	0.323	0.326	0.329	0.331	0.333	0.335	0.337	0.339	0.342	0.345	0.348	0.352	0.356	0.360	0.363	0.367	0.372	0.377	0.382	0.386	0.391	0.394
Samoa	0.531	0.535	0.539	0.543	0.546	0.550	0.555	0.559	0.563	0.567	0.571	0.576	0.581	0.586	0.590	0.595	0.599	0.603	0.607	0.610	0.612	0.615	0.618	0.620	0.623	0.626	0.629	0.633	0.637	0.641
Solomon Islands	0.279	0.283	0.288	0.294	0.300	0.306	0.312	0.317	0.321	0.326	0.328	0.330	0.331	0.332	0.333	0.336	0.339	0.343	0.348	0.353	0.358	0.364	0.371	0.377	0.382	0.387	0.393	0.398	0.403	0.407
Tokelau	0.427	0.432	0.438	0.444	0.450	0.456	0.463	0.470	0.477	0.484	0.491	0.498	0.504	0.511	0.519	0.526	0.534	0.542	0.550	0.557	0.565	0.573	0.580	0.588	0.595	0.602	0.608	0.615	0.621	0.626
Tonga	0.510	0.517	0.522	0.527	0.533	0.538	0.543	0.547	0.551	0.555	0.559	0.563	0.568	0.572	0.576	0.580	0.583	0.587	0.590	0.594	0.598	0.602	0.606	0.610	0.614	0.618	0.622	0.627	0.632	0.636
Tuvalu	0.426	0.434	0.442	0.450	0.458	0.465	0.470	0.476	0.483	0.490	0.496	0.502	0.509	0.514	0.519	0.523	0.527	0.531	0.537	0.541	0.545	0.549	0.553	0.558	0.562	0.567	0.573	0.579	0.584	0.589
Vanuatu	0.361	0.365	0.369	0.373	0.377	0.381	0.386	0.390	0.395	0.399	0.403	0.407	0.410	0.413	0.417	0.420	0.425	0.430	0.435	0.440	0.446	0.451	0.455	0.460	0.464	0.468	0.473	0.477	0.481	0.485

GBD_2019_appendix

Socio-Demographic Index values for all estimated GBD 2019 locations, 1990-2019

Location	1990	1991	1992	1993	1994	1995	1996	1997	1998	1999	2000	2001	2002	2003	2004	2005	2006	2007	2008	2009	2010	2011	2012	2013	2014	2015	2016	2017	2018	2019
Southeast Asia	0.455	0.463	0.471	0.479	0.488	0.496	0.504	0.512	0.517	0.523	0.528	0.533	0.538	0.543	0.548	0.554	0.559	0.566	0.572	0.578	0.585	0.592	0.599	0.606	0.612	0.619	0.626	0.632	0.639	0.644
Cambodia	0.266	0.272	0.277	0.282	0.286	0.291	0.296	0.301	0.307	0.313	0.321	0.328	0.337	0.345	0.354	0.363	0.373	0.382	0.391	0.399	0.406	0.413	0.421	0.428	0.435	0.442	0.449	0.456	0.463	0.469
Indonesia	0.452	0.462	0.472	0.481	0.490	0.499	0.508	0.516	0.522	0.527	0.533	0.537	0.542	0.547	0.552	0.558	0.564	0.571	0.578	0.585	0.593	0.601	0.609	0.617	0.625	0.633	0.640	0.647	0.654	0.660
Laos	0.268	0.274	0.279	0.285	0.290	0.296	0.302	0.309	0.315	0.322	0.329	0.336	0.344	0.351	0.359	0.367	0.376	0.385	0.394	0.403	0.413	0.422	0.431	0.441	0.450	0.458	0.467	0.475	0.483	0.490
Malaysia	0.542	0.548	0.554	0.562	0.572	0.581	0.590	0.600	0.611	0.622	0.630	0.638	0.646	0.652	0.659	0.665	0.671	0.677	0.681	0.687	0.693	0.698	0.704	0.710	0.716	0.722	0.726	0.728	0.732	0.737
Maldives	0.303	0.314	0.324	0.336	0.347	0.359	0.370	0.382	0.394	0.406	0.417	0.427	0.437	0.447	0.456	0.464	0.473	0.481	0.490	0.497	0.504	0.511	0.518	0.525	0.532	0.538	0.544	0.551	0.557	0.562
Mauritius	0.527	0.532	0.535	0.543	0.556	0.565	0.570	0.576	0.583	0.588	0.593	0.598	0.603	0.608	0.614	0.621	0.627	0.633	0.640	0.646	0.652	0.658	0.665	0.673	0.680	0.686	0.690	0.695	0.700	0.705
Myanmar	0.284	0.287	0.290	0.295	0.300	0.306	0.313	0.320	0.327	0.335	0.344	0.353	0.363	0.373	0.384	0.395	0.406	0.417	0.427	0.437	0.446	0.455	0.464	0.473	0.482	0.490	0.498	0.506	0.514	0.521
Philippines	0.497	0.501	0.505	0.509	0.513	0.518	0.522	0.526	0.529	0.532	0.534	0.537	0.540	0.542	0.545	0.547	0.550	0.554	0.558	0.562	0.567	0.572	0.577	0.583	0.589	0.596	0.603	0.610	0.617	0.623
Seychelles	0.567	0.576	0.584	0.592	0.600	0.607	0.614	0.621	0.629	0.636	0.642	0.647	0.652	0.656	0.659	0.662	0.666	0.670	0.673	0.676	0.679	0.683	0.687	0.691	0.696	0.702	0.707	0.713	0.719	0.724
Sri Lanka	0.504	0.511	0.518	0.525	0.532	0.539	0.547	0.554	0.561	0.567	0.573	0.578	0.582	0.587	0.592	0.597	0.602	0.609	0.615	0.621	0.628	0.636	0.644	0.651	0.658	0.666	0.672	0.678	0.684	0.690
Thailand	0.508	0.518	0.527	0.536	0.544	0.553	0.562	0.569	0.574	0.578	0.583	0.589	0.594	0.599	0.605	0.610	0.616	0.623	0.629	0.633	0.638	0.643	0.649	0.655	0.660	0.666	0.671	0.676	0.682	0.687
Timor-Leste	0.274	0.280	0.286	0.293	0.300	0.308	0.317	0.326	0.334	0.339	0.345	0.352	0.358	0.364	0.374	0.388	0.406	0.421	0.436	0.448	0.458	0.469	0.480	0.488	0.493	0.498	0.503	0.508	0.511	0.514
Vietnam	0.390	0.397	0.404	0.412	0.420	0.429	0.438	0.447	0.455	0.463	0.471	0.478	0.486	0.493	0.501	0.509	0.517	0.525	0.533	0.541	0.549	0.558	0.566	0.573	0.581	0.589	0.596	0.604	0.611	0.617
Sub-Saharan Africa	0.291	0.295	0.299	0.302	0.306	0.309	0.314	0.318	0.322	0.327	0.331	0.336	0.341	0.346	0.352	0.359	0.365	0.372	0.380	0.386	0.394	0.401	0.409	0.416	0.423	0.431	0.438	0.445	0.452	0.456
Central sub-Saharan Africa	0.269	0.273	0.276	0.279	0.281	0.283	0.286	0.289	0.292	0.295	0.298	0.302	0.308	0.313	0.320	0.328	0.336	0.346	0.357	0.367	0.378	0.389	0.400	0.412	0.423	0.434	0.445	0.454	0.463	0.470
Angola	0.238	0.243	0.247	0.250	0.253	0.257	0.262	0.267	0.273	0.278	0.283	0.289	0.296	0.303	0.311	0.319	0.330	0.341	0.353	0.364	0.376	0.387	0.398	0.410	0.421	0.432	0.443	0.454	0.463	0.470
Central African Republic	0.186	0.190	0.193	0.196	0.199	0.202	0.205	0.208	0.211	0.215	0.218	0.221	0.225	0.227	0.230	0.233	0.236	0.240	0.244	0.248	0.253	0.258	0.263	0.263	0.263	0.264	0.266	0.268	0.271	0.274
Congo (Brazzaville)	0.364	0.373	0.381	0.389	0.396	0.403	0.410	0.416	0.421	0.426	0.431	0.437	0.442	0.447	0.452	0.458	0.464	0.469	0.475	0.482	0.491	0.500	0.509	0.519	0.528	0.538	0.547	0.556	0.563	0.568
DR Congo	0.260	0.262	0.263	0.263	0.262	0.261	0.259	0.256	0.253	0.250	0.246	0.242	0.239	0.238	0.239	0.242	0.244	0.247	0.251	0.257	0.266	0.277	0.289	0.305	0.321	0.336	0.350	0.362	0.374	0.382
Equatorial Guinea	0.208	0.214	0.222	0.230	0.240	0.252	0.268	0.295	0.318	0.341	0.364	0.391	0.415	0.438	0.460	0.482	0.502	0.522	0.543	0.561	0.578	0.594	0.611	0.626	0.640	0.652	0.663	0.673	0.681	0.685
Gabon	0.388	0.399	0.409	0.420	0.431	0.442	0.453	0.463	0.474	0.483	0.493	0.502	0.510	0.519	0.528	0.537	0.546	0.554	0.563	0.570	0.579	0.587	0.596	0.605	0.614	0.623	0.632	0.641	0.649	0.656
Eastern sub-Saharan Africa	0.235	0.239	0.242	0.245	0.249	0.252	0.257	0.261	0.265	0.270	0.275	0.280	0.285	0.290	0.295	0.301	0.307	0.314	0.321	0.328	0.336	0.343	0.351	0.359	0.367	0.375	0.383	0.391	0.399	0.405
Burundi	0.198	0.201	0.204	0.207	0.209	0.210	0.210	0.211	0.212	0.213	0.213	0.214	0.216	0.218	0.220	0.223	0.226	0.230	0.234	0.238	0.243	0.248	0.254	0.260	0.266	0.270	0.274	0.278	0.282	0.284
Comoros	0.274	0.281	0.288	0.296	0.302	0.309	0.315	0.322	0.328	0.334	0.340	0.346	0.353	0.359	0.365	0.371	0.378	0.384	0.390	0.396	0.401	0.407	0.413	0.419	0.426	0.432	0.438	0.444	0.450	0.455
Djibouti	0.275	0.277	0.280	0.285	0.289	0.293	0.297	0.302	0.306	0.311	0.317	0.322	0.327	0.333	0.339	0.345	0.353	0.360	0.368	0.376	0.384	0.392	0.400	0.408	0.416	0.425	0.434	0.443	0.452	0.459
Eritrea	0.198	0.203	0.210	0.218	0.228	0.238	0.248	0.258	0.267	0.275	0.283	0.289	0.296	0.302	0.307	0.312	0.317	0.322	0.326	0.330	0.334	0.340	0.346	0.353	0.360	0.367	0.375	0.382	0.390	0.396
Ethiopia	0.144	0.146	0.147	0.149	0.151	0.153	0.157	0.161	0.164	0.167	0.171	0.176	0.180	0.183	0.189	0.195	0.203	0.213	0.223	0.233	0.244	0.256	0.268	0.279	0.291	0.302	0.313	0.324	0.334	0.343
Kenya	0.333	0.341	0.348	0.354	0.360	0.366	0.372	0.378	0.383	0.388	0.392	0.397	0.401	0.404	0.409	0.413	0.418	0.424	0.429	0.435	0.441	0.448	0.455	0.463	0.470	0.478	0.486	0.494	0.502	0.508
Madagascar	0.265	0.268	0.270	0.273	0.275	0.277	0.280	0.283	0.286	0.290	0.294	0.299	0.302	0.307	0.311	0.316	0.320	0.324	0.328	0.332	0.336	0.342	0.348	0.355	0.361	0.369	0.376	0.383	0.391	0.396
Malawi	0.213	0.215	0.215	0.217	0.218	0.220	0.225	0.230	0.235	0.240	0.245	0.249	0.254	0.260	0.267	0.274	0.281	0.290	0.299	0.308	0.317	0.326	0.335	0.342	0.350	0.358	0.365	0.372	0.379	0.384
Mozambique	0.120	0.122	0.123	0.126	0.129	0.131	0.137	0.144	0.152	0.159	0.165	0.172	0.179	0.186	0.194	0.201	0.208	0.215	0.223	0.230	0.237	0.244	0.252	0.260	0.268	0.277	0.285	0.294	0.301	0.307
Rwanda	0.257	0.259	0.262	0.265	0.261	0.261	0.262	0.265	0.269	0.272	0.277	0.283	0.291	0.298	0.307	0.315	0.325	0.333	0.342	0.351	0.359	0.368	0.376	0.384	0.391	0.399	0.407	0.415	0.422	0.429
Somalia	0.051	0.051	0.052	0.053	0.054	0.055	0.056	0.057	0.057	0.058	0.059	0.060	0.061	0.062	0.063	0.064	0.065	0.066	0.067	0.068	0.069	0.070	0.072	0.073	0.074	0.076	0.077	0.079	0.080	0.081

Socio-Demographic Index values for all estimated GBD 2019 locations, 1990-2019

Location	1990	1991	1992	1993	1994	1995	1996	1997	1998	1999	2000	2001	2002	2003	2004	2005	2006	2007	2008	2009	2010	2011	2012	2013	2014	2015	2016	2017	2018	2019
South Sudan	0.248	0.251	0.253	0.256	0.259	0.261	0.264	0.267	0.271	0.274	0.277	0.281	0.284	0.288	0.292	0.296	0.301	0.305	0.310	0.315	0.320	0.325	0.330	0.334	0.339	0.342	0.345	0.351	0.358	0.363
Uganda	0.167	0.169	0.172	0.176	0.181	0.187	0.194	0.201	0.208	0.216	0.225	0.234	0.243	0.253	0.262	0.272	0.282	0.293	0.303	0.314	0.325	0.335	0.345	0.355	0.364	0.373	0.382	0.391	0.399	0.404
Tanzania	0.260	0.265	0.269	0.273	0.276	0.280	0.284	0.288	0.292	0.296	0.301	0.305	0.310	0.315	0.320	0.326	0.331	0.337	0.342	0.348	0.354	0.361	0.368	0.375	0.383	0.391	0.399	0.408	0.416	0.423
Zambia	0.299	0.304	0.307	0.311	0.314	0.316	0.319	0.323	0.326	0.330	0.334	0.340	0.345	0.352	0.359	0.367	0.376	0.386	0.396	0.407	0.418	0.429	0.440	0.451	0.462	0.472	0.481	0.491	0.499	0.505
Southern sub-Saharan Africa	0.513	0.517	0.522	0.526	0.530	0.535	0.539	0.544	0.548	0.553	0.557	0.562	0.567	0.571	0.576	0.581	0.586	0.591	0.596	0.601	0.605	0.610	0.614	0.619	0.623	0.628	0.632	0.636	0.639	0.642
Botswana	0.431	0.441	0.451	0.459	0.467	0.475	0.483	0.491	0.498	0.506	0.514	0.521	0.528	0.535	0.541	0.548	0.555	0.562	0.569	0.575	0.581	0.587	0.593	0.600	0.606	0.612	0.618	0.624	0.630	0.634
eSwatini	0.392	0.401	0.410	0.419	0.427	0.435	0.443	0.450	0.456	0.462	0.468	0.474	0.479	0.484	0.490	0.495	0.501	0.507	0.513	0.519	0.526	0.532	0.538	0.545	0.551	0.557	0.563	0.569	0.574	0.577
Lesotho	0.321	0.327	0.333	0.340	0.346	0.353	0.360	0.367	0.373	0.380	0.387	0.393	0.399	0.405	0.411	0.417	0.423	0.429	0.435	0.441	0.448	0.455	0.462	0.469	0.476	0.483	0.489	0.496	0.502	0.507
Namibia	0.454	0.459	0.465	0.470	0.475	0.480	0.486	0.491	0.495	0.500	0.505	0.509	0.514	0.518	0.523	0.529	0.534	0.540	0.546	0.552	0.558	0.564	0.571	0.577	0.584	0.591	0.597	0.603	0.608	0.612
South Africa	0.552	0.556	0.560	0.564	0.567	0.572	0.576	0.580	0.584	0.588	0.593	0.597	0.602	0.607	0.611	0.616	0.622	0.627	0.632	0.637	0.642	0.647	0.651	0.656	0.660	0.664	0.668	0.672	0.676	0.678
Zimbabwe	0.394	0.403	0.409	0.415	0.421	0.426	0.432	0.437	0.441	0.444	0.446	0.448	0.448	0.447	0.444	0.441	0.437	0.433	0.428	0.425	0.425	0.427	0.432	0.438	0.445	0.452	0.459	0.465	0.471	0.476
Western sub-Saharan Africa	0.268	0.272	0.276	0.280	0.284	0.288	0.292	0.297	0.301	0.306	0.311	0.316	0.322	0.328	0.335	0.343	0.351	0.359	0.367	0.375	0.383	0.391	0.399	0.407	0.415	0.422	0.430	0.437	0.443	0.448
Benin	0.209	0.213	0.218	0.222	0.227	0.231	0.236	0.241	0.245	0.250	0.254	0.259	0.263	0.267	0.271	0.275	0.279	0.283	0.288	0.292	0.297	0.301	0.306	0.312	0.318	0.324	0.331	0.338	0.346	0.352
Burkina Faso	0.125	0.128	0.131	0.134	0.137	0.140	0.144	0.147	0.152	0.156	0.161	0.166	0.170	0.175	0.180	0.186	0.191	0.196	0.200	0.205	0.210	0.215	0.220	0.226	0.231	0.236	0.241	0.247	0.252	0.257
Cape Verde	0.292	0.298	0.305	0.311	0.318	0.326	0.333	0.342	0.350	0.360	0.370	0.379	0.388	0.397	0.407	0.415	0.424	0.434	0.443	0.452	0.461	0.469	0.477	0.484	0.491	0.498	0.505	0.512	0.519	0.525
Cameroon	0.313	0.320	0.325	0.330	0.334	0.338	0.342	0.346	0.349	0.353	0.357	0.360	0.364	0.368	0.373	0.378	0.384	0.390	0.397	0.404	0.412	0.420	0.428	0.436	0.445	0.455	0.464	0.474	0.483	0.490
Chad	0.108	0.112	0.115	0.118	0.120	0.123	0.125	0.128	0.130	0.132	0.135	0.137	0.140	0.144	0.149	0.156	0.162	0.167	0.173	0.179	0.185	0.191	0.197	0.203	0.210	0.216	0.223	0.228	0.234	0.238
Côte d'Ivoire	0.256	0.260	0.265	0.270	0.275	0.279	0.285	0.290	0.295	0.301	0.305	0.310	0.314	0.318	0.322	0.325	0.329	0.333	0.337	0.341	0.346	0.350	0.355	0.362	0.369	0.376	0.384	0.393	0.401	0.408
The Gambia	0.218	0.223	0.227	0.232	0.237	0.241	0.246	0.251	0.256	0.262	0.268	0.274	0.280	0.286	0.293	0.300	0.306	0.313	0.319	0.327	0.334	0.341	0.348	0.356	0.363	0.370	0.378	0.385	0.393	0.399
Ghana	0.355	0.362	0.368	0.374	0.380	0.385	0.391	0.396	0.401	0.406	0.411	0.415	0.420	0.426	0.431	0.437	0.444	0.450	0.458	0.466	0.474	0.484	0.494	0.504	0.514	0.523	0.531	0.541	0.549	0.557
Guinea	0.175	0.178	0.182	0.186	0.190	0.194	0.199	0.204	0.209	0.214	0.219	0.224	0.229	0.235	0.239	0.244	0.249	0.253	0.258	0.263	0.267	0.272	0.278	0.284	0.290	0.296	0.303	0.310	0.318	0.325
Guinea-Bissau	0.200	0.205	0.209	0.214	0.219	0.224	0.230	0.236	0.239	0.244	0.249	0.253	0.257	0.261	0.266	0.271	0.275	0.280	0.286	0.291	0.297	0.304	0.310	0.316	0.322	0.328	0.335	0.342	0.349	0.355
Liberia	0.221	0.222	0.219	0.214	0.206	0.196	0.183	0.176	0.175	0.184	0.203	0.220	0.238	0.245	0.252	0.258	0.265	0.272	0.279	0.287	0.296	0.305	0.314	0.325	0.335	0.344	0.351	0.358	0.365	0.370
Mali	0.126	0.129	0.132	0.136	0.139	0.143	0.147	0.151	0.155	0.159	0.163	0.168	0.173	0.178	0.183	0.188	0.193	0.198	0.203	0.209	0.214	0.220	0.225	0.230	0.235	0.241	0.247	0.253	0.259	0.263
Mauritania	0.308	0.314	0.319	0.326	0.332	0.338	0.344	0.349	0.355	0.360	0.365	0.369	0.374	0.379	0.384	0.390	0.398	0.406	0.413	0.420	0.427	0.435	0.443	0.450	0.459	0.467	0.474	0.482	0.490	0.496
Niger	0.073	0.075	0.076	0.078	0.079	0.081	0.082	0.084	0.085	0.087	0.089	0.091	0.093	0.096	0.098	0.101	0.104	0.108	0.111	0.115	0.119	0.123	0.128	0.133	0.138	0.143	0.148	0.153	0.158	0.162
Nigeria	0.305	0.308	0.312	0.315	0.319	0.324	0.329	0.334	0.339	0.344	0.350	0.356	0.363	0.371	0.381	0.392	0.402	0.412	0.422	0.432	0.442	0.451	0.460	0.469	0.478	0.487	0.495	0.503	0.510	0.515
São Tomé and PrÍncipe	0.299	0.302	0.306	0.309	0.313	0.317	0.322	0.327	0.332	0.338	0.344	0.351	0.358	0.365	0.373	0.381	0.390	0.398	0.407	0.416	0.424	0.433	0.443	0.452	0.461	0.470	0.478	0.487	0.495	0.502
Senegal	0.227	0.233	0.239	0.245	0.251	0.257	0.262	0.267	0.272	0.277	0.282	0.286	0.290	0.295	0.299	0.304	0.308	0.313	0.318	0.324	0.330	0.336	0.342	0.348	0.354	0.361	0.368	0.375	0.382	0.389
Sierra Leone	0.207	0.209	0.210	0.212	0.215	0.218	0.218	0.218	0.219	0.218	0.219	0.221	0.224	0.229	0.234	0.239	0.245	0.252	0.260	0.267	0.275	0.283	0.292	0.304	0.314	0.321	0.328	0.335	0.342	0.347
Togo	0.266	0.272	0.278	0.281	0.286	0.291	0.296	0.302	0.306	0.310	0.313	0.317	0.320	0.323	0.327	0.330	0.334	0.338	0.342	0.347	0.352	0.358	0.364	0.371	0.379	0.386	0.394	0.402	0.411	0.417

Measure	Location	Sex	Change Rate	95% CI (lower)	95% CI (upper)
Death	Global	Male	-0.2079	-0.3048	-0.0944
Death	Global	Female	-0.2621	-0.3598	-0.1515
Death	Global	Both	-0.2270	-0.3106	-0.1353
Death	High SDI	Both	-0.1974	-0.2558	-0.1331
Death	High-middle SDI	Both	-0.2644	-0.3505	-0.1677
Death	Low SDI	Both	-0.0699	-0.2298	0.1190
Death	Low-middle SDI	Both	-0.0514	-0.1851	0.1094
Death	Middle SDI	Both	-0.2731	-0.3979	-0.1194
Incidence	Global	Male	-0.0416	-0.1633	0.0953
Incidence	Global	Female	-0.1348	-0.2417	-0.0129
Incidence	Global	Both	-0.0757	-0.1742	0.0341
Incidence	High SDI	Both	-0.0637	-0.1532	0.0353
Incidence	High-middle SDI	Both	-0.0381	-0.1530	0.0961
Incidence	Low SDI	Both	-0.1027	-0.2531	0.0749
Incidence	Low-middle SDI	Both	0.0224	-0.1263	0.1960
Incidence	Middle SDI	Both	-0.1789	-0.3218	-0.0091

measure	cause	location	sex	year	Change in Rate	95% CI (lower)	95% CI (upper)	P Value
Death	colon and rectum cancer	Global	Both	1990-2019	-0.0437	-0.1206	0.0405	>0.05
Death	colon and rectum cancer	Global	Female	1990-2019	-0.1297	-0.2247	-0.0225	<0.05
Death	colon and rectum cancer	Global	Male	1990-2019	0.0297	-0.0571	0.1221	>0.05
Death	colon and rectum cancer	High SDI	Both	1990-2019	-0.2307	-0.2883	-0.1673	<0.05
Death	colon and rectum cancer	High-middle SDI	Both	1990-2019	0.0100	-0.0754	0.1054	>0.05
Death	colon and rectum cancer	Middle SDI	Both	1990-2010	0.3454	0.2160	0.4874	<0.05
Death	colon and rectum cancer	Middle SDI	Both	1990-2019	0.3663	0.2081	0.5497	<0.05
Death	colon and rectum cancer	Middle SDI	Both	2010-2019	0.0155	-0.0951	0.1398	>0.05
Death	colon and rectum cancer	Low-middle SDI	Both	1990-2019	0.3964	0.2100	0.6097	<0.05
Death	colon and rectum cancer	Low SDI	Both	1990-1995	-0.0088	-0.2084	0.2349	>0.05
Death	colon and rectum cancer	Low SDI	Both	1990-2019	0.1784	-0.0316	0.4412	>0.05
Death	colon and rectum cancer	Low SDI	Both	1995-2019	0.1888	-0.0136	0.4325	>0.05
Death	esophageal cancer	Global	Both	1990-2005	0.0381	-0.2066	0.3559	>0.05
Death	esophageal cancer	Global	Both	1990-2019	-0.2532	-0.3904	-0.0866	<0.05
Death	esophageal cancer	Global	Both	2005-2015	-0.2698	-0.4196	-0.0853	<0.05
Death	esophageal cancer	Global	Both	2015-2019	-0.0148	-0.1548	0.1479	>0.05
Death	esophageal cancer	Global	Female	1990-2005	-0.0538	-0.3252	0.3264	>0.05
Death	esophageal cancer	Global	Female	1990-2019	-0.3961	-0.5383	-0.2096	<0.05
Death	esophageal cancer	Global	Female	2005-2015	-0.3486	-0.5127	-0.1221	<0.05
Death	esophageal cancer	Global	Female	2015-2019	-0.0202	-0.1968	0.1940	>0.05
Death	esophageal cancer	Global	Male	1990-2005	0.0767	-0.1784	0.4070	>0.05
Death	esophageal cancer	Global	Male	1990-2019	-0.1884	-0.3488	0.0089	>0.05
Death	esophageal cancer	Global	Male	2005-2019	-0.2463	-0.4095	-0.0336	<0.05
Death	esophageal cancer	High SDI	Both	1990-2005	0.0051	-0.0387	0.0501	>0.05
Death	esophageal cancer	High SDI	Both	1990-2019	-0.0885	-0.1401	-0.0319	<0.05
Death	esophageal cancer	High SDI	Both	2005-2019	-0.0931	-0.1507	-0.0327	<0.05
Death	esophageal cancer	High-middle SDI	Both	1990-2005	0.0638	-0.1766	0.3732	>0.05
Death	esophageal cancer	High-middle SDI	Both	1990-2019	-0.2064	-0.3686	-0.0021	<0.05
Death	esophageal cancer	High-middle SDI	Both	2005-2019	-0.2540	-0.4360	-0.0099	<0.05
Death	esophageal cancer	Middle SDI	Both	1990-2005	0.0091	-0.3838	0.6549	>0.05
Death	esophageal cancer	Middle SDI	Both	1990-2019	-0.4234	-0.6031	-0.1559	<0.05
Death	esophageal cancer	Middle SDI	Both	2005-2015	-0.4010	-0.6050	-0.0974	<0.05
Death	esophageal cancer	Middle SDI	Both	2015-2019	-0.0462	-0.2526	0.2241	>0.05
Death	esophageal cancer	Low-middle SDI	Both	1990-2019	-0.1520	-0.3678	0.1358	>0.05
Death	esophageal cancer	Low SDI	Both	1990-2019	-0.1086	-0.3005	0.1333	>0.05
Death	liver cancer	Global	Both	1990-1995	0.1080	-0.0192	0.2488	>0.05
Death	liver cancer	Global	Both	1990-2019	-0.3340	-0.4169	-0.2400	<0.05
Death	liver cancer	Global	Both	1995-2019	-0.3989	-0.4599	-0.3317	<0.05
Death	liver cancer	Global	Female	1990-1995	0.0499	-0.1067	0.2400	>0.05
Death	liver cancer	Global	Female	1990-2019	-0.3495	-0.4516	-0.2279	<0.05
Death	liver cancer	Global	Female	1995-2019	-0.3804	-0.4652	-0.2832	<0.05
Death	liver cancer	Global	Male	1990-1995	0.1341	-0.0308	0.3231	>0.05
Death	liver cancer	Global	Male	1990-2019	-0.3230	-0.4240	-0.2007	<0.05
Death	liver cancer	Global	Male	1995-2019	-0.4031	-0.4770	-0.3207	<0.05
Death	liver cancer	High SDI	Both	1990-2000	0.4545	0.3909	0.5191	<0.05
Death	liver cancer	High SDI	Both	1990-2019	0.2546	0.1684	0.3490	<0.05
Death	liver cancer	High SDI	Both	2000-2019	-0.1374	-0.2004	-0.0721	<0.05
Death	liver cancer	High-middle SDI	Both	1990-2019	-0.5146	-0.5895	-0.4274	<0.05
Death	liver cancer	Middle SDI	Both	1990-2019	-0.4721	-0.5615	-0.3672	<0.05
Death	liver cancer	Low-middle SDI	Both	1990-2019	-0.2401	-0.3363	-0.1282	<0.05
Death	liver cancer	Low SDI	Both	1990-1995	0.0300	-0.1323	0.2260	>0.05
Death	liver cancer	Low SDI	Both	1990-2019	-0.0992	-0.2450	0.0715	>0.05
Death	liver cancer	Low SDI	Both	1995-2019	-0.1255	-0.2506	0.0215	>0.05
Death	pancreatic cancer	Global	Both	1990-2019	0.2397	0.1393	0.3487	<0.05
Death	pancreatic cancer	Global	Female	1990-2019	0.2430	0.1144	0.3881	<0.05
Death	pancreatic cancer	Global	Male	1990-2019	0.2341	0.1304	0.3490	<0.05
Death	pancreatic cancer	High SDI	Both	1990-2019	0.1312	0.0463	0.2225	<0.05
Death	pancreatic cancer	High-middle SDI	Both	1990-2019	0.2314	0.1233	0.3482	<0.05
Death	pancreatic cancer	Middle SDI	Both	1990-2019	0.7496	0.5352	1.0004	<0.05
Death	pancreatic cancer	Low-middle SDI	Both	1990-2019	0.9624	0.6512	1.3283	<0.05
Death	pancreatic cancer	Low SDI	Both	1990-1995	0.0441	-0.2138	0.3816	>0.05
Death	pancreatic cancer	Low SDI	Both	1990-2019	0.5932	0.2580	1.0175	<0.05
Death	pancreatic cancer	Low SDI	Both	1995-2019	0.5260	0.2068	0.9354	<0.05
Death	stomach cancer	Global	Both	1990-2019	-0.4198	-0.4762	-0.3552	<0.05
Death	stomach cancer	Global	Female	1990-2019	-0.4607	-0.5271	-0.3861	<0.05
Death	stomach cancer	Global	Male	1990-2019	-0.3994	-0.4737	-0.3179	<0.05
Death	stomach cancer	High SDI	Both	1990-2019	-0.5157	-0.5550	-0.4734	<0.05
Death	stomach cancer	High-middle SDI	Both	1990-2019	-0.4751	-0.5339	-0.4101	<0.05
Death	stomach cancer	Middle SDI	Both	1990-2005	-0.0708	-0.1730	0.0451	>0.05
Death	stomach cancer	Middle SDI	Both	1990-2019	-0.4112	-0.4910	-0.3178	<0.05
Death	stomach cancer	Middle SDI	Both	2005-2019	-0.3664	-0.4461	-0.2750	<0.05
Death	stomach cancer	Low-middle SDI	Both	1990-2019	-0.2716	-0.3549	-0.1782	<0.05
Death	stomach cancer	Low SDI	Both	1990-2019	-0.2602	-0.3586	-0.1476	<0.05

measure	cause	location	sex	year	Change in Rate	95% CI (lower)	95% CI (upper)	P Value
Incidence	colon and rectum cancer	Global	Both	1990-2019	0.2006	0.0985	0.3163	<0.05
Incidence	colon and rectum cancer	Global	Female	1990-2019	0.0651	-0.0442	0.1899	>0.05
Incidence	colon and rectum cancer	Global	Male	1990-2019	0.3123	0.1945	0.4485	<0.05
Incidence	colon and rectum cancer	High SDI	Both	1990-2019	0.0076	-0.0860	0.1090	>0.05
Incidence	colon and rectum cancer	High-middle SDI	Both	1990-2019	0.4340	0.2975	0.5868	<0.05
Incidence	colon and rectum cancer	Middle SDI	Both	1990-2019	0.8453	0.6274	1.0978	<0.05
Incidence	colon and rectum cancer	Low-middle SDI	Both	1990-2019	0.6387	0.4204	0.8918	<0.05
Incidence	colon and rectum cancer	Low SDI	Both	1990-2019	0.1830	-0.0315	0.4488	>0.05
Incidence	esophageal cancer	Global	Both	1990-2005	0.0790	-0.1650	0.4056	>0.05
Incidence	esophageal cancer	Global	Both	1990-2019	-0.1928	-0.3390	-0.0154	<0.05
Incidence	esophageal cancer	Global	Both	2005-2019	-0.2519	-0.4085	-0.0503	<0.05
Incidence	esophageal cancer	Global	Female	1990-2005	0.0051	-0.2875	0.4361	>0.05
Incidence	esophageal cancer	Global	Female	1990-2019	-0.3223	-0.4834	-0.1103	<0.05
Incidence	esophageal cancer	Global	Female	2005-2015	-0.3224	-0.4985	-0.0885	<0.05
Incidence	esophageal cancer	Global	Female	2015-2019	-0.0049	-0.1860	0.2228	>0.05
Incidence	esophageal cancer	Global	Male	1990-2005	0.1092	-0.1454	0.4394	>0.05
Incidence	esophageal cancer	Global	Male	1990-2019	-0.1344	-0.2992	0.0808	>0.05
Incidence	esophageal cancer	Global	Male	2005-2019	-0.2197	-0.3916	-0.0091	<0.05
Incidence	esophageal cancer	High SDI	Both	1990-2005	0.1172	0.0726	0.1647	<0.05
Incidence	esophageal cancer	High SDI	Both	1990-2019	0.0245	-0.0697	0.1289	>0.05
Incidence	esophageal cancer	High SDI	Both	2005-2019	-0.0829	-0.1715	0.0139	>0.05
Incidence	esophageal cancer	High-middle SDI	Both	1990-2019	-0.1271	-0.3130	0.1082	>0.05
Incidence	esophageal cancer	Middle SDI	Both	1990-2005	-0.0938	-0.4505	0.4922	>0.05
Incidence	esophageal cancer	Middle SDI	Both	1990-2019	-0.4798	-0.6388	-0.2492	<0.05
Incidence	esophageal cancer	Middle SDI	Both	2005-2015	-0.4126	-0.6081	-0.1293	<0.05
Incidence	esophageal cancer	Middle SDI	Both	2015-2019	-0.0227	-0.2053	0.1995	>0.05
Incidence	esophageal cancer	Low-middle SDI	Both	1990-2019	-0.1443	-0.3672	0.1647	>0.05
Incidence	esophageal cancer	Low SDI	Both	1990-2019	-0.1760	-0.3457	0.0399	>0.05
Incidence	liver cancer	Global	Both	1990-1995	0.1203	-0.0068	0.2668	>0.05
Incidence	liver cancer	Global	Both	1990-2019	-0.2749	-0.3691	-0.1668	<0.05
Incidence	liver cancer	Global	Both	1995-2019	-0.3527	-0.4209	-0.2774	<0.05
Incidence	liver cancer	Global	Female	1990-2005	-0.2520	-0.3513	-0.1386	<0.05
Incidence	liver cancer	Global	Female	1990-2019	-0.3049	-0.4144	-0.1779	<0.05
Incidence	liver cancer	Global	Female	2005-2019	-0.0708	-0.1825	0.0579	>0.05
Incidence	liver cancer	Global	Male	1990-2019	-0.2568	-0.3747	-0.1185	<0.05
Incidence	liver cancer	High SDI	Both	1990-2000	0.5369	0.4743	0.6015	<0.05
Incidence	liver cancer	High SDI	Both	1990-2019	0.4448	0.3076	0.6001	<0.05
Incidence	liver cancer	High SDI	Both	2000-2019	-0.0600	-0.1527	0.0421	>0.05
Incidence	liver cancer	High-middle SDI	Both	1990-2019	-0.4618	-0.5495	-0.3569	<0.05
Incidence	liver cancer	Middle SDI	Both	1990-2019	-0.5031	-0.5889	-0.3969	<0.05
Incidence	liver cancer	Low-middle SDI	Both	1990-2019	-0.2562	-0.3548	-0.1436	<0.05
Incidence	liver cancer	Low SDI	Both	1990-1995	-0.0710	-0.2170	0.0989	>0.05
Incidence	liver cancer	Low SDI	Both	1990-2019	-0.1403	-0.2742	0.0188	>0.05
Incidence	liver cancer	Low SDI	Both	1995-2019	-0.0746	-0.2085	0.0822	>0.05
Incidence	pancreatic cancer	Global	Both	1990-2019	0.2573	0.1458	0.3801	<0.05
Incidence	pancreatic cancer	Global	Female	1990-2019	0.2635	0.1244	0.4148	<0.05
Incidence	pancreatic cancer	Global	Male	1990-2019	0.2502	0.1364	0.3787	<0.05
Incidence	pancreatic cancer	High SDI	Both	1990-2019	0.1716	0.0555	0.2993	<0.05
Incidence	pancreatic cancer	High-middle SDI	Both	1990-2019	0.2475	0.1308	0.3715	<0.05
Incidence	pancreatic cancer	Middle SDI	Both	1990-2019	0.6514	0.4480	0.8829	<0.05
Incidence	pancreatic cancer	Low-middle SDI	Both	1990-2019	0.9489	0.6429	1.2987	<0.05
Incidence	pancreatic cancer	Low SDI	Both	1990-2019	0.5073	0.1915	0.8994	<0.05
Incidence	stomach cancer	Global	Both	1990-2019	-0.3054	-0.3787	-0.2245	<0.05
Incidence	stomach cancer	Global	Female	1990-2019	-0.3860	-0.4597	-0.3046	<0.05
Incidence	stomach cancer	Global	Male	1990-2019	-0.2639	-0.3615	-0.1551	<0.05
Incidence	stomach cancer	High SDI	Both	1990-2019	-0.4363	-0.4929	-0.3727	<0.05
Incidence	stomach cancer	High-middle SDI	Both	1990-2019	-0.3129	-0.3976	-0.2169	<0.05
Incidence	stomach cancer	Middle SDI	Both	1990-2019	-0.3381	-0.4360	-0.2239	<0.05
Incidence	stomach cancer	Low-middle SDI	Both	1990-2019	-0.2291	-0.3168	-0.1329	<0.05
Incidence	stomach cancer	Low SDI	Both	1990-2019	-0.2925	-0.3900	-0.1792	<0.05

Cause	Year	Age-standardised incidence rate (per 100 000 person-years)	95% CI (lower)	95% CI (upper)
Colon and rectum cance	1990	22.25	21.29	22.97
Esophageal cancer	1990	8.06	6.41	8.83
Liver cancer	1990	8.98	8.10	9.97
Pancreatic cancer	1990	5.22	4.97	5.40
Stomach cancer	1990	22.44	21.21	23.59
Colon and rectum cance	1991	22.44	21.49	23.16
Esophageal cancer	1991	8.09	6.38	8.84
Liver cancer	1991	9.31	8.56	10.15
Pancreatic cancer	1991	5.26	5.02	5.44
Stomach cancer	1991	22.12	20.96	23.31
Colon and rectum cance	1992	22.69	21.70	23.41
Esophageal cancer	1992	8.10	6.32	8.80
Liver cancer	1992	9.59	8.95	10.29
Pancreatic cancer	1992	5.31	5.06	5.48
Stomach cancer	1992	21.85	20.75	22.99
Colon and rectum cance	1993	23.24	22.23	23.87
Esophageal cancer	1993	8.15	6.35	8.82
Liver cancer	1993	9.80	9.23	10.41
Pancreatic cancer	1993	5.43	5.18	5.59
Stomach cancer	1993	21.78	20.72	22.77
Colon and rectum cance	1994	23.35	22.41	24.01
Esophageal cancer	1994	8.12	6.38	8.80
Liver cancer	1994	9.97	9.40	10.61
Pancreatic cancer	1994	5.46	5.21	5.62
Stomach cancer	1994	21.39	20.47	22.43
Colon and rectum cance	1995	23.47	22.47	24.10
Esophageal cancer	1995	8.10	6.30	8.72
Liver cancer	1995	10.06	9.44	10.79
Pancreatic cancer	1995	5.48	5.23	5.64
Stomach cancer	1995	20.97	20.07	21.88
Colon and rectum cance	1996	23.42	22.41	24.03
Esophageal cancer	1996	8.09	6.25	8.66
Liver cancer	1996	10.09	9.56	10.70
Pancreatic cancer	1996	5.47	5.21	5.62
Stomach cancer	1996	20.47	19.54	21.39
Colon and rectum cance	1997	23.44	22.43	24.07
Esophageal cancer	1997	8.08	6.20	8.65
Liver cancer	1997	10.05	9.54	10.58
Pancreatic cancer	1997	5.47	5.21	5.62
Stomach cancer	1997	20.06	19.17	20.96
Colon and rectum cance	1998	23.69	22.66	24.34
Esophageal cancer	1998	8.13	6.18	8.68
Liver cancer	1998	9.94	9.46	10.46
Pancreatic cancer	1998	5.51	5.26	5.67

Stomach cancer	1998	19.89	19.01	20.77
Colon and rectum cance	1999	24.08	22.99	24.72
Esophageal cancer	1999	8.22	6.16	8.80
Liver cancer	1999	9.78	9.29	10.30
Pancreatic cancer	1999	5.59	5.32	5.74
Stomach cancer	1999	19.87	18.99	20.70
Colon and rectum cance	2000	24.26	23.18	24.97
Esophageal cancer	2000	8.38	6.25	9.02
Liver cancer	2000	9.58	9.03	10.15
Pancreatic cancer	2000	5.64	5.38	5.82
Stomach cancer	2000	19.89	18.98	20.80
Colon and rectum cance	2001	24.42	23.15	25.16
Esophageal cancer	2001	8.50	6.14	9.20
Liver cancer	2001	9.16	8.70	9.60
Pancreatic cancer	2001	5.70	5.39	5.87
Stomach cancer	2001	19.90	18.85	20.85
Colon and rectum cance	2002	24.79	23.61	25.57
Esophageal cancer	2002	8.64	6.23	9.32
Liver cancer	2002	8.47	8.10	8.84
Pancreatic cancer	2002	5.79	5.51	5.97
Stomach cancer	2002	20.11	19.07	21.04
Colon and rectum cance	2003	25.21	23.96	26.03
Esophageal cancer	2003	8.77	6.29	9.47
Liver cancer	2003	7.70	7.37	8.03
Pancreatic cancer	2003	5.88	5.56	6.07
Stomach cancer	2003	20.38	19.35	21.36
Colon and rectum cance	2004	25.24	23.91	26.12
Esophageal cancer	2004	8.82	6.25	9.57
Liver cancer	2004	7.06	6.74	7.36
Pancreatic cancer	2004	5.91	5.57	6.10
Stomach cancer	2004	20.44	19.34	21.48
Colon and rectum cance	2005	25.50	24.08	26.36
Esophageal cancer	2005	8.70	6.25	9.39
Liver cancer	2005	6.73	6.41	7.05
Pancreatic cancer	2005	6.01	5.66	6.20
Stomach cancer	2005	20.23	19.10	21.28
Colon and rectum cance	2006	25.33	23.94	26.20
Esophageal cancer	2006	8.38	6.11	9.02
Liver cancer	2006	6.65	6.34	6.93
Pancreatic cancer	2006	6.02	5.69	6.23
Stomach cancer	2006	19.48	18.45	20.40
Colon and rectum cance	2007	25.51	24.15	26.43
Esophageal cancer	2007	8.12	6.04	8.73
Liver cancer	2007	6.59	6.29	6.86
Pancreatic cancer	2007	6.09	5.74	6.30
Stomach cancer	2007	19.06	18.08	20.07

Colon and rectum cance	2008	25.85	24.41	26.81
Esophageal cancer	2008	7.90	6.09	8.47
Liver cancer	2008	6.55	6.25	6.82
Pancreatic cancer	2008	6.17	5.82	6.39
Stomach cancer	2008	18.81	17.78	19.75
Colon and rectum cance	2009	26.01	24.52	26.94
Esophageal cancer	2009	7.69	6.06	8.21
Liver cancer	2009	6.52	6.21	6.81
Pancreatic cancer	2009	6.23	5.87	6.45
Stomach cancer	2009	18.50	17.45	19.43
Colon and rectum cance	2010	26.12	24.71	27.13
Esophageal cancer	2010	7.50	6.10	8.02
Liver cancer	2010	6.50	6.15	6.81
Pancreatic cancer	2010	6.29	5.91	6.54
Stomach cancer	2010	18.25	17.14	19.22
Colon and rectum cance	2011	26.10	24.57	27.13
Esophageal cancer	2011	7.26	6.03	7.78
Liver cancer	2011	6.48	6.16	6.77
Pancreatic cancer	2011	6.33	5.93	6.59
Stomach cancer	2011	17.81	16.71	18.81
Colon and rectum cance	2012	25.98	24.53	27.00
Esophageal cancer	2012	7.04	6.04	7.53
Liver cancer	2012	6.47	6.15	6.73
Pancreatic cancer	2012	6.35	5.97	6.61
Stomach cancer	2012	17.34	16.26	18.30
Colon and rectum cance	2013	25.88	24.35	27.00
Esophageal cancer	2013	6.82	5.91	7.31
Liver cancer	2013	6.47	6.14	6.73
Pancreatic cancer	2013	6.36	5.96	6.62
Stomach cancer	2013	16.86	15.76	17.94
Colon and rectum cance	2014	25.82	24.32	26.86
Esophageal cancer	2014	6.66	5.83	7.14
Liver cancer	2014	6.47	6.11	6.77
Pancreatic cancer	2014	6.37	5.96	6.64
Stomach cancer	2014	16.48	15.37	17.46
Colon and rectum cance	2015	25.95	24.35	27.07
Esophageal cancer	2015	6.55	5.85	7.04
Liver cancer	2015	6.47	6.08	6.83
Pancreatic cancer	2015	6.43	6.01	6.70
Stomach cancer	2015	16.17	15.02	17.23
Colon and rectum cance	2016	25.91	24.28	27.13
Esophageal cancer	2016	6.48	5.80	7.03
Liver cancer	2016	6.48	6.11	6.82
Pancreatic cancer	2016	6.44	6.00	6.75
Stomach cancer	2016	15.90	14.71	17.03
Colon and rectum cance	2017	26.00	24.20	27.57

Esophageal cancer	2017	6.44	5.79	7.00
Liver cancer	2017	6.49	6.03	6.98
Pancreatic cancer	2017	6.44	5.95	6.84
Stomach cancer	2017	15.69	14.40	16.97
Colon and rectum cance	2018	26.39	24.52	28.21
Esophageal cancer	2018	6.46	5.71	7.09
Liver cancer	2018	6.50	6.04	6.96
Pancreatic cancer	2018	6.51	6.01	6.98
Stomach cancer	2018	15.63	14.26	17.02
Colon and rectum cance	2019	26.71	24.58	28.89
Esophageal cancer	2019	6.51	5.69	7.25
Liver cancer	2019	6.51	5.95	7.16
Pancreatic cancer	2019	6.57	6.00	7.09
Stomach cancer	2019	15.59	14.11	17.15

Cause	Year	Age-standardised death rate (per 100 000 person-years)	95% CI (lower)	95% CI (upper)
Colon and rectum cance	1990	14.31	13.52	14.88
Esophageal cancer	1990	8.18	6.40	8.97
Liver cancer	1990	8.93	8.09	9.90
Pancreatic cancer	1990	5.34	5.07	5.52
Stomach cancer	1990	20.48	19.25	21.62
Colon and rectum cance	1991	14.27	13.49	14.83
Esophageal cancer	1991	8.19	6.45	8.93
Liver cancer	1991	9.15	8.33	10.12
Pancreatic cancer	1991	5.37	5.11	5.56
Stomach cancer	1991	20.08	18.92	21.20
Colon and rectum cance	1992	14.28	13.51	14.82
Esophageal cancer	1992	8.18	6.39	8.91
Liver cancer	1992	9.35	8.60	10.19
Pancreatic cancer	1992	5.42	5.13	5.60
Stomach cancer	1992	19.76	18.67	20.80
Colon and rectum cance	1993	14.44	13.69	14.95
Esophageal cancer	1993	8.22	6.35	8.88
Liver cancer	1993	9.56	8.82	10.35
Pancreatic cancer	1993	5.52	5.25	5.70
Stomach cancer	1993	19.61	18.64	20.57
Colon and rectum cance	1994	14.43	13.64	14.92
Esophageal cancer	1994	8.17	6.41	8.84
Liver cancer	1994	9.76	9.07	10.47
Pancreatic cancer	1994	5.56	5.26	5.72
Stomach cancer	1994	19.24	18.28	20.21
Colon and rectum cance	1995	14.38	13.63	14.87
Esophageal cancer	1995	8.13	6.33	8.74
Liver cancer	1995	9.89	9.25	10.55
Pancreatic cancer	1995	5.57	5.29	5.74
Stomach cancer	1995	18.78	17.84	19.71
Colon and rectum cance	1996	14.28	13.52	14.76
Esophageal cancer	1996	8.10	6.31	8.74
Liver cancer	1996	9.95	9.32	10.60
Pancreatic cancer	1996	5.56	5.29	5.73
Stomach cancer	1996	18.29	17.43	19.15
Colon and rectum cance	1997	14.20	13.43	14.68
Esophageal cancer	1997	8.08	6.24	8.64
Liver cancer	1997	9.89	9.35	10.48
Pancreatic cancer	1997	5.56	5.28	5.73
Stomach cancer	1997	17.86	16.96	18.67
Colon and rectum cance	1998	14.21	13.43	14.69
Esophageal cancer	1998	8.09	6.18	8.68
Liver cancer	1998	9.83	9.27	10.43
Pancreatic cancer	1998	5.59	5.31	5.75
Stomach cancer	1998	17.60	16.74	18.41

Colon and rectum cance	1999	14.34	13.54	14.82
Esophageal cancer	1999	8.17	6.17	8.77
Liver cancer	1999	9.69	9.13	10.25
Pancreatic cancer	1999	5.66	5.38	5.83
Stomach cancer	1999	17.53	16.68	18.33
Colon and rectum cance	2000	14.40	13.56	14.92
Esophageal cancer	2000	8.31	6.20	8.98
Liver cancer	2000	9.29	8.72	9.85
Pancreatic cancer	2000	5.72	5.41	5.89
Stomach cancer	2000	17.55	16.69	18.42
Colon and rectum cance	2001	14.43	13.55	14.94
Esophageal cancer	2001	8.41	6.10	9.09
Liver cancer	2001	8.61	8.05	9.14
Pancreatic cancer	2001	5.77	5.46	5.95
Stomach cancer	2001	17.51	16.54	18.36
Colon and rectum cance	2002	14.55	13.64	15.09
Esophageal cancer	2002	8.52	6.17	9.21
Liver cancer	2002	7.78	7.35	8.20
Pancreatic cancer	2002	5.86	5.52	6.05
Stomach cancer	2002	17.62	16.64	18.51
Colon and rectum cance	2003	14.62	13.70	15.17
Esophageal cancer	2003	8.61	6.15	9.34
Liver cancer	2003	7.10	6.76	7.45
Pancreatic cancer	2003	5.93	5.60	6.13
Stomach cancer	2003	17.68	16.80	18.56
Colon and rectum cance	2004	14.54	13.61	15.12
Esophageal cancer	2004	8.63	6.08	9.34
Liver cancer	2004	6.61	6.28	6.91
Pancreatic cancer	2004	5.97	5.63	6.17
Stomach cancer	2004	17.60	16.67	18.49
Colon and rectum cance	2005	14.58	13.66	15.17
Esophageal cancer	2005	8.49	6.09	9.19
Liver cancer	2005	6.38	6.08	6.69
Pancreatic cancer	2005	6.06	5.71	6.27
Stomach cancer	2005	17.27	16.33	18.17
Colon and rectum cance	2006	14.39	13.48	14.96
Esophageal cancer	2006	8.15	5.97	8.76
Liver cancer	2006	6.27	5.96	6.54
Pancreatic cancer	2006	6.08	5.71	6.28
Stomach cancer	2006	16.49	15.54	17.30
Colon and rectum cance	2007	14.31	13.32	14.90
Esophageal cancer	2007	7.85	5.91	8.45
Liver cancer	2007	6.20	5.88	6.45
Pancreatic cancer	2007	6.13	5.77	6.36
Stomach cancer	2007	15.93	15.00	16.71
Colon and rectum cance	2008	14.30	13.39	14.93
Esophageal cancer	2008	7.61	5.85	8.17

Liver cancer	2008	6.20	5.87	6.46
Pancreatic cancer	2008	6.21	5.84	6.45
Stomach cancer	2008	15.53	14.57	16.29
Colon and rectum cance	2009	14.19	13.21	14.75
Esophageal cancer	2009	7.37	5.84	7.87
Liver cancer	2009	6.12	5.79	6.39
Pancreatic cancer	2009	6.26	5.86	6.49
Stomach cancer	2009	15.10	14.16	15.85
Colon and rectum cance	2010	14.12	13.17	14.70
Esophageal cancer	2010	7.17	5.78	7.68
Liver cancer	2010	6.05	5.72	6.33
Pancreatic cancer	2010	6.33	5.93	6.58
Stomach cancer	2010	14.74	13.83	15.51
Colon and rectum cance	2011	14.00	13.02	14.63
Esophageal cancer	2011	6.93	5.84	7.44
Liver cancer	2011	5.96	5.62	6.27
Pancreatic cancer	2011	6.36	5.93	6.64
Stomach cancer	2011	14.27	13.31	15.11
Colon and rectum cance	2012	13.88	12.88	14.51
Esophageal cancer	2012	6.70	5.78	7.16
Liver cancer	2012	5.91	5.60	6.20
Pancreatic cancer	2012	6.39	5.97	6.66
Stomach cancer	2012	13.83	13.00	14.62
Colon and rectum cance	2013	13.74	12.77	14.37
Esophageal cancer	2013	6.49	5.66	6.92
Liver cancer	2013	5.90	5.54	6.21
Pancreatic cancer	2013	6.40	5.96	6.67
Stomach cancer	2013	13.36	12.44	14.12
Colon and rectum cance	2014	13.62	12.65	14.26
Esophageal cancer	2014	6.32	5.57	6.80
Liver cancer	2014	5.93	5.59	6.24
Pancreatic cancer	2014	6.41	5.97	6.69
Stomach cancer	2014	12.96	12.08	13.72
Colon and rectum cance	2015	13.61	12.63	14.25
Esophageal cancer	2015	6.20	5.54	6.71
Liver cancer	2015	5.97	5.62	6.29
Pancreatic cancer	2015	6.46	6.01	6.76
Stomach cancer	2015	12.66	11.80	13.42
Colon and rectum cance	2016	13.55	12.49	14.27
Esophageal cancer	2016	6.13	5.47	6.66
Liver cancer	2016	5.98	5.57	6.37
Pancreatic cancer	2016	6.47	6.00	6.81
Stomach cancer	2016	12.43	11.41	13.33
Colon and rectum cance	2017	13.50	12.46	14.23
Esophageal cancer	2017	6.07	5.37	6.58
Liver cancer	2017	5.96	5.52	6.37
Pancreatic cancer	2017	6.48	6.04	6.82

Stomach cancer	2017	12.15	11.20	12.98
Colon and rectum cance	2018	13.59	12.46	14.43
Esophageal cancer	2018	6.08	5.42	6.69
Liver cancer	2018	5.94	5.47	6.42
Pancreatic cancer	2018	6.55	6.07	6.97
Stomach cancer	2018	11.98	10.97	12.98
Colon and rectum cance	2019	13.69	12.60	14.51
Esophageal cancer	2019	6.11	5.38	6.76
Liver cancer	2019	5.95	5.44	6.44
Pancreatic cancer	2019	6.62	6.11	7.06
Stomach cancer	2019	11.88	10.82	12.82

Location_name	Sex_name	Cause_name	Year	Age-standardised incidence rate (per 100 000 person-years)	95% CI (lower)	95% CI (upper)
Afghanistan	Both	Colon and rectum cancer	2019	8.69	6.04	11.55
Afghanistan	Both	Esophageal cancer	2019	6.55	2.11	9.17
Afghanistan	Both	Liver cancer	2019	9.82	7.63	12.44
Afghanistan	Both	Pancreatic cancer	2019	2.57	1.78	3.71
Afghanistan	Both	Stomach cancer	2019	27.69	19.94	34.87
Albania	Both	Colon and rectum cancer	2019	15.15	11.40	19.90
Albania	Both	Esophageal cancer	2019	1.38	1.00	2.01
Albania	Both	Liver cancer	2019	6.58	4.82	8.76
Albania	Both	Pancreatic cancer	2019	6.53	4.90	8.55
Albania	Both	Stomach cancer	2019	11.60	8.72	15.35
Algeria	Both	Colon and rectum cancer	2019	10.52	8.35	13.05
Algeria	Both	Esophageal cancer	2019	1.08	0.79	1.37
Algeria	Both	Liver cancer	2019	2.21	1.73	2.78
Algeria	Both	Pancreatic cancer	2019	4.05	3.27	4.94
Algeria	Both	Stomach cancer	2019	4.98	4.06	6.09
American Samoa	Both	Colon and rectum cancer	2019	20.81	17.56	24.70
American Samoa	Both	Esophageal cancer	2019	1.42	1.11	1.68
American Samoa	Both	Liver cancer	2019	6.88	5.69	8.35
American Samoa	Both	Pancreatic cancer	2019	4.95	4.10	5.90
American Samoa	Both	Stomach cancer	2019	15.46	13.04	18.30
Andorra	Both	Colon and rectum cancer	2019	56.65	42.79	71.90
Andorra	Both	Esophageal cancer	2019	4.15	3.02	5.36
Andorra	Both	Liver cancer	2019	10.95	8.23	14.37
Andorra	Both	Pancreatic cancer	2019	10.31	7.83	13.25
Andorra	Both	Stomach cancer	2019	12.10	9.15	15.74
Angola	Both	Colon and rectum cancer	2019	10.02	8.06	12.55
Angola	Both	Esophageal cancer	2019	8.24	4.44	11.10
Angola	Both	Liver cancer	2019	2.39	1.92	2.99
Angola	Both	Pancreatic cancer	2019	3.19	2.55	4.04
Angola	Both	Stomach cancer	2019	8.39	6.79	10.61
Antigua and Barbuda	Both	Colon and rectum cancer	2019	25.58	22.05	29.43
Antigua and Barbuda	Both	Esophageal cancer	2019	2.98	2.53	3.54
Antigua and Barbuda	Both	Liver cancer	2019	2.69	2.29	3.13
Antigua and Barbuda	Both	Pancreatic cancer	2019	6.07	5.13	7.13
Antigua and Barbuda	Both	Stomach cancer	2019	11.37	9.74	13.05
Argentina	Both	Colon and rectum cancer	2019	34.68	27.56	43.36
Argentina	Both	Esophageal cancer	2019	4.97	3.93	6.25
Argentina	Both	Liver cancer	2019	2.05	1.61	2.56
Argentina	Both	Pancreatic cancer	2019	11.54	9.02	14.41
Argentina	Both	Stomach cancer	2019	9.89	7.87	12.44
Armenia	Both	Colon and rectum cancer	2019	21.37	17.85	25.01
Armenia	Both	Esophageal cancer	2019	1.53	1.26	1.84
Armenia	Both	Liver cancer	2019	6.60	5.47	7.85
Armenia	Both	Pancreatic cancer	2019	10.16	8.45	12.07
Armenia	Both	Stomach cancer	2019	13.40	11.19	15.74
Australia	Both	Colon and rectum cancer	2019	47.04	36.95	59.55
Australia	Both	Esophageal cancer	2019	4.34	3.34	5.61
Australia	Both	Liver cancer	2019	4.52	3.48	5.84
Australia	Both	Pancreatic cancer	2019	8.85	6.85	11.10
Australia	Both	Stomach cancer	2019	7.01	5.46	8.81
Austria	Both	Colon and rectum cancer	2019	33.07	26.71	40.55
Austria	Both	Esophageal cancer	2019	3.02	2.40	3.77
Austria	Both	Liver cancer	2019	5.50	4.41	6.89
Austria	Both	Pancreatic cancer	2019	10.83	8.81	13.17
Austria	Both	Stomach cancer	2019	8.43	6.83	10.22
Azerbaijan	Both	Colon and rectum cancer	2019	16.32	13.28	20.08
Azerbaijan	Both	Esophageal cancer	2019	8.67	6.49	13.36
Azerbaijan	Both	Liver cancer	2019	4.07	3.11	5.46
Azerbaijan	Both	Pancreatic cancer	2019	7.07	6.05	8.58
Azerbaijan	Both	Stomach cancer	2019	21.72	18.14	26.38
Bahamas	Both	Colon and rectum cancer	2019	27.96	22.83	34.01
Bahamas	Both	Esophageal cancer	2019	5.06	4.03	6.28

Bahamas	Both	Liver cancer	2019	3.10	2.56	3.81
Bahamas	Both	Pancreatic cancer	2019	4.53	3.67	5.58
Bahamas	Both	Stomach cancer	2019	9.35	7.61	11.37
Bahrain	Both	Colon and rectum cancer	2019	16.96	13.23	21.08
Bahrain	Both	Esophageal cancer	2019	1.94	1.45	2.48
Bahrain	Both	Liver cancer	2019	5.53	4.39	6.91
Bahrain	Both	Pancreatic cancer	2019	7.19	5.73	8.76
Bahrain	Both	Stomach cancer	2019	6.46	5.24	7.82
Bangladesh	Both	Colon and rectum cancer	2019	5.63	3.90	8.00
Bangladesh	Both	Esophageal cancer	2019	3.81	2.71	5.73
Bangladesh	Both	Liver cancer	2019	2.58	2.03	3.21
Bangladesh	Both	Pancreatic cancer	2019	2.07	1.30	3.09
Bangladesh	Both	Stomach cancer	2019	6.30	4.83	8.27
Barbados	Both	Colon and rectum cancer	2019	39.83	33.03	47.38
Barbados	Both	Esophageal cancer	2019	4.92	3.97	5.96
Barbados	Both	Liver cancer	2019	2.63	2.17	3.14
Barbados	Both	Pancreatic cancer	2019	7.78	6.31	9.26
Barbados	Both	Stomach cancer	2019	10.91	9.00	12.84
Belarus	Both	Colon and rectum cancer	2019	34.12	27.11	43.36
Belarus	Both	Esophageal cancer	2019	3.05	2.31	4.04
Belarus	Both	Liver cancer	2019	2.49	1.81	3.34
Belarus	Both	Pancreatic cancer	2019	7.07	5.52	9.16
Belarus	Both	Stomach cancer	2019	17.86	14.13	22.99
Belgium	Both	Colon and rectum cancer	2019	39.33	30.86	49.46
Belgium	Both	Esophageal cancer	2019	5.26	4.04	6.68
Belgium	Both	Liver cancer	2019	4.27	3.36	5.42
Belgium	Both	Pancreatic cancer	2019	9.28	7.25	11.67
Belgium	Both	Stomach cancer	2019	7.05	5.59	8.85
Belize	Both	Colon and rectum cancer	2019	14.64	12.76	16.95
Belize	Both	Esophageal cancer	2019	2.38	2.03	2.77
Belize	Both	Liver cancer	2019	3.04	2.60	3.52
Belize	Both	Pancreatic cancer	2019	6.09	5.10	7.08
Belize	Both	Stomach cancer	2019	10.12	8.74	11.66
Benin	Both	Colon and rectum cancer	2019	7.78	6.25	9.85
Benin	Both	Esophageal cancer	2019	4.86	2.88	6.63
Benin	Both	Liver cancer	2019	4.78	3.57	6.37
Benin	Both	Pancreatic cancer	2019	4.61	3.65	5.86
Benin	Both	Stomach cancer	2019	12.37	9.96	15.43
Bermuda	Both	Colon and rectum cancer	2019	43.29	36.11	52.78
Bermuda	Both	Esophageal cancer	2019	4.44	3.67	5.46
Bermuda	Both	Liver cancer	2019	2.09	1.73	2.56
Bermuda	Both	Pancreatic cancer	2019	9.39	7.76	11.48
Bermuda	Both	Stomach cancer	2019	6.70	5.59	8.00
Bhutan	Both	Colon and rectum cancer	2019	8.03	4.91	10.87
Bhutan	Both	Esophageal cancer	2019	4.69	3.30	6.46
Bhutan	Both	Liver cancer	2019	3.04	2.11	4.29
Bhutan	Both	Pancreatic cancer	2019	3.05	1.75	4.65
Bhutan	Both	Stomach cancer	2019	7.96	5.82	10.37
Bolivia (Plurinational State of)	Both	Colon and rectum cancer	2019	16.89	11.60	22.44
Bolivia (Plurinational State of)	Both	Esophageal cancer	2019	2.33	1.82	2.92
Bolivia (Plurinational State of)	Both	Liver cancer	2019	4.58	3.41	5.93
Bolivia (Plurinational State of)	Both	Pancreatic cancer	2019	4.94	3.40	6.66
Bolivia (Plurinational State of)	Both	Stomach cancer	2019	34.02	26.85	42.02
Bosnia and Herzegovina	Both	Colon and rectum cancer	2019	34.91	27.54	43.53
Bosnia and Herzegovina	Both	Esophageal cancer	2019	2.01	1.56	2.54
Bosnia and Herzegovina	Both	Liver cancer	2019	7.53	5.99	9.46
Bosnia and Herzegovina	Both	Pancreatic cancer	2019	9.74	7.78	12.10
Bosnia and Herzegovina	Both	Stomach cancer	2019	10.78	8.52	13.61
Botswana	Both	Colon and rectum cancer	2019	18.75	13.48	24.55
Botswana	Both	Esophageal cancer	2019	12.72	7.73	16.72
Botswana	Both	Liver cancer	2019	1.47	1.07	1.95
Botswana	Both	Pancreatic cancer	2019	7.49	5.52	10.04
Botswana	Both	Stomach cancer	2019	8.66	6.60	10.98
Brazil	Both	Colon and rectum cancer	2019	17.77	16.64	18.65

Brazil	Both	Esophageal cancer	2019	5.20	4.90	5.45
Brazil	Both	Liver cancer	2019	2.37	2.23	2.49
Brazil	Both	Pancreatic cancer	2019	6.16	5.68	6.53
Brazil	Both	Stomach cancer	2019	10.23	9.60	10.71
Brunei Darussalam	Both	Colon and rectum cancer	2019	49.38	43.44	55.88
Brunei Darussalam	Both	Esophageal cancer	2019	3.25	2.76	3.99
Brunei Darussalam	Both	Liver cancer	2019	11.75	9.96	13.78
Brunei Darussalam	Both	Pancreatic cancer	2019	9.24	8.03	10.51
Brunei Darussalam	Both	Stomach cancer	2019	17.31	15.13	19.53
Bulgaria	Both	Colon and rectum cancer	2019	43.84	35.01	54.21
Bulgaria	Both	Esophageal cancer	2019	1.98	1.53	2.50
Bulgaria	Both	Liver cancer	2019	4.50	3.58	5.58
Bulgaria	Both	Pancreatic cancer	2019	10.93	8.61	13.71
Bulgaria	Both	Stomach cancer	2019	11.59	9.24	14.50
Burkina Faso	Both	Colon and rectum cancer	2019	7.42	5.92	9.38
Burkina Faso	Both	Esophageal cancer	2019	4.78	2.78	6.12
Burkina Faso	Both	Liver cancer	2019	2.18	1.66	2.75
Burkina Faso	Both	Pancreatic cancer	2019	3.40	2.60	4.30
Burkina Faso	Both	Stomach cancer	2019	14.01	11.52	16.83
Burundi	Both	Colon and rectum cancer	2019	7.35	5.32	10.43
Burundi	Both	Esophageal cancer	2019	11.49	7.73	16.36
Burundi	Both	Liver cancer	2019	2.89	1.98	4.53
Burundi	Both	Pancreatic cancer	2019	2.17	1.61	2.98
Burundi	Both	Stomach cancer	2019	8.43	6.59	10.70
Cabo Verde	Both	Colon and rectum cancer	2019	13.39	10.67	15.73
Cabo Verde	Both	Esophageal cancer	2019	15.57	13.02	18.14
Cabo Verde	Both	Liver cancer	2019	11.56	9.52	13.94
Cabo Verde	Both	Pancreatic cancer	2019	10.12	7.94	12.33
Cabo Verde	Both	Stomach cancer	2019	23.79	20.66	26.99
Cambodia	Both	Colon and rectum cancer	2019	16.67	13.29	20.00
Cambodia	Both	Esophageal cancer	2019	2.82	2.17	3.89
Cambodia	Both	Liver cancer	2019	9.20	7.26	11.26
Cambodia	Both	Pancreatic cancer	2019	3.52	2.82	4.19
Cambodia	Both	Stomach cancer	2019	8.88	7.13	10.59
Cameroon	Both	Colon and rectum cancer	2019	11.18	8.58	14.61
Cameroon	Both	Esophageal cancer	2019	5.64	3.10	7.97
Cameroon	Both	Liver cancer	2019	0.65	0.47	0.87
Cameroon	Both	Pancreatic cancer	2019	7.49	5.31	10.17
Cameroon	Both	Stomach cancer	2019	13.01	10.12	16.59
Canada	Both	Colon and rectum cancer	2019	49.60	38.84	62.84
Canada	Both	Esophageal cancer	2019	4.42	3.44	5.59
Canada	Both	Liver cancer	2019	4.75	3.62	6.11
Canada	Both	Pancreatic cancer	2019	9.82	7.63	12.32
Canada	Both	Stomach cancer	2019	7.99	6.25	10.04
Central African Republic	Both	Colon and rectum cancer	2019	6.31	4.65	8.68
Central African Republic	Both	Esophageal cancer	2019	10.28	5.74	14.14
Central African Republic	Both	Liver cancer	2019	3.18	2.08	4.66
Central African Republic	Both	Pancreatic cancer	2019	2.06	1.47	2.78
Central African Republic	Both	Stomach cancer	2019	11.79	8.93	15.28
Chad	Both	Colon and rectum cancer	2019	7.32	5.68	9.41
Chad	Both	Esophageal cancer	2019	4.64	2.64	6.33
Chad	Both	Liver cancer	2019	5.31	4.05	6.84
Chad	Both	Pancreatic cancer	2019	2.66	2.13	3.31
Chad	Both	Stomach cancer	2019	14.88	11.80	18.40
Chile	Both	Colon and rectum cancer	2019	25.10	20.08	31.56
Chile	Both	Esophageal cancer	2019	3.87	3.02	4.89
Chile	Both	Liver cancer	2019	2.98	2.31	3.75
Chile	Both	Pancreatic cancer	2019	7.55	5.87	9.55
Chile	Both	Stomach cancer	2019	19.87	15.80	24.84
China	Both	Colon and rectum cancer	2019	30.55	26.37	35.50
China	Both	Esophageal cancer	2019	13.90	10.70	16.52
China	Both	Liver cancer	2019	10.46	8.74	12.42
China	Both	Pancreatic cancer	2019	5.78	4.94	6.69
China	Both	Stomach cancer	2019	30.64	25.82	36.15

Colombia	Both	Colon and rectum cancer	2019	17.15	13.28	21.89
Colombia	Both	Esophageal cancer	2019	1.91	1.44	2.47
Colombia	Both	Liver cancer	2019	2.51	1.91	3.22
Colombia	Both	Pancreatic cancer	2019	4.55	3.51	5.79
Colombia	Both	Stomach cancer	2019	14.99	11.67	19.02
Comoros	Both	Colon and rectum cancer	2019	8.96	6.63	11.43
Comoros	Both	Esophageal cancer	2019	11.25	7.65	15.77
Comoros	Both	Liver cancer	2019	2.79	1.94	4.33
Comoros	Both	Pancreatic cancer	2019	3.64	2.81	4.59
Comoros	Both	Stomach cancer	2019	6.67	5.30	8.41
Congo	Both	Colon and rectum cancer	2019	11.94	9.05	15.43
Congo	Both	Esophageal cancer	2019	10.05	5.57	13.71
Congo	Both	Liver cancer	2019	2.89	2.11	3.94
Congo	Both	Pancreatic cancer	2019	5.14	3.38	7.30
Congo	Both	Stomach cancer	2019	8.09	6.57	10.02
Cook Islands	Both	Colon and rectum cancer	2019	12.42	10.26	15.12
Cook Islands	Both	Esophageal cancer	2019	2.65	2.20	3.18
Cook Islands	Both	Liver cancer	2019	11.37	9.15	13.93
Cook Islands	Both	Pancreatic cancer	2019	4.82	4.05	5.71
Cook Islands	Both	Stomach cancer	2019	7.48	6.20	9.09
Costa Rica	Both	Colon and rectum cancer	2019	29.18	22.72	37.17
Costa Rica	Both	Esophageal cancer	2019	1.73	1.32	2.22
Costa Rica	Both	Liver cancer	2019	5.14	3.97	6.51
Costa Rica	Both	Pancreatic cancer	2019	6.95	5.40	8.75
Costa Rica	Both	Stomach cancer	2019	23.26	18.21	29.36
Côte d'Ivoire	Both	Colon and rectum cancer	2019	9.59	7.65	11.87
Côte d'Ivoire	Both	Esophageal cancer	2019	5.04	2.79	6.84
Côte d'Ivoire	Both	Liver cancer	2019	4.73	3.50	6.43
Côte d'Ivoire	Both	Pancreatic cancer	2019	5.12	3.99	6.39
Côte d'Ivoire	Both	Stomach cancer	2019	13.33	10.81	16.37
Croatia	Both	Colon and rectum cancer	2019	50.47	39.91	62.58
Croatia	Both	Esophageal cancer	2019	2.77	2.10	3.61
Croatia	Both	Liver cancer	2019	3.73	2.92	4.75
Croatia	Both	Pancreatic cancer	2019	9.58	7.60	12.02
Croatia	Both	Stomach cancer	2019	12.24	9.73	15.18
Cuba	Both	Colon and rectum cancer	2019	34.57	28.25	42.05
Cuba	Both	Esophageal cancer	2019	5.21	4.14	6.46
Cuba	Both	Liver cancer	2019	2.34	1.89	2.90
Cuba	Both	Pancreatic cancer	2019	6.04	4.92	7.40
Cuba	Both	Stomach cancer	2019	6.61	5.38	8.08
Cyprus	Both	Colon and rectum cancer	2019	38.15	33.10	43.43
Cyprus	Both	Esophageal cancer	2019	1.41	1.08	1.69
Cyprus	Both	Liver cancer	2019	3.69	3.16	4.32
Cyprus	Both	Pancreatic cancer	2019	8.07	6.99	9.24
Cyprus	Both	Stomach cancer	2019	9.00	7.85	10.27
Czechia	Both	Colon and rectum cancer	2019	42.14	34.45	51.15
Czechia	Both	Esophageal cancer	2019	3.36	2.68	4.19
Czechia	Both	Liver cancer	2019	2.96	2.42	3.65
Czechia	Both	Pancreatic cancer	2019	11.85	9.59	14.34
Czechia	Both	Stomach cancer	2019	8.00	6.50	9.71
Democratic People's Republic of Korea	Both	Colon and rectum cancer	2019	15.42	11.47	19.91
Democratic People's Republic of Korea	Both	Esophageal cancer	2019	8.96	6.96	11.60
Democratic People's Republic of Korea	Both	Liver cancer	2019	10.23	7.66	13.36
Democratic People's Republic of Korea	Both	Pancreatic cancer	2019	3.80	2.88	4.81
Democratic People's Republic of Korea	Both	Stomach cancer	2019	23.40	18.56	28.92
Democratic Republic of the Congo	Both	Colon and rectum cancer	2019	6.36	4.24	9.58
Democratic Republic of the Congo	Both	Esophageal cancer	2019	8.17	4.29	11.95
Democratic Republic of the Congo	Both	Liver cancer	2019	2.14	1.65	2.79
Democratic Republic of the Congo	Both	Pancreatic cancer	2019	2.26	1.72	2.89
Democratic Republic of the Congo	Both	Stomach cancer	2019	7.62	5.91	9.63
Denmark	Both	Colon and rectum cancer	2019	48.41	37.76	61.15
Denmark	Both	Esophageal cancer	2019	5.01	3.88	6.50
Denmark	Both	Liver cancer	2019	3.98	3.11	5.10
Denmark	Both	Pancreatic cancer	2019	10.19	7.86	12.92

Denmark	Both	Stomach cancer	2019	6.16	4.77	7.80
Djibouti	Both	Colon and rectum cancer	2019	11.92	9.06	15.80
Djibouti	Both	Esophageal cancer	2019	11.37	7.36	17.27
Djibouti	Both	Liver cancer	2019	3.22	2.07	5.23
Djibouti	Both	Pancreatic cancer	2019	4.02	2.88	5.50
Djibouti	Both	Stomach cancer	2019	7.60	5.84	10.13
Dominica	Both	Colon and rectum cancer	2019	19.85	16.29	24.15
Dominica	Both	Esophageal cancer	2019	4.87	3.91	6.02
Dominica	Both	Liver cancer	2019	3.18	2.58	3.95
Dominica	Both	Pancreatic cancer	2019	8.04	6.39	9.81
Dominica	Both	Stomach cancer	2019	19.79	16.20	24.01
Dominican Republic	Both	Colon and rectum cancer	2019	17.08	12.37	22.31
Dominican Republic	Both	Esophageal cancer	2019	2.43	1.68	3.38
Dominican Republic	Both	Liver cancer	2019	4.64	3.16	6.99
Dominican Republic	Both	Pancreatic cancer	2019	3.56	2.43	4.83
Dominican Republic	Both	Stomach cancer	2019	8.64	6.54	11.38
Ecuador	Both	Colon and rectum cancer	2019	19.25	15.35	24.31
Ecuador	Both	Esophageal cancer	2019	1.52	1.18	2.03
Ecuador	Both	Liver cancer	2019	3.40	2.72	4.37
Ecuador	Both	Pancreatic cancer	2019	5.46	4.35	6.86
Ecuador	Both	Stomach cancer	2019	22.30	17.83	28.19
Egypt	Both	Colon and rectum cancer	2019	9.78	7.07	13.43
Egypt	Both	Esophageal cancer	2019	1.50	1.04	2.01
Egypt	Both	Liver cancer	2019	20.92	15.09	28.51
Egypt	Both	Pancreatic cancer	2019	4.33	2.99	6.01
Egypt	Both	Stomach cancer	2019	5.01	3.82	6.52
El Salvador	Both	Colon and rectum cancer	2019	14.12	10.72	18.33
El Salvador	Both	Esophageal cancer	2019	1.59	1.20	2.06
El Salvador	Both	Liver cancer	2019	1.99	1.50	2.59
El Salvador	Both	Pancreatic cancer	2019	5.03	3.77	6.48
El Salvador	Both	Stomach cancer	2019	17.47	13.38	22.37
Equatorial Guinea	Both	Colon and rectum cancer	2019	15.60	9.84	22.43
Equatorial Guinea	Both	Esophageal cancer	2019	8.49	4.42	14.49
Equatorial Guinea	Both	Liver cancer	2019	3.17	1.87	4.63
Equatorial Guinea	Both	Pancreatic cancer	2019	5.91	3.63	8.84
Equatorial Guinea	Both	Stomach cancer	2019	6.09	4.48	8.40
Eritrea	Both	Colon and rectum cancer	2019	10.34	8.12	13.21
Eritrea	Both	Esophageal cancer	2019	13.20	8.37	18.14
Eritrea	Both	Liver cancer	2019	3.10	2.17	4.41
Eritrea	Both	Pancreatic cancer	2019	2.95	2.01	4.08
Eritrea	Both	Stomach cancer	2019	9.93	7.57	12.66
Estonia	Both	Colon and rectum cancer	2019	42.21	33.40	52.75
Estonia	Both	Esophageal cancer	2019	2.98	2.24	3.83
Estonia	Both	Liver cancer	2019	3.71	2.84	4.71
Estonia	Both	Pancreatic cancer	2019	10.44	8.15	13.18
Estonia	Both	Stomach cancer	2019	15.83	12.34	20.28
Eswatini	Both	Colon and rectum cancer	2019	14.40	9.82	19.67
Eswatini	Both	Esophageal cancer	2019	15.14	9.70	20.59
Eswatini	Both	Liver cancer	2019	18.43	5.61	32.97
Eswatini	Both	Pancreatic cancer	2019	7.66	5.25	10.57
Eswatini	Both	Stomach cancer	2019	8.57	6.54	11.12
Ethiopia	Both	Colon and rectum cancer	2019	7.70	5.78	10.75
Ethiopia	Both	Esophageal cancer	2019	2.67	2.11	3.72
Ethiopia	Both	Liver cancer	2019	2.73	2.21	3.47
Ethiopia	Both	Pancreatic cancer	2019	1.47	0.98	2.12
Ethiopia	Both	Stomach cancer	2019	6.17	5.09	7.73
Fiji	Both	Colon and rectum cancer	2019	13.52	10.86	16.56
Fiji	Both	Esophageal cancer	2019	2.67	1.51	3.45
Fiji	Both	Liver cancer	2019	6.04	4.67	7.62
Fiji	Both	Pancreatic cancer	2019	4.08	3.22	5.07
Fiji	Both	Stomach cancer	2019	7.02	5.63	8.76
Finland	Both	Colon and rectum cancer	2019	31.46	24.55	40.00
Finland	Both	Esophageal cancer	2019	3.21	2.44	4.14
Finland	Both	Liver cancer	2019	5.54	4.35	7.14

Finland	Both	Pancreatic cancer	2019	11.70	9.18	14.78
Finland	Both	Stomach cancer	2019	7.17	5.60	8.98
France	Both	Colon and rectum cancer	2019	38.47	30.10	49.30
France	Both	Esophageal cancer	2019	4.62	3.58	5.95
France	Both	Liver cancer	2019	6.66	5.05	8.72
France	Both	Pancreatic cancer	2019	9.78	7.61	12.39
France	Both	Stomach cancer	2019	6.93	5.38	8.78
Gabon	Both	Colon and rectum cancer	2019	16.38	12.14	20.32
Gabon	Both	Esophageal cancer	2019	10.56	6.01	13.93
Gabon	Both	Liver cancer	2019	3.27	2.15	4.67
Gabon	Both	Pancreatic cancer	2019	7.69	5.35	10.57
Gabon	Both	Stomach cancer	2019	7.68	6.02	9.42
Gambia	Both	Colon and rectum cancer	2019	6.84	4.99	9.15
Gambia	Both	Esophageal cancer	2019	2.09	1.57	2.66
Gambia	Both	Liver cancer	2019	38.21	27.53	49.67
Gambia	Both	Pancreatic cancer	2019	3.28	2.24	4.51
Gambia	Both	Stomach cancer	2019	5.21	4.14	6.41
Georgia	Both	Colon and rectum cancer	2019	19.13	16.02	22.48
Georgia	Both	Esophageal cancer	2019	1.80	1.47	2.17
Georgia	Both	Liver cancer	2019	3.52	2.87	4.32
Georgia	Both	Pancreatic cancer	2019	6.17	5.09	7.37
Georgia	Both	Stomach cancer	2019	14.37	12.09	17.01
Germany	Both	Colon and rectum cancer	2019	41.40	32.67	53.84
Germany	Both	Esophageal cancer	2019	5.48	4.24	7.20
Germany	Both	Liver cancer	2019	5.07	3.96	6.44
Germany	Both	Pancreatic cancer	2019	11.89	9.28	15.27
Germany	Both	Stomach cancer	2019	9.99	7.75	12.87
Ghana	Both	Colon and rectum cancer	2019	9.51	7.57	11.94
Ghana	Both	Esophageal cancer	2019	3.32	2.29	4.30
Ghana	Both	Liver cancer	2019	5.68	4.31	7.20
Ghana	Both	Pancreatic cancer	2019	9.07	6.94	11.97
Ghana	Both	Stomach cancer	2019	7.82	6.24	9.74
Greece	Both	Colon and rectum cancer	2019	33.21	26.10	42.09
Greece	Both	Esophageal cancer	2019	1.57	1.22	2.00
Greece	Both	Liver cancer	2019	3.52	2.78	4.42
Greece	Both	Pancreatic cancer	2019	10.03	7.85	12.72
Greece	Both	Stomach cancer	2019	11.03	8.67	13.97
Greenland	Both	Colon and rectum cancer	2019	47.44	39.57	55.72
Greenland	Both	Esophageal cancer	2019	12.96	10.41	15.38
Greenland	Both	Liver cancer	2019	6.20	4.93	7.74
Greenland	Both	Pancreatic cancer	2019	18.89	15.51	22.26
Greenland	Both	Stomach cancer	2019	11.26	9.25	13.40
Grenada	Both	Colon and rectum cancer	2019	27.86	25.13	30.69
Grenada	Both	Esophageal cancer	2019	6.48	5.68	7.32
Grenada	Both	Liver cancer	2019	2.96	2.61	3.36
Grenada	Both	Pancreatic cancer	2019	9.30	8.18	10.51
Grenada	Both	Stomach cancer	2019	10.36	9.31	11.53
Guam	Both	Colon and rectum cancer	2019	21.01	17.62	24.81
Guam	Both	Esophageal cancer	2019	2.61	2.05	3.20
Guam	Both	Liver cancer	2019	5.89	4.80	7.19
Guam	Both	Pancreatic cancer	2019	5.12	4.25	6.11
Guam	Both	Stomach cancer	2019	6.09	5.08	7.26
Guatemala	Both	Colon and rectum cancer	2019	11.51	9.18	14.40
Guatemala	Both	Esophageal cancer	2019	2.05	1.61	2.59
Guatemala	Both	Liver cancer	2019	4.47	3.55	5.60
Guatemala	Both	Pancreatic cancer	2019	4.44	3.46	5.57
Guatemala	Both	Stomach cancer	2019	27.21	21.64	33.73
Guinea	Both	Colon and rectum cancer	2019	7.30	5.56	9.43
Guinea	Both	Esophageal cancer	2019	1.91	1.44	2.45
Guinea	Both	Liver cancer	2019	32.17	22.33	41.90
Guinea	Both	Pancreatic cancer	2019	1.80	1.36	2.26
Guinea	Both	Stomach cancer	2019	15.00	11.88	18.58
Guinea-Bissau	Both	Colon and rectum cancer	2019	9.32	7.08	11.68
Guinea-Bissau	Both	Esophageal cancer	2019	6.19	3.41	8.26

Guinea-Bissau	Both	Liver cancer	2019	6.12	4.45	8.28
Guinea-Bissau	Both	Pancreatic cancer	2019	4.53	3.09	6.10
Guinea-Bissau	Both	Stomach cancer	2019	18.04	14.30	22.22
Guyana	Both	Colon and rectum cancer	2019	18.71	14.85	23.58
Guyana	Both	Esophageal cancer	2019	2.09	1.61	2.69
Guyana	Both	Liver cancer	2019	3.03	2.36	3.84
Guyana	Both	Pancreatic cancer	2019	5.86	4.48	7.38
Guyana	Both	Stomach cancer	2019	8.60	6.81	10.71
Haiti	Both	Colon and rectum cancer	2019	11.45	7.97	15.43
Haiti	Both	Esophageal cancer	2019	3.52	2.37	5.19
Haiti	Both	Liver cancer	2019	4.14	2.49	6.26
Haiti	Both	Pancreatic cancer	2019	2.39	1.66	3.32
Haiti	Both	Stomach cancer	2019	14.99	9.68	20.05
Honduras	Both	Colon and rectum cancer	2019	9.67	6.74	13.58
Honduras	Both	Esophageal cancer	2019	1.66	1.17	2.16
Honduras	Both	Liver cancer	2019	14.80	6.80	21.70
Honduras	Both	Pancreatic cancer	2019	4.25	2.56	6.09
Honduras	Both	Stomach cancer	2019	15.47	12.69	19.60
Hungary	Both	Colon and rectum cancer	2019	52.24	42.96	62.90
Hungary	Both	Esophageal cancer	2019	3.73	2.97	4.62
Hungary	Both	Liver cancer	2019	2.58	2.11	3.16
Hungary	Both	Pancreatic cancer	2019	11.88	9.79	14.32
Hungary	Both	Stomach cancer	2019	9.60	7.95	11.49
Iceland	Both	Colon and rectum cancer	2019	30.71	26.82	35.36
Iceland	Both	Esophageal cancer	2019	4.87	4.14	5.73
Iceland	Both	Liver cancer	2019	3.52	3.06	4.05
Iceland	Both	Pancreatic cancer	2019	8.51	7.30	9.90
Iceland	Both	Stomach cancer	2019	7.23	6.28	8.31
India	Both	Colon and rectum cancer	2019	8.59	7.22	9.94
India	Both	Esophageal cancer	2019	3.36	2.78	4.89
India	Both	Liver cancer	2019	2.61	2.20	3.10
India	Both	Pancreatic cancer	2019	2.95	2.55	3.40
India	Both	Stomach cancer	2019	7.14	6.12	8.27
Indonesia	Both	Colon and rectum cancer	2019	18.53	12.63	23.42
Indonesia	Both	Esophageal cancer	2019	2.36	1.89	3.41
Indonesia	Both	Liver cancer	2019	2.23	1.90	2.51
Indonesia	Both	Pancreatic cancer	2019	5.04	3.30	6.97
Indonesia	Both	Stomach cancer	2019	6.40	5.36	7.28
Iran (Islamic Republic of)	Both	Colon and rectum cancer	2019	13.88	12.76	15.07
Iran (Islamic Republic of)	Both	Esophageal cancer	2019	4.36	3.43	4.80
Iran (Islamic Republic of)	Both	Liver cancer	2019	3.85	3.50	4.25
Iran (Islamic Republic of)	Both	Pancreatic cancer	2019	4.72	4.35	5.14
Iran (Islamic Republic of)	Both	Stomach cancer	2019	16.79	15.49	18.16
Iraq	Both	Colon and rectum cancer	2019	11.13	8.73	13.83
Iraq	Both	Esophageal cancer	2019	1.26	0.97	1.55
Iraq	Both	Liver cancer	2019	6.09	4.71	7.57
Iraq	Both	Pancreatic cancer	2019	5.91	4.48	7.26
Iraq	Both	Stomach cancer	2019	5.78	4.53	7.07
Ireland	Both	Colon and rectum cancer	2019	45.95	35.89	57.72
Ireland	Both	Esophageal cancer	2019	7.61	5.82	9.89
Ireland	Both	Liver cancer	2019	4.00	3.07	5.10
Ireland	Both	Pancreatic cancer	2019	9.24	7.15	11.71
Ireland	Both	Stomach cancer	2019	8.73	6.80	10.94
Israel	Both	Colon and rectum cancer	2019	33.64	26.30	42.59
Israel	Both	Esophageal cancer	2019	1.62	1.24	2.08
Israel	Both	Liver cancer	2019	2.97	2.31	3.79
Israel	Both	Pancreatic cancer	2019	10.57	8.22	13.44
Israel	Both	Stomach cancer	2019	7.71	6.04	9.80
Italy	Both	Colon and rectum cancer	2019	43.54	35.99	51.56
Italy	Both	Esophageal cancer	2019	2.05	1.67	2.48
Italy	Both	Liver cancer	2019	5.97	4.81	7.37
Italy	Both	Pancreatic cancer	2019	10.00	8.26	11.84
Italy	Both	Stomach cancer	2019	12.74	10.43	15.13
Jamaica	Both	Colon and rectum cancer	2019	25.95	20.82	32.26

Jamaica	Both	Esophageal cancer	2019	2.59	2.00	3.30
Jamaica	Both	Liver cancer	2019	2.61	2.07	3.25
Jamaica	Both	Pancreatic cancer	2019	4.95	3.83	6.21
Jamaica	Both	Stomach cancer	2019	9.76	7.75	12.14
Japan	Both	Colon and rectum cancer	2019	47.59	40.18	55.59
Japan	Both	Esophageal cancer	2019	6.45	5.29	7.79
Japan	Both	Liver cancer	2019	12.71	10.51	14.98
Japan	Both	Pancreatic cancer	2019	10.69	8.82	12.47
Japan	Both	Stomach cancer	2019	28.29	23.71	33.27
Jordan	Both	Colon and rectum cancer	2019	19.14	15.96	22.86
Jordan	Both	Esophageal cancer	2019	1.14	0.93	1.41
Jordan	Both	Liver cancer	2019	2.46	1.98	3.04
Jordan	Both	Pancreatic cancer	2019	5.19	4.34	6.18
Jordan	Both	Stomach cancer	2019	5.34	4.45	6.41
Kazakhstan	Both	Colon and rectum cancer	2019	19.72	17.18	22.45
Kazakhstan	Both	Esophageal cancer	2019	7.35	6.27	8.51
Kazakhstan	Both	Liver cancer	2019	6.30	5.43	7.24
Kazakhstan	Both	Pancreatic cancer	2019	6.53	5.58	7.57
Kazakhstan	Both	Stomach cancer	2019	14.63	12.77	16.69
Kenya	Both	Colon and rectum cancer	2019	8.24	6.68	10.05
Kenya	Both	Esophageal cancer	2019	11.93	8.96	16.74
Kenya	Both	Liver cancer	2019	2.90	1.92	4.25
Kenya	Both	Pancreatic cancer	2019	2.98	2.33	3.88
Kenya	Both	Stomach cancer	2019	8.65	7.04	10.33
Kiribati	Both	Colon and rectum cancer	2019	11.45	8.70	15.08
Kiribati	Both	Esophageal cancer	2019	7.66	3.30	9.94
Kiribati	Both	Liver cancer	2019	11.69	9.09	14.66
Kiribati	Both	Pancreatic cancer	2019	2.79	2.11	3.64
Kiribati	Both	Stomach cancer	2019	15.90	12.53	19.69
Kuwait	Both	Colon and rectum cancer	2019	17.78	14.65	21.32
Kuwait	Both	Esophageal cancer	2019	1.50	1.19	1.87
Kuwait	Both	Liver cancer	2019	2.75	2.17	3.42
Kuwait	Both	Pancreatic cancer	2019	5.33	4.29	6.55
Kuwait	Both	Stomach cancer	2019	3.94	3.26	4.76
Kyrgyzstan	Both	Colon and rectum cancer	2019	10.52	9.21	11.99
Kyrgyzstan	Both	Esophageal cancer	2019	4.13	3.51	4.82
Kyrgyzstan	Both	Liver cancer	2019	2.54	2.16	2.91
Kyrgyzstan	Both	Pancreatic cancer	2019	5.09	4.37	5.82
Kyrgyzstan	Both	Stomach cancer	2019	16.97	14.81	19.24
Lao People's Democratic Republic	Both	Colon and rectum cancer	2019	14.90	10.64	19.54
Lao People's Democratic Republic	Both	Esophageal cancer	2019	2.39	1.66	3.50
Lao People's Democratic Republic	Both	Liver cancer	2019	6.72	5.06	8.54
Lao People's Democratic Republic	Both	Pancreatic cancer	2019	3.31	2.59	4.14
Lao People's Democratic Republic	Both	Stomach cancer	2019	7.77	5.95	9.66
Latvia	Both	Colon and rectum cancer	2019	30.54	25.44	36.96
Latvia	Both	Esophageal cancer	2019	3.30	2.54	4.28
Latvia	Both	Liver cancer	2019	2.68	2.22	3.26
Latvia	Both	Pancreatic cancer	2019	9.86	8.12	12.05
Latvia	Both	Stomach cancer	2019	14.05	11.76	16.93
Lebanon	Both	Colon and rectum cancer	2019	29.83	24.07	38.13
Lebanon	Both	Esophageal cancer	2019	1.30	0.97	1.72
Lebanon	Both	Liver cancer	2019	3.67	2.74	5.09
Lebanon	Both	Pancreatic cancer	2019	6.55	5.06	8.03
Lebanon	Both	Stomach cancer	2019	8.07	6.53	10.55
Lesotho	Both	Colon and rectum cancer	2019	12.01	8.83	15.54
Lesotho	Both	Esophageal cancer	2019	14.59	9.17	19.34
Lesotho	Both	Liver cancer	2019	14.44	6.05	22.70
Lesotho	Both	Pancreatic cancer	2019	5.67	3.96	7.60
Lesotho	Both	Stomach cancer	2019	11.52	8.72	14.67
Liberia	Both	Colon and rectum cancer	2019	6.84	4.66	9.71
Liberia	Both	Esophageal cancer	2019	4.88	2.69	6.74
Liberia	Both	Liver cancer	2019	4.92	3.60	7.24
Liberia	Both	Pancreatic cancer	2019	4.72	3.44	6.22
Liberia	Both	Stomach cancer	2019	11.13	8.52	14.25

Libya	Both	Colon and rectum cancer	2019	17.00	12.41	21.83
Libya	Both	Esophageal cancer	2019	1.37	0.97	1.75
Libya	Both	Liver cancer	2019	5.18	3.88	7.05
Libya	Both	Pancreatic cancer	2019	7.03	5.41	9.04
Libya	Both	Stomach cancer	2019	5.70	4.39	7.23
Lithuania	Both	Colon and rectum cancer	2019	29.20	24.01	35.37
Lithuania	Both	Esophageal cancer	2019	3.79	2.99	4.75
Lithuania	Both	Liver cancer	2019	3.20	2.50	3.95
Lithuania	Both	Pancreatic cancer	2019	9.01	7.29	11.11
Lithuania	Both	Stomach cancer	2019	13.93	11.27	17.01
Luxembourg	Both	Colon and rectum cancer	2019	37.17	30.27	45.07
Luxembourg	Both	Esophageal cancer	2019	3.84	3.07	4.86
Luxembourg	Both	Liver cancer	2019	4.03	3.14	5.25
Luxembourg	Both	Pancreatic cancer	2019	8.79	7.16	10.64
Luxembourg	Both	Stomach cancer	2019	6.68	5.45	8.07
Madagascar	Both	Colon and rectum cancer	2019	7.33	5.41	9.73
Madagascar	Both	Esophageal cancer	2019	9.74	6.18	13.64
Madagascar	Both	Liver cancer	2019	2.44	1.66	3.72
Madagascar	Both	Pancreatic cancer	2019	2.28	1.62	3.03
Madagascar	Both	Stomach cancer	2019	6.58	5.03	8.46
Malawi	Both	Colon and rectum cancer	2019	6.27	4.87	7.80
Malawi	Both	Esophageal cancer	2019	24.53	18.74	32.51
Malawi	Both	Liver cancer	2019	2.82	2.23	3.54
Malawi	Both	Pancreatic cancer	2019	2.90	2.24	3.63
Malawi	Both	Stomach cancer	2019	3.28	2.67	3.91
Malaysia	Both	Colon and rectum cancer	2019	29.57	23.27	36.50
Malaysia	Both	Esophageal cancer	2019	2.66	2.06	3.34
Malaysia	Both	Liver cancer	2019	6.08	4.65	7.83
Malaysia	Both	Pancreatic cancer	2019	3.61	2.81	4.53
Malaysia	Both	Stomach cancer	2019	7.24	5.86	8.96
Maldives	Both	Colon and rectum cancer	2019	13.80	11.28	16.40
Maldives	Both	Esophageal cancer	2019	2.05	1.65	2.48
Maldives	Both	Liver cancer	2019	5.47	4.31	6.70
Maldives	Both	Pancreatic cancer	2019	4.24	3.49	5.08
Maldives	Both	Stomach cancer	2019	3.79	3.11	4.52
Mali	Both	Colon and rectum cancer	2019	8.11	6.43	10.13
Mali	Both	Esophageal cancer	2019	2.55	1.95	3.37
Mali	Both	Liver cancer	2019	14.46	10.75	18.98
Mali	Both	Pancreatic cancer	2019	3.25	2.47	4.20
Mali	Both	Stomach cancer	2019	17.12	13.74	21.49
Malta	Both	Colon and rectum cancer	2019	32.92	27.83	39.12
Malta	Both	Esophageal cancer	2019	2.50	2.04	3.06
Malta	Both	Liver cancer	2019	2.50	2.11	2.95
Malta	Both	Pancreatic cancer	2019	8.89	7.32	10.63
Malta	Both	Stomach cancer	2019	6.79	5.71	7.93
Marshall Islands	Both	Colon and rectum cancer	2019	13.71	10.51	17.43
Marshall Islands	Both	Esophageal cancer	2019	2.68	1.86	3.73
Marshall Islands	Both	Liver cancer	2019	10.22	7.38	13.84
Marshall Islands	Both	Pancreatic cancer	2019	3.81	2.83	4.97
Marshall Islands	Both	Stomach cancer	2019	16.25	12.50	20.54
Mauritania	Both	Colon and rectum cancer	2019	8.84	6.80	11.08
Mauritania	Both	Esophageal cancer	2019	4.14	2.44	5.53
Mauritania	Both	Liver cancer	2019	4.10	3.03	5.29
Mauritania	Both	Pancreatic cancer	2019	6.02	4.39	7.83
Mauritania	Both	Stomach cancer	2019	10.16	7.86	12.86
Mauritius	Both	Colon and rectum cancer	2019	19.77	16.12	24.16
Mauritius	Both	Esophageal cancer	2019	2.53	2.03	3.18
Mauritius	Both	Liver cancer	2019	1.93	1.49	2.49
Mauritius	Both	Pancreatic cancer	2019	5.18	4.16	6.33
Mauritius	Both	Stomach cancer	2019	7.56	6.15	9.19
Mexico	Both	Colon and rectum cancer	2019	14.92	12.88	17.11
Mexico	Both	Esophageal cancer	2019	1.44	1.23	1.69
Mexico	Both	Liver cancer	2019	3.46	2.99	3.96
Mexico	Both	Pancreatic cancer	2019	5.78	4.97	6.64

Mexico	Both	Stomach cancer	2019	9.70	8.43	11.19
Micronesia (Federated States of)	Both	Colon and rectum cancer	2019	15.36	10.93	20.00
Micronesia (Federated States of)	Both	Esophageal cancer	2019	3.04	2.10	4.17
Micronesia (Federated States of)	Both	Liver cancer	2019	10.37	6.93	14.51
Micronesia (Federated States of)	Both	Pancreatic cancer	2019	5.06	3.54	6.81
Micronesia (Federated States of)	Both	Stomach cancer	2019	16.85	12.61	21.13
Monaco	Both	Colon and rectum cancer	2019	60.69	48.55	73.57
Monaco	Both	Esophageal cancer	2019	6.55	4.95	8.06
Monaco	Both	Liver cancer	2019	9.04	7.13	11.28
Monaco	Both	Pancreatic cancer	2019	18.14	14.37	21.72
Monaco	Both	Stomach cancer	2019	9.91	7.82	12.01
Mongolia	Both	Colon and rectum cancer	2019	11.09	8.77	14.12
Mongolia	Both	Esophageal cancer	2019	21.93	14.53	27.95
Mongolia	Both	Liver cancer	2019	105.22	82.57	131.46
Mongolia	Both	Pancreatic cancer	2019	5.97	4.70	7.51
Mongolia	Both	Stomach cancer	2019	43.70	34.29	55.10
Montenegro	Both	Colon and rectum cancer	2019	30.47	24.96	36.76
Montenegro	Both	Esophageal cancer	2019	2.40	1.90	2.98
Montenegro	Both	Liver cancer	2019	5.97	4.82	7.31
Montenegro	Both	Pancreatic cancer	2019	10.75	9.05	12.74
Montenegro	Both	Stomach cancer	2019	8.24	6.88	9.78
Morocco	Both	Colon and rectum cancer	2019	10.34	7.70	13.02
Morocco	Both	Esophageal cancer	2019	1.57	1.18	1.90
Morocco	Both	Liver cancer	2019	2.16	1.65	2.63
Morocco	Both	Pancreatic cancer	2019	3.77	2.69	4.90
Morocco	Both	Stomach cancer	2019	4.60	3.54	5.47
Mozambique	Both	Colon and rectum cancer	2019	8.73	6.43	11.37
Mozambique	Both	Esophageal cancer	2019	7.80	5.76	10.31
Mozambique	Both	Liver cancer	2019	3.67	2.55	4.85
Mozambique	Both	Pancreatic cancer	2019	3.61	2.53	5.07
Mozambique	Both	Stomach cancer	2019	6.93	5.51	8.66
Myanmar	Both	Colon and rectum cancer	2019	15.03	11.10	19.26
Myanmar	Both	Esophageal cancer	2019	2.25	1.79	3.39
Myanmar	Both	Liver cancer	2019	4.11	3.46	4.88
Myanmar	Both	Pancreatic cancer	2019	3.71	2.91	4.79
Myanmar	Both	Stomach cancer	2019	6.86	5.75	8.30
Namibia	Both	Colon and rectum cancer	2019	9.74	7.69	12.25
Namibia	Both	Esophageal cancer	2019	2.63	2.03	3.36
Namibia	Both	Liver cancer	2019	3.28	2.50	4.20
Namibia	Both	Pancreatic cancer	2019	3.83	2.99	4.89
Namibia	Both	Stomach cancer	2019	3.48	2.83	4.28
Nauru	Both	Colon and rectum cancer	2019	21.43	14.78	27.76
Nauru	Both	Esophageal cancer	2019	3.14	2.19	4.16
Nauru	Both	Liver cancer	2019	9.17	6.50	12.59
Nauru	Both	Pancreatic cancer	2019	5.84	4.04	7.89
Nauru	Both	Stomach cancer	2019	17.90	14.57	21.72
Nepal	Both	Colon and rectum cancer	2019	5.88	4.22	8.15
Nepal	Both	Esophageal cancer	2019	4.78	3.55	6.48
Nepal	Both	Liver cancer	2019	2.13	1.54	3.05
Nepal	Both	Pancreatic cancer	2019	2.76	1.65	4.08
Nepal	Both	Stomach cancer	2019	8.42	6.58	10.66
Netherlands	Both	Colon and rectum cancer	2019	55.39	43.29	69.76
Netherlands	Both	Esophageal cancer	2019	8.37	6.47	10.58
Netherlands	Both	Liver cancer	2019	3.33	2.58	4.19
Netherlands	Both	Pancreatic cancer	2019	11.29	8.83	14.11
Netherlands	Both	Stomach cancer	2019	10.23	8.04	12.75
New Zealand	Both	Colon and rectum cancer	2019	55.34	45.72	65.79
New Zealand	Both	Esophageal cancer	2019	4.79	3.86	5.79
New Zealand	Both	Liver cancer	2019	4.96	4.09	6.02
New Zealand	Both	Pancreatic cancer	2019	8.18	6.80	9.75
New Zealand	Both	Stomach cancer	2019	6.96	5.75	8.31
Nicaragua	Both	Colon and rectum cancer	2019	16.17	13.44	19.03
Nicaragua	Both	Esophageal cancer	2019	1.20	0.94	1.55
Nicaragua	Both	Liver cancer	2019	3.79	3.09	4.63

Nicaragua	Both	Pancreatic cancer	2019	5.49	4.54	6.48
Nicaragua	Both	Stomach cancer	2019	15.77	12.99	18.74
Niger	Both	Colon and rectum cancer	2019	5.63	4.19	7.56
Niger	Both	Esophageal cancer	2019	3.86	2.24	5.22
Niger	Both	Liver cancer	2019	0.61	0.45	0.80
Niger	Both	Pancreatic cancer	2019	2.19	1.46	3.10
Niger	Both	Stomach cancer	2019	13.26	10.13	16.66
Nigeria	Both	Colon and rectum cancer	2019	8.95	6.87	11.02
Nigeria	Both	Esophageal cancer	2019	0.91	0.65	1.58
Nigeria	Both	Liver cancer	2019	3.25	2.56	4.04
Nigeria	Both	Pancreatic cancer	2019	3.92	3.03	4.84
Nigeria	Both	Stomach cancer	2019	3.91	3.20	4.77
Niue	Both	Colon and rectum cancer	2019	20.40	15.71	26.06
Niue	Both	Esophageal cancer	2019	2.46	1.88	3.01
Niue	Both	Liver cancer	2019	7.37	5.65	9.46
Niue	Both	Pancreatic cancer	2019	6.18	4.67	7.91
Niue	Both	Stomach cancer	2019	11.01	9.04	13.30
North Macedonia	Both	Colon and rectum cancer	2019	35.36	28.19	43.94
North Macedonia	Both	Esophageal cancer	2019	1.52	1.19	1.93
North Macedonia	Both	Liver cancer	2019	8.39	6.65	10.65
North Macedonia	Both	Pancreatic cancer	2019	10.52	8.38	13.13
North Macedonia	Both	Stomach cancer	2019	16.15	12.88	20.23
Northern Mariana Islands	Both	Colon and rectum cancer	2019	28.86	24.45	33.06
Northern Mariana Islands	Both	Esophageal cancer	2019	2.95	2.10	3.52
Northern Mariana Islands	Both	Liver cancer	2019	7.97	6.58	9.70
Northern Mariana Islands	Both	Pancreatic cancer	2019	7.49	6.40	8.69
Northern Mariana Islands	Both	Stomach cancer	2019	13.01	11.03	15.13
Norway	Both	Colon and rectum cancer	2019	49.46	41.90	57.73
Norway	Both	Esophageal cancer	2019	2.85	2.37	3.40
Norway	Both	Liver cancer	2019	2.91	2.42	3.51
Norway	Both	Pancreatic cancer	2019	9.43	8.04	10.99
Norway	Both	Stomach cancer	2019	6.67	5.65	7.82
Oman	Both	Colon and rectum cancer	2019	15.32	12.63	18.56
Oman	Both	Esophageal cancer	2019	2.37	1.84	2.79
Oman	Both	Liver cancer	2019	5.27	4.47	6.30
Oman	Both	Pancreatic cancer	2019	5.99	5.22	6.90
Oman	Both	Stomach cancer	2019	8.55	7.51	9.82
Pakistan	Both	Colon and rectum cancer	2019	9.14	7.33	11.63
Pakistan	Both	Esophageal cancer	2019	7.86	6.30	9.63
Pakistan	Both	Liver cancer	2019	3.27	2.56	4.02
Pakistan	Both	Pancreatic cancer	2019	2.91	2.27	3.76
Pakistan	Both	Stomach cancer	2019	6.45	5.34	7.79
Palau	Both	Colon and rectum cancer	2019	19.94	15.70	24.69
Palau	Both	Esophageal cancer	2019	2.45	1.95	3.08
Palau	Both	Liver cancer	2019	9.97	7.68	12.94
Palau	Both	Pancreatic cancer	2019	11.43	8.81	14.37
Palau	Both	Stomach cancer	2019	12.25	9.69	15.32
Palestine	Both	Colon and rectum cancer	2019	26.12	22.15	30.27
Palestine	Both	Esophageal cancer	2019	1.10	0.90	1.49
Palestine	Both	Liver cancer	2019	6.56	5.52	7.88
Palestine	Both	Pancreatic cancer	2019	6.70	5.59	7.95
Palestine	Both	Stomach cancer	2019	7.16	6.13	8.27
Panama	Both	Colon and rectum cancer	2019	18.61	14.45	23.57
Panama	Both	Esophageal cancer	2019	1.60	1.19	2.07
Panama	Both	Liver cancer	2019	2.93	2.23	3.77
Panama	Both	Pancreatic cancer	2019	4.68	3.59	6.02
Panama	Both	Stomach cancer	2019	12.47	9.70	15.94
Papua New Guinea	Both	Colon and rectum cancer	2019	8.06	5.88	10.54
Papua New Guinea	Both	Esophageal cancer	2019	1.88	1.36	2.81
Papua New Guinea	Both	Liver cancer	2019	1.53	1.19	1.96
Papua New Guinea	Both	Pancreatic cancer	2019	1.72	1.21	2.44
Papua New Guinea	Both	Stomach cancer	2019	13.26	9.68	17.25
Paraguay	Both	Colon and rectum cancer	2019	17.37	13.38	22.12
Paraguay	Both	Esophageal cancer	2019	3.83	2.84	5.03

Paraguay	Both	Liver cancer	2019	2.05	1.54	2.67
Paraguay	Both	Pancreatic cancer	2019	5.96	4.53	7.51
Paraguay	Both	Stomach cancer	2019	8.91	6.85	11.42
Peru	Both	Colon and rectum cancer	2019	21.24	15.91	27.77
Peru	Both	Esophageal cancer	2019	1.29	0.96	1.70
Peru	Both	Liver cancer	2019	2.60	1.93	3.45
Peru	Both	Pancreatic cancer	2019	5.19	3.90	6.81
Peru	Both	Stomach cancer	2019	19.56	14.67	25.59
Philippines	Both	Colon and rectum cancer	2019	18.93	15.42	23.14
Philippines	Both	Esophageal cancer	2019	1.32	1.06	1.75
Philippines	Both	Liver cancer	2019	6.38	5.16	7.84
Philippines	Both	Pancreatic cancer	2019	4.19	3.39	5.19
Philippines	Both	Stomach cancer	2019	4.38	3.62	5.27
Poland	Both	Colon and rectum cancer	2019	34.96	29.84	41.55
Poland	Both	Esophageal cancer	2019	3.19	2.60	3.91
Poland	Both	Liver cancer	2019	1.95	1.63	2.31
Poland	Both	Pancreatic cancer	2019	9.94	8.41	11.70
Poland	Both	Stomach cancer	2019	9.33	7.89	11.08
Portugal	Both	Colon and rectum cancer	2019	45.29	35.23	57.72
Portugal	Both	Esophageal cancer	2019	3.47	2.61	4.55
Portugal	Both	Liver cancer	2019	4.90	3.79	6.26
Portugal	Both	Pancreatic cancer	2019	7.02	5.46	8.99
Portugal	Both	Stomach cancer	2019	14.64	11.41	18.59
Puerto Rico	Both	Colon and rectum cancer	2019	34.99	26.99	44.78
Puerto Rico	Both	Esophageal cancer	2019	2.56	1.96	3.31
Puerto Rico	Both	Liver cancer	2019	2.76	2.06	3.59
Puerto Rico	Both	Pancreatic cancer	2019	6.00	4.58	7.67
Puerto Rico	Both	Stomach cancer	2019	5.83	4.55	7.41
Qatar	Both	Colon and rectum cancer	2019	25.05	19.33	31.85
Qatar	Both	Esophageal cancer	2019	4.47	3.16	6.14
Qatar	Both	Liver cancer	2019	17.39	12.83	22.91
Qatar	Both	Pancreatic cancer	2019	7.62	5.76	10.04
Qatar	Both	Stomach cancer	2019	8.99	6.95	11.39
Republic of Korea	Both	Colon and rectum cancer	2019	37.16	31.14	44.01
Republic of Korea	Both	Esophageal cancer	2019	3.76	2.96	5.16
Republic of Korea	Both	Liver cancer	2019	22.80	18.72	27.32
Republic of Korea	Both	Pancreatic cancer	2019	8.98	7.51	10.71
Republic of Korea	Both	Stomach cancer	2019	28.67	23.65	34.17
Republic of Moldova	Both	Colon and rectum cancer	2019	29.17	25.39	33.14
Republic of Moldova	Both	Esophageal cancer	2019	1.79	1.51	2.13
Republic of Moldova	Both	Liver cancer	2019	2.30	1.95	2.70
Republic of Moldova	Both	Pancreatic cancer	2019	7.94	6.88	9.14
Republic of Moldova	Both	Stomach cancer	2019	10.40	8.97	11.94
Romania	Both	Colon and rectum cancer	2019	36.16	29.52	43.30
Romania	Both	Esophageal cancer	2019	2.59	2.07	3.15
Romania	Both	Liver cancer	2019	3.02	2.45	3.70
Romania	Both	Pancreatic cancer	2019	9.86	8.06	12.11
Romania	Both	Stomach cancer	2019	11.74	9.53	14.35
Russian Federation	Both	Colon and rectum cancer	2019	30.77	27.02	35.11
Russian Federation	Both	Esophageal cancer	2019	3.38	2.86	3.98
Russian Federation	Both	Liver cancer	2019	2.91	2.46	3.44
Russian Federation	Both	Pancreatic cancer	2019	7.88	6.94	8.97
Russian Federation	Both	Stomach cancer	2019	16.08	14.09	18.39
Rwanda	Both	Colon and rectum cancer	2019	9.32	7.43	11.66
Rwanda	Both	Esophageal cancer	2019	10.80	7.10	15.23
Rwanda	Both	Liver cancer	2019	4.40	3.48	5.65
Rwanda	Both	Pancreatic cancer	2019	3.37	2.68	4.29
Rwanda	Both	Stomach cancer	2019	6.87	5.41	8.46
Saint Kitts and Nevis	Both	Colon and rectum cancer	2019	31.11	26.32	36.41
Saint Kitts and Nevis	Both	Esophageal cancer	2019	4.33	3.60	5.14
Saint Kitts and Nevis	Both	Liver cancer	2019	3.73	3.12	4.44
Saint Kitts and Nevis	Both	Pancreatic cancer	2019	9.08	7.57	10.82
Saint Kitts and Nevis	Both	Stomach cancer	2019	10.69	8.99	12.49
Saint Lucia	Both	Colon and rectum cancer	2019	17.96	15.15	21.20

Saint Lucia	Both	Esophageal cancer	2019	4.32	3.58	5.15
Saint Lucia	Both	Liver cancer	2019	2.16	1.80	2.59
Saint Lucia	Both	Pancreatic cancer	2019	7.35	6.16	8.68
Saint Lucia	Both	Stomach cancer	2019	12.17	10.26	14.21
Saint Vincent and the Grenadines	Both	Colon and rectum cancer	2019	19.66	17.20	22.56
Saint Vincent and the Grenadines	Both	Esophageal cancer	2019	2.38	2.04	2.77
Saint Vincent and the Grenadines	Both	Liver cancer	2019	2.97	2.57	3.44
Saint Vincent and the Grenadines	Both	Pancreatic cancer	2019	6.16	5.34	7.07
Saint Vincent and the Grenadines	Both	Stomach cancer	2019	11.18	9.75	12.91
Samoa	Both	Colon and rectum cancer	2019	12.76	10.27	15.68
Samoa	Both	Esophageal cancer	2019	1.61	1.22	2.01
Samoa	Both	Liver cancer	2019	4.99	3.82	6.29
Samoa	Both	Pancreatic cancer	2019	4.22	3.40	5.41
Samoa	Both	Stomach cancer	2019	12.22	9.78	15.23
San Marino	Both	Colon and rectum cancer	2019	49.55	37.89	65.53
San Marino	Both	Esophageal cancer	2019	1.71	1.28	2.24
San Marino	Both	Liver cancer	2019	3.22	2.45	4.29
San Marino	Both	Pancreatic cancer	2019	10.90	8.29	14.47
San Marino	Both	Stomach cancer	2019	26.16	19.98	33.99
Sao Tome and Principe	Both	Colon and rectum cancer	2019	16.46	12.02	22.22
Sao Tome and Principe	Both	Esophageal cancer	2019	4.81	2.63	6.03
Sao Tome and Principe	Both	Liver cancer	2019	2.64	1.77	3.48
Sao Tome and Principe	Both	Pancreatic cancer	2019	2.96	1.98	4.11
Sao Tome and Principe	Both	Stomach cancer	2019	16.58	13.49	20.66
Saudi Arabia	Both	Colon and rectum cancer	2019	15.36	12.16	18.83
Saudi Arabia	Both	Esophageal cancer	2019	1.78	1.38	2.36
Saudi Arabia	Both	Liver cancer	2019	5.29	4.17	6.69
Saudi Arabia	Both	Pancreatic cancer	2019	5.02	4.06	6.14
Saudi Arabia	Both	Stomach cancer	2019	4.40	3.60	5.33
Senegal	Both	Colon and rectum cancer	2019	8.92	7.17	11.08
Senegal	Both	Esophageal cancer	2019	4.68	2.60	6.21
Senegal	Both	Liver cancer	2019	2.07	1.55	2.60
Senegal	Both	Pancreatic cancer	2019	4.53	3.69	5.58
Senegal	Both	Stomach cancer	2019	12.66	9.98	15.52
Serbia	Both	Colon and rectum cancer	2019	43.55	35.07	54.26
Serbia	Both	Esophageal cancer	2019	2.43	1.88	3.12
Serbia	Both	Liver cancer	2019	5.42	4.28	6.82
Serbia	Both	Pancreatic cancer	2019	10.21	8.06	12.76
Serbia	Both	Stomach cancer	2019	9.69	7.71	12.09
Seychelles	Both	Colon and rectum cancer	2019	35.70	31.42	40.58
Seychelles	Both	Esophageal cancer	2019	5.63	4.76	6.67
Seychelles	Both	Liver cancer	2019	5.56	4.68	6.58
Seychelles	Both	Pancreatic cancer	2019	7.17	6.26	8.25
Seychelles	Both	Stomach cancer	2019	7.15	6.16	8.21
Sierra Leone	Both	Colon and rectum cancer	2019	7.09	5.48	9.11
Sierra Leone	Both	Esophageal cancer	2019	4.32	2.35	5.83
Sierra Leone	Both	Liver cancer	2019	4.62	3.50	6.03
Sierra Leone	Both	Pancreatic cancer	2019	3.49	2.75	4.44
Sierra Leone	Both	Stomach cancer	2019	12.44	9.67	15.78
Singapore	Both	Colon and rectum cancer	2019	39.93	31.93	49.46
Singapore	Both	Esophageal cancer	2019	2.71	2.12	3.45
Singapore	Both	Liver cancer	2019	11.50	9.24	14.41
Singapore	Both	Pancreatic cancer	2019	6.07	4.86	7.53
Singapore	Both	Stomach cancer	2019	10.72	8.60	13.31
Slovakia	Both	Colon and rectum cancer	2019	56.45	44.36	71.04
Slovakia	Both	Esophageal cancer	2019	3.48	2.49	4.62
Slovakia	Both	Liver cancer	2019	3.41	2.66	4.33
Slovakia	Both	Pancreatic cancer	2019	11.51	9.00	14.39
Slovakia	Both	Stomach cancer	2019	12.19	9.58	15.17
Slovenia	Both	Colon and rectum cancer	2019	41.05	31.88	52.95
Slovenia	Both	Esophageal cancer	2019	2.85	2.15	3.76
Slovenia	Both	Liver cancer	2019	5.30	4.04	6.83
Slovenia	Both	Pancreatic cancer	2019	9.51	7.42	12.41
Slovenia	Both	Stomach cancer	2019	11.94	9.34	15.62

Solomon Islands	Both	Colon and rectum cancer	2019	12.36	8.44	16.09
Solomon Islands	Both	Esophageal cancer	2019	3.34	2.41	4.63
Solomon Islands	Both	Liver cancer	2019	5.37	4.30	6.55
Solomon Islands	Both	Pancreatic cancer	2019	2.97	2.06	3.94
Solomon Islands	Both	Stomach cancer	2019	23.66	18.58	28.89
Somalia	Both	Colon and rectum cancer	2019	4.95	3.13	9.24
Somalia	Both	Esophageal cancer	2019	12.37	7.94	17.53
Somalia	Both	Liver cancer	2019	3.33	2.15	5.91
Somalia	Both	Pancreatic cancer	2019	1.58	0.85	2.55
Somalia	Both	Stomach cancer	2019	9.93	7.35	13.21
South Africa	Both	Colon and rectum cancer	2019	12.88	11.62	14.50
South Africa	Both	Esophageal cancer	2019	9.91	8.78	12.09
South Africa	Both	Liver cancer	2019	5.60	4.94	6.35
South Africa	Both	Pancreatic cancer	2019	5.89	5.26	6.60
South Africa	Both	Stomach cancer	2019	5.39	5.00	5.83
South Sudan	Both	Colon and rectum cancer	2019	9.92	6.56	14.66
South Sudan	Both	Esophageal cancer	2019	10.07	6.70	14.51
South Sudan	Both	Liver cancer	2019	2.74	1.64	4.77
South Sudan	Both	Pancreatic cancer	2019	2.78	2.02	3.87
South Sudan	Both	Stomach cancer	2019	6.72	4.92	9.10
Spain	Both	Colon and rectum cancer	2019	50.14	39.34	63.84
Spain	Both	Esophageal cancer	2019	3.03	2.31	3.90
Spain	Both	Liver cancer	2019	6.01	4.63	7.72
Spain	Both	Pancreatic cancer	2019	8.36	6.49	10.47
Spain	Both	Stomach cancer	2019	11.46	8.95	14.37
Sri Lanka	Both	Colon and rectum cancer	2019	10.18	7.63	13.19
Sri Lanka	Both	Esophageal cancer	2019	4.61	3.40	6.19
Sri Lanka	Both	Liver cancer	2019	2.80	2.06	3.72
Sri Lanka	Both	Pancreatic cancer	2019	2.86	2.14	3.77
Sri Lanka	Both	Stomach cancer	2019	5.43	4.12	7.13
Sudan	Both	Colon and rectum cancer	2019	8.22	5.99	12.28
Sudan	Both	Esophageal cancer	2019	4.74	1.48	6.85
Sudan	Both	Liver cancer	2019	3.70	2.19	5.76
Sudan	Both	Pancreatic cancer	2019	3.59	2.44	5.52
Sudan	Both	Stomach cancer	2019	14.95	10.27	19.41
Suriname	Both	Colon and rectum cancer	2019	21.61	17.85	25.57
Suriname	Both	Esophageal cancer	2019	1.38	1.10	1.68
Suriname	Both	Liver cancer	2019	2.60	2.09	3.23
Suriname	Both	Pancreatic cancer	2019	6.95	5.74	8.32
Suriname	Both	Stomach cancer	2019	7.12	5.94	8.54
Sweden	Both	Colon and rectum cancer	2019	36.99	31.41	42.78
Sweden	Both	Esophageal cancer	2019	2.95	2.45	3.52
Sweden	Both	Liver cancer	2019	3.04	2.56	3.59
Sweden	Both	Pancreatic cancer	2019	8.40	7.06	9.72
Sweden	Both	Stomach cancer	2019	5.01	4.20	5.86
Switzerland	Both	Colon and rectum cancer	2019	33.59	25.91	42.71
Switzerland	Both	Esophageal cancer	2019	4.09	3.13	5.31
Switzerland	Both	Liver cancer	2019	5.79	4.44	7.59
Switzerland	Both	Pancreatic cancer	2019	9.39	7.28	11.91
Switzerland	Both	Stomach cancer	2019	6.64	5.18	8.42
Syrian Arab Republic	Both	Colon and rectum cancer	2019	8.54	6.26	11.28
Syrian Arab Republic	Both	Esophageal cancer	2019	0.92	0.69	1.19
Syrian Arab Republic	Both	Liver cancer	2019	4.50	3.42	5.90
Syrian Arab Republic	Both	Pancreatic cancer	2019	3.62	2.71	4.85
Syrian Arab Republic	Both	Stomach cancer	2019	4.97	3.78	6.55
Taiwan (Province of China)	Both	Colon and rectum cancer	2019	62.05	48.91	80.05
Taiwan (Province of China)	Both	Esophageal cancer	2019	9.99	7.58	13.27
Taiwan (Province of China)	Both	Liver cancer	2019	8.65	6.73	11.15
Taiwan (Province of China)	Both	Pancreatic cancer	2019	8.54	6.64	11.11
Taiwan (Province of China)	Both	Stomach cancer	2019	15.60	12.23	20.26
Tajikistan	Both	Colon and rectum cancer	2019	11.62	9.50	14.05
Tajikistan	Both	Esophageal cancer	2019	7.16	5.26	15.41
Tajikistan	Both	Liver cancer	2019	3.70	2.96	4.66
Tajikistan	Both	Pancreatic cancer	2019	5.18	4.31	6.35

Tajikistan	Both	Stomach cancer	2019	24.01	19.72	29.11
Thailand	Both	Colon and rectum cancer	2019	17.18	12.71	22.51
Thailand	Both	Esophageal cancer	2019	3.13	2.01	4.22
Thailand	Both	Liver cancer	2019	24.18	17.89	32.01
Thailand	Both	Pancreatic cancer	2019	3.95	2.96	5.09
Thailand	Both	Stomach cancer	2019	5.90	4.44	7.81
Timor-Leste	Both	Colon and rectum cancer	2019	13.91	9.69	17.85
Timor-Leste	Both	Esophageal cancer	2019	2.32	1.69	3.40
Timor-Leste	Both	Liver cancer	2019	6.19	4.18	8.57
Timor-Leste	Both	Pancreatic cancer	2019	2.86	2.05	3.69
Timor-Leste	Both	Stomach cancer	2019	7.30	5.36	8.99
Togo	Both	Colon and rectum cancer	2019	8.22	6.14	10.53
Togo	Both	Esophageal cancer	2019	4.66	2.56	6.32
Togo	Both	Liver cancer	2019	4.95	3.83	6.37
Togo	Both	Pancreatic cancer	2019	4.62	3.40	6.19
Togo	Both	Stomach cancer	2019	13.02	10.51	16.39
Tokelau	Both	Colon and rectum cancer	2019	14.11	10.54	18.35
Tokelau	Both	Esophageal cancer	2019	1.90	1.44	2.44
Tokelau	Both	Liver cancer	2019	7.32	5.29	9.90
Tokelau	Both	Pancreatic cancer	2019	4.26	3.00	5.51
Tokelau	Both	Stomach cancer	2019	9.68	7.70	12.16
Tonga	Both	Colon and rectum cancer	2019	8.28	6.45	10.46
Tonga	Both	Esophageal cancer	2019	2.07	1.57	2.67
Tonga	Both	Liver cancer	2019	24.33	17.65	31.90
Tonga	Both	Pancreatic cancer	2019	4.26	3.12	5.61
Tonga	Both	Stomach cancer	2019	13.34	10.91	16.05
Trinidad and Tobago	Both	Colon and rectum cancer	2019	21.07	16.12	27.06
Trinidad and Tobago	Both	Esophageal cancer	2019	1.53	1.12	2.01
Trinidad and Tobago	Both	Liver cancer	2019	2.51	1.90	3.29
Trinidad and Tobago	Both	Pancreatic cancer	2019	5.67	4.29	7.39
Trinidad and Tobago	Both	Stomach cancer	2019	4.49	3.41	5.78
Tunisia	Both	Colon and rectum cancer	2019	14.49	10.54	19.50
Tunisia	Both	Esophageal cancer	2019	0.96	0.67	1.30
Tunisia	Both	Liver cancer	2019	1.94	1.38	2.69
Tunisia	Both	Pancreatic cancer	2019	3.76	2.81	4.97
Tunisia	Both	Stomach cancer	2019	5.57	4.15	7.48
Turkey	Both	Colon and rectum cancer	2019	20.56	16.42	25.01
Turkey	Both	Esophageal cancer	2019	1.56	1.21	1.96
Turkey	Both	Liver cancer	2019	3.21	2.55	3.95
Turkey	Both	Pancreatic cancer	2019	8.08	6.47	9.93
Turkey	Both	Stomach cancer	2019	11.91	9.51	14.60
Turkmenistan	Both	Colon and rectum cancer	2019	9.19	7.47	11.33
Turkmenistan	Both	Esophageal cancer	2019	9.58	7.60	11.95
Turkmenistan	Both	Liver cancer	2019	5.55	4.38	7.01
Turkmenistan	Both	Pancreatic cancer	2019	3.72	2.99	4.64
Turkmenistan	Both	Stomach cancer	2019	9.35	7.44	11.66
Tuvalu	Both	Colon and rectum cancer	2019	13.03	9.60	16.87
Tuvalu	Both	Esophageal cancer	2019	2.30	1.68	3.10
Tuvalu	Both	Liver cancer	2019	8.47	6.24	11.32
Tuvalu	Both	Pancreatic cancer	2019	3.83	2.83	5.13
Tuvalu	Both	Stomach cancer	2019	13.25	10.32	17.00
Uganda	Both	Colon and rectum cancer	2019	12.31	9.77	14.83
Uganda	Both	Esophageal cancer	2019	15.61	12.06	19.47
Uganda	Both	Liver cancer	2019	6.06	4.81	7.51
Uganda	Both	Pancreatic cancer	2019	4.71	3.68	5.86
Uganda	Both	Stomach cancer	2019	8.16	6.59	9.84
Ukraine	Both	Colon and rectum cancer	2019	31.27	26.56	36.54
Ukraine	Both	Esophageal cancer	2019	2.99	2.39	3.72
Ukraine	Both	Liver cancer	2019	2.67	2.27	3.19
Ukraine	Both	Pancreatic cancer	2019	8.27	6.92	9.71
Ukraine	Both	Stomach cancer	2019	16.34	13.70	19.23
United Arab Emirates	Both	Colon and rectum cancer	2019	21.36	14.70	29.76
United Arab Emirates	Both	Esophageal cancer	2019	8.08	2.54	13.84
United Arab Emirates	Both	Liver cancer	2019	4.73	2.01	10.82

United Arab Emirates	Both	Pancreatic cancer	2019	17.01	9.09	25.36
United Arab Emirates	Both	Stomach cancer	2019	8.84	6.91	10.98
United Kingdom	Both	Colon and rectum cancer	2019	43.60	36.60	51.50
United Kingdom	Both	Esophageal cancer	2019	8.23	6.80	9.88
United Kingdom	Both	Liver cancer	2019	5.08	4.20	6.11
United Kingdom	Both	Pancreatic cancer	2019	9.22	7.69	10.83
United Kingdom	Both	Stomach cancer	2019	7.37	6.16	8.79
United Republic of Tanzania	Both	Colon and rectum cancer	2019	10.10	8.13	12.70
United Republic of Tanzania	Both	Esophageal cancer	2019	11.85	7.69	16.53
United Republic of Tanzania	Both	Liver cancer	2019	2.25	1.79	2.80
United Republic of Tanzania	Both	Pancreatic cancer	2019	3.63	2.87	4.64
United Republic of Tanzania	Both	Stomach cancer	2019	7.18	5.84	8.56
United States of America	Both	Colon and rectum cancer	2019	41.86	36.15	48.20
United States of America	Both	Esophageal cancer	2019	4.20	3.54	4.98
United States of America	Both	Liver cancer	2019	5.23	4.28	6.29
United States of America	Both	Pancreatic cancer	2019	10.37	8.94	11.96
United States of America	Both	Stomach cancer	2019	5.89	5.10	6.87
United States Virgin Islands	Both	Colon and rectum cancer	2019	43.04	35.69	50.62
United States Virgin Islands	Both	Esophageal cancer	2019	4.19	3.37	4.94
United States Virgin Islands	Both	Liver cancer	2019	2.49	2.06	2.95
United States Virgin Islands	Both	Pancreatic cancer	2019	10.86	8.89	13.01
United States Virgin Islands	Both	Stomach cancer	2019	10.32	8.65	12.03
Uruguay	Both	Colon and rectum cancer	2019	39.49	31.56	49.54
Uruguay	Both	Esophageal cancer	2019	6.12	4.76	7.83
Uruguay	Both	Liver cancer	2019	2.28	1.77	2.90
Uruguay	Both	Pancreatic cancer	2019	13.93	11.03	17.43
Uruguay	Both	Stomach cancer	2019	11.34	8.93	14.19
Uzbekistan	Both	Colon and rectum cancer	2019	12.82	11.10	14.70
Uzbekistan	Both	Esophageal cancer	2019	6.58	5.51	7.66
Uzbekistan	Both	Liver cancer	2019	6.34	5.27	7.46
Uzbekistan	Both	Pancreatic cancer	2019	4.83	4.12	5.65
Uzbekistan	Both	Stomach cancer	2019	13.46	11.39	15.56
Vanuatu	Both	Colon and rectum cancer	2019	10.10	7.56	13.08
Vanuatu	Both	Esophageal cancer	2019	2.56	1.87	3.55
Vanuatu	Both	Liver cancer	2019	9.05	6.21	12.85
Vanuatu	Both	Pancreatic cancer	2019	2.95	2.30	3.84
Vanuatu	Both	Stomach cancer	2019	14.99	11.05	19.54
Venezuela (Bolivarian Republic of)	Both	Colon and rectum cancer	2019	18.58	14.14	24.22
Venezuela (Bolivarian Republic of)	Both	Esophageal cancer	2019	1.97	1.44	2.59
Venezuela (Bolivarian Republic of)	Both	Liver cancer	2019	2.29	1.77	2.97
Venezuela (Bolivarian Republic of)	Both	Pancreatic cancer	2019	5.65	4.27	7.29
Venezuela (Bolivarian Republic of)	Both	Stomach cancer	2019	13.94	10.57	17.87
Viet Nam	Both	Colon and rectum cancer	2019	26.36	20.58	32.37
Viet Nam	Both	Esophageal cancer	2019	2.89	2.04	3.65
Viet Nam	Both	Liver cancer	2019	2.61	2.03	3.26
Viet Nam	Both	Pancreatic cancer	2019	4.30	3.38	5.40
Viet Nam	Both	Stomach cancer	2019	10.59	8.51	12.64
Yemen	Both	Colon and rectum cancer	2019	7.39	5.56	9.99
Yemen	Both	Esophageal cancer	2019	4.18	1.28	6.31
Yemen	Both	Liver cancer	2019	3.20	2.32	4.33
Yemen	Both	Pancreatic cancer	2019	2.12	1.61	2.80
Yemen	Both	Stomach cancer	2019	19.18	14.73	25.05
Zambia	Both	Colon and rectum cancer	2019	13.62	10.14	17.44
Zambia	Both	Esophageal cancer	2019	14.10	9.26	18.84
Zambia	Both	Liver cancer	2019	2.75	2.14	3.41
Zambia	Both	Pancreatic cancer	2019	4.79	3.45	6.58
Zambia	Both	Stomach cancer	2019	8.19	6.46	10.12
Zimbabwe	Both	Colon and rectum cancer	2019	13.76	10.57	17.23
Zimbabwe	Both	Esophageal cancer	2019	15.40	12.22	18.95
Zimbabwe	Both	Liver cancer	2019	13.64	10.27	18.13
Zimbabwe	Both	Pancreatic cancer	2019	7.72	5.95	9.75
Zimbabwe	Both	Stomach cancer	2019	13.11	10.24	16.39

Location_name	Sex_name	Cause_name	Year	Age-standardised incidence rate (per 100 000 person-years)	95% CI (lower)	95% CI (upper)
Afghanistan	Both	Colon and rectum cancer	2019	8.43	6.01	11.18
Afghanistan	Both	Esophageal cancer	2019	6.96	2.25	9.70
Afghanistan	Both	Liver cancer	2019	10.27	7.98	12.93
Afghanistan	Both	Pancreatic cancer	2019	2.72	1.91	3.89
Afghanistan	Both	Stomach cancer	2019	29.30	21.25	36.52
Albania	Both	Colon and rectum cancer	2019	9.15	6.96	11.89
Albania	Both	Esophageal cancer	2019	1.44	1.05	2.06
Albania	Both	Liver cancer	2019	6.84	5.04	9.09
Albania	Both	Pancreatic cancer	2019	6.68	5.05	8.73
Albania	Both	Stomach cancer	2019	10.68	8.05	14.07
Algeria	Both	Colon and rectum cancer	2019	8.05	6.40	9.77
Algeria	Both	Esophageal cancer	2019	1.15	0.85	1.44
Algeria	Both	Liver cancer	2019	2.20	1.72	2.78
Algeria	Both	Pancreatic cancer	2019	4.27	3.47	5.16
Algeria	Both	Stomach cancer	2019	5.10	4.17	6.18
American Samoa	Both	Colon and rectum cancer	2019	16.55	14.07	19.59
American Samoa	Both	Esophageal cancer	2019	1.52	1.18	1.78
American Samoa	Both	Liver cancer	2019	7.02	5.85	8.45
American Samoa	Both	Pancreatic cancer	2019	5.19	4.34	6.16
American Samoa	Both	Stomach cancer	2019	15.79	13.39	18.55
Andorra	Both	Colon and rectum cancer	2019	22.97	17.88	28.86
Andorra	Both	Esophageal cancer	2019	3.56	2.63	4.55
Andorra	Both	Liver cancer	2019	9.44	7.14	12.24
Andorra	Both	Pancreatic cancer	2019	10.26	7.83	13.13
Andorra	Both	Stomach cancer	2019	8.30	6.34	10.66
Angola	Both	Colon and rectum cancer	2019	9.65	7.82	11.99
Angola	Both	Esophageal cancer	2019	8.81	4.73	11.89
Angola	Both	Liver cancer	2019	2.59	2.09	3.23
Angola	Both	Pancreatic cancer	2019	3.40	2.74	4.26
Angola	Both	Stomach cancer	2019	8.96	7.31	11.24
Antigua and Barbuda	Both	Colon and rectum cancer	2019	16.16	14.01	18.45
Antigua and Barbuda	Both	Esophageal cancer	2019	3.11	2.64	3.66
Antigua and Barbuda	Both	Liver cancer	2019	2.85	2.44	3.31
Antigua and Barbuda	Both	Pancreatic cancer	2019	6.39	5.42	7.49
Antigua and Barbuda	Both	Stomach cancer	2019	11.05	9.48	12.67
Argentina	Both	Colon and rectum cancer	2019	23.59	22.00	25.13
Argentina	Both	Esophageal cancer	2019	5.08	4.66	5.53
Argentina	Both	Liver cancer	2019	2.15	2.00	2.33
Argentina	Both	Pancreatic cancer	2019	11.94	10.90	13.02
Argentina	Both	Stomach cancer	2019	9.44	8.78	10.15
Armenia	Both	Colon and rectum cancer	2019	14.30	12.07	16.63
Armenia	Both	Esophageal cancer	2019	1.62	1.34	1.94
Armenia	Both	Liver cancer	2019	7.07	5.88	8.38
Armenia	Both	Pancreatic cancer	2019	10.61	8.81	12.56
Armenia	Both	Stomach cancer	2019	13.01	10.90	15.23
Australia	Both	Colon and rectum cancer	2019	15.49	14.08	16.66
Australia	Both	Esophageal cancer	2019	4.03	3.61	4.45
Australia	Both	Liver cancer	2019	4.20	3.83	4.59
Australia	Both	Pancreatic cancer	2019	8.26	7.45	9.08
Australia	Both	Stomach cancer	2019	3.89	3.51	4.23
Austria	Both	Colon and rectum cancer	2019	13.54	12.36	14.54
Austria	Both	Esophageal cancer	2019	2.49	2.26	2.73
Austria	Both	Liver cancer	2019	4.50	4.04	4.99
Austria	Both	Pancreatic cancer	2019	10.34	9.48	11.24
Austria	Both	Stomach cancer	2019	5.42	4.96	5.87
Azerbaijan	Both	Colon and rectum cancer	2019	12.20	9.77	15.16
Azerbaijan	Both	Esophageal cancer	2019	9.32	6.87	14.35
Azerbaijan	Both	Liver cancer	2019	4.37	3.31	5.94
Azerbaijan	Both	Pancreatic cancer	2019	7.45	6.34	9.10
Azerbaijan	Both	Stomach cancer	2019	22.48	18.75	27.33
Bahamas	Both	Colon and rectum cancer	2019	18.41	15.08	22.19
Bahamas	Both	Esophageal cancer	2019	5.19	4.15	6.40
Bahamas	Both	Liver cancer	2019	3.21	2.66	3.96

Bahamas	Both	Pancreatic cancer	2019	4.69	3.82	5.73
Bahamas	Both	Stomach cancer	2019	9.16	7.51	11.14
Bahrain	Both	Colon and rectum cancer	2019	11.42	9.08	14.03
Bahrain	Both	Esophageal cancer	2019	2.03	1.52	2.57
Bahrain	Both	Liver cancer	2019	5.23	4.12	6.53
Bahrain	Both	Pancreatic cancer	2019	7.59	6.09	9.19
Bahrain	Both	Stomach cancer	2019	6.29	5.13	7.58
Bangladesh	Both	Colon and rectum cancer	2019	4.94	3.40	7.07
Bangladesh	Both	Esophageal cancer	2019	4.00	2.87	6.00
Bangladesh	Both	Liver cancer	2019	2.75	2.17	3.42
Bangladesh	Both	Pancreatic cancer	2019	2.21	1.39	3.30
Bangladesh	Both	Stomach cancer	2019	6.58	5.07	8.60
Barbados	Both	Colon and rectum cancer	2019	23.96	20.09	28.31
Barbados	Both	Esophageal cancer	2019	5.09	4.14	6.13
Barbados	Both	Liver cancer	2019	2.74	2.29	3.27
Barbados	Both	Pancreatic cancer	2019	8.15	6.64	9.67
Barbados	Both	Stomach cancer	2019	10.41	8.65	12.23
Belarus	Both	Colon and rectum cancer	2019	17.03	13.86	21.48
Belarus	Both	Esophageal cancer	2019	2.96	2.24	3.91
Belarus	Both	Liver cancer	2019	2.41	1.76	3.24
Belarus	Both	Pancreatic cancer	2019	7.06	5.54	9.10
Belarus	Both	Stomach cancer	2019	14.10	11.16	17.92
Belgium	Both	Colon and rectum cancer	2019	17.10	15.59	18.44
Belgium	Both	Esophageal cancer	2019	4.52	4.12	4.93
Belgium	Both	Liver cancer	2019	3.78	3.45	4.12
Belgium	Both	Pancreatic cancer	2019	9.28	8.45	10.13
Belgium	Both	Stomach cancer	2019	5.12	4.67	5.54
Belize	Both	Colon and rectum cancer	2019	10.42	9.02	11.99
Belize	Both	Esophageal cancer	2019	2.46	2.10	2.85
Belize	Both	Liver cancer	2019	3.21	2.75	3.70
Belize	Both	Pancreatic cancer	2019	6.35	5.34	7.38
Belize	Both	Stomach cancer	2019	10.17	8.81	11.72
Benin	Both	Colon and rectum cancer	2019	7.63	6.19	9.53
Benin	Both	Esophageal cancer	2019	5.18	3.05	6.99
Benin	Both	Liver cancer	2019	5.12	3.89	6.79
Benin	Both	Pancreatic cancer	2019	4.94	3.95	6.22
Benin	Both	Stomach cancer	2019	13.66	11.09	16.75
Bermuda	Both	Colon and rectum cancer	2019	18.81	15.73	22.69
Bermuda	Both	Esophageal cancer	2019	4.26	3.52	5.21
Bermuda	Both	Liver cancer	2019	2.05	1.70	2.51
Bermuda	Both	Pancreatic cancer	2019	9.59	7.90	11.74
Bermuda	Both	Stomach cancer	2019	5.07	4.24	6.01
Bhutan	Both	Colon and rectum cancer	2019	6.97	4.39	9.42
Bhutan	Both	Esophageal cancer	2019	5.00	3.56	6.86
Bhutan	Both	Liver cancer	2019	3.27	2.29	4.61
Bhutan	Both	Pancreatic cancer	2019	3.30	1.89	4.99
Bhutan	Both	Stomach cancer	2019	8.41	6.21	10.85
Bolivia (Plurinational State of)	Both	Colon and rectum cancer	2019	12.26	8.43	16.20
Bolivia (Plurinational State of)	Both	Esophageal cancer	2019	2.59	2.03	3.22
Bolivia (Plurinational State of)	Both	Liver cancer	2019	5.03	3.77	6.43
Bolivia (Plurinational State of)	Both	Pancreatic cancer	2019	5.34	3.69	7.18
Bolivia (Plurinational State of)	Both	Stomach cancer	2019	36.11	28.77	44.26
Bosnia and Herzegovina	Both	Colon and rectum cancer	2019	22.32	17.81	27.66
Bosnia and Herzegovina	Both	Esophageal cancer	2019	2.06	1.61	2.60
Bosnia and Herzegovina	Both	Liver cancer	2019	8.01	6.40	10.00
Bosnia and Herzegovina	Both	Pancreatic cancer	2019	10.07	8.06	12.48
Bosnia and Herzegovina	Both	Stomach cancer	2019	10.31	8.24	12.97
Botswana	Both	Colon and rectum cancer	2019	15.75	11.59	20.48
Botswana	Both	Esophageal cancer	2019	13.21	8.08	17.32
Botswana	Both	Liver cancer	2019	1.53	1.13	2.02
Botswana	Both	Pancreatic cancer	2019	7.96	5.90	10.63
Botswana	Both	Stomach cancer	2019	9.04	6.99	11.42
Brazil	Both	Colon and rectum cancer	2019	11.67	10.81	12.28
Brazil	Both	Esophageal cancer	2019	5.28	4.95	5.56
Brazil	Both	Liver cancer	2019	2.50	2.33	2.64

Brazil	Both	Pancreatic cancer	2019	6.45	5.94	6.84
Brazil	Both	Stomach cancer	2019	9.87	9.14	10.37
Brunei Darussalam	Both	Colon and rectum cancer	2019	30.26	26.58	34.10
Brunei Darussalam	Both	Esophageal cancer	2019	3.03	2.57	3.73
Brunei Darussalam	Both	Liver cancer	2019	11.53	9.82	13.38
Brunei Darussalam	Both	Pancreatic cancer	2019	9.30	8.06	10.61
Brunei Darussalam	Both	Stomach cancer	2019	14.11	12.38	15.85
Bulgaria	Both	Colon and rectum cancer	2019	25.11	20.41	30.62
Bulgaria	Both	Esophageal cancer	2019	1.95	1.51	2.46
Bulgaria	Both	Liver cancer	2019	4.62	3.68	5.71
Bulgaria	Both	Pancreatic cancer	2019	11.09	8.78	13.87
Bulgaria	Both	Stomach cancer	2019	10.81	8.65	13.46
Burkina Faso	Both	Colon and rectum cancer	2019	7.27	5.82	9.12
Burkina Faso	Both	Esophageal cancer	2019	5.09	2.96	6.48
Burkina Faso	Both	Liver cancer	2019	2.37	1.80	2.96
Burkina Faso	Both	Pancreatic cancer	2019	3.64	2.78	4.61
Burkina Faso	Both	Stomach cancer	2019	15.38	12.72	18.31
Burundi	Both	Colon and rectum cancer	2019	7.17	5.21	10.11
Burundi	Both	Esophageal cancer	2019	12.18	8.18	17.18
Burundi	Both	Liver cancer	2019	3.11	2.17	4.80
Burundi	Both	Pancreatic cancer	2019	2.31	1.72	3.16
Burundi	Both	Stomach cancer	2019	8.99	7.12	11.37
Cabo Verde	Both	Colon and rectum cancer	2019	11.35	8.97	13.38
Cabo Verde	Both	Esophageal cancer	2019	16.38	13.70	18.91
Cabo Verde	Both	Liver cancer	2019	12.34	10.20	14.87
Cabo Verde	Both	Pancreatic cancer	2019	10.82	8.42	13.17
Cabo Verde	Both	Stomach cancer	2019	25.65	22.46	28.98
Cambodia	Both	Colon and rectum cancer	2019	14.02	11.24	16.54
Cambodia	Both	Esophageal cancer	2019	2.97	2.30	4.10
Cambodia	Both	Liver cancer	2019	9.86	7.85	12.03
Cambodia	Both	Pancreatic cancer	2019	3.75	3.01	4.41
Cambodia	Both	Stomach cancer	2019	9.35	7.53	11.05
Cameroon	Both	Colon and rectum cancer	2019	10.64	8.25	13.84
Cameroon	Both	Esophageal cancer	2019	5.98	3.28	8.40
Cameroon	Both	Liver cancer	2019	0.69	0.51	0.92
Cameroon	Both	Pancreatic cancer	2019	7.99	5.69	10.76
Cameroon	Both	Stomach cancer	2019	14.24	11.19	17.99
Canada	Both	Colon and rectum cancer	2019	16.22	14.74	17.44
Canada	Both	Esophageal cancer	2019	3.64	3.27	3.98
Canada	Both	Liver cancer	2019	3.91	3.48	4.32
Canada	Both	Pancreatic cancer	2019	8.55	7.76	9.35
Canada	Both	Stomach cancer	2019	4.40	4.00	4.76
Central African Republic	Both	Colon and rectum cancer	2019	6.37	4.71	8.79
Central African Republic	Both	Esophageal cancer	2019	10.95	6.14	14.94
Central African Republic	Both	Liver cancer	2019	3.42	2.27	5.01
Central African Republic	Both	Pancreatic cancer	2019	2.19	1.60	2.91
Central African Republic	Both	Stomach cancer	2019	12.46	9.46	16.00
Chad	Both	Colon and rectum cancer	2019	7.37	5.77	9.35
Chad	Both	Esophageal cancer	2019	4.95	2.82	6.72
Chad	Both	Liver cancer	2019	5.68	4.37	7.29
Chad	Both	Pancreatic cancer	2019	2.86	2.31	3.56
Chad	Both	Stomach cancer	2019	16.38	13.04	20.17
Chile	Both	Colon and rectum cancer	2019	14.71	13.46	15.85
Chile	Both	Esophageal cancer	2019	4.00	3.54	4.42
Chile	Both	Liver cancer	2019	3.02	2.76	3.29
Chile	Both	Pancreatic cancer	2019	7.76	7.00	8.53
Chile	Both	Stomach cancer	2019	17.63	16.17	18.99
China	Both	Colon and rectum cancer	2019	13.86	11.92	16.01
China	Both	Esophageal cancer	2019	13.15	10.27	15.68
China	Both	Liver cancer	2019	9.41	7.95	11.13
China	Both	Pancreatic cancer	2019	5.99	5.12	6.93
China	Both	Stomach cancer	2019	21.72	18.31	25.31
Colombia	Both	Colon and rectum cancer	2019	9.63	7.48	12.24
Colombia	Both	Esophageal cancer	2019	2.00	1.52	2.58
Colombia	Both	Liver cancer	2019	2.65	2.03	3.40

Colombia	Both	Pancreatic cancer	2019	4.70	3.62	5.98
Colombia	Both	Stomach cancer	2019	12.73	9.91	16.09
Comoros	Both	Colon and rectum cancer	2019	8.56	6.39	10.80
Comoros	Both	Esophageal cancer	2019	11.94	8.15	16.62
Comoros	Both	Liver cancer	2019	3.01	2.12	4.65
Comoros	Both	Pancreatic cancer	2019	3.89	3.03	4.89
Comoros	Both	Stomach cancer	2019	7.16	5.76	8.95
Congo	Both	Colon and rectum cancer	2019	11.40	8.76	14.52
Congo	Both	Esophageal cancer	2019	10.76	5.96	14.53
Congo	Both	Liver cancer	2019	3.14	2.33	4.24
Congo	Both	Pancreatic cancer	2019	5.48	3.65	7.68
Congo	Both	Stomach cancer	2019	8.67	7.14	10.66
Cook Islands	Both	Colon and rectum cancer	2019	7.77	6.54	9.34
Cook Islands	Both	Esophageal cancer	2019	2.67	2.24	3.18
Cook Islands	Both	Liver cancer	2019	11.13	9.03	13.51
Cook Islands	Both	Pancreatic cancer	2019	4.95	4.17	5.85
Cook Islands	Both	Stomach cancer	2019	6.75	5.66	8.16
Costa Rica	Both	Colon and rectum cancer	2019	15.38	12.15	19.28
Costa Rica	Both	Esophageal cancer	2019	1.79	1.37	2.28
Costa Rica	Both	Liver cancer	2019	5.30	4.11	6.74
Costa Rica	Both	Pancreatic cancer	2019	7.20	5.57	9.08
Costa Rica	Both	Stomach cancer	2019	19.02	14.80	23.93
Côte d'Ivoire	Both	Colon and rectum cancer	2019	9.41	7.62	11.47
Côte d'Ivoire	Both	Esophageal cancer	2019	5.35	2.95	7.25
Côte d'Ivoire	Both	Liver cancer	2019	5.06	3.77	6.83
Côte d'Ivoire	Both	Pancreatic cancer	2019	5.48	4.33	6.79
Côte d'Ivoire	Both	Stomach cancer	2019	14.67	12.01	17.84
Croatia	Both	Colon and rectum cancer	2019	25.28	20.14	31.22
Croatia	Both	Esophageal cancer	2019	2.62	1.99	3.38
Croatia	Both	Liver cancer	2019	3.53	2.77	4.48
Croatia	Both	Pancreatic cancer	2019	9.07	7.23	11.36
Croatia	Both	Stomach cancer	2019	9.38	7.50	11.57
Cuba	Both	Colon and rectum cancer	2019	17.83	14.73	21.48
Cuba	Both	Esophageal cancer	2019	5.08	4.05	6.28
Cuba	Both	Liver cancer	2019	2.43	1.95	3.00
Cuba	Both	Pancreatic cancer	2019	6.16	5.03	7.54
Cuba	Both	Stomach cancer	2019	5.67	4.63	6.89
Cyprus	Both	Colon and rectum cancer	2019	14.32	12.40	16.32
Cyprus	Both	Esophageal cancer	2019	1.27	0.97	1.50
Cyprus	Both	Liver cancer	2019	3.42	2.95	3.94
Cyprus	Both	Pancreatic cancer	2019	8.22	7.13	9.37
Cyprus	Both	Stomach cancer	2019	6.66	5.87	7.60
Czechia	Both	Colon and rectum cancer	2019	21.58	17.80	25.98
Czechia	Both	Esophageal cancer	2019	3.09	2.48	3.85
Czechia	Both	Liver cancer	2019	2.99	2.45	3.68
Czechia	Both	Pancreatic cancer	2019	11.81	9.57	14.29
Czechia	Both	Stomach cancer	2019	6.08	4.98	7.33
Democratic People's Republic of Korea	Both	Colon and rectum cancer	2019	10.89	8.14	13.82
Democratic People's Republic of Korea	Both	Esophageal cancer	2019	9.01	7.06	11.56
Democratic People's Republic of Korea	Both	Liver cancer	2019	10.20	7.75	13.30
Democratic People's Republic of Korea	Both	Pancreatic cancer	2019	3.88	2.98	4.84
Democratic People's Republic of Korea	Both	Stomach cancer	2019	22.49	17.92	27.59
Democratic Republic of the Congo	Both	Colon and rectum cancer	2019	6.23	4.15	9.51
Democratic Republic of the Congo	Both	Esophageal cancer	2019	8.71	4.60	12.77
Democratic Republic of the Congo	Both	Liver cancer	2019	2.28	1.77	2.94
Democratic Republic of the Congo	Both	Pancreatic cancer	2019	2.40	1.83	3.10
Democratic Republic of the Congo	Both	Stomach cancer	2019	8.11	6.32	10.24
Denmark	Both	Colon and rectum cancer	2019	21.75	19.76	23.49
Denmark	Both	Esophageal cancer	2019	4.50	4.00	5.04
Denmark	Both	Liver cancer	2019	3.25	2.93	3.57
Denmark	Both	Pancreatic cancer	2019	10.07	8.99	11.09
Denmark	Both	Stomach cancer	2019	4.33	3.93	4.72
Djibouti	Both	Colon and rectum cancer	2019	11.19	8.66	14.66
Djibouti	Both	Esophageal cancer	2019	12.13	7.90	18.30
Djibouti	Both	Liver cancer	2019	3.49	2.29	5.59

Djibouti	Both	Pancreatic cancer	2019	4.31	3.12	5.82
Djibouti	Both	Stomach cancer	2019	8.17	6.37	10.74
Dominica	Both	Colon and rectum cancer	2019	14.96	12.42	18.09
Dominica	Both	Esophageal cancer	2019	5.14	4.14	6.32
Dominica	Both	Liver cancer	2019	3.39	2.76	4.16
Dominica	Both	Pancreatic cancer	2019	8.54	6.83	10.36
Dominica	Both	Stomach cancer	2019	20.49	16.91	24.66
Dominican Republic	Both	Colon and rectum cancer	2019	12.42	9.23	16.14
Dominican Republic	Both	Esophageal cancer	2019	2.55	1.79	3.52
Dominican Republic	Both	Liver cancer	2019	4.92	3.40	7.23
Dominican Republic	Both	Pancreatic cancer	2019	3.72	2.56	5.01
Dominican Republic	Both	Stomach cancer	2019	8.70	6.68	11.31
Ecuador	Both	Colon and rectum cancer	2019	11.34	9.07	14.16
Ecuador	Both	Esophageal cancer	2019	1.68	1.30	2.21
Ecuador	Both	Liver cancer	2019	3.71	2.97	4.73
Ecuador	Both	Pancreatic cancer	2019	5.83	4.64	7.31
Ecuador	Both	Stomach cancer	2019	21.86	17.57	27.51
Egypt	Both	Colon and rectum cancer	2019	7.45	5.40	10.11
Egypt	Both	Esophageal cancer	2019	1.54	1.06	2.09
Egypt	Both	Liver cancer	2019	21.25	15.44	28.92
Egypt	Both	Pancreatic cancer	2019	4.43	3.07	6.15
Egypt	Both	Stomach cancer	2019	5.21	4.00	6.76
El Salvador	Both	Colon and rectum cancer	2019	9.05	6.98	11.68
El Salvador	Both	Esophageal cancer	2019	1.68	1.28	2.17
El Salvador	Both	Liver cancer	2019	2.12	1.61	2.76
El Salvador	Both	Pancreatic cancer	2019	5.25	3.94	6.76
El Salvador	Both	Stomach cancer	2019	16.24	12.51	20.62
Equatorial Guinea	Both	Colon and rectum cancer	2019	14.24	9.12	20.02
Equatorial Guinea	Both	Esophageal cancer	2019	9.20	4.77	15.56
Equatorial Guinea	Both	Liver cancer	2019	3.48	2.04	5.04
Equatorial Guinea	Both	Pancreatic cancer	2019	6.36	3.97	9.37
Equatorial Guinea	Both	Stomach cancer	2019	6.58	4.92	9.00
Eritrea	Both	Colon and rectum cancer	2019	9.97	7.87	12.70
Eritrea	Both	Esophageal cancer	2019	13.92	8.85	18.99
Eritrea	Both	Liver cancer	2019	3.32	2.36	4.73
Eritrea	Both	Pancreatic cancer	2019	3.13	2.15	4.29
Eritrea	Both	Stomach cancer	2019	10.45	8.07	13.25
Estonia	Both	Colon and rectum cancer	2019	18.51	14.81	23.22
Estonia	Both	Esophageal cancer	2019	2.91	2.19	3.74
Estonia	Both	Liver cancer	2019	3.61	2.76	4.54
Estonia	Both	Pancreatic cancer	2019	10.19	7.98	12.86
Estonia	Both	Stomach cancer	2019	11.38	8.93	14.48
Eswatini	Both	Colon and rectum cancer	2019	13.52	9.32	18.23
Eswatini	Both	Esophageal cancer	2019	15.86	10.18	21.40
Eswatini	Both	Liver cancer	2019	19.09	5.98	33.88
Eswatini	Both	Pancreatic cancer	2019	8.18	5.65	11.23
Eswatini	Both	Stomach cancer	2019	9.15	6.98	11.75
Ethiopia	Both	Colon and rectum cancer	2019	7.33	5.47	10.36
Ethiopia	Both	Esophageal cancer	2019	2.87	2.24	4.03
Ethiopia	Both	Liver cancer	2019	3.02	2.40	3.84
Ethiopia	Both	Pancreatic cancer	2019	1.60	1.07	2.32
Ethiopia	Both	Stomach cancer	2019	6.64	5.45	8.34
Fiji	Both	Colon and rectum cancer	2019	11.65	9.51	14.20
Fiji	Both	Esophageal cancer	2019	2.90	1.65	3.72
Fiji	Both	Liver cancer	2019	6.23	4.86	7.83
Fiji	Both	Pancreatic cancer	2019	4.34	3.43	5.37
Fiji	Both	Stomach cancer	2019	7.44	5.99	9.19
Finland	Both	Colon and rectum cancer	2019	12.33	11.25	13.35
Finland	Both	Esophageal cancer	2019	2.50	2.23	2.79
Finland	Both	Liver cancer	2019	4.03	3.68	4.42
Finland	Both	Pancreatic cancer	2019	10.71	9.63	11.84
Finland	Both	Stomach cancer	2019	4.78	4.35	5.20
France	Both	Colon and rectum cancer	2019	16.41	14.69	17.82
France	Both	Esophageal cancer	2019	3.96	3.55	4.35
France	Both	Liver cancer	2019	5.80	5.10	6.56

France	Both	Pancreatic cancer	2019	9.76	8.72	10.72
France	Both	Stomach cancer	2019	4.86	4.36	5.27
Gabon	Both	Colon and rectum cancer	2019	14.86	11.25	18.24
Gabon	Both	Esophageal cancer	2019	11.22	6.38	14.63
Gabon	Both	Liver cancer	2019	3.54	2.33	5.01
Gabon	Both	Pancreatic cancer	2019	8.18	5.73	11.17
Gabon	Both	Stomach cancer	2019	8.15	6.43	9.99
Gambia	Both	Colon and rectum cancer	2019	6.56	4.78	8.76
Gambia	Both	Esophageal cancer	2019	2.22	1.67	2.79
Gambia	Both	Liver cancer	2019	39.51	29.01	50.99
Gambia	Both	Pancreatic cancer	2019	3.52	2.40	4.81
Gambia	Both	Stomach cancer	2019	5.81	4.64	7.07
Georgia	Both	Colon and rectum cancer	2019	13.19	11.08	15.44
Georgia	Both	Esophageal cancer	2019	1.85	1.52	2.22
Georgia	Both	Liver cancer	2019	3.63	2.97	4.42
Georgia	Both	Pancreatic cancer	2019	6.28	5.18	7.46
Georgia	Both	Stomach cancer	2019	14.07	11.86	16.62
Germany	Both	Colon and rectum cancer	2019	18.01	16.54	19.26
Germany	Both	Esophageal cancer	2019	3.71	3.39	4.07
Germany	Both	Liver cancer	2019	4.02	3.70	4.33
Germany	Both	Pancreatic cancer	2019	10.82	9.86	11.83
Germany	Both	Stomach cancer	2019	6.86	6.29	7.40
Ghana	Both	Colon and rectum cancer	2019	8.76	6.96	10.92
Ghana	Both	Esophageal cancer	2019	3.52	2.42	4.54
Ghana	Both	Liver cancer	2019	6.00	4.59	7.59
Ghana	Both	Pancreatic cancer	2019	9.58	7.34	12.57
Ghana	Both	Stomach cancer	2019	8.65	6.92	10.76
Greece	Both	Colon and rectum cancer	2019	14.87	13.67	15.87
Greece	Both	Esophageal cancer	2019	1.39	1.26	1.52
Greece	Both	Liver cancer	2019	3.21	2.95	3.47
Greece	Both	Pancreatic cancer	2019	9.98	9.03	10.84
Greece	Both	Stomach cancer	2019	8.05	7.39	8.65
Greenland	Both	Colon and rectum cancer	2019	31.38	26.04	37.13
Greenland	Both	Esophageal cancer	2019	13.13	10.43	15.69
Greenland	Both	Liver cancer	2019	6.35	5.03	7.96
Greenland	Both	Pancreatic cancer	2019	19.29	15.73	22.84
Greenland	Both	Stomach cancer	2019	10.82	8.84	12.93
Grenada	Both	Colon and rectum cancer	2019	19.29	17.44	21.14
Grenada	Both	Esophageal cancer	2019	6.66	5.85	7.56
Grenada	Both	Liver cancer	2019	3.13	2.76	3.54
Grenada	Both	Pancreatic cancer	2019	9.75	8.58	11.03
Grenada	Both	Stomach cancer	2019	10.43	9.38	11.58
Guam	Both	Colon and rectum cancer	2019	14.16	12.02	16.74
Guam	Both	Esophageal cancer	2019	2.65	2.08	3.22
Guam	Both	Liver cancer	2019	5.79	4.71	7.05
Guam	Both	Pancreatic cancer	2019	5.26	4.36	6.25
Guam	Both	Stomach cancer	2019	5.74	4.82	6.79
Guatemala	Both	Colon and rectum cancer	2019	9.09	7.32	11.27
Guatemala	Both	Esophageal cancer	2019	2.23	1.77	2.80
Guatemala	Both	Liver cancer	2019	4.81	3.84	6.01
Guatemala	Both	Pancreatic cancer	2019	4.73	3.70	5.89
Guatemala	Both	Stomach cancer	2019	27.97	22.45	34.43
Guinea	Both	Colon and rectum cancer	2019	7.24	5.51	9.24
Guinea	Both	Esophageal cancer	2019	2.02	1.54	2.57
Guinea	Both	Liver cancer	2019	34.05	23.98	44.01
Guinea	Both	Pancreatic cancer	2019	1.91	1.46	2.38
Guinea	Both	Stomach cancer	2019	16.24	12.95	20.00
Guinea-Bissau	Both	Colon and rectum cancer	2019	9.06	6.93	11.29
Guinea-Bissau	Both	Esophageal cancer	2019	6.42	3.55	8.59
Guinea-Bissau	Both	Liver cancer	2019	6.36	4.67	8.51
Guinea-Bissau	Both	Pancreatic cancer	2019	4.72	3.26	6.34
Guinea-Bissau	Both	Stomach cancer	2019	19.24	15.38	23.58
Guyana	Both	Colon and rectum cancer	2019	14.68	11.87	18.25
Guyana	Both	Esophageal cancer	2019	2.18	1.68	2.77
Guyana	Both	Liver cancer	2019	3.20	2.53	4.03

Guyana	Both	Pancreatic cancer	2019	6.14	4.72	7.69
Guyana	Both	Stomach cancer	2019	8.87	7.07	11.02
Haiti	Both	Colon and rectum cancer	2019	10.53	7.33	14.09
Haiti	Both	Esophageal cancer	2019	3.75	2.55	5.46
Haiti	Both	Liver cancer	2019	4.42	2.67	6.66
Haiti	Both	Pancreatic cancer	2019	2.54	1.78	3.49
Haiti	Both	Stomach cancer	2019	15.99	10.48	21.19
Honduras	Both	Colon and rectum cancer	2019	7.65	5.34	10.67
Honduras	Both	Esophageal cancer	2019	1.80	1.27	2.32
Honduras	Both	Liver cancer	2019	16.14	7.41	23.50
Honduras	Both	Pancreatic cancer	2019	4.61	2.80	6.54
Honduras	Both	Stomach cancer	2019	15.88	13.16	20.02
Hungary	Both	Colon and rectum cancer	2019	28.56	23.65	34.03
Hungary	Both	Esophageal cancer	2019	3.61	2.89	4.46
Hungary	Both	Liver cancer	2019	2.65	2.18	3.23
Hungary	Both	Pancreatic cancer	2019	12.02	9.90	14.42
Hungary	Both	Stomach cancer	2019	8.57	7.11	10.20
Iceland	Both	Colon and rectum cancer	2019	11.81	10.53	13.16
Iceland	Both	Esophageal cancer	2019	3.92	3.41	4.51
Iceland	Both	Liver cancer	2019	2.84	2.51	3.20
Iceland	Both	Pancreatic cancer	2019	8.12	7.08	9.23
Iceland	Both	Stomach cancer	2019	4.59	4.05	5.12
India	Both	Colon and rectum cancer	2019	7.50	6.35	8.76
India	Both	Esophageal cancer	2019	3.49	2.90	5.05
India	Both	Liver cancer	2019	2.75	2.32	3.27
India	Both	Pancreatic cancer	2019	3.13	2.71	3.60
India	Both	Stomach cancer	2019	7.32	6.30	8.44
Indonesia	Both	Colon and rectum cancer	2019	15.58	10.54	19.86
Indonesia	Both	Esophageal cancer	2019	2.51	2.02	3.58
Indonesia	Both	Liver cancer	2019	2.45	2.08	2.76
Indonesia	Both	Pancreatic cancer	2019	5.40	3.51	7.49
Indonesia	Both	Stomach cancer	2019	6.82	5.74	7.68
Iran (Islamic Republic of)	Both	Colon and rectum cancer	2019	9.31	8.53	10.08
Iran (Islamic Republic of)	Both	Esophageal cancer	2019	4.54	3.54	4.99
Iran (Islamic Republic of)	Both	Liver cancer	2019	3.64	3.29	4.02
Iran (Islamic Republic of)	Both	Pancreatic cancer	2019	4.89	4.51	5.32
Iran (Islamic Republic of)	Both	Stomach cancer	2019	16.17	14.86	17.40
Iraq	Both	Colon and rectum cancer	2019	8.32	6.58	10.16
Iraq	Both	Esophageal cancer	2019	1.30	1.01	1.59
Iraq	Both	Liver cancer	2019	6.07	4.72	7.47
Iraq	Both	Pancreatic cancer	2019	6.12	4.68	7.47
Iraq	Both	Stomach cancer	2019	5.71	4.55	6.89
Ireland	Both	Colon and rectum cancer	2019	18.01	16.26	19.51
Ireland	Both	Esophageal cancer	2019	6.41	5.59	7.31
Ireland	Both	Liver cancer	2019	3.40	3.04	3.75
Ireland	Both	Pancreatic cancer	2019	9.16	8.11	10.17
Ireland	Both	Stomach cancer	2019	5.79	5.25	6.32
Israel	Both	Colon and rectum cancer	2019	16.56	14.91	17.85
Israel	Both	Esophageal cancer	2019	1.53	1.35	1.71
Israel	Both	Liver cancer	2019	2.79	2.55	3.04
Israel	Both	Pancreatic cancer	2019	10.73	9.60	11.68
Israel	Both	Stomach cancer	2019	6.04	5.48	6.52
Italy	Both	Colon and rectum cancer	2019	15.83	14.41	16.70
Italy	Both	Esophageal cancer	2019	1.83	1.69	1.96
Italy	Both	Liver cancer	2019	4.80	4.36	5.17
Italy	Both	Pancreatic cancer	2019	9.59	8.70	10.18
Italy	Both	Stomach cancer	2019	8.26	7.50	8.72
Jamaica	Both	Colon and rectum cancer	2019	16.52	13.35	20.35
Jamaica	Both	Esophageal cancer	2019	2.70	2.10	3.43
Jamaica	Both	Liver cancer	2019	2.75	2.19	3.41
Jamaica	Both	Pancreatic cancer	2019	5.14	4.03	6.41
Jamaica	Both	Stomach cancer	2019	9.53	7.62	11.78
Japan	Both	Colon and rectum cancer	2019	15.85	13.93	16.93
Japan	Both	Esophageal cancer	2019	3.91	3.59	4.19
Japan	Both	Liver cancer	2019	8.78	7.80	9.42

Japan	Both	Pancreatic cancer	2019	9.60	8.40	10.26
Japan	Both	Stomach cancer	2019	14.07	12.45	15.01
Jordan	Both	Colon and rectum cancer	2019	13.07	10.93	15.46
Jordan	Both	Esophageal cancer	2019	1.16	0.95	1.43
Jordan	Both	Liver cancer	2019	2.38	1.91	2.97
Jordan	Both	Pancreatic cancer	2019	5.40	4.54	6.43
Jordan	Both	Stomach cancer	2019	5.14	4.31	6.12
Kazakhstan	Both	Colon and rectum cancer	2019	13.50	11.79	15.28
Kazakhstan	Both	Esophageal cancer	2019	7.80	6.67	9.02
Kazakhstan	Both	Liver cancer	2019	6.54	5.63	7.50
Kazakhstan	Both	Pancreatic cancer	2019	6.67	5.72	7.73
Kazakhstan	Both	Stomach cancer	2019	14.11	12.33	16.09
Kenya	Both	Colon and rectum cancer	2019	8.14	6.52	10.04
Kenya	Both	Esophageal cancer	2019	13.72	10.54	19.58
Kenya	Both	Liver cancer	2019	3.34	2.28	4.81
Kenya	Both	Pancreatic cancer	2019	3.43	2.67	4.44
Kenya	Both	Stomach cancer	2019	9.83	8.11	11.80
Kiribati	Both	Colon and rectum cancer	2019	10.76	8.26	14.05
Kiribati	Both	Esophageal cancer	2019	8.23	3.55	10.56
Kiribati	Both	Liver cancer	2019	11.95	9.29	15.08
Kiribati	Both	Pancreatic cancer	2019	3.00	2.30	3.90
Kiribati	Both	Stomach cancer	2019	16.60	13.28	20.33
Kuwait	Both	Colon and rectum cancer	2019	10.14	8.41	12.04
Kuwait	Both	Esophageal cancer	2019	1.53	1.22	1.90
Kuwait	Both	Liver cancer	2019	2.36	1.85	2.96
Kuwait	Both	Pancreatic cancer	2019	5.56	4.50	6.79
Kuwait	Both	Stomach cancer	2019	3.45	2.86	4.15
Kyrgyzstan	Both	Colon and rectum cancer	2019	8.24	7.25	9.34
Kyrgyzstan	Both	Esophageal cancer	2019	4.45	3.79	5.17
Kyrgyzstan	Both	Liver cancer	2019	2.70	2.31	3.09
Kyrgyzstan	Both	Pancreatic cancer	2019	5.30	4.56	6.06
Kyrgyzstan	Both	Stomach cancer	2019	16.78	14.69	19.02
Lao People's Democratic Republic	Both	Colon and rectum cancer	2019	13.33	9.60	17.23
Lao People's Democratic Republic	Both	Esophageal cancer	2019	2.53	1.77	3.69
Lao People's Democratic Republic	Both	Liver cancer	2019	7.11	5.40	9.01
Lao People's Democratic Republic	Both	Pancreatic cancer	2019	3.52	2.78	4.38
Lao People's Democratic Republic	Both	Stomach cancer	2019	8.27	6.40	10.19
Latvia	Both	Colon and rectum cancer	2019	17.63	14.77	21.21
Latvia	Both	Esophageal cancer	2019	3.30	2.55	4.25
Latvia	Both	Liver cancer	2019	2.70	2.25	3.29
Latvia	Both	Pancreatic cancer	2019	10.15	8.42	12.38
Latvia	Both	Stomach cancer	2019	11.56	9.72	13.89
Lebanon	Both	Colon and rectum cancer	2019	17.55	14.26	23.15
Lebanon	Both	Esophageal cancer	2019	1.25	0.94	1.66
Lebanon	Both	Liver cancer	2019	2.97	2.19	4.14
Lebanon	Both	Pancreatic cancer	2019	6.61	5.13	8.13
Lebanon	Both	Stomach cancer	2019	6.78	5.51	8.97
Lesotho	Both	Colon and rectum cancer	2019	11.67	8.66	15.10
Lesotho	Both	Esophageal cancer	2019	15.44	9.65	20.36
Lesotho	Both	Liver cancer	2019	15.17	6.45	23.71
Lesotho	Both	Pancreatic cancer	2019	6.11	4.31	8.13
Lesotho	Both	Stomach cancer	2019	12.35	9.40	15.57
Liberia	Both	Colon and rectum cancer	2019	6.68	4.57	9.51
Liberia	Both	Esophageal cancer	2019	5.21	2.87	7.15
Liberia	Both	Liver cancer	2019	5.29	3.89	7.78
Liberia	Both	Pancreatic cancer	2019	5.07	3.73	6.67
Liberia	Both	Stomach cancer	2019	12.33	9.54	15.64
Libya	Both	Colon and rectum cancer	2019	12.45	9.07	15.84
Libya	Both	Esophageal cancer	2019	1.41	1.01	1.80
Libya	Both	Liver cancer	2019	5.05	3.79	6.86
Libya	Both	Pancreatic cancer	2019	7.28	5.63	9.32
Libya	Both	Stomach cancer	2019	5.64	4.36	7.12
Lithuania	Both	Colon and rectum cancer	2019	16.90	14.05	20.27
Lithuania	Both	Esophageal cancer	2019	3.68	2.92	4.61
Lithuania	Both	Liver cancer	2019	3.03	2.38	3.70

Lithuania	Both	Pancreatic cancer	2019	9.18	7.45	11.31
Lithuania	Both	Stomach cancer	2019	11.66	9.45	14.13
Luxembourg	Both	Colon and rectum cancer	2019	15.97	13.87	18.08
Luxembourg	Both	Esophageal cancer	2019	3.34	2.83	4.00
Luxembourg	Both	Liver cancer	2019	3.62	2.94	4.50
Luxembourg	Both	Pancreatic cancer	2019	9.07	7.86	10.32
Luxembourg	Both	Stomach cancer	2019	4.75	4.07	5.42
Madagascar	Both	Colon and rectum cancer	2019	7.11	5.27	9.34
Madagascar	Both	Esophageal cancer	2019	10.31	6.61	14.36
Madagascar	Both	Liver cancer	2019	2.62	1.80	4.02
Madagascar	Both	Pancreatic cancer	2019	2.43	1.74	3.22
Madagascar	Both	Stomach cancer	2019	7.02	5.40	8.95
Malawi	Both	Colon and rectum cancer	2019	6.06	4.74	7.49
Malawi	Both	Esophageal cancer	2019	25.76	19.76	33.94
Malawi	Both	Liver cancer	2019	3.04	2.43	3.74
Malawi	Both	Pancreatic cancer	2019	3.11	2.42	3.87
Malawi	Both	Stomach cancer	2019	3.60	2.96	4.24
Malaysia	Both	Colon and rectum cancer	2019	20.32	16.15	24.85
Malaysia	Both	Esophageal cancer	2019	2.70	2.10	3.36
Malaysia	Both	Liver cancer	2019	6.22	4.78	7.94
Malaysia	Both	Pancreatic cancer	2019	3.80	2.98	4.76
Malaysia	Both	Stomach cancer	2019	7.04	5.73	8.64
Maldives	Both	Colon and rectum cancer	2019	8.71	7.13	10.38
Maldives	Both	Esophageal cancer	2019	2.01	1.62	2.44
Maldives	Both	Liver cancer	2019	5.63	4.46	6.89
Maldives	Both	Pancreatic cancer	2019	4.49	3.69	5.38
Maldives	Both	Stomach cancer	2019	3.60	2.98	4.26
Mali	Both	Colon and rectum cancer	2019	7.84	6.29	9.73
Mali	Both	Esophageal cancer	2019	2.69	2.08	3.53
Mali	Both	Liver cancer	2019	15.03	11.25	19.52
Mali	Both	Pancreatic cancer	2019	3.45	2.63	4.42
Mali	Both	Stomach cancer	2019	18.22	14.75	22.65
Malta	Both	Colon and rectum cancer	2019	14.08	12.38	15.89
Malta	Both	Esophageal cancer	2019	2.15	1.81	2.54
Malta	Both	Liver cancer	2019	2.20	1.93	2.49
Malta	Both	Pancreatic cancer	2019	8.68	7.34	9.99
Malta	Both	Stomach cancer	2019	4.93	4.34	5.55
Marshall Islands	Both	Colon and rectum cancer	2019	12.29	9.55	15.44
Marshall Islands	Both	Esophageal cancer	2019	2.87	2.00	3.93
Marshall Islands	Both	Liver cancer	2019	10.57	7.64	14.31
Marshall Islands	Both	Pancreatic cancer	2019	4.01	3.01	5.21
Marshall Islands	Both	Stomach cancer	2019	16.99	13.14	21.22
Mauritania	Both	Colon and rectum cancer	2019	8.35	6.53	10.34
Mauritania	Both	Esophageal cancer	2019	4.46	2.64	5.91
Mauritania	Both	Liver cancer	2019	4.43	3.30	5.68
Mauritania	Both	Pancreatic cancer	2019	6.47	4.79	8.33
Mauritania	Both	Stomach cancer	2019	11.29	8.81	14.13
Mauritius	Both	Colon and rectum cancer	2019	12.77	10.53	15.50
Mauritius	Both	Esophageal cancer	2019	2.52	2.02	3.15
Mauritius	Both	Liver cancer	2019	2.00	1.55	2.57
Mauritius	Both	Pancreatic cancer	2019	5.35	4.29	6.53
Mauritius	Both	Stomach cancer	2019	7.14	5.86	8.62
Mexico	Both	Colon and rectum cancer	2019	9.24	7.95	10.52
Mexico	Both	Esophageal cancer	2019	1.51	1.28	1.75
Mexico	Both	Liver cancer	2019	3.69	3.18	4.22
Mexico	Both	Pancreatic cancer	2019	6.01	5.16	6.87
Mexico	Both	Stomach cancer	2019	8.86	7.64	10.16
Micronesia (Federated States of)	Both	Colon and rectum cancer	2019	13.08	9.50	16.86
Micronesia (Federated States of)	Both	Esophageal cancer	2019	3.23	2.26	4.39
Micronesia (Federated States of)	Both	Liver cancer	2019	10.70	7.24	14.74
Micronesia (Federated States of)	Both	Pancreatic cancer	2019	5.31	3.75	7.09
Micronesia (Federated States of)	Both	Stomach cancer	2019	17.40	13.30	21.65
Monaco	Both	Colon and rectum cancer	2019	24.27	19.64	28.78
Monaco	Both	Esophageal cancer	2019	5.27	4.07	6.45
Monaco	Both	Liver cancer	2019	7.63	6.05	9.39

Monaco	Both	Pancreatic cancer	2019	17.81	14.20	21.23
Monaco	Both	Stomach cancer	2019	6.62	5.31	7.86
Mongolia	Both	Colon and rectum cancer	2019	9.31	7.44	11.69
Mongolia	Both	Esophageal cancer	2019	24.53	15.33	31.27
Mongolia	Both	Liver cancer	2019	115.23	91.48	142.48
Mongolia	Both	Pancreatic cancer	2019	6.28	4.99	7.86
Mongolia	Both	Stomach cancer	2019	46.04	36.30	57.48
Montenegro	Both	Colon and rectum cancer	2019	17.23	14.08	20.40
Montenegro	Both	Esophageal cancer	2019	2.37	1.88	2.93
Montenegro	Both	Liver cancer	2019	6.09	4.93	7.48
Montenegro	Both	Pancreatic cancer	2019	10.89	9.14	12.90
Montenegro	Both	Stomach cancer	2019	7.47	6.28	8.82
Morocco	Both	Colon and rectum cancer	2019	8.48	6.31	10.51
Morocco	Both	Esophageal cancer	2019	1.65	1.26	1.99
Morocco	Both	Liver cancer	2019	2.31	1.77	2.79
Morocco	Both	Pancreatic cancer	2019	3.96	2.82	5.17
Morocco	Both	Stomach cancer	2019	4.84	3.76	5.72
Mozambique	Both	Colon and rectum cancer	2019	8.66	6.46	11.20
Mozambique	Both	Esophageal cancer	2019	8.37	6.12	11.08
Mozambique	Both	Liver cancer	2019	3.99	2.79	5.24
Mozambique	Both	Pancreatic cancer	2019	3.91	2.75	5.48
Mozambique	Both	Stomach cancer	2019	7.59	6.06	9.42
Myanmar	Both	Colon and rectum cancer	2019	12.82	9.60	16.23
Myanmar	Both	Esophageal cancer	2019	2.37	1.90	3.58
Myanmar	Both	Liver cancer	2019	4.44	3.76	5.26
Myanmar	Both	Pancreatic cancer	2019	3.95	3.11	5.07
Myanmar	Both	Stomach cancer	2019	7.22	6.10	8.66
Namibia	Both	Colon and rectum cancer	2019	8.68	6.97	10.81
Namibia	Both	Esophageal cancer	2019	2.74	2.14	3.46
Namibia	Both	Liver cancer	2019	3.46	2.66	4.39
Namibia	Both	Pancreatic cancer	2019	4.11	3.23	5.22
Namibia	Both	Stomach cancer	2019	3.80	3.11	4.63
Nauru	Both	Colon and rectum cancer	2019	16.55	11.75	21.15
Nauru	Both	Esophageal cancer	2019	3.31	2.35	4.32
Nauru	Both	Liver cancer	2019	9.39	6.70	12.73
Nauru	Both	Pancreatic cancer	2019	6.08	4.26	8.17
Nauru	Both	Stomach cancer	2019	17.94	14.73	21.60
Nepal	Both	Colon and rectum cancer	2019	5.40	3.89	7.42
Nepal	Both	Esophageal cancer	2019	5.10	3.81	6.80
Nepal	Both	Liver cancer	2019	2.36	1.71	3.39
Nepal	Both	Pancreatic cancer	2019	2.99	1.79	4.42
Nepal	Both	Stomach cancer	2019	8.96	7.05	11.34
Netherlands	Both	Colon and rectum cancer	2019	21.75	19.80	23.55
Netherlands	Both	Esophageal cancer	2019	6.98	6.34	7.64
Netherlands	Both	Liver cancer	2019	2.75	2.53	2.97
Netherlands	Both	Pancreatic cancer	2019	11.10	10.03	12.16
Netherlands	Both	Stomach cancer	2019	6.86	6.20	7.45
New Zealand	Both	Colon and rectum cancer	2019	20.26	18.34	21.79
New Zealand	Both	Esophageal cancer	2019	3.93	3.53	4.33
New Zealand	Both	Liver cancer	2019	3.72	3.44	4.00
New Zealand	Both	Pancreatic cancer	2019	7.46	6.80	8.11
New Zealand	Both	Stomach cancer	2019	4.65	4.26	4.98
Nicaragua	Both	Colon and rectum cancer	2019	10.86	9.23	12.50
Nicaragua	Both	Esophageal cancer	2019	1.32	1.06	1.70
Nicaragua	Both	Liver cancer	2019	4.10	3.37	4.96
Nicaragua	Both	Pancreatic cancer	2019	5.97	5.00	6.96
Nicaragua	Both	Stomach cancer	2019	15.03	12.54	17.62
Niger	Both	Colon and rectum cancer	2019	5.62	4.24	7.51
Niger	Both	Esophageal cancer	2019	4.15	2.40	5.56
Niger	Both	Liver cancer	2019	0.65	0.49	0.84
Niger	Both	Pancreatic cancer	2019	2.36	1.58	3.33
Niger	Both	Stomach cancer	2019	14.68	11.25	18.24
Nigeria	Both	Colon and rectum cancer	2019	8.63	6.75	10.78
Nigeria	Both	Esophageal cancer	2019	1.00	0.71	1.76
Nigeria	Both	Liver cancer	2019	3.57	2.87	4.44

Nigeria	Both	Pancreatic cancer	2019	4.27	3.34	5.25
Nigeria	Both	Stomach cancer	2019	4.43	3.60	5.42
Niue	Both	Colon and rectum cancer	2019	13.61	10.77	17.01
Niue	Both	Esophageal cancer	2019	2.52	1.92	3.05
Niue	Both	Liver cancer	2019	7.34	5.67	9.36
Niue	Both	Pancreatic cancer	2019	6.40	4.89	8.14
Niue	Both	Stomach cancer	2019	10.43	8.58	12.41
North Macedonia	Both	Colon and rectum cancer	2019	22.13	17.80	27.27
North Macedonia	Both	Esophageal cancer	2019	1.56	1.23	1.97
North Macedonia	Both	Liver cancer	2019	8.82	7.01	11.10
North Macedonia	Both	Pancreatic cancer	2019	10.81	8.64	13.42
North Macedonia	Both	Stomach cancer	2019	15.32	12.30	19.13
Northern Mariana Islands	Both	Colon and rectum cancer	2019	18.34	15.88	20.86
Northern Mariana Islands	Both	Esophageal cancer	2019	3.01	2.12	3.55
Northern Mariana Islands	Both	Liver cancer	2019	7.84	6.50	9.45
Northern Mariana Islands	Both	Pancreatic cancer	2019	7.75	6.62	8.96
Northern Mariana Islands	Both	Stomach cancer	2019	12.02	10.31	13.89
Norway	Both	Colon and rectum cancer	2019	19.84	18.04	21.11
Norway	Both	Esophageal cancer	2019	2.44	2.26	2.65
Norway	Both	Liver cancer	2019	2.45	2.20	2.76
Norway	Both	Pancreatic cancer	2019	9.02	8.26	9.62
Norway	Both	Stomach cancer	2019	4.49	4.12	4.82
Oman	Both	Colon and rectum cancer	2019	10.20	8.48	12.04
Oman	Both	Esophageal cancer	2019	2.38	1.87	2.78
Oman	Both	Liver cancer	2019	4.75	3.98	5.69
Oman	Both	Pancreatic cancer	2019	6.21	5.43	7.15
Oman	Both	Stomach cancer	2019	7.98	6.95	9.13
Pakistan	Both	Colon and rectum cancer	2019	8.34	6.72	10.41
Pakistan	Both	Esophageal cancer	2019	8.23	6.62	9.96
Pakistan	Both	Liver cancer	2019	3.46	2.75	4.30
Pakistan	Both	Pancreatic cancer	2019	3.10	2.41	3.96
Pakistan	Both	Stomach cancer	2019	6.87	5.71	8.31
Palau	Both	Colon and rectum cancer	2019	13.69	10.87	16.66
Palau	Both	Esophageal cancer	2019	2.51	2.00	3.12
Palau	Both	Liver cancer	2019	9.79	7.59	12.53
Palau	Both	Pancreatic cancer	2019	11.95	9.23	14.96
Palau	Both	Stomach cancer	2019	11.10	8.87	13.73
Palestine	Both	Colon and rectum cancer	2019	19.60	16.69	22.68
Palestine	Both	Esophageal cancer	2019	1.16	0.94	1.58
Palestine	Both	Liver cancer	2019	6.61	5.56	7.88
Palestine	Both	Pancreatic cancer	2019	7.01	5.84	8.30
Palestine	Both	Stomach cancer	2019	7.12	6.13	8.22
Panama	Both	Colon and rectum cancer	2019	10.90	8.54	13.81
Panama	Both	Esophageal cancer	2019	1.64	1.24	2.12
Panama	Both	Liver cancer	2019	3.06	2.32	3.93
Panama	Both	Pancreatic cancer	2019	4.88	3.74	6.27
Panama	Both	Stomach cancer	2019	10.76	8.45	13.63
Papua New Guinea	Both	Colon and rectum cancer	2019	7.46	5.48	9.64
Papua New Guinea	Both	Esophageal cancer	2019	2.02	1.47	3.04
Papua New Guinea	Both	Liver cancer	2019	1.67	1.31	2.12
Papua New Guinea	Both	Pancreatic cancer	2019	1.81	1.28	2.56
Papua New Guinea	Both	Stomach cancer	2019	13.95	10.27	18.04
Paraguay	Both	Colon and rectum cancer	2019	12.16	9.47	15.31
Paraguay	Both	Esophageal cancer	2019	3.99	2.96	5.22
Paraguay	Both	Liver cancer	2019	2.19	1.64	2.82
Paraguay	Both	Pancreatic cancer	2019	6.26	4.77	7.87
Paraguay	Both	Stomach cancer	2019	8.72	6.76	11.08
Peru	Both	Colon and rectum cancer	2019	9.44	7.12	12.25
Peru	Both	Esophageal cancer	2019	1.38	1.03	1.80
Peru	Both	Liver cancer	2019	2.74	2.04	3.61
Peru	Both	Pancreatic cancer	2019	5.42	4.09	7.08
Peru	Both	Stomach cancer	2019	17.81	13.44	23.13
Philippines	Both	Colon and rectum cancer	2019	14.89	12.46	17.81
Philippines	Both	Esophageal cancer	2019	1.36	1.10	1.88
Philippines	Both	Liver cancer	2019	6.58	5.35	7.98

Philippines	Both	Pancreatic cancer	2019	4.41	3.64	5.32
Philippines	Both	Stomach cancer	2019	4.50	3.78	5.30
Poland	Both	Colon and rectum cancer	2019	24.72	20.98	28.96
Poland	Both	Esophageal cancer	2019	3.28	2.70	3.99
Poland	Both	Liver cancer	2019	2.06	1.73	2.44
Poland	Both	Pancreatic cancer	2019	10.58	8.88	12.52
Poland	Both	Stomach cancer	2019	9.66	8.10	11.35
Portugal	Both	Colon and rectum cancer	2019	20.11	18.52	21.52
Portugal	Both	Esophageal cancer	2019	3.17	2.86	3.50
Portugal	Both	Liver cancer	2019	4.59	4.19	4.97
Portugal	Both	Pancreatic cancer	2019	7.44	6.64	8.12
Portugal	Both	Stomach cancer	2019	11.95	10.94	12.82
Puerto Rico	Both	Colon and rectum cancer	2019	15.44	12.15	19.55
Puerto Rico	Both	Esophageal cancer	2019	2.50	1.92	3.22
Puerto Rico	Both	Liver cancer	2019	2.74	2.04	3.55
Puerto Rico	Both	Pancreatic cancer	2019	6.10	4.68	7.78
Puerto Rico	Both	Stomach cancer	2019	4.74	3.70	5.97
Qatar	Both	Colon and rectum cancer	2019	16.48	12.93	20.54
Qatar	Both	Esophageal cancer	2019	4.84	3.38	6.65
Qatar	Both	Liver cancer	2019	15.88	11.76	20.79
Qatar	Both	Pancreatic cancer	2019	8.07	6.09	10.50
Qatar	Both	Stomach cancer	2019	8.26	6.47	10.36
Republic of Korea	Both	Colon and rectum cancer	2019	13.87	12.22	15.30
Republic of Korea	Both	Esophageal cancer	2019	2.43	2.09	3.52
Republic of Korea	Both	Liver cancer	2019	16.20	14.47	17.94
Republic of Korea	Both	Pancreatic cancer	2019	8.25	7.34	9.20
Republic of Korea	Both	Stomach cancer	2019	14.09	12.57	15.60
Republic of Moldova	Both	Colon and rectum cancer	2019	17.66	15.45	20.01
Republic of Moldova	Both	Esophageal cancer	2019	1.78	1.50	2.11
Republic of Moldova	Both	Liver cancer	2019	2.40	2.03	2.81
Republic of Moldova	Both	Pancreatic cancer	2019	7.95	6.90	9.13
Republic of Moldova	Both	Stomach cancer	2019	9.32	8.04	10.66
Romania	Both	Colon and rectum cancer	2019	20.18	16.61	24.11
Romania	Both	Esophageal cancer	2019	2.52	2.02	3.06
Romania	Both	Liver cancer	2019	3.08	2.50	3.75
Romania	Both	Pancreatic cancer	2019	9.92	8.11	12.17
Romania	Both	Stomach cancer	2019	10.46	8.56	12.70
Russian Federation	Both	Colon and rectum cancer	2019	18.17	15.96	20.54
Russian Federation	Both	Esophageal cancer	2019	3.24	2.73	3.79
Russian Federation	Both	Liver cancer	2019	2.97	2.53	3.55
Russian Federation	Both	Pancreatic cancer	2019	8.23	7.20	9.34
Russian Federation	Both	Stomach cancer	2019	13.22	11.53	15.08
Rwanda	Both	Colon and rectum cancer	2019	8.79	7.08	10.83
Rwanda	Both	Esophageal cancer	2019	11.50	7.66	16.11
Rwanda	Both	Liver cancer	2019	4.72	3.78	5.98
Rwanda	Both	Pancreatic cancer	2019	3.62	2.90	4.58
Rwanda	Both	Stomach cancer	2019	7.36	5.87	9.03
Saint Kitts and Nevis	Both	Colon and rectum cancer	2019	18.32	15.84	21.11
Saint Kitts and Nevis	Both	Esophageal cancer	2019	4.36	3.67	5.15
Saint Kitts and Nevis	Both	Liver cancer	2019	3.88	3.28	4.57
Saint Kitts and Nevis	Both	Pancreatic cancer	2019	9.49	7.97	11.22
Saint Kitts and Nevis	Both	Stomach cancer	2019	9.99	8.52	11.56
Saint Lucia	Both	Colon and rectum cancer	2019	12.08	10.24	14.11
Saint Lucia	Both	Esophageal cancer	2019	4.47	3.70	5.31
Saint Lucia	Both	Liver cancer	2019	2.29	1.91	2.73
Saint Lucia	Both	Pancreatic cancer	2019	7.73	6.46	9.11
Saint Lucia	Both	Stomach cancer	2019	12.05	10.18	14.05
Saint Vincent and the Grenadines	Both	Colon and rectum cancer	2019	14.42	12.69	16.48
Saint Vincent and the Grenadines	Both	Esophageal cancer	2019	2.48	2.13	2.87
Saint Vincent and the Grenadines	Both	Liver cancer	2019	3.13	2.72	3.61
Saint Vincent and the Grenadines	Both	Pancreatic cancer	2019	6.50	5.64	7.45
Saint Vincent and the Grenadines	Both	Stomach cancer	2019	11.41	10.02	13.14
Samoa	Both	Colon and rectum cancer	2019	10.06	8.18	12.34
Samoa	Both	Esophageal cancer	2019	1.68	1.29	2.08
Samoa	Both	Liver cancer	2019	5.06	3.91	6.33

Samoa	Both	Pancreatic cancer	2019	4.40	3.57	5.59
Samoa	Both	Stomach cancer	2019	12.40	10.02	15.31
San Marino	Both	Colon and rectum cancer	2019	21.24	14.31	29.61
San Marino	Both	Esophageal cancer	2019	1.47	0.97	2.09
San Marino	Both	Liver cancer	2019	2.75	1.80	3.98
San Marino	Both	Pancreatic cancer	2019	11.10	7.38	16.00
San Marino	Both	Stomach cancer	2019	18.55	12.51	25.86
Sao Tome and Principe	Both	Colon and rectum cancer	2019	15.18	11.24	20.55
Sao Tome and Principe	Both	Esophageal cancer	2019	5.13	2.77	6.43
Sao Tome and Principe	Both	Liver cancer	2019	2.80	1.89	3.69
Sao Tome and Principe	Both	Pancreatic cancer	2019	3.13	2.09	4.31
Sao Tome and Principe	Both	Stomach cancer	2019	18.23	14.88	22.59
Saudi Arabia	Both	Colon and rectum cancer	2019	9.66	7.79	11.64
Saudi Arabia	Both	Esophageal cancer	2019	1.78	1.40	2.36
Saudi Arabia	Both	Liver cancer	2019	4.90	3.86	6.25
Saudi Arabia	Both	Pancreatic cancer	2019	5.13	4.16	6.24
Saudi Arabia	Both	Stomach cancer	2019	4.10	3.36	4.95
Senegal	Both	Colon and rectum cancer	2019	8.70	7.13	10.77
Senegal	Both	Esophageal cancer	2019	5.01	2.78	6.60
Senegal	Both	Liver cancer	2019	2.21	1.65	2.78
Senegal	Both	Pancreatic cancer	2019	4.87	3.98	5.98
Senegal	Both	Stomach cancer	2019	14.01	11.18	17.10
Serbia	Both	Colon and rectum cancer	2019	25.38	20.63	31.01
Serbia	Both	Esophageal cancer	2019	2.45	1.91	3.14
Serbia	Both	Liver cancer	2019	5.49	4.36	6.87
Serbia	Both	Pancreatic cancer	2019	10.80	8.55	13.44
Serbia	Both	Stomach cancer	2019	8.70	6.95	10.83
Seychelles	Both	Colon and rectum cancer	2019	25.33	22.17	28.67
Seychelles	Both	Esophageal cancer	2019	5.61	4.76	6.66
Seychelles	Both	Liver cancer	2019	5.70	4.81	6.72
Seychelles	Both	Pancreatic cancer	2019	7.50	6.54	8.61
Seychelles	Both	Stomach cancer	2019	6.85	5.96	7.82
Sierra Leone	Both	Colon and rectum cancer	2019	6.96	5.44	8.86
Sierra Leone	Both	Esophageal cancer	2019	4.60	2.51	6.21
Sierra Leone	Both	Liver cancer	2019	4.94	3.79	6.39
Sierra Leone	Both	Pancreatic cancer	2019	3.74	2.97	4.72
Sierra Leone	Both	Stomach cancer	2019	13.68	10.73	17.25
Singapore	Both	Colon and rectum cancer	2019	14.89	13.22	16.12
Singapore	Both	Esophageal cancer	2019	1.81	1.57	2.10
Singapore	Both	Liver cancer	2019	8.68	7.67	9.66
Singapore	Both	Pancreatic cancer	2019	5.59	4.97	6.14
Singapore	Both	Stomach cancer	2019	5.54	4.87	6.05
Slovakia	Both	Colon and rectum cancer	2019	26.31	20.96	32.80
Slovakia	Both	Esophageal cancer	2019	3.19	2.29	4.22
Slovakia	Both	Liver cancer	2019	3.39	2.64	4.28
Slovakia	Both	Pancreatic cancer	2019	10.42	8.17	13.03
Slovakia	Both	Stomach cancer	2019	8.32	6.61	10.31
Slovenia	Both	Colon and rectum cancer	2019	19.84	15.58	25.45
Slovenia	Both	Esophageal cancer	2019	2.54	1.91	3.33
Slovenia	Both	Liver cancer	2019	5.14	3.93	6.61
Slovenia	Both	Pancreatic cancer	2019	9.46	7.43	12.24
Slovenia	Both	Stomach cancer	2019	8.54	6.71	10.95
Solomon Islands	Both	Colon and rectum cancer	2019	10.43	7.38	13.29
Solomon Islands	Both	Esophageal cancer	2019	3.48	2.57	4.78
Solomon Islands	Both	Liver cancer	2019	5.52	4.46	6.68
Solomon Islands	Both	Pancreatic cancer	2019	3.07	2.18	4.03
Solomon Islands	Both	Stomach cancer	2019	23.87	18.94	29.03
Somalia	Both	Colon and rectum cancer	2019	5.01	3.17	9.27
Somalia	Both	Esophageal cancer	2019	13.21	8.48	18.69
Somalia	Both	Liver cancer	2019	3.61	2.35	6.40
Somalia	Both	Pancreatic cancer	2019	1.69	0.92	2.72
Somalia	Both	Stomach cancer	2019	10.62	7.89	13.96
South Africa	Both	Colon and rectum cancer	2019	11.21	10.13	12.60
South Africa	Both	Esophageal cancer	2019	10.57	9.46	12.54
South Africa	Both	Liver cancer	2019	5.87	5.18	6.68

South Africa	Both	Pancreatic cancer	2019	6.29	5.64	7.04
South Africa	Both	Stomach cancer	2019	5.70	5.32	6.09
South Sudan	Both	Colon and rectum cancer	2019	9.99	6.57	14.78
South Sudan	Both	Esophageal cancer	2019	10.79	7.13	15.50
South Sudan	Both	Liver cancer	2019	2.97	1.79	5.14
South Sudan	Both	Pancreatic cancer	2019	3.00	2.15	4.17
South Sudan	Both	Stomach cancer	2019	7.30	5.31	9.89
Spain	Both	Colon and rectum cancer	2019	18.85	17.05	20.21
Spain	Both	Esophageal cancer	2019	2.55	2.32	2.81
Spain	Both	Liver cancer	2019	5.18	4.67	5.67
Spain	Both	Pancreatic cancer	2019	8.04	7.25	8.80
Spain	Both	Stomach cancer	2019	7.02	6.39	7.61
Sri Lanka	Both	Colon and rectum cancer	2019	6.41	4.81	8.26
Sri Lanka	Both	Esophageal cancer	2019	4.49	3.32	6.01
Sri Lanka	Both	Liver cancer	2019	2.84	2.11	3.77
Sri Lanka	Both	Pancreatic cancer	2019	2.98	2.25	3.91
Sri Lanka	Both	Stomach cancer	2019	5.04	3.87	6.55
Sudan	Both	Colon and rectum cancer	2019	7.10	5.26	10.59
Sudan	Both	Esophageal cancer	2019	5.04	1.58	7.21
Sudan	Both	Liver cancer	2019	3.92	2.41	6.04
Sudan	Both	Pancreatic cancer	2019	3.76	2.60	5.77
Sudan	Both	Stomach cancer	2019	15.63	10.93	20.20
Suriname	Both	Colon and rectum cancer	2019	16.26	13.57	19.24
Suriname	Both	Esophageal cancer	2019	1.44	1.16	1.75
Suriname	Both	Liver cancer	2019	2.74	2.21	3.38
Suriname	Both	Pancreatic cancer	2019	7.27	5.99	8.70
Suriname	Both	Stomach cancer	2019	7.28	6.09	8.70
Sweden	Both	Colon and rectum cancer	2019	15.96	14.67	16.96
Sweden	Both	Esophageal cancer	2019	2.71	2.50	2.92
Sweden	Both	Liver cancer	2019	3.02	2.80	3.24
Sweden	Both	Pancreatic cancer	2019	9.04	8.31	9.69
Sweden	Both	Stomach cancer	2019	3.67	3.34	3.93
Switzerland	Both	Colon and rectum cancer	2019	11.85	10.70	12.84
Switzerland	Both	Esophageal cancer	2019	3.31	2.98	3.68
Switzerland	Both	Liver cancer	2019	4.43	3.97	4.91
Switzerland	Both	Pancreatic cancer	2019	8.73	7.82	9.59
Switzerland	Both	Stomach cancer	2019	4.03	3.63	4.38
Syrian Arab Republic	Both	Colon and rectum cancer	2019	6.28	4.70	8.15
Syrian Arab Republic	Both	Esophageal cancer	2019	0.96	0.73	1.23
Syrian Arab Republic	Both	Liver cancer	2019	4.41	3.35	5.77
Syrian Arab Republic	Both	Pancreatic cancer	2019	3.75	2.82	5.01
Syrian Arab Republic	Both	Stomach cancer	2019	4.97	3.82	6.47
Taiwan (Province of China)	Both	Colon and rectum cancer	2019	26.26	20.88	33.29
Taiwan (Province of China)	Both	Esophageal cancer	2019	7.89	6.02	10.38
Taiwan (Province of China)	Both	Liver cancer	2019	7.27	5.69	9.32
Taiwan (Province of China)	Both	Pancreatic cancer	2019	8.53	6.64	11.08
Taiwan (Province of China)	Both	Stomach cancer	2019	10.72	8.42	13.73
Tajikistan	Both	Colon and rectum cancer	2019	10.22	8.41	12.35
Tajikistan	Both	Esophageal cancer	2019	7.72	5.69	16.63
Tajikistan	Both	Liver cancer	2019	4.02	3.23	5.06
Tajikistan	Both	Pancreatic cancer	2019	5.62	4.68	6.87
Tajikistan	Both	Stomach cancer	2019	25.26	20.77	30.44
Thailand	Both	Colon and rectum cancer	2019	10.54	7.88	13.56
Thailand	Both	Esophageal cancer	2019	3.00	1.95	4.03
Thailand	Both	Liver cancer	2019	24.01	17.88	31.65
Thailand	Both	Pancreatic cancer	2019	4.11	3.07	5.30
Thailand	Both	Stomach cancer	2019	5.22	3.93	6.85
Timor-Leste	Both	Colon and rectum cancer	2019	12.29	8.48	15.79
Timor-Leste	Both	Esophageal cancer	2019	2.48	1.82	3.60
Timor-Leste	Both	Liver cancer	2019	6.60	4.53	9.09
Timor-Leste	Both	Pancreatic cancer	2019	3.07	2.20	3.96
Timor-Leste	Both	Stomach cancer	2019	7.85	5.83	9.60
Togo	Both	Colon and rectum cancer	2019	7.88	5.90	10.04
Togo	Both	Esophageal cancer	2019	4.92	2.69	6.63
Togo	Both	Liver cancer	2019	5.24	4.09	6.74

Togo	Both	Pancreatic cancer	2019	4.92	3.64	6.54
Togo	Both	Stomach cancer	2019	14.17	11.52	17.72
Tokelau	Both	Colon and rectum cancer	2019	10.85	8.14	13.95
Tokelau	Both	Esophageal cancer	2019	2.02	1.54	2.57
Tokelau	Both	Liver cancer	2019	7.48	5.46	10.06
Tokelau	Both	Pancreatic cancer	2019	4.46	3.15	5.76
Tokelau	Both	Stomach cancer	2019	9.86	8.00	12.28
Tonga	Both	Colon and rectum cancer	2019	6.91	5.40	8.68
Tonga	Both	Esophageal cancer	2019	2.21	1.67	2.86
Tonga	Both	Liver cancer	2019	24.74	18.09	32.04
Tonga	Both	Pancreatic cancer	2019	4.49	3.29	5.87
Tonga	Both	Stomach cancer	2019	13.85	11.50	16.52
Trinidad and Tobago	Both	Colon and rectum cancer	2019	13.97	10.74	17.96
Trinidad and Tobago	Both	Esophageal cancer	2019	1.58	1.17	2.07
Trinidad and Tobago	Both	Liver cancer	2019	2.66	2.03	3.46
Trinidad and Tobago	Both	Pancreatic cancer	2019	6.00	4.56	7.78
Trinidad and Tobago	Both	Stomach cancer	2019	4.54	3.46	5.81
Tunisia	Both	Colon and rectum cancer	2019	9.73	7.17	12.98
Tunisia	Both	Esophageal cancer	2019	0.97	0.68	1.31
Tunisia	Both	Liver cancer	2019	1.80	1.27	2.53
Tunisia	Both	Pancreatic cancer	2019	3.86	2.88	5.07
Tunisia	Both	Stomach cancer	2019	5.19	3.89	6.94
Turkey	Both	Colon and rectum cancer	2019	13.11	10.62	15.83
Turkey	Both	Esophageal cancer	2019	1.52	1.18	1.93
Turkey	Both	Liver cancer	2019	2.96	2.36	3.63
Turkey	Both	Pancreatic cancer	2019	8.24	6.62	10.08
Turkey	Both	Stomach cancer	2019	10.75	8.59	13.18
Turkmenistan	Both	Colon and rectum cancer	2019	7.08	5.81	8.71
Turkmenistan	Both	Esophageal cancer	2019	9.97	7.94	12.44
Turkmenistan	Both	Liver cancer	2019	5.59	4.41	7.07
Turkmenistan	Both	Pancreatic cancer	2019	3.86	3.12	4.80
Turkmenistan	Both	Stomach cancer	2019	9.23	7.35	11.45
Tuvalu	Both	Colon and rectum cancer	2019	10.90	8.08	13.99
Tuvalu	Both	Esophageal cancer	2019	2.45	1.80	3.30
Tuvalu	Both	Liver cancer	2019	8.72	6.51	11.52
Tuvalu	Both	Pancreatic cancer	2019	4.02	2.98	5.37
Tuvalu	Both	Stomach cancer	2019	13.67	10.71	17.41
Uganda	Both	Colon and rectum cancer	2019	11.55	9.24	13.75
Uganda	Both	Esophageal cancer	2019	16.53	12.84	20.60
Uganda	Both	Liver cancer	2019	6.39	5.12	7.88
Uganda	Both	Pancreatic cancer	2019	5.00	3.95	6.21
Uganda	Both	Stomach cancer	2019	8.64	7.02	10.41
Ukraine	Both	Colon and rectum cancer	2019	19.18	16.46	22.37
Ukraine	Both	Esophageal cancer	2019	2.78	2.20	3.42
Ukraine	Both	Liver cancer	2019	2.66	2.24	3.11
Ukraine	Both	Pancreatic cancer	2019	8.03	6.77	9.42
Ukraine	Both	Stomach cancer	2019	13.46	11.41	15.82
United Arab Emirates	Both	Colon and rectum cancer	2019	17.40	11.94	24.35
United Arab Emirates	Both	Esophageal cancer	2019	8.42	2.68	14.24
United Arab Emirates	Both	Liver cancer	2019	4.90	2.06	11.30
United Arab Emirates	Both	Pancreatic cancer	2019	17.57	9.45	26.17
United Arab Emirates	Both	Stomach cancer	2019	9.23	7.31	11.38
United Kingdom	Both	Colon and rectum cancer	2019	18.09	16.77	18.85
United Kingdom	Both	Esophageal cancer	2019	7.77	7.31	8.09
United Kingdom	Both	Liver cancer	2019	4.03	3.75	4.26
United Kingdom	Both	Pancreatic cancer	2019	9.22	8.58	9.66
United Kingdom	Both	Stomach cancer	2019	5.62	5.19	5.88
United Republic of Tanzania	Both	Colon and rectum cancer	2019	9.51	7.74	11.82
United Republic of Tanzania	Both	Esophageal cancer	2019	12.56	8.13	17.39
United Republic of Tanzania	Both	Liver cancer	2019	2.46	1.98	3.02
United Republic of Tanzania	Both	Pancreatic cancer	2019	3.87	3.09	4.90
United Republic of Tanzania	Both	Stomach cancer	2019	7.68	6.29	9.16
United States of America	Both	Colon and rectum cancer	2019	14.77	13.86	15.32
United States of America	Both	Esophageal cancer	2019	3.86	3.69	4.02
United States of America	Both	Liver cancer	2019	4.33	3.86	4.75

United States of America	Both	Pancreatic cancer	2019	10.06	9.43	10.52
United States of America	Both	Stomach cancer	2019	3.40	3.19	3.54
United States Virgin Islands	Both	Colon and rectum cancer	2019	26.57	22.30	31.01
United States Virgin Islands	Both	Esophageal cancer	2019	4.27	3.48	5.03
United States Virgin Islands	Both	Liver cancer	2019	2.60	2.17	3.07
United States Virgin Islands	Both	Pancreatic cancer	2019	11.30	9.28	13.44
United States Virgin Islands	Both	Stomach cancer	2019	9.79	8.29	11.34
Uruguay	Both	Colon and rectum cancer	2019	26.12	24.14	27.82
Uruguay	Both	Esophageal cancer	2019	6.22	5.63	6.93
Uruguay	Both	Liver cancer	2019	2.36	2.12	2.61
Uruguay	Both	Pancreatic cancer	2019	14.47	13.22	15.74
Uruguay	Both	Stomach cancer	2019	10.66	9.78	11.49
Uzbekistan	Both	Colon and rectum cancer	2019	10.35	9.07	11.79
Uzbekistan	Both	Esophageal cancer	2019	6.98	5.88	8.09
Uzbekistan	Both	Liver cancer	2019	6.68	5.58	7.83
Uzbekistan	Both	Pancreatic cancer	2019	5.18	4.41	6.03
Uzbekistan	Both	Stomach cancer	2019	13.43	11.44	15.44
Vanuatu	Both	Colon and rectum cancer	2019	9.29	7.09	11.92
Vanuatu	Both	Esophageal cancer	2019	2.75	2.02	3.80
Vanuatu	Both	Liver cancer	2019	9.36	6.41	13.20
Vanuatu	Both	Pancreatic cancer	2019	3.11	2.42	4.01
Vanuatu	Both	Stomach cancer	2019	15.75	11.67	20.34
Venezuela (Bolivarian Republic of)	Both	Colon and rectum cancer	2019	11.31	8.72	14.59
Venezuela (Bolivarian Republic of)	Both	Esophageal cancer	2019	2.05	1.50	2.67
Venezuela (Bolivarian Republic of)	Both	Liver cancer	2019	2.44	1.88	3.15
Venezuela (Bolivarian Republic of)	Both	Pancreatic cancer	2019	5.86	4.45	7.55
Venezuela (Bolivarian Republic of)	Both	Stomach cancer	2019	12.58	9.63	16.01
Viet Nam	Both	Colon and rectum cancer	2019	17.50	13.92	21.05
Viet Nam	Both	Esophageal cancer	2019	2.87	2.07	3.58
Viet Nam	Both	Liver cancer	2019	2.75	2.15	3.40
Viet Nam	Both	Pancreatic cancer	2019	4.53	3.57	5.68
Viet Nam	Both	Stomach cancer	2019	9.98	8.14	11.84
Yemen	Both	Colon and rectum cancer	2019	6.66	5.01	9.07
Yemen	Both	Esophageal cancer	2019	4.45	1.36	6.78
Yemen	Both	Liver cancer	2019	3.42	2.49	4.57
Yemen	Both	Pancreatic cancer	2019	2.23	1.70	2.93
Yemen	Both	Stomach cancer	2019	20.20	15.54	26.28
Zambia	Both	Colon and rectum cancer	2019	12.62	9.51	15.96
Zambia	Both	Esophageal cancer	2019	14.83	9.78	19.76
Zambia	Both	Liver cancer	2019	2.99	2.37	3.67
Zambia	Both	Pancreatic cancer	2019	5.09	3.71	6.95
Zambia	Both	Stomach cancer	2019	8.68	6.91	10.64
Zimbabwe	Both	Colon and rectum cancer	2019	12.93	9.93	16.20
Zimbabwe	Both	Esophageal cancer	2019	16.02	12.75	19.64
Zimbabwe	Both	Liver cancer	2019	14.03	10.58	18.67
Zimbabwe	Both	Pancreatic cancer	2019	8.06	6.24	10.23
Zimbabwe	Both	Stomach cancer	2019	13.73	10.81	17.17

sex_name	cause_name	year	Age-standardised incidence rate (per 100 000 person-years)	95% CI (lower)	95% CI (upper)
Male	Colon and rectum cancer	1990	25.19	24.24	26.11
Female	Colon and rectum cancer	1990	19.92	18.83	20.81
Both	Colon and rectum cancer	1990	22.25	21.29	22.97
Male	Colon and rectum cancer	1991	25.47	24.51	26.41
Female	Colon and rectum cancer	1991	20.03	18.95	20.94
Both	Colon and rectum cancer	1991	22.44	21.49	23.16
Male	Colon and rectum cancer	1992	25.85	24.91	26.70
Female	Colon and rectum cancer	1992	20.15	19.01	21.02
Both	Colon and rectum cancer	1992	22.69	21.70	23.41
Male	Colon and rectum cancer	1993	26.51	25.53	27.27
Female	Colon and rectum cancer	1993	20.60	19.45	21.39
Both	Colon and rectum cancer	1993	23.24	22.23	23.87
Male	Colon and rectum cancer	1994	26.70	25.77	27.47
Female	Colon and rectum cancer	1994	20.63	19.50	21.39
Both	Colon and rectum cancer	1994	23.35	22.41	24.01
Male	Colon and rectum cancer	1995	26.94	26.04	27.65
Female	Colon and rectum cancer	1995	20.63	19.46	21.33
Both	Colon and rectum cancer	1995	23.47	22.47	24.10
Male	Colon and rectum cancer	1996	26.92	26.03	27.64
Female	Colon and rectum cancer	1996	20.54	19.34	21.27
Both	Colon and rectum cancer	1996	23.42	22.41	24.03
Male	Colon and rectum cancer	1997	26.96	26.03	27.68
Female	Colon and rectum cancer	1997	20.53	19.29	21.27
Both	Colon and rectum cancer	1997	23.44	22.43	24.07
Male	Colon and rectum cancer	1998	27.34	26.44	28.04
Female	Colon and rectum cancer	1998	20.68	19.49	21.46
Both	Colon and rectum cancer	1998	23.69	22.66	24.34
Male	Colon and rectum cancer	1999	27.80	26.87	28.58
Female	Colon and rectum cancer	1999	20.99	19.73	21.75
Both	Colon and rectum cancer	1999	24.08	22.99	24.72
Male	Colon and rectum cancer	2000	28.02	27.08	28.79
Female	Colon and rectum cancer	2000	21.10	19.82	21.90
Both	Colon and rectum cancer	2000	24.26	23.18	24.97
Male	Colon and rectum cancer	2001	28.30	27.16	29.08
Female	Colon and rectum cancer	2001	21.16	19.82	22.01
Both	Colon and rectum cancer	2001	24.42	23.15	25.16
Male	Colon and rectum cancer	2002	28.85	27.78	29.76
Female	Colon and rectum cancer	2002	21.36	19.99	22.21
Both	Colon and rectum cancer	2002	24.79	23.61	25.57
Male	Colon and rectum cancer	2003	29.50	28.35	30.47
Female	Colon and rectum cancer	2003	21.55	20.27	22.43
Both	Colon and rectum cancer	2003	25.21	23.96	26.03
Male	Colon and rectum cancer	2004	29.76	28.54	30.86
Female	Colon and rectum cancer	2004	21.39	19.90	22.30
Both	Colon and rectum cancer	2004	25.24	23.91	26.12
Male	Colon and rectum cancer	2005	30.26	29.05	31.31
Female	Colon and rectum cancer	2005	21.44	19.95	22.35
Both	Colon and rectum cancer	2005	25.50	24.08	26.36

Male	Colon and rectum cancer	2006	30.16	28.92	31.19
Female	Colon and rectum cancer	2006	21.20	19.67	22.15
Both	Colon and rectum cancer	2006	25.33	23.94	26.20
Male	Colon and rectum cancer	2007	30.53	29.24	31.64
Female	Colon and rectum cancer	2007	21.21	19.71	22.15
Both	Colon and rectum cancer	2007	25.51	24.15	26.43
Male	Colon and rectum cancer	2008	31.10	29.77	32.23
Female	Colon and rectum cancer	2008	21.35	19.86	22.35
Both	Colon and rectum cancer	2008	25.85	24.41	26.81
Male	Colon and rectum cancer	2009	31.50	30.07	32.63
Female	Colon and rectum cancer	2009	21.31	19.80	22.24
Both	Colon and rectum cancer	2009	26.01	24.52	26.94
Male	Colon and rectum cancer	2010	31.85	30.34	33.25
Female	Colon and rectum cancer	2010	21.20	19.64	22.20
Both	Colon and rectum cancer	2010	26.12	24.71	27.13
Male	Colon and rectum cancer	2011	31.89	30.35	33.22
Female	Colon and rectum cancer	2011	21.12	19.56	22.13
Both	Colon and rectum cancer	2011	26.10	24.57	27.13
Male	Colon and rectum cancer	2012	31.92	30.30	33.29
Female	Colon and rectum cancer	2012	20.87	19.32	21.85
Both	Colon and rectum cancer	2012	25.98	24.53	27.00
Male	Colon and rectum cancer	2013	31.86	30.18	33.50
Female	Colon and rectum cancer	2013	20.73	19.18	21.76
Both	Colon and rectum cancer	2013	25.88	24.35	27.00
Male	Colon and rectum cancer	2014	31.81	30.06	33.32
Female	Colon and rectum cancer	2014	20.67	19.09	21.70
Both	Colon and rectum cancer	2014	25.82	24.32	26.86
Male	Colon and rectum cancer	2015	32.02	30.21	33.66
Female	Colon and rectum cancer	2015	20.72	19.18	21.84
Both	Colon and rectum cancer	2015	25.95	24.35	27.07
Male	Colon and rectum cancer	2016	31.98	30.11	33.79
Female	Colon and rectum cancer	2016	20.69	19.08	21.87
Both	Colon and rectum cancer	2016	25.91	24.28	27.13
Male	Colon and rectum cancer	2017	32.14	29.96	34.36
Female	Colon and rectum cancer	2017	20.71	18.89	22.25
Both	Colon and rectum cancer	2017	26.00	24.20	27.57
Male	Colon and rectum cancer	2018	32.66	29.98	35.23
Female	Colon and rectum cancer	2018	20.99	19.02	22.74
Both	Colon and rectum cancer	2018	26.39	24.52	28.21
Male	Colon and rectum cancer	2019	33.06	30.22	36.15
Female	Colon and rectum cancer	2019	21.22	19.05	23.16
Both	Colon and rectum cancer	2019	26.71	24.58	28.89

location_name	sex_name	cause_name	year	Age-standardised incidence rate (per 100 000 person-years)	95% CI (lower)	95% CI (upper)
High SDI	Male	Colon and rectum cancer	1990	52.12	50.63	53.10
High SDI	Female	Colon and rectum cancer	1990	35.39	33.72	36.39
High SDI	Both	Colon and rectum cancer	1990	42.45	40.91	43.35
High-middle SDI	Male	Colon and rectum cancer	1990	26.35	25.21	27.43
High-middle SDI	Female	Colon and rectum cancer	1990	20.08	19.10	21.08
High-middle SDI	Both	Colon and rectum cancer	1990	22.60	21.75	23.36
Low SDI	Male	Colon and rectum cancer	1990	6.71	5.51	8.38
Low SDI	Female	Colon and rectum cancer	1990	5.67	4.35	7.24
Low SDI	Both	Colon and rectum cancer	1990	6.20	5.19	7.26
Low-middle SDI	Male	Colon and rectum cancer	1990	6.94	6.17	8.44
Low-middle SDI	Female	Colon and rectum cancer	1990	6.81	5.88	7.90
Low-middle SDI	Both	Colon and rectum cancer	1990	6.88	6.23	7.71
Middle SDI	Male	Colon and rectum cancer	1990	10.89	9.85	12.03
Middle SDI	Female	Colon and rectum cancer	1990	9.63	8.74	10.61
Middle SDI	Both	Colon and rectum cancer	1990	10.23	9.50	11.03
High SDI	Male	Colon and rectum cancer	1991	52.80	51.25	53.82
High SDI	Female	Colon and rectum cancer	1991	35.69	33.95	36.66
High SDI	Both	Colon and rectum cancer	1991	42.94	41.30	43.85
High-middle SDI	Male	Colon and rectum cancer	1991	26.87	25.75	27.93
High-middle SDI	Female	Colon and rectum cancer	1991	20.35	19.43	21.30
High-middle SDI	Both	Colon and rectum cancer	1991	22.98	22.14	23.78
Low SDI	Male	Colon and rectum cancer	1991	5.90	4.85	7.52
Low SDI	Female	Colon and rectum cancer	1991	5.65	4.36	7.22
Low SDI	Both	Colon and rectum cancer	1991	5.78	4.83	6.79
Low-middle SDI	Male	Colon and rectum cancer	1991	6.89	6.14	8.39
Low-middle SDI	Female	Colon and rectum cancer	1991	6.81	5.84	7.88
Low-middle SDI	Both	Colon and rectum cancer	1991	6.85	6.22	7.64
Middle SDI	Male	Colon and rectum cancer	1991	9.36	8.56	10.24
Middle SDI	Female	Colon and rectum cancer	1991	9.72	8.85	10.74
Middle SDI	Both	Colon and rectum cancer	1991	9.55	8.90	10.25
High SDI	Male	Colon and rectum cancer	1992	53.71	52.03	54.74
High SDI	Female	Colon and rectum cancer	1992	35.94	34.15	36.92
High SDI	Both	Colon and rectum cancer	1992	43.51	41.83	44.45
High-middle SDI	Male	Colon and rectum cancer	1992	27.55	26.50	28.61
High-middle SDI	Female	Colon and rectum cancer	1992	20.58	19.71	21.42
High-middle SDI	Both	Colon and rectum cancer	1992	23.42	22.56	24.15
Low SDI	Male	Colon and rectum cancer	1992	5.87	4.86	7.48
Low SDI	Female	Colon and rectum cancer	1992	5.65	4.38	7.18
Low SDI	Both	Colon and rectum cancer	1992	5.76	4.86	6.76
Low-middle SDI	Male	Colon and rectum cancer	1992	6.93	6.19	8.39
Low-middle SDI	Female	Colon and rectum cancer	1992	6.87	5.93	7.99
Low-middle SDI	Both	Colon and rectum cancer	1992	6.91	6.23	7.70
Middle SDI	Male	Colon and rectum cancer	1992	9.62	8.86	10.39
Middle SDI	Female	Colon and rectum cancer	1992	9.86	9.05	10.86
Middle SDI	Both	Colon and rectum cancer	1992	9.74	9.13	10.48
High SDI	Male	Colon and rectum cancer	1993	55.21	53.58	56.24
High SDI	Female	Colon and rectum cancer	1993	36.81	34.90	37.81
High SDI	Both	Colon and rectum cancer	1993	44.69	42.89	45.63
High-middle SDI	Male	Colon and rectum cancer	1993	28.49	27.36	29.51
High-middle SDI	Female	Colon and rectum cancer	1993	21.33	20.36	22.13
High-middle SDI	Both	Colon and rectum cancer	1993	24.26	23.38	24.93
Low SDI	Male	Colon and rectum cancer	1993	5.87	4.88	7.47
Low SDI	Female	Colon and rectum cancer	1993	5.63	4.42	7.08
Low SDI	Both	Colon and rectum cancer	1993	5.76	4.89	6.70

Low-middle SDI	Male	Colon and rectum cancer	1993		7.08	6.37	8.46
Low-middle SDI	Female	Colon and rectum cancer	1993		6.95	6.08	7.99
Low-middle SDI	Both	Colon and rectum cancer	1993		7.02	6.41	7.76
Middle SDI	Male	Colon and rectum cancer	1993		9.75	9.05	10.46
Middle SDI	Female	Colon and rectum cancer	1993		10.02	9.16	11.07
Middle SDI	Both	Colon and rectum cancer	1993		9.89	9.24	10.57
High SDI	Male	Colon and rectum cancer	1994		55.45	53.76	56.51
High SDI	Female	Colon and rectum cancer	1994		36.81	34.84	37.84
High SDI	Both	Colon and rectum cancer	1994		44.83	42.99	45.80
High-middle SDI	Male	Colon and rectum cancer	1994		29.06	28.07	30.16
High-middle SDI	Female	Colon and rectum cancer	1994		21.57	20.64	22.35
High-middle SDI	Both	Colon and rectum cancer	1994		24.64	23.83	25.36
Low SDI	Male	Colon and rectum cancer	1994		5.87	4.96	7.49
Low SDI	Female	Colon and rectum cancer	1994		5.62	4.48	6.99
Low SDI	Both	Colon and rectum cancer	1994		5.75	4.90	6.66
Low-middle SDI	Male	Colon and rectum cancer	1994		7.20	6.55	8.49
Low-middle SDI	Female	Colon and rectum cancer	1994		7.04	6.20	7.98
Low-middle SDI	Both	Colon and rectum cancer	1994		7.12	6.51	7.84
Middle SDI	Male	Colon and rectum cancer	1994		10.03	9.40	10.71
Middle SDI	Female	Colon and rectum cancer	1994		10.08	9.30	11.04
Middle SDI	Both	Colon and rectum cancer	1994		10.07	9.51	10.67
High SDI	Male	Colon and rectum cancer	1995		56.10	54.35	57.22
High SDI	Female	Colon and rectum cancer	1995		37.08	34.97	38.14
High SDI	Both	Colon and rectum cancer	1995		45.30	43.41	46.28
High-middle SDI	Male	Colon and rectum cancer	1995		29.26	28.28	30.24
High-middle SDI	Female	Colon and rectum cancer	1995		21.41	20.49	22.14
High-middle SDI	Both	Colon and rectum cancer	1995		24.64	23.81	25.29
Low SDI	Male	Colon and rectum cancer	1995		5.86	4.97	7.49
Low SDI	Female	Colon and rectum cancer	1995		5.60	4.48	6.86
Low SDI	Both	Colon and rectum cancer	1995		5.74	4.95	6.61
Low-middle SDI	Male	Colon and rectum cancer	1995		7.26	6.62	8.47
Low-middle SDI	Female	Colon and rectum cancer	1995		7.10	6.30	7.97
Low-middle SDI	Both	Colon and rectum cancer	1995		7.18	6.62	7.87
Middle SDI	Male	Colon and rectum cancer	1995		10.26	9.65	10.94
Middle SDI	Female	Colon and rectum cancer	1995		10.16	9.45	11.06
Middle SDI	Both	Colon and rectum cancer	1995		10.22	9.67	10.83
High SDI	Male	Colon and rectum cancer	1996		55.69	53.89	56.80
High SDI	Female	Colon and rectum cancer	1996		36.83	34.75	37.89
High SDI	Both	Colon and rectum cancer	1996		45.01	43.06	46.02
High-middle SDI	Male	Colon and rectum cancer	1996		29.26	28.32	30.13
High-middle SDI	Female	Colon and rectum cancer	1996		21.23	20.25	21.96
High-middle SDI	Both	Colon and rectum cancer	1996		24.53	23.67	25.17
Low SDI	Male	Colon and rectum cancer	1996		5.87	5.01	7.45
Low SDI	Female	Colon and rectum cancer	1996		5.62	4.55	6.78
Low SDI	Both	Colon and rectum cancer	1996		5.75	4.99	6.63
Low-middle SDI	Male	Colon and rectum cancer	1996		7.41	6.79	8.61
Low-middle SDI	Female	Colon and rectum cancer	1996		7.24	6.46	8.09
Low-middle SDI	Both	Colon and rectum cancer	1996		7.33	6.77	7.98
Middle SDI	Male	Colon and rectum cancer	1996		10.55	9.94	11.21
Middle SDI	Female	Colon and rectum cancer	1996		10.40	9.64	11.30
Middle SDI	Both	Colon and rectum cancer	1996		10.48	9.90	11.13
High SDI	Male	Colon and rectum cancer	1997		55.37	53.61	56.47
High SDI	Female	Colon and rectum cancer	1997		36.77	34.62	37.87
High SDI	Both	Colon and rectum cancer	1997		44.88	42.83	45.88
High-middle SDI	Male	Colon and rectum cancer	1997		29.38	28.40	30.29
High-middle SDI	Female	Colon and rectum cancer	1997		21.17	20.13	21.83

High-middle SDI	Both	Colon and rectum cancer	1997		24.55	23.61	25.19
Low SDI	Male	Colon and rectum cancer	1997		5.93	5.12	7.48
Low SDI	Female	Colon and rectum cancer	1997		5.67	4.64	6.78
Low SDI	Both	Colon and rectum cancer	1997		5.81	5.06	6.70
Low-middle SDI	Male	Colon and rectum cancer	1997		7.70	7.04	8.86
Low-middle SDI	Female	Colon and rectum cancer	1997		7.44	6.64	8.34
Low-middle SDI	Both	Colon and rectum cancer	1997		7.57	6.98	8.21
Middle SDI	Male	Colon and rectum cancer	1997		10.86	10.25	11.57
Middle SDI	Female	Colon and rectum cancer	1997		10.58	9.81	11.43
Middle SDI	Both	Colon and rectum cancer	1997		10.72	10.11	11.34
High SDI	Male	Colon and rectum cancer	1998		56.01	54.18	57.12
High SDI	Female	Colon and rectum cancer	1998		37.10	34.96	38.23
High SDI	Both	Colon and rectum cancer	1998		45.38	43.31	46.40
High-middle SDI	Male	Colon and rectum cancer	1998		29.85	28.86	30.74
High-middle SDI	Female	Colon and rectum cancer	1998		21.31	20.33	22.08
High-middle SDI	Both	Colon and rectum cancer	1998		24.83	23.93	25.48
Low SDI	Male	Colon and rectum cancer	1998		5.96	5.17	7.56
Low SDI	Female	Colon and rectum cancer	1998		5.73	4.70	6.75
Low SDI	Both	Colon and rectum cancer	1998		5.85	5.10	6.74
Low-middle SDI	Male	Colon and rectum cancer	1998		7.86	7.26	8.96
Low-middle SDI	Female	Colon and rectum cancer	1998		7.53	6.72	8.37
Low-middle SDI	Both	Colon and rectum cancer	1998		7.70	7.16	8.33
Middle SDI	Male	Colon and rectum cancer	1998		11.21	10.57	11.89
Middle SDI	Female	Colon and rectum cancer	1998		10.76	9.99	11.62
Middle SDI	Both	Colon and rectum cancer	1998		10.98	10.38	11.67
High SDI	Male	Colon and rectum cancer	1999		56.58	54.74	57.67
High SDI	Female	Colon and rectum cancer	1999		37.70	35.43	38.87
High SDI	Both	Colon and rectum cancer	1999		46.00	43.87	47.03
High-middle SDI	Male	Colon and rectum cancer	1999		30.78	29.69	31.70
High-middle SDI	Female	Colon and rectum cancer	1999		21.81	20.88	22.55
High-middle SDI	Both	Colon and rectum cancer	1999		25.52	24.63	26.18
Low SDI	Male	Colon and rectum cancer	1999		5.92	5.14	7.41
Low SDI	Female	Colon and rectum cancer	1999		5.74	4.76	6.75
Low SDI	Both	Colon and rectum cancer	1999		5.83	5.07	6.74
Low-middle SDI	Male	Colon and rectum cancer	1999		7.88	7.29	8.92
Low-middle SDI	Female	Colon and rectum cancer	1999		7.56	6.73	8.36
Low-middle SDI	Both	Colon and rectum cancer	1999		7.72	7.17	8.32
Middle SDI	Male	Colon and rectum cancer	1999		11.57	10.85	12.38
Middle SDI	Female	Colon and rectum cancer	1999		10.96	10.14	11.84
Middle SDI	Both	Colon and rectum cancer	1999		11.26	10.58	11.96
High SDI	Male	Colon and rectum cancer	2000		56.33	54.52	57.46
High SDI	Female	Colon and rectum cancer	2000		37.89	35.67	39.07
High SDI	Both	Colon and rectum cancer	2000		46.03	43.94	47.11
High-middle SDI	Male	Colon and rectum cancer	2000		31.22	30.19	32.21
High-middle SDI	Female	Colon and rectum cancer	2000		21.91	20.91	22.70
High-middle SDI	Both	Colon and rectum cancer	2000		25.80	24.91	26.50
Low SDI	Male	Colon and rectum cancer	2000		5.96	5.14	7.45
Low SDI	Female	Colon and rectum cancer	2000		5.77	4.79	6.70
Low SDI	Both	Colon and rectum cancer	2000		5.87	5.11	6.76
Low-middle SDI	Male	Colon and rectum cancer	2000		8.07	7.48	9.10
Low-middle SDI	Female	Colon and rectum cancer	2000		7.64	6.84	8.49
Low-middle SDI	Both	Colon and rectum cancer	2000		7.86	7.29	8.45
Middle SDI	Male	Colon and rectum cancer	2000		12.04	11.31	12.85
Middle SDI	Female	Colon and rectum cancer	2000		11.21	10.35	12.11
Middle SDI	Both	Colon and rectum cancer	2000		11.62	10.94	12.32
High SDI	Male	Colon and rectum cancer	2001		56.11	54.18	57.31

High SDI	Female	Colon and rectum cancer	2001	37.86	35.53	39.05
High SDI	Both	Colon and rectum cancer	2001	45.95	43.77	47.08
High-middle SDI	Male	Colon and rectum cancer	2001	31.88	30.69	32.93
High-middle SDI	Female	Colon and rectum cancer	2001	22.07	20.90	22.91
High-middle SDI	Both	Colon and rectum cancer	2001	26.19	25.08	26.96
Low SDI	Male	Colon and rectum cancer	2001	6.00	5.22	7.51
Low SDI	Female	Colon and rectum cancer	2001	5.85	4.91	6.72
Low SDI	Both	Colon and rectum cancer	2001	5.93	5.19	6.86
Low-middle SDI	Male	Colon and rectum cancer	2001	8.31	7.68	9.29
Low-middle SDI	Female	Colon and rectum cancer	2001	7.80	7.03	8.62
Low-middle SDI	Both	Colon and rectum cancer	2001	8.05	7.50	8.64
Middle SDI	Male	Colon and rectum cancer	2001	12.55	11.79	13.50
Middle SDI	Female	Colon and rectum cancer	2001	11.48	10.57	12.41
Middle SDI	Both	Colon and rectum cancer	2001	12.02	11.25	12.77
High SDI	Male	Colon and rectum cancer	2002	56.25	54.35	57.43
High SDI	Female	Colon and rectum cancer	2002	37.92	35.62	39.16
High SDI	Both	Colon and rectum cancer	2002	46.08	43.84	47.21
High-middle SDI	Male	Colon and rectum cancer	2002	32.97	31.69	34.14
High-middle SDI	Female	Colon and rectum cancer	2002	22.49	21.40	23.36
High-middle SDI	Both	Colon and rectum cancer	2002	26.91	25.84	27.73
Low SDI	Male	Colon and rectum cancer	2002	6.06	5.27	7.58
Low SDI	Female	Colon and rectum cancer	2002	5.94	5.02	6.77
Low SDI	Both	Colon and rectum cancer	2002	6.00	5.30	6.96
Low-middle SDI	Male	Colon and rectum cancer	2002	8.52	7.90	9.47
Low-middle SDI	Female	Colon and rectum cancer	2002	7.96	7.20	8.73
Low-middle SDI	Both	Colon and rectum cancer	2002	8.24	7.68	8.82
Middle SDI	Male	Colon and rectum cancer	2002	13.42	12.56	14.44
Middle SDI	Female	Colon and rectum cancer	2002	11.84	10.90	12.85
Middle SDI	Both	Colon and rectum cancer	2002	12.62	11.84	13.40
High SDI	Male	Colon and rectum cancer	2003	56.50	54.40	57.71
High SDI	Female	Colon and rectum cancer	2003	37.92	35.53	39.16
High SDI	Both	Colon and rectum cancer	2003	46.23	43.92	47.40
High-middle SDI	Male	Colon and rectum cancer	2003	34.41	33.06	35.76
High-middle SDI	Female	Colon and rectum cancer	2003	23.09	22.00	23.96
High-middle SDI	Both	Colon and rectum cancer	2003	27.89	26.71	28.75
Low SDI	Male	Colon and rectum cancer	2003	6.13	5.35	7.60
Low SDI	Female	Colon and rectum cancer	2003	5.99	5.09	6.82
Low SDI	Both	Colon and rectum cancer	2003	6.06	5.37	7.05
Low-middle SDI	Male	Colon and rectum cancer	2003	8.67	8.06	9.53
Low-middle SDI	Female	Colon and rectum cancer	2003	7.95	7.27	8.65
Low-middle SDI	Both	Colon and rectum cancer	2003	8.31	7.81	8.89
Middle SDI	Male	Colon and rectum cancer	2003	14.11	13.22	15.23
Middle SDI	Female	Colon and rectum cancer	2003	12.25	11.24	13.28
Middle SDI	Both	Colon and rectum cancer	2003	13.17	12.37	13.98
High SDI	Male	Colon and rectum cancer	2004	55.60	53.52	56.85
High SDI	Female	Colon and rectum cancer	2004	37.20	34.73	38.47
High SDI	Both	Colon and rectum cancer	2004	45.44	43.11	46.67
High-middle SDI	Male	Colon and rectum cancer	2004	35.35	33.93	36.84
High-middle SDI	Female	Colon and rectum cancer	2004	23.16	21.86	24.13
High-middle SDI	Both	Colon and rectum cancer	2004	28.36	27.06	29.32
Low SDI	Male	Colon and rectum cancer	2004	6.14	5.37	7.55
Low SDI	Female	Colon and rectum cancer	2004	6.03	5.14	6.90
Low SDI	Both	Colon and rectum cancer	2004	6.09	5.38	7.05
Low-middle SDI	Male	Colon and rectum cancer	2004	8.74	8.15	9.60
Low-middle SDI	Female	Colon and rectum cancer	2004	7.94	7.25	8.64
Low-middle SDI	Both	Colon and rectum cancer	2004	8.33	7.80	8.90

Middle SDI	Male	Colon and rectum cancer	2004	14.89	13.86	16.13
Middle SDI	Female	Colon and rectum cancer	2004	12.64	11.62	13.69
Middle SDI	Both	Colon and rectum cancer	2004	13.75	12.88	14.67
High SDI	Male	Colon and rectum cancer	2005	55.58	53.43	56.85
High SDI	Female	Colon and rectum cancer	2005	36.84	34.37	38.13
High SDI	Both	Colon and rectum cancer	2005	45.26	42.83	46.48
High-middle SDI	Male	Colon and rectum cancer	2005	36.51	34.94	37.95
High-middle SDI	Female	Colon and rectum cancer	2005	23.57	22.27	24.46
High-middle SDI	Both	Colon and rectum cancer	2005	29.11	27.78	30.10
Low SDI	Male	Colon and rectum cancer	2005	6.20	5.42	7.58
Low SDI	Female	Colon and rectum cancer	2005	6.12	5.22	6.99
Low SDI	Both	Colon and rectum cancer	2005	6.16	5.44	7.16
Low-middle SDI	Male	Colon and rectum cancer	2005	9.02	8.42	9.84
Low-middle SDI	Female	Colon and rectum cancer	2005	8.12	7.38	8.86
Low-middle SDI	Both	Colon and rectum cancer	2005	8.57	8.02	9.15
Middle SDI	Male	Colon and rectum cancer	2005	15.29	14.28	16.52
Middle SDI	Female	Colon and rectum cancer	2005	12.93	11.91	14.04
Middle SDI	Both	Colon and rectum cancer	2005	14.09	13.24	15.03
High SDI	Male	Colon and rectum cancer	2006	54.76	52.58	56.07
High SDI	Female	Colon and rectum cancer	2006	36.18	33.64	37.53
High SDI	Both	Colon and rectum cancer	2006	44.56	42.18	45.90
High-middle SDI	Male	Colon and rectum cancer	2006	36.52	35.00	38.03
High-middle SDI	Female	Colon and rectum cancer	2006	23.35	21.95	24.33
High-middle SDI	Both	Colon and rectum cancer	2006	29.01	27.67	30.02
Low SDI	Male	Colon and rectum cancer	2006	6.22	5.48	7.59
Low SDI	Female	Colon and rectum cancer	2006	6.16	5.31	6.99
Low SDI	Both	Colon and rectum cancer	2006	6.19	5.48	7.16
Low-middle SDI	Male	Colon and rectum cancer	2006	9.26	8.62	10.08
Low-middle SDI	Female	Colon and rectum cancer	2006	8.24	7.56	8.98
Low-middle SDI	Both	Colon and rectum cancer	2006	8.74	8.20	9.29
Middle SDI	Male	Colon and rectum cancer	2006	15.63	14.69	16.80
Middle SDI	Female	Colon and rectum cancer	2006	13.15	12.16	14.13
Middle SDI	Both	Colon and rectum cancer	2006	14.36	13.49	15.18
High SDI	Male	Colon and rectum cancer	2007	54.73	52.55	56.01
High SDI	Female	Colon and rectum cancer	2007	35.88	33.31	37.38
High SDI	Both	Colon and rectum cancer	2007	44.41	42.02	45.75
High-middle SDI	Male	Colon and rectum cancer	2007	37.27	35.76	38.79
High-middle SDI	Female	Colon and rectum cancer	2007	23.64	22.20	24.68
High-middle SDI	Both	Colon and rectum cancer	2007	29.52	28.14	30.53
Low SDI	Male	Colon and rectum cancer	2007	6.31	5.57	7.60
Low SDI	Female	Colon and rectum cancer	2007	6.15	5.32	7.01
Low SDI	Both	Colon and rectum cancer	2007	6.23	5.56	7.17
Low-middle SDI	Male	Colon and rectum cancer	2007	9.49	8.91	10.29
Low-middle SDI	Female	Colon and rectum cancer	2007	8.34	7.61	9.07
Low-middle SDI	Both	Colon and rectum cancer	2007	8.90	8.35	9.46
Middle SDI	Male	Colon and rectum cancer	2007	16.35	15.31	17.55
Middle SDI	Female	Colon and rectum cancer	2007	13.43	12.48	14.42
Middle SDI	Both	Colon and rectum cancer	2007	14.85	14.05	15.75
High SDI	Male	Colon and rectum cancer	2008	55.02	52.73	56.37
High SDI	Female	Colon and rectum cancer	2008	35.91	33.32	37.40
High SDI	Both	Colon and rectum cancer	2008	44.59	42.11	45.93
High-middle SDI	Male	Colon and rectum cancer	2008	38.27	36.61	39.86
High-middle SDI	Female	Colon and rectum cancer	2008	24.06	22.64	25.15
High-middle SDI	Both	Colon and rectum cancer	2008	30.20	28.87	31.30
Low SDI	Male	Colon and rectum cancer	2008	6.52	5.77	7.77
Low SDI	Female	Colon and rectum cancer	2008	6.20	5.39	7.04

Low SDI	Both	Colon and rectum cancer	2008		6.36	5.67	7.31
Low-middle SDI	Male	Colon and rectum cancer	2008		9.78	9.17	10.58
Low-middle SDI	Female	Colon and rectum cancer	2008		8.42	7.67	9.19
Low-middle SDI	Both	Colon and rectum cancer	2008		9.09	8.51	9.69
Middle SDI	Male	Colon and rectum cancer	2008		17.08	15.97	18.33
Middle SDI	Female	Colon and rectum cancer	2008		13.80	12.81	14.77
Middle SDI	Both	Colon and rectum cancer	2008		15.39	14.51	16.37
High SDI	Male	Colon and rectum cancer	2009		55.05	52.76	56.45
High SDI	Female	Colon and rectum cancer	2009		35.61	32.92	37.07
High SDI	Both	Colon and rectum cancer	2009		44.46	41.95	45.76
High-middle SDI	Male	Colon and rectum cancer	2009		38.86	36.97	40.60
High-middle SDI	Female	Colon and rectum cancer	2009		24.21	22.74	25.33
High-middle SDI	Both	Colon and rectum cancer	2009		30.56	29.08	31.72
Low SDI	Male	Colon and rectum cancer	2009		6.58	5.83	7.78
Low SDI	Female	Colon and rectum cancer	2009		6.24	5.40	7.05
Low SDI	Both	Colon and rectum cancer	2009		6.41	5.73	7.33
Low-middle SDI	Male	Colon and rectum cancer	2009		9.95	9.25	10.68
Low-middle SDI	Female	Colon and rectum cancer	2009		8.44	7.73	9.10
Low-middle SDI	Both	Colon and rectum cancer	2009		9.17	8.62	9.75
Middle SDI	Male	Colon and rectum cancer	2009		17.88	16.68	19.22
Middle SDI	Female	Colon and rectum cancer	2009		14.16	13.06	15.12
Middle SDI	Both	Colon and rectum cancer	2009		15.96	14.99	16.91
High SDI	Male	Colon and rectum cancer	2010		54.60	52.30	55.95
High SDI	Female	Colon and rectum cancer	2010		34.99	32.31	36.46
High SDI	Both	Colon and rectum cancer	2010		43.94	41.38	45.29
High-middle SDI	Male	Colon and rectum cancer	2010		39.71	37.68	41.70
High-middle SDI	Female	Colon and rectum cancer	2010		24.34	22.88	25.52
High-middle SDI	Both	Colon and rectum cancer	2010		31.02	29.54	32.26
Low SDI	Male	Colon and rectum cancer	2010		6.60	5.88	7.79
Low SDI	Female	Colon and rectum cancer	2010		6.25	5.44	7.09
Low SDI	Both	Colon and rectum cancer	2010		6.42	5.75	7.34
Low-middle SDI	Male	Colon and rectum cancer	2010		10.13	9.50	10.94
Low-middle SDI	Female	Colon and rectum cancer	2010		8.53	7.80	9.25
Low-middle SDI	Both	Colon and rectum cancer	2010		9.31	8.77	9.91
Middle SDI	Male	Colon and rectum cancer	2010		18.64	17.29	20.20
Middle SDI	Female	Colon and rectum cancer	2010		14.51	13.42	15.57
Middle SDI	Both	Colon and rectum cancer	2010		16.51	15.46	17.46
High SDI	Male	Colon and rectum cancer	2011		54.18	51.85	55.52
High SDI	Female	Colon and rectum cancer	2011		34.92	32.13	36.36
High SDI	Both	Colon and rectum cancer	2011		43.73	41.11	45.03
High-middle SDI	Male	Colon and rectum cancer	2011		39.82	37.72	42.05
High-middle SDI	Female	Colon and rectum cancer	2011		24.18	22.75	25.51
High-middle SDI	Both	Colon and rectum cancer	2011		30.99	29.49	32.28
Low SDI	Male	Colon and rectum cancer	2011		6.66	5.92	7.77
Low SDI	Female	Colon and rectum cancer	2011		6.31	5.52	7.13
Low SDI	Both	Colon and rectum cancer	2011		6.48	5.82	7.33
Low-middle SDI	Male	Colon and rectum cancer	2011		10.29	9.63	11.02
Low-middle SDI	Female	Colon and rectum cancer	2011		8.66	7.89	9.43
Low-middle SDI	Both	Colon and rectum cancer	2011		9.46	8.87	10.09
Middle SDI	Male	Colon and rectum cancer	2011		19.08	17.62	20.65
Middle SDI	Female	Colon and rectum cancer	2011		14.68	13.50	15.88
Middle SDI	Both	Colon and rectum cancer	2011		16.81	15.72	17.93
High SDI	Male	Colon and rectum cancer	2012		53.57	51.16	54.95
High SDI	Female	Colon and rectum cancer	2012		34.32	31.54	35.72
High SDI	Both	Colon and rectum cancer	2012		43.14	40.50	44.46
High-middle SDI	Male	Colon and rectum cancer	2012		40.08	37.88	42.26

High-middle SDI	Female	Colon and rectum cancer	2012	23.94	22.24	25.21
High-middle SDI	Both	Colon and rectum cancer	2012	30.98	29.39	32.35
Low SDI	Male	Colon and rectum cancer	2012	6.76	5.99	7.80
Low SDI	Female	Colon and rectum cancer	2012	6.47	5.67	7.26
Low SDI	Both	Colon and rectum cancer	2012	6.61	5.96	7.40
Low-middle SDI	Male	Colon and rectum cancer	2012	10.49	9.80	11.25
Low-middle SDI	Female	Colon and rectum cancer	2012	8.81	8.09	9.52
Low-middle SDI	Both	Colon and rectum cancer	2012	9.63	9.04	10.24
Middle SDI	Male	Colon and rectum cancer	2012	19.35	17.86	20.94
Middle SDI	Female	Colon and rectum cancer	2012	14.72	13.56	15.78
Middle SDI	Both	Colon and rectum cancer	2012	16.95	15.84	17.96
High SDI	Male	Colon and rectum cancer	2013	53.23	50.74	54.68
High SDI	Female	Colon and rectum cancer	2013	34.04	31.31	35.46
High SDI	Both	Colon and rectum cancer	2013	42.85	40.17	44.23
High-middle SDI	Male	Colon and rectum cancer	2013	39.94	37.60	42.37
High-middle SDI	Female	Colon and rectum cancer	2013	23.74	22.15	25.01
High-middle SDI	Both	Colon and rectum cancer	2013	30.83	29.20	32.31
Low SDI	Male	Colon and rectum cancer	2013	6.92	6.14	8.03
Low SDI	Female	Colon and rectum cancer	2013	6.60	5.80	7.39
Low SDI	Both	Colon and rectum cancer	2013	6.75	6.05	7.54
Low-middle SDI	Male	Colon and rectum cancer	2013	10.73	10.00	11.54
Low-middle SDI	Female	Colon and rectum cancer	2013	9.03	8.27	9.80
Low-middle SDI	Both	Colon and rectum cancer	2013	9.85	9.26	10.49
Middle SDI	Male	Colon and rectum cancer	2013	19.52	18.00	21.28
Middle SDI	Female	Colon and rectum cancer	2013	14.80	13.53	15.94
Middle SDI	Both	Colon and rectum cancer	2013	17.07	15.97	18.29
High SDI	Male	Colon and rectum cancer	2014	52.49	50.00	53.99
High SDI	Female	Colon and rectum cancer	2014	33.76	31.02	35.21
High SDI	Both	Colon and rectum cancer	2014	42.38	39.77	43.76
High-middle SDI	Male	Colon and rectum cancer	2014	40.19	37.62	42.70
High-middle SDI	Female	Colon and rectum cancer	2014	23.72	22.08	25.05
High-middle SDI	Both	Colon and rectum cancer	2014	30.95	29.25	32.42
Low SDI	Male	Colon and rectum cancer	2014	6.93	6.13	7.88
Low SDI	Female	Colon and rectum cancer	2014	6.74	5.92	7.51
Low SDI	Both	Colon and rectum cancer	2014	6.83	6.11	7.56
Low-middle SDI	Male	Colon and rectum cancer	2014	10.92	10.16	11.75
Low-middle SDI	Female	Colon and rectum cancer	2014	9.29	8.49	10.09
Low-middle SDI	Both	Colon and rectum cancer	2014	10.08	9.43	10.74
Middle SDI	Male	Colon and rectum cancer	2014	19.75	18.08	21.68
Middle SDI	Female	Colon and rectum cancer	2014	14.94	13.64	16.22
Middle SDI	Both	Colon and rectum cancer	2014	17.26	16.02	18.42
High SDI	Male	Colon and rectum cancer	2015	52.57	50.08	54.16
High SDI	Female	Colon and rectum cancer	2015	33.69	31.01	35.14
High SDI	Both	Colon and rectum cancer	2015	42.40	39.80	43.80
High-middle SDI	Male	Colon and rectum cancer	2015	40.91	38.08	43.91
High-middle SDI	Female	Colon and rectum cancer	2015	24.03	22.47	25.48
High-middle SDI	Both	Colon and rectum cancer	2015	31.46	29.64	32.99
Low SDI	Male	Colon and rectum cancer	2015	7.02	6.24	7.97
Low SDI	Female	Colon and rectum cancer	2015	6.83	6.04	7.60
Low SDI	Both	Colon and rectum cancer	2015	6.92	6.23	7.66
Low-middle SDI	Male	Colon and rectum cancer	2015	11.04	10.24	11.94
Low-middle SDI	Female	Colon and rectum cancer	2015	9.58	8.63	10.48
Low-middle SDI	Both	Colon and rectum cancer	2015	10.28	9.59	11.04
Middle SDI	Male	Colon and rectum cancer	2015	19.86	18.08	21.81
Middle SDI	Female	Colon and rectum cancer	2015	14.96	13.67	16.28
Middle SDI	Both	Colon and rectum cancer	2015	17.32	16.05	18.59

High SDI	Male	Colon and rectum cancer	2016		52.47	49.95	54.20
High SDI	Female	Colon and rectum cancer	2016		33.85	31.23	35.31
High SDI	Both	Colon and rectum cancer	2016		42.46	39.91	43.91
High-middle SDI	Male	Colon and rectum cancer	2016		40.86	37.93	44.02
High-middle SDI	Female	Colon and rectum cancer	2016		23.87	22.13	25.48
High-middle SDI	Both	Colon and rectum cancer	2016		31.36	29.44	33.11
Low SDI	Male	Colon and rectum cancer	2016		7.06	6.28	8.04
Low SDI	Female	Colon and rectum cancer	2016		6.91	6.11	7.73
Low SDI	Both	Colon and rectum cancer	2016		6.98	6.29	7.72
Low-middle SDI	Male	Colon and rectum cancer	2016		11.20	10.34	12.26
Low-middle SDI	Female	Colon and rectum cancer	2016		9.75	8.78	10.81
Low-middle SDI	Both	Colon and rectum cancer	2016		10.45	9.66	11.32
Middle SDI	Male	Colon and rectum cancer	2016		19.99	18.08	22.04
Middle SDI	Female	Colon and rectum cancer	2016		15.05	13.54	16.48
Middle SDI	Both	Colon and rectum cancer	2016		17.42	16.13	18.76
High SDI	Male	Colon and rectum cancer	2017		52.26	49.32	54.91
High SDI	Female	Colon and rectum cancer	2017		33.63	30.84	35.74
High SDI	Both	Colon and rectum cancer	2017		42.25	39.37	44.53
High-middle SDI	Male	Colon and rectum cancer	2017		40.99	37.49	44.81
High-middle SDI	Female	Colon and rectum cancer	2017		23.89	21.85	25.96
High-middle SDI	Both	Colon and rectum cancer	2017		31.46	29.32	33.57
Low SDI	Male	Colon and rectum cancer	2017		7.13	6.30	8.16
Low SDI	Female	Colon and rectum cancer	2017		7.00	6.11	7.82
Low SDI	Both	Colon and rectum cancer	2017		7.06	6.36	7.85
Low-middle SDI	Male	Colon and rectum cancer	2017		11.47	10.49	12.63
Low-middle SDI	Female	Colon and rectum cancer	2017		9.92	8.78	11.06
Low-middle SDI	Both	Colon and rectum cancer	2017		10.67	9.78	11.62
Middle SDI	Male	Colon and rectum cancer	2017		20.52	18.41	22.87
Middle SDI	Female	Colon and rectum cancer	2017		15.40	13.64	17.22
Middle SDI	Both	Colon and rectum cancer	2017		17.86	16.28	19.38
High SDI	Male	Colon and rectum cancer	2018		52.67	47.89	57.23
High SDI	Female	Colon and rectum cancer	2018		33.84	30.59	36.87
High SDI	Both	Colon and rectum cancer	2018		42.57	39.03	45.94
High-middle SDI	Male	Colon and rectum cancer	2018		41.54	37.41	45.97
High-middle SDI	Female	Colon and rectum cancer	2018		24.28	21.85	26.56
High-middle SDI	Both	Colon and rectum cancer	2018		31.95	29.52	34.42
Low SDI	Male	Colon and rectum cancer	2018		7.29	6.37	8.34
Low SDI	Female	Colon and rectum cancer	2018		7.15	6.27	8.02
Low SDI	Both	Colon and rectum cancer	2018		7.22	6.49	8.01
Low-middle SDI	Male	Colon and rectum cancer	2018		11.88	10.75	13.10
Low-middle SDI	Female	Colon and rectum cancer	2018		10.17	8.92	11.39
Low-middle SDI	Both	Colon and rectum cancer	2018		10.99	10.02	11.95
Middle SDI	Male	Colon and rectum cancer	2018		21.20	18.69	23.79
Middle SDI	Female	Colon and rectum cancer	2018		15.85	13.89	17.83
Middle SDI	Both	Colon and rectum cancer	2018		18.42	16.58	20.26
High SDI	Male	Colon and rectum cancer	2019		52.80	47.70	58.26
High SDI	Female	Colon and rectum cancer	2019		34.08	30.07	37.55
High SDI	Both	Colon and rectum cancer	2019		42.78	38.75	46.64
High-middle SDI	Male	Colon and rectum cancer	2019		42.13	37.59	47.15
High-middle SDI	Female	Colon and rectum cancer	2019		24.58	21.85	27.26
High-middle SDI	Both	Colon and rectum cancer	2019		32.41	29.40	35.39
Low SDI	Male	Colon and rectum cancer	2019		7.40	6.48	8.48
Low SDI	Female	Colon and rectum cancer	2019		7.27	6.37	8.17
Low SDI	Both	Colon and rectum cancer	2019		7.33	6.55	8.13
Low-middle SDI	Male	Colon and rectum cancer	2019		12.21	10.94	13.50
Low-middle SDI	Female	Colon and rectum cancer	2019		10.40	9.14	11.72

Low-middle SDI	Both	Colon and rectum cancer	2019	11.27	10.23	12.35
Middle SDI	Male	Colon and rectum cancer	2019	21.79	19.20	24.81
Middle SDI	Female	Colon and rectum cancer	2019	16.19	14.08	18.38
Middle SDI	Both	Colon and rectum cancer	2019	18.87	16.99	20.86

sex_name	cause_name	year	Age-standardised death rate (per 100 000 person-years)	95% CI (lower)	95% CI (upper)
Male	Colon and rectum cancer	1990	16.16	15.44	16.93
Female	Colon and rectum cancer	1990	12.91	12.03	13.64
Both	Colon and rectum cancer	1990	14.31	13.52	14.88
Male	Colon and rectum cancer	1991	16.15	15.39	16.85
Female	Colon and rectum cancer	1991	12.84	11.97	13.56
Both	Colon and rectum cancer	1991	14.27	13.49	14.83
Male	Colon and rectum cancer	1992	16.21	15.47	16.88
Female	Colon and rectum cancer	1992	12.80	11.96	13.51
Both	Colon and rectum cancer	1992	14.28	13.51	14.82
Male	Colon and rectum cancer	1993	16.40	15.70	17.04
Female	Colon and rectum cancer	1993	12.93	12.05	13.52
Both	Colon and rectum cancer	1993	14.44	13.69	14.95
Male	Colon and rectum cancer	1994	16.41	15.71	17.01
Female	Colon and rectum cancer	1994	12.88	11.99	13.46
Both	Colon and rectum cancer	1994	14.43	13.64	14.92
Male	Colon and rectum cancer	1995	16.42	15.71	17.00
Female	Colon and rectum cancer	1995	12.78	11.93	13.38
Both	Colon and rectum cancer	1995	14.38	13.63	14.87
Male	Colon and rectum cancer	1996	16.33	15.61	16.86
Female	Colon and rectum cancer	1996	12.66	11.74	13.20
Both	Colon and rectum cancer	1996	14.28	13.52	14.76
Male	Colon and rectum cancer	1997	16.25	15.56	16.77
Female	Colon and rectum cancer	1997	12.58	11.69	13.13
Both	Colon and rectum cancer	1997	14.20	13.43	14.68
Male	Colon and rectum cancer	1998	16.31	15.61	16.86
Female	Colon and rectum cancer	1998	12.55	11.68	13.11
Both	Colon and rectum cancer	1998	14.21	13.43	14.69
Male	Colon and rectum cancer	1999	16.45	15.78	16.98
Female	Colon and rectum cancer	1999	12.65	11.77	13.24
Both	Colon and rectum cancer	1999	14.34	13.54	14.82
Male	Colon and rectum cancer	2000	16.54	15.84	17.06
Female	Colon and rectum cancer	2000	12.67	11.70	13.26
Both	Colon and rectum cancer	2000	14.40	13.56	14.92
Male	Colon and rectum cancer	2001	16.62	15.85	17.21
Female	Colon and rectum cancer	2001	12.65	11.68	13.23
Both	Colon and rectum cancer	2001	14.43	13.55	14.94
Male	Colon and rectum cancer	2002	16.84	16.05	17.40
Female	Colon and rectum cancer	2002	12.68	11.68	13.28
Both	Colon and rectum cancer	2002	14.55	13.64	15.09
Male	Colon and rectum cancer	2003	16.99	16.22	17.57
Female	Colon and rectum cancer	2003	12.66	11.65	13.29
Both	Colon and rectum cancer	2003	14.62	13.70	15.17
Male	Colon and rectum cancer	2004	17.04	16.22	17.68
Female	Colon and rectum cancer	2004	12.49	11.48	13.09
Both	Colon and rectum cancer	2004	14.54	13.61	15.12
Male	Colon and rectum cancer	2005	17.17	16.33	17.86
Female	Colon and rectum cancer	2005	12.44	11.43	13.06
Both	Colon and rectum cancer	2005	14.58	13.66	15.17
Male	Colon and rectum cancer	2006	16.98	16.12	17.66

Female	Colon and rectum cancer	2006	12.24	11.22	12.86
Both	Colon and rectum cancer	2006	14.39	13.48	14.96
Male	Colon and rectum cancer	2007	16.96	16.13	17.61
Female	Colon and rectum cancer	2007	12.11	11.08	12.74
Both	Colon and rectum cancer	2007	14.31	13.32	14.90
Male	Colon and rectum cancer	2008	17.05	16.20	17.72
Female	Colon and rectum cancer	2008	12.03	11.04	12.66
Both	Colon and rectum cancer	2008	14.30	13.39	14.93
Male	Colon and rectum cancer	2009	17.01	16.12	17.64
Female	Colon and rectum cancer	2009	11.86	10.84	12.48
Both	Colon and rectum cancer	2009	14.19	13.21	14.75
Male	Colon and rectum cancer	2010	17.02	16.16	17.72
Female	Colon and rectum cancer	2010	11.72	10.71	12.35
Both	Colon and rectum cancer	2010	14.12	13.17	14.70
Male	Colon and rectum cancer	2011	16.91	15.96	17.71
Female	Colon and rectum cancer	2011	11.59	10.54	12.23
Both	Colon and rectum cancer	2011	14.00	13.02	14.63
Male	Colon and rectum cancer	2012	16.83	15.89	17.67
Female	Colon and rectum cancer	2012	11.44	10.41	12.02
Both	Colon and rectum cancer	2012	13.88	12.88	14.51
Male	Colon and rectum cancer	2013	16.68	15.68	17.52
Female	Colon and rectum cancer	2013	11.31	10.25	11.93
Both	Colon and rectum cancer	2013	13.74	12.77	14.37
Male	Colon and rectum cancer	2014	16.53	15.55	17.32
Female	Colon and rectum cancer	2014	11.21	10.15	11.85
Both	Colon and rectum cancer	2014	13.62	12.65	14.26
Male	Colon and rectum cancer	2015	16.53	15.58	17.40
Female	Colon and rectum cancer	2015	11.20	10.08	11.86
Both	Colon and rectum cancer	2015	13.61	12.63	14.25
Male	Colon and rectum cancer	2016	16.47	15.36	17.41
Female	Colon and rectum cancer	2016	11.14	10.09	11.90
Both	Colon and rectum cancer	2016	13.55	12.49	14.27
Male	Colon and rectum cancer	2017	16.41	15.32	17.37
Female	Colon and rectum cancer	2017	11.09	9.95	11.81
Both	Colon and rectum cancer	2017	13.50	12.46	14.23
Male	Colon and rectum cancer	2018	16.52	15.39	17.65
Female	Colon and rectum cancer	2018	11.16	10.05	12.00
Both	Colon and rectum cancer	2018	13.59	12.46	14.43
Male	Colon and rectum cancer	2019	16.64	15.39	17.85
Female	Colon and rectum cancer	2019	11.24	10.01	12.17
Both	Colon and rectum cancer	2019	13.69	12.60	14.51

location_name	sex_name	cause_name	year	Age-standardised death rate (per 100 000 person-years)	95% CI (lower)	95% CI (upper)
High SDI	Male	Colon and rectum cancer	1990	26.02	25.01	26.59
High SDI	Female	Colon and rectum cancer	1990	17.84	16.68	18.47
High SDI	Both	Colon and rectum cancer	1990	21.18	20.08	21.75
High-middle SDI	Male	Colon and rectum cancer	1990	19.07	18.23	19.88
High-middle SDI	Female	Colon and rectum cancer	1990	14.14	13.35	14.87
High-middle SDI	Both	Colon and rectum cancer	1990	16.08	15.28	16.70
Low SDI	Male	Colon and rectum cancer	1990	6.78	5.57	8.45
Low SDI	Female	Colon and rectum cancer	1990	5.66	4.36	7.22
Low SDI	Both	Colon and rectum cancer	1990	6.22	5.21	7.26
Low-middle SDI	Male	Colon and rectum cancer	1990	6.58	5.85	8.03
Low-middle SDI	Female	Colon and rectum cancer	1990	6.41	5.52	7.44
Low-middle SDI	Both	Colon and rectum cancer	1990	6.50	5.87	7.28
Middle SDI	Male	Colon and rectum cancer	1990	9.33	8.46	10.29
Middle SDI	Female	Colon and rectum cancer	1990	8.27	7.49	9.12
Middle SDI	Both	Colon and rectum cancer	1990	8.77	8.10	9.44
High SDI	Male	Colon and rectum cancer	1991	25.89	24.91	26.46
High SDI	Female	Colon and rectum cancer	1991	17.64	16.47	18.25
High SDI	Both	Colon and rectum cancer	1991	21.02	19.93	21.57
High-middle SDI	Male	Colon and rectum cancer	1991	19.23	18.36	20.04
High-middle SDI	Female	Colon and rectum cancer	1991	14.20	13.41	14.95
High-middle SDI	Both	Colon and rectum cancer	1991	16.19	15.41	16.79
Low SDI	Male	Colon and rectum cancer	1991	6.77	5.62	8.43
Low SDI	Female	Colon and rectum cancer	1991	5.64	4.31	7.24
Low SDI	Both	Colon and rectum cancer	1991	6.21	5.19	7.26
Low-middle SDI	Male	Colon and rectum cancer	1991	6.61	5.91	7.99
Low-middle SDI	Female	Colon and rectum cancer	1991	6.40	5.50	7.43
Low-middle SDI	Both	Colon and rectum cancer	1991	6.51	5.87	7.28
Middle SDI	Male	Colon and rectum cancer	1991	9.41	8.61	10.26
Middle SDI	Female	Colon and rectum cancer	1991	8.29	7.57	9.22
Middle SDI	Both	Colon and rectum cancer	1991	8.82	8.22	9.51
High SDI	Male	Colon and rectum cancer	1992	25.82	24.83	26.40
High SDI	Female	Colon and rectum cancer	1992	17.45	16.27	18.05
High SDI	Both	Colon and rectum cancer	1992	20.89	19.77	21.45
High-middle SDI	Male	Colon and rectum cancer	1992	19.54	18.75	20.33
High-middle SDI	Female	Colon and rectum cancer	1992	14.29	13.53	14.89
High-middle SDI	Both	Colon and rectum cancer	1992	16.38	15.67	16.92
Low SDI	Male	Colon and rectum cancer	1992	6.75	5.55	8.45
Low SDI	Female	Colon and rectum cancer	1992	5.63	4.38	7.16
Low SDI	Both	Colon and rectum cancer	1992	6.20	5.22	7.23
Low-middle SDI	Male	Colon and rectum cancer	1992	6.64	5.87	7.99
Low-middle SDI	Female	Colon and rectum cancer	1992	6.44	5.52	7.52
Low-middle SDI	Both	Colon and rectum cancer	1992	6.55	5.92	7.32
Middle SDI	Male	Colon and rectum cancer	1992	9.49	8.73	10.34
Middle SDI	Female	Colon and rectum cancer	1992	8.37	7.67	9.21
Middle SDI	Both	Colon and rectum cancer	1992	8.90	8.34	9.53
High SDI	Male	Colon and rectum cancer	1993	25.91	24.95	26.45
High SDI	Female	Colon and rectum cancer	1993	17.45	16.19	18.03
High SDI	Both	Colon and rectum cancer	1993	20.94	19.81	21.50
High-middle SDI	Male	Colon and rectum cancer	1993	20.11	19.35	20.84

High-middle SDI	Female	Colon and rectum cancer	1993	14.73	13.96	15.32
High-middle SDI	Both	Colon and rectum cancer	1993	16.88	16.15	17.38
Low SDI	Male	Colon and rectum cancer	1993	6.77	5.65	8.42
Low SDI	Female	Colon and rectum cancer	1993	5.62	4.39	7.02
Low SDI	Both	Colon and rectum cancer	1993	6.20	5.27	7.18
Low-middle SDI	Male	Colon and rectum cancer	1993	6.76	6.09	8.02
Low-middle SDI	Female	Colon and rectum cancer	1993	6.49	5.63	7.42
Low-middle SDI	Both	Colon and rectum cancer	1993	6.63	6.03	7.35
Middle SDI	Male	Colon and rectum cancer	1993	9.62	8.90	10.39
Middle SDI	Female	Colon and rectum cancer	1993	8.43	7.74	9.25
Middle SDI	Both	Colon and rectum cancer	1993	9.00	8.45	9.60
High SDI	Male	Colon and rectum cancer	1994	25.69	24.72	26.25
High SDI	Female	Colon and rectum cancer	1994	17.22	15.96	17.81
High SDI	Both	Colon and rectum cancer	1994	20.73	19.59	21.30
High-middle SDI	Male	Colon and rectum cancer	1994	20.39	19.63	21.13
High-middle SDI	Female	Colon and rectum cancer	1994	14.87	14.12	15.42
High-middle SDI	Both	Colon and rectum cancer	1994	17.09	16.36	17.60
Low SDI	Male	Colon and rectum cancer	1994	6.76	5.69	8.40
Low SDI	Female	Colon and rectum cancer	1994	5.60	4.40	6.88
Low SDI	Both	Colon and rectum cancer	1994	6.18	5.29	7.13
Low-middle SDI	Male	Colon and rectum cancer	1994	6.85	6.19	8.06
Low-middle SDI	Female	Colon and rectum cancer	1994	6.53	5.70	7.41
Low-middle SDI	Both	Colon and rectum cancer	1994	6.69	6.12	7.38
Middle SDI	Male	Colon and rectum cancer	1994	9.70	9.07	10.46
Middle SDI	Female	Colon and rectum cancer	1994	8.44	7.73	9.19
Middle SDI	Both	Colon and rectum cancer	1994	9.04	8.47	9.64
High SDI	Male	Colon and rectum cancer	1995	25.66	24.67	26.25
High SDI	Female	Colon and rectum cancer	1995	17.12	15.81	17.73
High SDI	Both	Colon and rectum cancer	1995	20.68	19.49	21.25
High-middle SDI	Male	Colon and rectum cancer	1995	20.38	19.63	21.06
High-middle SDI	Female	Colon and rectum cancer	1995	14.68	13.92	15.24
High-middle SDI	Both	Colon and rectum cancer	1995	16.98	16.31	17.47
Low SDI	Male	Colon and rectum cancer	1995	6.75	5.73	8.44
Low SDI	Female	Colon and rectum cancer	1995	5.58	4.46	6.88
Low SDI	Both	Colon and rectum cancer	1995	6.17	5.29	7.13
Low-middle SDI	Male	Colon and rectum cancer	1995	6.88	6.27	7.98
Low-middle SDI	Female	Colon and rectum cancer	1995	6.56	5.75	7.43
Low-middle SDI	Both	Colon and rectum cancer	1995	6.73	6.19	7.37
Middle SDI	Male	Colon and rectum cancer	1995	9.85	9.21	10.55
Middle SDI	Female	Colon and rectum cancer	1995	8.44	7.73	9.23
Middle SDI	Both	Colon and rectum cancer	1995	9.11	8.54	9.74
High SDI	Male	Colon and rectum cancer	1996	25.31	24.33	25.89
High SDI	Female	Colon and rectum cancer	1996	16.89	15.58	17.51
High SDI	Both	Colon and rectum cancer	1996	20.42	19.25	20.99
High-middle SDI	Male	Colon and rectum cancer	1996	20.16	19.43	20.77
High-middle SDI	Female	Colon and rectum cancer	1996	14.41	13.61	14.93
High-middle SDI	Both	Colon and rectum cancer	1996	16.72	16.00	17.21
Low SDI	Male	Colon and rectum cancer	1996	6.76	5.78	8.40
Low SDI	Female	Colon and rectum cancer	1996	5.60	4.52	6.77
Low SDI	Both	Colon and rectum cancer	1996	6.18	5.32	7.12
Low-middle SDI	Male	Colon and rectum cancer	1996	7.00	6.37	8.12
Low-middle SDI	Female	Colon and rectum cancer	1996	6.66	5.94	7.45

Low-middle SDI	Both	Colon and rectum cancer	1996	6.83	6.31	7.49
Middle SDI	Male	Colon and rectum cancer	1996	10.09	9.44	10.82
Middle SDI	Female	Colon and rectum cancer	1996	8.55	7.87	9.25
Middle SDI	Both	Colon and rectum cancer	1996	9.28	8.73	9.85
High SDI	Male	Colon and rectum cancer	1997	24.92	23.85	25.48
High SDI	Female	Colon and rectum cancer	1997	16.69	15.42	17.33
High SDI	Both	Colon and rectum cancer	1997	20.16	18.95	20.73
High-middle SDI	Male	Colon and rectum cancer	1997	20.00	19.20	20.58
High-middle SDI	Female	Colon and rectum cancer	1997	14.23	13.50	14.76
High-middle SDI	Both	Colon and rectum cancer	1997	16.55	15.80	17.00
Low SDI	Male	Colon and rectum cancer	1997	6.82	5.86	8.45
Low SDI	Female	Colon and rectum cancer	1997	5.64	4.58	6.73
Low SDI	Both	Colon and rectum cancer	1997	6.23	5.41	7.16
Low-middle SDI	Male	Colon and rectum cancer	1997	7.25	6.64	8.37
Low-middle SDI	Female	Colon and rectum cancer	1997	6.82	6.04	7.65
Low-middle SDI	Both	Colon and rectum cancer	1997	7.04	6.50	7.68
Middle SDI	Male	Colon and rectum cancer	1997	10.27	9.67	10.91
Middle SDI	Female	Colon and rectum cancer	1997	8.61	7.95	9.34
Middle SDI	Both	Colon and rectum cancer	1997	9.40	8.88	9.98
High SDI	Male	Colon and rectum cancer	1998	24.86	23.81	25.40
High SDI	Female	Colon and rectum cancer	1998	16.66	15.39	17.29
High SDI	Both	Colon and rectum cancer	1998	20.14	18.93	20.71
High-middle SDI	Male	Colon and rectum cancer	1998	20.00	19.18	20.63
High-middle SDI	Female	Colon and rectum cancer	1998	14.11	13.40	14.59
High-middle SDI	Both	Colon and rectum cancer	1998	16.47	15.73	16.95
Low SDI	Male	Colon and rectum cancer	1998	6.85	5.93	8.52
Low SDI	Female	Colon and rectum cancer	1998	5.69	4.66	6.71
Low SDI	Both	Colon and rectum cancer	1998	6.27	5.46	7.25
Low-middle SDI	Male	Colon and rectum cancer	1998	7.38	6.80	8.47
Low-middle SDI	Female	Colon and rectum cancer	1998	6.90	6.11	7.71
Low-middle SDI	Both	Colon and rectum cancer	1998	7.14	6.60	7.77
Middle SDI	Male	Colon and rectum cancer	1998	10.50	9.87	11.25
Middle SDI	Female	Colon and rectum cancer	1998	8.66	7.95	9.42
Middle SDI	Both	Colon and rectum cancer	1998	9.53	8.95	10.16
High SDI	Male	Colon and rectum cancer	1999	24.87	23.83	25.42
High SDI	Female	Colon and rectum cancer	1999	16.76	15.44	17.39
High SDI	Both	Colon and rectum cancer	1999	20.21	18.99	20.79
High-middle SDI	Male	Colon and rectum cancer	1999	20.36	19.58	21.00
High-middle SDI	Female	Colon and rectum cancer	1999	14.30	13.50	14.81
High-middle SDI	Both	Colon and rectum cancer	1999	16.75	15.97	17.22
Low SDI	Male	Colon and rectum cancer	1999	6.81	5.88	8.38
Low SDI	Female	Colon and rectum cancer	1999	5.69	4.65	6.66
Low SDI	Both	Colon and rectum cancer	1999	6.25	5.44	7.27
Low-middle SDI	Male	Colon and rectum cancer	1999	7.37	6.78	8.38
Low-middle SDI	Female	Colon and rectum cancer	1999	6.91	6.15	7.70
Low-middle SDI	Both	Colon and rectum cancer	1999	7.14	6.61	7.74
Middle SDI	Male	Colon and rectum cancer	1999	10.71	9.98	11.46
Middle SDI	Female	Colon and rectum cancer	1999	8.75	7.99	9.49
Middle SDI	Both	Colon and rectum cancer	1999	9.68	9.08	10.30
High SDI	Male	Colon and rectum cancer	2000	24.67	23.59	25.23
High SDI	Female	Colon and rectum cancer	2000	16.75	15.43	17.41
High SDI	Both	Colon and rectum cancer	2000	20.14	18.89	20.75

High-middle SDI	Male	Colon and rectum cancer	2000	20.50	19.68	21.14
High-middle SDI	Female	Colon and rectum cancer	2000	14.25	13.45	14.81
High-middle SDI	Both	Colon and rectum cancer	2000	16.79	16.01	17.31
Low SDI	Male	Colon and rectum cancer	2000	6.86	5.89	8.48
Low SDI	Female	Colon and rectum cancer	2000	5.72	4.70	6.63
Low SDI	Both	Colon and rectum cancer	2000	6.29	5.47	7.28
Low-middle SDI	Male	Colon and rectum cancer	2000	7.51	6.93	8.46
Low-middle SDI	Female	Colon and rectum cancer	2000	6.97	6.21	7.73
Low-middle SDI	Both	Colon and rectum cancer	2000	7.24	6.72	7.78
Middle SDI	Male	Colon and rectum cancer	2000	11.02	10.31	11.76
Middle SDI	Female	Colon and rectum cancer	2000	8.87	8.12	9.62
Middle SDI	Both	Colon and rectum cancer	2000	9.89	9.22	10.57
High SDI	Male	Colon and rectum cancer	2001	24.38	23.31	24.96
High SDI	Female	Colon and rectum cancer	2001	16.62	15.27	17.29
High SDI	Both	Colon and rectum cancer	2001	19.96	18.67	20.58
High-middle SDI	Male	Colon and rectum cancer	2001	20.68	19.82	21.41
High-middle SDI	Female	Colon and rectum cancer	2001	14.21	13.36	14.76
High-middle SDI	Both	Colon and rectum cancer	2001	16.85	16.05	17.37
Low SDI	Male	Colon and rectum cancer	2001	6.88	5.96	8.50
Low SDI	Female	Colon and rectum cancer	2001	5.79	4.80	6.70
Low SDI	Both	Colon and rectum cancer	2001	6.33	5.56	7.32
Low-middle SDI	Male	Colon and rectum cancer	2001	7.68	7.12	8.61
Low-middle SDI	Female	Colon and rectum cancer	2001	7.08	6.36	7.83
Low-middle SDI	Both	Colon and rectum cancer	2001	7.38	6.87	7.94
Middle SDI	Male	Colon and rectum cancer	2001	11.35	10.57	12.28
Middle SDI	Female	Colon and rectum cancer	2001	8.98	8.23	9.71
Middle SDI	Both	Colon and rectum cancer	2001	10.11	9.43	10.76
High SDI	Male	Colon and rectum cancer	2002	24.23	23.07	24.84
High SDI	Female	Colon and rectum cancer	2002	16.50	15.13	17.16
High SDI	Both	Colon and rectum cancer	2002	19.84	18.56	20.48
High-middle SDI	Male	Colon and rectum cancer	2002	21.12	20.18	21.88
High-middle SDI	Female	Colon and rectum cancer	2002	14.30	13.42	14.85
High-middle SDI	Both	Colon and rectum cancer	2002	17.09	16.22	17.65
Low SDI	Male	Colon and rectum cancer	2002	6.94	6.06	8.48
Low SDI	Female	Colon and rectum cancer	2002	5.87	4.91	6.76
Low SDI	Both	Colon and rectum cancer	2002	6.40	5.62	7.41
Low-middle SDI	Male	Colon and rectum cancer	2002	7.84	7.28	8.76
Low-middle SDI	Female	Colon and rectum cancer	2002	7.20	6.49	7.99
Low-middle SDI	Both	Colon and rectum cancer	2002	7.52	7.00	8.08
Middle SDI	Male	Colon and rectum cancer	2002	11.81	11.00	12.69
Middle SDI	Female	Colon and rectum cancer	2002	9.14	8.37	9.91
Middle SDI	Both	Colon and rectum cancer	2002	10.41	9.73	11.14
High SDI	Male	Colon and rectum cancer	2003	23.97	22.79	24.61
High SDI	Female	Colon and rectum cancer	2003	16.30	14.92	16.97
High SDI	Both	Colon and rectum cancer	2003	19.64	18.32	20.27
High-middle SDI	Male	Colon and rectum cancer	2003	21.56	20.65	22.33
High-middle SDI	Female	Colon and rectum cancer	2003	14.39	13.49	14.96
High-middle SDI	Both	Colon and rectum cancer	2003	17.34	16.47	17.89
Low SDI	Male	Colon and rectum cancer	2003	7.02	6.09	8.58
Low SDI	Female	Colon and rectum cancer	2003	5.90	4.99	6.78
Low SDI	Both	Colon and rectum cancer	2003	6.45	5.68	7.51
Low-middle SDI	Male	Colon and rectum cancer	2003	7.94	7.39	8.80

Low-middle SDI	Female	Colon and rectum cancer	2003		7.17	6.47	7.90
Low-middle SDI	Both	Colon and rectum cancer	2003		7.55	7.03	8.11
Middle SDI	Male	Colon and rectum cancer	2003		12.22	11.40	13.14
Middle SDI	Female	Colon and rectum cancer	2003		9.30	8.47	10.08
Middle SDI	Both	Colon and rectum cancer	2003		10.69	10.00	11.41
High SDI	Male	Colon and rectum cancer	2004		23.51	22.30	24.15
High SDI	Female	Colon and rectum cancer	2004		15.87	14.49	16.56
High SDI	Both	Colon and rectum cancer	2004		19.20	17.87	19.86
High-middle SDI	Male	Colon and rectum cancer	2004		21.77	20.79	22.61
High-middle SDI	Female	Colon and rectum cancer	2004		14.22	13.32	14.81
High-middle SDI	Both	Colon and rectum cancer	2004		17.34	16.45	17.93
Low SDI	Male	Colon and rectum cancer	2004		7.02	6.14	8.57
Low SDI	Female	Colon and rectum cancer	2004		5.93	5.01	6.81
Low SDI	Both	Colon and rectum cancer	2004		6.46	5.70	7.49
Low-middle SDI	Male	Colon and rectum cancer	2004		7.94	7.42	8.70
Low-middle SDI	Female	Colon and rectum cancer	2004		7.14	6.45	7.78
Low-middle SDI	Both	Colon and rectum cancer	2004		7.53	7.03	8.03
Middle SDI	Male	Colon and rectum cancer	2004		12.68	11.83	13.66
Middle SDI	Female	Colon and rectum cancer	2004		9.41	8.61	10.19
Middle SDI	Both	Colon and rectum cancer	2004		10.97	10.26	11.70
High SDI	Male	Colon and rectum cancer	2005		23.30	22.08	23.95
High SDI	Female	Colon and rectum cancer	2005		15.60	14.21	16.30
High SDI	Both	Colon and rectum cancer	2005		18.97	17.64	19.65
High-middle SDI	Male	Colon and rectum cancer	2005		22.10	21.14	22.99
High-middle SDI	Female	Colon and rectum cancer	2005		14.26	13.35	14.86
High-middle SDI	Both	Colon and rectum cancer	2005		17.51	16.59	18.11
Low SDI	Male	Colon and rectum cancer	2005		7.07	6.18	8.56
Low SDI	Female	Colon and rectum cancer	2005		5.99	5.11	6.88
Low SDI	Both	Colon and rectum cancer	2005		6.52	5.76	7.56
Low-middle SDI	Male	Colon and rectum cancer	2005		8.14	7.60	8.88
Low-middle SDI	Female	Colon and rectum cancer	2005		7.27	6.57	7.94
Low-middle SDI	Both	Colon and rectum cancer	2005		7.69	7.17	8.23
Middle SDI	Male	Colon and rectum cancer	2005		12.96	12.07	14.02
Middle SDI	Female	Colon and rectum cancer	2005		9.47	8.68	10.24
Middle SDI	Both	Colon and rectum cancer	2005		11.14	10.40	11.91
High SDI	Male	Colon and rectum cancer	2006		22.84	21.64	23.51
High SDI	Female	Colon and rectum cancer	2006		15.24	13.84	15.98
High SDI	Both	Colon and rectum cancer	2006		18.59	17.26	19.29
High-middle SDI	Male	Colon and rectum cancer	2006		21.74	20.75	22.58
High-middle SDI	Female	Colon and rectum cancer	2006		13.95	13.00	14.54
High-middle SDI	Both	Colon and rectum cancer	2006		17.18	16.24	17.75
Low SDI	Male	Colon and rectum cancer	2006		7.09	6.23	8.53
Low SDI	Female	Colon and rectum cancer	2006		6.04	5.19	6.90
Low SDI	Both	Colon and rectum cancer	2006		6.55	5.80	7.55
Low-middle SDI	Male	Colon and rectum cancer	2006		8.28	7.73	9.03
Low-middle SDI	Female	Colon and rectum cancer	2006		7.34	6.62	8.01
Low-middle SDI	Both	Colon and rectum cancer	2006		7.80	7.28	8.36
Middle SDI	Male	Colon and rectum cancer	2006		13.01	12.12	13.96
Middle SDI	Female	Colon and rectum cancer	2006		9.48	8.69	10.19
Middle SDI	Both	Colon and rectum cancer	2006		11.16	10.49	11.88
High SDI	Male	Colon and rectum cancer	2007		22.54	21.31	23.20
High SDI	Female	Colon and rectum cancer	2007		14.95	13.54	15.71

High SDI	Both	Colon and rectum cancer	2007		18.30	16.96	19.02
High-middle SDI	Male	Colon and rectum cancer	2007		21.75	20.70	22.58
High-middle SDI	Female	Colon and rectum cancer	2007		13.86	12.88	14.46
High-middle SDI	Both	Colon and rectum cancer	2007		17.14	16.21	17.73
Low SDI	Male	Colon and rectum cancer	2007		7.18	6.29	8.56
Low SDI	Female	Colon and rectum cancer	2007		6.03	5.15	6.91
Low SDI	Both	Colon and rectum cancer	2007		6.59	5.84	7.59
Low-middle SDI	Male	Colon and rectum cancer	2007		8.40	7.83	9.09
Low-middle SDI	Female	Colon and rectum cancer	2007		7.38	6.67	8.06
Low-middle SDI	Both	Colon and rectum cancer	2007		7.87	7.34	8.42
Middle SDI	Male	Colon and rectum cancer	2007		13.19	12.27	14.15
Middle SDI	Female	Colon and rectum cancer	2007		9.50	8.76	10.16
Middle SDI	Both	Colon and rectum cancer	2007		11.26	10.53	11.96
High SDI	Male	Colon and rectum cancer	2008		22.31	21.09	22.97
High SDI	Female	Colon and rectum cancer	2008		14.71	13.29	15.48
High SDI	Both	Colon and rectum cancer	2008		18.07	16.74	18.78
High-middle SDI	Male	Colon and rectum cancer	2008		21.92	20.87	22.79
High-middle SDI	Female	Colon and rectum cancer	2008		13.84	12.87	14.47
High-middle SDI	Both	Colon and rectum cancer	2008		17.21	16.29	17.83
Low SDI	Male	Colon and rectum cancer	2008		7.21	6.35	8.54
Low SDI	Female	Colon and rectum cancer	2008		6.07	5.21	6.95
Low SDI	Both	Colon and rectum cancer	2008		6.63	5.87	7.61
Low-middle SDI	Male	Colon and rectum cancer	2008		8.58	8.02	9.27
Low-middle SDI	Female	Colon and rectum cancer	2008		7.41	6.73	8.10
Low-middle SDI	Both	Colon and rectum cancer	2008		7.98	7.47	8.52
Middle SDI	Male	Colon and rectum cancer	2008		13.51	12.60	14.52
Middle SDI	Female	Colon and rectum cancer	2008		9.57	8.77	10.22
Middle SDI	Both	Colon and rectum cancer	2008		11.44	10.69	12.15
High SDI	Male	Colon and rectum cancer	2009		21.96	20.70	22.59
High SDI	Female	Colon and rectum cancer	2009		14.35	12.95	15.10
High SDI	Both	Colon and rectum cancer	2009		17.73	16.40	18.42
High-middle SDI	Male	Colon and rectum cancer	2009		21.84	20.75	22.74
High-middle SDI	Female	Colon and rectum cancer	2009		13.68	12.72	14.35
High-middle SDI	Both	Colon and rectum cancer	2009		17.08	16.10	17.73
Low SDI	Male	Colon and rectum cancer	2009		7.25	6.41	8.57
Low SDI	Female	Colon and rectum cancer	2009		6.09	5.28	6.99
Low SDI	Both	Colon and rectum cancer	2009		6.65	5.95	7.61
Low-middle SDI	Male	Colon and rectum cancer	2009		8.61	7.99	9.29
Low-middle SDI	Female	Colon and rectum cancer	2009		7.36	6.72	7.95
Low-middle SDI	Both	Colon and rectum cancer	2009		7.97	7.42	8.49
Middle SDI	Male	Colon and rectum cancer	2009		13.84	12.93	14.86
Middle SDI	Female	Colon and rectum cancer	2009		9.63	8.81	10.30
Middle SDI	Both	Colon and rectum cancer	2009		11.63	10.84	12.29
High SDI	Male	Colon and rectum cancer	2010		21.55	20.36	22.19
High SDI	Female	Colon and rectum cancer	2010		13.98	12.56	14.72
High SDI	Both	Colon and rectum cancer	2010		17.34	16.01	18.03
High-middle SDI	Male	Colon and rectum cancer	2010		22.00	20.81	22.97
High-middle SDI	Female	Colon and rectum cancer	2010		13.60	12.64	14.29
High-middle SDI	Both	Colon and rectum cancer	2010		17.11	16.15	17.76
Low SDI	Male	Colon and rectum cancer	2010		7.25	6.40	8.52
Low SDI	Female	Colon and rectum cancer	2010		6.08	5.27	6.94
Low SDI	Both	Colon and rectum cancer	2010		6.65	5.93	7.55

Low-middle SDI	Male	Colon and rectum cancer	2010	8.67	8.12	9.32
Low-middle SDI	Female	Colon and rectum cancer	2010	7.37	6.65	7.96
Low-middle SDI	Both	Colon and rectum cancer	2010	8.00	7.48	8.50
Middle SDI	Male	Colon and rectum cancer	2010	14.14	13.16	15.15
Middle SDI	Female	Colon and rectum cancer	2010	9.68	8.84	10.41
Middle SDI	Both	Colon and rectum cancer	2010	11.79	11.01	12.51
High SDI	Male	Colon and rectum cancer	2011	21.25	20.00	21.91
High SDI	Female	Colon and rectum cancer	2011	13.79	12.40	14.51
High SDI	Both	Colon and rectum cancer	2011	17.12	15.80	17.79
High-middle SDI	Male	Colon and rectum cancer	2011	21.78	20.55	22.81
High-middle SDI	Female	Colon and rectum cancer	2011	13.37	12.36	14.09
High-middle SDI	Both	Colon and rectum cancer	2011	16.88	15.82	17.62
Low SDI	Male	Colon and rectum cancer	2011	7.30	6.48	8.45
Low SDI	Female	Colon and rectum cancer	2011	6.12	5.34	6.97
Low SDI	Both	Colon and rectum cancer	2011	6.69	6.00	7.55
Low-middle SDI	Male	Colon and rectum cancer	2011	8.71	8.11	9.36
Low-middle SDI	Female	Colon and rectum cancer	2011	7.43	6.73	8.06
Low-middle SDI	Both	Colon and rectum cancer	2011	8.05	7.51	8.61
Middle SDI	Male	Colon and rectum cancer	2011	14.23	13.07	15.46
Middle SDI	Female	Colon and rectum cancer	2011	9.66	8.80	10.38
Middle SDI	Both	Colon and rectum cancer	2011	11.82	10.95	12.63
High SDI	Male	Colon and rectum cancer	2012	20.92	19.72	21.57
High SDI	Female	Colon and rectum cancer	2012	13.55	12.18	14.28
High SDI	Both	Colon and rectum cancer	2012	16.85	15.55	17.52
High-middle SDI	Male	Colon and rectum cancer	2012	21.71	20.56	22.81
High-middle SDI	Female	Colon and rectum cancer	2012	13.14	12.11	13.80
High-middle SDI	Both	Colon and rectum cancer	2012	16.72	15.73	17.40
Low SDI	Male	Colon and rectum cancer	2012	7.37	6.54	8.53
Low SDI	Female	Colon and rectum cancer	2012	6.25	5.48	7.10
Low SDI	Both	Colon and rectum cancer	2012	6.79	6.14	7.64
Low-middle SDI	Male	Colon and rectum cancer	2012	8.79	8.21	9.44
Low-middle SDI	Female	Colon and rectum cancer	2012	7.51	6.85	8.09
Low-middle SDI	Both	Colon and rectum cancer	2012	8.13	7.62	8.64
Middle SDI	Male	Colon and rectum cancer	2012	14.28	13.19	15.51
Middle SDI	Female	Colon and rectum cancer	2012	9.57	8.78	10.26
Middle SDI	Both	Colon and rectum cancer	2012	11.79	10.97	12.60
High SDI	Male	Colon and rectum cancer	2013	20.60	19.39	21.25
High SDI	Female	Colon and rectum cancer	2013	13.34	11.97	14.06
High SDI	Both	Colon and rectum cancer	2013	16.59	15.29	17.28
High-middle SDI	Male	Colon and rectum cancer	2013	21.40	20.11	22.48
High-middle SDI	Female	Colon and rectum cancer	2013	12.90	11.83	13.55
High-middle SDI	Both	Colon and rectum cancer	2013	16.46	15.37	17.19
Low SDI	Male	Colon and rectum cancer	2013	7.51	6.69	8.53
Low SDI	Female	Colon and rectum cancer	2013	6.36	5.55	7.15
Low SDI	Both	Colon and rectum cancer	2013	6.92	6.20	7.70
Low-middle SDI	Male	Colon and rectum cancer	2013	8.99	8.40	9.66
Low-middle SDI	Female	Colon and rectum cancer	2013	7.68	6.92	8.34
Low-middle SDI	Both	Colon and rectum cancer	2013	8.31	7.78	8.84
Middle SDI	Male	Colon and rectum cancer	2013	14.26	13.13	15.46
Middle SDI	Female	Colon and rectum cancer	2013	9.53	8.64	10.26
Middle SDI	Both	Colon and rectum cancer	2013	11.76	10.90	12.58
High SDI	Male	Colon and rectum cancer	2014	20.21	18.97	20.86

High SDI	Female	Colon and rectum cancer	2014	13.14	11.78	13.86
High SDI	Both	Colon and rectum cancer	2014	16.31	15.00	17.02
High-middle SDI	Male	Colon and rectum cancer	2014	21.23	19.99	22.41
High-middle SDI	Female	Colon and rectum cancer	2014	12.74	11.74	13.44
High-middle SDI	Both	Colon and rectum cancer	2014	16.31	15.32	17.07
Low SDI	Male	Colon and rectum cancer	2014	7.49	6.67	8.59
Low SDI	Female	Colon and rectum cancer	2014	6.48	5.66	7.26
Low SDI	Both	Colon and rectum cancer	2014	6.97	6.27	7.73
Low-middle SDI	Male	Colon and rectum cancer	2014	9.12	8.45	9.81
Low-middle SDI	Female	Colon and rectum cancer	2014	7.87	7.08	8.54
Low-middle SDI	Both	Colon and rectum cancer	2014	8.47	7.92	9.03
Middle SDI	Male	Colon and rectum cancer	2014	14.23	13.06	15.50
Middle SDI	Female	Colon and rectum cancer	2014	9.50	8.66	10.24
Middle SDI	Both	Colon and rectum cancer	2014	11.73	10.84	12.57
High SDI	Male	Colon and rectum cancer	2015	20.10	18.89	20.78
High SDI	Female	Colon and rectum cancer	2015	13.07	11.67	13.80
High SDI	Both	Colon and rectum cancer	2015	16.24	14.91	16.96
High-middle SDI	Male	Colon and rectum cancer	2015	21.39	20.09	22.60
High-middle SDI	Female	Colon and rectum cancer	2015	12.75	11.69	13.46
High-middle SDI	Both	Colon and rectum cancer	2015	16.39	15.33	17.13
Low SDI	Male	Colon and rectum cancer	2015	7.55	6.72	8.52
Low SDI	Female	Colon and rectum cancer	2015	6.54	5.74	7.31
Low SDI	Both	Colon and rectum cancer	2015	7.03	6.36	7.79
Low-middle SDI	Male	Colon and rectum cancer	2015	9.16	8.50	9.94
Low-middle SDI	Female	Colon and rectum cancer	2015	8.07	7.23	8.86
Low-middle SDI	Both	Colon and rectum cancer	2015	8.59	7.99	9.22
Middle SDI	Male	Colon and rectum cancer	2015	14.19	12.95	15.46
Middle SDI	Female	Colon and rectum cancer	2015	9.44	8.56	10.22
Middle SDI	Both	Colon and rectum cancer	2015	11.67	10.79	12.52
High SDI	Male	Colon and rectum cancer	2016	19.96	18.67	20.68
High SDI	Female	Colon and rectum cancer	2016	13.02	11.67	13.77
High SDI	Both	Colon and rectum cancer	2016	16.16	14.82	16.87
High-middle SDI	Male	Colon and rectum cancer	2016	21.26	19.81	22.63
High-middle SDI	Female	Colon and rectum cancer	2016	12.61	11.52	13.41
High-middle SDI	Both	Colon and rectum cancer	2016	16.26	15.11	17.16
Low SDI	Male	Colon and rectum cancer	2016	7.56	6.72	8.61
Low SDI	Female	Colon and rectum cancer	2016	6.60	5.79	7.40
Low SDI	Both	Colon and rectum cancer	2016	7.06	6.33	7.88
Low-middle SDI	Male	Colon and rectum cancer	2016	9.23	8.51	10.06
Low-middle SDI	Female	Colon and rectum cancer	2016	8.17	7.29	9.01
Low-middle SDI	Both	Colon and rectum cancer	2016	8.68	7.99	9.34
Middle SDI	Male	Colon and rectum cancer	2016	14.22	12.84	15.61
Middle SDI	Female	Colon and rectum cancer	2016	9.44	8.51	10.34
Middle SDI	Both	Colon and rectum cancer	2016	11.68	10.67	12.65
High SDI	Male	Colon and rectum cancer	2017	19.88	18.66	20.67
High SDI	Female	Colon and rectum cancer	2017	12.94	11.56	13.71
High SDI	Both	Colon and rectum cancer	2017	16.08	14.75	16.80
High-middle SDI	Male	Colon and rectum cancer	2017	21.03	19.50	22.53
High-middle SDI	Female	Colon and rectum cancer	2017	12.48	11.35	13.38
High-middle SDI	Both	Colon and rectum cancer	2017	16.11	15.01	17.01
Low SDI	Male	Colon and rectum cancer	2017	7.61	6.72	8.69
Low SDI	Female	Colon and rectum cancer	2017	6.66	5.85	7.48

Low SDI	Both	Colon and rectum cancer	2017	7.12	6.37	7.91
Low-middle SDI	Male	Colon and rectum cancer	2017	9.36	8.53	10.22
Low-middle SDI	Female	Colon and rectum cancer	2017	8.25	7.28	9.17
Low-middle SDI	Both	Colon and rectum cancer	2017	8.78	7.99	9.52
Middle SDI	Male	Colon and rectum cancer	2017	14.27	12.83	15.78
Middle SDI	Female	Colon and rectum cancer	2017	9.47	8.40	10.41
Middle SDI	Both	Colon and rectum cancer	2017	11.72	10.72	12.67
High SDI	Male	Colon and rectum cancer	2018	20.01	18.72	20.88
High SDI	Female	Colon and rectum cancer	2018	13.02	11.60	13.82
High SDI	Both	Colon and rectum cancer	2018	16.19	14.86	16.96
High-middle SDI	Male	Colon and rectum cancer	2018	21.02	19.33	22.80
High-middle SDI	Female	Colon and rectum cancer	2018	12.55	11.41	13.53
High-middle SDI	Both	Colon and rectum cancer	2018	16.15	15.06	17.21
Low SDI	Male	Colon and rectum cancer	2018	7.75	6.82	8.80
Low SDI	Female	Colon and rectum cancer	2018	6.79	5.93	7.64
Low SDI	Both	Colon and rectum cancer	2018	7.25	6.50	8.03
Low-middle SDI	Male	Colon and rectum cancer	2018	9.57	8.59	10.66
Low-middle SDI	Female	Colon and rectum cancer	2018	8.38	7.32	9.40
Low-middle SDI	Both	Colon and rectum cancer	2018	8.95	8.09	9.82
Middle SDI	Male	Colon and rectum cancer	2018	14.45	12.90	16.07
Middle SDI	Female	Colon and rectum cancer	2018	9.56	8.48	10.62
Middle SDI	Both	Colon and rectum cancer	2018	11.85	10.65	12.95
High SDI	Male	Colon and rectum cancer	2019	20.08	18.75	20.97
High SDI	Female	Colon and rectum cancer	2019	13.14	11.72	13.98
High SDI	Both	Colon and rectum cancer	2019	16.29	14.93	17.09
High-middle SDI	Male	Colon and rectum cancer	2019	21.12	19.19	22.90
High-middle SDI	Female	Colon and rectum cancer	2019	12.61	11.37	13.74
High-middle SDI	Both	Colon and rectum cancer	2019	16.24	14.90	17.39
Low SDI	Male	Colon and rectum cancer	2019	7.83	6.86	8.98
Low SDI	Female	Colon and rectum cancer	2019	6.87	5.97	7.73
Low SDI	Both	Colon and rectum cancer	2019	7.33	6.53	8.14
Low-middle SDI	Male	Colon and rectum cancer	2019	9.72	8.73	10.78
Low-middle SDI	Female	Colon and rectum cancer	2019	8.49	7.40	9.64
Low-middle SDI	Both	Colon and rectum cancer	2019	9.08	8.22	9.94
Middle SDI	Male	Colon and rectum cancer	2019	14.64	12.92	16.53
Middle SDI	Female	Colon and rectum cancer	2019	9.63	8.39	10.83
Middle SDI	Both	Colon and rectum cancer	2019	11.98	10.76	13.09

location_name	sex_name	cause_name	year	Age-standardised incidence rate (per 100 000 person-years)	95% CI (lower)	95% CI (upper)
Afghanistan	Both	Colon and rectum cancer	2019	8.69	6.04	11.55
Albania	Both	Colon and rectum cancer	2019	15.15	11.40	19.90
Algeria	Both	Colon and rectum cancer	2019	10.52	8.35	13.05
American Samoa	Both	Colon and rectum cancer	2019	20.81	17.56	24.70
Andorra	Both	Colon and rectum cancer	2019	56.65	42.79	71.90
Angola	Both	Colon and rectum cancer	2019	10.02	8.06	12.55
Antigua and Barbuda	Both	Colon and rectum cancer	2019	25.58	22.05	29.43
Argentina	Both	Colon and rectum cancer	2019	34.68	27.56	43.36
Armenia	Both	Colon and rectum cancer	2019	21.37	17.85	25.01
Australia	Both	Colon and rectum cancer	2019	47.04	36.95	59.55
Austria	Both	Colon and rectum cancer	2019	33.07	26.71	40.55
Azerbaijan	Both	Colon and rectum cancer	2019	16.32	13.28	20.08
Bahamas	Both	Colon and rectum cancer	2019	27.96	22.83	34.01
Bahrain	Both	Colon and rectum cancer	2019	16.96	13.23	21.08
Bangladesh	Both	Colon and rectum cancer	2019	5.63	3.90	8.00
Barbados	Both	Colon and rectum cancer	2019	39.83	33.03	47.38
Belarus	Both	Colon and rectum cancer	2019	34.12	27.11	43.36
Belgium	Both	Colon and rectum cancer	2019	39.33	30.86	49.46
Belize	Both	Colon and rectum cancer	2019	14.64	12.76	16.95
Benin	Both	Colon and rectum cancer	2019	7.78	6.25	9.85
Bermuda	Both	Colon and rectum cancer	2019	43.29	36.11	52.78
Bhutan	Both	Colon and rectum cancer	2019	8.03	4.91	10.87
Bolivia (Plurinational State of)	Both	Colon and rectum cancer	2019	16.89	11.60	22.44
Bosnia and Herzegovina	Both	Colon and rectum cancer	2019	34.91	27.54	43.53
Botswana	Both	Colon and rectum cancer	2019	18.75	13.48	24.55
Brazil	Both	Colon and rectum cancer	2019	17.77	16.64	18.65
Brunei Darussalam	Both	Colon and rectum cancer	2019	49.38	43.44	55.88
Bulgaria	Both	Colon and rectum cancer	2019	43.84	35.01	54.21
Burkina Faso	Both	Colon and rectum cancer	2019	7.42	5.92	9.38
Burundi	Both	Colon and rectum cancer	2019	7.35	5.32	10.43
Cabo Verde	Both	Colon and rectum cancer	2019	13.39	10.67	15.73
Cambodia	Both	Colon and rectum cancer	2019	16.67	13.29	20.00
Cameroon	Both	Colon and rectum cancer	2019	11.18	8.58	14.61
Canada	Both	Colon and rectum cancer	2019	49.60	38.84	62.84
Central African Republic	Both	Colon and rectum cancer	2019	6.31	4.65	8.68
Chad	Both	Colon and rectum cancer	2019	7.32	5.68	9.41
Chile	Both	Colon and rectum cancer	2019	25.10	20.08	31.56
China	Both	Colon and rectum cancer	2019	30.55	26.37	35.50
Colombia	Both	Colon and rectum cancer	2019	17.15	13.28	21.89
Comoros	Both	Colon and rectum cancer	2019	8.96	6.63	11.43
Congo	Both	Colon and rectum cancer	2019	11.94	9.05	15.43
Cook Islands	Both	Colon and rectum cancer	2019	12.42	10.26	15.12
Costa Rica	Both	Colon and rectum cancer	2019	29.18	22.72	37.17
Côte d'Ivoire	Both	Colon and rectum cancer	2019	9.59	7.65	11.87
Croatia	Both	Colon and rectum cancer	2019	50.47	39.91	62.58
Cuba	Both	Colon and rectum cancer	2019	34.57	28.25	42.05
Cyprus	Both	Colon and rectum cancer	2019	38.15	33.10	43.43
Czechia	Both	Colon and rectum cancer	2019	42.14	34.45	51.15
Democratic People's Republic of Korea	Both	Colon and rectum cancer	2019	15.42	11.47	19.91
Democratic Republic of the Congo	Both	Colon and rectum cancer	2019	6.36	4.24	9.58
Denmark	Both	Colon and rectum cancer	2019	48.41	37.76	61.15
Djibouti	Both	Colon and rectum cancer	2019	11.92	9.06	15.80
Dominica	Both	Colon and rectum cancer	2019	19.85	16.29	24.15
Dominican Republic	Both	Colon and rectum cancer	2019	17.08	12.37	22.31
Ecuador	Both	Colon and rectum cancer	2019	19.25	15.35	24.31
Egypt	Both	Colon and rectum cancer	2019	9.78	7.07	13.43
El Salvador	Both	Colon and rectum cancer	2019	14.12	10.72	18.33
Equatorial Guinea	Both	Colon and rectum cancer	2019	15.60	9.84	22.43
Eritrea	Both	Colon and rectum cancer	2019	10.34	8.12	13.21
Estonia	Both	Colon and rectum cancer	2019	42.21	33.40	52.75
Eswatini	Both	Colon and rectum cancer	2019	14.40	9.82	19.67
Ethiopia	Both	Colon and rectum cancer	2019	7.70	5.78	10.75

Fiji	Both	Colon and rectum cancer	2019	13.52	10.86	16.56
Finland	Both	Colon and rectum cancer	2019	31.46	24.55	40.00
France	Both	Colon and rectum cancer	2019	38.47	30.10	49.30
Gabon	Both	Colon and rectum cancer	2019	16.38	12.14	20.32
Gambia	Both	Colon and rectum cancer	2019	6.84	4.99	9.15
Georgia	Both	Colon and rectum cancer	2019	19.13	16.02	22.48
Germany	Both	Colon and rectum cancer	2019	41.40	32.67	53.84
Ghana	Both	Colon and rectum cancer	2019	9.51	7.57	11.94
Greece	Both	Colon and rectum cancer	2019	33.21	26.10	42.09
Greenland	Both	Colon and rectum cancer	2019	47.44	39.57	55.72
Grenada	Both	Colon and rectum cancer	2019	27.86	25.13	30.69
Guam	Both	Colon and rectum cancer	2019	21.01	17.62	24.81
Guatemala	Both	Colon and rectum cancer	2019	11.51	9.18	14.40
Guinea	Both	Colon and rectum cancer	2019	7.30	5.56	9.43
Guinea-Bissau	Both	Colon and rectum cancer	2019	9.32	7.08	11.68
Guyana	Both	Colon and rectum cancer	2019	18.71	14.85	23.58
Haiti	Both	Colon and rectum cancer	2019	11.45	7.97	15.43
Honduras	Both	Colon and rectum cancer	2019	9.67	6.74	13.58
Hungary	Both	Colon and rectum cancer	2019	52.24	42.96	62.90
Iceland	Both	Colon and rectum cancer	2019	30.71	26.82	35.36
India	Both	Colon and rectum cancer	2019	8.59	7.22	9.94
Indonesia	Both	Colon and rectum cancer	2019	18.53	12.63	23.42
Iran (Islamic Republic of)	Both	Colon and rectum cancer	2019	13.88	12.76	15.07
Iraq	Both	Colon and rectum cancer	2019	11.13	8.73	13.83
Ireland	Both	Colon and rectum cancer	2019	45.95	35.89	57.72
Israel	Both	Colon and rectum cancer	2019	33.64	26.30	42.59
Italy	Both	Colon and rectum cancer	2019	43.54	35.99	51.56
Jamaica	Both	Colon and rectum cancer	2019	25.95	20.82	32.26
Japan	Both	Colon and rectum cancer	2019	47.59	40.18	55.59
Jordan	Both	Colon and rectum cancer	2019	19.14	15.96	22.86
Kazakhstan	Both	Colon and rectum cancer	2019	19.72	17.18	22.45
Kenya	Both	Colon and rectum cancer	2019	8.24	6.68	10.05
Kiribati	Both	Colon and rectum cancer	2019	11.45	8.70	15.08
Kuwait	Both	Colon and rectum cancer	2019	17.78	14.65	21.32
Kyrgyzstan	Both	Colon and rectum cancer	2019	10.52	9.21	11.99
Lao People's Democratic Republic	Both	Colon and rectum cancer	2019	14.90	10.64	19.54
Latvia	Both	Colon and rectum cancer	2019	30.54	25.44	36.96
Lebanon	Both	Colon and rectum cancer	2019	29.83	24.07	38.13
Lesotho	Both	Colon and rectum cancer	2019	12.01	8.83	15.54
Liberia	Both	Colon and rectum cancer	2019	6.84	4.66	9.71
Libya	Both	Colon and rectum cancer	2019	17.00	12.41	21.83
Lithuania	Both	Colon and rectum cancer	2019	29.20	24.01	35.37
Luxembourg	Both	Colon and rectum cancer	2019	37.17	30.27	45.07
Madagascar	Both	Colon and rectum cancer	2019	7.33	5.41	9.73
Malawi	Both	Colon and rectum cancer	2019	6.27	4.87	7.80
Malaysia	Both	Colon and rectum cancer	2019	29.57	23.27	36.50
Maldives	Both	Colon and rectum cancer	2019	13.80	11.28	16.40
Mali	Both	Colon and rectum cancer	2019	8.11	6.43	10.13
Malta	Both	Colon and rectum cancer	2019	32.92	27.83	39.12
Marshall Islands	Both	Colon and rectum cancer	2019	13.71	10.51	17.43
Mauritania	Both	Colon and rectum cancer	2019	8.84	6.80	11.08
Mauritius	Both	Colon and rectum cancer	2019	19.77	16.12	24.16
Mexico	Both	Colon and rectum cancer	2019	14.92	12.88	17.11
Micronesia (Federated States of)	Both	Colon and rectum cancer	2019	15.36	10.93	20.00
Monaco	Both	Colon and rectum cancer	2019	60.69	48.55	73.57
Mongolia	Both	Colon and rectum cancer	2019	11.09	8.77	14.12
Montenegro	Both	Colon and rectum cancer	2019	30.47	24.96	36.76
Morocco	Both	Colon and rectum cancer	2019	10.34	7.70	13.02
Mozambique	Both	Colon and rectum cancer	2019	8.73	6.43	11.37
Myanmar	Both	Colon and rectum cancer	2019	15.03	11.10	19.26
Namibia	Both	Colon and rectum cancer	2019	9.74	7.69	12.25
Nauru	Both	Colon and rectum cancer	2019	21.43	14.78	27.76
Nepal	Both	Colon and rectum cancer	2019	5.88	4.22	8.15
Netherlands	Both	Colon and rectum cancer	2019	55.39	43.29	69.76

New Zealand	Both	Colon and rectum cancer	2019	55.34	45.72	65.79
Nicaragua	Both	Colon and rectum cancer	2019	16.17	13.44	19.03
Niger	Both	Colon and rectum cancer	2019	5.63	4.19	7.56
Nigeria	Both	Colon and rectum cancer	2019	8.95	6.87	11.02
Niue	Both	Colon and rectum cancer	2019	20.40	15.71	26.06
North Macedonia	Both	Colon and rectum cancer	2019	35.36	28.19	43.94
Northern Mariana Islands	Both	Colon and rectum cancer	2019	28.86	24.45	33.06
Norway	Both	Colon and rectum cancer	2019	49.46	41.90	57.73
Oman	Both	Colon and rectum cancer	2019	15.32	12.63	18.56
Pakistan	Both	Colon and rectum cancer	2019	9.14	7.33	11.63
Palau	Both	Colon and rectum cancer	2019	19.94	15.70	24.69
Palestine	Both	Colon and rectum cancer	2019	26.12	22.15	30.27
Panama	Both	Colon and rectum cancer	2019	18.61	14.45	23.57
Papua New Guinea	Both	Colon and rectum cancer	2019	8.06	5.88	10.54
Paraguay	Both	Colon and rectum cancer	2019	17.37	13.38	22.12
Peru	Both	Colon and rectum cancer	2019	21.24	15.91	27.77
Philippines	Both	Colon and rectum cancer	2019	18.93	15.42	23.14
Poland	Both	Colon and rectum cancer	2019	34.96	29.84	41.55
Portugal	Both	Colon and rectum cancer	2019	45.29	35.23	57.72
Puerto Rico	Both	Colon and rectum cancer	2019	34.99	26.99	44.78
Qatar	Both	Colon and rectum cancer	2019	25.05	19.33	31.85
Republic of Korea	Both	Colon and rectum cancer	2019	37.16	31.14	44.01
Republic of Moldova	Both	Colon and rectum cancer	2019	29.17	25.39	33.14
Romania	Both	Colon and rectum cancer	2019	36.16	29.52	43.30
Russian Federation	Both	Colon and rectum cancer	2019	30.77	27.02	35.11
Rwanda	Both	Colon and rectum cancer	2019	9.32	7.43	11.66
Saint Kitts and Nevis	Both	Colon and rectum cancer	2019	31.11	26.32	36.41
Saint Lucia	Both	Colon and rectum cancer	2019	17.96	15.15	21.20
Saint Vincent and the Grenadines	Both	Colon and rectum cancer	2019	19.66	17.20	22.56
Samoa	Both	Colon and rectum cancer	2019	12.76	10.27	15.68
San Marino	Both	Colon and rectum cancer	2019	49.55	37.89	65.53
Sao Tome and Principe	Both	Colon and rectum cancer	2019	16.46	12.02	22.22
Saudi Arabia	Both	Colon and rectum cancer	2019	15.36	12.16	18.83
Senegal	Both	Colon and rectum cancer	2019	8.92	7.17	11.08
Serbia	Both	Colon and rectum cancer	2019	43.55	35.07	54.26
Seychelles	Both	Colon and rectum cancer	2019	35.70	31.42	40.58
Sierra Leone	Both	Colon and rectum cancer	2019	7.09	5.48	9.11
Singapore	Both	Colon and rectum cancer	2019	39.93	31.93	49.46
Slovakia	Both	Colon and rectum cancer	2019	56.45	44.36	71.04
Slovenia	Both	Colon and rectum cancer	2019	41.05	31.88	52.95
Solomon Islands	Both	Colon and rectum cancer	2019	12.36	8.44	16.09
Somalia	Both	Colon and rectum cancer	2019	4.95	3.13	9.24
South Africa	Both	Colon and rectum cancer	2019	12.88	11.62	14.50
South Sudan	Both	Colon and rectum cancer	2019	9.92	6.56	14.66
Spain	Both	Colon and rectum cancer	2019	50.14	39.34	63.84
Sri Lanka	Both	Colon and rectum cancer	2019	10.18	7.63	13.19
Sudan	Both	Colon and rectum cancer	2019	8.22	5.99	12.28
Suriname	Both	Colon and rectum cancer	2019	21.61	17.85	25.57
Sweden	Both	Colon and rectum cancer	2019	36.99	31.41	42.78
Switzerland	Both	Colon and rectum cancer	2019	33.59	25.91	42.71
Syrian Arab Republic	Both	Colon and rectum cancer	2019	8.54	6.26	11.28
Taiwan (Province of China)	Both	Colon and rectum cancer	2019	62.05	48.91	80.05
Tajikistan	Both	Colon and rectum cancer	2019	11.62	9.50	14.05
Thailand	Both	Colon and rectum cancer	2019	17.18	12.71	22.51
Timor-Leste	Both	Colon and rectum cancer	2019	13.91	9.69	17.85
Togo	Both	Colon and rectum cancer	2019	8.22	6.14	10.53
Tokelau	Both	Colon and rectum cancer	2019	14.11	10.54	18.35
Tonga	Both	Colon and rectum cancer	2019	8.28	6.45	10.46
Trinidad and Tobago	Both	Colon and rectum cancer	2019	21.07	16.12	27.06
Tunisia	Both	Colon and rectum cancer	2019	14.49	10.54	19.50
Turkey	Both	Colon and rectum cancer	2019	20.56	16.42	25.01
Turkmenistan	Both	Colon and rectum cancer	2019	9.19	7.47	11.33
Tuvalu	Both	Colon and rectum cancer	2019	13.03	9.60	16.87
Uganda	Both	Colon and rectum cancer	2019	12.31	9.77	14.83

Ukraine	Both	Colon and rectum cancer	2019	31.27	26.56	36.54
United Arab Emirates	Both	Colon and rectum cancer	2019	21.36	14.70	29.76
United Kingdom	Both	Colon and rectum cancer	2019	43.60	36.60	51.50
United Republic of Tanzania	Both	Colon and rectum cancer	2019	10.10	8.13	12.70
United States of America	Both	Colon and rectum cancer	2019	41.86	36.15	48.20
United States Virgin Islands	Both	Colon and rectum cancer	2019	43.04	35.69	50.62
Uruguay	Both	Colon and rectum cancer	2019	39.49	31.56	49.54
Uzbekistan	Both	Colon and rectum cancer	2019	12.82	11.10	14.70
Vanuatu	Both	Colon and rectum cancer	2019	10.10	7.56	13.08
Venezuela (Bolivarian Republic of)	Both	Colon and rectum cancer	2019	18.58	14.14	24.22
Viet Nam	Both	Colon and rectum cancer	2019	26.36	20.58	32.37
Yemen	Both	Colon and rectum cancer	2019	7.39	5.56	9.99
Zambia	Both	Colon and rectum cancer	2019	13.62	10.14	17.44
Zimbabwe	Both	Colon and rectum cancer	2019	13.76	10.57	17.23

location_name	sex_name	cause_name	year	Age-standardised death rate (per 100 000 person-years)	95% CI (lower)	95% CI (upper)
Afghanistan	Both	Colon and rectum cancer	2019	8.43	6.01	11.18
Albania	Both	Colon and rectum cancer	2019	9.15	6.96	11.89
Algeria	Both	Colon and rectum cancer	2019	8.05	6.40	9.77
American Samoa	Both	Colon and rectum cancer	2019	16.55	14.07	19.59
Andorra	Both	Colon and rectum cancer	2019	22.97	17.88	28.86
Angola	Both	Colon and rectum cancer	2019	9.65	7.82	11.99
Antigua and Barbuda	Both	Colon and rectum cancer	2019	16.16	14.01	18.45
Argentina	Both	Colon and rectum cancer	2019	23.59	22.00	25.13
Armenia	Both	Colon and rectum cancer	2019	14.30	12.07	16.63
Australia	Both	Colon and rectum cancer	2019	15.49	14.08	16.66
Austria	Both	Colon and rectum cancer	2019	13.54	12.36	14.54
Azerbaijan	Both	Colon and rectum cancer	2019	12.20	9.77	15.16
Bahamas	Both	Colon and rectum cancer	2019	18.41	15.08	22.19
Bahrain	Both	Colon and rectum cancer	2019	11.42	9.08	14.03
Bangladesh	Both	Colon and rectum cancer	2019	4.94	3.40	7.07
Barbados	Both	Colon and rectum cancer	2019	23.96	20.09	28.31
Belarus	Both	Colon and rectum cancer	2019	17.03	13.86	21.48
Belgium	Both	Colon and rectum cancer	2019	17.10	15.59	18.44
Belize	Both	Colon and rectum cancer	2019	10.42	9.02	11.99
Benin	Both	Colon and rectum cancer	2019	7.63	6.19	9.53
Bermuda	Both	Colon and rectum cancer	2019	18.81	15.73	22.69
Bhutan	Both	Colon and rectum cancer	2019	6.97	4.39	9.42
Bolivia (Plurinational State of)	Both	Colon and rectum cancer	2019	12.26	8.43	16.20
Bosnia and Herzegovina	Both	Colon and rectum cancer	2019	22.32	17.81	27.66
Botswana	Both	Colon and rectum cancer	2019	15.75	11.59	20.48
Brazil	Both	Colon and rectum cancer	2019	11.67	10.81	12.28
Brunei Darussalam	Both	Colon and rectum cancer	2019	30.26	26.58	34.10
Bulgaria	Both	Colon and rectum cancer	2019	25.11	20.41	30.62
Burkina Faso	Both	Colon and rectum cancer	2019	7.27	5.82	9.12
Burundi	Both	Colon and rectum cancer	2019	7.17	5.21	10.11
Cabo Verde	Both	Colon and rectum cancer	2019	11.35	8.97	13.38
Cambodia	Both	Colon and rectum cancer	2019	14.02	11.24	16.54
Cameroon	Both	Colon and rectum cancer	2019	10.64	8.25	13.84
Canada	Both	Colon and rectum cancer	2019	16.22	14.74	17.44
Central African Republic	Both	Colon and rectum cancer	2019	6.37	4.71	8.79
Chad	Both	Colon and rectum cancer	2019	7.37	5.77	9.35
Chile	Both	Colon and rectum cancer	2019	14.71	13.46	15.85
China	Both	Colon and rectum cancer	2019	13.86	11.92	16.01
Colombia	Both	Colon and rectum cancer	2019	9.63	7.48	12.24
Comoros	Both	Colon and rectum cancer	2019	8.56	6.39	10.80
Congo	Both	Colon and rectum cancer	2019	11.40	8.76	14.52
Cook Islands	Both	Colon and rectum cancer	2019	7.77	6.54	9.34
Costa Rica	Both	Colon and rectum cancer	2019	15.38	12.15	19.28
Côte d'Ivoire	Both	Colon and rectum cancer	2019	9.41	7.62	11.47
Croatia	Both	Colon and rectum cancer	2019	25.28	20.14	31.22
Cuba	Both	Colon and rectum cancer	2019	17.83	14.73	21.48
Cyprus	Both	Colon and rectum cancer	2019	14.32	12.40	16.32
Czechia	Both	Colon and rectum cancer	2019	21.58	17.80	25.98
Democratic People's Republic of Korea	Both	Colon and rectum cancer	2019	10.89	8.14	13.82
Democratic Republic of the Congo	Both	Colon and rectum cancer	2019	6.23	4.15	9.51
Denmark	Both	Colon and rectum cancer	2019	21.75	19.76	23.49
Djibouti	Both	Colon and rectum cancer	2019	11.19	8.66	14.66
Dominica	Both	Colon and rectum cancer	2019	14.96	12.42	18.09
Dominican Republic	Both	Colon and rectum cancer	2019	12.42	9.23	16.14
Ecuador	Both	Colon and rectum cancer	2019	11.34	9.07	14.16
Egypt	Both	Colon and rectum cancer	2019	7.45	5.40	10.11
El Salvador	Both	Colon and rectum cancer	2019	9.05	6.98	11.68
Equatorial Guinea	Both	Colon and rectum cancer	2019	14.24	9.12	20.02
Eritrea	Both	Colon and rectum cancer	2019	9.97	7.87	12.70
Estonia	Both	Colon and rectum cancer	2019	18.51	14.81	23.22

Eswatini	Both	Colon and rectum cancer	2019	13.52	9.32	18.23
Ethiopia	Both	Colon and rectum cancer	2019	7.33	5.47	10.36
Fiji	Both	Colon and rectum cancer	2019	11.65	9.51	14.20
Finland	Both	Colon and rectum cancer	2019	12.33	11.25	13.35
France	Both	Colon and rectum cancer	2019	16.41	14.69	17.82
Gabon	Both	Colon and rectum cancer	2019	14.86	11.25	18.24
Gambia	Both	Colon and rectum cancer	2019	6.56	4.78	8.76
Georgia	Both	Colon and rectum cancer	2019	13.19	11.08	15.44
Germany	Both	Colon and rectum cancer	2019	18.01	16.54	19.26
Ghana	Both	Colon and rectum cancer	2019	8.76	6.96	10.92
Greece	Both	Colon and rectum cancer	2019	14.87	13.67	15.87
Greenland	Both	Colon and rectum cancer	2019	31.38	26.04	37.13
Grenada	Both	Colon and rectum cancer	2019	19.29	17.44	21.14
Guam	Both	Colon and rectum cancer	2019	14.16	12.02	16.74
Guatemala	Both	Colon and rectum cancer	2019	9.09	7.32	11.27
Guinea	Both	Colon and rectum cancer	2019	7.24	5.51	9.24
Guinea-Bissau	Both	Colon and rectum cancer	2019	9.06	6.93	11.29
Guyana	Both	Colon and rectum cancer	2019	14.68	11.87	18.25
Haiti	Both	Colon and rectum cancer	2019	10.53	7.33	14.09
Honduras	Both	Colon and rectum cancer	2019	7.65	5.34	10.67
Hungary	Both	Colon and rectum cancer	2019	28.56	23.65	34.03
Iceland	Both	Colon and rectum cancer	2019	11.81	10.53	13.16
India	Both	Colon and rectum cancer	2019	7.50	6.35	8.76
Indonesia	Both	Colon and rectum cancer	2019	15.58	10.54	19.86
Iran (Islamic Republic of)	Both	Colon and rectum cancer	2019	9.31	8.53	10.08
Iraq	Both	Colon and rectum cancer	2019	8.32	6.58	10.16
Ireland	Both	Colon and rectum cancer	2019	18.01	16.26	19.51
Israel	Both	Colon and rectum cancer	2019	16.56	14.91	17.85
Italy	Both	Colon and rectum cancer	2019	15.83	14.41	16.70
Jamaica	Both	Colon and rectum cancer	2019	16.52	13.35	20.35
Japan	Both	Colon and rectum cancer	2019	15.85	13.93	16.93
Jordan	Both	Colon and rectum cancer	2019	13.07	10.93	15.46
Kazakhstan	Both	Colon and rectum cancer	2019	13.50	11.79	15.28
Kenya	Both	Colon and rectum cancer	2019	8.14	6.52	10.04
Kiribati	Both	Colon and rectum cancer	2019	10.76	8.26	14.05
Kuwait	Both	Colon and rectum cancer	2019	10.14	8.41	12.04
Kyrgyzstan	Both	Colon and rectum cancer	2019	8.24	7.25	9.34
Lao People's Democratic Republic	Both	Colon and rectum cancer	2019	13.33	9.60	17.23
Latvia	Both	Colon and rectum cancer	2019	17.63	14.77	21.21
Lebanon	Both	Colon and rectum cancer	2019	17.55	14.26	23.15
Lesotho	Both	Colon and rectum cancer	2019	11.67	8.66	15.10
Liberia	Both	Colon and rectum cancer	2019	6.68	4.57	9.51
Libya	Both	Colon and rectum cancer	2019	12.45	9.07	15.84
Lithuania	Both	Colon and rectum cancer	2019	16.90	14.05	20.27
Luxembourg	Both	Colon and rectum cancer	2019	15.97	13.87	18.08
Madagascar	Both	Colon and rectum cancer	2019	7.11	5.27	9.34
Malawi	Both	Colon and rectum cancer	2019	6.06	4.74	7.49
Malaysia	Both	Colon and rectum cancer	2019	20.32	16.15	24.85
Maldives	Both	Colon and rectum cancer	2019	8.71	7.13	10.38
Mali	Both	Colon and rectum cancer	2019	7.84	6.29	9.73
Malta	Both	Colon and rectum cancer	2019	14.08	12.38	15.89
Marshall Islands	Both	Colon and rectum cancer	2019	12.29	9.55	15.44
Mauritania	Both	Colon and rectum cancer	2019	8.35	6.53	10.34
Mauritius	Both	Colon and rectum cancer	2019	12.77	10.53	15.50
Mexico	Both	Colon and rectum cancer	2019	9.24	7.95	10.52
Micronesia (Federated States of)	Both	Colon and rectum cancer	2019	13.08	9.50	16.86
Monaco	Both	Colon and rectum cancer	2019	24.27	19.64	28.78
Mongolia	Both	Colon and rectum cancer	2019	9.31	7.44	11.69
Montenegro	Both	Colon and rectum cancer	2019	17.23	14.08	20.40
Morocco	Both	Colon and rectum cancer	2019	8.48	6.31	10.51
Mozambique	Both	Colon and rectum cancer	2019	8.66	6.46	11.20
Myanmar	Both	Colon and rectum cancer	2019	12.82	9.60	16.23

Namibia	Both	Colon and rectum cancer	2019	8.68	6.97	10.81
Nauru	Both	Colon and rectum cancer	2019	16.55	11.75	21.15
Nepal	Both	Colon and rectum cancer	2019	5.40	3.89	7.42
Netherlands	Both	Colon and rectum cancer	2019	21.75	19.80	23.55
New Zealand	Both	Colon and rectum cancer	2019	20.26	18.34	21.79
Nicaragua	Both	Colon and rectum cancer	2019	10.86	9.23	12.50
Niger	Both	Colon and rectum cancer	2019	5.62	4.24	7.51
Nigeria	Both	Colon and rectum cancer	2019	8.63	6.75	10.78
Niue	Both	Colon and rectum cancer	2019	13.61	10.77	17.01
North Macedonia	Both	Colon and rectum cancer	2019	22.13	17.80	27.27
Northern Mariana Islands	Both	Colon and rectum cancer	2019	18.34	15.88	20.86
Norway	Both	Colon and rectum cancer	2019	19.84	18.04	21.11
Oman	Both	Colon and rectum cancer	2019	10.20	8.48	12.04
Pakistan	Both	Colon and rectum cancer	2019	8.34	6.72	10.41
Palau	Both	Colon and rectum cancer	2019	13.69	10.87	16.66
Palestine	Both	Colon and rectum cancer	2019	19.60	16.69	22.68
Panama	Both	Colon and rectum cancer	2019	10.90	8.54	13.81
Papua New Guinea	Both	Colon and rectum cancer	2019	7.46	5.48	9.64
Paraguay	Both	Colon and rectum cancer	2019	12.16	9.47	15.31
Peru	Both	Colon and rectum cancer	2019	9.44	7.12	12.25
Philippines	Both	Colon and rectum cancer	2019	14.89	12.46	17.81
Poland	Both	Colon and rectum cancer	2019	24.72	20.98	28.96
Portugal	Both	Colon and rectum cancer	2019	20.11	18.52	21.52
Puerto Rico	Both	Colon and rectum cancer	2019	15.44	12.15	19.55
Qatar	Both	Colon and rectum cancer	2019	16.48	12.93	20.54
Republic of Korea	Both	Colon and rectum cancer	2019	13.87	12.22	15.30
Republic of Moldova	Both	Colon and rectum cancer	2019	17.66	15.45	20.01
Romania	Both	Colon and rectum cancer	2019	20.18	16.61	24.11
Russian Federation	Both	Colon and rectum cancer	2019	18.17	15.96	20.54
Rwanda	Both	Colon and rectum cancer	2019	8.79	7.08	10.83
Saint Kitts and Nevis	Both	Colon and rectum cancer	2019	18.32	15.84	21.11
Saint Lucia	Both	Colon and rectum cancer	2019	12.08	10.24	14.11
Saint Vincent and the Grenadines	Both	Colon and rectum cancer	2019	14.42	12.69	16.48
Samoa	Both	Colon and rectum cancer	2019	10.06	8.18	12.34
San Marino	Both	Colon and rectum cancer	2019	21.24	14.31	29.61
Sao Tome and Principe	Both	Colon and rectum cancer	2019	15.18	11.24	20.55
Saudi Arabia	Both	Colon and rectum cancer	2019	9.66	7.79	11.64
Senegal	Both	Colon and rectum cancer	2019	8.70	7.13	10.77
Serbia	Both	Colon and rectum cancer	2019	25.38	20.63	31.01
Seychelles	Both	Colon and rectum cancer	2019	25.33	22.17	28.67
Sierra Leone	Both	Colon and rectum cancer	2019	6.96	5.44	8.86
Singapore	Both	Colon and rectum cancer	2019	14.89	13.22	16.12
Slovakia	Both	Colon and rectum cancer	2019	26.31	20.96	32.80
Slovenia	Both	Colon and rectum cancer	2019	19.84	15.58	25.45
Solomon Islands	Both	Colon and rectum cancer	2019	10.43	7.38	13.29
Somalia	Both	Colon and rectum cancer	2019	5.01	3.17	9.27
South Africa	Both	Colon and rectum cancer	2019	11.21	10.13	12.60
South Sudan	Both	Colon and rectum cancer	2019	9.99	6.57	14.78
Spain	Both	Colon and rectum cancer	2019	18.85	17.05	20.21
Sri Lanka	Both	Colon and rectum cancer	2019	6.41	4.81	8.26
Sudan	Both	Colon and rectum cancer	2019	7.10	5.26	10.59
Suriname	Both	Colon and rectum cancer	2019	16.26	13.57	19.24
Sweden	Both	Colon and rectum cancer	2019	15.96	14.67	16.96
Switzerland	Both	Colon and rectum cancer	2019	11.85	10.70	12.84
Syrian Arab Republic	Both	Colon and rectum cancer	2019	6.28	4.70	8.15
Taiwan (Province of China)	Both	Colon and rectum cancer	2019	26.26	20.88	33.29
Tajikistan	Both	Colon and rectum cancer	2019	10.22	8.41	12.35
Thailand	Both	Colon and rectum cancer	2019	10.54	7.88	13.56
Timor-Leste	Both	Colon and rectum cancer	2019	12.29	8.48	15.79
Togo	Both	Colon and rectum cancer	2019	7.88	5.90	10.04
Tokelau	Both	Colon and rectum cancer	2019	10.85	8.14	13.95
Tonga	Both	Colon and rectum cancer	2019	6.91	5.40	8.68

Trinidad and Tobago	Both	Colon and rectum cancer	2019	13.97	10.74	17.96
Tunisia	Both	Colon and rectum cancer	2019	9.73	7.17	12.98
Turkey	Both	Colon and rectum cancer	2019	13.11	10.62	15.83
Turkmenistan	Both	Colon and rectum cancer	2019	7.08	5.81	8.71
Tuvalu	Both	Colon and rectum cancer	2019	10.90	8.08	13.99
Uganda	Both	Colon and rectum cancer	2019	11.55	9.24	13.75
Ukraine	Both	Colon and rectum cancer	2019	19.18	16.46	22.37
United Arab Emirates	Both	Colon and rectum cancer	2019	17.40	11.94	24.35
United Kingdom	Both	Colon and rectum cancer	2019	18.09	16.77	18.85
United Republic of Tanzania	Both	Colon and rectum cancer	2019	9.51	7.74	11.82
United States of America	Both	Colon and rectum cancer	2019	14.77	13.86	15.32
United States Virgin Islands	Both	Colon and rectum cancer	2019	26.57	22.30	31.01
Uruguay	Both	Colon and rectum cancer	2019	26.12	24.14	27.82
Uzbekistan	Both	Colon and rectum cancer	2019	10.35	9.07	11.79
Vanuatu	Both	Colon and rectum cancer	2019	9.29	7.09	11.92
Venezuela (Bolivarian Republic of)	Both	Colon and rectum cancer	2019	11.31	8.72	14.59
Viet Nam	Both	Colon and rectum cancer	2019	17.50	13.92	21.05
Yemen	Both	Colon and rectum cancer	2019	6.66	5.01	9.07
Zambia	Both	Colon and rectum cancer	2019	12.62	9.51	15.96
Zimbabwe	Both	Colon and rectum cancer	2019	12.93	9.93	16.20

sex_name	cause_name	year	Age-standardised incidence rate (per 100 000 person-years)	95% CI (lower)	95% CI (upper)
Male	Liver cancer	1990	13.07	11.42	14.87
Female	Liver cancer	1990	5.22	4.62	5.96
Both	Liver cancer	1990	8.98	8.10	9.97
Male	Liver cancer	1991	13.63	12.34	15.09
Female	Liver cancer	1991	5.33	4.81	5.97
Both	Liver cancer	1991	9.31	8.56	10.15
Male	Liver cancer	1992	14.11	13.01	15.30
Female	Liver cancer	1992	5.42	4.93	6.01
Both	Liver cancer	1992	9.59	8.95	10.29
Male	Liver cancer	1993	14.49	13.48	15.57
Female	Liver cancer	1993	5.49	5.04	6.05
Both	Liver cancer	1993	9.80	9.23	10.41
Male	Liver cancer	1994	14.78	13.74	15.95
Female	Liver cancer	1994	5.53	5.09	6.08
Both	Liver cancer	1994	9.97	9.40	10.61
Male	Liver cancer	1995	14.96	13.79	16.34
Female	Liver cancer	1995	5.56	5.09	6.11
Both	Liver cancer	1995	10.06	9.44	10.79
Male	Liver cancer	1996	15.02	14.06	16.16
Female	Liver cancer	1996	5.55	5.14	6.04
Both	Liver cancer	1996	10.09	9.56	10.70
Male	Liver cancer	1997	14.98	14.12	15.90
Female	Liver cancer	1997	5.51	5.13	5.92
Both	Liver cancer	1997	10.05	9.54	10.58
Male	Liver cancer	1998	14.85	13.96	15.78
Female	Liver cancer	1998	5.43	5.07	5.82
Both	Liver cancer	1998	9.94	9.46	10.46
Male	Liver cancer	1999	14.64	13.67	15.59
Female	Liver cancer	1999	5.33	4.99	5.71
Both	Liver cancer	1999	9.78	9.29	10.30
Male	Liver cancer	2000	14.35	13.26	15.44
Female	Liver cancer	2000	5.21	4.86	5.60
Both	Liver cancer	2000	9.58	9.03	10.15
Male	Liver cancer	2001	13.71	12.91	14.54
Female	Liver cancer	2001	5.00	4.69	5.30
Both	Liver cancer	2001	9.16	8.70	9.60
Male	Liver cancer	2002	12.63	12.02	13.26
Female	Liver cancer	2002	4.69	4.40	4.95
Both	Liver cancer	2002	8.47	8.10	8.84
Male	Liver cancer	2003	11.41	10.88	11.96
Female	Liver cancer	2003	4.34	4.07	4.59
Both	Liver cancer	2003	7.70	7.37	8.03
Male	Liver cancer	2004	10.39	9.93	10.90
Female	Liver cancer	2004	4.06	3.77	4.29
Both	Liver cancer	2004	7.06	6.74	7.36
Male	Liver cancer	2005	9.87	9.40	10.39
Female	Liver cancer	2005	3.91	3.62	4.14
Both	Liver cancer	2005	6.73	6.41	7.05

Male	Liver cancer	2006	9.74	9.33	10.20
Female	Liver cancer	2006	3.86	3.59	4.06
Both	Liver cancer	2006	6.65	6.34	6.93
Male	Liver cancer	2007	9.66	9.23	10.12
Female	Liver cancer	2007	3.83	3.58	4.01
Both	Liver cancer	2007	6.59	6.29	6.86
Male	Liver cancer	2008	9.60	9.18	10.06
Female	Liver cancer	2008	3.80	3.56	3.98
Both	Liver cancer	2008	6.55	6.25	6.82
Male	Liver cancer	2009	9.57	9.10	10.06
Female	Liver cancer	2009	3.78	3.52	3.96
Both	Liver cancer	2009	6.52	6.21	6.81
Male	Liver cancer	2010	9.54	9.03	10.13
Female	Liver cancer	2010	3.76	3.49	3.96
Both	Liver cancer	2010	6.50	6.15	6.81
Male	Liver cancer	2011	9.53	9.06	10.05
Female	Liver cancer	2011	3.74	3.46	3.92
Both	Liver cancer	2011	6.48	6.16	6.77
Male	Liver cancer	2012	9.54	9.09	10.03
Female	Liver cancer	2012	3.71	3.44	3.88
Both	Liver cancer	2012	6.47	6.15	6.73
Male	Liver cancer	2013	9.56	9.10	10.03
Female	Liver cancer	2013	3.68	3.42	3.87
Both	Liver cancer	2013	6.47	6.14	6.73
Male	Liver cancer	2014	9.59	9.07	10.11
Female	Liver cancer	2014	3.66	3.40	3.87
Both	Liver cancer	2014	6.47	6.11	6.77
Male	Liver cancer	2015	9.61	9.02	10.26
Female	Liver cancer	2015	3.65	3.36	3.89
Both	Liver cancer	2015	6.47	6.08	6.83
Male	Liver cancer	2016	9.64	9.08	10.22
Female	Liver cancer	2016	3.64	3.34	3.89
Both	Liver cancer	2016	6.48	6.11	6.82
Male	Liver cancer	2017	9.67	8.86	10.50
Female	Liver cancer	2017	3.63	3.27	3.97
Both	Liver cancer	2017	6.49	6.03	6.98
Male	Liver cancer	2018	9.69	8.95	10.44
Female	Liver cancer	2018	3.63	3.27	3.95
Both	Liver cancer	2018	6.50	6.04	6.96
Male	Liver cancer	2019	9.71	8.69	10.84
Female	Liver cancer	2019	3.63	3.23	4.05
Both	Liver cancer	2019	6.51	5.95	7.16

location_name	sex_name	cause_name	year	Age-standardised incidence rate (per 100 000 person-years)	95% CI (lower)	95% CI (upper)
High SDI	Male	Liver cancer	1990	8.47	8.25	8.68
High SDI	Female	Liver cancer	1990	2.61	2.48	2.72
High SDI	Both	Liver cancer	1990	5.27	5.11	5.39
High-middle SDI	Male	Liver cancer	1990	15.39	12.98	17.97
High-middle SDI	Female	Liver cancer	1990	5.26	4.54	6.06
High-middle SDI	Both	Liver cancer	1990	9.92	8.73	11.20
Low SDI	Male	Liver cancer	1990	5.14	4.32	5.98
Low SDI	Female	Liver cancer	1990	3.00	2.48	3.65
Low SDI	Both	Liver cancer	1990	4.08	3.58	4.63
Low-middle SDI	Male	Liver cancer	1990	6.71	5.87	7.71
Low-middle SDI	Female	Liver cancer	1990	3.95	3.51	4.56
Low-middle SDI	Both	Liver cancer	1990	5.36	4.85	5.93
Middle SDI	Male	Liver cancer	1990	20.34	16.92	24.28
Middle SDI	Female	Liver cancer	1990	9.13	7.71	10.94
Middle SDI	Both	Liver cancer	1990	14.73	12.80	17.04
High SDI	Male	Liver cancer	1991	8.91	8.70	9.10
High SDI	Female	Liver cancer	1991	2.77	2.62	2.87
High SDI	Both	Liver cancer	1991	5.55	5.39	5.67
High-middle SDI	Male	Liver cancer	1991	16.11	14.10	18.17
High-middle SDI	Female	Liver cancer	1991	5.38	4.76	6.07
High-middle SDI	Both	Liver cancer	1991	10.32	9.32	11.39
Low SDI	Male	Liver cancer	1991	4.41	3.79	5.07
Low SDI	Female	Liver cancer	1991	3.01	2.49	3.62
Low SDI	Both	Liver cancer	1991	3.72	3.27	4.18
Low-middle SDI	Male	Liver cancer	1991	6.83	6.12	7.74
Low-middle SDI	Female	Liver cancer	1991	3.96	3.56	4.52
Low-middle SDI	Both	Liver cancer	1991	5.43	4.99	5.94
Middle SDI	Male	Liver cancer	1991	15.98	14.03	18.01
Middle SDI	Female	Liver cancer	1991	9.21	7.99	10.77
Middle SDI	Both	Liver cancer	1991	12.61	11.34	14.04
High SDI	Male	Liver cancer	1992	9.36	9.13	9.54
High SDI	Female	Liver cancer	1992	2.93	2.76	3.02
High SDI	Both	Liver cancer	1992	5.84	5.66	5.97
High-middle SDI	Male	Liver cancer	1992	16.70	15.09	18.40
High-middle SDI	Female	Liver cancer	1992	5.46	4.89	6.09
High-middle SDI	Both	Liver cancer	1992	10.64	9.80	11.56
Low SDI	Male	Liver cancer	1992	4.47	3.89	5.12
Low SDI	Female	Liver cancer	1992	3.01	2.50	3.63
Low SDI	Both	Liver cancer	1992	3.75	3.32	4.19
Low-middle SDI	Male	Liver cancer	1992	7.02	6.36	7.88
Low-middle SDI	Female	Liver cancer	1992	3.97	3.59	4.52
Low-middle SDI	Both	Liver cancer	1992	5.53	5.12	6.00
Middle SDI	Male	Liver cancer	1992	16.48	14.89	18.19
Middle SDI	Female	Liver cancer	1992	9.24	8.14	10.64
Middle SDI	Both	Liver cancer	1992	12.87	11.80	14.06
High SDI	Male	Liver cancer	1993	9.78	9.54	9.99
High SDI	Female	Liver cancer	1993	3.08	2.90	3.18
High SDI	Both	Liver cancer	1993	6.12	5.91	6.26

High-middle SDI	Male	Liver cancer	1993	17.17	15.66	18.83
High-middle SDI	Female	Liver cancer	1993	5.53	4.99	6.13
High-middle SDI	Both	Liver cancer	1993	10.89	10.12	11.74
Low SDI	Male	Liver cancer	1993	4.52	3.95	5.15
Low SDI	Female	Liver cancer	1993	3.01	2.50	3.63
Low SDI	Both	Liver cancer	1993	3.77	3.34	4.20
Low-middle SDI	Male	Liver cancer	1993	7.17	6.43	8.00
Low-middle SDI	Female	Liver cancer	1993	3.97	3.59	4.51
Low-middle SDI	Both	Liver cancer	1993	5.60	5.17	6.08
Middle SDI	Male	Liver cancer	1993	16.84	15.38	18.41
Middle SDI	Female	Liver cancer	1993	9.22	8.26	10.54
Middle SDI	Both	Liver cancer	1993	13.04	12.07	14.11
High SDI	Male	Liver cancer	1994	10.15	9.88	10.40
High SDI	Female	Liver cancer	1994	3.22	3.03	3.34
High SDI	Both	Liver cancer	1994	6.37	6.15	6.53
High-middle SDI	Male	Liver cancer	1994	17.52	15.93	19.31
High-middle SDI	Female	Liver cancer	1994	5.56	5.03	6.18
High-middle SDI	Both	Liver cancer	1994	11.08	10.26	11.99
Low SDI	Male	Liver cancer	1994	4.55	4.00	5.16
Low SDI	Female	Liver cancer	1994	3.01	2.50	3.60
Low SDI	Both	Liver cancer	1994	3.78	3.37	4.20
Low-middle SDI	Male	Liver cancer	1994	7.27	6.41	8.16
Low-middle SDI	Female	Liver cancer	1994	3.96	3.57	4.47
Low-middle SDI	Both	Liver cancer	1994	5.64	5.17	6.16
Middle SDI	Male	Liver cancer	1994	17.13	15.67	18.91
Middle SDI	Female	Liver cancer	1994	9.17	8.23	10.42
Middle SDI	Both	Liver cancer	1994	13.16	12.22	14.21
High SDI	Male	Liver cancer	1995	10.46	10.15	10.76
High SDI	Female	Liver cancer	1995	3.36	3.15	3.48
High SDI	Both	Liver cancer	1995	6.58	6.34	6.77
High-middle SDI	Male	Liver cancer	1995	17.73	15.88	19.92
High-middle SDI	Female	Liver cancer	1995	5.58	4.97	6.23
High-middle SDI	Both	Liver cancer	1995	11.19	10.25	12.26
Low SDI	Male	Liver cancer	1995	4.58	4.03	5.18
Low SDI	Female	Liver cancer	1995	3.00	2.49	3.60
Low SDI	Both	Liver cancer	1995	3.79	3.39	4.20
Low-middle SDI	Male	Liver cancer	1995	7.31	6.34	8.25
Low-middle SDI	Female	Liver cancer	1995	3.94	3.54	4.47
Low-middle SDI	Both	Liver cancer	1995	5.65	5.11	6.23
Middle SDI	Male	Liver cancer	1995	17.29	15.59	19.31
Middle SDI	Female	Liver cancer	1995	9.09	8.11	10.38
Middle SDI	Both	Liver cancer	1995	13.19	12.18	14.33
High SDI	Male	Liver cancer	1996	10.83	10.52	11.10
High SDI	Female	Liver cancer	1996	3.51	3.29	3.65
High SDI	Both	Liver cancer	1996	6.84	6.59	7.02
High-middle SDI	Male	Liver cancer	1996	17.76	16.18	19.52
High-middle SDI	Female	Liver cancer	1996	5.54	5.03	6.10
High-middle SDI	Both	Liver cancer	1996	11.19	10.41	12.04
Low SDI	Male	Liver cancer	1996	4.59	4.07	5.17
Low SDI	Female	Liver cancer	1996	2.99	2.50	3.56
Low SDI	Both	Liver cancer	1996	3.79	3.39	4.20

Low-middle SDI	Male	Liver cancer	1996	7.30	6.38	8.14
Low-middle SDI	Female	Liver cancer	1996	3.90	3.52	4.40
Low-middle SDI	Both	Liver cancer	1996	5.62	5.10	6.16
Middle SDI	Male	Liver cancer	1996	17.25	15.84	18.93
Middle SDI	Female	Liver cancer	1996	8.92	8.08	9.98
Middle SDI	Both	Liver cancer	1996	13.09	12.25	14.03
High SDI	Male	Liver cancer	1997	11.34	11.03	11.59
High SDI	Female	Liver cancer	1997	3.72	3.47	3.86
High SDI	Both	Liver cancer	1997	7.20	6.95	7.38
High-middle SDI	Male	Liver cancer	1997	17.55	16.16	19.03
High-middle SDI	Female	Liver cancer	1997	5.43	4.99	5.92
High-middle SDI	Both	Liver cancer	1997	11.04	10.35	11.76
Low SDI	Male	Liver cancer	1997	4.59	4.08	5.16
Low SDI	Female	Liver cancer	1997	2.98	2.50	3.53
Low SDI	Both	Liver cancer	1997	3.78	3.39	4.19
Low-middle SDI	Male	Liver cancer	1997	7.22	6.33	7.96
Low-middle SDI	Female	Liver cancer	1997	3.83	3.48	4.30
Low-middle SDI	Both	Liver cancer	1997	5.54	5.02	6.05
Middle SDI	Male	Liver cancer	1997	17.05	15.75	18.55
Middle SDI	Female	Liver cancer	1997	8.65	7.91	9.55
Middle SDI	Both	Liver cancer	1997	12.84	12.08	13.67
High SDI	Male	Liver cancer	1998	11.88	11.56	12.12
High SDI	Female	Liver cancer	1998	3.94	3.67	4.09
High SDI	Both	Liver cancer	1998	7.57	7.29	7.75
High-middle SDI	Male	Liver cancer	1998	17.17	15.83	18.59
High-middle SDI	Female	Liver cancer	1998	5.29	4.86	5.76
High-middle SDI	Both	Liver cancer	1998	10.78	10.11	11.45
Low SDI	Male	Liver cancer	1998	4.58	4.07	5.14
Low SDI	Female	Liver cancer	1998	2.96	2.51	3.50
Low SDI	Both	Liver cancer	1998	3.77	3.39	4.16
Low-middle SDI	Male	Liver cancer	1998	7.09	6.20	7.82
Low-middle SDI	Female	Liver cancer	1998	3.75	3.41	4.19
Low-middle SDI	Both	Liver cancer	1998	5.43	4.91	5.90
Middle SDI	Male	Liver cancer	1998	16.70	15.38	18.09
Middle SDI	Female	Liver cancer	1998	8.31	7.65	9.12
Middle SDI	Both	Liver cancer	1998	12.49	11.77	13.27
High SDI	Male	Liver cancer	1999	12.34	12.01	12.59
High SDI	Female	Liver cancer	1999	4.13	3.84	4.28
High SDI	Both	Liver cancer	1999	7.90	7.60	8.07
High-middle SDI	Male	Liver cancer	1999	16.70	15.22	18.23
High-middle SDI	Female	Liver cancer	1999	5.12	4.72	5.58
High-middle SDI	Both	Liver cancer	1999	10.46	9.77	11.22
Low SDI	Male	Liver cancer	1999	4.56	4.08	5.13
Low SDI	Female	Liver cancer	1999	2.95	2.50	3.48
Low SDI	Both	Liver cancer	1999	3.75	3.37	4.13
Low-middle SDI	Male	Liver cancer	1999	6.93	6.05	7.63
Low-middle SDI	Female	Liver cancer	1999	3.65	3.30	4.08
Low-middle SDI	Both	Liver cancer	1999	5.30	4.76	5.79
Middle SDI	Male	Liver cancer	1999	16.27	14.87	17.72
Middle SDI	Female	Liver cancer	1999	7.94	7.28	8.76
Middle SDI	Both	Liver cancer	1999	12.09	11.30	12.90

High SDI	Male	Liver cancer	2000		12.62	12.26	12.90
High SDI	Female	Liver cancer	2000		4.24	3.94	4.41
High SDI	Both	Liver cancer	2000		8.10	7.78	8.29
High-middle SDI	Male	Liver cancer	2000		16.17	14.50	17.94
High-middle SDI	Female	Liver cancer	2000		4.95	4.52	5.42
High-middle SDI	Both	Liver cancer	2000		10.12	9.31	10.99
Low SDI	Male	Liver cancer	2000		4.54	4.05	5.11
Low SDI	Female	Liver cancer	2000		2.93	2.50	3.45
Low SDI	Both	Liver cancer	2000		3.73	3.36	4.12
Low-middle SDI	Male	Liver cancer	2000		6.74	5.83	7.51
Low-middle SDI	Female	Liver cancer	2000		3.56	3.18	3.99
Low-middle SDI	Both	Liver cancer	2000		5.16	4.62	5.64
Middle SDI	Male	Liver cancer	2000		15.79	14.15	17.42
Middle SDI	Female	Liver cancer	2000		7.57	6.88	8.38
Middle SDI	Both	Liver cancer	2000		11.66	10.83	12.59
High SDI	Male	Liver cancer	2001		12.75	12.38	13.01
High SDI	Female	Liver cancer	2001		4.30	3.97	4.47
High SDI	Both	Liver cancer	2001		8.19	7.87	8.38
High-middle SDI	Male	Liver cancer	2001		15.15	13.92	16.45
High-middle SDI	Female	Liver cancer	2001		4.68	4.34	5.06
High-middle SDI	Both	Liver cancer	2001		9.51	8.91	10.16
Low SDI	Male	Liver cancer	2001		4.52	4.06	5.06
Low SDI	Female	Liver cancer	2001		2.92	2.50	3.40
Low SDI	Both	Liver cancer	2001		3.71	3.35	4.08
Low-middle SDI	Male	Liver cancer	2001		6.46	5.67	7.09
Low-middle SDI	Female	Liver cancer	2001		3.44	3.09	3.82
Low-middle SDI	Both	Liver cancer	2001		4.95	4.46	5.37
Middle SDI	Male	Liver cancer	2001		14.87	13.64	16.13
Middle SDI	Female	Liver cancer	2001		7.07	6.53	7.71
Middle SDI	Both	Liver cancer	2001		10.94	10.30	11.64
High SDI	Male	Liver cancer	2002		12.83	12.45	13.09
High SDI	Female	Liver cancer	2002		4.32	3.99	4.50
High SDI	Both	Liver cancer	2002		8.25	7.91	8.44
High-middle SDI	Male	Liver cancer	2002		13.39	12.52	14.37
High-middle SDI	Female	Liver cancer	2002		4.27	3.99	4.58
High-middle SDI	Both	Liver cancer	2002		8.47	8.02	8.97
Low SDI	Male	Liver cancer	2002		4.51	4.05	5.01
Low SDI	Female	Liver cancer	2002		2.90	2.50	3.35
Low SDI	Both	Liver cancer	2002		3.70	3.35	4.04
Low-middle SDI	Male	Liver cancer	2002		6.05	5.34	6.63
Low-middle SDI	Female	Liver cancer	2002		3.28	2.96	3.62
Low-middle SDI	Both	Liver cancer	2002		4.66	4.20	5.03
Middle SDI	Male	Liver cancer	2002		13.37	12.50	14.33
Middle SDI	Female	Liver cancer	2002		6.36	5.93	6.89
Middle SDI	Both	Liver cancer	2002		9.83	9.31	10.39
High SDI	Male	Liver cancer	2003		12.86	12.46	13.13
High SDI	Female	Liver cancer	2003		4.33	3.99	4.51
High SDI	Both	Liver cancer	2003		8.27	7.92	8.46
High-middle SDI	Male	Liver cancer	2003		11.40	10.71	12.15
High-middle SDI	Female	Liver cancer	2003		3.82	3.57	4.08
High-middle SDI	Both	Liver cancer	2003		7.30	6.93	7.69

Low SDI	Male	Liver cancer	2003	4.49	4.01	5.00
Low SDI	Female	Liver cancer	2003	2.89	2.51	3.31
Low SDI	Both	Liver cancer	2003	3.68	3.34	4.01
Low-middle SDI	Male	Liver cancer	2003	5.60	4.96	6.14
Low-middle SDI	Female	Liver cancer	2003	3.12	2.80	3.44
Low-middle SDI	Both	Liver cancer	2003	4.36	3.94	4.69
Middle SDI	Male	Liver cancer	2003	11.70	10.98	12.49
Middle SDI	Female	Liver cancer	2003	5.62	5.23	6.10
Middle SDI	Both	Liver cancer	2003	8.62	8.17	9.11
High SDI	Male	Liver cancer	2004	12.86	12.45	13.16
High SDI	Female	Liver cancer	2004	4.33	3.98	4.52
High SDI	Both	Liver cancer	2004	8.27	7.92	8.49
High-middle SDI	Male	Liver cancer	2004	9.71	9.10	10.39
High-middle SDI	Female	Liver cancer	2004	3.44	3.19	3.69
High-middle SDI	Both	Liver cancer	2004	6.31	5.96	6.68
Low SDI	Male	Liver cancer	2004	4.46	3.99	4.99
Low SDI	Female	Liver cancer	2004	2.87	2.51	3.27
Low SDI	Both	Liver cancer	2004	3.66	3.33	3.99
Low-middle SDI	Male	Liver cancer	2004	5.24	4.64	5.75
Low-middle SDI	Female	Liver cancer	2004	2.99	2.67	3.31
Low-middle SDI	Both	Liver cancer	2004	4.10	3.70	4.43
Middle SDI	Male	Liver cancer	2004	10.30	9.65	11.05
Middle SDI	Female	Liver cancer	2004	5.01	4.65	5.46
Middle SDI	Both	Liver cancer	2004	7.62	7.20	8.11
High SDI	Male	Liver cancer	2005	12.85	12.42	13.17
High SDI	Female	Liver cancer	2005	4.33	3.98	4.53
High SDI	Both	Liver cancer	2005	8.28	7.90	8.51
High-middle SDI	Male	Liver cancer	2005	8.85	8.23	9.59
High-middle SDI	Female	Liver cancer	2005	3.22	2.96	3.48
High-middle SDI	Both	Liver cancer	2005	5.80	5.45	6.20
Low SDI	Male	Liver cancer	2005	4.44	3.95	5.00
Low SDI	Female	Liver cancer	2005	2.85	2.50	3.24
Low SDI	Both	Liver cancer	2005	3.63	3.29	3.97
Low-middle SDI	Male	Liver cancer	2005	5.04	4.47	5.57
Low-middle SDI	Female	Liver cancer	2005	2.92	2.59	3.24
Low-middle SDI	Both	Liver cancer	2005	3.97	3.57	4.29
Middle SDI	Male	Liver cancer	2005	9.59	8.92	10.37
Middle SDI	Female	Liver cancer	2005	4.70	4.30	5.14
Middle SDI	Both	Liver cancer	2005	7.11	6.68	7.63
High SDI	Male	Liver cancer	2006	12.81	12.35	13.12
High SDI	Female	Liver cancer	2006	4.34	3.97	4.53
High SDI	Both	Liver cancer	2006	8.26	7.88	8.50
High-middle SDI	Male	Liver cancer	2006	8.64	8.10	9.27
High-middle SDI	Female	Liver cancer	2006	3.15	2.92	3.36
High-middle SDI	Both	Liver cancer	2006	5.66	5.35	6.01
Low SDI	Male	Liver cancer	2006	4.41	3.93	4.92
Low SDI	Female	Liver cancer	2006	2.83	2.48	3.19
Low SDI	Both	Liver cancer	2006	3.61	3.27	3.94
Low-middle SDI	Male	Liver cancer	2006	4.98	4.45	5.45
Low-middle SDI	Female	Liver cancer	2006	2.89	2.57	3.20
Low-middle SDI	Both	Liver cancer	2006	3.92	3.54	4.23

Middle SDI	Male	Liver cancer	2006	9.44	8.87	10.13
Middle SDI	Female	Liver cancer	2006	4.61	4.28	4.98
Middle SDI	Both	Liver cancer	2006	6.99	6.61	7.44
High SDI	Male	Liver cancer	2007	12.71	12.23	13.05
High SDI	Female	Liver cancer	2007	4.34	3.95	4.54
High SDI	Both	Liver cancer	2007	8.22	7.82	8.47
High-middle SDI	Male	Liver cancer	2007	8.51	8.02	9.08
High-middle SDI	Female	Liver cancer	2007	3.10	2.90	3.28
High-middle SDI	Both	Liver cancer	2007	5.58	5.29	5.89
Low SDI	Male	Liver cancer	2007	4.38	3.91	4.87
Low SDI	Female	Liver cancer	2007	2.81	2.49	3.15
Low SDI	Both	Liver cancer	2007	3.58	3.23	3.91
Low-middle SDI	Male	Liver cancer	2007	4.93	4.45	5.36
Low-middle SDI	Female	Liver cancer	2007	2.87	2.55	3.18
Low-middle SDI	Both	Liver cancer	2007	3.89	3.52	4.18
Middle SDI	Male	Liver cancer	2007	9.41	8.87	10.07
Middle SDI	Female	Liver cancer	2007	4.55	4.27	4.88
Middle SDI	Both	Liver cancer	2007	6.94	6.58	7.37
High SDI	Male	Liver cancer	2008	12.57	12.07	12.95
High SDI	Female	Liver cancer	2008	4.32	3.92	4.54
High SDI	Both	Liver cancer	2008	8.15	7.72	8.41
High-middle SDI	Male	Liver cancer	2008	8.43	7.94	9.01
High-middle SDI	Female	Liver cancer	2008	3.06	2.87	3.24
High-middle SDI	Both	Liver cancer	2008	5.52	5.24	5.84
Low SDI	Male	Liver cancer	2008	4.74	4.26	5.22
Low SDI	Female	Liver cancer	2008	2.79	2.47	3.11
Low SDI	Both	Liver cancer	2008	3.74	3.38	4.08
Low-middle SDI	Male	Liver cancer	2008	4.90	4.45	5.30
Low-middle SDI	Female	Liver cancer	2008	2.85	2.53	3.14
Low-middle SDI	Both	Liver cancer	2008	3.85	3.51	4.16
Middle SDI	Male	Liver cancer	2008	9.40	8.86	10.08
Middle SDI	Female	Liver cancer	2008	4.52	4.24	4.82
Middle SDI	Both	Liver cancer	2008	6.92	6.55	7.35
High SDI	Male	Liver cancer	2009	12.42	11.89	12.84
High SDI	Female	Liver cancer	2009	4.31	3.88	4.53
High SDI	Both	Liver cancer	2009	8.08	7.62	8.35
High-middle SDI	Male	Liver cancer	2009	8.38	7.85	9.00
High-middle SDI	Female	Liver cancer	2009	3.03	2.83	3.23
High-middle SDI	Both	Liver cancer	2009	5.49	5.19	5.83
Low SDI	Male	Liver cancer	2009	4.71	4.24	5.20
Low SDI	Female	Liver cancer	2009	2.77	2.45	3.09
Low SDI	Both	Liver cancer	2009	3.72	3.36	4.07
Low-middle SDI	Male	Liver cancer	2009	4.88	4.46	5.26
Low-middle SDI	Female	Liver cancer	2009	2.83	2.52	3.12
Low-middle SDI	Both	Liver cancer	2009	3.83	3.50	4.13
Middle SDI	Male	Liver cancer	2009	9.42	8.79	10.19
Middle SDI	Female	Liver cancer	2009	4.51	4.20	4.82
Middle SDI	Both	Liver cancer	2009	6.92	6.52	7.38
High SDI	Male	Liver cancer	2010	12.29	11.72	12.74
High SDI	Female	Liver cancer	2010	4.28	3.84	4.52
High SDI	Both	Liver cancer	2010	8.01	7.54	8.29

High-middle SDI	Male	Liver cancer	2010	8.34	7.70	9.07
High-middle SDI	Female	Liver cancer	2010	3.00	2.77	3.21
High-middle SDI	Both	Liver cancer	2010	5.46	5.10	5.83
Low SDI	Male	Liver cancer	2010	4.68	4.20	5.19
Low SDI	Female	Liver cancer	2010	2.75	2.42	3.08
Low SDI	Both	Liver cancer	2010	3.70	3.34	4.05
Low-middle SDI	Male	Liver cancer	2010	4.87	4.47	5.27
Low-middle SDI	Female	Liver cancer	2010	2.82	2.50	3.11
Low-middle SDI	Both	Liver cancer	2010	3.82	3.49	4.13
Middle SDI	Male	Liver cancer	2010	9.45	8.71	10.33
Middle SDI	Female	Liver cancer	2010	4.49	4.14	4.82
Middle SDI	Both	Liver cancer	2010	6.92	6.47	7.43
High SDI	Male	Liver cancer	2011	12.15	11.60	12.54
High SDI	Female	Liver cancer	2011	4.25	3.81	4.47
High SDI	Both	Liver cancer	2011	7.93	7.44	8.20
High-middle SDI	Male	Liver cancer	2011	8.28	7.76	8.91
High-middle SDI	Female	Liver cancer	2011	2.95	2.74	3.14
High-middle SDI	Both	Liver cancer	2011	5.41	5.10	5.72
Low SDI	Male	Liver cancer	2011	4.66	4.19	5.16
Low SDI	Female	Liver cancer	2011	2.73	2.41	3.04
Low SDI	Both	Liver cancer	2011	3.67	3.32	4.02
Low-middle SDI	Male	Liver cancer	2011	4.89	4.51	5.25
Low-middle SDI	Female	Liver cancer	2011	2.81	2.51	3.10
Low-middle SDI	Both	Liver cancer	2011	3.83	3.51	4.11
Middle SDI	Male	Liver cancer	2011	9.52	8.87	10.34
Middle SDI	Female	Liver cancer	2011	4.48	4.16	4.76
Middle SDI	Both	Liver cancer	2011	6.94	6.53	7.42
High SDI	Male	Liver cancer	2012	12.00	11.44	12.34
High SDI	Female	Liver cancer	2012	4.20	3.77	4.43
High SDI	Both	Liver cancer	2012	7.83	7.36	8.09
High-middle SDI	Male	Liver cancer	2012	8.21	7.74	8.78
High-middle SDI	Female	Liver cancer	2012	2.89	2.70	3.06
High-middle SDI	Both	Liver cancer	2012	5.35	5.08	5.63
Low SDI	Male	Liver cancer	2012	4.63	4.16	5.13
Low SDI	Female	Liver cancer	2012	2.72	2.40	3.02
Low SDI	Both	Liver cancer	2012	3.65	3.29	4.00
Low-middle SDI	Male	Liver cancer	2012	4.93	4.56	5.26
Low-middle SDI	Female	Liver cancer	2012	2.82	2.51	3.09
Low-middle SDI	Both	Liver cancer	2012	3.84	3.54	4.12
Middle SDI	Male	Liver cancer	2012	9.65	9.03	10.42
Middle SDI	Female	Liver cancer	2012	4.47	4.16	4.74
Middle SDI	Both	Liver cancer	2012	7.00	6.61	7.42
High SDI	Male	Liver cancer	2013	11.84	11.29	12.17
High SDI	Female	Liver cancer	2013	4.15	3.72	4.38
High SDI	Both	Liver cancer	2013	7.73	7.26	7.99
High-middle SDI	Male	Liver cancer	2013	8.15	7.61	8.73
High-middle SDI	Female	Liver cancer	2013	2.84	2.65	3.02
High-middle SDI	Both	Liver cancer	2013	5.29	4.98	5.58
Low SDI	Male	Liver cancer	2013	4.60	4.12	5.11
Low SDI	Female	Liver cancer	2013	2.71	2.39	3.02
Low SDI	Both	Liver cancer	2013	3.63	3.26	3.98

Low-middle SDI	Male	Liver cancer	2013	4.98	4.61	5.30
Low-middle SDI	Female	Liver cancer	2013	2.82	2.53	3.10
Low-middle SDI	Both	Liver cancer	2013	3.87	3.57	4.14
Middle SDI	Male	Liver cancer	2013	9.81	9.15	10.59
Middle SDI	Female	Liver cancer	2013	4.47	4.16	4.76
Middle SDI	Both	Liver cancer	2013	7.07	6.67	7.48
High SDI	Male	Liver cancer	2014	11.70	11.12	12.06
High SDI	Female	Liver cancer	2014	4.10	3.68	4.34
High SDI	Both	Liver cancer	2014	7.65	7.18	7.91
High-middle SDI	Male	Liver cancer	2014	8.10	7.46	8.77
High-middle SDI	Female	Liver cancer	2014	2.79	2.60	2.98
High-middle SDI	Both	Liver cancer	2014	5.25	4.92	5.59
Low SDI	Male	Liver cancer	2014	4.57	4.08	5.10
Low SDI	Female	Liver cancer	2014	2.70	2.37	3.01
Low SDI	Both	Liver cancer	2014	3.61	3.24	3.98
Low-middle SDI	Male	Liver cancer	2014	5.04	4.66	5.36
Low-middle SDI	Female	Liver cancer	2014	2.83	2.54	3.13
Low-middle SDI	Both	Liver cancer	2014	3.90	3.61	4.17
Middle SDI	Male	Liver cancer	2014	9.97	9.17	10.84
Middle SDI	Female	Liver cancer	2014	4.47	4.13	4.81
Middle SDI	Both	Liver cancer	2014	7.14	6.70	7.62
High SDI	Male	Liver cancer	2015	11.60	11.02	12.00
High SDI	Female	Liver cancer	2015	4.07	3.64	4.32
High SDI	Both	Liver cancer	2015	7.59	7.11	7.87
High-middle SDI	Male	Liver cancer	2015	8.10	7.30	8.95
High-middle SDI	Female	Liver cancer	2015	2.77	2.54	3.01
High-middle SDI	Both	Liver cancer	2015	5.24	4.83	5.64
Low SDI	Male	Liver cancer	2015	4.54	4.01	5.08
Low SDI	Female	Liver cancer	2015	2.68	2.36	3.01
Low SDI	Both	Liver cancer	2015	3.59	3.21	3.98
Low-middle SDI	Male	Liver cancer	2015	5.09	4.67	5.46
Low-middle SDI	Female	Liver cancer	2015	2.84	2.55	3.15
Low-middle SDI	Both	Liver cancer	2015	3.93	3.61	4.20
Middle SDI	Male	Liver cancer	2015	10.09	9.13	11.15
Middle SDI	Female	Liver cancer	2015	4.47	4.05	4.87
Middle SDI	Both	Liver cancer	2015	7.19	6.68	7.75
High SDI	Male	Liver cancer	2016	11.53	10.90	11.98
High SDI	Female	Liver cancer	2016	4.04	3.60	4.31
High SDI	Both	Liver cancer	2016	7.54	7.07	7.87
High-middle SDI	Male	Liver cancer	2016	8.13	7.50	8.84
High-middle SDI	Female	Liver cancer	2016	2.76	2.55	2.98
High-middle SDI	Both	Liver cancer	2016	5.25	4.93	5.61
Low SDI	Male	Liver cancer	2016	4.50	4.00	5.04
Low SDI	Female	Liver cancer	2016	2.67	2.36	3.00
Low SDI	Both	Liver cancer	2016	3.56	3.19	3.96
Low-middle SDI	Male	Liver cancer	2016	5.15	4.78	5.52
Low-middle SDI	Female	Liver cancer	2016	2.84	2.53	3.16
Low-middle SDI	Both	Liver cancer	2016	3.96	3.67	4.25
Middle SDI	Male	Liver cancer	2016	10.23	9.40	11.20
Middle SDI	Female	Liver cancer	2016	4.46	4.10	4.86
Middle SDI	Both	Liver cancer	2016	7.26	6.76	7.78

High SDI	Male	Liver cancer	2017	11.49	10.74	12.08
High SDI	Female	Liver cancer	2017	4.02	3.58	4.33
High SDI	Both	Liver cancer	2017	7.52	6.99	7.94
High-middle SDI	Male	Liver cancer	2017	8.16	7.21	9.20
High-middle SDI	Female	Liver cancer	2017	2.75	2.47	3.07
High-middle SDI	Both	Liver cancer	2017	5.27	4.77	5.78
Low SDI	Male	Liver cancer	2017	4.47	3.92	5.01
Low SDI	Female	Liver cancer	2017	2.65	2.35	3.01
Low SDI	Both	Liver cancer	2017	3.54	3.17	3.94
Low-middle SDI	Male	Liver cancer	2017	5.20	4.76	5.70
Low-middle SDI	Female	Liver cancer	2017	2.85	2.50	3.23
Low-middle SDI	Both	Liver cancer	2017	3.98	3.65	4.32
Middle SDI	Male	Liver cancer	2017	10.36	9.12	11.76
Middle SDI	Female	Liver cancer	2017	4.46	3.93	5.02
Middle SDI	Both	Liver cancer	2017	7.32	6.59	8.09
High SDI	Male	Liver cancer	2018	11.52	10.68	12.32
High SDI	Female	Liver cancer	2018	4.03	3.56	4.38
High SDI	Both	Liver cancer	2018	7.55	6.97	8.05
High-middle SDI	Male	Liver cancer	2018	8.21	7.34	9.12
High-middle SDI	Female	Liver cancer	2018	2.75	2.48	3.03
High-middle SDI	Both	Liver cancer	2018	5.30	4.82	5.75
Low SDI	Male	Liver cancer	2018	4.44	3.89	4.99
Low SDI	Female	Liver cancer	2018	2.65	2.34	3.00
Low SDI	Both	Liver cancer	2018	3.52	3.15	3.93
Low-middle SDI	Male	Liver cancer	2018	5.21	4.82	5.67
Low-middle SDI	Female	Liver cancer	2018	2.85	2.50	3.23
Low-middle SDI	Both	Liver cancer	2018	3.99	3.68	4.33
Middle SDI	Male	Liver cancer	2018	10.40	9.21	11.61
Middle SDI	Female	Liver cancer	2018	4.45	3.93	5.01
Middle SDI	Both	Liver cancer	2018	7.33	6.67	8.03
High SDI	Male	Liver cancer	2019	11.61	10.44	12.95
High SDI	Female	Liver cancer	2019	4.06	3.56	4.53
High SDI	Both	Liver cancer	2019	7.61	6.88	8.36
High-middle SDI	Male	Liver cancer	2019	8.28	7.01	9.66
High-middle SDI	Female	Liver cancer	2019	2.75	2.41	3.14
High-middle SDI	Both	Liver cancer	2019	5.34	4.70	6.04
Low SDI	Male	Liver cancer	2019	4.41	3.86	4.97
Low SDI	Female	Liver cancer	2019	2.65	2.32	2.99
Low SDI	Both	Liver cancer	2019	3.51	3.11	3.91
Low-middle SDI	Male	Liver cancer	2019	5.21	4.64	5.82
Low-middle SDI	Female	Liver cancer	2019	2.85	2.44	3.31
Low-middle SDI	Both	Liver cancer	2019	3.99	3.60	4.44
Middle SDI	Male	Liver cancer	2019	10.41	8.84	12.14
Middle SDI	Female	Liver cancer	2019	4.43	3.75	5.15
Middle SDI	Both	Liver cancer	2019	7.32	6.41	8.28

sex_name	cause_name	year	Age-standardised death rate (per 100 000 person-years)	95% CI (lower)	95% CI (upper)
Male	Liver cancer	1990	12.90	11.30	14.67
Female	Liver cancer	1990	5.33	4.67	6.09
Both	Liver cancer	1990	8.93	8.09	9.90
Male	Liver cancer	1991	13.30	11.81	14.97
Female	Liver cancer	1991	5.39	4.76	6.17
Both	Liver cancer	1991	9.15	8.33	10.12
Male	Liver cancer	1992	13.64	12.25	15.20
Female	Liver cancer	1992	5.45	4.85	6.14
Both	Liver cancer	1992	9.35	8.60	10.19
Male	Liver cancer	1993	14.01	12.64	15.58
Female	Liver cancer	1993	5.51	4.96	6.15
Both	Liver cancer	1993	9.56	8.82	10.35
Male	Liver cancer	1994	14.37	13.11	15.75
Female	Liver cancer	1994	5.57	5.05	6.15
Both	Liver cancer	1994	9.76	9.07	10.47
Male	Liver cancer	1995	14.63	13.46	15.98
Female	Liver cancer	1995	5.59	5.09	6.17
Both	Liver cancer	1995	9.89	9.25	10.55
Male	Liver cancer	1996	14.76	13.65	15.99
Female	Liver cancer	1996	5.59	5.15	6.11
Both	Liver cancer	1996	9.95	9.32	10.60
Male	Liver cancer	1997	14.71	13.63	15.88
Female	Liver cancer	1997	5.53	5.14	6.05
Both	Liver cancer	1997	9.89	9.35	10.48
Male	Liver cancer	1998	14.65	13.65	15.78
Female	Liver cancer	1998	5.46	5.06	5.88
Both	Liver cancer	1998	9.83	9.27	10.43
Male	Liver cancer	1999	14.45	13.41	15.56
Female	Liver cancer	1999	5.39	4.99	5.81
Both	Liver cancer	1999	9.69	9.13	10.25
Male	Liver cancer	2000	13.86	12.74	14.89
Female	Liver cancer	2000	5.17	4.77	5.58
Both	Liver cancer	2000	9.29	8.72	9.85
Male	Liver cancer	2001	12.83	11.73	13.86
Female	Liver cancer	2001	4.82	4.47	5.20
Both	Liver cancer	2001	8.61	8.05	9.14
Male	Liver cancer	2002	11.48	10.70	12.29
Female	Liver cancer	2002	4.47	4.15	4.80
Both	Liver cancer	2002	7.78	7.35	8.20
Male	Liver cancer	2003	10.41	9.82	11.05
Female	Liver cancer	2003	4.15	3.86	4.44
Both	Liver cancer	2003	7.10	6.76	7.45
Male	Liver cancer	2004	9.63	9.14	10.23
Female	Liver cancer	2004	3.92	3.66	4.16
Both	Liver cancer	2004	6.61	6.28	6.91
Male	Liver cancer	2005	9.29	8.80	9.80
Female	Liver cancer	2005	3.81	3.54	4.04
Both	Liver cancer	2005	6.38	6.08	6.69
Male	Liver cancer	2006	9.09	8.64	9.58

Female	Liver cancer	2006	3.77	3.49	4.00
Both	Liver cancer	2006	6.27	5.96	6.54
Male	Liver cancer	2007	8.99	8.54	9.42
Female	Liver cancer	2007	3.72	3.43	3.91
Both	Liver cancer	2007	6.20	5.88	6.45
Male	Liver cancer	2008	9.00	8.55	9.45
Female	Liver cancer	2008	3.72	3.44	3.91
Both	Liver cancer	2008	6.20	5.87	6.46
Male	Liver cancer	2009	8.89	8.43	9.35
Female	Liver cancer	2009	3.67	3.39	3.87
Both	Liver cancer	2009	6.12	5.79	6.39
Male	Liver cancer	2010	8.78	8.31	9.27
Female	Liver cancer	2010	3.63	3.36	3.83
Both	Liver cancer	2010	6.05	5.72	6.33
Male	Liver cancer	2011	8.65	8.16	9.19
Female	Liver cancer	2011	3.58	3.30	3.78
Both	Liver cancer	2011	5.96	5.62	6.27
Male	Liver cancer	2012	8.60	8.13	9.12
Female	Liver cancer	2012	3.52	3.26	3.73
Both	Liver cancer	2012	5.91	5.60	6.20
Male	Liver cancer	2013	8.60	8.10	9.15
Female	Liver cancer	2013	3.50	3.21	3.71
Both	Liver cancer	2013	5.90	5.54	6.21
Male	Liver cancer	2014	8.66	8.10	9.22
Female	Liver cancer	2014	3.51	3.22	3.74
Both	Liver cancer	2014	5.93	5.59	6.24
Male	Liver cancer	2015	8.74	8.19	9.32
Female	Liver cancer	2015	3.51	3.20	3.74
Both	Liver cancer	2015	5.97	5.62	6.29
Male	Liver cancer	2016	8.77	8.15	9.46
Female	Liver cancer	2016	3.50	3.18	3.76
Both	Liver cancer	2016	5.98	5.57	6.37
Male	Liver cancer	2017	8.76	8.03	9.49
Female	Liver cancer	2017	3.48	3.15	3.78
Both	Liver cancer	2017	5.96	5.52	6.37
Male	Liver cancer	2018	8.73	7.93	9.50
Female	Liver cancer	2018	3.47	3.10	3.81
Both	Liver cancer	2018	5.94	5.47	6.42
Male	Liver cancer	2019	8.73	7.88	9.60
Female	Liver cancer	2019	3.46	3.08	3.83
Both	Liver cancer	2019	5.95	5.44	6.44

location_name	sex_name	cause_name	year	Age-standardised death rate (per 100 000 person-years)	95% CI (lower)	95% CI (upper)
High SDI	Male	Liver cancer	1990	7.53	7.32	7.73
High SDI	Female	Liver cancer	1990	2.39	2.25	2.49
High SDI	Both	Liver cancer	1990	4.69	4.54	4.81
High-middle SDI	Male	Liver cancer	1990	15.37	13.00	17.97
High-middle SDI	Female	Liver cancer	1990	5.45	4.70	6.26
High-middle SDI	Both	Liver cancer	1990	9.96	8.75	11.27
Low SDI	Male	Liver cancer	1990	5.51	4.64	6.37
Low SDI	Female	Liver cancer	1990	3.20	2.65	3.91
Low SDI	Both	Liver cancer	1990	4.37	3.80	4.97
Low-middle SDI	Male	Liver cancer	1990	6.90	6.06	7.86
Low-middle SDI	Female	Liver cancer	1990	4.19	3.70	4.83
Low-middle SDI	Both	Liver cancer	1990	5.57	5.05	6.13
Middle SDI	Male	Liver cancer	1990	20.52	17.17	24.35
Middle SDI	Female	Liver cancer	1990	9.55	8.11	11.43
Middle SDI	Both	Liver cancer	1990	15.00	13.15	17.26
High SDI	Male	Liver cancer	1991	7.73	7.54	7.91
High SDI	Female	Liver cancer	1991	2.45	2.32	2.54
High SDI	Both	Liver cancer	1991	4.82	4.66	4.93
High-middle SDI	Male	Liver cancer	1991	16.02	13.73	18.42
High-middle SDI	Female	Liver cancer	1991	5.53	4.79	6.39
High-middle SDI	Both	Liver cancer	1991	10.31	9.18	11.55
Low SDI	Male	Liver cancer	1991	5.57	4.74	6.44
Low SDI	Female	Liver cancer	1991	3.20	2.63	3.89
Low SDI	Both	Liver cancer	1991	4.39	3.84	4.97
Low-middle SDI	Male	Liver cancer	1991	7.09	6.23	8.12
Low-middle SDI	Female	Liver cancer	1991	4.18	3.69	4.87
Low-middle SDI	Both	Liver cancer	1991	5.66	5.15	6.25
Middle SDI	Male	Liver cancer	1991	20.99	18.02	24.52
Middle SDI	Female	Liver cancer	1991	9.59	8.19	11.46
Middle SDI	Both	Liver cancer	1991	15.25	13.53	17.32
High SDI	Male	Liver cancer	1992	7.98	7.77	8.15
High SDI	Female	Liver cancer	1992	2.54	2.40	2.62
High SDI	Both	Liver cancer	1992	4.98	4.81	5.09
High-middle SDI	Male	Liver cancer	1992	16.53	14.44	18.94
High-middle SDI	Female	Liver cancer	1992	5.59	4.92	6.35
High-middle SDI	Both	Liver cancer	1992	10.58	9.47	11.78
Low SDI	Male	Liver cancer	1992	5.64	4.82	6.45
Low SDI	Female	Liver cancer	1992	3.20	2.65	3.90
Low SDI	Both	Liver cancer	1992	4.43	3.90	4.95
Low-middle SDI	Male	Liver cancer	1992	7.26	6.32	8.30
Low-middle SDI	Female	Liver cancer	1992	4.20	3.73	4.85
Low-middle SDI	Both	Liver cancer	1992	5.76	5.20	6.37
Middle SDI	Male	Liver cancer	1992	21.35	18.50	24.48
Middle SDI	Female	Liver cancer	1992	9.61	8.25	11.28
Middle SDI	Both	Liver cancer	1992	15.44	13.82	17.33
High SDI	Male	Liver cancer	1993	8.27	8.03	8.46
High SDI	Female	Liver cancer	1993	2.63	2.47	2.74
High SDI	Both	Liver cancer	1993	5.16	4.98	5.30
High-middle SDI	Male	Liver cancer	1993	16.96	14.95	19.22

High-middle SDI	Female	Liver cancer	1993	5.66	5.03	6.35
High-middle SDI	Both	Liver cancer	1993	10.82	9.82	11.86
Low SDI	Male	Liver cancer	1993	5.70	4.93	6.49
Low SDI	Female	Liver cancer	1993	3.20	2.67	3.89
Low SDI	Both	Liver cancer	1993	4.46	3.94	4.99
Low-middle SDI	Male	Liver cancer	1993	7.44	6.44	8.50
Low-middle SDI	Female	Liver cancer	1993	4.22	3.75	4.85
Low-middle SDI	Both	Liver cancer	1993	5.86	5.27	6.46
Middle SDI	Male	Liver cancer	1993	21.84	19.05	24.93
Middle SDI	Female	Liver cancer	1993	9.61	8.35	11.14
Middle SDI	Both	Liver cancer	1993	15.67	14.07	17.41
High SDI	Male	Liver cancer	1994	8.58	8.33	8.79
High SDI	Female	Liver cancer	1994	2.75	2.57	2.85
High SDI	Both	Liver cancer	1994	5.37	5.17	5.50
High-middle SDI	Male	Liver cancer	1994	17.35	15.54	19.40
High-middle SDI	Female	Liver cancer	1994	5.72	5.07	6.38
High-middle SDI	Both	Liver cancer	1994	11.03	10.11	12.05
Low SDI	Male	Liver cancer	1994	5.75	5.00	6.50
Low SDI	Female	Liver cancer	1994	3.20	2.67	3.89
Low SDI	Both	Liver cancer	1994	4.48	3.99	4.96
Low-middle SDI	Male	Liver cancer	1994	7.59	6.53	8.53
Low-middle SDI	Female	Liver cancer	1994	4.21	3.77	4.78
Low-middle SDI	Both	Liver cancer	1994	5.92	5.34	6.52
Middle SDI	Male	Liver cancer	1994	22.30	19.72	25.20
Middle SDI	Female	Liver cancer	1994	9.59	8.46	11.08
Middle SDI	Both	Liver cancer	1994	15.89	14.47	17.50
High SDI	Male	Liver cancer	1995	9.01	8.73	9.24
High SDI	Female	Liver cancer	1995	2.92	2.72	3.03
High SDI	Both	Liver cancer	1995	5.66	5.44	5.81
High-middle SDI	Male	Liver cancer	1995	17.55	15.74	19.55
High-middle SDI	Female	Liver cancer	1995	5.71	5.11	6.38
High-middle SDI	Both	Liver cancer	1995	11.11	10.22	12.06
Low SDI	Male	Liver cancer	1995	5.79	5.09	6.54
Low SDI	Female	Liver cancer	1995	3.20	2.65	3.85
Low SDI	Both	Liver cancer	1995	4.50	4.02	5.00
Low-middle SDI	Male	Liver cancer	1995	7.65	6.66	8.67
Low-middle SDI	Female	Liver cancer	1995	4.16	3.72	4.75
Low-middle SDI	Both	Liver cancer	1995	5.93	5.36	6.53
Middle SDI	Male	Liver cancer	1995	22.54	20.30	25.19
Middle SDI	Female	Liver cancer	1995	9.49	8.41	10.82
Middle SDI	Both	Liver cancer	1995	15.96	14.66	17.36
High SDI	Male	Liver cancer	1996	9.34	9.04	9.55
High SDI	Female	Liver cancer	1996	3.07	2.86	3.18
High SDI	Both	Liver cancer	1996	5.90	5.66	6.04
High-middle SDI	Male	Liver cancer	1996	17.61	15.86	19.62
High-middle SDI	Female	Liver cancer	1996	5.66	5.11	6.31
High-middle SDI	Both	Liver cancer	1996	11.11	10.19	12.07
Low SDI	Male	Liver cancer	1996	5.83	5.13	6.57
Low SDI	Female	Liver cancer	1996	3.19	2.68	3.81
Low SDI	Both	Liver cancer	1996	4.51	4.03	4.99
Low-middle SDI	Male	Liver cancer	1996	7.68	6.67	8.62

Low-middle SDI	Female	Liver cancer	1996	4.12	3.68	4.66
Low-middle SDI	Both	Liver cancer	1996	5.91	5.35	6.49
Middle SDI	Male	Liver cancer	1996	22.61	20.43	25.00
Middle SDI	Female	Liver cancer	1996	9.35	8.38	10.56
Middle SDI	Both	Liver cancer	1996	15.90	14.70	17.33
High SDI	Male	Liver cancer	1997	9.67	9.38	9.88
High SDI	Female	Liver cancer	1997	3.22	3.00	3.34
High SDI	Both	Liver cancer	1997	6.13	5.88	6.28
High-middle SDI	Male	Liver cancer	1997	17.44	15.69	19.32
High-middle SDI	Female	Liver cancer	1997	5.55	5.04	6.10
High-middle SDI	Both	Liver cancer	1997	10.98	10.13	11.93
Low SDI	Male	Liver cancer	1997	5.85	5.17	6.57
Low SDI	Female	Liver cancer	1997	3.18	2.67	3.81
Low SDI	Both	Liver cancer	1997	4.51	4.06	4.97
Low-middle SDI	Male	Liver cancer	1997	7.68	6.81	8.56
Low-middle SDI	Female	Liver cancer	1997	4.07	3.65	4.56
Low-middle SDI	Both	Liver cancer	1997	5.89	5.34	6.42
Middle SDI	Male	Liver cancer	1997	22.23	20.18	24.51
Middle SDI	Female	Liver cancer	1997	9.07	8.27	10.14
Middle SDI	Both	Liver cancer	1997	15.56	14.50	16.77
High SDI	Male	Liver cancer	1998	10.18	9.86	10.40
High SDI	Female	Liver cancer	1998	3.39	3.15	3.52
High SDI	Both	Liver cancer	1998	6.47	6.21	6.63
High-middle SDI	Male	Liver cancer	1998	17.18	15.51	18.98
High-middle SDI	Female	Liver cancer	1998	5.42	4.94	5.92
High-middle SDI	Both	Liver cancer	1998	10.78	9.96	11.68
Low SDI	Male	Liver cancer	1998	5.82	5.20	6.55
Low SDI	Female	Liver cancer	1998	3.17	2.69	3.75
Low SDI	Both	Liver cancer	1998	4.49	4.04	4.94
Low-middle SDI	Male	Liver cancer	1998	7.55	6.65	8.43
Low-middle SDI	Female	Liver cancer	1998	3.98	3.56	4.48
Low-middle SDI	Both	Liver cancer	1998	5.77	5.23	6.30
Middle SDI	Male	Liver cancer	1998	21.86	19.88	24.23
Middle SDI	Female	Liver cancer	1998	8.78	7.93	9.75
Middle SDI	Both	Liver cancer	1998	15.22	14.11	16.47
High SDI	Male	Liver cancer	1999	10.53	10.19	10.76
High SDI	Female	Liver cancer	1999	3.54	3.27	3.69
High SDI	Both	Liver cancer	1999	6.72	6.43	6.88
High-middle SDI	Male	Liver cancer	1999	16.73	15.06	18.50
High-middle SDI	Female	Liver cancer	1999	5.29	4.81	5.78
High-middle SDI	Both	Liver cancer	1999	10.51	9.70	11.33
Low SDI	Male	Liver cancer	1999	5.78	5.13	6.53
Low SDI	Female	Liver cancer	1999	3.15	2.67	3.73
Low SDI	Both	Liver cancer	1999	4.45	4.01	4.90
Low-middle SDI	Male	Liver cancer	1999	7.35	6.41	8.21
Low-middle SDI	Female	Liver cancer	1999	3.89	3.46	4.38
Low-middle SDI	Both	Liver cancer	1999	5.62	5.05	6.15
Middle SDI	Male	Liver cancer	1999	21.34	19.37	23.46
Middle SDI	Female	Liver cancer	1999	8.49	7.72	9.39
Middle SDI	Both	Liver cancer	1999	14.81	13.74	15.97
High SDI	Male	Liver cancer	2000	10.66	10.34	10.88

High SDI	Female	Liver cancer	2000	3.63	3.36	3.77
High SDI	Both	Liver cancer	2000	6.83	6.54	6.99
High-middle SDI	Male	Liver cancer	2000	15.79	14.22	17.50
High-middle SDI	Female	Liver cancer	2000	5.00	4.56	5.49
High-middle SDI	Both	Liver cancer	2000	9.92	9.16	10.76
Low SDI	Male	Liver cancer	2000	5.75	5.11	6.47
Low SDI	Female	Liver cancer	2000	3.13	2.67	3.67
Low SDI	Both	Liver cancer	2000	4.43	3.97	4.87
Low-middle SDI	Male	Liver cancer	2000	7.11	6.20	7.87
Low-middle SDI	Female	Liver cancer	2000	3.76	3.36	4.24
Low-middle SDI	Both	Liver cancer	2000	5.44	4.89	5.91
Middle SDI	Male	Liver cancer	2000	20.09	18.05	22.05
Middle SDI	Female	Liver cancer	2000	7.92	7.17	8.86
Middle SDI	Both	Liver cancer	2000	13.91	12.82	15.01
High SDI	Male	Liver cancer	2001	10.60	10.27	10.84
High SDI	Female	Liver cancer	2001	3.64	3.36	3.78
High SDI	Both	Liver cancer	2001	6.82	6.50	6.98
High-middle SDI	Male	Liver cancer	2001	14.15	12.66	15.74
High-middle SDI	Female	Liver cancer	2001	4.55	4.15	5.02
High-middle SDI	Both	Liver cancer	2001	8.92	8.20	9.68
Low SDI	Male	Liver cancer	2001	5.71	5.10	6.40
Low SDI	Female	Liver cancer	2001	3.11	2.66	3.63
Low SDI	Both	Liver cancer	2001	4.40	3.97	4.83
Low-middle SDI	Male	Liver cancer	2001	6.77	5.89	7.48
Low-middle SDI	Female	Liver cancer	2001	3.60	3.21	4.05
Low-middle SDI	Both	Liver cancer	2001	5.18	4.67	5.64
Middle SDI	Male	Liver cancer	2001	18.15	16.00	20.07
Middle SDI	Female	Liver cancer	2001	7.11	6.45	7.93
Middle SDI	Both	Liver cancer	2001	12.53	11.50	13.57
High SDI	Male	Liver cancer	2002	10.46	10.11	10.71
High SDI	Female	Liver cancer	2002	3.62	3.32	3.77
High SDI	Both	Liver cancer	2002	6.74	6.42	6.92
High-middle SDI	Male	Liver cancer	2002	12.02	10.95	13.10
High-middle SDI	Female	Liver cancer	2002	4.09	3.73	4.49
High-middle SDI	Both	Liver cancer	2002	7.68	7.14	8.26
Low SDI	Male	Liver cancer	2002	5.68	5.10	6.35
Low SDI	Female	Liver cancer	2002	3.09	2.66	3.57
Low SDI	Both	Liver cancer	2002	4.37	3.95	4.80
Low-middle SDI	Male	Liver cancer	2002	6.35	5.64	7.02
Low-middle SDI	Female	Liver cancer	2002	3.46	3.09	3.84
Low-middle SDI	Both	Liver cancer	2002	4.90	4.44	5.30
Middle SDI	Male	Liver cancer	2002	15.67	14.18	17.20
Middle SDI	Female	Liver cancer	2002	6.33	5.73	7.07
Middle SDI	Both	Liver cancer	2002	10.90	10.11	11.75
High SDI	Male	Liver cancer	2003	10.48	10.12	10.70
High SDI	Female	Liver cancer	2003	3.63	3.31	3.78
High SDI	Both	Liver cancer	2003	6.76	6.43	6.93
High-middle SDI	Male	Liver cancer	2003	10.24	9.44	11.11
High-middle SDI	Female	Liver cancer	2003	3.66	3.35	3.98
High-middle SDI	Both	Liver cancer	2003	6.64	6.22	7.11
Low SDI	Male	Liver cancer	2003	5.70	5.11	6.37

Low SDI	Female	Liver cancer	2003		3.07	2.66	3.54
Low SDI	Both	Liver cancer	2003		4.36	3.95	4.77
Low-middle SDI	Male	Liver cancer	2003		5.91	5.27	6.50
Low-middle SDI	Female	Liver cancer	2003		3.28	2.91	3.64
Low-middle SDI	Both	Liver cancer	2003		4.58	4.14	4.96
Middle SDI	Male	Liver cancer	2003		13.69	12.54	15.00
Middle SDI	Female	Liver cancer	2003		5.66	5.15	6.26
Middle SDI	Both	Liver cancer	2003		9.59	8.96	10.30
High SDI	Male	Liver cancer	2004		10.38	10.01	10.61
High SDI	Female	Liver cancer	2004		3.60	3.28	3.76
High SDI	Both	Liver cancer	2004		6.70	6.35	6.88
High-middle SDI	Male	Liver cancer	2004		9.03	8.34	9.80
High-middle SDI	Female	Liver cancer	2004		3.35	3.09	3.63
High-middle SDI	Both	Liver cancer	2004		5.91	5.54	6.31
Low SDI	Male	Liver cancer	2004		5.66	5.09	6.32
Low SDI	Female	Liver cancer	2004		3.06	2.66	3.48
Low SDI	Both	Liver cancer	2004		4.34	3.94	4.74
Low-middle SDI	Male	Liver cancer	2004		5.55	4.91	6.14
Low-middle SDI	Female	Liver cancer	2004		3.14	2.77	3.51
Low-middle SDI	Both	Liver cancer	2004		4.33	3.88	4.69
Middle SDI	Male	Liver cancer	2004		12.32	11.38	13.47
Middle SDI	Female	Liver cancer	2004		5.17	4.72	5.69
Middle SDI	Both	Liver cancer	2004		8.66	8.15	9.31
High SDI	Male	Liver cancer	2005		10.30	9.92	10.56
High SDI	Female	Liver cancer	2005		3.57	3.24	3.73
High SDI	Both	Liver cancer	2005		6.65	6.30	6.84
High-middle SDI	Male	Liver cancer	2005		8.54	7.94	9.27
High-middle SDI	Female	Liver cancer	2005		3.22	2.99	3.47
High-middle SDI	Both	Liver cancer	2005		5.62	5.29	6.00
Low SDI	Male	Liver cancer	2005		5.64	5.04	6.28
Low SDI	Female	Liver cancer	2005		3.04	2.67	3.47
Low SDI	Both	Liver cancer	2005		4.32	3.91	4.74
Low-middle SDI	Male	Liver cancer	2005		5.42	4.83	5.95
Low-middle SDI	Female	Liver cancer	2005		3.11	2.75	3.44
Low-middle SDI	Both	Liver cancer	2005		4.25	3.83	4.62
Middle SDI	Male	Liver cancer	2005		11.69	10.81	12.63
Middle SDI	Female	Liver cancer	2005		4.92	4.52	5.35
Middle SDI	Both	Liver cancer	2005		8.22	7.71	8.77
High SDI	Male	Liver cancer	2006		10.12	9.72	10.37
High SDI	Female	Liver cancer	2006		3.58	3.23	3.75
High SDI	Both	Liver cancer	2006		6.58	6.22	6.77
High-middle SDI	Male	Liver cancer	2006		8.28	7.75	8.90
High-middle SDI	Female	Liver cancer	2006		3.17	2.93	3.41
High-middle SDI	Both	Liver cancer	2006		5.48	5.15	5.79
Low SDI	Male	Liver cancer	2006		5.57	4.99	6.23
Low SDI	Female	Liver cancer	2006		3.02	2.66	3.42
Low SDI	Both	Liver cancer	2006		4.27	3.86	4.68
Low-middle SDI	Male	Liver cancer	2006		5.37	4.84	5.87
Low-middle SDI	Female	Liver cancer	2006		3.09	2.74	3.43
Low-middle SDI	Both	Liver cancer	2006		4.21	3.81	4.55
Middle SDI	Male	Liver cancer	2006		11.39	10.57	12.29

Middle SDI	Female	Liver cancer	2006	4.81	4.45	5.19
Middle SDI	Both	Liver cancer	2006	8.01	7.53	8.49
High SDI	Male	Liver cancer	2007	9.98	9.55	10.28
High SDI	Female	Liver cancer	2007	3.57	3.21	3.75
High SDI	Both	Liver cancer	2007	6.51	6.13	6.72
High-middle SDI	Male	Liver cancer	2007	8.16	7.60	8.72
High-middle SDI	Female	Liver cancer	2007	3.11	2.86	3.32
High-middle SDI	Both	Liver cancer	2007	5.39	5.08	5.68
Low SDI	Male	Liver cancer	2007	5.55	4.98	6.16
Low SDI	Female	Liver cancer	2007	2.98	2.63	3.38
Low SDI	Both	Liver cancer	2007	4.24	3.84	4.64
Low-middle SDI	Male	Liver cancer	2007	5.31	4.81	5.78
Low-middle SDI	Female	Liver cancer	2007	3.07	2.71	3.40
Low-middle SDI	Both	Liver cancer	2007	4.17	3.78	4.51
Middle SDI	Male	Liver cancer	2007	11.27	10.50	12.05
Middle SDI	Female	Liver cancer	2007	4.70	4.35	5.05
Middle SDI	Both	Liver cancer	2007	7.90	7.44	8.34
High SDI	Male	Liver cancer	2008	9.82	9.38	10.15
High SDI	Female	Liver cancer	2008	3.54	3.17	3.73
High SDI	Both	Liver cancer	2008	6.42	6.03	6.66
High-middle SDI	Male	Liver cancer	2008	8.24	7.69	8.81
High-middle SDI	Female	Liver cancer	2008	3.12	2.89	3.33
High-middle SDI	Both	Liver cancer	2008	5.44	5.12	5.75
Low SDI	Male	Liver cancer	2008	5.52	4.93	6.15
Low SDI	Female	Liver cancer	2008	2.97	2.60	3.35
Low SDI	Both	Liver cancer	2008	4.22	3.81	4.62
Low-middle SDI	Male	Liver cancer	2008	5.32	4.82	5.76
Low-middle SDI	Female	Liver cancer	2008	3.06	2.71	3.38
Low-middle SDI	Both	Liver cancer	2008	4.17	3.79	4.49
Middle SDI	Male	Liver cancer	2008	11.34	10.53	12.19
Middle SDI	Female	Liver cancer	2008	4.71	4.37	5.04
Middle SDI	Both	Liver cancer	2008	7.93	7.48	8.41
High SDI	Male	Liver cancer	2009	9.63	9.19	9.96
High SDI	Female	Liver cancer	2009	3.50	3.12	3.69
High SDI	Both	Liver cancer	2009	6.32	5.91	6.55
High-middle SDI	Male	Liver cancer	2009	8.03	7.47	8.61
High-middle SDI	Female	Liver cancer	2009	3.03	2.81	3.26
High-middle SDI	Both	Liver cancer	2009	5.30	4.99	5.62
Low SDI	Male	Liver cancer	2009	5.50	4.93	6.10
Low SDI	Female	Liver cancer	2009	2.95	2.58	3.32
Low SDI	Both	Liver cancer	2009	4.19	3.78	4.59
Low-middle SDI	Male	Liver cancer	2009	5.25	4.78	5.68
Low-middle SDI	Female	Liver cancer	2009	3.01	2.66	3.33
Low-middle SDI	Both	Liver cancer	2009	4.11	3.76	4.41
Middle SDI	Male	Liver cancer	2009	11.31	10.49	12.28
Middle SDI	Female	Liver cancer	2009	4.67	4.32	5.03
Middle SDI	Both	Liver cancer	2009	7.89	7.44	8.44
High SDI	Male	Liver cancer	2010	9.49	9.03	9.86
High SDI	Female	Liver cancer	2010	3.47	3.08	3.66
High SDI	Both	Liver cancer	2010	6.24	5.83	6.48
High-middle SDI	Male	Liver cancer	2010	7.80	7.24	8.41

High-middle SDI	Female	Liver cancer	2010	2.94	2.73	3.16
High-middle SDI	Both	Liver cancer	2010	5.15	4.83	5.46
Low SDI	Male	Liver cancer	2010	5.48	4.90	6.08
Low SDI	Female	Liver cancer	2010	2.93	2.58	3.30
Low SDI	Both	Liver cancer	2010	4.17	3.77	4.57
Low-middle SDI	Male	Liver cancer	2010	5.23	4.79	5.62
Low-middle SDI	Female	Liver cancer	2010	3.01	2.66	3.32
Low-middle SDI	Both	Liver cancer	2010	4.09	3.73	4.39
Middle SDI	Male	Liver cancer	2010	11.22	10.37	12.30
Middle SDI	Female	Liver cancer	2010	4.64	4.29	4.98
Middle SDI	Both	Liver cancer	2010	7.83	7.32	8.41
High SDI	Male	Liver cancer	2011	9.37	8.89	9.69
High SDI	Female	Liver cancer	2011	3.43	3.05	3.63
High SDI	Both	Liver cancer	2011	6.16	5.74	6.40
High-middle SDI	Male	Liver cancer	2011	7.50	6.95	8.15
High-middle SDI	Female	Liver cancer	2011	2.84	2.59	3.05
High-middle SDI	Both	Liver cancer	2011	4.96	4.63	5.29
Low SDI	Male	Liver cancer	2011	5.45	4.87	6.08
Low SDI	Female	Liver cancer	2011	2.91	2.55	3.26
Low SDI	Both	Liver cancer	2011	4.14	3.75	4.56
Low-middle SDI	Male	Liver cancer	2011	5.21	4.77	5.60
Low-middle SDI	Female	Liver cancer	2011	3.00	2.63	3.34
Low-middle SDI	Both	Liver cancer	2011	4.08	3.70	4.38
Middle SDI	Male	Liver cancer	2011	11.16	10.24	12.28
Middle SDI	Female	Liver cancer	2011	4.58	4.20	4.91
Middle SDI	Both	Liver cancer	2011	7.76	7.24	8.38
High SDI	Male	Liver cancer	2012	9.22	8.74	9.49
High SDI	Female	Liver cancer	2012	3.40	3.02	3.60
High SDI	Both	Liver cancer	2012	6.08	5.66	6.31
High-middle SDI	Male	Liver cancer	2012	7.34	6.82	7.93
High-middle SDI	Female	Liver cancer	2012	2.75	2.52	2.95
High-middle SDI	Both	Liver cancer	2012	4.83	4.55	5.15
Low SDI	Male	Liver cancer	2012	5.40	4.79	5.99
Low SDI	Female	Liver cancer	2012	2.89	2.54	3.22
Low SDI	Both	Liver cancer	2012	4.10	3.70	4.52
Low-middle SDI	Male	Liver cancer	2012	5.21	4.80	5.59
Low-middle SDI	Female	Liver cancer	2012	2.96	2.63	3.27
Low-middle SDI	Both	Liver cancer	2012	4.05	3.72	4.33
Middle SDI	Male	Liver cancer	2012	11.20	10.26	12.32
Middle SDI	Female	Liver cancer	2012	4.51	4.16	4.83
Middle SDI	Both	Liver cancer	2012	7.74	7.20	8.33
High SDI	Male	Liver cancer	2013	9.08	8.61	9.34
High SDI	Female	Liver cancer	2013	3.36	2.98	3.55
High SDI	Both	Liver cancer	2013	6.00	5.58	6.21
High-middle SDI	Male	Liver cancer	2013	7.26	6.71	7.89
High-middle SDI	Female	Liver cancer	2013	2.70	2.48	2.89
High-middle SDI	Both	Liver cancer	2013	4.78	4.46	5.08
Low SDI	Male	Liver cancer	2013	5.39	4.81	6.01
Low SDI	Female	Liver cancer	2013	2.89	2.52	3.23
Low SDI	Both	Liver cancer	2013	4.10	3.70	4.52
Low-middle SDI	Male	Liver cancer	2013	5.28	4.91	5.64

Low-middle SDI	Female	Liver cancer	2013	2.97	2.62	3.28
Low-middle SDI	Both	Liver cancer	2013	4.08	3.77	4.36
Middle SDI	Male	Liver cancer	2013	11.33	10.35	12.48
Middle SDI	Female	Liver cancer	2013	4.52	4.15	4.85
Middle SDI	Both	Liver cancer	2013	7.81	7.26	8.40
High SDI	Male	Liver cancer	2014	8.95	8.51	9.23
High SDI	Female	Liver cancer	2014	3.32	2.94	3.52
High SDI	Both	Liver cancer	2014	5.93	5.51	6.14
High-middle SDI	Male	Liver cancer	2014	7.33	6.71	7.95
High-middle SDI	Female	Liver cancer	2014	2.70	2.49	2.90
High-middle SDI	Both	Liver cancer	2014	4.81	4.48	5.14
Low SDI	Male	Liver cancer	2014	5.30	4.70	5.93
Low SDI	Female	Liver cancer	2014	2.87	2.51	3.21
Low SDI	Both	Liver cancer	2014	4.05	3.64	4.46
Low-middle SDI	Male	Liver cancer	2014	5.34	4.98	5.67
Low-middle SDI	Female	Liver cancer	2014	2.98	2.65	3.30
Low-middle SDI	Both	Liver cancer	2014	4.12	3.82	4.39
Middle SDI	Male	Liver cancer	2014	11.56	10.42	12.78
Middle SDI	Female	Liver cancer	2014	4.54	4.15	4.94
Middle SDI	Both	Liver cancer	2014	7.93	7.31	8.59
High SDI	Male	Liver cancer	2015	8.89	8.43	9.16
High SDI	Female	Liver cancer	2015	3.31	2.93	3.51
High SDI	Both	Liver cancer	2015	5.89	5.48	6.11
High-middle SDI	Male	Liver cancer	2015	7.40	6.73	8.13
High-middle SDI	Female	Liver cancer	2015	2.69	2.45	2.92
High-middle SDI	Both	Liver cancer	2015	4.84	4.47	5.20
Low SDI	Male	Liver cancer	2015	5.28	4.69	5.92
Low SDI	Female	Liver cancer	2015	2.86	2.54	3.21
Low SDI	Both	Liver cancer	2015	4.03	3.63	4.46
Low-middle SDI	Male	Liver cancer	2015	5.45	5.03	5.84
Low-middle SDI	Female	Liver cancer	2015	3.03	2.67	3.37
Low-middle SDI	Both	Liver cancer	2015	4.19	3.86	4.49
Middle SDI	Male	Liver cancer	2015	11.75	10.66	13.02
Middle SDI	Female	Liver cancer	2015	4.54	4.12	4.96
Middle SDI	Both	Liver cancer	2015	8.01	7.40	8.68
High SDI	Male	Liver cancer	2016	8.83	8.35	9.15
High SDI	Female	Liver cancer	2016	3.29	2.92	3.50
High SDI	Both	Liver cancer	2016	5.86	5.46	6.10
High-middle SDI	Male	Liver cancer	2016	7.41	6.63	8.21
High-middle SDI	Female	Liver cancer	2016	2.67	2.41	2.91
High-middle SDI	Both	Liver cancer	2016	4.84	4.42	5.27
Low SDI	Male	Liver cancer	2016	5.23	4.59	5.86
Low SDI	Female	Liver cancer	2016	2.84	2.49	3.20
Low SDI	Both	Liver cancer	2016	4.00	3.58	4.44
Low-middle SDI	Male	Liver cancer	2016	5.51	5.06	5.96
Low-middle SDI	Female	Liver cancer	2016	3.03	2.67	3.40
Low-middle SDI	Both	Liver cancer	2016	4.22	3.88	4.54
Middle SDI	Male	Liver cancer	2016	11.85	10.58	13.25
Middle SDI	Female	Liver cancer	2016	4.53	4.07	5.00
Middle SDI	Both	Liver cancer	2016	8.05	7.37	8.84
High SDI	Male	Liver cancer	2017	8.79	8.27	9.15

High SDI	Female	Liver cancer	2017	3.26	2.88	3.47
High SDI	Both	Liver cancer	2017	5.83	5.41	6.10
High-middle SDI	Male	Liver cancer	2017	7.39	6.54	8.27
High-middle SDI	Female	Liver cancer	2017	2.65	2.37	2.95
High-middle SDI	Both	Liver cancer	2017	4.82	4.40	5.26
Low SDI	Male	Liver cancer	2017	5.19	4.50	5.80
Low SDI	Female	Liver cancer	2017	2.83	2.48	3.19
Low SDI	Both	Liver cancer	2017	3.97	3.56	4.43
Low-middle SDI	Male	Liver cancer	2017	5.55	5.08	6.08
Low-middle SDI	Female	Liver cancer	2017	3.03	2.66	3.44
Low-middle SDI	Both	Liver cancer	2017	4.24	3.89	4.61
Middle SDI	Male	Liver cancer	2017	11.83	10.44	13.40
Middle SDI	Female	Liver cancer	2017	4.50	3.97	5.02
Middle SDI	Both	Liver cancer	2017	8.02	7.23	8.87
High SDI	Male	Liver cancer	2018	8.82	8.24	9.22
High SDI	Female	Liver cancer	2018	3.27	2.88	3.50
High SDI	Both	Liver cancer	2018	5.86	5.42	6.15
High-middle SDI	Male	Liver cancer	2018	7.37	6.53	8.36
High-middle SDI	Female	Liver cancer	2018	2.64	2.35	2.94
High-middle SDI	Both	Liver cancer	2018	4.81	4.37	5.31
Low SDI	Male	Liver cancer	2018	5.16	4.51	5.79
Low SDI	Female	Liver cancer	2018	2.83	2.49	3.23
Low SDI	Both	Liver cancer	2018	3.96	3.52	4.42
Low-middle SDI	Male	Liver cancer	2018	5.56	5.03	6.15
Low-middle SDI	Female	Liver cancer	2018	3.04	2.63	3.52
Low-middle SDI	Both	Liver cancer	2018	4.25	3.85	4.64
Middle SDI	Male	Liver cancer	2018	11.73	10.09	13.34
Middle SDI	Female	Liver cancer	2018	4.45	3.86	5.10
Middle SDI	Both	Liver cancer	2018	7.95	7.03	8.90
High SDI	Male	Liver cancer	2019	8.86	8.27	9.31
High SDI	Female	Liver cancer	2019	3.29	2.90	3.52
High SDI	Both	Liver cancer	2019	5.89	5.44	6.21
High-middle SDI	Male	Liver cancer	2019	7.41	6.42	8.49
High-middle SDI	Female	Liver cancer	2019	2.63	2.31	2.98
High-middle SDI	Both	Liver cancer	2019	4.83	4.34	5.38
Low SDI	Male	Liver cancer	2019	5.12	4.44	5.78
Low SDI	Female	Liver cancer	2019	2.82	2.46	3.19
Low SDI	Both	Liver cancer	2019	3.93	3.49	4.38
Low-middle SDI	Male	Liver cancer	2019	5.54	4.99	6.14
Low-middle SDI	Female	Liver cancer	2019	3.02	2.57	3.53
Low-middle SDI	Both	Liver cancer	2019	4.23	3.86	4.68
Middle SDI	Male	Liver cancer	2019	11.71	9.76	13.67
Middle SDI	Female	Liver cancer	2019	4.42	3.76	5.13
Middle SDI	Both	Liver cancer	2019	7.92	6.97	8.93

location_name	sex_name	cause_name	year	Age-standardised incidence rate (per 100 000 person-years)	95% CI (lower)	95% CI (upper)
Afghanistan	Both	Liver cancer	2019	9.82	7.63	12.44
Albania	Both	Liver cancer	2019	6.58	4.82	8.76
Algeria	Both	Liver cancer	2019	2.21	1.73	2.78
American Samoa	Both	Liver cancer	2019	6.88	5.69	8.35
Andorra	Both	Liver cancer	2019	10.95	8.23	14.37
Angola	Both	Liver cancer	2019	2.39	1.92	2.99
Antigua and Barbuda	Both	Liver cancer	2019	2.69	2.29	3.13
Argentina	Both	Liver cancer	2019	2.05	1.61	2.56
Armenia	Both	Liver cancer	2019	6.60	5.47	7.85
Australia	Both	Liver cancer	2019	4.52	3.48	5.84
Austria	Both	Liver cancer	2019	5.50	4.41	6.89
Azerbaijan	Both	Liver cancer	2019	4.07	3.11	5.46
Bahamas	Both	Liver cancer	2019	3.10	2.56	3.81
Bahrain	Both	Liver cancer	2019	5.53	4.39	6.91
Bangladesh	Both	Liver cancer	2019	2.58	2.03	3.21
Barbados	Both	Liver cancer	2019	2.63	2.17	3.14
Belarus	Both	Liver cancer	2019	2.49	1.81	3.34
Belgium	Both	Liver cancer	2019	4.27	3.36	5.42
Belize	Both	Liver cancer	2019	3.04	2.60	3.52
Benin	Both	Liver cancer	2019	4.78	3.57	6.37
Bermuda	Both	Liver cancer	2019	2.09	1.73	2.56
Bhutan	Both	Liver cancer	2019	3.04	2.11	4.29
Bolivia (Plurinational State of)	Both	Liver cancer	2019	4.58	3.41	5.93
Bosnia and Herzegovina	Both	Liver cancer	2019	7.53	5.99	9.46
Botswana	Both	Liver cancer	2019	1.47	1.07	1.95
Brazil	Both	Liver cancer	2019	2.37	2.23	2.49
Brunei Darussalam	Both	Liver cancer	2019	11.75	9.96	13.78
Bulgaria	Both	Liver cancer	2019	4.50	3.58	5.58
Burkina Faso	Both	Liver cancer	2019	2.18	1.66	2.75
Burundi	Both	Liver cancer	2019	2.89	1.98	4.53
Cabo Verde	Both	Liver cancer	2019	11.56	9.52	13.94
Cambodia	Both	Liver cancer	2019	9.20	7.26	11.26
Cameroon	Both	Liver cancer	2019	0.65	0.47	0.87
Canada	Both	Liver cancer	2019	4.75	3.62	6.11
Central African Republic	Both	Liver cancer	2019	3.18	2.08	4.66
Chad	Both	Liver cancer	2019	5.31	4.05	6.84
Chile	Both	Liver cancer	2019	2.98	2.31	3.75
China	Both	Liver cancer	2019	10.46	8.74	12.42
Colombia	Both	Liver cancer	2019	2.51	1.91	3.22
Comoros	Both	Liver cancer	2019	2.79	1.94	4.33
Congo	Both	Liver cancer	2019	2.89	2.11	3.94
Cook Islands	Both	Liver cancer	2019	11.37	9.15	13.93
Costa Rica	Both	Liver cancer	2019	5.14	3.97	6.51
Côte d'Ivoire	Both	Liver cancer	2019	4.73	3.50	6.43
Croatia	Both	Liver cancer	2019	3.73	2.92	4.75
Cuba	Both	Liver cancer	2019	2.34	1.89	2.90
Cyprus	Both	Liver cancer	2019	3.69	3.16	4.32
Czechia	Both	Liver cancer	2019	2.96	2.42	3.65
Democratic People's Republic of Korea	Both	Liver cancer	2019	10.23	7.66	13.36
Democratic Republic of the Congo	Both	Liver cancer	2019	2.14	1.65	2.79
Denmark	Both	Liver cancer	2019	3.98	3.11	5.10
Djibouti	Both	Liver cancer	2019	3.22	2.07	5.23
Dominica	Both	Liver cancer	2019	3.18	2.58	3.95
Dominican Republic	Both	Liver cancer	2019	4.64	3.16	6.99
Ecuador	Both	Liver cancer	2019	3.40	2.72	4.37
Egypt	Both	Liver cancer	2019	20.92	15.09	28.51

El Salvador	Both	Liver cancer	2019	1.99	1.50	2.59
Equatorial Guinea	Both	Liver cancer	2019	3.17	1.87	4.63
Eritrea	Both	Liver cancer	2019	3.10	2.17	4.41
Estonia	Both	Liver cancer	2019	3.71	2.84	4.71
Eswatini	Both	Liver cancer	2019	18.43	5.61	32.97
Ethiopia	Both	Liver cancer	2019	2.73	2.21	3.47
Fiji	Both	Liver cancer	2019	6.04	4.67	7.62
Finland	Both	Liver cancer	2019	5.54	4.35	7.14
France	Both	Liver cancer	2019	6.66	5.05	8.72
Gabon	Both	Liver cancer	2019	3.27	2.15	4.67
Gambia	Both	Liver cancer	2019	38.21	27.53	49.67
Georgia	Both	Liver cancer	2019	3.52	2.87	4.32
Germany	Both	Liver cancer	2019	5.07	3.96	6.44
Ghana	Both	Liver cancer	2019	5.68	4.31	7.20
Greece	Both	Liver cancer	2019	3.52	2.78	4.42
Greenland	Both	Liver cancer	2019	6.20	4.93	7.74
Grenada	Both	Liver cancer	2019	2.96	2.61	3.36
Guam	Both	Liver cancer	2019	5.89	4.80	7.19
Guatemala	Both	Liver cancer	2019	4.47	3.55	5.60
Guinea	Both	Liver cancer	2019	32.17	22.33	41.90
Guinea-Bissau	Both	Liver cancer	2019	6.12	4.45	8.28
Guyana	Both	Liver cancer	2019	3.03	2.36	3.84
Haiti	Both	Liver cancer	2019	4.14	2.49	6.26
Honduras	Both	Liver cancer	2019	14.80	6.80	21.70
Hungary	Both	Liver cancer	2019	2.58	2.11	3.16
Iceland	Both	Liver cancer	2019	3.52	3.06	4.05
India	Both	Liver cancer	2019	2.61	2.20	3.10
Indonesia	Both	Liver cancer	2019	2.23	1.90	2.51
Iran (Islamic Republic of)	Both	Liver cancer	2019	3.85	3.50	4.25
Iraq	Both	Liver cancer	2019	6.09	4.71	7.57
Ireland	Both	Liver cancer	2019	4.00	3.07	5.10
Israel	Both	Liver cancer	2019	2.97	2.31	3.79
Italy	Both	Liver cancer	2019	5.97	4.81	7.37
Jamaica	Both	Liver cancer	2019	2.61	2.07	3.25
Japan	Both	Liver cancer	2019	12.71	10.51	14.98
Jordan	Both	Liver cancer	2019	2.46	1.98	3.04
Kazakhstan	Both	Liver cancer	2019	6.30	5.43	7.24
Kenya	Both	Liver cancer	2019	2.90	1.92	4.25
Kiribati	Both	Liver cancer	2019	11.69	9.09	14.66
Kuwait	Both	Liver cancer	2019	2.75	2.17	3.42
Kyrgyzstan	Both	Liver cancer	2019	2.54	2.16	2.91
Lao People's Democratic Republic	Both	Liver cancer	2019	6.72	5.06	8.54
Latvia	Both	Liver cancer	2019	2.68	2.22	3.26
Lebanon	Both	Liver cancer	2019	3.67	2.74	5.09
Lesotho	Both	Liver cancer	2019	14.44	6.05	22.70
Liberia	Both	Liver cancer	2019	4.92	3.60	7.24
Libya	Both	Liver cancer	2019	5.18	3.88	7.05
Lithuania	Both	Liver cancer	2019	3.20	2.50	3.95
Luxembourg	Both	Liver cancer	2019	4.03	3.14	5.25
Madagascar	Both	Liver cancer	2019	2.44	1.66	3.72
Malawi	Both	Liver cancer	2019	2.82	2.23	3.54
Malaysia	Both	Liver cancer	2019	6.08	4.65	7.83
Maldives	Both	Liver cancer	2019	5.47	4.31	6.70
Mali	Both	Liver cancer	2019	14.46	10.75	18.98
Malta	Both	Liver cancer	2019	2.50	2.11	2.95
Marshall Islands	Both	Liver cancer	2019	10.22	7.38	13.84
Mauritania	Both	Liver cancer	2019	4.10	3.03	5.29
Mauritius	Both	Liver cancer	2019	1.93	1.49	2.49

Mexico	Both	Liver cancer	2019	3.46	2.99	3.96
Micronesia (Federated States of)	Both	Liver cancer	2019	10.37	6.93	14.51
Monaco	Both	Liver cancer	2019	9.04	7.13	11.28
Mongolia	Both	Liver cancer	2019	105.22	82.57	131.46
Montenegro	Both	Liver cancer	2019	5.97	4.82	7.31
Morocco	Both	Liver cancer	2019	2.16	1.65	2.63
Mozambique	Both	Liver cancer	2019	3.67	2.55	4.85
Myanmar	Both	Liver cancer	2019	4.11	3.46	4.88
Namibia	Both	Liver cancer	2019	3.28	2.50	4.20
Nauru	Both	Liver cancer	2019	9.17	6.50	12.59
Nepal	Both	Liver cancer	2019	2.13	1.54	3.05
Netherlands	Both	Liver cancer	2019	3.33	2.58	4.19
New Zealand	Both	Liver cancer	2019	4.96	4.09	6.02
Nicaragua	Both	Liver cancer	2019	3.79	3.09	4.63
Niger	Both	Liver cancer	2019	0.61	0.45	0.80
Nigeria	Both	Liver cancer	2019	3.25	2.56	4.04
Niue	Both	Liver cancer	2019	7.37	5.65	9.46
North Macedonia	Both	Liver cancer	2019	8.39	6.65	10.65
Northern Mariana Islands	Both	Liver cancer	2019	7.97	6.58	9.70
Norway	Both	Liver cancer	2019	2.91	2.42	3.51
Oman	Both	Liver cancer	2019	5.27	4.47	6.30
Pakistan	Both	Liver cancer	2019	3.27	2.56	4.02
Palau	Both	Liver cancer	2019	9.97	7.68	12.94
Palestine	Both	Liver cancer	2019	6.56	5.52	7.88
Panama	Both	Liver cancer	2019	2.93	2.23	3.77
Papua New Guinea	Both	Liver cancer	2019	1.53	1.19	1.96
Paraguay	Both	Liver cancer	2019	2.05	1.54	2.67
Peru	Both	Liver cancer	2019	2.60	1.93	3.45
Philippines	Both	Liver cancer	2019	6.38	5.16	7.84
Poland	Both	Liver cancer	2019	1.95	1.63	2.31
Portugal	Both	Liver cancer	2019	4.90	3.79	6.26
Puerto Rico	Both	Liver cancer	2019	2.76	2.06	3.59
Qatar	Both	Liver cancer	2019	17.39	12.83	22.91
Republic of Korea	Both	Liver cancer	2019	22.80	18.72	27.32
Republic of Moldova	Both	Liver cancer	2019	2.30	1.95	2.70
Romania	Both	Liver cancer	2019	3.02	2.45	3.70
Russian Federation	Both	Liver cancer	2019	2.91	2.46	3.44
Rwanda	Both	Liver cancer	2019	4.40	3.48	5.65
Saint Kitts and Nevis	Both	Liver cancer	2019	3.73	3.12	4.44
Saint Lucia	Both	Liver cancer	2019	2.16	1.80	2.59
Saint Vincent and the Grenadines	Both	Liver cancer	2019	2.97	2.57	3.44
Samoa	Both	Liver cancer	2019	4.99	3.82	6.29
San Marino	Both	Liver cancer	2019	3.22	2.45	4.29
Sao Tome and Principe	Both	Liver cancer	2019	2.64	1.77	3.48
Saudi Arabia	Both	Liver cancer	2019	5.29	4.17	6.69
Senegal	Both	Liver cancer	2019	2.07	1.55	2.60
Serbia	Both	Liver cancer	2019	5.42	4.28	6.82
Seychelles	Both	Liver cancer	2019	5.56	4.68	6.58
Sierra Leone	Both	Liver cancer	2019	4.62	3.50	6.03
Singapore	Both	Liver cancer	2019	11.50	9.24	14.41
Slovakia	Both	Liver cancer	2019	3.41	2.66	4.33
Slovenia	Both	Liver cancer	2019	5.30	4.04	6.83
Solomon Islands	Both	Liver cancer	2019	5.37	4.30	6.55
Somalia	Both	Liver cancer	2019	3.33	2.15	5.91
South Africa	Both	Liver cancer	2019	5.60	4.94	6.35
South Sudan	Both	Liver cancer	2019	2.74	1.64	4.77
Spain	Both	Liver cancer	2019	6.01	4.63	7.72
Sri Lanka	Both	Liver cancer	2019	2.80	2.06	3.72

Sudan	Both	Liver cancer	2019	3.70	2.19	5.76
Suriname	Both	Liver cancer	2019	2.60	2.09	3.23
Sweden	Both	Liver cancer	2019	3.04	2.56	3.59
Switzerland	Both	Liver cancer	2019	5.79	4.44	7.59
Syrian Arab Republic	Both	Liver cancer	2019	4.50	3.42	5.90
Taiwan (Province of China)	Both	Liver cancer	2019	8.65	6.73	11.15
Tajikistan	Both	Liver cancer	2019	3.70	2.96	4.66
Thailand	Both	Liver cancer	2019	24.18	17.89	32.01
Timor-Leste	Both	Liver cancer	2019	6.19	4.18	8.57
Togo	Both	Liver cancer	2019	4.95	3.83	6.37
Tokelau	Both	Liver cancer	2019	7.32	5.29	9.90
Tonga	Both	Liver cancer	2019	24.33	17.65	31.90
Trinidad and Tobago	Both	Liver cancer	2019	2.51	1.90	3.29
Tunisia	Both	Liver cancer	2019	1.94	1.38	2.69
Turkey	Both	Liver cancer	2019	3.21	2.55	3.95
Turkmenistan	Both	Liver cancer	2019	5.55	4.38	7.01
Tuvalu	Both	Liver cancer	2019	8.47	6.24	11.32
Uganda	Both	Liver cancer	2019	6.06	4.81	7.51
Ukraine	Both	Liver cancer	2019	2.67	2.27	3.19
United Arab Emirates	Both	Liver cancer	2019	4.73	2.01	10.82
United Kingdom	Both	Liver cancer	2019	5.08	4.20	6.11
United Republic of Tanzania	Both	Liver cancer	2019	2.25	1.79	2.80
United States of America	Both	Liver cancer	2019	5.23	4.28	6.29
United States Virgin Islands	Both	Liver cancer	2019	2.49	2.06	2.95
Uruguay	Both	Liver cancer	2019	2.28	1.77	2.90
Uzbekistan	Both	Liver cancer	2019	6.34	5.27	7.46
Vanuatu	Both	Liver cancer	2019	9.05	6.21	12.85
Venezuela (Bolivarian Republic of)	Both	Liver cancer	2019	2.29	1.77	2.97
Viet Nam	Both	Liver cancer	2019	2.61	2.03	3.26
Yemen	Both	Liver cancer	2019	3.20	2.32	4.33
Zambia	Both	Liver cancer	2019	2.75	2.14	3.41
Zimbabwe	Both	Liver cancer	2019	13.64	10.27	18.13

location_name	sex_name	cause_name	year	Age-standardised death rate (per 100 000 person-years)	95% CI (lower)	95% CI (upper)
Afghanistan	Both	Liver cancer	2019	10.27	7.98	12.93
Albania	Both	Liver cancer	2019	6.84	5.04	9.09
Algeria	Both	Liver cancer	2019	2.20	1.72	2.78
American Samoa	Both	Liver cancer	2019	7.02	5.85	8.45
Andorra	Both	Liver cancer	2019	9.44	7.14	12.24
Angola	Both	Liver cancer	2019	2.59	2.09	3.23
Antigua and Barbuda	Both	Liver cancer	2019	2.85	2.44	3.31
Argentina	Both	Liver cancer	2019	2.15	2.00	2.33
Armenia	Both	Liver cancer	2019	7.07	5.88	8.38
Australia	Both	Liver cancer	2019	4.20	3.83	4.59
Austria	Both	Liver cancer	2019	4.50	4.04	4.99
Azerbaijan	Both	Liver cancer	2019	4.37	3.31	5.94
Bahamas	Both	Liver cancer	2019	3.21	2.66	3.96
Bahrain	Both	Liver cancer	2019	5.23	4.12	6.53
Bangladesh	Both	Liver cancer	2019	2.75	2.17	3.42
Barbados	Both	Liver cancer	2019	2.74	2.29	3.27
Belarus	Both	Liver cancer	2019	2.41	1.76	3.24
Belgium	Both	Liver cancer	2019	3.78	3.45	4.12
Belize	Both	Liver cancer	2019	3.21	2.75	3.70
Benin	Both	Liver cancer	2019	5.12	3.89	6.79
Bermuda	Both	Liver cancer	2019	2.05	1.70	2.51
Bhutan	Both	Liver cancer	2019	3.27	2.29	4.61
Bolivia (Plurinational State of)	Both	Liver cancer	2019	5.03	3.77	6.43
Bosnia and Herzegovina	Both	Liver cancer	2019	8.01	6.40	10.00
Botswana	Both	Liver cancer	2019	1.53	1.13	2.02
Brazil	Both	Liver cancer	2019	2.50	2.33	2.64
Brunei Darussalam	Both	Liver cancer	2019	11.53	9.82	13.38
Bulgaria	Both	Liver cancer	2019	4.62	3.68	5.71
Burkina Faso	Both	Liver cancer	2019	2.37	1.80	2.96
Burundi	Both	Liver cancer	2019	3.11	2.17	4.80
Cabo Verde	Both	Liver cancer	2019	12.34	10.20	14.87
Cambodia	Both	Liver cancer	2019	9.86	7.85	12.03
Cameroon	Both	Liver cancer	2019	0.69	0.51	0.92
Canada	Both	Liver cancer	2019	3.91	3.48	4.32
Central African Republic	Both	Liver cancer	2019	3.42	2.27	5.01
Chad	Both	Liver cancer	2019	5.68	4.37	7.29
Chile	Both	Liver cancer	2019	3.02	2.76	3.29
China	Both	Liver cancer	2019	9.41	7.95	11.13
Colombia	Both	Liver cancer	2019	2.65	2.03	3.40
Comoros	Both	Liver cancer	2019	3.01	2.12	4.65
Congo	Both	Liver cancer	2019	3.14	2.33	4.24
Cook Islands	Both	Liver cancer	2019	11.13	9.03	13.51
Costa Rica	Both	Liver cancer	2019	5.30	4.11	6.74
Côte d'Ivoire	Both	Liver cancer	2019	5.06	3.77	6.83
Croatia	Both	Liver cancer	2019	3.53	2.77	4.48
Cuba	Both	Liver cancer	2019	2.43	1.95	3.00
Cyprus	Both	Liver cancer	2019	3.42	2.95	3.94
Czechia	Both	Liver cancer	2019	2.99	2.45	3.68
Democratic People's Republic of Korea	Both	Liver cancer	2019	10.20	7.75	13.30
Democratic Republic of the Congo	Both	Liver cancer	2019	2.28	1.77	2.94
Denmark	Both	Liver cancer	2019	3.25	2.93	3.57
Djibouti	Both	Liver cancer	2019	3.49	2.29	5.59
Dominica	Both	Liver cancer	2019	3.39	2.76	4.16
Dominican Republic	Both	Liver cancer	2019	4.92	3.40	7.23
Ecuador	Both	Liver cancer	2019	3.71	2.97	4.73

Egypt	Both	Liver cancer	2019	21.25	15.44	28.92
El Salvador	Both	Liver cancer	2019	2.12	1.61	2.76
Equatorial Guinea	Both	Liver cancer	2019	3.48	2.04	5.04
Eritrea	Both	Liver cancer	2019	3.32	2.36	4.73
Estonia	Both	Liver cancer	2019	3.61	2.76	4.54
Eswatini	Both	Liver cancer	2019	19.09	5.98	33.88
Ethiopia	Both	Liver cancer	2019	3.02	2.40	3.84
Fiji	Both	Liver cancer	2019	6.23	4.86	7.83
Finland	Both	Liver cancer	2019	4.03	3.68	4.42
France	Both	Liver cancer	2019	5.80	5.10	6.56
Gabon	Both	Liver cancer	2019	3.54	2.33	5.01
Gambia	Both	Liver cancer	2019	39.51	29.01	50.99
Georgia	Both	Liver cancer	2019	3.63	2.97	4.42
Germany	Both	Liver cancer	2019	4.02	3.70	4.33
Ghana	Both	Liver cancer	2019	6.00	4.59	7.59
Greece	Both	Liver cancer	2019	3.21	2.95	3.47
Greenland	Both	Liver cancer	2019	6.35	5.03	7.96
Grenada	Both	Liver cancer	2019	3.13	2.76	3.54
Guam	Both	Liver cancer	2019	5.79	4.71	7.05
Guatemala	Both	Liver cancer	2019	4.81	3.84	6.01
Guinea	Both	Liver cancer	2019	34.05	23.98	44.01
Guinea-Bissau	Both	Liver cancer	2019	6.36	4.67	8.51
Guyana	Both	Liver cancer	2019	3.20	2.53	4.03
Haiti	Both	Liver cancer	2019	4.42	2.67	6.66
Honduras	Both	Liver cancer	2019	16.14	7.41	23.50
Hungary	Both	Liver cancer	2019	2.65	2.18	3.23
Iceland	Both	Liver cancer	2019	2.84	2.51	3.20
India	Both	Liver cancer	2019	2.75	2.32	3.27
Indonesia	Both	Liver cancer	2019	2.45	2.08	2.76
Iran (Islamic Republic of)	Both	Liver cancer	2019	3.64	3.29	4.02
Iraq	Both	Liver cancer	2019	6.07	4.72	7.47
Ireland	Both	Liver cancer	2019	3.40	3.04	3.75
Israel	Both	Liver cancer	2019	2.79	2.55	3.04
Italy	Both	Liver cancer	2019	4.80	4.36	5.17
Jamaica	Both	Liver cancer	2019	2.75	2.19	3.41
Japan	Both	Liver cancer	2019	8.78	7.80	9.42
Jordan	Both	Liver cancer	2019	2.38	1.91	2.97
Kazakhstan	Both	Liver cancer	2019	6.54	5.63	7.50
Kenya	Both	Liver cancer	2019	3.34	2.28	4.81
Kiribati	Both	Liver cancer	2019	11.95	9.29	15.08
Kuwait	Both	Liver cancer	2019	2.36	1.85	2.96
Kyrgyzstan	Both	Liver cancer	2019	2.70	2.31	3.09
Lao People's Democratic Republic	Both	Liver cancer	2019	7.11	5.40	9.01
Latvia	Both	Liver cancer	2019	2.70	2.25	3.29
Lebanon	Both	Liver cancer	2019	2.97	2.19	4.14
Lesotho	Both	Liver cancer	2019	15.17	6.45	23.71
Liberia	Both	Liver cancer	2019	5.29	3.89	7.78
Libya	Both	Liver cancer	2019	5.05	3.79	6.86
Lithuania	Both	Liver cancer	2019	3.03	2.38	3.70
Luxembourg	Both	Liver cancer	2019	3.62	2.94	4.50
Madagascar	Both	Liver cancer	2019	2.62	1.80	4.02
Malawi	Both	Liver cancer	2019	3.04	2.43	3.74
Malaysia	Both	Liver cancer	2019	6.22	4.78	7.94
Maldives	Both	Liver cancer	2019	5.63	4.46	6.89
Mali	Both	Liver cancer	2019	15.03	11.25	19.52
Malta	Both	Liver cancer	2019	2.20	1.93	2.49
Marshall Islands	Both	Liver cancer	2019	10.57	7.64	14.31

Mauritania	Both	Liver cancer	2019	4.43	3.30	5.68
Mauritius	Both	Liver cancer	2019	2.00	1.55	2.57
Mexico	Both	Liver cancer	2019	3.69	3.18	4.22
Micronesia (Federated States of)	Both	Liver cancer	2019	10.70	7.24	14.74
Monaco	Both	Liver cancer	2019	7.63	6.05	9.39
Mongolia	Both	Liver cancer	2019	115.23	91.48	142.48
Montenegro	Both	Liver cancer	2019	6.09	4.93	7.48
Morocco	Both	Liver cancer	2019	2.31	1.77	2.79
Mozambique	Both	Liver cancer	2019	3.99	2.79	5.24
Myanmar	Both	Liver cancer	2019	4.44	3.76	5.26
Namibia	Both	Liver cancer	2019	3.46	2.66	4.39
Nauru	Both	Liver cancer	2019	9.39	6.70	12.73
Nepal	Both	Liver cancer	2019	2.36	1.71	3.39
Netherlands	Both	Liver cancer	2019	2.75	2.53	2.97
New Zealand	Both	Liver cancer	2019	3.72	3.44	4.00
Nicaragua	Both	Liver cancer	2019	4.10	3.37	4.96
Niger	Both	Liver cancer	2019	0.65	0.49	0.84
Nigeria	Both	Liver cancer	2019	3.57	2.87	4.44
Niue	Both	Liver cancer	2019	7.34	5.67	9.36
North Macedonia	Both	Liver cancer	2019	8.82	7.01	11.10
Northern Mariana Islands	Both	Liver cancer	2019	7.84	6.50	9.45
Norway	Both	Liver cancer	2019	2.45	2.20	2.76
Oman	Both	Liver cancer	2019	4.75	3.98	5.69
Pakistan	Both	Liver cancer	2019	3.46	2.75	4.30
Palau	Both	Liver cancer	2019	9.79	7.59	12.53
Palestine	Both	Liver cancer	2019	6.61	5.56	7.88
Panama	Both	Liver cancer	2019	3.06	2.32	3.93
Papua New Guinea	Both	Liver cancer	2019	1.67	1.31	2.12
Paraguay	Both	Liver cancer	2019	2.19	1.64	2.82
Peru	Both	Liver cancer	2019	2.74	2.04	3.61
Philippines	Both	Liver cancer	2019	6.58	5.35	7.98
Poland	Both	Liver cancer	2019	2.06	1.73	2.44
Portugal	Both	Liver cancer	2019	4.59	4.19	4.97
Puerto Rico	Both	Liver cancer	2019	2.74	2.04	3.55
Qatar	Both	Liver cancer	2019	15.88	11.76	20.79
Republic of Korea	Both	Liver cancer	2019	16.20	14.47	17.94
Republic of Moldova	Both	Liver cancer	2019	2.40	2.03	2.81
Romania	Both	Liver cancer	2019	3.08	2.50	3.75
Russian Federation	Both	Liver cancer	2019	2.97	2.53	3.55
Rwanda	Both	Liver cancer	2019	4.72	3.78	5.98
Saint Kitts and Nevis	Both	Liver cancer	2019	3.88	3.28	4.57
Saint Lucia	Both	Liver cancer	2019	2.29	1.91	2.73
Saint Vincent and the Grenadines	Both	Liver cancer	2019	3.13	2.72	3.61
Samoa	Both	Liver cancer	2019	5.06	3.91	6.33
San Marino	Both	Liver cancer	2019	2.75	1.80	3.98
Sao Tome and Principe	Both	Liver cancer	2019	2.80	1.89	3.69
Saudi Arabia	Both	Liver cancer	2019	4.90	3.86	6.25
Senegal	Both	Liver cancer	2019	2.21	1.65	2.78
Serbia	Both	Liver cancer	2019	5.49	4.36	6.87
Seychelles	Both	Liver cancer	2019	5.70	4.81	6.72
Sierra Leone	Both	Liver cancer	2019	4.94	3.79	6.39
Singapore	Both	Liver cancer	2019	8.68	7.67	9.66
Slovakia	Both	Liver cancer	2019	3.39	2.64	4.28
Slovenia	Both	Liver cancer	2019	5.14	3.93	6.61
Solomon Islands	Both	Liver cancer	2019	5.52	4.46	6.68
Somalia	Both	Liver cancer	2019	3.61	2.35	6.40
South Africa	Both	Liver cancer	2019	5.87	5.18	6.68

South Sudan	Both	Liver cancer	2019	2.97	1.79	5.14
Spain	Both	Liver cancer	2019	5.18	4.67	5.67
Sri Lanka	Both	Liver cancer	2019	2.84	2.11	3.77
Sudan	Both	Liver cancer	2019	3.92	2.41	6.04
Suriname	Both	Liver cancer	2019	2.74	2.21	3.38
Sweden	Both	Liver cancer	2019	3.02	2.80	3.24
Switzerland	Both	Liver cancer	2019	4.43	3.97	4.91
Syrian Arab Republic	Both	Liver cancer	2019	4.41	3.35	5.77
Taiwan (Province of China)	Both	Liver cancer	2019	7.27	5.69	9.32
Tajikistan	Both	Liver cancer	2019	4.02	3.23	5.06
Thailand	Both	Liver cancer	2019	24.01	17.88	31.65
Timor-Leste	Both	Liver cancer	2019	6.60	4.53	9.09
Togo	Both	Liver cancer	2019	5.24	4.09	6.74
Tokelau	Both	Liver cancer	2019	7.48	5.46	10.06
Tonga	Both	Liver cancer	2019	24.74	18.09	32.04
Trinidad and Tobago	Both	Liver cancer	2019	2.66	2.03	3.46
Tunisia	Both	Liver cancer	2019	1.80	1.27	2.53
Turkey	Both	Liver cancer	2019	2.96	2.36	3.63
Turkmenistan	Both	Liver cancer	2019	5.59	4.41	7.07
Tuvalu	Both	Liver cancer	2019	8.72	6.51	11.52
Uganda	Both	Liver cancer	2019	6.39	5.12	7.88
Ukraine	Both	Liver cancer	2019	2.66	2.24	3.11
United Arab Emirates	Both	Liver cancer	2019	4.90	2.06	11.30
United Kingdom	Both	Liver cancer	2019	4.03	3.75	4.26
United Republic of Tanzania	Both	Liver cancer	2019	2.46	1.98	3.02
United States of America	Both	Liver cancer	2019	4.33	3.86	4.75
United States Virgin Islands	Both	Liver cancer	2019	2.60	2.17	3.07
Uruguay	Both	Liver cancer	2019	2.36	2.12	2.61
Uzbekistan	Both	Liver cancer	2019	6.68	5.58	7.83
Vanuatu	Both	Liver cancer	2019	9.36	6.41	13.20
Venezuela (Bolivarian Republic of)	Both	Liver cancer	2019	2.44	1.88	3.15
Viet Nam	Both	Liver cancer	2019	2.75	2.15	3.40
Yemen	Both	Liver cancer	2019	3.42	2.49	4.57
Zambia	Both	Liver cancer	2019	2.99	2.37	3.67
Zimbabwe	Both	Liver cancer	2019	14.03	10.58	18.67

sex_name	cause_name	year	Age-standardised incidence rate (per 100 000 person-years)	95% CI (lower)	95% CI (upper)
Male	Stomach cancer	1990	30.42	28.41	32.49
Female	Stomach cancer	1990	15.81	14.72	16.82
Both	Stomach cancer	1990	22.44	21.21	23.59
Male	Stomach cancer	1991	30.03	28.21	32.08
Female	Stomach cancer	1991	15.52	14.55	16.49
Both	Stomach cancer	1991	22.12	20.96	23.31
Male	Stomach cancer	1992	29.71	27.87	31.60
Female	Stomach cancer	1992	15.29	14.34	16.33
Both	Stomach cancer	1992	21.85	20.75	22.99
Male	Stomach cancer	1993	29.60	27.90	31.25
Female	Stomach cancer	1993	15.22	14.33	16.24
Both	Stomach cancer	1993	21.78	20.72	22.77
Male	Stomach cancer	1994	29.08	27.59	30.69
Female	Stomach cancer	1994	14.93	14.05	15.84
Both	Stomach cancer	1994	21.39	20.47	22.43
Male	Stomach cancer	1995	28.62	27.20	30.18
Female	Stomach cancer	1995	14.55	13.74	15.40
Both	Stomach cancer	1995	20.97	20.07	21.88
Male	Stomach cancer	1996	27.96	26.55	29.41
Female	Stomach cancer	1996	14.18	13.34	15.01
Both	Stomach cancer	1996	20.47	19.54	21.39
Male	Stomach cancer	1997	27.42	26.14	28.88
Female	Stomach cancer	1997	13.88	13.06	14.68
Both	Stomach cancer	1997	20.06	19.17	20.96
Male	Stomach cancer	1998	27.30	25.99	28.68
Female	Stomach cancer	1998	13.66	12.91	14.46
Both	Stomach cancer	1998	19.89	19.01	20.77
Male	Stomach cancer	1999	27.30	25.92	28.67
Female	Stomach cancer	1999	13.63	12.85	14.38
Both	Stomach cancer	1999	19.87	18.99	20.70
Male	Stomach cancer	2000	27.42	26.06	28.90
Female	Stomach cancer	2000	13.53	12.73	14.30
Both	Stomach cancer	2000	19.89	18.98	20.80
Male	Stomach cancer	2001	27.54	25.99	29.09
Female	Stomach cancer	2001	13.42	12.52	14.23
Both	Stomach cancer	2001	19.90	18.85	20.85
Male	Stomach cancer	2002	27.92	26.41	29.53
Female	Stomach cancer	2002	13.47	12.63	14.30
Both	Stomach cancer	2002	20.11	19.07	21.04
Male	Stomach cancer	2003	28.43	26.85	30.17
Female	Stomach cancer	2003	13.50	12.66	14.26
Both	Stomach cancer	2003	20.38	19.35	21.36
Male	Stomach cancer	2004	28.72	27.10	30.56
Female	Stomach cancer	2004	13.36	12.42	14.14
Both	Stomach cancer	2004	20.44	19.34	21.48
Male	Stomach cancer	2005	28.55	26.89	30.27
Female	Stomach cancer	2005	13.10	12.15	13.90
Both	Stomach cancer	2005	20.23	19.10	21.28

Male	Stomach cancer	2006	27.51	25.90	29.13
Female	Stomach cancer	2006	12.58	11.69	13.29
Both	Stomach cancer	2006	19.48	18.45	20.40
Male	Stomach cancer	2007	27.01	25.51	28.75
Female	Stomach cancer	2007	12.22	11.35	12.91
Both	Stomach cancer	2007	19.06	18.08	20.07
Male	Stomach cancer	2008	26.77	25.16	28.34
Female	Stomach cancer	2008	11.95	11.08	12.61
Both	Stomach cancer	2008	18.81	17.78	19.75
Male	Stomach cancer	2009	26.48	24.83	28.09
Female	Stomach cancer	2009	11.64	10.77	12.28
Both	Stomach cancer	2009	18.50	17.45	19.43
Male	Stomach cancer	2010	26.23	24.38	28.08
Female	Stomach cancer	2010	11.39	10.58	12.05
Both	Stomach cancer	2010	18.25	17.14	19.22
Male	Stomach cancer	2011	25.60	23.84	27.31
Female	Stomach cancer	2011	11.09	10.27	11.82
Both	Stomach cancer	2011	17.81	16.71	18.81
Male	Stomach cancer	2012	25.04	23.23	26.80
Female	Stomach cancer	2012	10.71	9.81	11.36
Both	Stomach cancer	2012	17.34	16.26	18.30
Male	Stomach cancer	2013	24.33	22.51	26.23
Female	Stomach cancer	2013	10.42	9.58	11.05
Both	Stomach cancer	2013	16.86	15.76	17.94
Male	Stomach cancer	2014	23.77	21.88	25.58
Female	Stomach cancer	2014	10.20	9.38	10.86
Both	Stomach cancer	2014	16.48	15.37	17.46
Male	Stomach cancer	2015	23.32	21.31	25.35
Female	Stomach cancer	2015	10.01	9.15	10.74
Both	Stomach cancer	2015	16.17	15.02	17.23
Male	Stomach cancer	2016	22.91	20.95	25.00
Female	Stomach cancer	2016	9.87	9.03	10.62
Both	Stomach cancer	2016	15.90	14.71	17.03
Male	Stomach cancer	2017	22.58	20.33	24.96
Female	Stomach cancer	2017	9.75	8.82	10.65
Both	Stomach cancer	2017	15.69	14.40	16.97
Male	Stomach cancer	2018	22.45	19.97	24.94
Female	Stomach cancer	2018	9.73	8.69	10.67
Both	Stomach cancer	2018	15.63	14.26	17.02
Male	Stomach cancer	2019	22.39	19.80	25.34
Female	Stomach cancer	2019	9.71	8.67	10.72
Both	Stomach cancer	2019	15.59	14.11	17.15

location_name	sex_name	cause_name	year	Age-standardised incidence rate (per 100 000 person-years)	95% CI (lower)	95% CI (upper)
High SDI	Male	Stomach cancer	1990	32.11	31.18	32.72
High SDI	Female	Stomach cancer	1990	14.43	13.75	14.81
High SDI	Both	Stomach cancer	1990	21.99	21.24	22.41
High-middle SDI	Male	Stomach cancer	1990	39.32	36.44	42.21
High-middle SDI	Female	Stomach cancer	1990	18.18	16.94	19.32
High-middle SDI	Both	Stomach cancer	1990	27.30	25.74	28.82
Low SDI	Male	Stomach cancer	1990	13.68	12.03	15.25
Low SDI	Female	Stomach cancer	1990	8.95	7.32	10.37
Low SDI	Both	Stomach cancer	1990	11.33	10.01	12.51
Low-middle SDI	Male	Stomach cancer	1990	16.40	14.79	17.75
Low-middle SDI	Female	Stomach cancer	1990	11.91	10.38	13.35
Low-middle SDI	Both	Stomach cancer	1990	14.16	12.98	15.20
Middle SDI	Male	Stomach cancer	1990	31.83	27.80	36.21
Middle SDI	Female	Stomach cancer	1990	17.71	15.72	19.77
Middle SDI	Both	Stomach cancer	1990	24.50	22.18	27.08
High SDI	Male	Stomach cancer	1991	31.80	30.86	32.39
High SDI	Female	Stomach cancer	1991	14.21	13.47	14.60
High SDI	Both	Stomach cancer	1991	21.75	20.98	22.16
High-middle SDI	Male	Stomach cancer	1991	38.56	35.91	41.43
High-middle SDI	Female	Stomach cancer	1991	17.75	16.55	18.90
High-middle SDI	Both	Stomach cancer	1991	26.75	25.32	28.21
Low SDI	Male	Stomach cancer	1991	12.11	10.65	13.50
Low SDI	Female	Stomach cancer	1991	8.85	7.32	10.29
Low SDI	Both	Stomach cancer	1991	10.49	9.30	11.56
Low-middle SDI	Male	Stomach cancer	1991	16.08	14.59	17.34
Low-middle SDI	Female	Stomach cancer	1991	11.71	10.26	13.09
Low-middle SDI	Both	Stomach cancer	1991	13.90	12.86	14.91
Middle SDI	Male	Stomach cancer	1991	26.13	23.11	29.37
Middle SDI	Female	Stomach cancer	1991	17.45	15.52	19.47
Middle SDI	Both	Stomach cancer	1991	21.64	19.76	23.75
High SDI	Male	Stomach cancer	1992	31.53	30.65	32.11
High SDI	Female	Stomach cancer	1992	13.96	13.21	14.36
High SDI	Both	Stomach cancer	1992	21.50	20.71	21.93
High-middle SDI	Male	Stomach cancer	1992	38.21	35.66	40.85
High-middle SDI	Female	Stomach cancer	1992	17.37	16.26	18.49
High-middle SDI	Both	Stomach cancer	1992	26.41	24.94	27.80
Low SDI	Male	Stomach cancer	1992	11.98	10.52	13.36
Low SDI	Female	Stomach cancer	1992	8.81	7.27	10.23
Low SDI	Both	Stomach cancer	1992	10.41	9.24	11.48
Low-middle SDI	Male	Stomach cancer	1992	15.91	14.45	17.18
Low-middle SDI	Female	Stomach cancer	1992	11.69	10.21	13.18
Low-middle SDI	Both	Stomach cancer	1992	13.80	12.70	14.83
Middle SDI	Male	Stomach cancer	1992	26.11	23.19	29.33
Middle SDI	Female	Stomach cancer	1992	17.24	15.52	19.25
Middle SDI	Both	Stomach cancer	1992	21.52	19.70	23.67
High SDI	Male	Stomach cancer	1993	31.40	30.48	31.98
High SDI	Female	Stomach cancer	1993	13.82	13.07	14.23
High SDI	Both	Stomach cancer	1993	21.39	20.58	21.82
High-middle SDI	Male	Stomach cancer	1993	38.34	35.86	40.68
High-middle SDI	Female	Stomach cancer	1993	17.53	16.49	18.63

High-middle SDI	Both	Stomach cancer	1993		26.59	25.30	27.77
Low SDI	Male	Stomach cancer	1993		11.89	10.39	13.26
Low SDI	Female	Stomach cancer	1993		8.72	7.23	10.13
Low SDI	Both	Stomach cancer	1993		10.31	9.15	11.36
Low-middle SDI	Male	Stomach cancer	1993		15.78	14.43	16.96
Low-middle SDI	Female	Stomach cancer	1993		11.51	10.12	12.92
Low-middle SDI	Both	Stomach cancer	1993		13.64	12.69	14.63
Middle SDI	Male	Stomach cancer	1993		25.59	22.84	28.41
Middle SDI	Female	Stomach cancer	1993		17.07	15.36	19.24
Middle SDI	Both	Stomach cancer	1993		21.19	19.46	23.07
High SDI	Male	Stomach cancer	1994		30.79	29.90	31.38
High SDI	Female	Stomach cancer	1994		13.43	12.66	13.84
High SDI	Both	Stomach cancer	1994		20.93	20.12	21.35
High-middle SDI	Male	Stomach cancer	1994		37.79	35.63	40.29
High-middle SDI	Female	Stomach cancer	1994		17.26	16.25	18.25
High-middle SDI	Both	Stomach cancer	1994		26.22	25.01	27.52
Low SDI	Male	Stomach cancer	1994		11.78	10.36	13.15
Low SDI	Female	Stomach cancer	1994		8.64	7.23	9.95
Low SDI	Both	Stomach cancer	1994		10.22	9.09	11.20
Low-middle SDI	Male	Stomach cancer	1994		15.61	14.36	16.77
Low-middle SDI	Female	Stomach cancer	1994		11.34	10.06	12.60
Low-middle SDI	Both	Stomach cancer	1994		13.47	12.51	14.39
Middle SDI	Male	Stomach cancer	1994		25.30	22.89	27.82
Middle SDI	Female	Stomach cancer	1994		16.73	15.12	18.71
Middle SDI	Both	Stomach cancer	1994		20.87	19.43	22.71
High SDI	Male	Stomach cancer	1995		30.47	29.53	31.12
High SDI	Female	Stomach cancer	1995		13.28	12.47	13.70
High SDI	Both	Stomach cancer	1995		20.71	19.86	21.16
High-middle SDI	Male	Stomach cancer	1995		36.66	34.67	38.87
High-middle SDI	Female	Stomach cancer	1995		16.54	15.61	17.46
High-middle SDI	Both	Stomach cancer	1995		25.31	24.17	26.44
Low SDI	Male	Stomach cancer	1995		11.69	10.22	12.95
Low SDI	Female	Stomach cancer	1995		8.54	7.17	9.83
Low SDI	Both	Stomach cancer	1995		10.12	9.00	11.11
Low-middle SDI	Male	Stomach cancer	1995		15.37	14.19	16.45
Low-middle SDI	Female	Stomach cancer	1995		11.13	9.81	12.35
Low-middle SDI	Both	Stomach cancer	1995		13.24	12.32	14.13
Middle SDI	Male	Stomach cancer	1995		25.13	22.96	27.79
Middle SDI	Female	Stomach cancer	1995		16.31	14.81	18.12
Middle SDI	Both	Stomach cancer	1995		20.57	19.17	22.23
High SDI	Male	Stomach cancer	1996		29.52	28.51	30.12
High SDI	Female	Stomach cancer	1996		12.85	12.00	13.27
High SDI	Both	Stomach cancer	1996		20.07	19.20	20.52
High-middle SDI	Male	Stomach cancer	1996		35.25	33.35	37.22
High-middle SDI	Female	Stomach cancer	1996		15.88	15.02	16.76
High-middle SDI	Both	Stomach cancer	1996		24.32	23.25	25.41
Low SDI	Male	Stomach cancer	1996		11.58	10.25	12.83
Low SDI	Female	Stomach cancer	1996		8.46	7.12	9.79
Low SDI	Both	Stomach cancer	1996		10.02	8.91	10.98
Low-middle SDI	Male	Stomach cancer	1996		15.27	14.06	16.41
Low-middle SDI	Female	Stomach cancer	1996		11.07	9.80	12.14
Low-middle SDI	Both	Stomach cancer	1996		13.15	12.22	14.03

Middle SDI	Male	Stomach cancer	1996		24.80	22.78	27.13
Middle SDI	Female	Stomach cancer	1996		16.07	14.73	17.85
Middle SDI	Both	Stomach cancer	1996		20.29	18.91	21.84
High SDI	Male	Stomach cancer	1997		28.63	27.67	29.24
High SDI	Female	Stomach cancer	1997		12.53	11.69	12.95
High SDI	Both	Stomach cancer	1997		19.51	18.66	19.97
High-middle SDI	Male	Stomach cancer	1997		34.18	32.30	36.22
High-middle SDI	Female	Stomach cancer	1997		15.34	14.50	16.20
High-middle SDI	Both	Stomach cancer	1997		23.55	22.44	24.59
Low SDI	Male	Stomach cancer	1997		11.53	10.15	12.80
Low SDI	Female	Stomach cancer	1997		8.41	7.13	9.61
Low SDI	Both	Stomach cancer	1997		9.97	8.90	10.92
Low-middle SDI	Male	Stomach cancer	1997		15.46	14.38	16.52
Low-middle SDI	Female	Stomach cancer	1997		11.16	9.86	12.35
Low-middle SDI	Both	Stomach cancer	1997		13.29	12.39	14.16
Middle SDI	Male	Stomach cancer	1997		24.58	22.58	26.82
Middle SDI	Female	Stomach cancer	1997		15.76	14.50	17.37
Middle SDI	Both	Stomach cancer	1997		20.02	18.82	21.48
High SDI	Male	Stomach cancer	1998		28.49	27.53	29.10
High SDI	Female	Stomach cancer	1998		12.36	11.50	12.78
High SDI	Both	Stomach cancer	1998		19.37	18.49	19.83
High-middle SDI	Male	Stomach cancer	1998		33.74	31.90	35.86
High-middle SDI	Female	Stomach cancer	1998		14.96	14.13	15.81
High-middle SDI	Both	Stomach cancer	1998		23.14	22.09	24.26
Low SDI	Male	Stomach cancer	1998		11.41	10.07	12.64
Low SDI	Female	Stomach cancer	1998		8.36	7.14	9.64
Low SDI	Both	Stomach cancer	1998		9.88	8.80	10.79
Low-middle SDI	Male	Stomach cancer	1998		15.46	14.31	16.49
Low-middle SDI	Female	Stomach cancer	1998		11.10	9.85	12.20
Low-middle SDI	Both	Stomach cancer	1998		13.25	12.37	14.08
Middle SDI	Male	Stomach cancer	1998		24.64	22.71	26.90
Middle SDI	Female	Stomach cancer	1998		15.50	14.27	17.07
Middle SDI	Both	Stomach cancer	1998		19.91	18.73	21.38
High SDI	Male	Stomach cancer	1999		28.02	27.07	28.66
High SDI	Female	Stomach cancer	1999		12.18	11.33	12.61
High SDI	Both	Stomach cancer	1999		19.07	18.20	19.55
High-middle SDI	Male	Stomach cancer	1999		34.13	32.28	36.11
High-middle SDI	Female	Stomach cancer	1999		15.02	14.16	15.81
High-middle SDI	Both	Stomach cancer	1999		23.36	22.30	24.38
Low SDI	Male	Stomach cancer	1999		11.20	9.85	12.46
Low SDI	Female	Stomach cancer	1999		8.26	7.08	9.42
Low SDI	Both	Stomach cancer	1999		9.72	8.70	10.63
Low-middle SDI	Male	Stomach cancer	1999		15.23	14.07	16.21
Low-middle SDI	Female	Stomach cancer	1999		10.96	9.82	12.02
Low-middle SDI	Both	Stomach cancer	1999		13.07	12.22	13.83
Middle SDI	Male	Stomach cancer	1999		24.79	22.81	26.99
Middle SDI	Female	Stomach cancer	1999		15.57	14.33	17.09
Middle SDI	Both	Stomach cancer	1999		20.02	18.70	21.44
High SDI	Male	Stomach cancer	2000		26.94	25.97	27.56
High SDI	Female	Stomach cancer	2000		11.80	10.97	12.24
High SDI	Both	Stomach cancer	2000		18.40	17.56	18.88
High-middle SDI	Male	Stomach cancer	2000		34.73	32.69	36.89

High-middle SDI	Female	Stomach cancer	2000		14.97	14.16	15.79
High-middle SDI	Both	Stomach cancer	2000		23.63	22.58	24.77
Low SDI	Male	Stomach cancer	2000		11.13	9.79	12.33
Low SDI	Female	Stomach cancer	2000		8.18	7.03	9.34
Low SDI	Both	Stomach cancer	2000		9.64	8.61	10.53
Low-middle SDI	Male	Stomach cancer	2000		15.38	14.26	16.44
Low-middle SDI	Female	Stomach cancer	2000		10.88	9.77	11.93
Low-middle SDI	Both	Stomach cancer	2000		13.10	12.30	13.91
Middle SDI	Male	Stomach cancer	2000		25.37	23.37	27.66
Middle SDI	Female	Stomach cancer	2000		15.72	14.50	17.19
Middle SDI	Both	Stomach cancer	2000		20.39	19.08	21.85
High SDI	Male	Stomach cancer	2001		26.19	25.24	26.82
High SDI	Female	Stomach cancer	2001		11.45	10.61	11.87
High SDI	Both	Stomach cancer	2001		17.89	17.04	18.37
High-middle SDI	Male	Stomach cancer	2001		35.11	32.87	37.48
High-middle SDI	Female	Stomach cancer	2001		14.89	14.00	15.77
High-middle SDI	Both	Stomach cancer	2001		23.77	22.54	25.02
Low SDI	Male	Stomach cancer	2001		11.03	9.69	12.21
Low SDI	Female	Stomach cancer	2001		8.19	7.07	9.30
Low SDI	Both	Stomach cancer	2001		9.59	8.60	10.47
Low-middle SDI	Male	Stomach cancer	2001		15.47	14.38	16.51
Low-middle SDI	Female	Stomach cancer	2001		10.87	9.80	11.91
Low-middle SDI	Both	Stomach cancer	2001		13.13	12.35	13.91
Middle SDI	Male	Stomach cancer	2001		25.82	23.63	28.20
Middle SDI	Female	Stomach cancer	2001		15.77	14.38	17.42
Middle SDI	Both	Stomach cancer	2001		20.64	19.20	22.29
High SDI	Male	Stomach cancer	2002		25.66	24.65	26.29
High SDI	Female	Stomach cancer	2002		11.22	10.36	11.66
High SDI	Both	Stomach cancer	2002		17.55	16.67	18.03
High-middle SDI	Male	Stomach cancer	2002		35.94	33.63	38.42
High-middle SDI	Female	Stomach cancer	2002		15.05	14.15	16.01
High-middle SDI	Both	Stomach cancer	2002		24.24	22.90	25.47
Low SDI	Male	Stomach cancer	2002		10.93	9.67	12.06
Low SDI	Female	Stomach cancer	2002		8.18	7.09	9.21
Low SDI	Both	Stomach cancer	2002		9.53	8.57	10.39
Low-middle SDI	Male	Stomach cancer	2002		15.55	14.51	16.48
Low-middle SDI	Female	Stomach cancer	2002		10.93	9.85	11.95
Low-middle SDI	Both	Stomach cancer	2002		13.20	12.42	13.96
Middle SDI	Male	Stomach cancer	2002		26.75	24.42	29.32
Middle SDI	Female	Stomach cancer	2002		16.04	14.64	17.59
Middle SDI	Both	Stomach cancer	2002		21.22	19.87	22.75
High SDI	Male	Stomach cancer	2003		25.58	24.51	26.23
High SDI	Female	Stomach cancer	2003		11.08	10.19	11.52
High SDI	Both	Stomach cancer	2003		17.45	16.54	17.94
High-middle SDI	Male	Stomach cancer	2003		36.86	34.31	39.51
High-middle SDI	Female	Stomach cancer	2003		15.23	14.31	16.13
High-middle SDI	Both	Stomach cancer	2003		24.77	23.37	26.15
Low SDI	Male	Stomach cancer	2003		10.87	9.65	11.94
Low SDI	Female	Stomach cancer	2003		8.12	7.06	9.19
Low SDI	Both	Stomach cancer	2003		9.47	8.54	10.27
Low-middle SDI	Male	Stomach cancer	2003		15.58	14.50	16.68
Low-middle SDI	Female	Stomach cancer	2003		10.71	9.74	11.71

Low-middle SDI	Both	Stomach cancer	2003		13.10	12.32	13.85
Middle SDI	Male	Stomach cancer	2003		27.34	25.14	29.97
Middle SDI	Female	Stomach cancer	2003		16.28	14.96	17.83
Middle SDI	Both	Stomach cancer	2003		21.64	20.24	23.17
High SDI	Male	Stomach cancer	2004		24.83	23.74	25.46
High SDI	Female	Stomach cancer	2004		10.83	9.96	11.28
High SDI	Both	Stomach cancer	2004		16.99	16.07	17.48
High-middle SDI	Male	Stomach cancer	2004		37.56	35.05	40.39
High-middle SDI	Female	Stomach cancer	2004		15.11	14.08	16.07
High-middle SDI	Both	Stomach cancer	2004		25.03	23.57	26.44
Low SDI	Male	Stomach cancer	2004		10.68	9.44	11.79
Low SDI	Female	Stomach cancer	2004		8.02	7.00	9.05
Low SDI	Both	Stomach cancer	2004		9.32	8.44	10.13
Low-middle SDI	Male	Stomach cancer	2004		15.41	14.37	16.46
Low-middle SDI	Female	Stomach cancer	2004		10.46	9.55	11.37
Low-middle SDI	Both	Stomach cancer	2004		12.89	12.15	13.65
Middle SDI	Male	Stomach cancer	2004		28.07	25.58	30.89
Middle SDI	Female	Stomach cancer	2004		16.29	14.91	17.80
Middle SDI	Both	Stomach cancer	2004		22.01	20.52	23.66
High SDI	Male	Stomach cancer	2005		24.52	23.42	25.16
High SDI	Female	Stomach cancer	2005		10.62	9.73	11.07
High SDI	Both	Stomach cancer	2005		16.75	15.80	17.24
High-middle SDI	Male	Stomach cancer	2005		37.47	34.90	40.20
High-middle SDI	Female	Stomach cancer	2005		14.86	13.79	15.86
High-middle SDI	Both	Stomach cancer	2005		24.86	23.39	26.25
Low SDI	Male	Stomach cancer	2005		10.53	9.33	11.60
Low SDI	Female	Stomach cancer	2005		7.97	6.97	9.02
Low SDI	Both	Stomach cancer	2005		9.22	8.33	10.05
Low-middle SDI	Male	Stomach cancer	2005		15.38	14.35	16.35
Low-middle SDI	Female	Stomach cancer	2005		10.44	9.51	11.28
Low-middle SDI	Both	Stomach cancer	2005		12.85	12.10	13.58
Middle SDI	Male	Stomach cancer	2005		27.55	25.35	30.14
Middle SDI	Female	Stomach cancer	2005		15.82	14.54	17.29
Middle SDI	Both	Stomach cancer	2005		21.51	20.10	23.13
High SDI	Male	Stomach cancer	2006		23.73	22.62	24.38
High SDI	Female	Stomach cancer	2006		10.24	9.35	10.68
High SDI	Both	Stomach cancer	2006		16.20	15.23	16.73
High-middle SDI	Male	Stomach cancer	2006		35.61	33.22	38.11
High-middle SDI	Female	Stomach cancer	2006		14.15	13.09	15.02
High-middle SDI	Both	Stomach cancer	2006		23.65	22.31	24.95
Low SDI	Male	Stomach cancer	2006		10.41	9.20	11.57
Low SDI	Female	Stomach cancer	2006		7.87	6.85	8.93
Low SDI	Both	Stomach cancer	2006		9.11	8.22	9.91
Low-middle SDI	Male	Stomach cancer	2006		15.22	14.25	16.17
Low-middle SDI	Female	Stomach cancer	2006		10.25	9.38	11.14
Low-middle SDI	Both	Stomach cancer	2006		12.68	11.94	13.42
Middle SDI	Male	Stomach cancer	2006		26.47	24.33	28.85
Middle SDI	Female	Stomach cancer	2006		15.10	13.93	16.40
Middle SDI	Both	Stomach cancer	2006		20.61	19.34	21.91
High SDI	Male	Stomach cancer	2007		23.33	22.17	23.97
High SDI	Female	Stomach cancer	2007		10.00	9.11	10.46
High SDI	Both	Stomach cancer	2007		15.91	14.94	16.43

High-middle SDI	Male	Stomach cancer	2007		34.65	32.19	37.25
High-middle SDI	Female	Stomach cancer	2007		13.73	12.67	14.62
High-middle SDI	Both	Stomach cancer	2007		23.01	21.66	24.32
Low SDI	Male	Stomach cancer	2007		10.40	9.24	11.47
Low SDI	Female	Stomach cancer	2007		7.72	6.81	8.69
Low SDI	Both	Stomach cancer	2007		9.03	8.18	9.87
Low-middle SDI	Male	Stomach cancer	2007		15.15	14.21	16.16
Low-middle SDI	Female	Stomach cancer	2007		10.09	9.25	10.92
Low-middle SDI	Both	Stomach cancer	2007		12.55	11.84	13.30
Middle SDI	Male	Stomach cancer	2007		26.14	24.04	28.47
Middle SDI	Female	Stomach cancer	2007		14.51	13.31	15.61
Middle SDI	Both	Stomach cancer	2007		20.14	18.96	21.58
High SDI	Male	Stomach cancer	2008		22.97	21.82	23.64
High SDI	Female	Stomach cancer	2008		9.82	8.93	10.30
High SDI	Both	Stomach cancer	2008		15.67	14.67	16.21
High-middle SDI	Male	Stomach cancer	2008		34.23	31.93	36.70
High-middle SDI	Female	Stomach cancer	2008		13.40	12.42	14.31
High-middle SDI	Both	Stomach cancer	2008		22.65	21.41	23.99
Low SDI	Male	Stomach cancer	2008		10.61	9.41	11.73
Low SDI	Female	Stomach cancer	2008		7.65	6.73	8.62
Low SDI	Both	Stomach cancer	2008		9.09	8.24	9.94
Low-middle SDI	Male	Stomach cancer	2008		15.11	14.17	16.06
Low-middle SDI	Female	Stomach cancer	2008		9.89	9.01	10.79
Low-middle SDI	Both	Stomach cancer	2008		12.43	11.67	13.10
Middle SDI	Male	Stomach cancer	2008		26.01	23.87	28.28
Middle SDI	Female	Stomach cancer	2008		14.11	13.02	15.16
Middle SDI	Both	Stomach cancer	2008		19.85	18.58	21.20
High SDI	Male	Stomach cancer	2009		22.57	21.36	23.23
High SDI	Female	Stomach cancer	2009		9.56	8.66	10.03
High SDI	Both	Stomach cancer	2009		15.36	14.33	15.90
High-middle SDI	Male	Stomach cancer	2009		33.59	31.06	36.13
High-middle SDI	Female	Stomach cancer	2009		13.02	12.04	13.89
High-middle SDI	Both	Stomach cancer	2009		22.15	20.66	23.46
Low SDI	Male	Stomach cancer	2009		10.50	9.31	11.57
Low SDI	Female	Stomach cancer	2009		7.56	6.67	8.49
Low SDI	Both	Stomach cancer	2009		8.99	8.13	9.82
Low-middle SDI	Male	Stomach cancer	2009		14.83	13.84	15.70
Low-middle SDI	Female	Stomach cancer	2009		9.60	8.81	10.40
Low-middle SDI	Both	Stomach cancer	2009		12.14	11.45	12.82
Middle SDI	Male	Stomach cancer	2009		26.05	23.80	28.28
Middle SDI	Female	Stomach cancer	2009		13.80	12.71	14.85
Middle SDI	Both	Stomach cancer	2009		19.70	18.37	21.02
High SDI	Male	Stomach cancer	2010		22.08	20.87	22.77
High SDI	Female	Stomach cancer	2010		9.31	8.41	9.80
High SDI	Both	Stomach cancer	2010		15.02	13.98	15.58
High-middle SDI	Male	Stomach cancer	2010		33.32	30.76	36.04
High-middle SDI	Female	Stomach cancer	2010		12.69	11.67	13.61
High-middle SDI	Both	Stomach cancer	2010		21.85	20.37	23.22
Low SDI	Male	Stomach cancer	2010		10.32	9.14	11.43
Low SDI	Female	Stomach cancer	2010		7.44	6.57	8.38
Low SDI	Both	Stomach cancer	2010		8.83	7.98	9.69
Low-middle SDI	Male	Stomach cancer	2010		14.60	13.69	15.65

Low-middle SDI	Female	Stomach cancer	2010		9.47	8.72	10.25
Low-middle SDI	Both	Stomach cancer	2010		11.96	11.26	12.65
Middle SDI	Male	Stomach cancer	2010		25.94	23.58	28.44
Middle SDI	Female	Stomach cancer	2010		13.47	12.34	14.58
Middle SDI	Both	Stomach cancer	2010		19.46	18.03	20.78
High SDI	Male	Stomach cancer	2011		21.50	20.27	22.21
High SDI	Female	Stomach cancer	2011		9.17	8.24	9.66
High SDI	Both	Stomach cancer	2011		14.69	13.64	15.26
High-middle SDI	Male	Stomach cancer	2011		32.32	29.71	35.03
High-middle SDI	Female	Stomach cancer	2011		12.19	11.25	13.21
High-middle SDI	Both	Stomach cancer	2011		21.14	19.68	22.59
Low SDI	Male	Stomach cancer	2011		10.18	8.97	11.29
Low SDI	Female	Stomach cancer	2011		7.35	6.51	8.28
Low SDI	Both	Stomach cancer	2011		8.72	7.90	9.54
Low-middle SDI	Male	Stomach cancer	2011		14.27	13.33	15.17
Low-middle SDI	Female	Stomach cancer	2011		9.37	8.56	10.14
Low-middle SDI	Both	Stomach cancer	2011		11.74	11.05	12.44
Middle SDI	Male	Stomach cancer	2011		25.42	23.19	27.91
Middle SDI	Female	Stomach cancer	2011		13.03	11.90	14.17
Middle SDI	Both	Stomach cancer	2011		18.98	17.60	20.39
High SDI	Male	Stomach cancer	2012		20.77	19.59	21.48
High SDI	Female	Stomach cancer	2012		8.82	7.90	9.31
High SDI	Both	Stomach cancer	2012		14.18	13.16	14.74
High-middle SDI	Male	Stomach cancer	2012		31.63	28.87	34.64
High-middle SDI	Female	Stomach cancer	2012		11.67	10.71	12.63
High-middle SDI	Both	Stomach cancer	2012		20.55	19.01	22.07
Low SDI	Male	Stomach cancer	2012		10.06	8.92	11.14
Low SDI	Female	Stomach cancer	2012		7.38	6.56	8.26
Low SDI	Both	Stomach cancer	2012		8.67	7.86	9.52
Low-middle SDI	Male	Stomach cancer	2012		14.02	13.15	14.92
Low-middle SDI	Female	Stomach cancer	2012		9.22	8.47	9.96
Low-middle SDI	Both	Stomach cancer	2012		11.55	10.88	12.18
Middle SDI	Male	Stomach cancer	2012		24.84	22.52	27.24
Middle SDI	Female	Stomach cancer	2012		12.47	11.41	13.50
Middle SDI	Both	Stomach cancer	2012		18.40	17.05	19.74
High SDI	Male	Stomach cancer	2013		20.14	18.88	20.84
High SDI	Female	Stomach cancer	2013		8.60	7.67	9.08
High SDI	Both	Stomach cancer	2013		13.78	12.72	14.34
High-middle SDI	Male	Stomach cancer	2013		30.70	28.07	33.70
High-middle SDI	Female	Stomach cancer	2013		11.25	10.30	12.16
High-middle SDI	Both	Stomach cancer	2013		19.91	18.51	21.40
Low SDI	Male	Stomach cancer	2013		9.98	8.87	11.06
Low SDI	Female	Stomach cancer	2013		7.29	6.48	8.18
Low SDI	Both	Stomach cancer	2013		8.59	7.75	9.47
Low-middle SDI	Male	Stomach cancer	2013		13.76	12.88	14.70
Low-middle SDI	Female	Stomach cancer	2013		9.19	8.42	9.96
Low-middle SDI	Both	Stomach cancer	2013		11.39	10.72	12.04
Middle SDI	Male	Stomach cancer	2013		24.06	21.64	26.69
Middle SDI	Female	Stomach cancer	2013		12.07	11.01	13.16
Middle SDI	Both	Stomach cancer	2013		17.80	16.44	19.27
High SDI	Male	Stomach cancer	2014		19.40	18.17	20.09
High SDI	Female	Stomach cancer	2014		8.37	7.47	8.86

High SDI	Both	Stomach cancer	2014		13.34	12.31	13.89
High-middle SDI	Male	Stomach cancer	2014		30.19	27.22	33.18
High-middle SDI	Female	Stomach cancer	2014		10.98	10.06	11.93
High-middle SDI	Both	Stomach cancer	2014		19.55	18.00	21.07
Low SDI	Male	Stomach cancer	2014		9.69	8.59	10.79
Low SDI	Female	Stomach cancer	2014		7.27	6.47	8.17
Low SDI	Both	Stomach cancer	2014		8.43	7.64	9.27
Low-middle SDI	Male	Stomach cancer	2014		13.54	12.61	14.44
Low-middle SDI	Female	Stomach cancer	2014		9.17	8.39	9.99
Low-middle SDI	Both	Stomach cancer	2014		11.27	10.62	11.93
Middle SDI	Male	Stomach cancer	2014		23.48	21.04	26.11
Middle SDI	Female	Stomach cancer	2014		11.77	10.64	12.82
Middle SDI	Both	Stomach cancer	2014		17.35	15.92	18.74
High SDI	Male	Stomach cancer	2015		18.89	17.63	19.60
High SDI	Female	Stomach cancer	2015		8.14	7.26	8.63
High SDI	Both	Stomach cancer	2015		12.99	11.95	13.56
High-middle SDI	Male	Stomach cancer	2015		29.94	26.56	33.40
High-middle SDI	Female	Stomach cancer	2015		10.82	9.88	11.84
High-middle SDI	Both	Stomach cancer	2015		19.37	17.69	21.01
Low SDI	Male	Stomach cancer	2015		9.58	8.50	10.71
Low SDI	Female	Stomach cancer	2015		7.19	6.39	8.08
Low SDI	Both	Stomach cancer	2015		8.34	7.54	9.17
Low-middle SDI	Male	Stomach cancer	2015		13.32	12.39	14.25
Low-middle SDI	Female	Stomach cancer	2015		9.21	8.41	10.07
Low-middle SDI	Both	Stomach cancer	2015		11.18	10.52	11.88
Middle SDI	Male	Stomach cancer	2015		22.91	20.37	25.59
Middle SDI	Female	Stomach cancer	2015		11.39	10.21	12.53
Middle SDI	Both	Stomach cancer	2015		16.88	15.46	18.35
High SDI	Male	Stomach cancer	2016		18.44	17.16	19.17
High SDI	Female	Stomach cancer	2016		8.07	7.18	8.56
High SDI	Both	Stomach cancer	2016		12.75	11.69	13.33
High-middle SDI	Male	Stomach cancer	2016		29.58	26.15	33.10
High-middle SDI	Female	Stomach cancer	2016		10.63	9.65	11.75
High-middle SDI	Both	Stomach cancer	2016		19.11	17.40	20.81
Low SDI	Male	Stomach cancer	2016		9.39	8.24	10.47
Low SDI	Female	Stomach cancer	2016		7.10	6.33	8.03
Low SDI	Both	Stomach cancer	2016		8.20	7.38	9.04
Low-middle SDI	Male	Stomach cancer	2016		13.17	12.14	14.22
Low-middle SDI	Female	Stomach cancer	2016		9.13	8.27	10.04
Low-middle SDI	Both	Stomach cancer	2016		11.06	10.31	11.79
Middle SDI	Male	Stomach cancer	2016		22.47	19.90	25.43
Middle SDI	Female	Stomach cancer	2016		11.17	9.96	12.39
Middle SDI	Both	Stomach cancer	2016		16.54	14.98	18.09
High SDI	Male	Stomach cancer	2017		18.01	16.77	18.91
High SDI	Female	Stomach cancer	2017		7.91	7.03	8.49
High SDI	Both	Stomach cancer	2017		12.48	11.46	13.19
High-middle SDI	Male	Stomach cancer	2017		29.16	25.43	32.95
High-middle SDI	Female	Stomach cancer	2017		10.48	9.34	11.80
High-middle SDI	Both	Stomach cancer	2017		18.86	16.95	20.78
Low SDI	Male	Stomach cancer	2017		9.29	8.19	10.42
Low SDI	Female	Stomach cancer	2017		7.05	6.28	7.98
Low SDI	Both	Stomach cancer	2017		8.13	7.33	8.97

Low-middle SDI	Male	Stomach cancer	2017		13.11	12.01	14.24
Low-middle SDI	Female	Stomach cancer	2017		9.04	8.10	10.04
Low-middle SDI	Both	Stomach cancer	2017		10.98	10.17	11.81
Middle SDI	Male	Stomach cancer	2017		22.26	19.30	25.39
Middle SDI	Female	Stomach cancer	2017		11.06	9.68	12.50
Middle SDI	Both	Stomach cancer	2017		16.38	14.71	18.10
High SDI	Male	Stomach cancer	2018		17.90	16.23	19.64
High SDI	Female	Stomach cancer	2018		7.92	6.93	8.71
High SDI	Both	Stomach cancer	2018		12.45	11.31	13.50
High-middle SDI	Male	Stomach cancer	2018		28.93	25.02	33.02
High-middle SDI	Female	Stomach cancer	2018		10.48	9.21	11.83
High-middle SDI	Both	Stomach cancer	2018		18.77	16.83	20.72
Low SDI	Male	Stomach cancer	2018		9.23	8.18	10.33
Low SDI	Female	Stomach cancer	2018		7.03	6.26	8.00
Low SDI	Both	Stomach cancer	2018		8.09	7.32	8.94
Low-middle SDI	Male	Stomach cancer	2018		13.11	11.89	14.42
Low-middle SDI	Female	Stomach cancer	2018		8.99	7.96	10.02
Low-middle SDI	Both	Stomach cancer	2018		10.95	10.15	11.88
Middle SDI	Male	Stomach cancer	2018		22.11	18.93	25.47
Middle SDI	Female	Stomach cancer	2018		11.03	9.49	12.58
Middle SDI	Both	Stomach cancer	2018		16.28	14.47	18.23
High SDI	Male	Stomach cancer	2019		17.78	15.90	19.81
High SDI	Female	Stomach cancer	2019		7.91	6.83	8.83
High SDI	Both	Stomach cancer	2019		12.40	11.06	13.58
High-middle SDI	Male	Stomach cancer	2019		28.89	24.80	33.66
High-middle SDI	Female	Stomach cancer	2019		10.45	9.16	12.02
High-middle SDI	Both	Stomach cancer	2019		18.76	16.62	21.02
Low SDI	Male	Stomach cancer	2019		9.13	8.09	10.21
Low SDI	Female	Stomach cancer	2019		6.97	6.19	7.92
Low SDI	Both	Stomach cancer	2019		8.01	7.23	8.84
Low-middle SDI	Male	Stomach cancer	2019		13.09	11.76	14.48
Low-middle SDI	Female	Stomach cancer	2019		8.94	7.91	10.07
Low-middle SDI	Both	Stomach cancer	2019		10.92	9.97	11.94
Middle SDI	Male	Stomach cancer	2019		22.03	18.61	25.84
Middle SDI	Female	Stomach cancer	2019		10.98	9.41	12.64
Middle SDI	Both	Stomach cancer	2019		16.22	14.33	18.42

sex_name	cause_name	year	Age-standardised death rate (per 100 000 person-years)	95% CI (lower)	95% CI (upper)
Male	Stomach cancer	1990	27.62	25.53	29.78
Female	Stomach cancer	1990	14.68	13.59	15.72
Both	Stomach cancer	1990	20.48	19.25	21.62
Male	Stomach cancer	1991	27.13	25.25	29.06
Female	Stomach cancer	1991	14.35	13.30	15.46
Both	Stomach cancer	1991	20.08	18.92	21.20
Male	Stomach cancer	1992	26.72	24.96	28.49
Female	Stomach cancer	1992	14.10	13.14	15.10
Both	Stomach cancer	1992	19.76	18.67	20.80
Male	Stomach cancer	1993	26.50	24.96	28.17
Female	Stomach cancer	1993	13.99	13.07	14.94
Both	Stomach cancer	1993	19.61	18.64	20.57
Male	Stomach cancer	1994	26.00	24.54	27.58
Female	Stomach cancer	1994	13.70	12.86	14.56
Both	Stomach cancer	1994	19.24	18.28	20.21
Male	Stomach cancer	1995	25.48	24.08	26.91
Female	Stomach cancer	1995	13.29	12.40	14.25
Both	Stomach cancer	1995	18.78	17.84	19.71
Male	Stomach cancer	1996	24.83	23.55	26.19
Female	Stomach cancer	1996	12.93	12.11	13.77
Both	Stomach cancer	1996	18.29	17.43	19.15
Male	Stomach cancer	1997	24.27	22.99	25.57
Female	Stomach cancer	1997	12.62	11.84	13.40
Both	Stomach cancer	1997	17.86	16.96	18.67
Male	Stomach cancer	1998	24.01	22.71	25.35
Female	Stomach cancer	1998	12.35	11.56	13.09
Both	Stomach cancer	1998	17.60	16.74	18.41
Male	Stomach cancer	1999	23.92	22.65	25.23
Female	Stomach cancer	1999	12.29	11.45	13.06
Both	Stomach cancer	1999	17.53	16.68	18.33
Male	Stomach cancer	2000	24.03	22.85	25.37
Female	Stomach cancer	2000	12.20	11.43	12.97
Both	Stomach cancer	2000	17.55	16.69	18.42
Male	Stomach cancer	2001	24.06	22.65	25.56
Female	Stomach cancer	2001	12.09	11.27	12.82
Both	Stomach cancer	2001	17.51	16.54	18.36
Male	Stomach cancer	2002	24.26	22.88	25.65
Female	Stomach cancer	2002	12.11	11.28	12.85
Both	Stomach cancer	2002	17.62	16.64	18.51
Male	Stomach cancer	2003	24.42	22.99	25.89
Female	Stomach cancer	2003	12.07	11.18	12.81
Both	Stomach cancer	2003	17.68	16.80	18.56
Male	Stomach cancer	2004	24.46	23.00	25.98
Female	Stomach cancer	2004	11.86	11.04	12.59
Both	Stomach cancer	2004	17.60	16.67	18.49
Male	Stomach cancer	2005	24.08	22.75	25.54
Female	Stomach cancer	2005	11.56	10.71	12.25
Both	Stomach cancer	2005	17.27	16.33	18.17
Male	Stomach cancer	2006	22.98	21.56	24.32

Female	Stomach cancer	2006	11.05	10.26	11.65
Both	Stomach cancer	2006	16.49	15.54	17.30
Male	Stomach cancer	2007	22.25	20.85	23.52
Female	Stomach cancer	2007	10.63	9.84	11.21
Both	Stomach cancer	2007	15.93	15.00	16.71
Male	Stomach cancer	2008	21.77	20.36	23.13
Female	Stomach cancer	2008	10.29	9.50	10.86
Both	Stomach cancer	2008	15.53	14.57	16.29
Male	Stomach cancer	2009	21.25	19.93	22.51
Female	Stomach cancer	2009	9.95	9.16	10.56
Both	Stomach cancer	2009	15.10	14.16	15.85
Male	Stomach cancer	2010	20.79	19.45	22.08
Female	Stomach cancer	2010	9.66	8.92	10.25
Both	Stomach cancer	2010	14.74	13.83	15.51
Male	Stomach cancer	2011	20.14	18.72	21.53
Female	Stomach cancer	2011	9.35	8.51	9.94
Both	Stomach cancer	2011	14.27	13.31	15.11
Male	Stomach cancer	2012	19.56	18.28	20.95
Female	Stomach cancer	2012	9.01	8.25	9.55
Both	Stomach cancer	2012	13.83	13.00	14.62
Male	Stomach cancer	2013	18.88	17.50	20.23
Female	Stomach cancer	2013	8.73	7.97	9.26
Both	Stomach cancer	2013	13.36	12.44	14.12
Male	Stomach cancer	2014	18.26	16.87	19.67
Female	Stomach cancer	2014	8.51	7.83	9.08
Both	Stomach cancer	2014	12.96	12.08	13.72
Male	Stomach cancer	2015	17.82	16.40	19.17
Female	Stomach cancer	2015	8.33	7.61	8.88
Both	Stomach cancer	2015	12.66	11.80	13.42
Male	Stomach cancer	2016	17.49	15.95	19.00
Female	Stomach cancer	2016	8.19	7.45	8.79
Both	Stomach cancer	2016	12.43	11.41	13.33
Male	Stomach cancer	2017	17.05	15.47	18.58
Female	Stomach cancer	2017	8.04	7.24	8.74
Both	Stomach cancer	2017	12.15	11.20	12.98
Male	Stomach cancer	2018	16.75	15.03	18.41
Female	Stomach cancer	2018	7.97	7.20	8.74
Both	Stomach cancer	2018	11.98	10.97	12.98
Male	Stomach cancer	2019	16.59	14.80	18.34
Female	Stomach cancer	2019	7.92	7.07	8.76
Both	Stomach cancer	2019	11.88	10.82	12.82

location_name	sex_name	cause_name	year	Age-standardised death rate (per 100 000 person-years)	95% CI (lower)	95% CI (upper)
High SDI	Male	Stomach cancer	1990	21.55	20.80	21.94
High SDI	Female	Stomach cancer	1990	10.06	9.47	10.36
High SDI	Both	Stomach cancer	1990	14.83	14.21	15.14
High-middle SDI	Male	Stomach cancer	1990	38.03	35.11	41.01
High-middle SDI	Female	Stomach cancer	1990	17.81	16.54	19.09
High-middle SDI	Both	Stomach cancer	1990	26.38	24.76	27.91
Low SDI	Male	Stomach cancer	1990	14.75	12.95	16.50
Low SDI	Female	Stomach cancer	1990	9.43	7.72	10.95
Low SDI	Both	Stomach cancer	1990	12.09	10.78	13.28
Low-middle SDI	Male	Stomach cancer	1990	17.19	15.54	18.58
Low-middle SDI	Female	Stomach cancer	1990	12.45	10.87	13.98
Low-middle SDI	Both	Stomach cancer	1990	14.81	13.54	15.91
Middle SDI	Male	Stomach cancer	1990	32.10	27.89	36.25
Middle SDI	Female	Stomach cancer	1990	18.28	16.15	20.36
Middle SDI	Both	Stomach cancer	1990	24.85	22.60	27.23
High SDI	Male	Stomach cancer	1991	21.08	20.35	21.46
High SDI	Female	Stomach cancer	1991	9.77	9.17	10.06
High SDI	Both	Stomach cancer	1991	14.47	13.84	14.79
High-middle SDI	Male	Stomach cancer	1991	37.12	34.45	39.96
High-middle SDI	Female	Stomach cancer	1991	17.33	16.13	18.62
High-middle SDI	Both	Stomach cancer	1991	25.74	24.27	27.22
Low SDI	Male	Stomach cancer	1991	14.62	12.83	16.27
Low SDI	Female	Stomach cancer	1991	9.33	7.65	10.85
Low SDI	Both	Stomach cancer	1991	11.97	10.59	13.20
Low-middle SDI	Male	Stomach cancer	1991	17.00	15.48	18.37
Low-middle SDI	Female	Stomach cancer	1991	12.24	10.64	13.78
Low-middle SDI	Both	Stomach cancer	1991	14.61	13.50	15.69
Middle SDI	Male	Stomach cancer	1991	31.70	27.91	35.67
Middle SDI	Female	Stomach cancer	1991	17.98	16.06	20.18
Middle SDI	Both	Stomach cancer	1991	24.51	22.42	26.85
High SDI	Male	Stomach cancer	1992	20.62	19.89	21.01
High SDI	Female	Stomach cancer	1992	9.48	8.87	9.78
High SDI	Both	Stomach cancer	1992	14.12	13.49	14.45
High-middle SDI	Male	Stomach cancer	1992	36.65	34.22	39.24
High-middle SDI	Female	Stomach cancer	1992	16.91	15.82	18.10
High-middle SDI	Both	Stomach cancer	1992	25.32	23.97	26.65
Low SDI	Male	Stomach cancer	1992	14.48	12.71	16.12
Low SDI	Female	Stomach cancer	1992	9.29	7.67	10.82
Low SDI	Both	Stomach cancer	1992	11.88	10.52	13.09
Low-middle SDI	Male	Stomach cancer	1992	16.81	15.19	18.14
Low-middle SDI	Female	Stomach cancer	1992	12.20	10.63	13.78
Low-middle SDI	Both	Stomach cancer	1992	14.49	13.34	15.57
Middle SDI	Male	Stomach cancer	1992	31.18	27.59	34.99
Middle SDI	Female	Stomach cancer	1992	17.74	15.96	19.73
Middle SDI	Both	Stomach cancer	1992	24.15	22.11	26.31
High SDI	Male	Stomach cancer	1993	20.26	19.51	20.65
High SDI	Female	Stomach cancer	1993	9.27	8.65	9.58
High SDI	Both	Stomach cancer	1993	13.86	13.22	14.18
High-middle SDI	Male	Stomach cancer	1993	36.65	34.39	39.13

High-middle SDI	Female	Stomach cancer	1993	17.01	15.99	18.04
High-middle SDI	Both	Stomach cancer	1993	25.42	24.23	26.62
Low SDI	Male	Stomach cancer	1993	14.39	12.66	16.00
Low SDI	Female	Stomach cancer	1993	9.20	7.66	10.71
Low SDI	Both	Stomach cancer	1993	11.79	10.46	12.96
Low-middle SDI	Male	Stomach cancer	1993	16.68	15.17	17.89
Low-middle SDI	Female	Stomach cancer	1993	12.01	10.56	13.38
Low-middle SDI	Both	Stomach cancer	1993	14.33	13.26	15.34
Middle SDI	Male	Stomach cancer	1993	30.77	27.72	34.36
Middle SDI	Female	Stomach cancer	1993	17.54	15.79	19.49
Middle SDI	Both	Stomach cancer	1993	23.85	21.96	25.97
High SDI	Male	Stomach cancer	1994	19.78	19.03	20.16
High SDI	Female	Stomach cancer	1994	8.98	8.36	9.30
High SDI	Both	Stomach cancer	1994	13.51	12.85	13.82
High-middle SDI	Male	Stomach cancer	1994	36.05	34.03	38.11
High-middle SDI	Female	Stomach cancer	1994	16.72	15.74	17.71
High-middle SDI	Both	Stomach cancer	1994	25.02	23.84	26.13
Low SDI	Male	Stomach cancer	1994	14.27	12.53	15.80
Low SDI	Female	Stomach cancer	1994	9.11	7.58	10.55
Low SDI	Both	Stomach cancer	1994	11.68	10.41	12.80
Low-middle SDI	Male	Stomach cancer	1994	16.50	15.03	17.70
Low-middle SDI	Female	Stomach cancer	1994	11.83	10.49	13.18
Low-middle SDI	Both	Stomach cancer	1994	14.14	13.07	15.11
Middle SDI	Male	Stomach cancer	1994	30.04	27.18	33.46
Middle SDI	Female	Stomach cancer	1994	17.14	15.55	19.01
Middle SDI	Both	Stomach cancer	1994	23.30	21.58	25.30
High SDI	Male	Stomach cancer	1995	19.40	18.65	19.78
High SDI	Female	Stomach cancer	1995	8.79	8.18	9.10
High SDI	Both	Stomach cancer	1995	13.24	12.59	13.56
High-middle SDI	Male	Stomach cancer	1995	34.87	32.92	36.95
High-middle SDI	Female	Stomach cancer	1995	15.97	14.95	16.91
High-middle SDI	Both	Stomach cancer	1995	24.08	23.00	25.22
Low SDI	Male	Stomach cancer	1995	14.16	12.51	15.66
Low SDI	Female	Stomach cancer	1995	9.01	7.58	10.40
Low SDI	Both	Stomach cancer	1995	11.57	10.33	12.68
Low-middle SDI	Male	Stomach cancer	1995	16.24	14.92	17.33
Low-middle SDI	Female	Stomach cancer	1995	11.59	10.20	12.92
Low-middle SDI	Both	Stomach cancer	1995	13.89	12.88	14.86
Middle SDI	Male	Stomach cancer	1995	29.75	27.21	32.67
Middle SDI	Female	Stomach cancer	1995	16.66	15.16	18.57
Middle SDI	Both	Stomach cancer	1995	22.91	21.30	24.88
High SDI	Male	Stomach cancer	1996	18.75	18.00	19.14
High SDI	Female	Stomach cancer	1996	8.51	7.89	8.81
High SDI	Both	Stomach cancer	1996	12.81	12.16	13.13
High-middle SDI	Male	Stomach cancer	1996	33.37	31.60	35.23
High-middle SDI	Female	Stomach cancer	1996	15.25	14.34	16.17
High-middle SDI	Both	Stomach cancer	1996	23.01	21.94	24.11
Low SDI	Male	Stomach cancer	1996	14.04	12.39	15.55
Low SDI	Female	Stomach cancer	1996	8.93	7.53	10.30
Low SDI	Both	Stomach cancer	1996	11.46	10.22	12.56
Low-middle SDI	Male	Stomach cancer	1996	16.12	14.84	17.21

Low-middle SDI	Female	Stomach cancer	1996	11.51	10.17	12.75
Low-middle SDI	Both	Stomach cancer	1996	13.78	12.81	14.72
Middle SDI	Male	Stomach cancer	1996	29.44	27.07	32.29
Middle SDI	Female	Stomach cancer	1996	16.35	14.85	18.07
Middle SDI	Both	Stomach cancer	1996	22.60	21.06	24.39
High SDI	Male	Stomach cancer	1997	18.11	17.33	18.52
High SDI	Female	Stomach cancer	1997	8.25	7.64	8.55
High SDI	Both	Stomach cancer	1997	12.39	11.73	12.72
High-middle SDI	Male	Stomach cancer	1997	32.17	30.40	33.98
High-middle SDI	Female	Stomach cancer	1997	14.67	13.82	15.44
High-middle SDI	Both	Stomach cancer	1997	22.16	21.11	23.17
Low SDI	Male	Stomach cancer	1997	13.98	12.33	15.49
Low SDI	Female	Stomach cancer	1997	8.87	7.52	10.20
Low SDI	Both	Stomach cancer	1997	11.40	10.22	12.48
Low-middle SDI	Male	Stomach cancer	1997	16.30	15.06	17.36
Low-middle SDI	Female	Stomach cancer	1997	11.59	10.27	12.78
Low-middle SDI	Both	Stomach cancer	1997	13.91	12.96	14.78
Middle SDI	Male	Stomach cancer	1997	28.96	26.53	31.37
Middle SDI	Female	Stomach cancer	1997	15.98	14.68	17.53
Middle SDI	Both	Stomach cancer	1997	22.16	20.81	23.84
High SDI	Male	Stomach cancer	1998	17.78	17.03	18.19
High SDI	Female	Stomach cancer	1998	8.06	7.46	8.38
High SDI	Both	Stomach cancer	1998	12.16	11.50	12.48
High-middle SDI	Male	Stomach cancer	1998	31.51	29.72	33.38
High-middle SDI	Female	Stomach cancer	1998	14.18	13.40	14.95
High-middle SDI	Both	Stomach cancer	1998	21.60	20.51	22.58
Low SDI	Male	Stomach cancer	1998	13.84	12.18	15.21
Low SDI	Female	Stomach cancer	1998	8.83	7.52	10.14
Low SDI	Both	Stomach cancer	1998	11.30	10.08	12.33
Low-middle SDI	Male	Stomach cancer	1998	16.28	15.09	17.38
Low-middle SDI	Female	Stomach cancer	1998	11.53	10.19	12.68
Low-middle SDI	Both	Stomach cancer	1998	13.86	12.92	14.74
Middle SDI	Male	Stomach cancer	1998	28.92	26.72	31.56
Middle SDI	Female	Stomach cancer	1998	15.65	14.36	17.25
Middle SDI	Both	Stomach cancer	1998	21.96	20.60	23.66
High SDI	Male	Stomach cancer	1999	17.37	16.65	17.78
High SDI	Female	Stomach cancer	1999	7.89	7.26	8.20
High SDI	Both	Stomach cancer	1999	11.89	11.23	12.22
High-middle SDI	Male	Stomach cancer	1999	31.64	29.90	33.44
High-middle SDI	Female	Stomach cancer	1999	14.16	13.35	14.97
High-middle SDI	Both	Stomach cancer	1999	21.66	20.69	22.66
Low SDI	Male	Stomach cancer	1999	13.60	11.95	15.08
Low SDI	Female	Stomach cancer	1999	8.72	7.46	10.00
Low SDI	Both	Stomach cancer	1999	11.13	9.98	12.11
Low-middle SDI	Male	Stomach cancer	1999	16.01	14.74	17.11
Low-middle SDI	Female	Stomach cancer	1999	11.40	10.18	12.52
Low-middle SDI	Both	Stomach cancer	1999	13.66	12.71	14.53
Middle SDI	Male	Stomach cancer	1999	28.97	26.58	31.58
Middle SDI	Female	Stomach cancer	1999	15.68	14.36	17.28
Middle SDI	Both	Stomach cancer	1999	22.01	20.54	23.61
High SDI	Male	Stomach cancer	2000	16.73	15.97	17.12

High SDI	Female	Stomach cancer	2000	7.63	7.01	7.93
High SDI	Both	Stomach cancer	2000	11.48	10.81	11.81
High-middle SDI	Male	Stomach cancer	2000	31.99	30.25	34.01
High-middle SDI	Female	Stomach cancer	2000	14.06	13.19	14.85
High-middle SDI	Both	Stomach cancer	2000	21.78	20.80	22.85
Low SDI	Male	Stomach cancer	2000	13.53	11.88	14.90
Low SDI	Female	Stomach cancer	2000	8.65	7.42	9.90
Low SDI	Both	Stomach cancer	2000	11.05	9.86	12.00
Low-middle SDI	Male	Stomach cancer	2000	16.14	14.99	17.16
Low-middle SDI	Female	Stomach cancer	2000	11.32	10.15	12.48
Low-middle SDI	Both	Stomach cancer	2000	13.68	12.79	14.53
Middle SDI	Male	Stomach cancer	2000	29.54	27.15	32.05
Middle SDI	Female	Stomach cancer	2000	15.81	14.46	17.53
Middle SDI	Both	Stomach cancer	2000	22.36	20.90	24.07
High SDI	Male	Stomach cancer	2001	16.18	15.44	16.60
High SDI	Female	Stomach cancer	2001	7.38	6.75	7.69
High SDI	Both	Stomach cancer	2001	11.12	10.46	11.45
High-middle SDI	Male	Stomach cancer	2001	32.07	30.03	34.24
High-middle SDI	Female	Stomach cancer	2001	13.90	13.05	14.73
High-middle SDI	Both	Stomach cancer	2001	21.74	20.61	22.84
Low SDI	Male	Stomach cancer	2001	13.41	11.77	14.73
Low SDI	Female	Stomach cancer	2001	8.66	7.44	9.87
Low SDI	Both	Stomach cancer	2001	10.99	9.82	11.93
Low-middle SDI	Male	Stomach cancer	2001	16.21	15.13	17.30
Low-middle SDI	Female	Stomach cancer	2001	11.31	10.13	12.35
Low-middle SDI	Both	Stomach cancer	2001	13.70	12.86	14.54
Middle SDI	Male	Stomach cancer	2001	30.02	27.40	32.89
Middle SDI	Female	Stomach cancer	2001	15.80	14.54	17.31
Middle SDI	Both	Stomach cancer	2001	22.58	21.04	24.26
High SDI	Male	Stomach cancer	2002	15.75	15.02	16.16
High SDI	Female	Stomach cancer	2002	7.17	6.55	7.48
High SDI	Both	Stomach cancer	2002	10.82	10.16	11.16
High-middle SDI	Male	Stomach cancer	2002	32.48	30.47	34.62
High-middle SDI	Female	Stomach cancer	2002	13.94	13.02	14.75
High-middle SDI	Both	Stomach cancer	2002	21.95	20.78	23.07
Low SDI	Male	Stomach cancer	2002	13.29	11.81	14.64
Low SDI	Female	Stomach cancer	2002	8.66	7.48	9.80
Low SDI	Both	Stomach cancer	2002	10.93	9.82	11.86
Low-middle SDI	Male	Stomach cancer	2002	16.25	15.21	17.25
Low-middle SDI	Female	Stomach cancer	2002	11.38	10.15	12.49
Low-middle SDI	Both	Stomach cancer	2002	13.76	12.87	14.63
Middle SDI	Male	Stomach cancer	2002	30.71	28.05	33.50
Middle SDI	Female	Stomach cancer	2002	16.01	14.64	17.51
Middle SDI	Both	Stomach cancer	2002	23.03	21.49	24.79
High SDI	Male	Stomach cancer	2003	15.42	14.67	15.82
High SDI	Female	Stomach cancer	2003	6.99	6.35	7.30
High SDI	Both	Stomach cancer	2003	10.59	9.91	10.92
High-middle SDI	Male	Stomach cancer	2003	32.78	30.69	34.92
High-middle SDI	Female	Stomach cancer	2003	13.95	13.01	14.74
High-middle SDI	Both	Stomach cancer	2003	22.10	20.94	23.20
Low SDI	Male	Stomach cancer	2003	13.23	11.64	14.54

Low SDI	Female	Stomach cancer	2003	8.59	7.47	9.71
Low SDI	Both	Stomach cancer	2003	10.86	9.79	11.81
Low-middle SDI	Male	Stomach cancer	2003	16.24	15.13	17.25
Low-middle SDI	Female	Stomach cancer	2003	11.16	10.05	12.18
Low-middle SDI	Both	Stomach cancer	2003	13.63	12.79	14.50
Middle SDI	Male	Stomach cancer	2003	31.28	28.67	33.97
Middle SDI	Female	Stomach cancer	2003	16.17	14.81	17.65
Middle SDI	Both	Stomach cancer	2003	23.39	21.86	25.05
High SDI	Male	Stomach cancer	2004	14.91	14.14	15.31
High SDI	Female	Stomach cancer	2004	6.75	6.12	7.07
High SDI	Both	Stomach cancer	2004	10.25	9.56	10.58
High-middle SDI	Male	Stomach cancer	2004	32.90	30.56	35.14
High-middle SDI	Female	Stomach cancer	2004	13.70	12.77	14.47
High-middle SDI	Both	Stomach cancer	2004	22.03	20.77	23.13
Low SDI	Male	Stomach cancer	2004	13.01	11.50	14.30
Low SDI	Female	Stomach cancer	2004	8.50	7.42	9.60
Low SDI	Both	Stomach cancer	2004	10.70	9.69	11.61
Low-middle SDI	Male	Stomach cancer	2004	15.99	14.93	16.93
Low-middle SDI	Female	Stomach cancer	2004	10.89	9.90	11.92
Low-middle SDI	Both	Stomach cancer	2004	13.37	12.56	14.11
Middle SDI	Male	Stomach cancer	2004	31.94	29.25	35.03
Middle SDI	Female	Stomach cancer	2004	16.06	14.80	17.49
Middle SDI	Both	Stomach cancer	2004	23.65	22.10	25.43
High SDI	Male	Stomach cancer	2005	14.60	13.83	15.02
High SDI	Female	Stomach cancer	2005	6.57	5.94	6.89
High SDI	Both	Stomach cancer	2005	10.02	9.34	10.36
High-middle SDI	Male	Stomach cancer	2005	32.38	30.32	34.63
High-middle SDI	Female	Stomach cancer	2005	13.33	12.34	14.14
High-middle SDI	Both	Stomach cancer	2005	21.60	20.40	22.77
Low SDI	Male	Stomach cancer	2005	12.85	11.40	14.14
Low SDI	Female	Stomach cancer	2005	8.45	7.38	9.55
Low SDI	Both	Stomach cancer	2005	10.58	9.56	11.49
Low-middle SDI	Male	Stomach cancer	2005	15.90	14.85	16.96
Low-middle SDI	Female	Stomach cancer	2005	10.86	9.92	11.88
Low-middle SDI	Both	Stomach cancer	2005	13.31	12.49	14.16
Middle SDI	Male	Stomach cancer	2005	31.41	28.90	34.29
Middle SDI	Female	Stomach cancer	2005	15.50	14.25	16.80
Middle SDI	Both	Stomach cancer	2005	23.09	21.59	24.80
High SDI	Male	Stomach cancer	2006	14.11	13.35	14.52
High SDI	Female	Stomach cancer	2006	6.34	5.71	6.66
High SDI	Both	Stomach cancer	2006	9.69	9.02	10.03
High-middle SDI	Male	Stomach cancer	2006	30.34	28.35	32.40
High-middle SDI	Female	Stomach cancer	2006	12.56	11.68	13.28
High-middle SDI	Both	Stomach cancer	2006	20.28	19.13	21.34
Low SDI	Male	Stomach cancer	2006	12.70	11.25	13.94
Low SDI	Female	Stomach cancer	2006	8.36	7.36	9.40
Low SDI	Both	Stomach cancer	2006	10.46	9.49	11.40
Low-middle SDI	Male	Stomach cancer	2006	15.70	14.71	16.64
Low-middle SDI	Female	Stomach cancer	2006	10.67	9.68	11.62
Low-middle SDI	Both	Stomach cancer	2006	13.10	12.34	13.89
Middle SDI	Male	Stomach cancer	2006	29.84	27.48	32.47

Middle SDI	Female	Stomach cancer	2006	14.66	13.48	15.82
Middle SDI	Both	Stomach cancer	2006	21.91	20.47	23.37
High SDI	Male	Stomach cancer	2007	13.74	12.95	14.15
High SDI	Female	Stomach cancer	2007	6.14	5.52	6.47
High SDI	Both	Stomach cancer	2007	9.43	8.74	9.77
High-middle SDI	Male	Stomach cancer	2007	29.00	27.04	30.87
High-middle SDI	Female	Stomach cancer	2007	12.01	11.11	12.75
High-middle SDI	Both	Stomach cancer	2007	19.39	18.21	20.39
Low SDI	Male	Stomach cancer	2007	12.66	11.29	13.91
Low SDI	Female	Stomach cancer	2007	8.21	7.24	9.26
Low SDI	Both	Stomach cancer	2007	10.37	9.46	11.32
Low-middle SDI	Male	Stomach cancer	2007	15.54	14.49	16.50
Low-middle SDI	Female	Stomach cancer	2007	10.48	9.54	11.38
Low-middle SDI	Both	Stomach cancer	2007	12.93	12.14	13.68
Middle SDI	Male	Stomach cancer	2007	28.80	26.37	31.20
Middle SDI	Female	Stomach cancer	2007	13.93	12.80	14.95
Middle SDI	Both	Stomach cancer	2007	21.02	19.62	22.42
High SDI	Male	Stomach cancer	2008	13.39	12.59	13.82
High SDI	Female	Stomach cancer	2008	5.96	5.32	6.29
High SDI	Both	Stomach cancer	2008	9.18	8.48	9.54
High-middle SDI	Male	Stomach cancer	2008	28.16	26.25	29.90
High-middle SDI	Female	Stomach cancer	2008	11.55	10.66	12.31
High-middle SDI	Both	Stomach cancer	2008	18.77	17.63	19.76
Low SDI	Male	Stomach cancer	2008	12.48	11.08	13.78
Low SDI	Female	Stomach cancer	2008	8.14	7.21	9.21
Low SDI	Both	Stomach cancer	2008	10.24	9.30	11.17
Low-middle SDI	Male	Stomach cancer	2008	15.44	14.44	16.47
Low-middle SDI	Female	Stomach cancer	2008	10.27	9.37	11.17
Low-middle SDI	Both	Stomach cancer	2008	12.77	12.01	13.55
Middle SDI	Male	Stomach cancer	2008	28.26	25.90	30.81
Middle SDI	Female	Stomach cancer	2008	13.38	12.32	14.35
Middle SDI	Both	Stomach cancer	2008	20.47	19.06	21.85
High SDI	Male	Stomach cancer	2009	13.02	12.21	13.43
High SDI	Female	Stomach cancer	2009	5.75	5.12	6.09
High SDI	Both	Stomach cancer	2009	8.91	8.22	9.27
High-middle SDI	Male	Stomach cancer	2009	27.20	25.36	29.08
High-middle SDI	Female	Stomach cancer	2009	11.07	10.21	11.86
High-middle SDI	Both	Stomach cancer	2009	18.07	17.01	19.13
Low SDI	Male	Stomach cancer	2009	12.33	10.91	13.59
Low SDI	Female	Stomach cancer	2009	8.03	7.09	9.11
Low SDI	Both	Stomach cancer	2009	10.10	9.16	11.03
Low-middle SDI	Male	Stomach cancer	2009	15.07	14.06	16.02
Low-middle SDI	Female	Stomach cancer	2009	9.93	9.11	10.74
Low-middle SDI	Both	Stomach cancer	2009	12.41	11.67	13.08
Middle SDI	Male	Stomach cancer	2009	27.83	25.38	30.30
Middle SDI	Female	Stomach cancer	2009	12.94	11.95	13.97
Middle SDI	Both	Stomach cancer	2009	20.01	18.65	21.31
High SDI	Male	Stomach cancer	2010	12.67	11.86	13.08
High SDI	Female	Stomach cancer	2010	5.58	4.96	5.93
High SDI	Both	Stomach cancer	2010	8.68	7.98	9.05
High-middle SDI	Male	Stomach cancer	2010	26.60	24.69	28.53

High-middle SDI	Female	Stomach cancer	2010	10.68	9.92	11.44
High-middle SDI	Both	Stomach cancer	2010	17.59	16.43	18.66
Low SDI	Male	Stomach cancer	2010	12.11	10.71	13.35
Low SDI	Female	Stomach cancer	2010	7.91	7.00	8.90
Low SDI	Both	Stomach cancer	2010	9.93	8.99	10.82
Low-middle SDI	Male	Stomach cancer	2010	14.74	13.83	15.65
Low-middle SDI	Female	Stomach cancer	2010	9.76	8.90	10.59
Low-middle SDI	Both	Stomach cancer	2010	12.16	11.45	12.86
Middle SDI	Male	Stomach cancer	2010	27.26	24.93	29.65
Middle SDI	Female	Stomach cancer	2010	12.48	11.43	13.41
Middle SDI	Both	Stomach cancer	2010	19.49	18.08	20.82
High SDI	Male	Stomach cancer	2011	12.33	11.51	12.75
High SDI	Female	Stomach cancer	2011	5.46	4.83	5.80
High SDI	Both	Stomach cancer	2011	8.47	7.77	8.83
High-middle SDI	Male	Stomach cancer	2011	25.55	23.63	27.57
High-middle SDI	Female	Stomach cancer	2011	10.17	9.29	10.96
High-middle SDI	Both	Stomach cancer	2011	16.86	15.67	17.93
Low SDI	Male	Stomach cancer	2011	11.95	10.57	13.20
Low SDI	Female	Stomach cancer	2011	7.81	6.94	8.80
Low SDI	Both	Stomach cancer	2011	9.79	8.88	10.72
Low-middle SDI	Male	Stomach cancer	2011	14.33	13.36	15.22
Low-middle SDI	Female	Stomach cancer	2011	9.63	8.76	10.46
Low-middle SDI	Both	Stomach cancer	2011	11.90	11.16	12.58
Middle SDI	Male	Stomach cancer	2011	26.34	23.98	28.80
Middle SDI	Female	Stomach cancer	2011	11.96	10.78	12.90
Middle SDI	Both	Stomach cancer	2011	18.77	17.29	20.12
High SDI	Male	Stomach cancer	2012	11.93	11.14	12.36
High SDI	Female	Stomach cancer	2012	5.30	4.67	5.63
High SDI	Both	Stomach cancer	2012	8.20	7.52	8.57
High-middle SDI	Male	Stomach cancer	2012	24.77	22.89	26.77
High-middle SDI	Female	Stomach cancer	2012	9.67	8.77	10.44
High-middle SDI	Both	Stomach cancer	2012	16.24	15.21	17.29
Low SDI	Male	Stomach cancer	2012	11.79	10.46	13.01
Low SDI	Female	Stomach cancer	2012	7.84	6.95	8.81
Low SDI	Both	Stomach cancer	2012	9.73	8.86	10.61
Low-middle SDI	Male	Stomach cancer	2012	14.01	13.11	14.87
Low-middle SDI	Female	Stomach cancer	2012	9.46	8.68	10.20
Low-middle SDI	Both	Stomach cancer	2012	11.65	10.99	12.31
Middle SDI	Male	Stomach cancer	2012	25.52	23.25	28.03
Middle SDI	Female	Stomach cancer	2012	11.36	10.39	12.25
Middle SDI	Both	Stomach cancer	2012	18.05	16.77	19.40
High SDI	Male	Stomach cancer	2013	11.53	10.72	11.95
High SDI	Female	Stomach cancer	2013	5.14	4.52	5.47
High SDI	Both	Stomach cancer	2013	7.95	7.24	8.30
High-middle SDI	Male	Stomach cancer	2013	23.79	21.83	25.79
High-middle SDI	Female	Stomach cancer	2013	9.25	8.45	9.94
High-middle SDI	Both	Stomach cancer	2013	15.58	14.43	16.61
Low SDI	Male	Stomach cancer	2013	11.68	10.37	12.93
Low SDI	Female	Stomach cancer	2013	7.75	6.86	8.68
Low SDI	Both	Stomach cancer	2013	9.63	8.73	10.57
Low-middle SDI	Male	Stomach cancer	2013	13.77	12.95	14.60

Low-middle SDI	Female	Stomach cancer	2013	9.42	8.58	10.20
Low-middle SDI	Both	Stomach cancer	2013	11.50	10.84	12.13
Middle SDI	Male	Stomach cancer	2013	24.48	22.18	26.87
Middle SDI	Female	Stomach cancer	2013	10.92	9.92	11.81
Middle SDI	Both	Stomach cancer	2013	17.32	16.00	18.62
High SDI	Male	Stomach cancer	2014	11.11	10.30	11.55
High SDI	Female	Stomach cancer	2014	4.99	4.38	5.32
High SDI	Both	Stomach cancer	2014	7.68	6.99	8.06
High-middle SDI	Male	Stomach cancer	2014	23.06	20.97	25.08
High-middle SDI	Female	Stomach cancer	2014	8.94	8.21	9.65
High-middle SDI	Both	Stomach cancer	2014	15.10	14.03	16.15
Low SDI	Male	Stomach cancer	2014	11.34	10.05	12.53
Low SDI	Female	Stomach cancer	2014	7.72	6.88	8.69
Low SDI	Both	Stomach cancer	2014	9.45	8.54	10.34
Low-middle SDI	Male	Stomach cancer	2014	13.53	12.66	14.37
Low-middle SDI	Female	Stomach cancer	2014	9.39	8.55	10.22
Low-middle SDI	Both	Stomach cancer	2014	11.36	10.68	11.97
Middle SDI	Male	Stomach cancer	2014	23.54	21.16	26.03
Middle SDI	Female	Stomach cancer	2014	10.52	9.58	11.39
Middle SDI	Both	Stomach cancer	2014	16.66	15.26	18.00
High SDI	Male	Stomach cancer	2015	10.82	10.02	11.28
High SDI	Female	Stomach cancer	2015	4.87	4.26	5.22
High SDI	Both	Stomach cancer	2015	7.50	6.80	7.87
High-middle SDI	Male	Stomach cancer	2015	22.64	20.53	24.84
High-middle SDI	Female	Stomach cancer	2015	8.73	7.93	9.46
High-middle SDI	Both	Stomach cancer	2015	14.81	13.69	15.85
Low SDI	Male	Stomach cancer	2015	11.19	9.88	12.40
Low SDI	Female	Stomach cancer	2015	7.63	6.78	8.59
Low SDI	Both	Stomach cancer	2015	9.33	8.44	10.20
Low-middle SDI	Male	Stomach cancer	2015	13.25	12.33	14.06
Low-middle SDI	Female	Stomach cancer	2015	9.41	8.53	10.22
Low-middle SDI	Both	Stomach cancer	2015	11.23	10.55	11.92
Middle SDI	Male	Stomach cancer	2015	22.75	20.34	25.42
Middle SDI	Female	Stomach cancer	2015	10.12	9.10	11.10
Middle SDI	Both	Stomach cancer	2015	16.06	14.73	17.51
High SDI	Male	Stomach cancer	2016	10.57	9.75	11.03
High SDI	Female	Stomach cancer	2016	4.81	4.19	5.15
High SDI	Both	Stomach cancer	2016	7.35	6.65	7.73
High-middle SDI	Male	Stomach cancer	2016	22.28	19.81	24.61
High-middle SDI	Female	Stomach cancer	2016	8.54	7.71	9.34
High-middle SDI	Both	Stomach cancer	2016	14.55	13.33	15.77
Low SDI	Male	Stomach cancer	2016	10.96	9.66	12.22
Low SDI	Female	Stomach cancer	2016	7.53	6.71	8.58
Low SDI	Both	Stomach cancer	2016	9.17	8.31	10.09
Low-middle SDI	Male	Stomach cancer	2016	13.06	12.07	14.02
Low-middle SDI	Female	Stomach cancer	2016	9.30	8.37	10.25
Low-middle SDI	Both	Stomach cancer	2016	11.09	10.34	11.84
Middle SDI	Male	Stomach cancer	2016	22.21	19.77	24.99
Middle SDI	Female	Stomach cancer	2016	9.90	8.87	10.99
Middle SDI	Both	Stomach cancer	2016	15.68	14.22	17.23
High SDI	Male	Stomach cancer	2017	10.35	9.54	10.82

High SDI	Female	Stomach cancer	2017	4.73	4.13	5.07
High SDI	Both	Stomach cancer	2017	7.22	6.54	7.60
High-middle SDI	Male	Stomach cancer	2017	21.65	19.21	24.14
High-middle SDI	Female	Stomach cancer	2017	8.35	7.49	9.23
High-middle SDI	Both	Stomach cancer	2017	14.18	12.91	15.44
Low SDI	Male	Stomach cancer	2017	10.83	9.54	12.07
Low SDI	Female	Stomach cancer	2017	7.48	6.70	8.45
Low SDI	Both	Stomach cancer	2017	9.08	8.23	9.99
Low-middle SDI	Male	Stomach cancer	2017	12.93	11.90	13.99
Low-middle SDI	Female	Stomach cancer	2017	9.18	8.22	10.22
Low-middle SDI	Both	Stomach cancer	2017	10.96	10.15	11.75
Middle SDI	Male	Stomach cancer	2017	21.48	18.96	24.18
Middle SDI	Female	Stomach cancer	2017	9.65	8.57	10.71
Middle SDI	Both	Stomach cancer	2017	15.20	13.72	16.68
High SDI	Male	Stomach cancer	2018	10.28	9.49	10.80
High SDI	Female	Stomach cancer	2018	4.74	4.09	5.09
High SDI	Both	Stomach cancer	2018	7.20	6.50	7.61
High-middle SDI	Male	Stomach cancer	2018	21.20	18.65	23.97
High-middle SDI	Female	Stomach cancer	2018	8.27	7.40	9.28
High-middle SDI	Both	Stomach cancer	2018	13.96	12.60	15.36
Low SDI	Male	Stomach cancer	2018	10.75	9.46	12.03
Low SDI	Female	Stomach cancer	2018	7.46	6.63	8.51
Low SDI	Both	Stomach cancer	2018	9.03	8.18	9.96
Low-middle SDI	Male	Stomach cancer	2018	12.85	11.65	14.06
Low-middle SDI	Female	Stomach cancer	2018	9.11	8.14	10.26
Low-middle SDI	Both	Stomach cancer	2018	10.88	9.99	11.77
Middle SDI	Male	Stomach cancer	2018	20.94	18.17	23.88
Middle SDI	Female	Stomach cancer	2018	9.49	8.30	10.72
Middle SDI	Both	Stomach cancer	2018	14.85	13.24	16.63
High SDI	Male	Stomach cancer	2019	10.23	9.39	10.74
High SDI	Female	Stomach cancer	2019	4.74	4.12	5.11
High SDI	Both	Stomach cancer	2019	7.18	6.50	7.59
High-middle SDI	Male	Stomach cancer	2019	20.99	18.10	23.90
High-middle SDI	Female	Stomach cancer	2019	8.22	7.24	9.31
High-middle SDI	Both	Stomach cancer	2019	13.85	12.41	15.21
Low SDI	Male	Stomach cancer	2019	10.62	9.39	11.92
Low SDI	Female	Stomach cancer	2019	7.40	6.59	8.42
Low SDI	Both	Stomach cancer	2019	8.94	8.09	9.89
Low-middle SDI	Male	Stomach cancer	2019	12.74	11.54	14.11
Low-middle SDI	Female	Stomach cancer	2019	9.03	7.93	10.20
Low-middle SDI	Both	Stomach cancer	2019	10.79	9.86	11.75
Middle SDI	Male	Stomach cancer	2019	20.64	17.70	23.68
Middle SDI	Female	Stomach cancer	2019	9.35	8.06	10.72
Middle SDI	Both	Stomach cancer	2019	14.63	12.98	16.34

location_name	sex_name	cause_name	year	Age-standardised incidence rate (per 100 000 person-years)	95% CI (lower)	95% CI (upper)
Afghanistan	Both	Stomach cancer	2019	27.69	19.94	34.87
Albania	Both	Stomach cancer	2019	11.60	8.72	15.35
Algeria	Both	Stomach cancer	2019	4.98	4.06	6.09
American Samoa	Both	Stomach cancer	2019	15.46	13.04	18.30
Andorra	Both	Stomach cancer	2019	12.10	9.15	15.74
Angola	Both	Stomach cancer	2019	8.39	6.79	10.61
Antigua and Barbuda	Both	Stomach cancer	2019	11.37	9.74	13.05
Argentina	Both	Stomach cancer	2019	9.89	7.87	12.44
Armenia	Both	Stomach cancer	2019	13.40	11.19	15.74
Australia	Both	Stomach cancer	2019	7.01	5.46	8.81
Austria	Both	Stomach cancer	2019	8.43	6.83	10.22
Azerbaijan	Both	Stomach cancer	2019	21.72	18.14	26.38
Bahamas	Both	Stomach cancer	2019	9.35	7.61	11.37
Bahrain	Both	Stomach cancer	2019	6.46	5.24	7.82
Bangladesh	Both	Stomach cancer	2019	6.30	4.83	8.27
Barbados	Both	Stomach cancer	2019	10.91	9.00	12.84
Belarus	Both	Stomach cancer	2019	17.86	14.13	22.99
Belgium	Both	Stomach cancer	2019	7.05	5.59	8.85
Belize	Both	Stomach cancer	2019	10.12	8.74	11.66
Benin	Both	Stomach cancer	2019	12.37	9.96	15.43
Bermuda	Both	Stomach cancer	2019	6.70	5.59	8.00
Bhutan	Both	Stomach cancer	2019	7.96	5.82	10.37
Bolivia (Plurinational State of)	Both	Stomach cancer	2019	34.02	26.85	42.02
Bosnia and Herzegovina	Both	Stomach cancer	2019	10.78	8.52	13.61
Botswana	Both	Stomach cancer	2019	8.66	6.60	10.98
Brazil	Both	Stomach cancer	2019	10.23	9.60	10.71
Brunei Darussalam	Both	Stomach cancer	2019	17.31	15.13	19.53
Bulgaria	Both	Stomach cancer	2019	11.59	9.24	14.50
Burkina Faso	Both	Stomach cancer	2019	14.01	11.52	16.83
Burundi	Both	Stomach cancer	2019	8.43	6.59	10.70
Cabo Verde	Both	Stomach cancer	2019	23.79	20.66	26.99
Cambodia	Both	Stomach cancer	2019	8.88	7.13	10.59
Cameroon	Both	Stomach cancer	2019	13.01	10.12	16.59
Canada	Both	Stomach cancer	2019	7.99	6.25	10.04
Central African Republic	Both	Stomach cancer	2019	11.79	8.93	15.28
Chad	Both	Stomach cancer	2019	14.88	11.80	18.40
Chile	Both	Stomach cancer	2019	19.87	15.80	24.84
China	Both	Stomach cancer	2019	30.64	25.82	36.15
Colombia	Both	Stomach cancer	2019	14.99	11.67	19.02
Comoros	Both	Stomach cancer	2019	6.67	5.30	8.41
Congo	Both	Stomach cancer	2019	8.09	6.57	10.02
Cook Islands	Both	Stomach cancer	2019	7.48	6.20	9.09
Costa Rica	Both	Stomach cancer	2019	23.26	18.21	29.36
Côte d'Ivoire	Both	Stomach cancer	2019	13.33	10.81	16.37
Croatia	Both	Stomach cancer	2019	12.24	9.73	15.18
Cuba	Both	Stomach cancer	2019	6.61	5.38	8.08
Cyprus	Both	Stomach cancer	2019	9.00	7.85	10.27
Czechia	Both	Stomach cancer	2019	8.00	6.50	9.71
Democratic People's Republic of Korea	Both	Stomach cancer	2019	23.40	18.56	28.92
Democratic Republic of the Congo	Both	Stomach cancer	2019	7.62	5.91	9.63
Denmark	Both	Stomach cancer	2019	6.16	4.77	7.80
Djibouti	Both	Stomach cancer	2019	7.60	5.84	10.13
Dominica	Both	Stomach cancer	2019	19.79	16.20	24.01
Dominican Republic	Both	Stomach cancer	2019	8.64	6.54	11.38
Ecuador	Both	Stomach cancer	2019	22.30	17.83	28.19
Egypt	Both	Stomach cancer	2019	5.01	3.82	6.52
El Salvador	Both	Stomach cancer	2019	17.47	13.38	22.37
Equatorial Guinea	Both	Stomach cancer	2019	6.09	4.48	8.40

Eritrea	Both	Stomach cancer	2019	9.93	7.57	12.66
Estonia	Both	Stomach cancer	2019	15.83	12.34	20.28
Eswatini	Both	Stomach cancer	2019	8.57	6.54	11.12
Ethiopia	Both	Stomach cancer	2019	6.17	5.09	7.73
Fiji	Both	Stomach cancer	2019	7.02	5.63	8.76
Finland	Both	Stomach cancer	2019	7.17	5.60	8.98
France	Both	Stomach cancer	2019	6.93	5.38	8.78
Gabon	Both	Stomach cancer	2019	7.68	6.02	9.42
Gambia	Both	Stomach cancer	2019	5.21	4.14	6.41
Georgia	Both	Stomach cancer	2019	14.37	12.09	17.01
Germany	Both	Stomach cancer	2019	9.99	7.75	12.87
Ghana	Both	Stomach cancer	2019	7.82	6.24	9.74
Greece	Both	Stomach cancer	2019	11.03	8.67	13.97
Greenland	Both	Stomach cancer	2019	11.26	9.25	13.40
Grenada	Both	Stomach cancer	2019	10.36	9.31	11.53
Guam	Both	Stomach cancer	2019	6.09	5.08	7.26
Guatemala	Both	Stomach cancer	2019	27.21	21.64	33.73
Guinea	Both	Stomach cancer	2019	15.00	11.88	18.58
Guinea-Bissau	Both	Stomach cancer	2019	18.04	14.30	22.22
Guyana	Both	Stomach cancer	2019	8.60	6.81	10.71
Haiti	Both	Stomach cancer	2019	14.99	9.68	20.05
Honduras	Both	Stomach cancer	2019	15.47	12.69	19.60
Hungary	Both	Stomach cancer	2019	9.60	7.95	11.49
Iceland	Both	Stomach cancer	2019	7.23	6.28	8.31
India	Both	Stomach cancer	2019	7.14	6.12	8.27
Indonesia	Both	Stomach cancer	2019	6.40	5.36	7.28
Iran (Islamic Republic of)	Both	Stomach cancer	2019	16.79	15.49	18.16
Iraq	Both	Stomach cancer	2019	5.78	4.53	7.07
Ireland	Both	Stomach cancer	2019	8.73	6.80	10.94
Israel	Both	Stomach cancer	2019	7.71	6.04	9.80
Italy	Both	Stomach cancer	2019	12.74	10.43	15.13
Jamaica	Both	Stomach cancer	2019	9.76	7.75	12.14
Japan	Both	Stomach cancer	2019	28.29	23.71	33.27
Jordan	Both	Stomach cancer	2019	5.34	4.45	6.41
Kazakhstan	Both	Stomach cancer	2019	14.63	12.77	16.69
Kenya	Both	Stomach cancer	2019	8.65	7.04	10.33
Kiribati	Both	Stomach cancer	2019	15.90	12.53	19.69
Kuwait	Both	Stomach cancer	2019	3.94	3.26	4.76
Kyrgyzstan	Both	Stomach cancer	2019	16.97	14.81	19.24
Lao People's Democratic Republic	Both	Stomach cancer	2019	7.77	5.95	9.66
Latvia	Both	Stomach cancer	2019	14.05	11.76	16.93
Lebanon	Both	Stomach cancer	2019	8.07	6.53	10.55
Lesotho	Both	Stomach cancer	2019	11.52	8.72	14.67
Liberia	Both	Stomach cancer	2019	11.13	8.52	14.25
Libya	Both	Stomach cancer	2019	5.70	4.39	7.23
Lithuania	Both	Stomach cancer	2019	13.93	11.27	17.01
Luxembourg	Both	Stomach cancer	2019	6.68	5.45	8.07
Madagascar	Both	Stomach cancer	2019	6.58	5.03	8.46
Malawi	Both	Stomach cancer	2019	3.28	2.67	3.91
Malaysia	Both	Stomach cancer	2019	7.24	5.86	8.96
Maldives	Both	Stomach cancer	2019	3.79	3.11	4.52
Mali	Both	Stomach cancer	2019	17.12	13.74	21.49
Malta	Both	Stomach cancer	2019	6.79	5.71	7.93
Marshall Islands	Both	Stomach cancer	2019	16.25	12.50	20.54
Mauritania	Both	Stomach cancer	2019	10.16	7.86	12.86
Mauritius	Both	Stomach cancer	2019	7.56	6.15	9.19
Mexico	Both	Stomach cancer	2019	9.70	8.43	11.19
Micronesia (Federated States of)	Both	Stomach cancer	2019	16.85	12.61	21.13
Monaco	Both	Stomach cancer	2019	9.91	7.82	12.01
Mongolia	Both	Stomach cancer	2019	43.70	34.29	55.10

Montenegro	Both	Stomach cancer	2019	8.24	6.88	9.78
Morocco	Both	Stomach cancer	2019	4.60	3.54	5.47
Mozambique	Both	Stomach cancer	2019	6.93	5.51	8.66
Myanmar	Both	Stomach cancer	2019	6.86	5.75	8.30
Namibia	Both	Stomach cancer	2019	3.48	2.83	4.28
Nauru	Both	Stomach cancer	2019	17.90	14.57	21.72
Nepal	Both	Stomach cancer	2019	8.42	6.58	10.66
Netherlands	Both	Stomach cancer	2019	10.23	8.04	12.75
New Zealand	Both	Stomach cancer	2019	6.96	5.75	8.31
Nicaragua	Both	Stomach cancer	2019	15.77	12.99	18.74
Niger	Both	Stomach cancer	2019	13.26	10.13	16.66
Nigeria	Both	Stomach cancer	2019	3.91	3.20	4.77
Niue	Both	Stomach cancer	2019	11.01	9.04	13.30
North Macedonia	Both	Stomach cancer	2019	16.15	12.88	20.23
Northern Mariana Islands	Both	Stomach cancer	2019	13.01	11.03	15.13
Norway	Both	Stomach cancer	2019	6.67	5.65	7.82
Oman	Both	Stomach cancer	2019	8.55	7.51	9.82
Pakistan	Both	Stomach cancer	2019	6.45	5.34	7.79
Palau	Both	Stomach cancer	2019	12.25	9.69	15.32
Palestine	Both	Stomach cancer	2019	7.16	6.13	8.27
Panama	Both	Stomach cancer	2019	12.47	9.70	15.94
Papua New Guinea	Both	Stomach cancer	2019	13.26	9.68	17.25
Paraguay	Both	Stomach cancer	2019	8.91	6.85	11.42
Peru	Both	Stomach cancer	2019	19.56	14.67	25.59
Philippines	Both	Stomach cancer	2019	4.38	3.62	5.27
Poland	Both	Stomach cancer	2019	9.33	7.89	11.08
Portugal	Both	Stomach cancer	2019	14.64	11.41	18.59
Puerto Rico	Both	Stomach cancer	2019	5.83	4.55	7.41
Qatar	Both	Stomach cancer	2019	8.99	6.95	11.39
Republic of Korea	Both	Stomach cancer	2019	28.67	23.65	34.17
Republic of Moldova	Both	Stomach cancer	2019	10.40	8.97	11.94
Romania	Both	Stomach cancer	2019	11.74	9.53	14.35
Russian Federation	Both	Stomach cancer	2019	16.08	14.09	18.39
Rwanda	Both	Stomach cancer	2019	6.87	5.41	8.46
Saint Kitts and Nevis	Both	Stomach cancer	2019	10.69	8.99	12.49
Saint Lucia	Both	Stomach cancer	2019	12.17	10.26	14.21
Saint Vincent and the Grenadines	Both	Stomach cancer	2019	11.18	9.75	12.91
Samoa	Both	Stomach cancer	2019	12.22	9.78	15.23
San Marino	Both	Stomach cancer	2019	26.16	19.98	33.99
Sao Tome and Principe	Both	Stomach cancer	2019	16.58	13.49	20.66
Saudi Arabia	Both	Stomach cancer	2019	4.40	3.60	5.33
Senegal	Both	Stomach cancer	2019	12.66	9.98	15.52
Serbia	Both	Stomach cancer	2019	9.69	7.71	12.09
Seychelles	Both	Stomach cancer	2019	7.15	6.16	8.21
Sierra Leone	Both	Stomach cancer	2019	12.44	9.67	15.78
Singapore	Both	Stomach cancer	2019	10.72	8.60	13.31
Slovakia	Both	Stomach cancer	2019	12.19	9.58	15.17
Slovenia	Both	Stomach cancer	2019	11.94	9.34	15.62
Solomon Islands	Both	Stomach cancer	2019	23.66	18.58	28.89
Somalia	Both	Stomach cancer	2019	9.93	7.35	13.21
South Africa	Both	Stomach cancer	2019	5.39	5.00	5.83
South Sudan	Both	Stomach cancer	2019	6.72	4.92	9.10
Spain	Both	Stomach cancer	2019	11.46	8.95	14.37
Sri Lanka	Both	Stomach cancer	2019	5.43	4.12	7.13
Sudan	Both	Stomach cancer	2019	14.95	10.27	19.41
Suriname	Both	Stomach cancer	2019	7.12	5.94	8.54
Sweden	Both	Stomach cancer	2019	5.01	4.20	5.86
Switzerland	Both	Stomach cancer	2019	6.64	5.18	8.42
Syrian Arab Republic	Both	Stomach cancer	2019	4.97	3.78	6.55
Taiwan (Province of China)	Both	Stomach cancer	2019	15.60	12.23	20.26

Tajikistan	Both	Stomach cancer	2019	24.01	19.72	29.11
Thailand	Both	Stomach cancer	2019	5.90	4.44	7.81
Timor-Leste	Both	Stomach cancer	2019	7.30	5.36	8.99
Togo	Both	Stomach cancer	2019	13.02	10.51	16.39
Tokelau	Both	Stomach cancer	2019	9.68	7.70	12.16
Tonga	Both	Stomach cancer	2019	13.34	10.91	16.05
Trinidad and Tobago	Both	Stomach cancer	2019	4.49	3.41	5.78
Tunisia	Both	Stomach cancer	2019	5.57	4.15	7.48
Turkey	Both	Stomach cancer	2019	11.91	9.51	14.60
Turkmenistan	Both	Stomach cancer	2019	9.35	7.44	11.66
Tuvalu	Both	Stomach cancer	2019	13.25	10.32	17.00
Uganda	Both	Stomach cancer	2019	8.16	6.59	9.84
Ukraine	Both	Stomach cancer	2019	16.34	13.70	19.23
United Arab Emirates	Both	Stomach cancer	2019	8.84	6.91	10.98
United Kingdom	Both	Stomach cancer	2019	7.37	6.16	8.79
United Republic of Tanzania	Both	Stomach cancer	2019	7.18	5.84	8.56
United States of America	Both	Stomach cancer	2019	5.89	5.10	6.87
United States Virgin Islands	Both	Stomach cancer	2019	10.32	8.65	12.03
Uruguay	Both	Stomach cancer	2019	11.34	8.93	14.19
Uzbekistan	Both	Stomach cancer	2019	13.46	11.39	15.56
Vanuatu	Both	Stomach cancer	2019	14.99	11.05	19.54
Venezuela (Bolivarian Republic of)	Both	Stomach cancer	2019	13.94	10.57	17.87
Viet Nam	Both	Stomach cancer	2019	10.59	8.51	12.64
Yemen	Both	Stomach cancer	2019	19.18	14.73	25.05
Zambia	Both	Stomach cancer	2019	8.19	6.46	10.12
Zimbabwe	Both	Stomach cancer	2019	13.11	10.24	16.39

location_name	sex_name	cause_name	year	Age-standardised death rate (per 100 000 person-years)	95% CI (lower)	95% CI (upper)
Afghanistan	Both	Stomach cancer	2019	29.30	21.25	36.52
Albania	Both	Stomach cancer	2019	10.68	8.05	14.07
Algeria	Both	Stomach cancer	2019	5.10	4.17	6.18
American Samoa	Both	Stomach cancer	2019	15.79	13.39	18.55
Andorra	Both	Stomach cancer	2019	8.30	6.34	10.66
Angola	Both	Stomach cancer	2019	8.96	7.31	11.24
Antigua and Barbuda	Both	Stomach cancer	2019	11.05	9.48	12.67
Argentina	Both	Stomach cancer	2019	9.44	8.78	10.15
Armenia	Both	Stomach cancer	2019	13.01	10.90	15.23
Australia	Both	Stomach cancer	2019	3.89	3.51	4.23
Austria	Both	Stomach cancer	2019	5.42	4.96	5.87
Azerbaijan	Both	Stomach cancer	2019	22.48	18.75	27.33
Bahamas	Both	Stomach cancer	2019	9.16	7.51	11.14
Bahrain	Both	Stomach cancer	2019	6.29	5.13	7.58
Bangladesh	Both	Stomach cancer	2019	6.58	5.07	8.60
Barbados	Both	Stomach cancer	2019	10.41	8.65	12.23
Belarus	Both	Stomach cancer	2019	14.10	11.16	17.92
Belgium	Both	Stomach cancer	2019	5.12	4.67	5.54
Belize	Both	Stomach cancer	2019	10.17	8.81	11.72
Benin	Both	Stomach cancer	2019	13.66	11.09	16.75
Bermuda	Both	Stomach cancer	2019	5.07	4.24	6.01
Bhutan	Both	Stomach cancer	2019	8.41	6.21	10.85
Bolivia (Plurinational State of)	Both	Stomach cancer	2019	36.11	28.77	44.26
Bosnia and Herzegovina	Both	Stomach cancer	2019	10.31	8.24	12.97
Botswana	Both	Stomach cancer	2019	9.04	6.99	11.42
Brazil	Both	Stomach cancer	2019	9.87	9.14	10.37
Brunei Darussalam	Both	Stomach cancer	2019	14.11	12.38	15.85
Bulgaria	Both	Stomach cancer	2019	10.81	8.65	13.46
Burkina Faso	Both	Stomach cancer	2019	15.38	12.72	18.31
Burundi	Both	Stomach cancer	2019	8.99	7.12	11.37
Cabo Verde	Both	Stomach cancer	2019	25.65	22.46	28.98
Cambodia	Both	Stomach cancer	2019	9.35	7.53	11.05
Cameroon	Both	Stomach cancer	2019	14.24	11.19	17.99
Canada	Both	Stomach cancer	2019	4.40	4.00	4.76
Central African Republic	Both	Stomach cancer	2019	12.46	9.46	16.00
Chad	Both	Stomach cancer	2019	16.38	13.04	20.17
Chile	Both	Stomach cancer	2019	17.63	16.17	18.99
China	Both	Stomach cancer	2019	21.72	18.31	25.31
Colombia	Both	Stomach cancer	2019	12.73	9.91	16.09
Comoros	Both	Stomach cancer	2019	7.16	5.76	8.95
Congo	Both	Stomach cancer	2019	8.67	7.14	10.66
Cook Islands	Both	Stomach cancer	2019	6.75	5.66	8.16
Costa Rica	Both	Stomach cancer	2019	19.02	14.80	23.93
Côte d'Ivoire	Both	Stomach cancer	2019	14.67	12.01	17.84
Croatia	Both	Stomach cancer	2019	9.38	7.50	11.57
Cuba	Both	Stomach cancer	2019	5.67	4.63	6.89
Cyprus	Both	Stomach cancer	2019	6.66	5.87	7.60
Czechia	Both	Stomach cancer	2019	6.08	4.98	7.33
Democratic People's Republic of Korea	Both	Stomach cancer	2019	22.49	17.92	27.59
Democratic Republic of the Congo	Both	Stomach cancer	2019	8.11	6.32	10.24
Denmark	Both	Stomach cancer	2019	4.33	3.93	4.72
Djibouti	Both	Stomach cancer	2019	8.17	6.37	10.74
Dominica	Both	Stomach cancer	2019	20.49	16.91	24.66
Dominican Republic	Both	Stomach cancer	2019	8.70	6.68	11.31
Ecuador	Both	Stomach cancer	2019	21.86	17.57	27.51
Egypt	Both	Stomach cancer	2019	5.21	4.00	6.76

El Salvador	Both	Stomach cancer	2019	16.24	12.51	20.62
Equatorial Guinea	Both	Stomach cancer	2019	6.58	4.92	9.00
Eritrea	Both	Stomach cancer	2019	10.45	8.07	13.25
Estonia	Both	Stomach cancer	2019	11.38	8.93	14.48
Eswatini	Both	Stomach cancer	2019	9.15	6.98	11.75
Ethiopia	Both	Stomach cancer	2019	6.64	5.45	8.34
Fiji	Both	Stomach cancer	2019	7.44	5.99	9.19
Finland	Both	Stomach cancer	2019	4.78	4.35	5.20
France	Both	Stomach cancer	2019	4.86	4.36	5.27
Gabon	Both	Stomach cancer	2019	8.15	6.43	9.99
Gambia	Both	Stomach cancer	2019	5.81	4.64	7.07
Georgia	Both	Stomach cancer	2019	14.07	11.86	16.62
Germany	Both	Stomach cancer	2019	6.86	6.29	7.40
Ghana	Both	Stomach cancer	2019	8.65	6.92	10.76
Greece	Both	Stomach cancer	2019	8.05	7.39	8.65
Greenland	Both	Stomach cancer	2019	10.82	8.84	12.93
Grenada	Both	Stomach cancer	2019	10.43	9.38	11.58
Guam	Both	Stomach cancer	2019	5.74	4.82	6.79
Guatemala	Both	Stomach cancer	2019	27.97	22.45	34.43
Guinea	Both	Stomach cancer	2019	16.24	12.95	20.00
Guinea-Bissau	Both	Stomach cancer	2019	19.24	15.38	23.58
Guyana	Both	Stomach cancer	2019	8.87	7.07	11.02
Haiti	Both	Stomach cancer	2019	15.99	10.48	21.19
Honduras	Both	Stomach cancer	2019	15.88	13.16	20.02
Hungary	Both	Stomach cancer	2019	8.57	7.11	10.20
Iceland	Both	Stomach cancer	2019	4.59	4.05	5.12
India	Both	Stomach cancer	2019	7.32	6.30	8.44
Indonesia	Both	Stomach cancer	2019	6.82	5.74	7.68
Iran (Islamic Republic of)	Both	Stomach cancer	2019	16.17	14.86	17.40
Iraq	Both	Stomach cancer	2019	5.71	4.55	6.89
Ireland	Both	Stomach cancer	2019	5.79	5.25	6.32
Israel	Both	Stomach cancer	2019	6.04	5.48	6.52
Italy	Both	Stomach cancer	2019	8.26	7.50	8.72
Jamaica	Both	Stomach cancer	2019	9.53	7.62	11.78
Japan	Both	Stomach cancer	2019	14.07	12.45	15.01
Jordan	Both	Stomach cancer	2019	5.14	4.31	6.12
Kazakhstan	Both	Stomach cancer	2019	14.11	12.33	16.09
Kenya	Both	Stomach cancer	2019	9.83	8.11	11.80
Kiribati	Both	Stomach cancer	2019	16.60	13.28	20.33
Kuwait	Both	Stomach cancer	2019	3.45	2.86	4.15
Kyrgyzstan	Both	Stomach cancer	2019	16.78	14.69	19.02
Lao People's Democratic Republic	Both	Stomach cancer	2019	8.27	6.40	10.19
Latvia	Both	Stomach cancer	2019	11.56	9.72	13.89
Lebanon	Both	Stomach cancer	2019	6.78	5.51	8.97
Lesotho	Both	Stomach cancer	2019	12.35	9.40	15.57
Liberia	Both	Stomach cancer	2019	12.33	9.54	15.64
Libya	Both	Stomach cancer	2019	5.64	4.36	7.12
Lithuania	Both	Stomach cancer	2019	11.66	9.45	14.13
Luxembourg	Both	Stomach cancer	2019	4.75	4.07	5.42
Madagascar	Both	Stomach cancer	2019	7.02	5.40	8.95
Malawi	Both	Stomach cancer	2019	3.60	2.96	4.24
Malaysia	Both	Stomach cancer	2019	7.04	5.73	8.64
Maldives	Both	Stomach cancer	2019	3.60	2.98	4.26
Mali	Both	Stomach cancer	2019	18.22	14.75	22.65
Malta	Both	Stomach cancer	2019	4.93	4.34	5.55
Marshall Islands	Both	Stomach cancer	2019	16.99	13.14	21.22
Mauritania	Both	Stomach cancer	2019	11.29	8.81	14.13
Mauritius	Both	Stomach cancer	2019	7.14	5.86	8.62

Mexico	Both	Stomach cancer	2019	8.86	7.64	10.16
Micronesia (Federated States of)	Both	Stomach cancer	2019	17.40	13.30	21.65
Monaco	Both	Stomach cancer	2019	6.62	5.31	7.86
Mongolia	Both	Stomach cancer	2019	46.04	36.30	57.48
Montenegro	Both	Stomach cancer	2019	7.47	6.28	8.82
Morocco	Both	Stomach cancer	2019	4.84	3.76	5.72
Mozambique	Both	Stomach cancer	2019	7.59	6.06	9.42
Myanmar	Both	Stomach cancer	2019	7.22	6.10	8.66
Namibia	Both	Stomach cancer	2019	3.80	3.11	4.63
Nauru	Both	Stomach cancer	2019	17.94	14.73	21.60
Nepal	Both	Stomach cancer	2019	8.96	7.05	11.34
Netherlands	Both	Stomach cancer	2019	6.86	6.20	7.45
New Zealand	Both	Stomach cancer	2019	4.65	4.26	4.98
Nicaragua	Both	Stomach cancer	2019	15.03	12.54	17.62
Niger	Both	Stomach cancer	2019	14.68	11.25	18.24
Nigeria	Both	Stomach cancer	2019	4.43	3.60	5.42
Niue	Both	Stomach cancer	2019	10.43	8.58	12.41
North Macedonia	Both	Stomach cancer	2019	15.32	12.30	19.13
Northern Mariana Islands	Both	Stomach cancer	2019	12.02	10.31	13.89
Norway	Both	Stomach cancer	2019	4.49	4.12	4.82
Oman	Both	Stomach cancer	2019	7.98	6.95	9.13
Pakistan	Both	Stomach cancer	2019	6.87	5.71	8.31
Palau	Both	Stomach cancer	2019	11.10	8.87	13.73
Palestine	Both	Stomach cancer	2019	7.12	6.13	8.22
Panama	Both	Stomach cancer	2019	10.76	8.45	13.63
Papua New Guinea	Both	Stomach cancer	2019	13.95	10.27	18.04
Paraguay	Both	Stomach cancer	2019	8.72	6.76	11.08
Peru	Both	Stomach cancer	2019	17.81	13.44	23.13
Philippines	Both	Stomach cancer	2019	4.50	3.78	5.30
Poland	Both	Stomach cancer	2019	9.66	8.10	11.35
Portugal	Both	Stomach cancer	2019	11.95	10.94	12.82
Puerto Rico	Both	Stomach cancer	2019	4.74	3.70	5.97
Qatar	Both	Stomach cancer	2019	8.26	6.47	10.36
Republic of Korea	Both	Stomach cancer	2019	14.09	12.57	15.60
Republic of Moldova	Both	Stomach cancer	2019	9.32	8.04	10.66
Romania	Both	Stomach cancer	2019	10.46	8.56	12.70
Russian Federation	Both	Stomach cancer	2019	13.22	11.53	15.08
Rwanda	Both	Stomach cancer	2019	7.36	5.87	9.03
Saint Kitts and Nevis	Both	Stomach cancer	2019	9.99	8.52	11.56
Saint Lucia	Both	Stomach cancer	2019	12.05	10.18	14.05
Saint Vincent and the Grenadines	Both	Stomach cancer	2019	11.41	10.02	13.14
Samoa	Both	Stomach cancer	2019	12.40	10.02	15.31
San Marino	Both	Stomach cancer	2019	18.55	12.51	25.86
Sao Tome and Principe	Both	Stomach cancer	2019	18.23	14.88	22.59
Saudi Arabia	Both	Stomach cancer	2019	4.10	3.36	4.95
Senegal	Both	Stomach cancer	2019	14.01	11.18	17.10
Serbia	Both	Stomach cancer	2019	8.70	6.95	10.83
Seychelles	Both	Stomach cancer	2019	6.85	5.96	7.82
Sierra Leone	Both	Stomach cancer	2019	13.68	10.73	17.25
Singapore	Both	Stomach cancer	2019	5.54	4.87	6.05
Slovakia	Both	Stomach cancer	2019	8.32	6.61	10.31
Slovenia	Both	Stomach cancer	2019	8.54	6.71	10.95
Solomon Islands	Both	Stomach cancer	2019	23.87	18.94	29.03
Somalia	Both	Stomach cancer	2019	10.62	7.89	13.96
South Africa	Both	Stomach cancer	2019	5.70	5.32	6.09
South Sudan	Both	Stomach cancer	2019	7.30	5.31	9.89
Spain	Both	Stomach cancer	2019	7.02	6.39	7.61
Sri Lanka	Both	Stomach cancer	2019	5.04	3.87	6.55

Sudan	Both	Stomach cancer	2019	15.63	10.93	20.20
Suriname	Both	Stomach cancer	2019	7.28	6.09	8.70
Sweden	Both	Stomach cancer	2019	3.67	3.34	3.93
Switzerland	Both	Stomach cancer	2019	4.03	3.63	4.38
Syrian Arab Republic	Both	Stomach cancer	2019	4.97	3.82	6.47
Taiwan (Province of China)	Both	Stomach cancer	2019	10.72	8.42	13.73
Tajikistan	Both	Stomach cancer	2019	25.26	20.77	30.44
Thailand	Both	Stomach cancer	2019	5.22	3.93	6.85
Timor-Leste	Both	Stomach cancer	2019	7.85	5.83	9.60
Togo	Both	Stomach cancer	2019	14.17	11.52	17.72
Tokelau	Both	Stomach cancer	2019	9.86	8.00	12.28
Tonga	Both	Stomach cancer	2019	13.85	11.50	16.52
Trinidad and Tobago	Both	Stomach cancer	2019	4.54	3.46	5.81
Tunisia	Both	Stomach cancer	2019	5.19	3.89	6.94
Turkey	Both	Stomach cancer	2019	10.75	8.59	13.18
Turkmenistan	Both	Stomach cancer	2019	9.23	7.35	11.45
Tuvalu	Both	Stomach cancer	2019	13.67	10.71	17.41
Uganda	Both	Stomach cancer	2019	8.64	7.02	10.41
Ukraine	Both	Stomach cancer	2019	13.46	11.41	15.82
United Arab Emirates	Both	Stomach cancer	2019	9.23	7.31	11.38
United Kingdom	Both	Stomach cancer	2019	5.62	5.19	5.88
United Republic of Tanzania	Both	Stomach cancer	2019	7.68	6.29	9.16
United States of America	Both	Stomach cancer	2019	3.40	3.19	3.54
United States Virgin Islands	Both	Stomach cancer	2019	9.79	8.29	11.34
Uruguay	Both	Stomach cancer	2019	10.66	9.78	11.49
Uzbekistan	Both	Stomach cancer	2019	13.43	11.44	15.44
Vanuatu	Both	Stomach cancer	2019	15.75	11.67	20.34
Venezuela (Bolivarian Republic of)	Both	Stomach cancer	2019	12.58	9.63	16.01
Viet Nam	Both	Stomach cancer	2019	9.98	8.14	11.84
Yemen	Both	Stomach cancer	2019	20.20	15.54	26.28
Zambia	Both	Stomach cancer	2019	8.68	6.91	10.64
Zimbabwe	Both	Stomach cancer	2019	13.73	10.81	17.17

sex_name	cause_name	year	Age-standardised incidence rate (per 100 000 person-years)	95% CI (lower)	95% CI (upper)
Male	Esophageal cancer	1990	11.71	9.35	13.06
Female	Esophageal cancer	1990	4.91	3.55	5.51
Both	Esophageal cancer	1990	8.06	6.41	8.83
Male	Esophageal cancer	1991	11.76	9.27	13.07
Female	Esophageal cancer	1991	4.92	3.48	5.51
Both	Esophageal cancer	1991	8.09	6.38	8.84
Male	Esophageal cancer	1992	11.75	9.25	12.97
Female	Esophageal cancer	1992	4.92	3.51	5.47
Both	Esophageal cancer	1992	8.10	6.32	8.80
Male	Esophageal cancer	1993	11.83	9.40	13.04
Female	Esophageal cancer	1993	4.96	3.52	5.50
Both	Esophageal cancer	1993	8.15	6.35	8.82
Male	Esophageal cancer	1994	11.80	9.37	12.88
Female	Esophageal cancer	1994	4.91	3.45	5.43
Both	Esophageal cancer	1994	8.12	6.38	8.80
Male	Esophageal cancer	1995	11.83	9.34	12.89
Female	Esophageal cancer	1995	4.85	3.37	5.35
Both	Esophageal cancer	1995	8.10	6.30	8.72
Male	Esophageal cancer	1996	11.80	9.25	12.77
Female	Esophageal cancer	1996	4.84	3.37	5.33
Both	Esophageal cancer	1996	8.09	6.25	8.66
Male	Esophageal cancer	1997	11.79	9.26	12.81
Female	Esophageal cancer	1997	4.85	3.32	5.32
Both	Esophageal cancer	1997	8.08	6.20	8.65
Male	Esophageal cancer	1998	11.90	9.31	12.86
Female	Esophageal cancer	1998	4.84	3.25	5.32
Both	Esophageal cancer	1998	8.13	6.18	8.68
Male	Esophageal cancer	1999	12.06	9.30	13.06
Female	Esophageal cancer	1999	4.88	3.28	5.35
Both	Esophageal cancer	1999	8.22	6.16	8.80
Male	Esophageal cancer	2000	12.30	9.39	13.35
Female	Esophageal cancer	2000	4.96	3.27	5.44
Both	Esophageal cancer	2000	8.38	6.25	9.02
Male	Esophageal cancer	2001	12.51	9.40	13.65
Female	Esophageal cancer	2001	4.99	3.21	5.52
Both	Esophageal cancer	2001	8.50	6.14	9.20
Male	Esophageal cancer	2002	12.71	9.48	13.88
Female	Esophageal cancer	2002	5.07	3.26	5.62
Both	Esophageal cancer	2002	8.64	6.23	9.32
Male	Esophageal cancer	2003	12.92	9.57	14.11
Female	Esophageal cancer	2003	5.13	3.27	5.66
Both	Esophageal cancer	2003	8.77	6.29	9.47
Male	Esophageal cancer	2004	13.06	9.50	14.32
Female	Esophageal cancer	2004	5.09	3.25	5.64
Both	Esophageal cancer	2004	8.82	6.25	9.57
Male	Esophageal cancer	2005	12.98	9.48	14.12
Female	Esophageal cancer	2005	4.94	3.14	5.44
Both	Esophageal cancer	2005	8.70	6.25	9.39

Male	Esophageal cancer	2006	12.56	9.30	13.64
Female	Esophageal cancer	2006	4.70	3.10	5.16
Both	Esophageal cancer	2006	8.38	6.11	9.02
Male	Esophageal cancer	2007	12.23	9.32	13.28
Female	Esophageal cancer	2007	4.49	3.08	4.91
Both	Esophageal cancer	2007	8.12	6.04	8.73
Male	Esophageal cancer	2008	12.00	9.44	12.96
Female	Esophageal cancer	2008	4.30	3.03	4.68
Both	Esophageal cancer	2008	7.90	6.09	8.47
Male	Esophageal cancer	2009	11.75	9.40	12.66
Female	Esophageal cancer	2009	4.12	2.95	4.49
Both	Esophageal cancer	2009	7.69	6.06	8.21
Male	Esophageal cancer	2010	11.53	9.47	12.47
Female	Esophageal cancer	2010	3.97	2.93	4.32
Both	Esophageal cancer	2010	7.50	6.10	8.02
Male	Esophageal cancer	2011	11.21	9.41	12.12
Female	Esophageal cancer	2011	3.80	2.93	4.16
Both	Esophageal cancer	2011	7.26	6.03	7.78
Male	Esophageal cancer	2012	10.94	9.33	11.84
Female	Esophageal cancer	2012	3.61	2.87	3.94
Both	Esophageal cancer	2012	7.04	6.04	7.53
Male	Esophageal cancer	2013	10.62	9.18	11.51
Female	Esophageal cancer	2013	3.49	2.85	3.78
Both	Esophageal cancer	2013	6.82	5.91	7.31
Male	Esophageal cancer	2014	10.37	9.10	11.31
Female	Esophageal cancer	2014	3.41	2.78	3.70
Both	Esophageal cancer	2014	6.66	5.83	7.14
Male	Esophageal cancer	2015	10.21	8.97	11.21
Female	Esophageal cancer	2015	3.35	2.83	3.64
Both	Esophageal cancer	2015	6.55	5.85	7.04
Male	Esophageal cancer	2016	10.09	8.97	11.12
Female	Esophageal cancer	2016	3.31	2.76	3.66
Both	Esophageal cancer	2016	6.48	5.80	7.03
Male	Esophageal cancer	2017	10.02	8.78	11.17
Female	Esophageal cancer	2017	3.30	2.75	3.68
Both	Esophageal cancer	2017	6.44	5.79	7.00
Male	Esophageal cancer	2018	10.06	8.76	11.27
Female	Esophageal cancer	2018	3.31	2.76	3.72
Both	Esophageal cancer	2018	6.46	5.71	7.09
Male	Esophageal cancer	2019	10.13	8.73	11.56
Female	Esophageal cancer	2019	3.33	2.74	3.77
Both	Esophageal cancer	2019	6.51	5.69	7.25

location_name	sex_name	cause_name	year	Age-standardised incidence rate (per 100 000 person-years)	95% CI (lower)	95% CI (upper)
High SDI	Male	Esophageal cancer	1990	8.85	8.64	9.03
High SDI	Female	Esophageal cancer	1990	2.06	1.95	2.13
High SDI	Both	Esophageal cancer	1990	5.07	4.93	5.17
High-middle SDI	Male	Esophageal cancer	1990	13.05	11.03	14.63
High-middle SDI	Female	Esophageal cancer	1990	4.22	3.32	4.78
High-middle SDI	Both	Esophageal cancer	1990	8.08	6.86	8.85
Low SDI	Male	Esophageal cancer	1990	6.71	5.24	7.93
Low SDI	Female	Esophageal cancer	1990	5.29	4.20	6.33
Low SDI	Both	Esophageal cancer	1990	6.02	5.00	6.89
Low-middle SDI	Male	Esophageal cancer	1990	6.24	5.52	8.21
Low-middle SDI	Female	Esophageal cancer	1990	3.86	3.23	5.28
Low-middle SDI	Both	Esophageal cancer	1990	5.07	4.51	6.54
Middle SDI	Male	Esophageal cancer	1990	17.63	10.89	21.04
Middle SDI	Female	Esophageal cancer	1990	9.54	4.99	11.32
Middle SDI	Both	Esophageal cancer	1990	13.49	8.11	15.54
High SDI	Male	Esophageal cancer	1991	8.97	8.74	9.14
High SDI	Female	Esophageal cancer	1991	2.08	1.96	2.14
High SDI	Both	Esophageal cancer	1991	5.14	4.98	5.23
High-middle SDI	Male	Esophageal cancer	1991	13.00	10.81	14.58
High-middle SDI	Female	Esophageal cancer	1991	4.17	3.28	4.70
High-middle SDI	Both	Esophageal cancer	1991	8.04	6.76	8.80
Low SDI	Male	Esophageal cancer	1991	5.65	4.53	6.74
Low SDI	Female	Esophageal cancer	1991	5.27	4.22	6.27
Low SDI	Both	Esophageal cancer	1991	5.48	4.63	6.31
Low-middle SDI	Male	Esophageal cancer	1991	6.16	5.46	8.21
Low-middle SDI	Female	Esophageal cancer	1991	3.81	3.18	5.26
Low-middle SDI	Both	Esophageal cancer	1991	5.00	4.46	6.51
Middle SDI	Male	Esophageal cancer	1991	14.17	8.45	16.75
Middle SDI	Female	Esophageal cancer	1991	9.59	4.84	11.30
Middle SDI	Both	Esophageal cancer	1991	11.85	6.88	13.61
High SDI	Male	Esophageal cancer	1992	9.08	8.86	9.23
High SDI	Female	Esophageal cancer	1992	2.09	1.98	2.16
High SDI	Both	Esophageal cancer	1992	5.20	5.05	5.30
High-middle SDI	Male	Esophageal cancer	1992	12.99	10.84	14.40
High-middle SDI	Female	Esophageal cancer	1992	4.17	3.29	4.67
High-middle SDI	Both	Esophageal cancer	1992	8.05	6.82	8.79
Low SDI	Male	Esophageal cancer	1992	5.63	4.46	6.70
Low SDI	Female	Esophageal cancer	1992	5.26	4.22	6.26
Low SDI	Both	Esophageal cancer	1992	5.46	4.63	6.24
Low-middle SDI	Male	Esophageal cancer	1992	6.13	5.43	8.21
Low-middle SDI	Female	Esophageal cancer	1992	3.81	3.17	5.27
Low-middle SDI	Both	Esophageal cancer	1992	4.98	4.44	6.53
Middle SDI	Male	Esophageal cancer	1992	14.25	8.54	16.80
Middle SDI	Female	Esophageal cancer	1992	9.56	4.86	11.17
Middle SDI	Both	Esophageal cancer	1992	11.87	6.94	13.57
High SDI	Male	Esophageal cancer	1993	9.26	9.03	9.41
High SDI	Female	Esophageal cancer	1993	2.13	2.01	2.20
High SDI	Both	Esophageal cancer	1993	5.31	5.15	5.40
High-middle SDI	Male	Esophageal cancer	1993	13.11	10.99	14.53
High-middle SDI	Female	Esophageal cancer	1993	4.22	3.29	4.70

High-middle SDI	Both	Esophageal cancer	1993	8.14	6.90	8.82
Low SDI	Male	Esophageal cancer	1993	5.61	4.51	6.64
Low SDI	Female	Esophageal cancer	1993	5.24	4.19	6.21
Low SDI	Both	Esophageal cancer	1993	5.44	4.57	6.25
Low-middle SDI	Male	Esophageal cancer	1993	6.13	5.45	8.18
Low-middle SDI	Female	Esophageal cancer	1993	3.78	3.19	5.22
Low-middle SDI	Both	Esophageal cancer	1993	4.96	4.47	6.45
Middle SDI	Male	Esophageal cancer	1993	14.14	8.56	16.66
Middle SDI	Female	Esophageal cancer	1993	9.56	4.77	11.16
Middle SDI	Both	Esophageal cancer	1993	11.83	6.81	13.42
High SDI	Male	Esophageal cancer	1994	9.30	9.08	9.46
High SDI	Female	Esophageal cancer	1994	2.13	2.01	2.20
High SDI	Both	Esophageal cancer	1994	5.34	5.18	5.43
High-middle SDI	Male	Esophageal cancer	1994	13.14	11.03	14.54
High-middle SDI	Female	Esophageal cancer	1994	4.19	3.20	4.66
High-middle SDI	Both	Esophageal cancer	1994	8.14	6.82	8.83
Low SDI	Male	Esophageal cancer	1994	5.60	4.48	6.65
Low SDI	Female	Esophageal cancer	1994	5.22	4.24	6.22
Low SDI	Both	Esophageal cancer	1994	5.42	4.59	6.21
Low-middle SDI	Male	Esophageal cancer	1994	6.12	5.47	8.24
Low-middle SDI	Female	Esophageal cancer	1994	3.75	3.20	5.12
Low-middle SDI	Both	Esophageal cancer	1994	4.94	4.43	6.50
Middle SDI	Male	Esophageal cancer	1994	14.16	8.59	16.44
Middle SDI	Female	Esophageal cancer	1994	9.39	4.62	10.83
Middle SDI	Both	Esophageal cancer	1994	11.75	6.80	13.29
High SDI	Male	Esophageal cancer	1995	9.41	9.16	9.57
High SDI	Female	Esophageal cancer	1995	2.14	2.02	2.21
High SDI	Both	Esophageal cancer	1995	5.40	5.23	5.50
High-middle SDI	Male	Esophageal cancer	1995	13.09	10.85	14.38
High-middle SDI	Female	Esophageal cancer	1995	4.12	3.07	4.61
High-middle SDI	Both	Esophageal cancer	1995	8.09	6.63	8.74
Low SDI	Male	Esophageal cancer	1995	5.64	4.46	6.70
Low SDI	Female	Esophageal cancer	1995	5.24	4.24	6.21
Low SDI	Both	Esophageal cancer	1995	5.45	4.54	6.25
Low-middle SDI	Male	Esophageal cancer	1995	6.09	5.48	8.18
Low-middle SDI	Female	Esophageal cancer	1995	3.71	3.17	5.04
Low-middle SDI	Both	Esophageal cancer	1995	4.91	4.42	6.40
Middle SDI	Male	Esophageal cancer	1995	14.20	8.55	16.44
Middle SDI	Female	Esophageal cancer	1995	9.17	4.52	10.63
Middle SDI	Both	Esophageal cancer	1995	11.65	6.67	13.12
High SDI	Male	Esophageal cancer	1996	9.42	9.17	9.58
High SDI	Female	Esophageal cancer	1996	2.15	2.03	2.22
High SDI	Both	Esophageal cancer	1996	5.41	5.24	5.51
High-middle SDI	Male	Esophageal cancer	1996	13.01	10.55	14.26
High-middle SDI	Female	Esophageal cancer	1996	4.10	3.00	4.57
High-middle SDI	Both	Esophageal cancer	1996	8.04	6.47	8.70
Low SDI	Male	Esophageal cancer	1996	5.66	4.55	6.72
Low SDI	Female	Esophageal cancer	1996	5.25	4.22	6.21
Low SDI	Both	Esophageal cancer	1996	5.47	4.60	6.26
Low-middle SDI	Male	Esophageal cancer	1996	6.12	5.52	8.14
Low-middle SDI	Female	Esophageal cancer	1996	3.71	3.18	4.98
Low-middle SDI	Both	Esophageal cancer	1996	4.92	4.44	6.43

Middle SDI	Male	Esophageal cancer	1996	14.13	8.44	16.20
Middle SDI	Female	Esophageal cancer	1996	9.12	4.41	10.52
Middle SDI	Both	Esophageal cancer	1996	11.59	6.66	13.01
High SDI	Male	Esophageal cancer	1997	9.42	9.16	9.58
High SDI	Female	Esophageal cancer	1997	2.14	2.02	2.22
High SDI	Both	Esophageal cancer	1997	5.41	5.24	5.51
High-middle SDI	Male	Esophageal cancer	1997	12.99	10.41	14.31
High-middle SDI	Female	Esophageal cancer	1997	4.10	2.94	4.56
High-middle SDI	Both	Esophageal cancer	1997	8.04	6.31	8.72
Low SDI	Male	Esophageal cancer	1997	5.68	4.60	6.68
Low SDI	Female	Esophageal cancer	1997	5.25	4.16	6.21
Low SDI	Both	Esophageal cancer	1997	5.48	4.59	6.23
Low-middle SDI	Male	Esophageal cancer	1997	6.22	5.59	8.45
Low-middle SDI	Female	Esophageal cancer	1997	3.76	3.24	5.01
Low-middle SDI	Both	Esophageal cancer	1997	4.99	4.50	6.54
Middle SDI	Male	Esophageal cancer	1997	14.06	8.52	16.04
Middle SDI	Female	Esophageal cancer	1997	9.06	4.35	10.38
Middle SDI	Both	Esophageal cancer	1997	11.53	6.57	12.88
High SDI	Male	Esophageal cancer	1998	9.56	9.29	9.73
High SDI	Female	Esophageal cancer	1998	2.16	2.04	2.23
High SDI	Both	Esophageal cancer	1998	5.49	5.31	5.60
High-middle SDI	Male	Esophageal cancer	1998	13.08	10.35	14.37
High-middle SDI	Female	Esophageal cancer	1998	4.10	2.87	4.57
High-middle SDI	Both	Esophageal cancer	1998	8.07	6.29	8.74
Low SDI	Male	Esophageal cancer	1998	5.70	4.58	6.67
Low SDI	Female	Esophageal cancer	1998	5.26	4.20	6.23
Low SDI	Both	Esophageal cancer	1998	5.49	4.63	6.23
Low-middle SDI	Male	Esophageal cancer	1998	6.23	5.61	8.37
Low-middle SDI	Female	Esophageal cancer	1998	3.74	3.20	5.05
Low-middle SDI	Both	Esophageal cancer	1998	4.98	4.49	6.49
Middle SDI	Male	Esophageal cancer	1998	14.16	8.56	16.14
Middle SDI	Female	Esophageal cancer	1998	8.96	4.21	10.24
Middle SDI	Both	Esophageal cancer	1998	11.51	6.46	12.80
High SDI	Male	Esophageal cancer	1999	9.68	9.40	9.87
High SDI	Female	Esophageal cancer	1999	2.18	2.05	2.26
High SDI	Both	Esophageal cancer	1999	5.56	5.37	5.68
High-middle SDI	Male	Esophageal cancer	1999	13.34	10.26	14.65
High-middle SDI	Female	Esophageal cancer	1999	4.15	2.84	4.66
High-middle SDI	Both	Esophageal cancer	1999	8.23	6.26	8.92
Low SDI	Male	Esophageal cancer	1999	5.67	4.58	6.64
Low SDI	Female	Esophageal cancer	1999	5.23	4.17	6.21
Low SDI	Both	Esophageal cancer	1999	5.46	4.60	6.19
Low-middle SDI	Male	Esophageal cancer	1999	6.17	5.57	8.22
Low-middle SDI	Female	Esophageal cancer	1999	3.70	3.19	4.89
Low-middle SDI	Both	Esophageal cancer	1999	4.93	4.45	6.37
Middle SDI	Male	Esophageal cancer	1999	14.35	8.39	16.34
Middle SDI	Female	Esophageal cancer	1999	9.02	4.23	10.34
Middle SDI	Both	Esophageal cancer	1999	11.64	6.46	12.96
High SDI	Male	Esophageal cancer	2000	9.67	9.40	9.88
High SDI	Female	Esophageal cancer	2000	2.18	2.05	2.26
High SDI	Both	Esophageal cancer	2000	5.57	5.38	5.69
High-middle SDI	Male	Esophageal cancer	2000	13.77	10.52	15.25

High-middle SDI	Female	Esophageal cancer	2000		4.25	2.79	4.78
High-middle SDI	Both	Esophageal cancer	2000		8.49	6.42	9.28
Low SDI	Male	Esophageal cancer	2000		5.68	4.62	6.58
Low SDI	Female	Esophageal cancer	2000		5.19	4.14	6.12
Low SDI	Both	Esophageal cancer	2000		5.45	4.53	6.17
Low-middle SDI	Male	Esophageal cancer	2000		6.19	5.58	8.30
Low-middle SDI	Female	Esophageal cancer	2000		3.68	3.18	4.89
Low-middle SDI	Both	Esophageal cancer	2000		4.93	4.48	6.28
Middle SDI	Male	Esophageal cancer	2000		14.70	8.76	16.86
Middle SDI	Female	Esophageal cancer	2000		9.21	4.20	10.60
Middle SDI	Both	Esophageal cancer	2000		11.91	6.57	13.36
High SDI	Male	Esophageal cancer	2001		9.72	9.44	9.95
High SDI	Female	Esophageal cancer	2001		2.18	2.05	2.26
High SDI	Both	Esophageal cancer	2001		5.60	5.40	5.73
High-middle SDI	Male	Esophageal cancer	2001		14.06	10.20	15.69
High-middle SDI	Female	Esophageal cancer	2001		4.31	2.79	4.86
High-middle SDI	Both	Esophageal cancer	2001		8.66	6.22	9.50
Low SDI	Male	Esophageal cancer	2001		5.67	4.65	6.57
Low SDI	Female	Esophageal cancer	2001		5.13	4.10	6.06
Low SDI	Both	Esophageal cancer	2001		5.41	4.62	6.14
Low-middle SDI	Male	Esophageal cancer	2001		6.23	5.64	8.43
Low-middle SDI	Female	Esophageal cancer	2001		3.67	3.17	4.90
Low-middle SDI	Both	Esophageal cancer	2001		4.94	4.51	6.38
Middle SDI	Male	Esophageal cancer	2001		15.00	8.60	17.14
Middle SDI	Female	Esophageal cancer	2001		9.27	4.06	10.74
Middle SDI	Both	Esophageal cancer	2001		12.09	6.41	13.59
High SDI	Male	Esophageal cancer	2002		9.80	9.51	10.04
High SDI	Female	Esophageal cancer	2002		2.19	2.06	2.28
High SDI	Both	Esophageal cancer	2002		5.64	5.45	5.78
High-middle SDI	Male	Esophageal cancer	2002		14.32	10.41	15.98
High-middle SDI	Female	Esophageal cancer	2002		4.40	2.81	5.00
High-middle SDI	Both	Esophageal cancer	2002		8.83	6.28	9.69
Low SDI	Male	Esophageal cancer	2002		5.66	4.69	6.53
Low SDI	Female	Esophageal cancer	2002		5.10	4.10	6.02
Low SDI	Both	Esophageal cancer	2002		5.39	4.58	6.15
Low-middle SDI	Male	Esophageal cancer	2002		6.25	5.68	8.37
Low-middle SDI	Female	Esophageal cancer	2002		3.66	3.19	4.86
Low-middle SDI	Both	Esophageal cancer	2002		4.95	4.50	6.41
Middle SDI	Male	Esophageal cancer	2002		15.40	8.74	17.66
Middle SDI	Female	Esophageal cancer	2002		9.47	4.15	10.93
Middle SDI	Both	Esophageal cancer	2002		12.38	6.57	13.83
High SDI	Male	Esophageal cancer	2003		9.91	9.59	10.17
High SDI	Female	Esophageal cancer	2003		2.20	2.06	2.29
High SDI	Both	Esophageal cancer	2003		5.70	5.48	5.85
High-middle SDI	Male	Esophageal cancer	2003		14.55	10.51	16.22
High-middle SDI	Female	Esophageal cancer	2003		4.49	2.76	5.08
High-middle SDI	Both	Esophageal cancer	2003		8.98	6.34	9.87
Low SDI	Male	Esophageal cancer	2003		5.69	4.70	6.50
Low SDI	Female	Esophageal cancer	2003		5.07	4.07	5.99
Low SDI	Both	Esophageal cancer	2003		5.39	4.61	6.12
Low-middle SDI	Male	Esophageal cancer	2003		6.21	5.65	8.24
Low-middle SDI	Female	Esophageal cancer	2003		3.61	3.16	4.77

Low-middle SDI	Both	Esophageal cancer	2003	4.89	4.49	6.35
Middle SDI	Male	Esophageal cancer	2003	15.66	8.88	17.87
Middle SDI	Female	Esophageal cancer	2003	9.61	4.11	11.01
Middle SDI	Both	Esophageal cancer	2003	12.58	6.64	14.06
High SDI	Male	Esophageal cancer	2004	9.79	9.48	10.04
High SDI	Female	Esophageal cancer	2004	2.17	2.03	2.27
High SDI	Both	Esophageal cancer	2004	5.64	5.42	5.79
High-middle SDI	Male	Esophageal cancer	2004	14.78	10.40	16.47
High-middle SDI	Female	Esophageal cancer	2004	4.47	2.79	5.09
High-middle SDI	Both	Esophageal cancer	2004	9.08	6.38	10.00
Low SDI	Male	Esophageal cancer	2004	5.66	4.61	6.55
Low SDI	Female	Esophageal cancer	2004	5.02	4.02	5.96
Low SDI	Both	Esophageal cancer	2004	5.35	4.60	6.08
Low-middle SDI	Male	Esophageal cancer	2004	6.12	5.61	8.29
Low-middle SDI	Female	Esophageal cancer	2004	3.50	3.10	4.71
Low-middle SDI	Both	Esophageal cancer	2004	4.79	4.41	6.18
Middle SDI	Male	Esophageal cancer	2004	15.94	8.92	18.30
Middle SDI	Female	Esophageal cancer	2004	9.53	4.12	10.95
Middle SDI	Both	Esophageal cancer	2004	12.67	6.64	14.23
High SDI	Male	Esophageal cancer	2005	9.85	9.53	10.12
High SDI	Female	Esophageal cancer	2005	2.16	2.01	2.25
High SDI	Both	Esophageal cancer	2005	5.67	5.45	5.83
High-middle SDI	Male	Esophageal cancer	2005	14.71	10.39	16.32
High-middle SDI	Female	Esophageal cancer	2005	4.33	2.71	4.95
High-middle SDI	Both	Esophageal cancer	2005	8.98	6.26	9.88
Low SDI	Male	Esophageal cancer	2005	5.65	4.65	6.55
Low SDI	Female	Esophageal cancer	2005	4.96	3.98	5.83
Low SDI	Both	Esophageal cancer	2005	5.32	4.53	6.07
Low-middle SDI	Male	Esophageal cancer	2005	6.18	5.62	8.29
Low-middle SDI	Female	Esophageal cancer	2005	3.49	3.07	4.62
Low-middle SDI	Both	Esophageal cancer	2005	4.82	4.43	6.19
Middle SDI	Male	Esophageal cancer	2005	15.50	8.76	17.54
Middle SDI	Female	Esophageal cancer	2005	9.09	3.86	10.40
Middle SDI	Both	Esophageal cancer	2005	12.22	6.42	13.66
High SDI	Male	Esophageal cancer	2006	9.76	9.43	10.04
High SDI	Female	Esophageal cancer	2006	2.13	1.98	2.23
High SDI	Both	Esophageal cancer	2006	5.62	5.39	5.79
High-middle SDI	Male	Esophageal cancer	2006	14.11	10.06	15.59
High-middle SDI	Female	Esophageal cancer	2006	4.12	2.62	4.68
High-middle SDI	Both	Esophageal cancer	2006	8.60	6.09	9.45
Low SDI	Male	Esophageal cancer	2006	5.59	4.62	6.45
Low SDI	Female	Esophageal cancer	2006	4.89	3.96	5.76
Low SDI	Both	Esophageal cancer	2006	5.25	4.50	5.99
Low-middle SDI	Male	Esophageal cancer	2006	6.11	5.60	8.30
Low-middle SDI	Female	Esophageal cancer	2006	3.43	3.02	4.61
Low-middle SDI	Both	Esophageal cancer	2006	4.75	4.38	6.17
Middle SDI	Male	Esophageal cancer	2006	14.73	8.59	16.70
Middle SDI	Female	Esophageal cancer	2006	8.48	3.82	9.68
Middle SDI	Both	Esophageal cancer	2006	11.54	6.33	12.83
High SDI	Male	Esophageal cancer	2007	9.74	9.41	10.03
High SDI	Female	Esophageal cancer	2007	2.11	1.96	2.21
High SDI	Both	Esophageal cancer	2007	5.61	5.38	5.78

High-middle SDI	Male	Esophageal cancer	2007		13.67	9.91	15.15
High-middle SDI	Female	Esophageal cancer	2007		3.95	2.56	4.51
High-middle SDI	Both	Esophageal cancer	2007		8.32	5.96	9.15
Low SDI	Male	Esophageal cancer	2007		5.55	4.63	6.40
Low SDI	Female	Esophageal cancer	2007		4.81	3.90	5.67
Low SDI	Both	Esophageal cancer	2007		5.19	4.45	5.93
Low-middle SDI	Male	Esophageal cancer	2007		6.05	5.52	8.34
Low-middle SDI	Female	Esophageal cancer	2007		3.37	2.97	4.56
Low-middle SDI	Both	Esophageal cancer	2007		4.68	4.30	6.28
Middle SDI	Male	Esophageal cancer	2007		14.20	8.64	16.09
Middle SDI	Female	Esophageal cancer	2007		7.90	3.78	8.91
Middle SDI	Both	Esophageal cancer	2007		10.97	6.28	12.17
High SDI	Male	Esophageal cancer	2008		9.74	9.38	10.03
High SDI	Female	Esophageal cancer	2008		2.11	1.95	2.21
High SDI	Both	Esophageal cancer	2008		5.61	5.37	5.79
High-middle SDI	Male	Esophageal cancer	2008		13.36	10.02	14.79
High-middle SDI	Female	Esophageal cancer	2008		3.79	2.53	4.35
High-middle SDI	Both	Esophageal cancer	2008		8.09	5.95	8.87
Low SDI	Male	Esophageal cancer	2008		5.57	4.67	6.43
Low SDI	Female	Esophageal cancer	2008		4.75	3.87	5.63
Low SDI	Both	Esophageal cancer	2008		5.17	4.47	5.91
Low-middle SDI	Male	Esophageal cancer	2008		6.05	5.51	8.47
Low-middle SDI	Female	Esophageal cancer	2008		3.30	2.91	4.56
Low-middle SDI	Both	Esophageal cancer	2008		4.65	4.26	6.26
Middle SDI	Male	Esophageal cancer	2008		13.64	8.81	15.34
Middle SDI	Female	Esophageal cancer	2008		7.36	3.69	8.27
Middle SDI	Both	Esophageal cancer	2008		10.41	6.32	11.48
High SDI	Male	Esophageal cancer	2009		9.69	9.32	10.02
High SDI	Female	Esophageal cancer	2009		2.08	1.93	2.19
High SDI	Both	Esophageal cancer	2009		5.58	5.34	5.77
High-middle SDI	Male	Esophageal cancer	2009		13.06	9.91	14.41
High-middle SDI	Female	Esophageal cancer	2009		3.65	2.45	4.19
High-middle SDI	Both	Esophageal cancer	2009		7.88	5.87	8.62
Low SDI	Male	Esophageal cancer	2009		5.55	4.65	6.44
Low SDI	Female	Esophageal cancer	2009		4.69	3.78	5.56
Low SDI	Both	Esophageal cancer	2009		5.13	4.41	5.89
Low-middle SDI	Male	Esophageal cancer	2009		5.95	5.41	8.52
Low-middle SDI	Female	Esophageal cancer	2009		3.18	2.80	4.38
Low-middle SDI	Both	Esophageal cancer	2009		4.53	4.15	6.18
Middle SDI	Male	Esophageal cancer	2009		13.15	9.13	14.66
Middle SDI	Female	Esophageal cancer	2009		6.90	3.61	7.77
Middle SDI	Both	Esophageal cancer	2009		9.92	6.32	10.92
High SDI	Male	Esophageal cancer	2010		9.61	9.23	9.92
High SDI	Female	Esophageal cancer	2010		2.06	1.90	2.17
High SDI	Both	Esophageal cancer	2010		5.54	5.29	5.73
High-middle SDI	Male	Esophageal cancer	2010		12.90	9.84	14.30
High-middle SDI	Female	Esophageal cancer	2010		3.53	2.44	4.10
High-middle SDI	Both	Esophageal cancer	2010		7.74	5.85	8.53
Low SDI	Male	Esophageal cancer	2010		5.53	4.58	6.41
Low SDI	Female	Esophageal cancer	2010		4.64	3.79	5.51
Low SDI	Both	Esophageal cancer	2010		5.09	4.39	5.85
Low-middle SDI	Male	Esophageal cancer	2010		5.89	5.35	8.52

Low-middle SDI	Female	Esophageal cancer	2010	3.14	2.75	4.28
Low-middle SDI	Both	Esophageal cancer	2010	4.48	4.07	6.26
Middle SDI	Male	Esophageal cancer	2010	12.63	9.05	14.17
Middle SDI	Female	Esophageal cancer	2010	6.47	3.60	7.32
Middle SDI	Both	Esophageal cancer	2010	9.44	6.30	10.40
High SDI	Male	Esophageal cancer	2011	9.51	9.11	9.85
High SDI	Female	Esophageal cancer	2011	2.05	1.89	2.16
High SDI	Both	Esophageal cancer	2011	5.49	5.24	5.69
High-middle SDI	Male	Esophageal cancer	2011	12.60	9.83	14.05
High-middle SDI	Female	Esophageal cancer	2011	3.39	2.38	4.00
High-middle SDI	Both	Esophageal cancer	2011	7.53	5.85	8.29
Low SDI	Male	Esophageal cancer	2011	5.52	4.60	6.39
Low SDI	Female	Esophageal cancer	2011	4.60	3.74	5.55
Low SDI	Both	Esophageal cancer	2011	5.07	4.38	5.83
Low-middle SDI	Male	Esophageal cancer	2011	5.79	5.22	8.43
Low-middle SDI	Female	Esophageal cancer	2011	3.08	2.71	4.30
Low-middle SDI	Both	Esophageal cancer	2011	4.40	4.00	6.24
Middle SDI	Male	Esophageal cancer	2011	11.96	9.05	13.32
Middle SDI	Female	Esophageal cancer	2011	6.00	3.53	6.81
Middle SDI	Both	Esophageal cancer	2011	8.88	6.28	9.73
High SDI	Male	Esophageal cancer	2012	9.36	8.98	9.70
High SDI	Female	Esophageal cancer	2012	2.02	1.86	2.13
High SDI	Both	Esophageal cancer	2012	5.41	5.16	5.60
High-middle SDI	Male	Esophageal cancer	2012	12.38	9.77	13.89
High-middle SDI	Female	Esophageal cancer	2012	3.22	2.29	3.77
High-middle SDI	Both	Esophageal cancer	2012	7.35	5.85	8.15
Low SDI	Male	Esophageal cancer	2012	5.50	4.59	6.45
Low SDI	Female	Esophageal cancer	2012	4.59	3.73	5.45
Low SDI	Both	Esophageal cancer	2012	5.05	4.34	5.79
Low-middle SDI	Male	Esophageal cancer	2012	5.75	5.19	8.69
Low-middle SDI	Female	Esophageal cancer	2012	3.01	2.64	4.25
Low-middle SDI	Both	Esophageal cancer	2012	4.34	3.95	6.25
Middle SDI	Male	Esophageal cancer	2012	11.34	9.03	12.61
Middle SDI	Female	Esophageal cancer	2012	5.52	3.46	6.18
Middle SDI	Both	Esophageal cancer	2012	8.32	6.20	9.08
High SDI	Male	Esophageal cancer	2013	9.27	8.86	9.63
High SDI	Female	Esophageal cancer	2013	2.01	1.84	2.12
High SDI	Both	Esophageal cancer	2013	5.37	5.10	5.58
High-middle SDI	Male	Esophageal cancer	2013	12.05	9.45	13.53
High-middle SDI	Female	Esophageal cancer	2013	3.11	2.23	3.62
High-middle SDI	Both	Esophageal cancer	2013	7.14	5.62	7.89
Low SDI	Male	Esophageal cancer	2013	5.52	4.55	6.44
Low SDI	Female	Esophageal cancer	2013	4.58	3.70	5.46
Low SDI	Both	Esophageal cancer	2013	5.06	4.38	5.84
Low-middle SDI	Male	Esophageal cancer	2013	5.71	5.14	8.49
Low-middle SDI	Female	Esophageal cancer	2013	3.00	2.64	4.20
Low-middle SDI	Both	Esophageal cancer	2013	4.31	3.92	6.29
Middle SDI	Male	Esophageal cancer	2013	10.69	8.75	11.97
Middle SDI	Female	Esophageal cancer	2013	5.18	3.41	5.80
Middle SDI	Both	Esophageal cancer	2013	7.82	6.14	8.56
High SDI	Male	Esophageal cancer	2014	9.12	8.72	9.49
High SDI	Female	Esophageal cancer	2014	1.99	1.83	2.10

High SDI	Both	Esophageal cancer	2014	5.29	5.03	5.51
High-middle SDI	Male	Esophageal cancer	2014	11.85	9.37	13.35
High-middle SDI	Female	Esophageal cancer	2014	3.05	2.22	3.55
High-middle SDI	Both	Esophageal cancer	2014	7.03	5.55	7.80
Low SDI	Male	Esophageal cancer	2014	5.48	4.48	6.41
Low SDI	Female	Esophageal cancer	2014	4.56	3.76	5.43
Low SDI	Both	Esophageal cancer	2014	5.03	4.31	5.81
Low-middle SDI	Male	Esophageal cancer	2014	5.65	5.05	8.58
Low-middle SDI	Female	Esophageal cancer	2014	2.99	2.64	4.18
Low-middle SDI	Both	Esophageal cancer	2014	4.27	3.86	6.22
Middle SDI	Male	Esophageal cancer	2014	10.23	8.70	11.49
Middle SDI	Female	Esophageal cancer	2014	4.95	3.32	5.56
Middle SDI	Both	Esophageal cancer	2014	7.48	6.10	8.13
High SDI	Male	Esophageal cancer	2015	9.08	8.67	9.47
High SDI	Female	Esophageal cancer	2015	1.98	1.81	2.10
High SDI	Both	Esophageal cancer	2015	5.27	5.01	5.50
High-middle SDI	Male	Esophageal cancer	2015	11.74	9.34	13.47
High-middle SDI	Female	Esophageal cancer	2015	3.00	2.17	3.52
High-middle SDI	Both	Esophageal cancer	2015	6.96	5.54	7.82
Low SDI	Male	Esophageal cancer	2015	5.45	4.49	6.44
Low SDI	Female	Esophageal cancer	2015	4.53	3.70	5.40
Low SDI	Both	Esophageal cancer	2015	5.00	4.26	5.75
Low-middle SDI	Male	Esophageal cancer	2015	5.64	5.05	8.42
Low-middle SDI	Female	Esophageal cancer	2015	3.02	2.68	4.25
Low-middle SDI	Both	Esophageal cancer	2015	4.29	3.87	6.23
Middle SDI	Male	Esophageal cancer	2015	9.87	8.50	11.18
Middle SDI	Female	Esophageal cancer	2015	4.72	3.28	5.33
Middle SDI	Both	Esophageal cancer	2015	7.18	6.03	7.89
High SDI	Male	Esophageal cancer	2016	9.01	8.60	9.42
High SDI	Female	Esophageal cancer	2016	1.97	1.81	2.09
High SDI	Both	Esophageal cancer	2016	5.24	4.98	5.48
High-middle SDI	Male	Esophageal cancer	2016	11.70	9.38	13.48
High-middle SDI	Female	Esophageal cancer	2016	3.00	2.17	3.61
High-middle SDI	Both	Esophageal cancer	2016	6.94	5.58	7.81
Low SDI	Male	Esophageal cancer	2016	5.43	4.44	6.43
Low SDI	Female	Esophageal cancer	2016	4.51	3.71	5.39
Low SDI	Both	Esophageal cancer	2016	4.97	4.23	5.76
Low-middle SDI	Male	Esophageal cancer	2016	5.66	5.04	8.33
Low-middle SDI	Female	Esophageal cancer	2016	3.03	2.68	4.28
Low-middle SDI	Both	Esophageal cancer	2016	4.30	3.88	6.13
Middle SDI	Male	Esophageal cancer	2016	9.63	8.35	11.05
Middle SDI	Female	Esophageal cancer	2016	4.58	3.18	5.25
Middle SDI	Both	Esophageal cancer	2016	6.99	5.92	7.71
High SDI	Male	Esophageal cancer	2017	8.91	8.36	9.47
High SDI	Female	Esophageal cancer	2017	1.94	1.76	2.10
High SDI	Both	Esophageal cancer	2017	5.19	4.86	5.52
High-middle SDI	Male	Esophageal cancer	2017	11.64	9.31	13.59
High-middle SDI	Female	Esophageal cancer	2017	3.02	2.16	3.66
High-middle SDI	Both	Esophageal cancer	2017	6.93	5.54	7.87
Low SDI	Male	Esophageal cancer	2017	5.41	4.44	6.51
Low SDI	Female	Esophageal cancer	2017	4.49	3.61	5.37
Low SDI	Both	Esophageal cancer	2017	4.96	4.17	5.74

Low-middle SDI	Male	Esophageal cancer	2017		5.69	5.02	8.33
Low-middle SDI	Female	Esophageal cancer	2017		3.02	2.64	4.17
Low-middle SDI	Both	Esophageal cancer	2017		4.30	3.86	6.18
Middle SDI	Male	Esophageal cancer	2017		9.56	8.12	11.14
Middle SDI	Female	Esophageal cancer	2017		4.54	3.17	5.31
Middle SDI	Both	Esophageal cancer	2017		6.93	5.96	7.75
High SDI	Male	Esophageal cancer	2018		8.93	8.15	9.77
High SDI	Female	Esophageal cancer	2018		1.94	1.73	2.13
High SDI	Both	Esophageal cancer	2018		5.20	4.75	5.66
High-middle SDI	Male	Esophageal cancer	2018		11.70	9.32	13.72
High-middle SDI	Female	Esophageal cancer	2018		3.04	2.17	3.76
High-middle SDI	Both	Esophageal cancer	2018		6.98	5.63	7.91
Low SDI	Male	Esophageal cancer	2018		5.43	4.48	6.54
Low SDI	Female	Esophageal cancer	2018		4.49	3.64	5.41
Low SDI	Both	Esophageal cancer	2018		4.97	4.21	5.80
Low-middle SDI	Male	Esophageal cancer	2018		5.74	5.02	8.55
Low-middle SDI	Female	Esophageal cancer	2018		3.02	2.63	4.22
Low-middle SDI	Both	Esophageal cancer	2018		4.32	3.85	6.13
Middle SDI	Male	Esophageal cancer	2018		9.60	8.08	11.24
Middle SDI	Female	Esophageal cancer	2018		4.55	3.16	5.41
Middle SDI	Both	Esophageal cancer	2018		6.96	5.88	7.84
High SDI	Male	Esophageal cancer	2019		8.92	8.03	9.84
High SDI	Female	Esophageal cancer	2019		1.93	1.69	2.16
High SDI	Both	Esophageal cancer	2019		5.20	4.71	5.70
High-middle SDI	Male	Esophageal cancer	2019		11.84	9.08	14.20
High-middle SDI	Female	Esophageal cancer	2019		3.06	2.18	3.84
High-middle SDI	Both	Esophageal cancer	2019		7.06	5.50	8.22
Low SDI	Male	Esophageal cancer	2019		5.43	4.38	6.54
Low SDI	Female	Esophageal cancer	2019		4.48	3.65	5.38
Low SDI	Both	Esophageal cancer	2019		4.96	4.14	5.84
Low-middle SDI	Male	Esophageal cancer	2019		5.77	5.00	8.55
Low-middle SDI	Female	Esophageal cancer	2019		3.01	2.58	4.10
Low-middle SDI	Both	Esophageal cancer	2019		4.34	3.82	6.14
Middle SDI	Male	Esophageal cancer	2019		9.72	8.01	11.46
Middle SDI	Female	Esophageal cancer	2019		4.58	3.13	5.46
Middle SDI	Both	Esophageal cancer	2019		7.02	5.81	7.99

sex_name	cause_name	year	Age-standardised death rate (per 100 000 person-years)	95% CI (lower)	95% CI (upper)
Male	Esophageal cancer	1990	11.93	9.40	13.28
Female	Esophageal cancer	1990	5.01	3.64	5.60
Both	Esophageal cancer	1990	8.18	6.40	8.97
Male	Esophageal cancer	1991	11.96	9.52	13.30
Female	Esophageal cancer	1991	5.00	3.61	5.59
Both	Esophageal cancer	1991	8.19	6.45	8.93
Male	Esophageal cancer	1992	11.94	9.39	13.26
Female	Esophageal cancer	1992	4.99	3.59	5.55
Both	Esophageal cancer	1992	8.18	6.39	8.91
Male	Esophageal cancer	1993	11.98	9.41	13.13
Female	Esophageal cancer	1993	5.01	3.55	5.56
Both	Esophageal cancer	1993	8.22	6.35	8.88
Male	Esophageal cancer	1994	11.94	9.37	13.11
Female	Esophageal cancer	1994	4.95	3.51	5.49
Both	Esophageal cancer	1994	8.17	6.41	8.84
Male	Esophageal cancer	1995	11.95	9.40	13.01
Female	Esophageal cancer	1995	4.86	3.42	5.37
Both	Esophageal cancer	1995	8.13	6.33	8.74
Male	Esophageal cancer	1996	11.91	9.33	12.90
Female	Esophageal cancer	1996	4.84	3.40	5.33
Both	Esophageal cancer	1996	8.10	6.31	8.74
Male	Esophageal cancer	1997	11.88	9.25	12.88
Female	Esophageal cancer	1997	4.82	3.33	5.29
Both	Esophageal cancer	1997	8.08	6.24	8.64
Male	Esophageal cancer	1998	11.96	9.21	12.94
Female	Esophageal cancer	1998	4.79	3.28	5.25
Both	Esophageal cancer	1998	8.09	6.18	8.68
Male	Esophageal cancer	1999	12.09	9.17	13.14
Female	Esophageal cancer	1999	4.81	3.28	5.29
Both	Esophageal cancer	1999	8.17	6.17	8.77
Male	Esophageal cancer	2000	12.33	9.33	13.44
Female	Esophageal cancer	2000	4.87	3.23	5.36
Both	Esophageal cancer	2000	8.31	6.20	8.98
Male	Esophageal cancer	2001	12.52	9.26	13.71
Female	Esophageal cancer	2001	4.88	3.20	5.40
Both	Esophageal cancer	2001	8.41	6.10	9.09
Male	Esophageal cancer	2002	12.69	9.41	13.87
Female	Esophageal cancer	2002	4.94	3.21	5.47
Both	Esophageal cancer	2002	8.52	6.17	9.21
Male	Esophageal cancer	2003	12.84	9.29	14.06
Female	Esophageal cancer	2003	4.97	3.16	5.51
Both	Esophageal cancer	2003	8.61	6.15	9.34
Male	Esophageal cancer	2004	12.96	9.28	14.20
Female	Esophageal cancer	2004	4.91	3.09	5.43
Both	Esophageal cancer	2004	8.63	6.08	9.34
Male	Esophageal cancer	2005	12.84	9.33	14.04
Female	Esophageal cancer	2005	4.74	3.07	5.22
Both	Esophageal cancer	2005	8.49	6.09	9.19
Male	Esophageal cancer	2006	12.38	9.17	13.43

Female	Esophageal cancer	2006	4.50	3.00	4.92
Both	Esophageal cancer	2006	8.15	5.97	8.76
Male	Esophageal cancer	2007	12.01	9.06	13.04
Female	Esophageal cancer	2007	4.27	2.92	4.64
Both	Esophageal cancer	2007	7.85	5.91	8.45
Male	Esophageal cancer	2008	11.73	9.11	12.69
Female	Esophageal cancer	2008	4.06	2.89	4.41
Both	Esophageal cancer	2008	7.61	5.85	8.17
Male	Esophageal cancer	2009	11.44	9.02	12.35
Female	Esophageal cancer	2009	3.86	2.81	4.20
Both	Esophageal cancer	2009	7.37	5.84	7.87
Male	Esophageal cancer	2010	11.20	9.08	12.09
Female	Esophageal cancer	2010	3.70	2.79	4.03
Both	Esophageal cancer	2010	7.17	5.78	7.68
Male	Esophageal cancer	2011	10.87	9.14	11.78
Female	Esophageal cancer	2011	3.54	2.75	3.86
Both	Esophageal cancer	2011	6.93	5.84	7.44
Male	Esophageal cancer	2012	10.58	9.16	11.46
Female	Esophageal cancer	2012	3.36	2.69	3.65
Both	Esophageal cancer	2012	6.70	5.78	7.16
Male	Esophageal cancer	2013	10.25	8.91	11.15
Female	Esophageal cancer	2013	3.24	2.65	3.52
Both	Esophageal cancer	2013	6.49	5.66	6.92
Male	Esophageal cancer	2014	9.99	8.81	10.89
Female	Esophageal cancer	2014	3.15	2.65	3.43
Both	Esophageal cancer	2014	6.32	5.57	6.80
Male	Esophageal cancer	2015	9.82	8.65	10.87
Female	Esophageal cancer	2015	3.09	2.62	3.37
Both	Esophageal cancer	2015	6.20	5.54	6.71
Male	Esophageal cancer	2016	9.70	8.57	10.73
Female	Esophageal cancer	2016	3.05	2.60	3.36
Both	Esophageal cancer	2016	6.13	5.47	6.66
Male	Esophageal cancer	2017	9.61	8.48	10.63
Female	Esophageal cancer	2017	3.02	2.60	3.34
Both	Esophageal cancer	2017	6.07	5.37	6.58
Male	Esophageal cancer	2018	9.62	8.42	10.85
Female	Esophageal cancer	2018	3.02	2.52	3.38
Both	Esophageal cancer	2018	6.08	5.42	6.69
Male	Esophageal cancer	2019	9.68	8.34	10.96
Female	Esophageal cancer	2019	3.02	2.52	3.43
Both	Esophageal cancer	2019	6.11	5.38	6.76

location_name	sex_name	cause_name	year	Age-standardised death rate (per 100 000 person-years)	95% CI (lower)	95% CI (upper)
High SDI	Male	Esophageal cancer	1990	8.12	7.90	8.28
High SDI	Female	Esophageal cancer	1990	1.86	1.74	1.92
High SDI	Both	Esophageal cancer	1990	4.59	4.45	4.68
High-middle SDI	Male	Esophageal cancer	1990	13.56	11.23	15.21
High-middle SDI	Female	Esophageal cancer	1990	4.40	3.48	4.92
High-middle SDI	Both	Esophageal cancer	1990	8.34	6.98	9.14
Low SDI	Male	Esophageal cancer	1990	7.14	5.55	8.48
Low SDI	Female	Esophageal cancer	1990	5.58	4.46	6.64
Low SDI	Both	Esophageal cancer	1990	6.38	5.24	7.31
Low-middle SDI	Male	Esophageal cancer	1990	6.57	5.80	8.58
Low-middle SDI	Female	Esophageal cancer	1990	4.09	3.42	5.66
Low-middle SDI	Both	Esophageal cancer	1990	5.34	4.75	6.86
Middle SDI	Male	Esophageal cancer	1990	18.49	11.25	21.92
Middle SDI	Female	Esophageal cancer	1990	10.03	5.28	11.79
Middle SDI	Both	Esophageal cancer	1990	14.13	8.38	16.22
High SDI	Male	Esophageal cancer	1991	8.16	7.94	8.30
High SDI	Female	Esophageal cancer	1991	1.85	1.74	1.91
High SDI	Both	Esophageal cancer	1991	4.61	4.46	4.69
High-middle SDI	Male	Esophageal cancer	1991	13.48	11.42	15.03
High-middle SDI	Female	Esophageal cancer	1991	4.33	3.46	4.86
High-middle SDI	Both	Esophageal cancer	1991	8.28	7.01	9.06
Low SDI	Male	Esophageal cancer	1991	7.15	5.54	8.41
Low SDI	Female	Esophageal cancer	1991	5.57	4.44	6.62
Low SDI	Both	Esophageal cancer	1991	6.38	5.38	7.30
Low-middle SDI	Male	Esophageal cancer	1991	6.54	5.80	8.70
Low-middle SDI	Female	Esophageal cancer	1991	4.04	3.39	5.66
Low-middle SDI	Both	Esophageal cancer	1991	5.30	4.74	6.79
Middle SDI	Male	Esophageal cancer	1991	18.61	11.53	21.96
Middle SDI	Female	Esophageal cancer	1991	10.05	5.18	11.76
Middle SDI	Both	Esophageal cancer	1991	14.20	8.46	16.19
High SDI	Male	Esophageal cancer	1992	8.20	7.97	8.35
High SDI	Female	Esophageal cancer	1992	1.85	1.74	1.91
High SDI	Both	Esophageal cancer	1992	4.64	4.48	4.72
High-middle SDI	Male	Esophageal cancer	1992	13.47	11.24	14.94
High-middle SDI	Female	Esophageal cancer	1992	4.32	3.42	4.83
High-middle SDI	Both	Esophageal cancer	1992	8.28	6.99	9.03
Low SDI	Male	Esophageal cancer	1992	7.13	5.49	8.38
Low SDI	Female	Esophageal cancer	1992	5.56	4.44	6.60
Low SDI	Both	Esophageal cancer	1992	6.37	5.27	7.25
Low-middle SDI	Male	Esophageal cancer	1992	6.50	5.77	8.55
Low-middle SDI	Female	Esophageal cancer	1992	4.03	3.37	5.62
Low-middle SDI	Both	Esophageal cancer	1992	5.28	4.73	6.91
Middle SDI	Male	Esophageal cancer	1992	18.48	11.17	21.78
Middle SDI	Female	Esophageal cancer	1992	10.00	5.17	11.65
Middle SDI	Both	Esophageal cancer	1992	14.11	8.22	16.15
High SDI	Male	Esophageal cancer	1993	8.28	8.05	8.43
High SDI	Female	Esophageal cancer	1993	1.86	1.74	1.92
High SDI	Both	Esophageal cancer	1993	4.68	4.52	4.77
High-middle SDI	Male	Esophageal cancer	1993	13.57	11.41	14.98

High-middle SDI	Female	Esophageal cancer	1993	4.36	3.43	4.84
High-middle SDI	Both	Esophageal cancer	1993	8.36	7.01	9.06
Low SDI	Male	Esophageal cancer	1993	7.12	5.61	8.36
Low SDI	Female	Esophageal cancer	1993	5.54	4.42	6.57
Low SDI	Both	Esophageal cancer	1993	6.35	5.29	7.26
Low-middle SDI	Male	Esophageal cancer	1993	6.50	5.85	8.63
Low-middle SDI	Female	Esophageal cancer	1993	4.00	3.38	5.55
Low-middle SDI	Both	Esophageal cancer	1993	5.26	4.73	6.80
Middle SDI	Male	Esophageal cancer	1993	18.43	11.07	21.43
Middle SDI	Female	Esophageal cancer	1993	9.98	5.15	11.55
Middle SDI	Both	Esophageal cancer	1993	14.08	8.16	15.96
High SDI	Male	Esophageal cancer	1994	8.29	8.06	8.43
High SDI	Female	Esophageal cancer	1994	1.85	1.74	1.91
High SDI	Both	Esophageal cancer	1994	4.69	4.53	4.77
High-middle SDI	Male	Esophageal cancer	1994	13.59	11.32	14.92
High-middle SDI	Female	Esophageal cancer	1994	4.31	3.34	4.79
High-middle SDI	Both	Esophageal cancer	1994	8.35	7.01	9.04
Low SDI	Male	Esophageal cancer	1994	7.11	5.58	8.31
Low SDI	Female	Esophageal cancer	1994	5.52	4.44	6.53
Low SDI	Both	Esophageal cancer	1994	6.34	5.32	7.23
Low-middle SDI	Male	Esophageal cancer	1994	6.49	5.83	8.71
Low-middle SDI	Female	Esophageal cancer	1994	3.96	3.35	5.48
Low-middle SDI	Both	Esophageal cancer	1994	5.23	4.74	6.76
Middle SDI	Male	Esophageal cancer	1994	18.20	11.11	21.14
Middle SDI	Female	Esophageal cancer	1994	9.77	4.88	11.35
Middle SDI	Both	Esophageal cancer	1994	13.86	8.23	15.76
High SDI	Male	Esophageal cancer	1995	8.31	8.08	8.47
High SDI	Female	Esophageal cancer	1995	1.85	1.73	1.91
High SDI	Both	Esophageal cancer	1995	4.70	4.54	4.79
High-middle SDI	Male	Esophageal cancer	1995	13.54	11.24	14.86
High-middle SDI	Female	Esophageal cancer	1995	4.22	3.23	4.70
High-middle SDI	Both	Esophageal cancer	1995	8.28	6.93	8.95
Low SDI	Male	Esophageal cancer	1995	7.15	5.60	8.36
Low SDI	Female	Esophageal cancer	1995	5.54	4.46	6.56
Low SDI	Both	Esophageal cancer	1995	6.36	5.30	7.24
Low-middle SDI	Male	Esophageal cancer	1995	6.47	5.84	8.55
Low-middle SDI	Female	Esophageal cancer	1995	3.92	3.34	5.34
Low-middle SDI	Both	Esophageal cancer	1995	5.19	4.71	6.79
Middle SDI	Male	Esophageal cancer	1995	18.20	11.18	20.94
Middle SDI	Female	Esophageal cancer	1995	9.51	4.84	11.02
Middle SDI	Both	Esophageal cancer	1995	13.72	8.02	15.42
High SDI	Male	Esophageal cancer	1996	8.28	8.03	8.43
High SDI	Female	Esophageal cancer	1996	1.84	1.72	1.91
High SDI	Both	Esophageal cancer	1996	4.69	4.52	4.78
High-middle SDI	Male	Esophageal cancer	1996	13.44	10.92	14.74
High-middle SDI	Female	Esophageal cancer	1996	4.18	3.16	4.69
High-middle SDI	Both	Esophageal cancer	1996	8.22	6.70	8.89
Low SDI	Male	Esophageal cancer	1996	7.17	5.61	8.39
Low SDI	Female	Esophageal cancer	1996	5.55	4.41	6.57
Low SDI	Both	Esophageal cancer	1996	6.38	5.22	7.28
Low-middle SDI	Male	Esophageal cancer	1996	6.50	5.85	8.71

Low-middle SDI	Female	Esophageal cancer	1996	3.91	3.33	5.34
Low-middle SDI	Both	Esophageal cancer	1996	5.20	4.71	6.74
Middle SDI	Male	Esophageal cancer	1996	18.10	11.11	20.64
Middle SDI	Female	Esophageal cancer	1996	9.42	4.68	10.80
Middle SDI	Both	Esophageal cancer	1996	13.63	8.10	15.33
High SDI	Male	Esophageal cancer	1997	8.23	7.98	8.38
High SDI	Female	Esophageal cancer	1997	1.83	1.71	1.90
High SDI	Both	Esophageal cancer	1997	4.66	4.49	4.75
High-middle SDI	Male	Esophageal cancer	1997	13.42	10.59	14.75
High-middle SDI	Female	Esophageal cancer	1997	4.15	3.02	4.62
High-middle SDI	Both	Esophageal cancer	1997	8.19	6.49	8.85
Low SDI	Male	Esophageal cancer	1997	7.20	5.68	8.35
Low SDI	Female	Esophageal cancer	1997	5.55	4.44	6.52
Low SDI	Both	Esophageal cancer	1997	6.39	5.29	7.25
Low-middle SDI	Male	Esophageal cancer	1997	6.60	5.96	8.78
Low-middle SDI	Female	Esophageal cancer	1997	3.96	3.38	5.39
Low-middle SDI	Both	Esophageal cancer	1997	5.28	4.77	6.83
Middle SDI	Male	Esophageal cancer	1997	17.97	10.93	20.36
Middle SDI	Female	Esophageal cancer	1997	9.31	4.59	10.65
Middle SDI	Both	Esophageal cancer	1997	13.50	7.97	14.95
High SDI	Male	Esophageal cancer	1998	8.26	8.01	8.42
High SDI	Female	Esophageal cancer	1998	1.83	1.71	1.89
High SDI	Both	Esophageal cancer	1998	4.68	4.51	4.78
High-middle SDI	Male	Esophageal cancer	1998	13.49	10.56	14.78
High-middle SDI	Female	Esophageal cancer	1998	4.12	2.93	4.59
High-middle SDI	Both	Esophageal cancer	1998	8.21	6.36	8.88
Low SDI	Male	Esophageal cancer	1998	7.23	5.72	8.39
Low SDI	Female	Esophageal cancer	1998	5.57	4.45	6.57
Low SDI	Both	Esophageal cancer	1998	6.41	5.31	7.27
Low-middle SDI	Male	Esophageal cancer	1998	6.62	5.99	8.87
Low-middle SDI	Female	Esophageal cancer	1998	3.94	3.36	5.28
Low-middle SDI	Both	Esophageal cancer	1998	5.27	4.77	6.97
Middle SDI	Male	Esophageal cancer	1998	18.10	10.95	20.57
Middle SDI	Female	Esophageal cancer	1998	9.16	4.47	10.53
Middle SDI	Both	Esophageal cancer	1998	13.47	7.85	15.01
High SDI	Male	Esophageal cancer	1999	8.29	8.02	8.48
High SDI	Female	Esophageal cancer	1999	1.83	1.71	1.90
High SDI	Both	Esophageal cancer	1999	4.70	4.51	4.81
High-middle SDI	Male	Esophageal cancer	1999	13.72	10.59	15.06
High-middle SDI	Female	Esophageal cancer	1999	4.15	2.92	4.60
High-middle SDI	Both	Esophageal cancer	1999	8.33	6.38	9.04
Low SDI	Male	Esophageal cancer	1999	7.19	5.67	8.33
Low SDI	Female	Esophageal cancer	1999	5.53	4.44	6.57
Low SDI	Both	Esophageal cancer	1999	6.38	5.22	7.22
Low-middle SDI	Male	Esophageal cancer	1999	6.55	5.92	8.75
Low-middle SDI	Female	Esophageal cancer	1999	3.90	3.35	5.22
Low-middle SDI	Both	Esophageal cancer	1999	5.22	4.74	6.68
Middle SDI	Male	Esophageal cancer	1999	18.32	10.83	20.75
Middle SDI	Female	Esophageal cancer	1999	9.19	4.45	10.54
Middle SDI	Both	Esophageal cancer	1999	13.60	7.69	15.18
High SDI	Male	Esophageal cancer	2000	8.27	8.00	8.46

High SDI	Female	Esophageal cancer	2000	1.82	1.69	1.89
High SDI	Both	Esophageal cancer	2000	4.69	4.51	4.80
High-middle SDI	Male	Esophageal cancer	2000	14.15	10.71	15.68
High-middle SDI	Female	Esophageal cancer	2000	4.23	2.87	4.70
High-middle SDI	Both	Esophageal cancer	2000	8.58	6.50	9.34
Low SDI	Male	Esophageal cancer	2000	7.19	5.68	8.29
Low SDI	Female	Esophageal cancer	2000	5.50	4.44	6.49
Low SDI	Both	Esophageal cancer	2000	6.36	5.29	7.22
Low-middle SDI	Male	Esophageal cancer	2000	6.57	5.94	8.71
Low-middle SDI	Female	Esophageal cancer	2000	3.89	3.37	5.20
Low-middle SDI	Both	Esophageal cancer	2000	5.22	4.74	6.74
Middle SDI	Male	Esophageal cancer	2000	18.75	11.04	21.40
Middle SDI	Female	Esophageal cancer	2000	9.36	4.31	10.74
Middle SDI	Both	Esophageal cancer	2000	13.90	7.80	15.55
High SDI	Male	Esophageal cancer	2001	8.26	7.99	8.46
High SDI	Female	Esophageal cancer	2001	1.81	1.68	1.88
High SDI	Both	Esophageal cancer	2001	4.68	4.50	4.80
High-middle SDI	Male	Esophageal cancer	2001	14.42	10.47	16.05
High-middle SDI	Female	Esophageal cancer	2001	4.25	2.85	4.81
High-middle SDI	Both	Esophageal cancer	2001	8.72	6.36	9.57
Low SDI	Male	Esophageal cancer	2001	7.15	5.65	8.27
Low SDI	Female	Esophageal cancer	2001	5.44	4.35	6.40
Low SDI	Both	Esophageal cancer	2001	6.30	5.24	7.13
Low-middle SDI	Male	Esophageal cancer	2001	6.62	5.99	8.80
Low-middle SDI	Female	Esophageal cancer	2001	3.88	3.34	5.21
Low-middle SDI	Both	Esophageal cancer	2001	5.23	4.76	6.80
Middle SDI	Male	Esophageal cancer	2001	19.16	11.05	21.89
Middle SDI	Female	Esophageal cancer	2001	9.39	4.25	10.80
Middle SDI	Both	Esophageal cancer	2001	14.10	7.60	15.79
High SDI	Male	Esophageal cancer	2002	8.26	7.98	8.48
High SDI	Female	Esophageal cancer	2002	1.80	1.68	1.88
High SDI	Both	Esophageal cancer	2002	4.69	4.50	4.82
High-middle SDI	Male	Esophageal cancer	2002	14.63	10.67	16.29
High-middle SDI	Female	Esophageal cancer	2002	4.30	2.79	4.81
High-middle SDI	Both	Esophageal cancer	2002	8.85	6.37	9.71
Low SDI	Male	Esophageal cancer	2002	7.13	5.70	8.24
Low SDI	Female	Esophageal cancer	2002	5.41	4.29	6.40
Low SDI	Both	Esophageal cancer	2002	6.28	5.28	7.14
Low-middle SDI	Male	Esophageal cancer	2002	6.64	6.03	8.84
Low-middle SDI	Female	Esophageal cancer	2002	3.87	3.36	5.15
Low-middle SDI	Both	Esophageal cancer	2002	5.24	4.78	6.80
Middle SDI	Male	Esophageal cancer	2002	19.49	11.06	22.25
Middle SDI	Female	Esophageal cancer	2002	9.54	4.21	11.03
Middle SDI	Both	Esophageal cancer	2002	14.34	7.72	16.11
High SDI	Male	Esophageal cancer	2003	8.24	7.93	8.48
High SDI	Female	Esophageal cancer	2003	1.79	1.66	1.87
High SDI	Both	Esophageal cancer	2003	4.68	4.48	4.82
High-middle SDI	Male	Esophageal cancer	2003	14.82	10.56	16.44
High-middle SDI	Female	Esophageal cancer	2003	4.33	2.79	4.87
High-middle SDI	Both	Esophageal cancer	2003	8.95	6.34	9.85
Low SDI	Male	Esophageal cancer	2003	7.14	5.79	8.23

Low SDI	Female	Esophageal cancer	2003		5.37	4.33	6.36
Low SDI	Both	Esophageal cancer	2003		6.27	5.29	7.10
Low-middle SDI	Male	Esophageal cancer	2003		6.59	6.01	8.76
Low-middle SDI	Female	Esophageal cancer	2003		3.81	3.32	5.16
Low-middle SDI	Both	Esophageal cancer	2003		5.18	4.74	6.68
Middle SDI	Male	Esophageal cancer	2003		19.85	11.02	22.67
Middle SDI	Female	Esophageal cancer	2003		9.64	4.14	11.16
Middle SDI	Both	Esophageal cancer	2003		14.57	7.70	16.34
High SDI	Male	Esophageal cancer	2004		8.14	7.85	8.36
High SDI	Female	Esophageal cancer	2004		1.76	1.63	1.84
High SDI	Both	Esophageal cancer	2004		4.62	4.43	4.75
High-middle SDI	Male	Esophageal cancer	2004		15.00	10.60	16.69
High-middle SDI	Female	Esophageal cancer	2004		4.27	2.74	4.82
High-middle SDI	Both	Esophageal cancer	2004		9.01	6.33	9.89
Low SDI	Male	Esophageal cancer	2004		7.10	5.65	8.20
Low SDI	Female	Esophageal cancer	2004		5.32	4.26	6.26
Low SDI	Both	Esophageal cancer	2004		6.22	5.31	7.06
Low-middle SDI	Male	Esophageal cancer	2004		6.49	5.88	8.78
Low-middle SDI	Female	Esophageal cancer	2004		3.70	3.25	4.96
Low-middle SDI	Both	Esophageal cancer	2004		5.07	4.66	6.50
Middle SDI	Male	Esophageal cancer	2004		20.22	11.07	23.15
Middle SDI	Female	Esophageal cancer	2004		9.51	4.05	10.98
Middle SDI	Both	Esophageal cancer	2004		14.67	7.71	16.45
High SDI	Male	Esophageal cancer	2005		8.13	7.83	8.37
High SDI	Female	Esophageal cancer	2005		1.74	1.61	1.82
High SDI	Both	Esophageal cancer	2005		4.61	4.41	4.75
High-middle SDI	Male	Esophageal cancer	2005		14.89	10.55	16.47
High-middle SDI	Female	Esophageal cancer	2005		4.11	2.65	4.64
High-middle SDI	Both	Esophageal cancer	2005		8.87	6.29	9.73
Low SDI	Male	Esophageal cancer	2005		7.08	5.69	8.14
Low SDI	Female	Esophageal cancer	2005		5.27	4.19	6.21
Low SDI	Both	Esophageal cancer	2005		6.18	5.19	7.06
Low-middle SDI	Male	Esophageal cancer	2005		6.56	5.99	8.82
Low-middle SDI	Female	Esophageal cancer	2005		3.70	3.25	4.96
Low-middle SDI	Both	Esophageal cancer	2005		5.10	4.68	6.56
Middle SDI	Male	Esophageal cancer	2005		19.88	11.04	22.60
Middle SDI	Female	Esophageal cancer	2005		9.04	4.01	10.33
Middle SDI	Both	Esophageal cancer	2005		14.26	7.64	15.91
High SDI	Male	Esophageal cancer	2006		8.04	7.73	8.27
High SDI	Female	Esophageal cancer	2006		1.71	1.57	1.79
High SDI	Both	Esophageal cancer	2006		4.56	4.36	4.70
High-middle SDI	Male	Esophageal cancer	2006		14.22	10.23	15.78
High-middle SDI	Female	Esophageal cancer	2006		3.88	2.57	4.36
High-middle SDI	Both	Esophageal cancer	2006		8.45	6.04	9.28
Low SDI	Male	Esophageal cancer	2006		7.00	5.61	8.06
Low SDI	Female	Esophageal cancer	2006		5.20	4.18	6.10
Low SDI	Both	Esophageal cancer	2006		6.11	5.19	6.96
Low-middle SDI	Male	Esophageal cancer	2006		6.49	5.91	8.87
Low-middle SDI	Female	Esophageal cancer	2006		3.64	3.21	4.91
Low-middle SDI	Both	Esophageal cancer	2006		5.03	4.61	6.54
Middle SDI	Male	Esophageal cancer	2006		18.93	10.91	21.45

Middle SDI	Female	Esophageal cancer	2006	8.39	3.87	9.52
Middle SDI	Both	Esophageal cancer	2006	13.46	7.33	14.95
High SDI	Male	Esophageal cancer	2007	7.98	7.67	8.22
High SDI	Female	Esophageal cancer	2007	1.69	1.55	1.77
High SDI	Both	Esophageal cancer	2007	4.53	4.32	4.67
High-middle SDI	Male	Esophageal cancer	2007	13.72	10.00	15.12
High-middle SDI	Female	Esophageal cancer	2007	3.68	2.45	4.14
High-middle SDI	Both	Esophageal cancer	2007	8.13	5.91	8.89
Low SDI	Male	Esophageal cancer	2007	6.94	5.67	8.07
Low SDI	Female	Esophageal cancer	2007	5.12	4.14	6.00
Low SDI	Both	Esophageal cancer	2007	6.04	5.16	6.89
Low-middle SDI	Male	Esophageal cancer	2007	6.41	5.87	8.82
Low-middle SDI	Female	Esophageal cancer	2007	3.57	3.14	4.85
Low-middle SDI	Both	Esophageal cancer	2007	4.96	4.55	6.55
Middle SDI	Male	Esophageal cancer	2007	18.12	10.78	20.49
Middle SDI	Female	Esophageal cancer	2007	7.76	3.70	8.70
Middle SDI	Both	Esophageal cancer	2007	12.74	7.29	14.12
High SDI	Male	Esophageal cancer	2008	7.94	7.61	8.19
High SDI	Female	Esophageal cancer	2008	1.67	1.53	1.75
High SDI	Both	Esophageal cancer	2008	4.50	4.30	4.65
High-middle SDI	Male	Esophageal cancer	2008	13.37	10.03	14.71
High-middle SDI	Female	Esophageal cancer	2008	3.50	2.35	4.00
High-middle SDI	Both	Esophageal cancer	2008	7.87	5.83	8.59
Low SDI	Male	Esophageal cancer	2008	6.89	5.55	8.02
Low SDI	Female	Esophageal cancer	2008	5.06	4.10	5.99
Low SDI	Both	Esophageal cancer	2008	5.98	5.14	6.82
Low-middle SDI	Male	Esophageal cancer	2008	6.41	5.83	8.89
Low-middle SDI	Female	Esophageal cancer	2008	3.50	3.07	4.82
Low-middle SDI	Both	Esophageal cancer	2008	4.92	4.50	6.57
Middle SDI	Male	Esophageal cancer	2008	17.49	10.87	19.72
Middle SDI	Female	Esophageal cancer	2008	7.18	3.64	8.05
Middle SDI	Both	Esophageal cancer	2008	12.12	7.22	13.42
High SDI	Male	Esophageal cancer	2009	7.85	7.52	8.10
High SDI	Female	Esophageal cancer	2009	1.64	1.50	1.72
High SDI	Both	Esophageal cancer	2009	4.46	4.24	4.61
High-middle SDI	Male	Esophageal cancer	2009	13.01	9.79	14.35
High-middle SDI	Female	Esophageal cancer	2009	3.34	2.33	3.84
High-middle SDI	Both	Esophageal cancer	2009	7.62	5.76	8.35
Low SDI	Male	Esophageal cancer	2009	6.85	5.56	8.01
Low SDI	Female	Esophageal cancer	2009	5.00	4.04	5.96
Low SDI	Both	Esophageal cancer	2009	5.92	5.06	6.77
Low-middle SDI	Male	Esophageal cancer	2009	6.29	5.71	8.95
Low-middle SDI	Female	Esophageal cancer	2009	3.36	2.95	4.69
Low-middle SDI	Both	Esophageal cancer	2009	4.79	4.38	6.54
Middle SDI	Male	Esophageal cancer	2009	16.94	10.97	19.06
Middle SDI	Female	Esophageal cancer	2009	6.67	3.55	7.44
Middle SDI	Both	Esophageal cancer	2009	11.58	7.32	12.79
High SDI	Male	Esophageal cancer	2010	7.78	7.45	8.03
High SDI	Female	Esophageal cancer	2010	1.62	1.48	1.71
High SDI	Both	Esophageal cancer	2010	4.42	4.20	4.57
High-middle SDI	Male	Esophageal cancer	2010	12.80	9.82	14.18

High-middle SDI	Female	Esophageal cancer	2010	3.21	2.27	3.67
High-middle SDI	Both	Esophageal cancer	2010	7.46	5.67	8.19
Low SDI	Male	Esophageal cancer	2010	6.81	5.58	7.97
Low SDI	Female	Esophageal cancer	2010	4.94	4.02	5.87
Low SDI	Both	Esophageal cancer	2010	5.88	5.03	6.75
Low-middle SDI	Male	Esophageal cancer	2010	6.22	5.63	8.91
Low-middle SDI	Female	Esophageal cancer	2010	3.31	2.90	4.61
Low-middle SDI	Both	Esophageal cancer	2010	4.72	4.29	6.53
Middle SDI	Male	Esophageal cancer	2010	16.37	10.96	18.34
Middle SDI	Female	Esophageal cancer	2010	6.21	3.55	6.98
Middle SDI	Both	Esophageal cancer	2010	11.06	7.23	12.21
High SDI	Male	Esophageal cancer	2011	7.71	7.37	7.99
High SDI	Female	Esophageal cancer	2011	1.60	1.47	1.69
High SDI	Both	Esophageal cancer	2011	4.38	4.16	4.54
High-middle SDI	Male	Esophageal cancer	2011	12.47	9.88	13.82
High-middle SDI	Female	Esophageal cancer	2011	3.07	2.21	3.56
High-middle SDI	Both	Esophageal cancer	2011	7.24	5.72	7.98
Low SDI	Male	Esophageal cancer	2011	6.79	5.61	8.05
Low SDI	Female	Esophageal cancer	2011	4.90	3.91	5.82
Low SDI	Both	Esophageal cancer	2011	5.84	4.97	6.77
Low-middle SDI	Male	Esophageal cancer	2011	6.10	5.51	8.91
Low-middle SDI	Female	Esophageal cancer	2011	3.25	2.86	4.56
Low-middle SDI	Both	Esophageal cancer	2011	4.63	4.21	6.57
Middle SDI	Male	Esophageal cancer	2011	15.58	11.27	17.49
Middle SDI	Female	Esophageal cancer	2011	5.75	3.46	6.47
Middle SDI	Both	Esophageal cancer	2011	10.43	7.44	11.49
High SDI	Male	Esophageal cancer	2012	7.61	7.28	7.89
High SDI	Female	Esophageal cancer	2012	1.59	1.45	1.68
High SDI	Both	Esophageal cancer	2012	4.34	4.11	4.49
High-middle SDI	Male	Esophageal cancer	2012	12.21	9.80	13.60
High-middle SDI	Female	Esophageal cancer	2012	2.91	2.11	3.41
High-middle SDI	Both	Esophageal cancer	2012	7.05	5.61	7.76
Low SDI	Male	Esophageal cancer	2012	6.76	5.54	7.91
Low SDI	Female	Esophageal cancer	2012	4.88	3.99	5.87
Low SDI	Both	Esophageal cancer	2012	5.82	4.98	6.74
Low-middle SDI	Male	Esophageal cancer	2012	6.04	5.45	8.94
Low-middle SDI	Female	Esophageal cancer	2012	3.17	2.79	4.53
Low-middle SDI	Both	Esophageal cancer	2012	4.56	4.14	6.40
Middle SDI	Male	Esophageal cancer	2012	14.88	11.10	16.64
Middle SDI	Female	Esophageal cancer	2012	5.27	3.33	5.88
Middle SDI	Both	Esophageal cancer	2012	9.84	7.23	10.82
High SDI	Male	Esophageal cancer	2013	7.52	7.17	7.80
High SDI	Female	Esophageal cancer	2013	1.57	1.43	1.66
High SDI	Both	Esophageal cancer	2013	4.29	4.06	4.45
High-middle SDI	Male	Esophageal cancer	2013	11.86	9.51	13.22
High-middle SDI	Female	Esophageal cancer	2013	2.80	2.07	3.23
High-middle SDI	Both	Esophageal cancer	2013	6.83	5.52	7.53
Low SDI	Male	Esophageal cancer	2013	6.76	5.54	8.04
Low SDI	Female	Esophageal cancer	2013	4.87	3.93	5.80
Low SDI	Both	Esophageal cancer	2013	5.82	4.91	6.73
Low-middle SDI	Male	Esophageal cancer	2013	6.01	5.40	9.00

Low-middle SDI	Female	Esophageal cancer	2013	3.17	2.80	4.48
Low-middle SDI	Both	Esophageal cancer	2013	4.54	4.11	6.52
Middle SDI	Male	Esophageal cancer	2013	14.14	10.89	15.88
Middle SDI	Female	Esophageal cancer	2013	4.93	3.27	5.51
Middle SDI	Both	Esophageal cancer	2013	9.30	7.02	10.20
High SDI	Male	Esophageal cancer	2014	7.41	7.07	7.69
High SDI	Female	Esophageal cancer	2014	1.55	1.41	1.65
High SDI	Both	Esophageal cancer	2014	4.24	4.02	4.40
High-middle SDI	Male	Esophageal cancer	2014	11.63	9.36	13.09
High-middle SDI	Female	Esophageal cancer	2014	2.72	2.06	3.14
High-middle SDI	Both	Esophageal cancer	2014	6.69	5.40	7.42
Low SDI	Male	Esophageal cancer	2014	6.70	5.47	8.04
Low SDI	Female	Esophageal cancer	2014	4.85	3.93	5.78
Low SDI	Both	Esophageal cancer	2014	5.77	4.92	6.68
Low-middle SDI	Male	Esophageal cancer	2014	5.95	5.34	8.89
Low-middle SDI	Female	Esophageal cancer	2014	3.15	2.75	4.42
Low-middle SDI	Both	Esophageal cancer	2014	4.49	4.07	6.47
Middle SDI	Male	Esophageal cancer	2014	13.58	10.98	15.27
Middle SDI	Female	Esophageal cancer	2014	4.67	3.22	5.21
Middle SDI	Both	Esophageal cancer	2014	8.89	6.95	9.79
High SDI	Male	Esophageal cancer	2015	7.37	7.02	7.67
High SDI	Female	Esophageal cancer	2015	1.54	1.40	1.64
High SDI	Both	Esophageal cancer	2015	4.22	3.99	4.39
High-middle SDI	Male	Esophageal cancer	2015	11.49	9.23	13.05
High-middle SDI	Female	Esophageal cancer	2015	2.67	2.01	3.11
High-middle SDI	Both	Esophageal cancer	2015	6.61	5.28	7.36
Low SDI	Male	Esophageal cancer	2015	6.66	5.38	8.02
Low SDI	Female	Esophageal cancer	2015	4.82	3.88	5.79
Low SDI	Both	Esophageal cancer	2015	5.74	4.83	6.65
Low-middle SDI	Male	Esophageal cancer	2015	5.93	5.31	8.90
Low-middle SDI	Female	Esophageal cancer	2015	3.18	2.79	4.49
Low-middle SDI	Both	Esophageal cancer	2015	4.50	4.07	6.53
Middle SDI	Male	Esophageal cancer	2015	13.11	10.55	14.91
Middle SDI	Female	Esophageal cancer	2015	4.45	3.11	5.02
Middle SDI	Both	Esophageal cancer	2015	8.54	6.83	9.54
High SDI	Male	Esophageal cancer	2016	7.32	6.96	7.61
High SDI	Female	Esophageal cancer	2016	1.53	1.39	1.63
High SDI	Both	Esophageal cancer	2016	4.20	3.97	4.37
High-middle SDI	Male	Esophageal cancer	2016	11.43	9.25	13.01
High-middle SDI	Female	Esophageal cancer	2016	2.67	1.98	3.12
High-middle SDI	Both	Esophageal cancer	2016	6.58	5.35	7.38
Low SDI	Male	Esophageal cancer	2016	6.62	5.35	8.06
Low SDI	Female	Esophageal cancer	2016	4.79	3.90	5.75
Low SDI	Both	Esophageal cancer	2016	5.70	4.81	6.68
Low-middle SDI	Male	Esophageal cancer	2016	5.95	5.27	8.98
Low-middle SDI	Female	Esophageal cancer	2016	3.18	2.79	4.41
Low-middle SDI	Both	Esophageal cancer	2016	4.51	4.04	6.49
Middle SDI	Male	Esophageal cancer	2016	12.80	10.52	14.57
Middle SDI	Female	Esophageal cancer	2016	4.31	3.05	4.90
Middle SDI	Both	Esophageal cancer	2016	8.32	6.79	9.26
High SDI	Male	Esophageal cancer	2017	7.26	6.89	7.58

High SDI	Female	Esophageal cancer	2017	1.51	1.37	1.61
High SDI	Both	Esophageal cancer	2017	4.16	3.92	4.35
High-middle SDI	Male	Esophageal cancer	2017	11.33	9.04	13.13
High-middle SDI	Female	Esophageal cancer	2017	2.66	1.98	3.18
High-middle SDI	Both	Esophageal cancer	2017	6.54	5.27	7.36
Low SDI	Male	Esophageal cancer	2017	6.60	5.33	8.00
Low SDI	Female	Esophageal cancer	2017	4.77	3.89	5.75
Low SDI	Both	Esophageal cancer	2017	5.68	4.80	6.66
Low-middle SDI	Male	Esophageal cancer	2017	5.97	5.27	8.73
Low-middle SDI	Female	Esophageal cancer	2017	3.17	2.78	4.40
Low-middle SDI	Both	Esophageal cancer	2017	4.51	4.05	6.39
Middle SDI	Male	Esophageal cancer	2017	12.58	10.20	14.44
Middle SDI	Female	Esophageal cancer	2017	4.22	2.96	4.85
Middle SDI	Both	Esophageal cancer	2017	8.16	6.55	9.13
High SDI	Male	Esophageal cancer	2018	7.28	6.88	7.64
High SDI	Female	Esophageal cancer	2018	1.51	1.36	1.61
High SDI	Both	Esophageal cancer	2018	4.18	3.94	4.38
High-middle SDI	Male	Esophageal cancer	2018	11.35	9.05	13.30
High-middle SDI	Female	Esophageal cancer	2018	2.66	1.95	3.22
High-middle SDI	Both	Esophageal cancer	2018	6.56	5.28	7.49
Low SDI	Male	Esophageal cancer	2018	6.63	5.44	8.09
Low SDI	Female	Esophageal cancer	2018	4.77	3.84	5.78
Low SDI	Both	Esophageal cancer	2018	5.70	4.78	6.69
Low-middle SDI	Male	Esophageal cancer	2018	6.02	5.26	9.12
Low-middle SDI	Female	Esophageal cancer	2018	3.16	2.74	4.39
Low-middle SDI	Both	Esophageal cancer	2018	4.52	4.02	6.49
Middle SDI	Male	Esophageal cancer	2018	12.54	10.02	14.67
Middle SDI	Female	Esophageal cancer	2018	4.20	2.88	4.89
Middle SDI	Both	Esophageal cancer	2018	8.12	6.58	9.27
High SDI	Male	Esophageal cancer	2019	7.28	6.89	7.64
High SDI	Female	Esophageal cancer	2019	1.51	1.36	1.61
High SDI	Both	Esophageal cancer	2019	4.18	3.93	4.38
High-middle SDI	Male	Esophageal cancer	2019	11.47	8.91	13.59
High-middle SDI	Female	Esophageal cancer	2019	2.66	1.96	3.29
High-middle SDI	Both	Esophageal cancer	2019	6.62	5.29	7.62
Low SDI	Male	Esophageal cancer	2019	6.63	5.29	8.19
Low SDI	Female	Esophageal cancer	2019	4.76	3.87	5.75
Low SDI	Both	Esophageal cancer	2019	5.69	4.75	6.75
Low-middle SDI	Male	Esophageal cancer	2019	6.04	5.29	8.88
Low-middle SDI	Female	Esophageal cancer	2019	3.15	2.72	4.39
Low-middle SDI	Both	Esophageal cancer	2019	4.53	4.02	6.39
Middle SDI	Male	Esophageal cancer	2019	12.62	10.12	15.04
Middle SDI	Female	Esophageal cancer	2019	4.18	2.96	4.95
Middle SDI	Both	Esophageal cancer	2019	8.15	6.54	9.39

location_name	sex_name	cause_name	year	Age-standardised incidence rate (per 100 000 person-years)	95% CI (lower)	95% CI (upper)
Afghanistan	Both	Esophageal cancer	2019	6.55	2.11	9.17
Albania	Both	Esophageal cancer	2019	1.38	1.00	2.01
Algeria	Both	Esophageal cancer	2019	1.08	0.79	1.37
American Samoa	Both	Esophageal cancer	2019	1.42	1.11	1.68
Andorra	Both	Esophageal cancer	2019	4.15	3.02	5.36
Angola	Both	Esophageal cancer	2019	8.24	4.44	11.10
Antigua and Barbuda	Both	Esophageal cancer	2019	2.98	2.53	3.54
Argentina	Both	Esophageal cancer	2019	4.97	3.93	6.25
Armenia	Both	Esophageal cancer	2019	1.53	1.26	1.84
Australia	Both	Esophageal cancer	2019	4.34	3.34	5.61
Austria	Both	Esophageal cancer	2019	3.02	2.40	3.77
Azerbaijan	Both	Esophageal cancer	2019	8.67	6.49	13.36
Bahamas	Both	Esophageal cancer	2019	5.06	4.03	6.28
Bahrain	Both	Esophageal cancer	2019	1.94	1.45	2.48
Bangladesh	Both	Esophageal cancer	2019	3.81	2.71	5.73
Barbados	Both	Esophageal cancer	2019	4.92	3.97	5.96
Belarus	Both	Esophageal cancer	2019	3.05	2.31	4.04
Belgium	Both	Esophageal cancer	2019	5.26	4.04	6.68
Belize	Both	Esophageal cancer	2019	2.38	2.03	2.77
Benin	Both	Esophageal cancer	2019	4.86	2.88	6.63
Bermuda	Both	Esophageal cancer	2019	4.44	3.67	5.46
Bhutan	Both	Esophageal cancer	2019	4.69	3.30	6.46
Bolivia (Plurinational State of)	Both	Esophageal cancer	2019	2.33	1.82	2.92
Bosnia and Herzegovina	Both	Esophageal cancer	2019	2.01	1.56	2.54
Botswana	Both	Esophageal cancer	2019	12.72	7.73	16.72
Brazil	Both	Esophageal cancer	2019	5.20	4.90	5.45
Brunei Darussalam	Both	Esophageal cancer	2019	3.25	2.76	3.99
Bulgaria	Both	Esophageal cancer	2019	1.98	1.53	2.50
Burkina Faso	Both	Esophageal cancer	2019	4.78	2.78	6.12
Burundi	Both	Esophageal cancer	2019	11.49	7.73	16.36
Cabo Verde	Both	Esophageal cancer	2019	15.57	13.02	18.14
Cambodia	Both	Esophageal cancer	2019	2.82	2.17	3.89
Cameroon	Both	Esophageal cancer	2019	5.64	3.10	7.97
Canada	Both	Esophageal cancer	2019	4.42	3.44	5.59
Central African Republic	Both	Esophageal cancer	2019	10.28	5.74	14.14
Chad	Both	Esophageal cancer	2019	4.64	2.64	6.33
Chile	Both	Esophageal cancer	2019	3.87	3.02	4.89
China	Both	Esophageal cancer	2019	13.90	10.70	16.52
Colombia	Both	Esophageal cancer	2019	1.91	1.44	2.47
Comoros	Both	Esophageal cancer	2019	11.25	7.65	15.77
Congo	Both	Esophageal cancer	2019	10.05	5.57	13.71
Cook Islands	Both	Esophageal cancer	2019	2.65	2.20	3.18
Costa Rica	Both	Esophageal cancer	2019	1.73	1.32	2.22
Côte d'Ivoire	Both	Esophageal cancer	2019	5.04	2.79	6.84
Croatia	Both	Esophageal cancer	2019	2.77	2.10	3.61
Cuba	Both	Esophageal cancer	2019	5.21	4.14	6.46
Cyprus	Both	Esophageal cancer	2019	1.41	1.08	1.69
Czechia	Both	Esophageal cancer	2019	3.36	2.68	4.19
Democratic People's Republic of Korea	Both	Esophageal cancer	2019	8.96	6.96	11.60
Democratic Republic of the Congo	Both	Esophageal cancer	2019	8.17	4.29	11.95
Denmark	Both	Esophageal cancer	2019	5.01	3.88	6.50
Djibouti	Both	Esophageal cancer	2019	11.37	7.36	17.27
Dominica	Both	Esophageal cancer	2019	4.87	3.91	6.02
Dominican Republic	Both	Esophageal cancer	2019	2.43	1.68	3.38
Ecuador	Both	Esophageal cancer	2019	1.52	1.18	2.03
Egypt	Both	Esophageal cancer	2019	1.50	1.04	2.01
El Salvador	Both	Esophageal cancer	2019	1.59	1.20	2.06
Equatorial Guinea	Both	Esophageal cancer	2019	8.49	4.42	14.49

Eritrea	Both	Esophageal cancer	2019	13.20	8.37	18.14
Estonia	Both	Esophageal cancer	2019	2.98	2.24	3.83
Eswatini	Both	Esophageal cancer	2019	15.14	9.70	20.59
Ethiopia	Both	Esophageal cancer	2019	2.67	2.11	3.72
Fiji	Both	Esophageal cancer	2019	2.67	1.51	3.45
Finland	Both	Esophageal cancer	2019	3.21	2.44	4.14
France	Both	Esophageal cancer	2019	4.62	3.58	5.95
Gabon	Both	Esophageal cancer	2019	10.56	6.01	13.93
Gambia	Both	Esophageal cancer	2019	2.09	1.57	2.66
Georgia	Both	Esophageal cancer	2019	1.80	1.47	2.17
Germany	Both	Esophageal cancer	2019	5.48	4.24	7.20
Ghana	Both	Esophageal cancer	2019	3.32	2.29	4.30
Greece	Both	Esophageal cancer	2019	1.57	1.22	2.00
Greenland	Both	Esophageal cancer	2019	12.96	10.41	15.38
Grenada	Both	Esophageal cancer	2019	6.48	5.68	7.32
Guam	Both	Esophageal cancer	2019	2.61	2.05	3.20
Guatemala	Both	Esophageal cancer	2019	2.05	1.61	2.59
Guinea	Both	Esophageal cancer	2019	1.91	1.44	2.45
Guinea-Bissau	Both	Esophageal cancer	2019	6.19	3.41	8.26
Guyana	Both	Esophageal cancer	2019	2.09	1.61	2.69
Haiti	Both	Esophageal cancer	2019	3.52	2.37	5.19
Honduras	Both	Esophageal cancer	2019	1.66	1.17	2.16
Hungary	Both	Esophageal cancer	2019	3.73	2.97	4.62
Iceland	Both	Esophageal cancer	2019	4.87	4.14	5.73
India	Both	Esophageal cancer	2019	3.36	2.78	4.89
Indonesia	Both	Esophageal cancer	2019	2.36	1.89	3.41
Iran (Islamic Republic of)	Both	Esophageal cancer	2019	4.36	3.43	4.80
Iraq	Both	Esophageal cancer	2019	1.26	0.97	1.55
Ireland	Both	Esophageal cancer	2019	7.61	5.82	9.89
Israel	Both	Esophageal cancer	2019	1.62	1.24	2.08
Italy	Both	Esophageal cancer	2019	2.05	1.67	2.48
Jamaica	Both	Esophageal cancer	2019	2.59	2.00	3.30
Japan	Both	Esophageal cancer	2019	6.45	5.29	7.79
Jordan	Both	Esophageal cancer	2019	1.14	0.93	1.41
Kazakhstan	Both	Esophageal cancer	2019	7.35	6.27	8.51
Kenya	Both	Esophageal cancer	2019	11.93	8.96	16.74
Kiribati	Both	Esophageal cancer	2019	7.66	3.30	9.94
Kuwait	Both	Esophageal cancer	2019	1.50	1.19	1.87
Kyrgyzstan	Both	Esophageal cancer	2019	4.13	3.51	4.82
Lao People's Democratic Republic	Both	Esophageal cancer	2019	2.39	1.66	3.50
Latvia	Both	Esophageal cancer	2019	3.30	2.54	4.28
Lebanon	Both	Esophageal cancer	2019	1.30	0.97	1.72
Lesotho	Both	Esophageal cancer	2019	14.59	9.17	19.34
Liberia	Both	Esophageal cancer	2019	4.88	2.69	6.74
Libya	Both	Esophageal cancer	2019	1.37	0.97	1.75
Lithuania	Both	Esophageal cancer	2019	3.79	2.99	4.75
Luxembourg	Both	Esophageal cancer	2019	3.84	3.07	4.86
Madagascar	Both	Esophageal cancer	2019	9.74	6.18	13.64
Malawi	Both	Esophageal cancer	2019	24.53	18.74	32.51
Malaysia	Both	Esophageal cancer	2019	2.66	2.06	3.34
Maldives	Both	Esophageal cancer	2019	2.05	1.65	2.48
Mali	Both	Esophageal cancer	2019	2.55	1.95	3.37
Malta	Both	Esophageal cancer	2019	2.50	2.04	3.06
Marshall Islands	Both	Esophageal cancer	2019	2.68	1.86	3.73
Mauritania	Both	Esophageal cancer	2019	4.14	2.44	5.53
Mauritius	Both	Esophageal cancer	2019	2.53	2.03	3.18
Mexico	Both	Esophageal cancer	2019	1.44	1.23	1.69
Micronesia (Federated States of)	Both	Esophageal cancer	2019	3.04	2.10	4.17
Monaco	Both	Esophageal cancer	2019	6.55	4.95	8.06
Mongolia	Both	Esophageal cancer	2019	21.93	14.53	27.95

Montenegro	Both	Esophageal cancer	2019	2.40	1.90	2.98
Morocco	Both	Esophageal cancer	2019	1.57	1.18	1.90
Mozambique	Both	Esophageal cancer	2019	7.80	5.76	10.31
Myanmar	Both	Esophageal cancer	2019	2.25	1.79	3.39
Namibia	Both	Esophageal cancer	2019	2.63	2.03	3.36
Nauru	Both	Esophageal cancer	2019	3.14	2.19	4.16
Nepal	Both	Esophageal cancer	2019	4.78	3.55	6.48
Netherlands	Both	Esophageal cancer	2019	8.37	6.47	10.58
New Zealand	Both	Esophageal cancer	2019	4.79	3.86	5.79
Nicaragua	Both	Esophageal cancer	2019	1.20	0.94	1.55
Niger	Both	Esophageal cancer	2019	3.86	2.24	5.22
Nigeria	Both	Esophageal cancer	2019	0.91	0.65	1.58
Niue	Both	Esophageal cancer	2019	2.46	1.88	3.01
North Macedonia	Both	Esophageal cancer	2019	1.52	1.19	1.93
Northern Mariana Islands	Both	Esophageal cancer	2019	2.95	2.10	3.52
Norway	Both	Esophageal cancer	2019	2.85	2.37	3.40
Oman	Both	Esophageal cancer	2019	2.37	1.84	2.79
Pakistan	Both	Esophageal cancer	2019	7.86	6.30	9.63
Palau	Both	Esophageal cancer	2019	2.45	1.95	3.08
Palestine	Both	Esophageal cancer	2019	1.10	0.90	1.49
Panama	Both	Esophageal cancer	2019	1.60	1.19	2.07
Papua New Guinea	Both	Esophageal cancer	2019	1.88	1.36	2.81
Paraguay	Both	Esophageal cancer	2019	3.83	2.84	5.03
Peru	Both	Esophageal cancer	2019	1.29	0.96	1.70
Philippines	Both	Esophageal cancer	2019	1.32	1.06	1.75
Poland	Both	Esophageal cancer	2019	3.19	2.60	3.91
Portugal	Both	Esophageal cancer	2019	3.47	2.61	4.55
Puerto Rico	Both	Esophageal cancer	2019	2.56	1.96	3.31
Qatar	Both	Esophageal cancer	2019	4.47	3.16	6.14
Republic of Korea	Both	Esophageal cancer	2019	3.76	2.96	5.16
Republic of Moldova	Both	Esophageal cancer	2019	1.79	1.51	2.13
Romania	Both	Esophageal cancer	2019	2.59	2.07	3.15
Russian Federation	Both	Esophageal cancer	2019	3.38	2.86	3.98
Rwanda	Both	Esophageal cancer	2019	10.80	7.10	15.23
Saint Kitts and Nevis	Both	Esophageal cancer	2019	4.33	3.60	5.14
Saint Lucia	Both	Esophageal cancer	2019	4.32	3.58	5.15
Saint Vincent and the Grenadines	Both	Esophageal cancer	2019	2.38	2.04	2.77
Samoa	Both	Esophageal cancer	2019	1.61	1.22	2.01
San Marino	Both	Esophageal cancer	2019	1.71	1.28	2.24
Sao Tome and Principe	Both	Esophageal cancer	2019	4.81	2.63	6.03
Saudi Arabia	Both	Esophageal cancer	2019	1.78	1.38	2.36
Senegal	Both	Esophageal cancer	2019	4.68	2.60	6.21
Serbia	Both	Esophageal cancer	2019	2.43	1.88	3.12
Seychelles	Both	Esophageal cancer	2019	5.63	4.76	6.67
Sierra Leone	Both	Esophageal cancer	2019	4.32	2.35	5.83
Singapore	Both	Esophageal cancer	2019	2.71	2.12	3.45
Slovakia	Both	Esophageal cancer	2019	3.48	2.49	4.62
Slovenia	Both	Esophageal cancer	2019	2.85	2.15	3.76
Solomon Islands	Both	Esophageal cancer	2019	3.34	2.41	4.63
Somalia	Both	Esophageal cancer	2019	12.37	7.94	17.53
South Africa	Both	Esophageal cancer	2019	9.91	8.78	12.09
South Sudan	Both	Esophageal cancer	2019	10.07	6.70	14.51
Spain	Both	Esophageal cancer	2019	3.03	2.31	3.90
Sri Lanka	Both	Esophageal cancer	2019	4.61	3.40	6.19
Sudan	Both	Esophageal cancer	2019	4.74	1.48	6.85
Suriname	Both	Esophageal cancer	2019	1.38	1.10	1.68
Sweden	Both	Esophageal cancer	2019	2.95	2.45	3.52
Switzerland	Both	Esophageal cancer	2019	4.09	3.13	5.31
Syrian Arab Republic	Both	Esophageal cancer	2019	0.92	0.69	1.19
Taiwan (Province of China)	Both	Esophageal cancer	2019	9.99	7.58	13.27

Tajikistan	Both	Esophageal cancer	2019	7.16	5.26	15.41
Thailand	Both	Esophageal cancer	2019	3.13	2.01	4.22
Timor-Leste	Both	Esophageal cancer	2019	2.32	1.69	3.40
Togo	Both	Esophageal cancer	2019	4.66	2.56	6.32
Tokelau	Both	Esophageal cancer	2019	1.90	1.44	2.44
Tonga	Both	Esophageal cancer	2019	2.07	1.57	2.67
Trinidad and Tobago	Both	Esophageal cancer	2019	1.53	1.12	2.01
Tunisia	Both	Esophageal cancer	2019	0.96	0.67	1.30
Turkey	Both	Esophageal cancer	2019	1.56	1.21	1.96
Turkmenistan	Both	Esophageal cancer	2019	9.58	7.60	11.95
Tuvalu	Both	Esophageal cancer	2019	2.30	1.68	3.10
Uganda	Both	Esophageal cancer	2019	15.61	12.06	19.47
Ukraine	Both	Esophageal cancer	2019	2.99	2.39	3.72
United Arab Emirates	Both	Esophageal cancer	2019	8.08	2.54	13.84
United Kingdom	Both	Esophageal cancer	2019	8.23	6.80	9.88
United Republic of Tanzania	Both	Esophageal cancer	2019	11.85	7.69	16.53
United States of America	Both	Esophageal cancer	2019	4.20	3.54	4.98
United States Virgin Islands	Both	Esophageal cancer	2019	4.19	3.37	4.94
Uruguay	Both	Esophageal cancer	2019	6.12	4.76	7.83
Uzbekistan	Both	Esophageal cancer	2019	6.58	5.51	7.66
Vanuatu	Both	Esophageal cancer	2019	2.56	1.87	3.55
Venezuela (Bolivarian Republic of)	Both	Esophageal cancer	2019	1.97	1.44	2.59
Viet Nam	Both	Esophageal cancer	2019	2.89	2.04	3.65
Yemen	Both	Esophageal cancer	2019	4.18	1.28	6.31
Zambia	Both	Esophageal cancer	2019	14.10	9.26	18.84
Zimbabwe	Both	Esophageal cancer	2019	15.40	12.22	18.95

location_name	sex_name	cause_name	year	Age-standardised death rate (per 100 000 person-years)	95% CI (lower)	95% CI (upper)
Afghanistan	Both	Esophageal cancer	2019	6.96	2.25	9.70
Albania	Both	Esophageal cancer	2019	1.44	1.05	2.06
Algeria	Both	Esophageal cancer	2019	1.15	0.85	1.44
American Samoa	Both	Esophageal cancer	2019	1.52	1.18	1.78
Andorra	Both	Esophageal cancer	2019	3.56	2.63	4.55
Angola	Both	Esophageal cancer	2019	8.81	4.73	11.89
Antigua and Barbuda	Both	Esophageal cancer	2019	3.11	2.64	3.66
Argentina	Both	Esophageal cancer	2019	5.08	4.66	5.53
Armenia	Both	Esophageal cancer	2019	1.62	1.34	1.94
Australia	Both	Esophageal cancer	2019	4.03	3.61	4.45
Austria	Both	Esophageal cancer	2019	2.49	2.26	2.73
Azerbaijan	Both	Esophageal cancer	2019	9.32	6.87	14.35
Bahamas	Both	Esophageal cancer	2019	5.19	4.15	6.40
Bahrain	Both	Esophageal cancer	2019	2.03	1.52	2.57
Bangladesh	Both	Esophageal cancer	2019	4.00	2.87	6.00
Barbados	Both	Esophageal cancer	2019	5.09	4.14	6.13
Belarus	Both	Esophageal cancer	2019	2.96	2.24	3.91
Belgium	Both	Esophageal cancer	2019	4.52	4.12	4.93
Belize	Both	Esophageal cancer	2019	2.46	2.10	2.85
Benin	Both	Esophageal cancer	2019	5.18	3.05	6.99
Bermuda	Both	Esophageal cancer	2019	4.26	3.52	5.21
Bhutan	Both	Esophageal cancer	2019	5.00	3.56	6.86
Bolivia (Plurinational State of)	Both	Esophageal cancer	2019	2.59	2.03	3.22
Bosnia and Herzegovina	Both	Esophageal cancer	2019	2.06	1.61	2.60
Botswana	Both	Esophageal cancer	2019	13.21	8.08	17.32
Brazil	Both	Esophageal cancer	2019	5.28	4.95	5.56
Brunei Darussalam	Both	Esophageal cancer	2019	3.03	2.57	3.73
Bulgaria	Both	Esophageal cancer	2019	1.95	1.51	2.46
Burkina Faso	Both	Esophageal cancer	2019	5.09	2.96	6.48
Burundi	Both	Esophageal cancer	2019	12.18	8.18	17.18
Cabo Verde	Both	Esophageal cancer	2019	16.38	13.70	18.91
Cambodia	Both	Esophageal cancer	2019	2.97	2.30	4.10
Cameroon	Both	Esophageal cancer	2019	5.98	3.28	8.40
Canada	Both	Esophageal cancer	2019	3.64	3.27	3.98
Central African Republic	Both	Esophageal cancer	2019	10.95	6.14	14.94
Chad	Both	Esophageal cancer	2019	4.95	2.82	6.72
Chile	Both	Esophageal cancer	2019	4.00	3.54	4.42
China	Both	Esophageal cancer	2019	13.15	10.27	15.68
Colombia	Both	Esophageal cancer	2019	2.00	1.52	2.58
Comoros	Both	Esophageal cancer	2019	11.94	8.15	16.62
Congo	Both	Esophageal cancer	2019	10.76	5.96	14.53
Cook Islands	Both	Esophageal cancer	2019	2.67	2.24	3.18
Costa Rica	Both	Esophageal cancer	2019	1.79	1.37	2.28
Côte d'Ivoire	Both	Esophageal cancer	2019	5.35	2.95	7.25
Croatia	Both	Esophageal cancer	2019	2.62	1.99	3.38
Cuba	Both	Esophageal cancer	2019	5.08	4.05	6.28
Cyprus	Both	Esophageal cancer	2019	1.27	0.97	1.50
Czechia	Both	Esophageal cancer	2019	3.09	2.48	3.85
Democratic People's Republic of Korea	Both	Esophageal cancer	2019	9.01	7.06	11.56
Democratic Republic of the Congo	Both	Esophageal cancer	2019	8.71	4.60	12.77
Denmark	Both	Esophageal cancer	2019	4.50	4.00	5.04
Djibouti	Both	Esophageal cancer	2019	12.13	7.90	18.30
Dominica	Both	Esophageal cancer	2019	5.14	4.14	6.32
Dominican Republic	Both	Esophageal cancer	2019	2.55	1.79	3.52
Ecuador	Both	Esophageal cancer	2019	1.68	1.30	2.21
Egypt	Both	Esophageal cancer	2019	1.54	1.06	2.09
El Salvador	Both	Esophageal cancer	2019	1.68	1.28	2.17

Equatorial Guinea	Both	Esophageal cancer	2019	9.20	4.77	15.56
Eritrea	Both	Esophageal cancer	2019	13.92	8.85	18.99
Estonia	Both	Esophageal cancer	2019	2.91	2.19	3.74
Eswatini	Both	Esophageal cancer	2019	15.86	10.18	21.40
Ethiopia	Both	Esophageal cancer	2019	2.87	2.24	4.03
Fiji	Both	Esophageal cancer	2019	2.90	1.65	3.72
Finland	Both	Esophageal cancer	2019	2.50	2.23	2.79
France	Both	Esophageal cancer	2019	3.96	3.55	4.35
Gabon	Both	Esophageal cancer	2019	11.22	6.38	14.63
Gambia	Both	Esophageal cancer	2019	2.22	1.67	2.79
Georgia	Both	Esophageal cancer	2019	1.85	1.52	2.22
Germany	Both	Esophageal cancer	2019	3.71	3.39	4.07
Ghana	Both	Esophageal cancer	2019	3.52	2.42	4.54
Greece	Both	Esophageal cancer	2019	1.39	1.26	1.52
Greenland	Both	Esophageal cancer	2019	13.13	10.43	15.69
Grenada	Both	Esophageal cancer	2019	6.66	5.85	7.56
Guam	Both	Esophageal cancer	2019	2.65	2.08	3.22
Guatemala	Both	Esophageal cancer	2019	2.23	1.77	2.80
Guinea	Both	Esophageal cancer	2019	2.02	1.54	2.57
Guinea-Bissau	Both	Esophageal cancer	2019	6.42	3.55	8.59
Guyana	Both	Esophageal cancer	2019	2.18	1.68	2.77
Haiti	Both	Esophageal cancer	2019	3.75	2.55	5.46
Honduras	Both	Esophageal cancer	2019	1.80	1.27	2.32
Hungary	Both	Esophageal cancer	2019	3.61	2.89	4.46
Iceland	Both	Esophageal cancer	2019	3.92	3.41	4.51
India	Both	Esophageal cancer	2019	3.49	2.90	5.05
Indonesia	Both	Esophageal cancer	2019	2.51	2.02	3.58
Iran (Islamic Republic of)	Both	Esophageal cancer	2019	4.54	3.54	4.99
Iraq	Both	Esophageal cancer	2019	1.30	1.01	1.59
Ireland	Both	Esophageal cancer	2019	6.41	5.59	7.31
Israel	Both	Esophageal cancer	2019	1.53	1.35	1.71
Italy	Both	Esophageal cancer	2019	1.83	1.69	1.96
Jamaica	Both	Esophageal cancer	2019	2.70	2.10	3.43
Japan	Both	Esophageal cancer	2019	3.91	3.59	4.19
Jordan	Both	Esophageal cancer	2019	1.16	0.95	1.43
Kazakhstan	Both	Esophageal cancer	2019	7.80	6.67	9.02
Kenya	Both	Esophageal cancer	2019	13.72	10.54	19.58
Kiribati	Both	Esophageal cancer	2019	8.23	3.55	10.56
Kuwait	Both	Esophageal cancer	2019	1.53	1.22	1.90
Kyrgyzstan	Both	Esophageal cancer	2019	4.45	3.79	5.17
Lao People's Democratic Republic	Both	Esophageal cancer	2019	2.53	1.77	3.69
Latvia	Both	Esophageal cancer	2019	3.30	2.55	4.25
Lebanon	Both	Esophageal cancer	2019	1.25	0.94	1.66
Lesotho	Both	Esophageal cancer	2019	15.44	9.65	20.36
Liberia	Both	Esophageal cancer	2019	5.21	2.87	7.15
Libya	Both	Esophageal cancer	2019	1.41	1.01	1.80
Lithuania	Both	Esophageal cancer	2019	3.68	2.92	4.61
Luxembourg	Both	Esophageal cancer	2019	3.34	2.83	4.00
Madagascar	Both	Esophageal cancer	2019	10.31	6.61	14.36
Malawi	Both	Esophageal cancer	2019	25.76	19.76	33.94
Malaysia	Both	Esophageal cancer	2019	2.70	2.10	3.36
Maldives	Both	Esophageal cancer	2019	2.01	1.62	2.44
Mali	Both	Esophageal cancer	2019	2.69	2.08	3.53
Malta	Both	Esophageal cancer	2019	2.15	1.81	2.54
Marshall Islands	Both	Esophageal cancer	2019	2.87	2.00	3.93
Mauritania	Both	Esophageal cancer	2019	4.46	2.64	5.91
Mauritius	Both	Esophageal cancer	2019	2.52	2.02	3.15
Mexico	Both	Esophageal cancer	2019	1.51	1.28	1.75
Micronesia (Federated States of)	Both	Esophageal cancer	2019	3.23	2.26	4.39

Monaco	Both	Esophageal cancer	2019	5.27	4.07	6.45
Mongolia	Both	Esophageal cancer	2019	24.53	15.33	31.27
Montenegro	Both	Esophageal cancer	2019	2.37	1.88	2.93
Morocco	Both	Esophageal cancer	2019	1.65	1.26	1.99
Mozambique	Both	Esophageal cancer	2019	8.37	6.12	11.08
Myanmar	Both	Esophageal cancer	2019	2.37	1.90	3.58
Namibia	Both	Esophageal cancer	2019	2.74	2.14	3.46
Nauru	Both	Esophageal cancer	2019	3.31	2.35	4.32
Nepal	Both	Esophageal cancer	2019	5.10	3.81	6.80
Netherlands	Both	Esophageal cancer	2019	6.98	6.34	7.64
New Zealand	Both	Esophageal cancer	2019	3.93	3.53	4.33
Nicaragua	Both	Esophageal cancer	2019	1.32	1.06	1.70
Niger	Both	Esophageal cancer	2019	4.15	2.40	5.56
Nigeria	Both	Esophageal cancer	2019	1.00	0.71	1.76
Niue	Both	Esophageal cancer	2019	2.52	1.92	3.05
North Macedonia	Both	Esophageal cancer	2019	1.56	1.23	1.97
Northern Mariana Islands	Both	Esophageal cancer	2019	3.01	2.12	3.55
Norway	Both	Esophageal cancer	2019	2.44	2.26	2.65
Oman	Both	Esophageal cancer	2019	2.38	1.87	2.78
Pakistan	Both	Esophageal cancer	2019	8.23	6.62	9.96
Palau	Both	Esophageal cancer	2019	2.51	2.00	3.12
Palestine	Both	Esophageal cancer	2019	1.16	0.94	1.58
Panama	Both	Esophageal cancer	2019	1.64	1.24	2.12
Papua New Guinea	Both	Esophageal cancer	2019	2.02	1.47	3.04
Paraguay	Both	Esophageal cancer	2019	3.99	2.96	5.22
Peru	Both	Esophageal cancer	2019	1.38	1.03	1.80
Philippines	Both	Esophageal cancer	2019	1.36	1.10	1.88
Poland	Both	Esophageal cancer	2019	3.28	2.70	3.99
Portugal	Both	Esophageal cancer	2019	3.17	2.86	3.50
Puerto Rico	Both	Esophageal cancer	2019	2.50	1.92	3.22
Qatar	Both	Esophageal cancer	2019	4.84	3.38	6.65
Republic of Korea	Both	Esophageal cancer	2019	2.43	2.09	3.52
Republic of Moldova	Both	Esophageal cancer	2019	1.78	1.50	2.11
Romania	Both	Esophageal cancer	2019	2.52	2.02	3.06
Russian Federation	Both	Esophageal cancer	2019	3.24	2.73	3.79
Rwanda	Both	Esophageal cancer	2019	11.50	7.66	16.11
Saint Kitts and Nevis	Both	Esophageal cancer	2019	4.36	3.67	5.15
Saint Lucia	Both	Esophageal cancer	2019	4.47	3.70	5.31
Saint Vincent and the Grenadines	Both	Esophageal cancer	2019	2.48	2.13	2.87
Samoa	Both	Esophageal cancer	2019	1.68	1.29	2.08
San Marino	Both	Esophageal cancer	2019	1.47	0.97	2.09
Sao Tome and Principe	Both	Esophageal cancer	2019	5.13	2.77	6.43
Saudi Arabia	Both	Esophageal cancer	2019	1.78	1.40	2.36
Senegal	Both	Esophageal cancer	2019	5.01	2.78	6.60
Serbia	Both	Esophageal cancer	2019	2.45	1.91	3.14
Seychelles	Both	Esophageal cancer	2019	5.61	4.76	6.66
Sierra Leone	Both	Esophageal cancer	2019	4.60	2.51	6.21
Singapore	Both	Esophageal cancer	2019	1.81	1.57	2.10
Slovakia	Both	Esophageal cancer	2019	3.19	2.29	4.22
Slovenia	Both	Esophageal cancer	2019	2.54	1.91	3.33
Solomon Islands	Both	Esophageal cancer	2019	3.48	2.57	4.78
Somalia	Both	Esophageal cancer	2019	13.21	8.48	18.69
South Africa	Both	Esophageal cancer	2019	10.57	9.46	12.54
South Sudan	Both	Esophageal cancer	2019	10.79	7.13	15.50
Spain	Both	Esophageal cancer	2019	2.55	2.32	2.81
Sri Lanka	Both	Esophageal cancer	2019	4.49	3.32	6.01
Sudan	Both	Esophageal cancer	2019	5.04	1.58	7.21
Suriname	Both	Esophageal cancer	2019	1.44	1.16	1.75
Sweden	Both	Esophageal cancer	2019	2.71	2.50	2.92

Switzerland	Both	Esophageal cancer	2019	3.31	2.98	3.68
Syrian Arab Republic	Both	Esophageal cancer	2019	0.96	0.73	1.23
Taiwan (Province of China)	Both	Esophageal cancer	2019	7.89	6.02	10.38
Tajikistan	Both	Esophageal cancer	2019	7.72	5.69	16.63
Thailand	Both	Esophageal cancer	2019	3.00	1.95	4.03
Timor-Leste	Both	Esophageal cancer	2019	2.48	1.82	3.60
Togo	Both	Esophageal cancer	2019	4.92	2.69	6.63
Tokelau	Both	Esophageal cancer	2019	2.02	1.54	2.57
Tonga	Both	Esophageal cancer	2019	2.21	1.67	2.86
Trinidad and Tobago	Both	Esophageal cancer	2019	1.58	1.17	2.07
Tunisia	Both	Esophageal cancer	2019	0.97	0.68	1.31
Turkey	Both	Esophageal cancer	2019	1.52	1.18	1.93
Turkmenistan	Both	Esophageal cancer	2019	9.97	7.94	12.44
Tuvalu	Both	Esophageal cancer	2019	2.45	1.80	3.30
Uganda	Both	Esophageal cancer	2019	16.53	12.84	20.60
Ukraine	Both	Esophageal cancer	2019	2.78	2.20	3.42
United Arab Emirates	Both	Esophageal cancer	2019	8.42	2.68	14.24
United Kingdom	Both	Esophageal cancer	2019	7.77	7.31	8.09
United Republic of Tanzania	Both	Esophageal cancer	2019	12.56	8.13	17.39
United States of America	Both	Esophageal cancer	2019	3.86	3.69	4.02
United States Virgin Islands	Both	Esophageal cancer	2019	4.27	3.48	5.03
Uruguay	Both	Esophageal cancer	2019	6.22	5.63	6.93
Uzbekistan	Both	Esophageal cancer	2019	6.98	5.88	8.09
Vanuatu	Both	Esophageal cancer	2019	2.75	2.02	3.80
Venezuela (Bolivarian Republic of)	Both	Esophageal cancer	2019	2.05	1.50	2.67
Viet Nam	Both	Esophageal cancer	2019	2.87	2.07	3.58
Yemen	Both	Esophageal cancer	2019	4.45	1.36	6.78
Zambia	Both	Esophageal cancer	2019	14.83	9.78	19.76
Zimbabwe	Both	Esophageal cancer	2019	16.02	12.75	19.64

sex_name	cause_name	year	Age-standardised incidence rate (per 100 000 person-years)	95% CI (lower)	95% CI (upper)
Male	Pancreatic cancer	1990	5.99	5.71	6.26
Female	Pancreatic cancer	1990	4.52	4.27	4.70
Both	Pancreatic cancer	1990	5.22	4.97	5.40
Male	Pancreatic cancer	1991	6.03	5.76	6.30
Female	Pancreatic cancer	1991	4.56	4.31	4.73
Both	Pancreatic cancer	1991	5.26	5.02	5.44
Male	Pancreatic cancer	1992	6.08	5.81	6.33
Female	Pancreatic cancer	1992	4.60	4.34	4.76
Both	Pancreatic cancer	1992	5.31	5.06	5.48
Male	Pancreatic cancer	1993	6.20	5.95	6.44
Female	Pancreatic cancer	1993	4.71	4.45	4.87
Both	Pancreatic cancer	1993	5.43	5.18	5.59
Male	Pancreatic cancer	1994	6.24	5.97	6.46
Female	Pancreatic cancer	1994	4.74	4.46	4.91
Both	Pancreatic cancer	1994	5.46	5.21	5.62
Male	Pancreatic cancer	1995	6.26	6.01	6.46
Female	Pancreatic cancer	1995	4.75	4.48	4.92
Both	Pancreatic cancer	1995	5.48	5.23	5.64
Male	Pancreatic cancer	1996	6.22	5.98	6.43
Female	Pancreatic cancer	1996	4.76	4.47	4.93
Both	Pancreatic cancer	1996	5.47	5.21	5.62
Male	Pancreatic cancer	1997	6.20	5.97	6.39
Female	Pancreatic cancer	1997	4.78	4.49	4.96
Both	Pancreatic cancer	1997	5.47	5.21	5.62
Male	Pancreatic cancer	1998	6.25	6.02	6.44
Female	Pancreatic cancer	1998	4.82	4.53	4.99
Both	Pancreatic cancer	1998	5.51	5.26	5.67
Male	Pancreatic cancer	1999	6.33	6.07	6.52
Female	Pancreatic cancer	1999	4.88	4.57	5.06
Both	Pancreatic cancer	1999	5.59	5.32	5.74
Male	Pancreatic cancer	2000	6.39	6.15	6.60
Female	Pancreatic cancer	2000	4.93	4.62	5.12
Both	Pancreatic cancer	2000	5.64	5.38	5.82
Male	Pancreatic cancer	2001	6.46	6.19	6.64
Female	Pancreatic cancer	2001	4.98	4.64	5.18
Both	Pancreatic cancer	2001	5.70	5.39	5.87
Male	Pancreatic cancer	2002	6.57	6.31	6.79
Female	Pancreatic cancer	2002	5.05	4.72	5.25
Both	Pancreatic cancer	2002	5.79	5.51	5.97
Male	Pancreatic cancer	2003	6.68	6.39	6.92
Female	Pancreatic cancer	2003	5.12	4.78	5.33
Both	Pancreatic cancer	2003	5.88	5.56	6.07
Male	Pancreatic cancer	2004	6.72	6.44	6.95
Female	Pancreatic cancer	2004	5.13	4.76	5.35
Both	Pancreatic cancer	2004	5.91	5.57	6.10
Male	Pancreatic cancer	2005	6.85	6.56	7.08
Female	Pancreatic cancer	2005	5.21	4.82	5.42
Both	Pancreatic cancer	2005	6.01	5.66	6.20

Male	Pancreatic cancer	2006	6.86	6.58	7.09
Female	Pancreatic cancer	2006	5.23	4.85	5.45
Both	Pancreatic cancer	2006	6.02	5.69	6.23
Male	Pancreatic cancer	2007	6.94	6.67	7.16
Female	Pancreatic cancer	2007	5.28	4.88	5.51
Both	Pancreatic cancer	2007	6.09	5.74	6.30
Male	Pancreatic cancer	2008	7.05	6.76	7.28
Female	Pancreatic cancer	2008	5.35	4.92	5.58
Both	Pancreatic cancer	2008	6.17	5.82	6.39
Male	Pancreatic cancer	2009	7.13	6.81	7.36
Female	Pancreatic cancer	2009	5.39	4.97	5.63
Both	Pancreatic cancer	2009	6.23	5.87	6.45
Male	Pancreatic cancer	2010	7.23	6.90	7.51
Female	Pancreatic cancer	2010	5.42	4.99	5.68
Both	Pancreatic cancer	2010	6.29	5.91	6.54
Male	Pancreatic cancer	2011	7.25	6.90	7.52
Female	Pancreatic cancer	2011	5.46	5.01	5.74
Both	Pancreatic cancer	2011	6.33	5.93	6.59
Male	Pancreatic cancer	2012	7.30	6.93	7.59
Female	Pancreatic cancer	2012	5.47	5.02	5.74
Both	Pancreatic cancer	2012	6.35	5.97	6.61
Male	Pancreatic cancer	2013	7.31	6.93	7.64
Female	Pancreatic cancer	2013	5.48	5.02	5.76
Both	Pancreatic cancer	2013	6.36	5.96	6.62
Male	Pancreatic cancer	2014	7.30	6.93	7.62
Female	Pancreatic cancer	2014	5.50	5.03	5.79
Both	Pancreatic cancer	2014	6.37	5.96	6.64
Male	Pancreatic cancer	2015	7.37	6.98	7.70
Female	Pancreatic cancer	2015	5.55	5.08	5.85
Both	Pancreatic cancer	2015	6.43	6.01	6.70
Male	Pancreatic cancer	2016	7.37	6.97	7.74
Female	Pancreatic cancer	2016	5.58	5.09	5.90
Both	Pancreatic cancer	2016	6.44	6.00	6.75
Male	Pancreatic cancer	2017	7.36	6.89	7.84
Female	Pancreatic cancer	2017	5.58	5.05	6.00
Both	Pancreatic cancer	2017	6.44	5.95	6.84
Male	Pancreatic cancer	2018	7.43	6.88	7.99
Female	Pancreatic cancer	2018	5.65	5.05	6.14
Both	Pancreatic cancer	2018	6.51	6.01	6.98
Male	Pancreatic cancer	2019	7.49	6.85	8.12
Female	Pancreatic cancer	2019	5.71	5.11	6.28
Both	Pancreatic cancer	2019	6.57	6.00	7.09

location_name	sex_name	cause_name	year	Age-standardised incidence rate (per 100 000 person-years)	95% CI (lower)	95% CI (upper)
High SDI	Male	Pancreatic cancer	1990	10.42	10.12	10.61
High SDI	Female	Pancreatic cancer	1990	7.26	6.86	7.47
High SDI	Both	Pancreatic cancer	1990	8.67	8.30	8.86
High-middle SDI	Male	Pancreatic cancer	1990	7.56	7.20	7.93
High-middle SDI	Female	Pancreatic cancer	1990	4.98	4.71	5.25
High-middle SDI	Both	Pancreatic cancer	1990	6.14	5.89	6.37
Low SDI	Male	Pancreatic cancer	1990	1.97	1.44	2.48
Low SDI	Female	Pancreatic cancer	1990	1.21	0.97	1.55
Low SDI	Both	Pancreatic cancer	1990	1.59	1.27	1.91
Low-middle SDI	Male	Pancreatic cancer	1990	2.04	1.63	2.51
Low-middle SDI	Female	Pancreatic cancer	1990	1.54	1.33	1.82
Low-middle SDI	Both	Pancreatic cancer	1990	1.79	1.53	2.04
Middle SDI	Male	Pancreatic cancer	1990	3.07	2.76	3.40
Middle SDI	Female	Pancreatic cancer	1990	2.38	2.21	2.56
Middle SDI	Both	Pancreatic cancer	1990	2.72	2.54	2.91
High SDI	Male	Pancreatic cancer	1991	10.47	10.15	10.66
High SDI	Female	Pancreatic cancer	1991	7.32	6.91	7.53
High SDI	Both	Pancreatic cancer	1991	8.73	8.34	8.92
High-middle SDI	Male	Pancreatic cancer	1991	7.66	7.32	8.00
High-middle SDI	Female	Pancreatic cancer	1991	5.06	4.80	5.31
High-middle SDI	Both	Pancreatic cancer	1991	6.23	5.98	6.45
Low SDI	Male	Pancreatic cancer	1991	1.70	1.25	2.19
Low SDI	Female	Pancreatic cancer	1991	1.22	0.98	1.57
Low SDI	Both	Pancreatic cancer	1991	1.47	1.16	1.76
Low-middle SDI	Male	Pancreatic cancer	1991	2.01	1.60	2.47
Low-middle SDI	Female	Pancreatic cancer	1991	1.55	1.35	1.83
Low-middle SDI	Both	Pancreatic cancer	1991	1.78	1.52	2.03
Middle SDI	Male	Pancreatic cancer	1991	2.68	2.45	2.91
Middle SDI	Female	Pancreatic cancer	1991	2.42	2.24	2.61
Middle SDI	Both	Pancreatic cancer	1991	2.55	2.40	2.69
High SDI	Male	Pancreatic cancer	1992	10.52	10.20	10.71
High SDI	Female	Pancreatic cancer	1992	7.36	6.94	7.58
High SDI	Both	Pancreatic cancer	1992	8.78	8.38	8.97
High-middle SDI	Male	Pancreatic cancer	1992	7.82	7.49	8.14
High-middle SDI	Female	Pancreatic cancer	1992	5.13	4.87	5.35
High-middle SDI	Both	Pancreatic cancer	1992	6.34	6.08	6.55
Low SDI	Male	Pancreatic cancer	1992	1.71	1.25	2.19
Low SDI	Female	Pancreatic cancer	1992	1.23	0.99	1.58
Low SDI	Both	Pancreatic cancer	1992	1.47	1.18	1.77
Low-middle SDI	Male	Pancreatic cancer	1992	2.02	1.62	2.47
Low-middle SDI	Female	Pancreatic cancer	1992	1.58	1.37	1.86
Low-middle SDI	Both	Pancreatic cancer	1992	1.80	1.54	2.06
Middle SDI	Male	Pancreatic cancer	1992	2.75	2.55	2.96
Middle SDI	Female	Pancreatic cancer	1992	2.46	2.29	2.65
Middle SDI	Both	Pancreatic cancer	1992	2.61	2.46	2.75
High SDI	Male	Pancreatic cancer	1993	10.69	10.37	10.87
High SDI	Female	Pancreatic cancer	1993	7.51	7.05	7.74
High SDI	Both	Pancreatic cancer	1993	8.94	8.54	9.14
High-middle SDI	Male	Pancreatic cancer	1993	8.09	7.78	8.40

High-middle SDI	Female	Pancreatic cancer	1993		5.32	5.05	5.55
High-middle SDI	Both	Pancreatic cancer	1993		6.58	6.32	6.77
Low SDI	Male	Pancreatic cancer	1993		1.72	1.28	2.21
Low SDI	Female	Pancreatic cancer	1993		1.25	1.01	1.58
Low SDI	Both	Pancreatic cancer	1993		1.49	1.19	1.78
Low-middle SDI	Male	Pancreatic cancer	1993		2.07	1.68	2.50
Low-middle SDI	Female	Pancreatic cancer	1993		1.63	1.43	1.89
Low-middle SDI	Both	Pancreatic cancer	1993		1.85	1.60	2.09
Middle SDI	Male	Pancreatic cancer	1993		2.75	2.57	2.96
Middle SDI	Female	Pancreatic cancer	1993		2.53	2.36	2.75
Middle SDI	Both	Pancreatic cancer	1993		2.64	2.50	2.79
High SDI	Male	Pancreatic cancer	1994		10.67	10.34	10.87
High SDI	Female	Pancreatic cancer	1994		7.52	7.06	7.75
High SDI	Both	Pancreatic cancer	1994		8.95	8.54	9.16
High-middle SDI	Male	Pancreatic cancer	1994		8.21	7.89	8.53
High-middle SDI	Female	Pancreatic cancer	1994		5.39	5.12	5.62
High-middle SDI	Both	Pancreatic cancer	1994		6.68	6.44	6.87
Low SDI	Male	Pancreatic cancer	1994		1.74	1.30	2.22
Low SDI	Female	Pancreatic cancer	1994		1.26	1.03	1.59
Low SDI	Both	Pancreatic cancer	1994		1.50	1.21	1.80
Low-middle SDI	Male	Pancreatic cancer	1994		2.11	1.74	2.53
Low-middle SDI	Female	Pancreatic cancer	1994		1.68	1.48	1.93
Low-middle SDI	Both	Pancreatic cancer	1994		1.89	1.65	2.12
Middle SDI	Male	Pancreatic cancer	1994		2.84	2.67	3.02
Middle SDI	Female	Pancreatic cancer	1994		2.58	2.40	2.79
Middle SDI	Both	Pancreatic cancer	1994		2.71	2.58	2.85
High SDI	Male	Pancreatic cancer	1995		10.77	10.44	10.97
High SDI	Female	Pancreatic cancer	1995		7.59	7.14	7.83
High SDI	Both	Pancreatic cancer	1995		9.04	8.62	9.26
High-middle SDI	Male	Pancreatic cancer	1995		8.16	7.87	8.48
High-middle SDI	Female	Pancreatic cancer	1995		5.35	5.10	5.55
High-middle SDI	Both	Pancreatic cancer	1995		6.63	6.39	6.82
Low SDI	Male	Pancreatic cancer	1995		1.75	1.31	2.22
Low SDI	Female	Pancreatic cancer	1995		1.28	1.04	1.61
Low SDI	Both	Pancreatic cancer	1995		1.52	1.23	1.82
Low-middle SDI	Male	Pancreatic cancer	1995		2.14	1.77	2.53
Low-middle SDI	Female	Pancreatic cancer	1995		1.72	1.52	1.96
Low-middle SDI	Both	Pancreatic cancer	1995		1.93	1.69	2.16
Middle SDI	Male	Pancreatic cancer	1995		2.90	2.74	3.07
Middle SDI	Female	Pancreatic cancer	1995		2.62	2.45	2.81
Middle SDI	Both	Pancreatic cancer	1995		2.76	2.62	2.89
High SDI	Male	Pancreatic cancer	1996		10.69	10.33	10.89
High SDI	Female	Pancreatic cancer	1996		7.60	7.12	7.84
High SDI	Both	Pancreatic cancer	1996		9.01	8.57	9.23
High-middle SDI	Male	Pancreatic cancer	1996		8.00	7.72	8.27
High-middle SDI	Female	Pancreatic cancer	1996		5.31	5.06	5.52
High-middle SDI	Both	Pancreatic cancer	1996		6.53	6.29	6.72
Low SDI	Male	Pancreatic cancer	1996		1.77	1.34	2.24
Low SDI	Female	Pancreatic cancer	1996		1.30	1.06	1.64
Low SDI	Both	Pancreatic cancer	1996		1.54	1.25	1.84
Low-middle SDI	Male	Pancreatic cancer	1996		2.19	1.84	2.58
Low-middle SDI	Female	Pancreatic cancer	1996		1.78	1.59	2.02

Low-middle SDI	Both	Pancreatic cancer	1996		1.99	1.75	2.21
Middle SDI	Male	Pancreatic cancer	1996		2.92	2.76	3.10
Middle SDI	Female	Pancreatic cancer	1996		2.70	2.52	2.91
Middle SDI	Both	Pancreatic cancer	1996		2.82	2.67	2.97
High SDI	Male	Pancreatic cancer	1997		10.62	10.27	10.83
High SDI	Female	Pancreatic cancer	1997		7.61	7.14	7.86
High SDI	Both	Pancreatic cancer	1997		8.99	8.56	9.22
High-middle SDI	Male	Pancreatic cancer	1997		7.88	7.60	8.15
High-middle SDI	Female	Pancreatic cancer	1997		5.30	5.04	5.48
High-middle SDI	Both	Pancreatic cancer	1997		6.47	6.22	6.65
Low SDI	Male	Pancreatic cancer	1997		1.80	1.36	2.27
Low SDI	Female	Pancreatic cancer	1997		1.33	1.09	1.69
Low SDI	Both	Pancreatic cancer	1997		1.57	1.28	1.87
Low-middle SDI	Male	Pancreatic cancer	1997		2.28	1.94	2.69
Low-middle SDI	Female	Pancreatic cancer	1997		1.86	1.67	2.09
Low-middle SDI	Both	Pancreatic cancer	1997		2.07	1.84	2.29
Middle SDI	Male	Pancreatic cancer	1997		2.99	2.84	3.16
Middle SDI	Female	Pancreatic cancer	1997		2.78	2.58	2.99
Middle SDI	Both	Pancreatic cancer	1997		2.89	2.74	3.05
High SDI	Male	Pancreatic cancer	1998		10.73	10.38	10.93
High SDI	Female	Pancreatic cancer	1998		7.69	7.21	7.94
High SDI	Both	Pancreatic cancer	1998		9.09	8.64	9.32
High-middle SDI	Male	Pancreatic cancer	1998		7.85	7.60	8.10
High-middle SDI	Female	Pancreatic cancer	1998		5.30	5.04	5.49
High-middle SDI	Both	Pancreatic cancer	1998		6.46	6.22	6.64
Low SDI	Male	Pancreatic cancer	1998		1.82	1.39	2.31
Low SDI	Female	Pancreatic cancer	1998		1.37	1.12	1.73
Low SDI	Both	Pancreatic cancer	1998		1.60	1.31	1.91
Low-middle SDI	Male	Pancreatic cancer	1998		2.33	2.00	2.74
Low-middle SDI	Female	Pancreatic cancer	1998		1.92	1.73	2.15
Low-middle SDI	Both	Pancreatic cancer	1998		2.13	1.91	2.34
Middle SDI	Male	Pancreatic cancer	1998		3.08	2.91	3.25
Middle SDI	Female	Pancreatic cancer	1998		2.83	2.64	3.06
Middle SDI	Both	Pancreatic cancer	1998		2.96	2.82	3.13
High SDI	Male	Pancreatic cancer	1999		10.84	10.48	11.06
High SDI	Female	Pancreatic cancer	1999		7.82	7.32	8.09
High SDI	Both	Pancreatic cancer	1999		9.22	8.75	9.46
High-middle SDI	Male	Pancreatic cancer	1999		7.97	7.68	8.22
High-middle SDI	Female	Pancreatic cancer	1999		5.36	5.11	5.56
High-middle SDI	Both	Pancreatic cancer	1999		6.55	6.31	6.73
Low SDI	Male	Pancreatic cancer	1999		1.83	1.40	2.31
Low SDI	Female	Pancreatic cancer	1999		1.39	1.14	1.75
Low SDI	Both	Pancreatic cancer	1999		1.61	1.31	1.91
Low-middle SDI	Male	Pancreatic cancer	1999		2.36	2.01	2.76
Low-middle SDI	Female	Pancreatic cancer	1999		1.95	1.76	2.19
Low-middle SDI	Both	Pancreatic cancer	1999		2.16	1.93	2.37
Middle SDI	Male	Pancreatic cancer	1999		3.16	2.97	3.35
Middle SDI	Female	Pancreatic cancer	1999		2.90	2.70	3.12
Middle SDI	Both	Pancreatic cancer	1999		3.03	2.87	3.21
High SDI	Male	Pancreatic cancer	2000		10.83	10.46	11.05
High SDI	Female	Pancreatic cancer	2000		7.88	7.37	8.15
High SDI	Both	Pancreatic cancer	2000		9.24	8.80	9.49

High-middle SDI	Male	Pancreatic cancer	2000	8.07	7.79	8.35
High-middle SDI	Female	Pancreatic cancer	2000	5.42	5.16	5.62
High-middle SDI	Both	Pancreatic cancer	2000	6.63	6.38	6.83
Low SDI	Male	Pancreatic cancer	2000	1.86	1.42	2.33
Low SDI	Female	Pancreatic cancer	2000	1.42	1.16	1.78
Low SDI	Both	Pancreatic cancer	2000	1.64	1.33	1.95
Low-middle SDI	Male	Pancreatic cancer	2000	2.42	2.07	2.81
Low-middle SDI	Female	Pancreatic cancer	2000	2.01	1.81	2.25
Low-middle SDI	Both	Pancreatic cancer	2000	2.21	1.99	2.44
Middle SDI	Male	Pancreatic cancer	2000	3.26	3.08	3.47
Middle SDI	Female	Pancreatic cancer	2000	2.98	2.75	3.22
Middle SDI	Both	Pancreatic cancer	2000	3.12	2.95	3.32
High SDI	Male	Pancreatic cancer	2001	10.85	10.47	11.06
High SDI	Female	Pancreatic cancer	2001	7.93	7.40	8.21
High SDI	Both	Pancreatic cancer	2001	9.29	8.80	9.54
High-middle SDI	Male	Pancreatic cancer	2001	8.18	7.86	8.48
High-middle SDI	Female	Pancreatic cancer	2001	5.48	5.18	5.70
High-middle SDI	Both	Pancreatic cancer	2001	6.72	6.42	6.92
Low SDI	Male	Pancreatic cancer	2001	1.89	1.45	2.37
Low SDI	Female	Pancreatic cancer	2001	1.46	1.19	1.82
Low SDI	Both	Pancreatic cancer	2001	1.68	1.37	2.00
Low-middle SDI	Male	Pancreatic cancer	2001	2.49	2.14	2.88
Low-middle SDI	Female	Pancreatic cancer	2001	2.09	1.90	2.30
Low-middle SDI	Both	Pancreatic cancer	2001	2.29	2.07	2.51
Middle SDI	Male	Pancreatic cancer	2001	3.35	3.15	3.57
Middle SDI	Female	Pancreatic cancer	2001	3.04	2.81	3.29
Middle SDI	Both	Pancreatic cancer	2001	3.20	3.01	3.40
High SDI	Male	Pancreatic cancer	2002	10.94	10.55	11.16
High SDI	Female	Pancreatic cancer	2002	8.02	7.47	8.31
High SDI	Both	Pancreatic cancer	2002	9.38	8.88	9.65
High-middle SDI	Male	Pancreatic cancer	2002	8.36	8.03	8.69
High-middle SDI	Female	Pancreatic cancer	2002	5.58	5.29	5.81
High-middle SDI	Both	Pancreatic cancer	2002	6.85	6.57	7.08
Low SDI	Male	Pancreatic cancer	2002	1.92	1.47	2.42
Low SDI	Female	Pancreatic cancer	2002	1.51	1.25	1.88
Low SDI	Both	Pancreatic cancer	2002	1.72	1.41	2.04
Low-middle SDI	Male	Pancreatic cancer	2002	2.55	2.22	2.94
Low-middle SDI	Female	Pancreatic cancer	2002	2.17	1.98	2.40
Low-middle SDI	Both	Pancreatic cancer	2002	2.36	2.15	2.57
Middle SDI	Male	Pancreatic cancer	2002	3.52	3.30	3.76
Middle SDI	Female	Pancreatic cancer	2002	3.11	2.87	3.37
Middle SDI	Both	Pancreatic cancer	2002	3.32	3.12	3.53
High SDI	Male	Pancreatic cancer	2003	11.08	10.68	11.31
High SDI	Female	Pancreatic cancer	2003	8.11	7.55	8.41
High SDI	Both	Pancreatic cancer	2003	9.50	8.98	9.76
High-middle SDI	Male	Pancreatic cancer	2003	8.56	8.23	8.91
High-middle SDI	Female	Pancreatic cancer	2003	5.70	5.39	5.94
High-middle SDI	Both	Pancreatic cancer	2003	7.01	6.71	7.24
Low SDI	Male	Pancreatic cancer	2003	1.96	1.50	2.44
Low SDI	Female	Pancreatic cancer	2003	1.54	1.27	1.92
Low SDI	Both	Pancreatic cancer	2003	1.75	1.42	2.08
Low-middle SDI	Male	Pancreatic cancer	2003	2.59	2.23	2.96

Low-middle SDI	Female	Pancreatic cancer	2003		2.20	2.01	2.43
Low-middle SDI	Both	Pancreatic cancer	2003		2.39	2.18	2.60
Middle SDI	Male	Pancreatic cancer	2003		3.60	3.39	3.84
Middle SDI	Female	Pancreatic cancer	2003		3.17	2.93	3.43
Middle SDI	Both	Pancreatic cancer	2003		3.39	3.20	3.61
High SDI	Male	Pancreatic cancer	2004		11.02	10.60	11.26
High SDI	Female	Pancreatic cancer	2004		8.13	7.53	8.43
High SDI	Both	Pancreatic cancer	2004		9.48	8.95	9.75
High-middle SDI	Male	Pancreatic cancer	2004		8.66	8.30	9.04
High-middle SDI	Female	Pancreatic cancer	2004		5.72	5.36	5.96
High-middle SDI	Both	Pancreatic cancer	2004		7.07	6.74	7.31
Low SDI	Male	Pancreatic cancer	2004		1.97	1.53	2.44
Low SDI	Female	Pancreatic cancer	2004		1.58	1.30	1.97
Low SDI	Both	Pancreatic cancer	2004		1.78	1.45	2.11
Low-middle SDI	Male	Pancreatic cancer	2004		2.60	2.26	2.94
Low-middle SDI	Female	Pancreatic cancer	2004		2.22	2.03	2.45
Low-middle SDI	Both	Pancreatic cancer	2004		2.41	2.18	2.62
Middle SDI	Male	Pancreatic cancer	2004		3.69	3.48	3.96
Middle SDI	Female	Pancreatic cancer	2004		3.23	2.97	3.49
Middle SDI	Both	Pancreatic cancer	2004		3.46	3.27	3.69
High SDI	Male	Pancreatic cancer	2005		11.18	10.76	11.42
High SDI	Female	Pancreatic cancer	2005		8.24	7.62	8.56
High SDI	Both	Pancreatic cancer	2005		9.62	9.07	9.90
High-middle SDI	Male	Pancreatic cancer	2005		8.90	8.52	9.24
High-middle SDI	Female	Pancreatic cancer	2005		5.83	5.44	6.08
High-middle SDI	Both	Pancreatic cancer	2005		7.23	6.90	7.49
Low SDI	Male	Pancreatic cancer	2005		2.00	1.56	2.43
Low SDI	Female	Pancreatic cancer	2005		1.63	1.34	2.02
Low SDI	Both	Pancreatic cancer	2005		1.81	1.50	2.14
Low-middle SDI	Male	Pancreatic cancer	2005		2.67	2.35	3.01
Low-middle SDI	Female	Pancreatic cancer	2005		2.29	2.09	2.52
Low-middle SDI	Both	Pancreatic cancer	2005		2.48	2.28	2.70
Middle SDI	Male	Pancreatic cancer	2005		3.72	3.49	4.00
Middle SDI	Female	Pancreatic cancer	2005		3.28	3.03	3.55
Middle SDI	Both	Pancreatic cancer	2005		3.51	3.30	3.73
High SDI	Male	Pancreatic cancer	2006		11.19	10.75	11.44
High SDI	Female	Pancreatic cancer	2006		8.28	7.64	8.62
High SDI	Both	Pancreatic cancer	2006		9.65	9.09	9.95
High-middle SDI	Male	Pancreatic cancer	2006		8.84	8.48	9.19
High-middle SDI	Female	Pancreatic cancer	2006		5.82	5.46	6.06
High-middle SDI	Both	Pancreatic cancer	2006		7.20	6.86	7.45
Low SDI	Male	Pancreatic cancer	2006		2.01	1.59	2.46
Low SDI	Female	Pancreatic cancer	2006		1.66	1.37	2.05
Low SDI	Both	Pancreatic cancer	2006		1.84	1.52	2.17
Low-middle SDI	Male	Pancreatic cancer	2006		2.76	2.44	3.10
Low-middle SDI	Female	Pancreatic cancer	2006		2.36	2.16	2.57
Low-middle SDI	Both	Pancreatic cancer	2006		2.56	2.34	2.77
Middle SDI	Male	Pancreatic cancer	2006		3.77	3.56	4.02
Middle SDI	Female	Pancreatic cancer	2006		3.33	3.08	3.59
Middle SDI	Both	Pancreatic cancer	2006		3.55	3.35	3.77
High SDI	Male	Pancreatic cancer	2007		11.31	10.85	11.58
High SDI	Female	Pancreatic cancer	2007		8.37	7.68	8.72

High SDI	Both	Pancreatic cancer	2007	9.76	9.16	10.06
High-middle SDI	Male	Pancreatic cancer	2007	8.91	8.54	9.28
High-middle SDI	Female	Pancreatic cancer	2007	5.89	5.50	6.15
High-middle SDI	Both	Pancreatic cancer	2007	7.28	6.93	7.55
Low SDI	Male	Pancreatic cancer	2007	2.04	1.62	2.48
Low SDI	Female	Pancreatic cancer	2007	1.69	1.41	2.06
Low SDI	Both	Pancreatic cancer	2007	1.87	1.55	2.19
Low-middle SDI	Male	Pancreatic cancer	2007	2.82	2.51	3.18
Low-middle SDI	Female	Pancreatic cancer	2007	2.41	2.21	2.62
Low-middle SDI	Both	Pancreatic cancer	2007	2.62	2.41	2.83
Middle SDI	Male	Pancreatic cancer	2007	3.89	3.66	4.17
Middle SDI	Female	Pancreatic cancer	2007	3.37	3.11	3.63
Middle SDI	Both	Pancreatic cancer	2007	3.63	3.44	3.86
High SDI	Male	Pancreatic cancer	2008	11.46	10.99	11.74
High SDI	Female	Pancreatic cancer	2008	8.51	7.80	8.88
High SDI	Both	Pancreatic cancer	2008	9.91	9.29	10.21
High-middle SDI	Male	Pancreatic cancer	2008	9.04	8.67	9.42
High-middle SDI	Female	Pancreatic cancer	2008	5.95	5.56	6.23
High-middle SDI	Both	Pancreatic cancer	2008	7.37	7.02	7.63
Low SDI	Male	Pancreatic cancer	2008	2.12	1.70	2.54
Low SDI	Female	Pancreatic cancer	2008	1.72	1.43	2.09
Low SDI	Both	Pancreatic cancer	2008	1.92	1.61	2.25
Low-middle SDI	Male	Pancreatic cancer	2008	2.90	2.59	3.24
Low-middle SDI	Female	Pancreatic cancer	2008	2.45	2.24	2.67
Low-middle SDI	Both	Pancreatic cancer	2008	2.68	2.47	2.87
Middle SDI	Male	Pancreatic cancer	2008	4.00	3.74	4.29
Middle SDI	Female	Pancreatic cancer	2008	3.43	3.17	3.69
Middle SDI	Both	Pancreatic cancer	2008	3.72	3.50	3.96
High SDI	Male	Pancreatic cancer	2009	11.59	11.09	11.87
High SDI	Female	Pancreatic cancer	2009	8.59	7.85	8.97
High SDI	Both	Pancreatic cancer	2009	10.01	9.35	10.34
High-middle SDI	Male	Pancreatic cancer	2009	9.07	8.66	9.45
High-middle SDI	Female	Pancreatic cancer	2009	6.00	5.60	6.27
High-middle SDI	Both	Pancreatic cancer	2009	7.41	7.05	7.69
Low SDI	Male	Pancreatic cancer	2009	2.15	1.74	2.58
Low SDI	Female	Pancreatic cancer	2009	1.77	1.46	2.13
Low SDI	Both	Pancreatic cancer	2009	1.96	1.65	2.28
Low-middle SDI	Male	Pancreatic cancer	2009	2.94	2.64	3.28
Low-middle SDI	Female	Pancreatic cancer	2009	2.49	2.28	2.70
Low-middle SDI	Both	Pancreatic cancer	2009	2.71	2.51	2.91
Middle SDI	Male	Pancreatic cancer	2009	4.12	3.86	4.42
Middle SDI	Female	Pancreatic cancer	2009	3.49	3.20	3.74
Middle SDI	Both	Pancreatic cancer	2009	3.80	3.56	4.05
High SDI	Male	Pancreatic cancer	2010	11.66	11.14	11.95
High SDI	Female	Pancreatic cancer	2010	8.63	7.87	9.03
High SDI	Both	Pancreatic cancer	2010	10.07	9.39	10.42
High-middle SDI	Male	Pancreatic cancer	2010	9.21	8.75	9.67
High-middle SDI	Female	Pancreatic cancer	2010	6.04	5.64	6.37
High-middle SDI	Both	Pancreatic cancer	2010	7.49	7.11	7.80
Low SDI	Male	Pancreatic cancer	2010	2.17	1.76	2.56
Low SDI	Female	Pancreatic cancer	2010	1.80	1.51	2.15
Low SDI	Both	Pancreatic cancer	2010	1.98	1.68	2.30

Low-middle SDI	Male	Pancreatic cancer	2010	2.98	2.69	3.29
Low-middle SDI	Female	Pancreatic cancer	2010	2.53	2.32	2.74
Low-middle SDI	Both	Pancreatic cancer	2010	2.76	2.56	2.96
Middle SDI	Male	Pancreatic cancer	2010	4.23	3.93	4.57
Middle SDI	Female	Pancreatic cancer	2010	3.56	3.27	3.84
Middle SDI	Both	Pancreatic cancer	2010	3.89	3.64	4.16
High SDI	Male	Pancreatic cancer	2011	11.70	11.18	12.01
High SDI	Female	Pancreatic cancer	2011	8.74	7.95	9.16
High SDI	Both	Pancreatic cancer	2011	10.15	9.46	10.51
High-middle SDI	Male	Pancreatic cancer	2011	9.18	8.70	9.66
High-middle SDI	Female	Pancreatic cancer	2011	6.04	5.62	6.36
High-middle SDI	Both	Pancreatic cancer	2011	7.48	7.09	7.81
Low SDI	Male	Pancreatic cancer	2011	2.20	1.80	2.58
Low SDI	Female	Pancreatic cancer	2011	1.84	1.55	2.18
Low SDI	Both	Pancreatic cancer	2011	2.02	1.72	2.32
Low-middle SDI	Male	Pancreatic cancer	2011	3.02	2.74	3.31
Low-middle SDI	Female	Pancreatic cancer	2011	2.59	2.38	2.80
Low-middle SDI	Both	Pancreatic cancer	2011	2.81	2.62	2.99
Middle SDI	Male	Pancreatic cancer	2011	4.30	3.99	4.64
Middle SDI	Female	Pancreatic cancer	2011	3.60	3.30	3.91
Middle SDI	Both	Pancreatic cancer	2011	3.95	3.67	4.23
High SDI	Male	Pancreatic cancer	2012	11.71	11.17	12.01
High SDI	Female	Pancreatic cancer	2012	8.73	7.92	9.15
High SDI	Both	Pancreatic cancer	2012	10.15	9.44	10.52
High-middle SDI	Male	Pancreatic cancer	2012	9.21	8.70	9.72
High-middle SDI	Female	Pancreatic cancer	2012	6.01	5.57	6.33
High-middle SDI	Both	Pancreatic cancer	2012	7.48	7.06	7.81
Low SDI	Male	Pancreatic cancer	2012	2.24	1.84	2.60
Low SDI	Female	Pancreatic cancer	2012	1.91	1.62	2.26
Low SDI	Both	Pancreatic cancer	2012	2.07	1.77	2.38
Low-middle SDI	Male	Pancreatic cancer	2012	3.10	2.84	3.39
Low-middle SDI	Female	Pancreatic cancer	2012	2.68	2.46	2.90
Low-middle SDI	Both	Pancreatic cancer	2012	2.89	2.70	3.08
Middle SDI	Male	Pancreatic cancer	2012	4.35	4.04	4.72
Middle SDI	Female	Pancreatic cancer	2012	3.64	3.32	3.93
Middle SDI	Both	Pancreatic cancer	2012	3.99	3.71	4.28
High SDI	Male	Pancreatic cancer	2013	11.74	11.15	12.07
High SDI	Female	Pancreatic cancer	2013	8.77	7.92	9.22
High SDI	Both	Pancreatic cancer	2013	10.18	9.44	10.57
High-middle SDI	Male	Pancreatic cancer	2013	9.14	8.64	9.65
High-middle SDI	Female	Pancreatic cancer	2013	5.97	5.55	6.29
High-middle SDI	Both	Pancreatic cancer	2013	7.43	7.01	7.76
Low SDI	Male	Pancreatic cancer	2013	2.29	1.90	2.64
Low SDI	Female	Pancreatic cancer	2013	1.98	1.68	2.31
Low SDI	Both	Pancreatic cancer	2013	2.13	1.83	2.44
Low-middle SDI	Male	Pancreatic cancer	2013	3.19	2.91	3.49
Low-middle SDI	Female	Pancreatic cancer	2013	2.78	2.55	3.00
Low-middle SDI	Both	Pancreatic cancer	2013	2.98	2.77	3.17
Middle SDI	Male	Pancreatic cancer	2013	4.39	4.08	4.79
Middle SDI	Female	Pancreatic cancer	2013	3.68	3.35	3.99
Middle SDI	Both	Pancreatic cancer	2013	4.03	3.77	4.34
High SDI	Male	Pancreatic cancer	2014	11.65	11.07	11.99

High SDI	Female	Pancreatic cancer	2014	8.77	7.92	9.22
High SDI	Both	Pancreatic cancer	2014	10.14	9.41	10.55
High-middle SDI	Male	Pancreatic cancer	2014	9.12	8.56	9.66
High-middle SDI	Female	Pancreatic cancer	2014	5.97	5.55	6.30
High-middle SDI	Both	Pancreatic cancer	2014	7.42	7.01	7.77
Low SDI	Male	Pancreatic cancer	2014	2.30	1.92	2.67
Low SDI	Female	Pancreatic cancer	2014	2.04	1.74	2.37
Low SDI	Both	Pancreatic cancer	2014	2.17	1.87	2.49
Low-middle SDI	Male	Pancreatic cancer	2014	3.26	2.99	3.54
Low-middle SDI	Female	Pancreatic cancer	2014	2.88	2.64	3.11
Low-middle SDI	Both	Pancreatic cancer	2014	3.07	2.86	3.27
Middle SDI	Male	Pancreatic cancer	2014	4.44	4.09	4.85
Middle SDI	Female	Pancreatic cancer	2014	3.74	3.41	4.09
Middle SDI	Both	Pancreatic cancer	2014	4.09	3.81	4.38
High SDI	Male	Pancreatic cancer	2015	11.72	11.13	12.07
High SDI	Female	Pancreatic cancer	2015	8.81	7.94	9.29
High SDI	Both	Pancreatic cancer	2015	10.21	9.45	10.62
High-middle SDI	Male	Pancreatic cancer	2015	9.23	8.65	9.84
High-middle SDI	Female	Pancreatic cancer	2015	6.04	5.60	6.39
High-middle SDI	Both	Pancreatic cancer	2015	7.51	7.08	7.87
Low SDI	Male	Pancreatic cancer	2015	2.34	1.97	2.69
Low SDI	Female	Pancreatic cancer	2015	2.10	1.79	2.42
Low SDI	Both	Pancreatic cancer	2015	2.22	1.92	2.51
Low-middle SDI	Male	Pancreatic cancer	2015	3.32	3.06	3.59
Low-middle SDI	Female	Pancreatic cancer	2015	3.01	2.75	3.24
Low-middle SDI	Both	Pancreatic cancer	2015	3.16	2.95	3.36
Middle SDI	Male	Pancreatic cancer	2015	4.48	4.11	4.90
Middle SDI	Female	Pancreatic cancer	2015	3.79	3.42	4.12
Middle SDI	Both	Pancreatic cancer	2015	4.13	3.82	4.44
High SDI	Male	Pancreatic cancer	2016	11.70	11.09	12.08
High SDI	Female	Pancreatic cancer	2016	8.87	8.00	9.37
High SDI	Both	Pancreatic cancer	2016	10.23	9.46	10.67
High-middle SDI	Male	Pancreatic cancer	2016	9.19	8.58	9.80
High-middle SDI	Female	Pancreatic cancer	2016	6.02	5.54	6.43
High-middle SDI	Both	Pancreatic cancer	2016	7.48	7.01	7.86
Low SDI	Male	Pancreatic cancer	2016	2.36	2.00	2.70
Low SDI	Female	Pancreatic cancer	2016	2.15	1.85	2.46
Low SDI	Both	Pancreatic cancer	2016	2.25	1.96	2.55
Low-middle SDI	Male	Pancreatic cancer	2016	3.39	3.12	3.67
Low-middle SDI	Female	Pancreatic cancer	2016	3.11	2.82	3.40
Low-middle SDI	Both	Pancreatic cancer	2016	3.25	3.02	3.48
Middle SDI	Male	Pancreatic cancer	2016	4.53	4.14	5.00
Middle SDI	Female	Pancreatic cancer	2016	3.84	3.45	4.20
Middle SDI	Both	Pancreatic cancer	2016	4.18	3.87	4.51
High SDI	Male	Pancreatic cancer	2017	11.58	10.85	12.22
High SDI	Female	Pancreatic cancer	2017	8.78	7.89	9.41
High SDI	Both	Pancreatic cancer	2017	10.12	9.30	10.75
High-middle SDI	Male	Pancreatic cancer	2017	9.15	8.44	9.89
High-middle SDI	Female	Pancreatic cancer	2017	6.04	5.48	6.55
High-middle SDI	Both	Pancreatic cancer	2017	7.48	6.94	7.94
Low SDI	Male	Pancreatic cancer	2017	2.39	2.03	2.74
Low SDI	Female	Pancreatic cancer	2017	2.20	1.90	2.50

Low SDI	Both	Pancreatic cancer	2017		2.30	2.00	2.58
Low-middle SDI	Male	Pancreatic cancer	2017		3.46	3.17	3.77
Low-middle SDI	Female	Pancreatic cancer	2017		3.18	2.86	3.50
Low-middle SDI	Both	Pancreatic cancer	2017		3.32	3.06	3.57
Middle SDI	Male	Pancreatic cancer	2017		4.63	4.15	5.16
Middle SDI	Female	Pancreatic cancer	2017		3.94	3.48	4.40
Middle SDI	Both	Pancreatic cancer	2017		4.28	3.92	4.69
High SDI	Male	Pancreatic cancer	2018		11.62	10.55	12.61
High SDI	Female	Pancreatic cancer	2018		8.78	7.82	9.67
High SDI	Both	Pancreatic cancer	2018		10.15	9.21	11.00
High-middle SDI	Male	Pancreatic cancer	2018		9.23	8.45	10.13
High-middle SDI	Female	Pancreatic cancer	2018		6.14	5.52	6.71
High-middle SDI	Both	Pancreatic cancer	2018		7.57	6.99	8.13
Low SDI	Male	Pancreatic cancer	2018		2.44	2.09	2.79
Low SDI	Female	Pancreatic cancer	2018		2.27	1.98	2.59
Low SDI	Both	Pancreatic cancer	2018		2.36	2.06	2.65
Low-middle SDI	Male	Pancreatic cancer	2018		3.55	3.25	3.86
Low-middle SDI	Female	Pancreatic cancer	2018		3.27	2.92	3.62
Low-middle SDI	Both	Pancreatic cancer	2018		3.41	3.15	3.66
Middle SDI	Male	Pancreatic cancer	2018		4.74	4.23	5.37
Middle SDI	Female	Pancreatic cancer	2018		4.07	3.57	4.59
Middle SDI	Both	Pancreatic cancer	2018		4.40	3.99	4.87
High SDI	Male	Pancreatic cancer	2019		11.60	10.43	12.86
High SDI	Female	Pancreatic cancer	2019		8.82	7.74	9.79
High SDI	Both	Pancreatic cancer	2019		10.16	9.12	11.11
High-middle SDI	Male	Pancreatic cancer	2019		9.33	8.40	10.33
High-middle SDI	Female	Pancreatic cancer	2019		6.22	5.52	6.92
High-middle SDI	Both	Pancreatic cancer	2019		7.66	6.97	8.33
Low SDI	Male	Pancreatic cancer	2019		2.48	2.11	2.82
Low SDI	Female	Pancreatic cancer	2019		2.33	2.03	2.65
Low SDI	Both	Pancreatic cancer	2019		2.40	2.11	2.70
Low-middle SDI	Male	Pancreatic cancer	2019		3.63	3.29	4.00
Low-middle SDI	Female	Pancreatic cancer	2019		3.36	2.98	3.74
Low-middle SDI	Both	Pancreatic cancer	2019		3.50	3.20	3.79
Middle SDI	Male	Pancreatic cancer	2019		4.84	4.25	5.49
Middle SDI	Female	Pancreatic cancer	2019		4.16	3.57	4.76
Middle SDI	Both	Pancreatic cancer	2019		4.49	4.01	5.01

sex_name	cause_name	year	Age-standardised death rate (per 100 000 person-years)	95% CI (lower)	95% CI (upper)
Male	Pancreatic cancer	1990	6.12	5.81	6.42
Female	Pancreatic cancer	1990	4.64	4.34	4.82
Both	Pancreatic cancer	1990	5.34	5.07	5.52
Male	Pancreatic cancer	1991	6.15	5.86	6.43
Female	Pancreatic cancer	1991	4.67	4.40	4.86
Both	Pancreatic cancer	1991	5.37	5.11	5.56
Male	Pancreatic cancer	1992	6.20	5.89	6.47
Female	Pancreatic cancer	1992	4.71	4.42	4.88
Both	Pancreatic cancer	1992	5.42	5.13	5.60
Male	Pancreatic cancer	1993	6.31	6.04	6.56
Female	Pancreatic cancer	1993	4.81	4.52	4.99
Both	Pancreatic cancer	1993	5.52	5.25	5.70
Male	Pancreatic cancer	1994	6.34	6.07	6.57
Female	Pancreatic cancer	1994	4.84	4.54	5.02
Both	Pancreatic cancer	1994	5.56	5.26	5.72
Male	Pancreatic cancer	1995	6.36	6.10	6.59
Female	Pancreatic cancer	1995	4.85	4.53	5.03
Both	Pancreatic cancer	1995	5.57	5.29	5.74
Male	Pancreatic cancer	1996	6.32	6.08	6.53
Female	Pancreatic cancer	1996	4.85	4.54	5.03
Both	Pancreatic cancer	1996	5.56	5.29	5.73
Male	Pancreatic cancer	1997	6.30	6.05	6.50
Female	Pancreatic cancer	1997	4.88	4.55	5.06
Both	Pancreatic cancer	1997	5.56	5.28	5.73
Male	Pancreatic cancer	1998	6.34	6.09	6.55
Female	Pancreatic cancer	1998	4.90	4.58	5.09
Both	Pancreatic cancer	1998	5.59	5.31	5.75
Male	Pancreatic cancer	1999	6.41	6.15	6.61
Female	Pancreatic cancer	1999	4.96	4.64	5.15
Both	Pancreatic cancer	1999	5.66	5.38	5.83
Male	Pancreatic cancer	2000	6.47	6.21	6.66
Female	Pancreatic cancer	2000	5.01	4.66	5.21
Both	Pancreatic cancer	2000	5.72	5.41	5.89
Male	Pancreatic cancer	2001	6.54	6.24	6.75
Female	Pancreatic cancer	2001	5.05	4.70	5.25
Both	Pancreatic cancer	2001	5.77	5.46	5.95
Male	Pancreatic cancer	2002	6.65	6.34	6.88
Female	Pancreatic cancer	2002	5.12	4.75	5.33
Both	Pancreatic cancer	2002	5.86	5.52	6.05
Male	Pancreatic cancer	2003	6.74	6.46	6.97
Female	Pancreatic cancer	2003	5.17	4.79	5.40
Both	Pancreatic cancer	2003	5.93	5.60	6.13
Male	Pancreatic cancer	2004	6.79	6.49	7.03
Female	Pancreatic cancer	2004	5.19	4.81	5.42
Both	Pancreatic cancer	2004	5.97	5.63	6.17
Male	Pancreatic cancer	2005	6.91	6.60	7.15
Female	Pancreatic cancer	2005	5.26	4.88	5.49
Both	Pancreatic cancer	2005	6.06	5.71	6.27

Male	Pancreatic cancer	2006	6.92	6.63	7.14
Female	Pancreatic cancer	2006	5.28	4.87	5.51
Both	Pancreatic cancer	2006	6.08	5.71	6.28
Male	Pancreatic cancer	2007	6.99	6.67	7.22
Female	Pancreatic cancer	2007	5.33	4.91	5.58
Both	Pancreatic cancer	2007	6.13	5.77	6.36
Male	Pancreatic cancer	2008	7.10	6.78	7.34
Female	Pancreatic cancer	2008	5.39	4.96	5.64
Both	Pancreatic cancer	2008	6.21	5.84	6.45
Male	Pancreatic cancer	2009	7.17	6.82	7.42
Female	Pancreatic cancer	2009	5.42	4.99	5.69
Both	Pancreatic cancer	2009	6.26	5.86	6.49
Male	Pancreatic cancer	2010	7.27	6.91	7.54
Female	Pancreatic cancer	2010	5.46	5.01	5.75
Both	Pancreatic cancer	2010	6.33	5.93	6.58
Male	Pancreatic cancer	2011	7.30	6.93	7.61
Female	Pancreatic cancer	2011	5.49	5.02	5.79
Both	Pancreatic cancer	2011	6.36	5.93	6.64
Male	Pancreatic cancer	2012	7.34	6.98	7.67
Female	Pancreatic cancer	2012	5.51	5.04	5.79
Both	Pancreatic cancer	2012	6.39	5.97	6.66
Male	Pancreatic cancer	2013	7.36	6.96	7.68
Female	Pancreatic cancer	2013	5.52	5.02	5.80
Both	Pancreatic cancer	2013	6.40	5.96	6.67
Male	Pancreatic cancer	2014	7.36	6.98	7.69
Female	Pancreatic cancer	2014	5.54	5.04	5.86
Both	Pancreatic cancer	2014	6.41	5.97	6.69
Male	Pancreatic cancer	2015	7.41	7.02	7.76
Female	Pancreatic cancer	2015	5.59	5.07	5.91
Both	Pancreatic cancer	2015	6.46	6.01	6.76
Male	Pancreatic cancer	2016	7.42	6.97	7.82
Female	Pancreatic cancer	2016	5.61	5.10	5.98
Both	Pancreatic cancer	2016	6.47	6.00	6.81
Male	Pancreatic cancer	2017	7.41	6.95	7.85
Female	Pancreatic cancer	2017	5.62	5.09	6.00
Both	Pancreatic cancer	2017	6.48	6.04	6.82
Male	Pancreatic cancer	2018	7.49	7.00	7.98
Female	Pancreatic cancer	2018	5.70	5.14	6.11
Both	Pancreatic cancer	2018	6.55	6.07	6.97
Male	Pancreatic cancer	2019	7.55	7.01	8.09
Female	Pancreatic cancer	2019	5.77	5.15	6.24
Both	Pancreatic cancer	2019	6.62	6.11	7.06

location_name	sex_name	cause_name	year	Age-standardised death rate (per 100 000 person-years)	95% CI (lower)	95% CI (upper)
High SDI	Male	Pancreatic cancer	1990	10.27	9.94	10.46
High SDI	Female	Pancreatic cancer	1990	7.15	6.71	7.37
High SDI	Both	Pancreatic cancer	1990	8.52	8.12	8.72
High-middle SDI	Male	Pancreatic cancer	1990	7.82	7.42	8.20
High-middle SDI	Female	Pancreatic cancer	1990	5.22	4.92	5.48
High-middle SDI	Both	Pancreatic cancer	1990	6.37	6.09	6.61
Low SDI	Male	Pancreatic cancer	1990	2.11	1.55	2.65
Low SDI	Female	Pancreatic cancer	1990	1.30	1.04	1.67
Low SDI	Both	Pancreatic cancer	1990	1.71	1.35	2.04
Low-middle SDI	Male	Pancreatic cancer	1990	2.18	1.73	2.64
Low-middle SDI	Female	Pancreatic cancer	1990	1.65	1.43	1.95
Low-middle SDI	Both	Pancreatic cancer	1990	1.91	1.62	2.18
Middle SDI	Male	Pancreatic cancer	1990	3.24	2.92	3.59
Middle SDI	Female	Pancreatic cancer	1990	2.53	2.34	2.72
Middle SDI	Both	Pancreatic cancer	1990	2.87	2.68	3.06
High SDI	Male	Pancreatic cancer	1991	10.29	9.96	10.48
High SDI	Female	Pancreatic cancer	1991	7.18	6.74	7.40
High SDI	Both	Pancreatic cancer	1991	8.56	8.16	8.75
High-middle SDI	Male	Pancreatic cancer	1991	7.90	7.52	8.28
High-middle SDI	Female	Pancreatic cancer	1991	5.29	4.99	5.57
High-middle SDI	Both	Pancreatic cancer	1991	6.45	6.15	6.68
Low SDI	Male	Pancreatic cancer	1991	2.12	1.57	2.67
Low SDI	Female	Pancreatic cancer	1991	1.31	1.04	1.69
Low SDI	Both	Pancreatic cancer	1991	1.72	1.37	2.05
Low-middle SDI	Male	Pancreatic cancer	1991	2.19	1.74	2.67
Low-middle SDI	Female	Pancreatic cancer	1991	1.66	1.44	1.97
Low-middle SDI	Both	Pancreatic cancer	1991	1.93	1.64	2.20
Middle SDI	Male	Pancreatic cancer	1991	3.28	2.98	3.62
Middle SDI	Female	Pancreatic cancer	1991	2.58	2.38	2.79
Middle SDI	Both	Pancreatic cancer	1991	2.92	2.74	3.10
High SDI	Male	Pancreatic cancer	1992	10.33	10.00	10.53
High SDI	Female	Pancreatic cancer	1992	7.22	6.77	7.44
High SDI	Both	Pancreatic cancer	1992	8.60	8.18	8.80
High-middle SDI	Male	Pancreatic cancer	1992	8.06	7.71	8.40
High-middle SDI	Female	Pancreatic cancer	1992	5.36	5.07	5.60
High-middle SDI	Both	Pancreatic cancer	1992	6.56	6.28	6.79
Low SDI	Male	Pancreatic cancer	1992	2.13	1.58	2.68
Low SDI	Female	Pancreatic cancer	1992	1.32	1.06	1.71
Low SDI	Both	Pancreatic cancer	1992	1.73	1.38	2.07
Low-middle SDI	Male	Pancreatic cancer	1992	2.21	1.76	2.70
Low-middle SDI	Female	Pancreatic cancer	1992	1.69	1.47	2.00
Low-middle SDI	Both	Pancreatic cancer	1992	1.95	1.66	2.23
Middle SDI	Male	Pancreatic cancer	1992	3.30	3.03	3.60
Middle SDI	Female	Pancreatic cancer	1992	2.62	2.42	2.82
Middle SDI	Both	Pancreatic cancer	1992	2.96	2.77	3.12
High SDI	Male	Pancreatic cancer	1993	10.45	10.12	10.64
High SDI	Female	Pancreatic cancer	1993	7.32	6.85	7.55
High SDI	Both	Pancreatic cancer	1993	8.71	8.29	8.92
High-middle SDI	Male	Pancreatic cancer	1993	8.34	7.99	8.67

High-middle SDI	Female	Pancreatic cancer	1993		5.55	5.26	5.79
High-middle SDI	Both	Pancreatic cancer	1993		6.80	6.52	7.01
Low SDI	Male	Pancreatic cancer	1993		2.15	1.60	2.69
Low SDI	Female	Pancreatic cancer	1993		1.34	1.07	1.71
Low SDI	Both	Pancreatic cancer	1993		1.75	1.40	2.09
Low-middle SDI	Male	Pancreatic cancer	1993		2.26	1.83	2.72
Low-middle SDI	Female	Pancreatic cancer	1993		1.74	1.52	2.03
Low-middle SDI	Both	Pancreatic cancer	1993		2.00	1.73	2.26
Middle SDI	Male	Pancreatic cancer	1993		3.36	3.10	3.63
Middle SDI	Female	Pancreatic cancer	1993		2.70	2.51	2.92
Middle SDI	Both	Pancreatic cancer	1993		3.02	2.86	3.19
High SDI	Male	Pancreatic cancer	1994		10.44	10.09	10.64
High SDI	Female	Pancreatic cancer	1994		7.33	6.85	7.57
High SDI	Both	Pancreatic cancer	1994		8.72	8.30	8.95
High-middle SDI	Male	Pancreatic cancer	1994		8.45	8.11	8.78
High-middle SDI	Female	Pancreatic cancer	1994		5.63	5.32	5.87
High-middle SDI	Both	Pancreatic cancer	1994		6.90	6.64	7.10
Low SDI	Male	Pancreatic cancer	1994		2.17	1.61	2.71
Low SDI	Female	Pancreatic cancer	1994		1.36	1.09	1.73
Low SDI	Both	Pancreatic cancer	1994		1.77	1.42	2.10
Low-middle SDI	Male	Pancreatic cancer	1994		2.30	1.89	2.75
Low-middle SDI	Female	Pancreatic cancer	1994		1.79	1.58	2.07
Low-middle SDI	Both	Pancreatic cancer	1994		2.05	1.78	2.30
Middle SDI	Male	Pancreatic cancer	1994		3.39	3.16	3.63
Middle SDI	Female	Pancreatic cancer	1994		2.75	2.55	2.95
Middle SDI	Both	Pancreatic cancer	1994		3.06	2.90	3.23
High SDI	Male	Pancreatic cancer	1995		10.49	10.13	10.70
High SDI	Female	Pancreatic cancer	1995		7.37	6.89	7.62
High SDI	Both	Pancreatic cancer	1995		8.77	8.32	9.00
High-middle SDI	Male	Pancreatic cancer	1995		8.39	8.09	8.70
High-middle SDI	Female	Pancreatic cancer	1995		5.58	5.27	5.79
High-middle SDI	Both	Pancreatic cancer	1995		6.85	6.59	7.05
Low SDI	Male	Pancreatic cancer	1995		2.19	1.64	2.72
Low SDI	Female	Pancreatic cancer	1995		1.37	1.11	1.75
Low SDI	Both	Pancreatic cancer	1995		1.78	1.43	2.12
Low-middle SDI	Male	Pancreatic cancer	1995		2.34	1.94	2.78
Low-middle SDI	Female	Pancreatic cancer	1995		1.83	1.62	2.12
Low-middle SDI	Both	Pancreatic cancer	1995		2.09	1.82	2.33
Middle SDI	Male	Pancreatic cancer	1995		3.46	3.25	3.69
Middle SDI	Female	Pancreatic cancer	1995		2.78	2.58	3.01
Middle SDI	Both	Pancreatic cancer	1995		3.12	2.95	3.29
High SDI	Male	Pancreatic cancer	1996		10.41	10.04	10.62
High SDI	Female	Pancreatic cancer	1996		7.37	6.87	7.62
High SDI	Both	Pancreatic cancer	1996		8.74	8.29	8.98
High-middle SDI	Male	Pancreatic cancer	1996		8.24	7.96	8.50
High-middle SDI	Female	Pancreatic cancer	1996		5.54	5.26	5.75
High-middle SDI	Both	Pancreatic cancer	1996		6.75	6.48	6.95
Low SDI	Male	Pancreatic cancer	1996		2.21	1.66	2.74
Low SDI	Female	Pancreatic cancer	1996		1.40	1.14	1.77
Low SDI	Both	Pancreatic cancer	1996		1.81	1.46	2.14
Low-middle SDI	Male	Pancreatic cancer	1996		2.40	2.01	2.82

Low-middle SDI	Female	Pancreatic cancer	1996		1.90	1.68	2.18
Low-middle SDI	Both	Pancreatic cancer	1996		2.15	1.89	2.39
Middle SDI	Male	Pancreatic cancer	1996		3.55	3.35	3.78
Middle SDI	Female	Pancreatic cancer	1996		2.88	2.67	3.09
Middle SDI	Both	Pancreatic cancer	1996		3.20	3.03	3.37
High SDI	Male	Pancreatic cancer	1997		10.33	9.96	10.54
High SDI	Female	Pancreatic cancer	1997		7.37	6.89	7.63
High SDI	Both	Pancreatic cancer	1997		8.72	8.26	8.95
High-middle SDI	Male	Pancreatic cancer	1997		8.12	7.81	8.39
High-middle SDI	Female	Pancreatic cancer	1997		5.53	5.24	5.73
High-middle SDI	Both	Pancreatic cancer	1997		6.70	6.41	6.88
Low SDI	Male	Pancreatic cancer	1997		2.24	1.71	2.79
Low SDI	Female	Pancreatic cancer	1997		1.43	1.16	1.82
Low SDI	Both	Pancreatic cancer	1997		1.84	1.49	2.19
Low-middle SDI	Male	Pancreatic cancer	1997		2.49	2.11	2.93
Low-middle SDI	Female	Pancreatic cancer	1997		1.98	1.78	2.25
Low-middle SDI	Both	Pancreatic cancer	1997		2.24	1.98	2.48
Middle SDI	Male	Pancreatic cancer	1997		3.62	3.42	3.82
Middle SDI	Female	Pancreatic cancer	1997		2.96	2.74	3.19
Middle SDI	Both	Pancreatic cancer	1997		3.28	3.12	3.46
High SDI	Male	Pancreatic cancer	1998		10.41	10.04	10.61
High SDI	Female	Pancreatic cancer	1998		7.43	6.93	7.69
High SDI	Both	Pancreatic cancer	1998		8.78	8.33	9.02
High-middle SDI	Male	Pancreatic cancer	1998		8.09	7.79	8.38
High-middle SDI	Female	Pancreatic cancer	1998		5.51	5.24	5.71
High-middle SDI	Both	Pancreatic cancer	1998		6.67	6.41	6.87
Low SDI	Male	Pancreatic cancer	1998		2.28	1.74	2.80
Low SDI	Female	Pancreatic cancer	1998		1.47	1.20	1.86
Low SDI	Both	Pancreatic cancer	1998		1.87	1.53	2.20
Low-middle SDI	Male	Pancreatic cancer	1998		2.56	2.17	2.98
Low-middle SDI	Female	Pancreatic cancer	1998		2.04	1.83	2.30
Low-middle SDI	Both	Pancreatic cancer	1998		2.30	2.04	2.54
Middle SDI	Male	Pancreatic cancer	1998		3.71	3.51	3.96
Middle SDI	Female	Pancreatic cancer	1998		3.02	2.80	3.25
Middle SDI	Both	Pancreatic cancer	1998		3.36	3.17	3.56
High SDI	Male	Pancreatic cancer	1999		10.48	10.12	10.69
High SDI	Female	Pancreatic cancer	1999		7.53	7.01	7.79
High SDI	Both	Pancreatic cancer	1999		8.88	8.41	9.12
High-middle SDI	Male	Pancreatic cancer	1999		8.20	7.93	8.48
High-middle SDI	Female	Pancreatic cancer	1999		5.58	5.27	5.78
High-middle SDI	Both	Pancreatic cancer	1999		6.76	6.49	6.95
Low SDI	Male	Pancreatic cancer	1999		2.29	1.75	2.82
Low SDI	Female	Pancreatic cancer	1999		1.49	1.22	1.88
Low SDI	Both	Pancreatic cancer	1999		1.89	1.54	2.24
Low-middle SDI	Male	Pancreatic cancer	1999		2.58	2.20	3.00
Low-middle SDI	Female	Pancreatic cancer	1999		2.09	1.88	2.35
Low-middle SDI	Both	Pancreatic cancer	1999		2.33	2.08	2.58
Middle SDI	Male	Pancreatic cancer	1999		3.80	3.57	4.03
Middle SDI	Female	Pancreatic cancer	1999		3.09	2.84	3.35
Middle SDI	Both	Pancreatic cancer	1999		3.44	3.24	3.64
High SDI	Male	Pancreatic cancer	2000		10.46	10.09	10.68

High SDI	Female	Pancreatic cancer	2000		7.56	7.03	7.82
High SDI	Both	Pancreatic cancer	2000		8.89	8.42	9.14
High-middle SDI	Male	Pancreatic cancer	2000		8.32	8.00	8.60
High-middle SDI	Female	Pancreatic cancer	2000		5.64	5.33	5.88
High-middle SDI	Both	Pancreatic cancer	2000		6.85	6.56	7.06
Low SDI	Male	Pancreatic cancer	2000		2.32	1.78	2.86
Low SDI	Female	Pancreatic cancer	2000		1.52	1.24	1.92
Low SDI	Both	Pancreatic cancer	2000		1.92	1.57	2.28
Low-middle SDI	Male	Pancreatic cancer	2000		2.65	2.27	3.07
Low-middle SDI	Female	Pancreatic cancer	2000		2.15	1.93	2.41
Low-middle SDI	Both	Pancreatic cancer	2000		2.40	2.15	2.64
Middle SDI	Male	Pancreatic cancer	2000		3.91	3.68	4.18
Middle SDI	Female	Pancreatic cancer	2000		3.17	2.93	3.44
Middle SDI	Both	Pancreatic cancer	2000		3.54	3.33	3.77
High SDI	Male	Pancreatic cancer	2001		10.46	10.08	10.69
High SDI	Female	Pancreatic cancer	2001		7.59	7.05	7.87
High SDI	Both	Pancreatic cancer	2001		8.92	8.43	9.17
High-middle SDI	Male	Pancreatic cancer	2001		8.42	8.10	8.76
High-middle SDI	Female	Pancreatic cancer	2001		5.70	5.37	5.94
High-middle SDI	Both	Pancreatic cancer	2001		6.93	6.62	7.15
Low SDI	Male	Pancreatic cancer	2001		2.36	1.81	2.91
Low SDI	Female	Pancreatic cancer	2001		1.57	1.28	1.97
Low SDI	Both	Pancreatic cancer	2001		1.96	1.60	2.33
Low-middle SDI	Male	Pancreatic cancer	2001		2.73	2.35	3.14
Low-middle SDI	Female	Pancreatic cancer	2001		2.23	2.02	2.48
Low-middle SDI	Both	Pancreatic cancer	2001		2.48	2.24	2.71
Middle SDI	Male	Pancreatic cancer	2001		4.02	3.77	4.31
Middle SDI	Female	Pancreatic cancer	2001		3.24	3.00	3.49
Middle SDI	Both	Pancreatic cancer	2001		3.62	3.42	3.84
High SDI	Male	Pancreatic cancer	2002		10.54	10.14	10.77
High SDI	Female	Pancreatic cancer	2002		7.65	7.09	7.94
High SDI	Both	Pancreatic cancer	2002		8.99	8.48	9.25
High-middle SDI	Male	Pancreatic cancer	2002		8.61	8.25	8.95
High-middle SDI	Female	Pancreatic cancer	2002		5.80	5.46	6.03
High-middle SDI	Both	Pancreatic cancer	2002		7.07	6.74	7.30
Low SDI	Male	Pancreatic cancer	2002		2.40	1.85	2.94
Low SDI	Female	Pancreatic cancer	2002		1.62	1.33	2.03
Low SDI	Both	Pancreatic cancer	2002		2.01	1.64	2.38
Low-middle SDI	Male	Pancreatic cancer	2002		2.79	2.42	3.21
Low-middle SDI	Female	Pancreatic cancer	2002		2.32	2.10	2.58
Low-middle SDI	Both	Pancreatic cancer	2002		2.55	2.31	2.79
Middle SDI	Male	Pancreatic cancer	2002		4.15	3.89	4.44
Middle SDI	Female	Pancreatic cancer	2002		3.31	3.05	3.59
Middle SDI	Both	Pancreatic cancer	2002		3.73	3.50	3.99
High SDI	Male	Pancreatic cancer	2003		10.61	10.20	10.85
High SDI	Female	Pancreatic cancer	2003		7.71	7.12	8.00
High SDI	Both	Pancreatic cancer	2003		9.05	8.53	9.32
High-middle SDI	Male	Pancreatic cancer	2003		8.79	8.44	9.11
High-middle SDI	Female	Pancreatic cancer	2003		5.90	5.53	6.15
High-middle SDI	Both	Pancreatic cancer	2003		7.21	6.88	7.44
Low SDI	Male	Pancreatic cancer	2003		2.44	1.89	3.00

Low SDI	Female	Pancreatic cancer	2003		1.66	1.37	2.07
Low SDI	Both	Pancreatic cancer	2003		2.05	1.67	2.42
Low-middle SDI	Male	Pancreatic cancer	2003		2.84	2.47	3.24
Low-middle SDI	Female	Pancreatic cancer	2003		2.35	2.13	2.60
Low-middle SDI	Both	Pancreatic cancer	2003		2.59	2.35	2.82
Middle SDI	Male	Pancreatic cancer	2003		4.25	3.99	4.53
Middle SDI	Female	Pancreatic cancer	2003		3.38	3.11	3.65
Middle SDI	Both	Pancreatic cancer	2003		3.81	3.60	4.05
High SDI	Male	Pancreatic cancer	2004		10.59	10.16	10.83
High SDI	Female	Pancreatic cancer	2004		7.72	7.13	8.02
High SDI	Both	Pancreatic cancer	2004		9.05	8.52	9.33
High-middle SDI	Male	Pancreatic cancer	2004		8.90	8.53	9.25
High-middle SDI	Female	Pancreatic cancer	2004		5.93	5.56	6.18
High-middle SDI	Both	Pancreatic cancer	2004		7.28	6.94	7.53
Low SDI	Male	Pancreatic cancer	2004		2.46	1.91	2.99
Low SDI	Female	Pancreatic cancer	2004		1.70	1.39	2.12
Low SDI	Both	Pancreatic cancer	2004		2.08	1.71	2.45
Low-middle SDI	Male	Pancreatic cancer	2004		2.85	2.49	3.22
Low-middle SDI	Female	Pancreatic cancer	2004		2.38	2.15	2.62
Low-middle SDI	Both	Pancreatic cancer	2004		2.61	2.36	2.84
Middle SDI	Male	Pancreatic cancer	2004		4.36	4.09	4.67
Middle SDI	Female	Pancreatic cancer	2004		3.44	3.16	3.72
Middle SDI	Both	Pancreatic cancer	2004		3.89	3.66	4.14
High SDI	Male	Pancreatic cancer	2005		10.69	10.26	10.94
High SDI	Female	Pancreatic cancer	2005		7.80	7.19	8.12
High SDI	Both	Pancreatic cancer	2005		9.15	8.60	9.43
High-middle SDI	Male	Pancreatic cancer	2005		9.12	8.77	9.49
High-middle SDI	Female	Pancreatic cancer	2005		6.03	5.64	6.30
High-middle SDI	Both	Pancreatic cancer	2005		7.44	7.08	7.70
Low SDI	Male	Pancreatic cancer	2005		2.49	1.95	3.00
Low SDI	Female	Pancreatic cancer	2005		1.75	1.45	2.18
Low SDI	Both	Pancreatic cancer	2005		2.12	1.73	2.49
Low-middle SDI	Male	Pancreatic cancer	2005		2.93	2.58	3.31
Low-middle SDI	Female	Pancreatic cancer	2005		2.45	2.23	2.70
Low-middle SDI	Both	Pancreatic cancer	2005		2.69	2.45	2.92
Middle SDI	Male	Pancreatic cancer	2005		4.46	4.18	4.78
Middle SDI	Female	Pancreatic cancer	2005		3.50	3.23	3.77
Middle SDI	Both	Pancreatic cancer	2005		3.97	3.73	4.23
High SDI	Male	Pancreatic cancer	2006		10.71	10.27	10.96
High SDI	Female	Pancreatic cancer	2006		7.84	7.19	8.17
High SDI	Both	Pancreatic cancer	2006		9.18	8.62	9.48
High-middle SDI	Male	Pancreatic cancer	2006		9.06	8.69	9.40
High-middle SDI	Female	Pancreatic cancer	2006		6.03	5.63	6.29
High-middle SDI	Both	Pancreatic cancer	2006		7.41	7.04	7.66
Low SDI	Male	Pancreatic cancer	2006		2.51	1.99	3.02
Low SDI	Female	Pancreatic cancer	2006		1.79	1.48	2.22
Low SDI	Both	Pancreatic cancer	2006		2.15	1.77	2.52
Low-middle SDI	Male	Pancreatic cancer	2006		3.02	2.68	3.39
Low-middle SDI	Female	Pancreatic cancer	2006		2.52	2.30	2.76
Low-middle SDI	Both	Pancreatic cancer	2006		2.77	2.54	2.99
Middle SDI	Male	Pancreatic cancer	2006		4.51	4.23	4.81

Middle SDI	Female	Pancreatic cancer	2006	3.55	3.28	3.81
Middle SDI	Both	Pancreatic cancer	2006	4.02	3.79	4.26
High SDI	Male	Pancreatic cancer	2007	10.80	10.34	11.07
High SDI	Female	Pancreatic cancer	2007	7.91	7.23	8.26
High SDI	Both	Pancreatic cancer	2007	9.27	8.67	9.58
High-middle SDI	Male	Pancreatic cancer	2007	9.12	8.71	9.47
High-middle SDI	Female	Pancreatic cancer	2007	6.09	5.68	6.38
High-middle SDI	Both	Pancreatic cancer	2007	7.47	7.08	7.74
Low SDI	Male	Pancreatic cancer	2007	2.55	2.03	3.04
Low SDI	Female	Pancreatic cancer	2007	1.82	1.51	2.24
Low SDI	Both	Pancreatic cancer	2007	2.18	1.80	2.55
Low-middle SDI	Male	Pancreatic cancer	2007	3.09	2.75	3.47
Low-middle SDI	Female	Pancreatic cancer	2007	2.58	2.36	2.84
Low-middle SDI	Both	Pancreatic cancer	2007	2.83	2.61	3.07
Middle SDI	Male	Pancreatic cancer	2007	4.58	4.29	4.90
Middle SDI	Female	Pancreatic cancer	2007	3.59	3.33	3.86
Middle SDI	Both	Pancreatic cancer	2007	4.08	3.82	4.34
High SDI	Male	Pancreatic cancer	2008	10.93	10.45	11.21
High SDI	Female	Pancreatic cancer	2008	8.01	7.31	8.38
High SDI	Both	Pancreatic cancer	2008	9.38	8.78	9.70
High-middle SDI	Male	Pancreatic cancer	2008	9.25	8.81	9.62
High-middle SDI	Female	Pancreatic cancer	2008	6.15	5.72	6.44
High-middle SDI	Both	Pancreatic cancer	2008	7.56	7.17	7.84
Low SDI	Male	Pancreatic cancer	2008	2.57	2.06	3.05
Low SDI	Female	Pancreatic cancer	2008	1.86	1.54	2.27
Low SDI	Both	Pancreatic cancer	2008	2.21	1.84	2.57
Low-middle SDI	Male	Pancreatic cancer	2008	3.17	2.83	3.53
Low-middle SDI	Female	Pancreatic cancer	2008	2.63	2.41	2.87
Low-middle SDI	Both	Pancreatic cancer	2008	2.90	2.67	3.13
Middle SDI	Male	Pancreatic cancer	2008	4.71	4.40	5.03
Middle SDI	Female	Pancreatic cancer	2008	3.65	3.35	3.92
Middle SDI	Both	Pancreatic cancer	2008	4.17	3.90	4.43
High SDI	Male	Pancreatic cancer	2009	11.03	10.52	11.30
High SDI	Female	Pancreatic cancer	2009	8.07	7.35	8.43
High SDI	Both	Pancreatic cancer	2009	9.46	8.83	9.78
High-middle SDI	Male	Pancreatic cancer	2009	9.28	8.85	9.69
High-middle SDI	Female	Pancreatic cancer	2009	6.18	5.74	6.50
High-middle SDI	Both	Pancreatic cancer	2009	7.59	7.19	7.88
Low SDI	Male	Pancreatic cancer	2009	2.60	2.09	3.06
Low SDI	Female	Pancreatic cancer	2009	1.90	1.58	2.31
Low SDI	Both	Pancreatic cancer	2009	2.24	1.88	2.61
Low-middle SDI	Male	Pancreatic cancer	2009	3.21	2.88	3.55
Low-middle SDI	Female	Pancreatic cancer	2009	2.66	2.43	2.91
Low-middle SDI	Both	Pancreatic cancer	2009	2.93	2.71	3.15
Middle SDI	Male	Pancreatic cancer	2009	4.84	4.53	5.21
Middle SDI	Female	Pancreatic cancer	2009	3.71	3.41	4.01
Middle SDI	Both	Pancreatic cancer	2009	4.27	4.00	4.54
High SDI	Male	Pancreatic cancer	2010	11.10	10.60	11.39
High SDI	Female	Pancreatic cancer	2010	8.12	7.40	8.52
High SDI	Both	Pancreatic cancer	2010	9.52	8.87	9.88
High-middle SDI	Male	Pancreatic cancer	2010	9.42	8.95	9.90

High-middle SDI	Female	Pancreatic cancer	2010		6.24	5.79	6.58
High-middle SDI	Both	Pancreatic cancer	2010		7.69	7.27	8.00
Low SDI	Male	Pancreatic cancer	2010		2.62	2.12	3.07
Low SDI	Female	Pancreatic cancer	2010		1.93	1.60	2.33
Low SDI	Both	Pancreatic cancer	2010		2.27	1.92	2.64
Low-middle SDI	Male	Pancreatic cancer	2010		3.25	2.94	3.57
Low-middle SDI	Female	Pancreatic cancer	2010		2.71	2.48	2.95
Low-middle SDI	Both	Pancreatic cancer	2010		2.98	2.76	3.19
Middle SDI	Male	Pancreatic cancer	2010		5.00	4.65	5.42
Middle SDI	Female	Pancreatic cancer	2010		3.78	3.45	4.10
Middle SDI	Both	Pancreatic cancer	2010		4.38	4.08	4.68
High SDI	Male	Pancreatic cancer	2011		11.16	10.63	11.46
High SDI	Female	Pancreatic cancer	2011		8.20	7.44	8.60
High SDI	Both	Pancreatic cancer	2011		9.59	8.93	9.95
High-middle SDI	Male	Pancreatic cancer	2011		9.39	8.85	9.93
High-middle SDI	Female	Pancreatic cancer	2011		6.22	5.74	6.59
High-middle SDI	Both	Pancreatic cancer	2011		7.67	7.19	8.02
Low SDI	Male	Pancreatic cancer	2011		2.66	2.16	3.08
Low SDI	Female	Pancreatic cancer	2011		1.98	1.66	2.36
Low SDI	Both	Pancreatic cancer	2011		2.31	1.95	2.67
Low-middle SDI	Male	Pancreatic cancer	2011		3.29	2.99	3.58
Low-middle SDI	Female	Pancreatic cancer	2011		2.78	2.53	3.01
Low-middle SDI	Both	Pancreatic cancer	2011		3.03	2.81	3.22
Middle SDI	Male	Pancreatic cancer	2011		5.08	4.69	5.55
Middle SDI	Female	Pancreatic cancer	2011		3.83	3.46	4.15
Middle SDI	Both	Pancreatic cancer	2011		4.44	4.09	4.78
High SDI	Male	Pancreatic cancer	2012		11.18	10.63	11.50
High SDI	Female	Pancreatic cancer	2012		8.23	7.44	8.64
High SDI	Both	Pancreatic cancer	2012		9.62	8.93	9.99
High-middle SDI	Male	Pancreatic cancer	2012		9.41	8.94	9.94
High-middle SDI	Female	Pancreatic cancer	2012		6.20	5.73	6.54
High-middle SDI	Both	Pancreatic cancer	2012		7.66	7.23	8.00
Low SDI	Male	Pancreatic cancer	2012		2.69	2.21	3.11
Low SDI	Female	Pancreatic cancer	2012		2.05	1.74	2.45
Low SDI	Both	Pancreatic cancer	2012		2.36	2.01	2.72
Low-middle SDI	Male	Pancreatic cancer	2012		3.37	3.07	3.66
Low-middle SDI	Female	Pancreatic cancer	2012		2.87	2.64	3.11
Low-middle SDI	Both	Pancreatic cancer	2012		3.12	2.91	3.32
Middle SDI	Male	Pancreatic cancer	2012		5.15	4.77	5.63
Middle SDI	Female	Pancreatic cancer	2012		3.86	3.52	4.17
Middle SDI	Both	Pancreatic cancer	2012		4.49	4.18	4.83
High SDI	Male	Pancreatic cancer	2013		11.19	10.62	11.51
High SDI	Female	Pancreatic cancer	2013		8.25	7.44	8.67
High SDI	Both	Pancreatic cancer	2013		9.64	8.93	10.02
High-middle SDI	Male	Pancreatic cancer	2013		9.34	8.81	9.87
High-middle SDI	Female	Pancreatic cancer	2013		6.16	5.68	6.49
High-middle SDI	Both	Pancreatic cancer	2013		7.61	7.13	7.95
Low SDI	Male	Pancreatic cancer	2013		2.74	2.29	3.16
Low SDI	Female	Pancreatic cancer	2013		2.12	1.79	2.49
Low SDI	Both	Pancreatic cancer	2013		2.43	2.08	2.79
Low-middle SDI	Male	Pancreatic cancer	2013		3.47	3.17	3.77

Low-middle SDI	Female	Pancreatic cancer	2013	2.97	2.72	3.21
Low-middle SDI	Both	Pancreatic cancer	2013	3.21	3.00	3.42
Middle SDI	Male	Pancreatic cancer	2013	5.21	4.79	5.70
Middle SDI	Female	Pancreatic cancer	2013	3.91	3.54	4.23
Middle SDI	Both	Pancreatic cancer	2013	4.54	4.20	4.87
High SDI	Male	Pancreatic cancer	2014	11.13	10.55	11.47
High SDI	Female	Pancreatic cancer	2014	8.24	7.40	8.67
High SDI	Both	Pancreatic cancer	2014	9.61	8.88	10.01
High-middle SDI	Male	Pancreatic cancer	2014	9.32	8.79	9.90
High-middle SDI	Female	Pancreatic cancer	2014	6.16	5.69	6.52
High-middle SDI	Both	Pancreatic cancer	2014	7.61	7.16	7.97
Low SDI	Male	Pancreatic cancer	2014	2.75	2.30	3.17
Low SDI	Female	Pancreatic cancer	2014	2.19	1.86	2.55
Low SDI	Both	Pancreatic cancer	2014	2.47	2.12	2.83
Low-middle SDI	Male	Pancreatic cancer	2014	3.54	3.26	3.82
Low-middle SDI	Female	Pancreatic cancer	2014	3.08	2.81	3.33
Low-middle SDI	Both	Pancreatic cancer	2014	3.31	3.08	3.52
Middle SDI	Male	Pancreatic cancer	2014	5.27	4.84	5.78
Middle SDI	Female	Pancreatic cancer	2014	3.97	3.60	4.30
Middle SDI	Both	Pancreatic cancer	2014	4.60	4.26	4.96
High SDI	Male	Pancreatic cancer	2015	11.18	10.57	11.53
High SDI	Female	Pancreatic cancer	2015	8.29	7.44	8.74
High SDI	Both	Pancreatic cancer	2015	9.66	8.91	10.07
High-middle SDI	Male	Pancreatic cancer	2015	9.41	8.84	10.01
High-middle SDI	Female	Pancreatic cancer	2015	6.21	5.72	6.58
High-middle SDI	Both	Pancreatic cancer	2015	7.67	7.22	8.02
Low SDI	Male	Pancreatic cancer	2015	2.79	2.33	3.21
Low SDI	Female	Pancreatic cancer	2015	2.25	1.93	2.60
Low SDI	Both	Pancreatic cancer	2015	2.51	2.18	2.86
Low-middle SDI	Male	Pancreatic cancer	2015	3.61	3.34	3.88
Low-middle SDI	Female	Pancreatic cancer	2015	3.21	2.94	3.47
Low-middle SDI	Both	Pancreatic cancer	2015	3.41	3.19	3.63
Middle SDI	Male	Pancreatic cancer	2015	5.32	4.86	5.85
Middle SDI	Female	Pancreatic cancer	2015	4.01	3.61	4.37
Middle SDI	Both	Pancreatic cancer	2015	4.64	4.29	5.03
High SDI	Male	Pancreatic cancer	2016	11.16	10.54	11.54
High SDI	Female	Pancreatic cancer	2016	8.32	7.46	8.80
High SDI	Both	Pancreatic cancer	2016	9.67	8.90	10.11
High-middle SDI	Male	Pancreatic cancer	2016	9.38	8.72	10.04
High-middle SDI	Female	Pancreatic cancer	2016	6.20	5.68	6.61
High-middle SDI	Both	Pancreatic cancer	2016	7.66	7.15	8.09
Low SDI	Male	Pancreatic cancer	2016	2.81	2.38	3.25
Low SDI	Female	Pancreatic cancer	2016	2.30	1.97	2.66
Low SDI	Both	Pancreatic cancer	2016	2.55	2.20	2.89
Low-middle SDI	Male	Pancreatic cancer	2016	3.68	3.41	3.95
Low-middle SDI	Female	Pancreatic cancer	2016	3.31	3.00	3.60
Low-middle SDI	Both	Pancreatic cancer	2016	3.49	3.25	3.73
Middle SDI	Male	Pancreatic cancer	2016	5.38	4.87	5.98
Middle SDI	Female	Pancreatic cancer	2016	4.07	3.65	4.47
Middle SDI	Both	Pancreatic cancer	2016	4.70	4.30	5.12
High SDI	Male	Pancreatic cancer	2017	11.07	10.44	11.48

High SDI	Female	Pancreatic cancer	2017	8.24	7.42	8.75
High SDI	Both	Pancreatic cancer	2017	9.59	8.80	10.04
High-middle SDI	Male	Pancreatic cancer	2017	9.34	8.59	10.09
High-middle SDI	Female	Pancreatic cancer	2017	6.22	5.65	6.71
High-middle SDI	Both	Pancreatic cancer	2017	7.65	7.13	8.09
Low SDI	Male	Pancreatic cancer	2017	2.85	2.41	3.27
Low SDI	Female	Pancreatic cancer	2017	2.36	2.03	2.70
Low SDI	Both	Pancreatic cancer	2017	2.60	2.27	2.94
Low-middle SDI	Male	Pancreatic cancer	2017	3.75	3.45	4.05
Low-middle SDI	Female	Pancreatic cancer	2017	3.39	3.05	3.75
Low-middle SDI	Both	Pancreatic cancer	2017	3.57	3.28	3.82
Middle SDI	Male	Pancreatic cancer	2017	5.48	4.90	6.09
Middle SDI	Female	Pancreatic cancer	2017	4.17	3.69	4.61
Middle SDI	Both	Pancreatic cancer	2017	4.80	4.39	5.24
High SDI	Male	Pancreatic cancer	2018	11.10	10.48	11.57
High SDI	Female	Pancreatic cancer	2018	8.25	7.39	8.78
High SDI	Both	Pancreatic cancer	2018	9.61	8.80	10.10
High-middle SDI	Male	Pancreatic cancer	2018	9.42	8.61	10.28
High-middle SDI	Female	Pancreatic cancer	2018	6.32	5.72	6.87
High-middle SDI	Both	Pancreatic cancer	2018	7.75	7.20	8.29
Low SDI	Male	Pancreatic cancer	2018	2.91	2.48	3.33
Low SDI	Female	Pancreatic cancer	2018	2.44	2.11	2.79
Low SDI	Both	Pancreatic cancer	2018	2.67	2.33	3.01
Low-middle SDI	Male	Pancreatic cancer	2018	3.85	3.51	4.20
Low-middle SDI	Female	Pancreatic cancer	2018	3.49	3.10	3.86
Low-middle SDI	Both	Pancreatic cancer	2018	3.67	3.35	3.96
Middle SDI	Male	Pancreatic cancer	2018	5.60	4.94	6.34
Middle SDI	Female	Pancreatic cancer	2018	4.30	3.79	4.84
Middle SDI	Both	Pancreatic cancer	2018	4.92	4.47	5.47
High SDI	Male	Pancreatic cancer	2019	11.10	10.44	11.59
High SDI	Female	Pancreatic cancer	2019	8.31	7.43	8.86
High SDI	Both	Pancreatic cancer	2019	9.64	8.83	10.16
High-middle SDI	Male	Pancreatic cancer	2019	9.52	8.59	10.47
High-middle SDI	Female	Pancreatic cancer	2019	6.41	5.76	7.03
High-middle SDI	Both	Pancreatic cancer	2019	7.84	7.17	8.40
Low SDI	Male	Pancreatic cancer	2019	2.95	2.51	3.38
Low SDI	Female	Pancreatic cancer	2019	2.50	2.16	2.86
Low SDI	Both	Pancreatic cancer	2019	2.72	2.38	3.09
Low-middle SDI	Male	Pancreatic cancer	2019	3.93	3.57	4.33
Low-middle SDI	Female	Pancreatic cancer	2019	3.58	3.16	3.99
Low-middle SDI	Both	Pancreatic cancer	2019	3.75	3.44	4.09
Middle SDI	Male	Pancreatic cancer	2019	5.72	4.96	6.54
Middle SDI	Female	Pancreatic cancer	2019	4.39	3.82	5.02
Middle SDI	Both	Pancreatic cancer	2019	5.03	4.48	5.63

location_name	sex_name	cause_name	year	Age-standardised incidence rate (per 100 000 person-years)	95% CI (lower)	95% CI (upper)
Afghanistan	Both	Pancreatic cancer	2019	2.57	1.78	3.71
Albania	Both	Pancreatic cancer	2019	6.53	4.90	8.55
Algeria	Both	Pancreatic cancer	2019	4.05	3.27	4.94
American Samoa	Both	Pancreatic cancer	2019	4.95	4.10	5.90
Andorra	Both	Pancreatic cancer	2019	10.31	7.83	13.25
Angola	Both	Pancreatic cancer	2019	3.19	2.55	4.04
Antigua and Barbuda	Both	Pancreatic cancer	2019	6.07	5.13	7.13
Argentina	Both	Pancreatic cancer	2019	11.54	9.02	14.41
Armenia	Both	Pancreatic cancer	2019	10.16	8.45	12.07
Australia	Both	Pancreatic cancer	2019	8.85	6.85	11.10
Austria	Both	Pancreatic cancer	2019	10.83	8.81	13.17
Azerbaijan	Both	Pancreatic cancer	2019	7.07	6.05	8.58
Bahamas	Both	Pancreatic cancer	2019	4.53	3.67	5.58
Bahrain	Both	Pancreatic cancer	2019	7.19	5.73	8.76
Bangladesh	Both	Pancreatic cancer	2019	2.07	1.30	3.09
Barbados	Both	Pancreatic cancer	2019	7.78	6.31	9.26
Belarus	Both	Pancreatic cancer	2019	7.07	5.52	9.16
Belgium	Both	Pancreatic cancer	2019	9.28	7.25	11.67
Belize	Both	Pancreatic cancer	2019	6.09	5.10	7.08
Benin	Both	Pancreatic cancer	2019	4.61	3.65	5.86
Bermuda	Both	Pancreatic cancer	2019	9.39	7.76	11.48
Bhutan	Both	Pancreatic cancer	2019	3.05	1.75	4.65
Bolivia (Plurinational State of)	Both	Pancreatic cancer	2019	4.94	3.40	6.66
Bosnia and Herzegovina	Both	Pancreatic cancer	2019	9.74	7.78	12.10
Botswana	Both	Pancreatic cancer	2019	7.49	5.52	10.04
Brazil	Both	Pancreatic cancer	2019	6.16	5.68	6.53
Brunei Darussalam	Both	Pancreatic cancer	2019	9.24	8.03	10.51
Bulgaria	Both	Pancreatic cancer	2019	10.93	8.61	13.71
Burkina Faso	Both	Pancreatic cancer	2019	3.40	2.60	4.30
Burundi	Both	Pancreatic cancer	2019	2.17	1.61	2.98
Cabo Verde	Both	Pancreatic cancer	2019	10.12	7.94	12.33
Cambodia	Both	Pancreatic cancer	2019	3.52	2.82	4.19
Cameroon	Both	Pancreatic cancer	2019	7.49	5.31	10.17
Canada	Both	Pancreatic cancer	2019	9.82	7.63	12.32
Central African Republic	Both	Pancreatic cancer	2019	2.06	1.47	2.78
Chad	Both	Pancreatic cancer	2019	2.66	2.13	3.31
Chile	Both	Pancreatic cancer	2019	7.55	5.87	9.55
China	Both	Pancreatic cancer	2019	5.78	4.94	6.69
Colombia	Both	Pancreatic cancer	2019	4.55	3.51	5.79
Comoros	Both	Pancreatic cancer	2019	3.64	2.81	4.59
Congo	Both	Pancreatic cancer	2019	5.14	3.38	7.30
Cook Islands	Both	Pancreatic cancer	2019	4.82	4.05	5.71
Costa Rica	Both	Pancreatic cancer	2019	6.95	5.40	8.75
Côte d'Ivoire	Both	Pancreatic cancer	2019	5.12	3.99	6.39
Croatia	Both	Pancreatic cancer	2019	9.58	7.60	12.02
Cuba	Both	Pancreatic cancer	2019	6.04	4.92	7.40
Cyprus	Both	Pancreatic cancer	2019	8.07	6.99	9.24
Czechia	Both	Pancreatic cancer	2019	11.85	9.59	14.34
Democratic People's Republic of Korea	Both	Pancreatic cancer	2019	3.80	2.88	4.81
Democratic Republic of the Congo	Both	Pancreatic cancer	2019	2.26	1.72	2.89
Denmark	Both	Pancreatic cancer	2019	10.19	7.86	12.92
Djibouti	Both	Pancreatic cancer	2019	4.02	2.88	5.50
Dominica	Both	Pancreatic cancer	2019	8.04	6.39	9.81
Dominican Republic	Both	Pancreatic cancer	2019	3.56	2.43	4.83
Ecuador	Both	Pancreatic cancer	2019	5.46	4.35	6.86
Egypt	Both	Pancreatic cancer	2019	4.33	2.99	6.01
El Salvador	Both	Pancreatic cancer	2019	5.03	3.77	6.48
Equatorial Guinea	Both	Pancreatic cancer	2019	5.91	3.63	8.84
Eritrea	Both	Pancreatic cancer	2019	2.95	2.01	4.08

Estonia	Both	Pancreatic cancer	2019	10.44	8.15	13.18
Eswatini	Both	Pancreatic cancer	2019	7.66	5.25	10.57
Ethiopia	Both	Pancreatic cancer	2019	1.47	0.98	2.12
Fiji	Both	Pancreatic cancer	2019	4.08	3.22	5.07
Finland	Both	Pancreatic cancer	2019	11.70	9.18	14.78
France	Both	Pancreatic cancer	2019	9.78	7.61	12.39
Gabon	Both	Pancreatic cancer	2019	7.69	5.35	10.57
Gambia	Both	Pancreatic cancer	2019	3.28	2.24	4.51
Georgia	Both	Pancreatic cancer	2019	6.17	5.09	7.37
Germany	Both	Pancreatic cancer	2019	11.89	9.28	15.27
Ghana	Both	Pancreatic cancer	2019	9.07	6.94	11.97
Greece	Both	Pancreatic cancer	2019	10.03	7.85	12.72
Greenland	Both	Pancreatic cancer	2019	18.89	15.51	22.26
Grenada	Both	Pancreatic cancer	2019	9.30	8.18	10.51
Guam	Both	Pancreatic cancer	2019	5.12	4.25	6.11
Guatemala	Both	Pancreatic cancer	2019	4.44	3.46	5.57
Guinea	Both	Pancreatic cancer	2019	1.80	1.36	2.26
Guinea-Bissau	Both	Pancreatic cancer	2019	4.53	3.09	6.10
Guyana	Both	Pancreatic cancer	2019	5.86	4.48	7.38
Haiti	Both	Pancreatic cancer	2019	2.39	1.66	3.32
Honduras	Both	Pancreatic cancer	2019	4.25	2.56	6.09
Hungary	Both	Pancreatic cancer	2019	11.88	9.79	14.32
Iceland	Both	Pancreatic cancer	2019	8.51	7.30	9.90
India	Both	Pancreatic cancer	2019	2.95	2.55	3.40
Indonesia	Both	Pancreatic cancer	2019	5.04	3.30	6.97
Iran (Islamic Republic of)	Both	Pancreatic cancer	2019	4.72	4.35	5.14
Iraq	Both	Pancreatic cancer	2019	5.91	4.48	7.26
Ireland	Both	Pancreatic cancer	2019	9.24	7.15	11.71
Israel	Both	Pancreatic cancer	2019	10.57	8.22	13.44
Italy	Both	Pancreatic cancer	2019	10.00	8.26	11.84
Jamaica	Both	Pancreatic cancer	2019	4.95	3.83	6.21
Japan	Both	Pancreatic cancer	2019	10.69	8.82	12.47
Jordan	Both	Pancreatic cancer	2019	5.19	4.34	6.18
Kazakhstan	Both	Pancreatic cancer	2019	6.53	5.58	7.57
Kenya	Both	Pancreatic cancer	2019	2.98	2.33	3.88
Kiribati	Both	Pancreatic cancer	2019	2.79	2.11	3.64
Kuwait	Both	Pancreatic cancer	2019	5.33	4.29	6.55
Kyrgyzstan	Both	Pancreatic cancer	2019	5.09	4.37	5.82
Lao People's Democratic Republic	Both	Pancreatic cancer	2019	3.31	2.59	4.14
Latvia	Both	Pancreatic cancer	2019	9.86	8.12	12.05
Lebanon	Both	Pancreatic cancer	2019	6.55	5.06	8.03
Lesotho	Both	Pancreatic cancer	2019	5.67	3.96	7.60
Liberia	Both	Pancreatic cancer	2019	4.72	3.44	6.22
Libya	Both	Pancreatic cancer	2019	7.03	5.41	9.04
Lithuania	Both	Pancreatic cancer	2019	9.01	7.29	11.11
Luxembourg	Both	Pancreatic cancer	2019	8.79	7.16	10.64
Madagascar	Both	Pancreatic cancer	2019	2.28	1.62	3.03
Malawi	Both	Pancreatic cancer	2019	2.90	2.24	3.63
Malaysia	Both	Pancreatic cancer	2019	3.61	2.81	4.53
Maldives	Both	Pancreatic cancer	2019	4.24	3.49	5.08
Mali	Both	Pancreatic cancer	2019	3.25	2.47	4.20
Malta	Both	Pancreatic cancer	2019	8.89	7.32	10.63
Marshall Islands	Both	Pancreatic cancer	2019	3.81	2.83	4.97
Mauritania	Both	Pancreatic cancer	2019	6.02	4.39	7.83
Mauritius	Both	Pancreatic cancer	2019	5.18	4.16	6.33
Mexico	Both	Pancreatic cancer	2019	5.78	4.97	6.64
Micronesia (Federated States of)	Both	Pancreatic cancer	2019	5.06	3.54	6.81
Monaco	Both	Pancreatic cancer	2019	18.14	14.37	21.72
Mongolia	Both	Pancreatic cancer	2019	5.97	4.70	7.51
Montenegro	Both	Pancreatic cancer	2019	10.75	9.05	12.74
Morocco	Both	Pancreatic cancer	2019	3.77	2.69	4.90

Mozambique	Both	Pancreatic cancer	2019	3.61	2.53	5.07
Myanmar	Both	Pancreatic cancer	2019	3.71	2.91	4.79
Namibia	Both	Pancreatic cancer	2019	3.83	2.99	4.89
Nauru	Both	Pancreatic cancer	2019	5.84	4.04	7.89
Nepal	Both	Pancreatic cancer	2019	2.76	1.65	4.08
Netherlands	Both	Pancreatic cancer	2019	11.29	8.83	14.11
New Zealand	Both	Pancreatic cancer	2019	8.18	6.80	9.75
Nicaragua	Both	Pancreatic cancer	2019	5.49	4.54	6.48
Niger	Both	Pancreatic cancer	2019	2.19	1.46	3.10
Nigeria	Both	Pancreatic cancer	2019	3.92	3.03	4.84
Niue	Both	Pancreatic cancer	2019	6.18	4.67	7.91
North Macedonia	Both	Pancreatic cancer	2019	10.52	8.38	13.13
Northern Mariana Islands	Both	Pancreatic cancer	2019	7.49	6.40	8.69
Norway	Both	Pancreatic cancer	2019	9.43	8.04	10.99
Oman	Both	Pancreatic cancer	2019	5.99	5.22	6.90
Pakistan	Both	Pancreatic cancer	2019	2.91	2.27	3.76
Palau	Both	Pancreatic cancer	2019	11.43	8.81	14.37
Palestine	Both	Pancreatic cancer	2019	6.70	5.59	7.95
Panama	Both	Pancreatic cancer	2019	4.68	3.59	6.02
Papua New Guinea	Both	Pancreatic cancer	2019	1.72	1.21	2.44
Paraguay	Both	Pancreatic cancer	2019	5.96	4.53	7.51
Peru	Both	Pancreatic cancer	2019	5.19	3.90	6.81
Philippines	Both	Pancreatic cancer	2019	4.19	3.39	5.19
Poland	Both	Pancreatic cancer	2019	9.94	8.41	11.70
Portugal	Both	Pancreatic cancer	2019	7.02	5.46	8.99
Puerto Rico	Both	Pancreatic cancer	2019	6.00	4.58	7.67
Qatar	Both	Pancreatic cancer	2019	7.62	5.76	10.04
Republic of Korea	Both	Pancreatic cancer	2019	8.98	7.51	10.71
Republic of Moldova	Both	Pancreatic cancer	2019	7.94	6.88	9.14
Romania	Both	Pancreatic cancer	2019	9.86	8.06	12.11
Russian Federation	Both	Pancreatic cancer	2019	7.88	6.94	8.97
Rwanda	Both	Pancreatic cancer	2019	3.37	2.68	4.29
Saint Kitts and Nevis	Both	Pancreatic cancer	2019	9.08	7.57	10.82
Saint Lucia	Both	Pancreatic cancer	2019	7.35	6.16	8.68
Saint Vincent and the Grenadines	Both	Pancreatic cancer	2019	6.16	5.34	7.07
Samoa	Both	Pancreatic cancer	2019	4.22	3.40	5.41
San Marino	Both	Pancreatic cancer	2019	10.90	8.29	14.47
Sao Tome and Principe	Both	Pancreatic cancer	2019	2.96	1.98	4.11
Saudi Arabia	Both	Pancreatic cancer	2019	5.02	4.06	6.14
Senegal	Both	Pancreatic cancer	2019	4.53	3.69	5.58
Serbia	Both	Pancreatic cancer	2019	10.21	8.06	12.76
Seychelles	Both	Pancreatic cancer	2019	7.17	6.26	8.25
Sierra Leone	Both	Pancreatic cancer	2019	3.49	2.75	4.44
Singapore	Both	Pancreatic cancer	2019	6.07	4.86	7.53
Slovakia	Both	Pancreatic cancer	2019	11.51	9.00	14.39
Slovenia	Both	Pancreatic cancer	2019	9.51	7.42	12.41
Solomon Islands	Both	Pancreatic cancer	2019	2.97	2.06	3.94
Somalia	Both	Pancreatic cancer	2019	1.58	0.85	2.55
South Africa	Both	Pancreatic cancer	2019	5.89	5.26	6.60
South Sudan	Both	Pancreatic cancer	2019	2.78	2.02	3.87
Spain	Both	Pancreatic cancer	2019	8.36	6.49	10.47
Sri Lanka	Both	Pancreatic cancer	2019	2.86	2.14	3.77
Sudan	Both	Pancreatic cancer	2019	3.59	2.44	5.52
Suriname	Both	Pancreatic cancer	2019	6.95	5.74	8.32
Sweden	Both	Pancreatic cancer	2019	8.40	7.06	9.72
Switzerland	Both	Pancreatic cancer	2019	9.39	7.28	11.91
Syrian Arab Republic	Both	Pancreatic cancer	2019	3.62	2.71	4.85
Taiwan (Province of China)	Both	Pancreatic cancer	2019	8.54	6.64	11.11
Tajikistan	Both	Pancreatic cancer	2019	5.18	4.31	6.35
Thailand	Both	Pancreatic cancer	2019	3.95	2.96	5.09
Timor-Leste	Both	Pancreatic cancer	2019	2.86	2.05	3.69

Togo	Both	Pancreatic cancer	2019	4.62	3.40	6.19
Tokelau	Both	Pancreatic cancer	2019	4.26	3.00	5.51
Tonga	Both	Pancreatic cancer	2019	4.26	3.12	5.61
Trinidad and Tobago	Both	Pancreatic cancer	2019	5.67	4.29	7.39
Tunisia	Both	Pancreatic cancer	2019	3.76	2.81	4.97
Turkey	Both	Pancreatic cancer	2019	8.08	6.47	9.93
Turkmenistan	Both	Pancreatic cancer	2019	3.72	2.99	4.64
Tuvalu	Both	Pancreatic cancer	2019	3.83	2.83	5.13
Uganda	Both	Pancreatic cancer	2019	4.71	3.68	5.86
Ukraine	Both	Pancreatic cancer	2019	8.27	6.92	9.71
United Arab Emirates	Both	Pancreatic cancer	2019	17.01	9.09	25.36
United Kingdom	Both	Pancreatic cancer	2019	9.22	7.69	10.83
United Republic of Tanzania	Both	Pancreatic cancer	2019	3.63	2.87	4.64
United States of America	Both	Pancreatic cancer	2019	10.37	8.94	11.96
United States Virgin Islands	Both	Pancreatic cancer	2019	10.86	8.89	13.01
Uruguay	Both	Pancreatic cancer	2019	13.93	11.03	17.43
Uzbekistan	Both	Pancreatic cancer	2019	4.83	4.12	5.65
Vanuatu	Both	Pancreatic cancer	2019	2.95	2.30	3.84
Venezuela (Bolivarian Republic of)	Both	Pancreatic cancer	2019	5.65	4.27	7.29
Viet Nam	Both	Pancreatic cancer	2019	4.30	3.38	5.40
Yemen	Both	Pancreatic cancer	2019	2.12	1.61	2.80
Zambia	Both	Pancreatic cancer	2019	4.79	3.45	6.58
Zimbabwe	Both	Pancreatic cancer	2019	7.72	5.95	9.75

location_name	sex_name	cause_name	year	Age-standardised death rate (per 100 000 person-years)	95% CI (lower)	95% CI (upper)
Afghanistan	Both	Pancreatic cancer	2019	2.72	1.91	3.89
Albania	Both	Pancreatic cancer	2019	6.68	5.05	8.73
Algeria	Both	Pancreatic cancer	2019	4.27	3.47	5.16
American Samoa	Both	Pancreatic cancer	2019	5.19	4.34	6.16
Andorra	Both	Pancreatic cancer	2019	10.26	7.83	13.13
Angola	Both	Pancreatic cancer	2019	3.40	2.74	4.26
Antigua and Barbuda	Both	Pancreatic cancer	2019	6.39	5.42	7.49
Argentina	Both	Pancreatic cancer	2019	11.94	10.90	13.02
Armenia	Both	Pancreatic cancer	2019	10.61	8.81	12.56
Australia	Both	Pancreatic cancer	2019	8.26	7.45	9.08
Austria	Both	Pancreatic cancer	2019	10.34	9.48	11.24
Azerbaijan	Both	Pancreatic cancer	2019	7.45	6.34	9.10
Bahamas	Both	Pancreatic cancer	2019	4.69	3.82	5.73
Bahrain	Both	Pancreatic cancer	2019	7.59	6.09	9.19
Bangladesh	Both	Pancreatic cancer	2019	2.21	1.39	3.30
Barbados	Both	Pancreatic cancer	2019	8.15	6.64	9.67
Belarus	Both	Pancreatic cancer	2019	7.06	5.54	9.10
Belgium	Both	Pancreatic cancer	2019	9.28	8.45	10.13
Belize	Both	Pancreatic cancer	2019	6.35	5.34	7.38
Benin	Both	Pancreatic cancer	2019	4.94	3.95	6.22
Bermuda	Both	Pancreatic cancer	2019	9.59	7.90	11.74
Bhutan	Both	Pancreatic cancer	2019	3.30	1.89	4.99
Bolivia (Plurinational State of)	Both	Pancreatic cancer	2019	5.34	3.69	7.18
Bosnia and Herzegovina	Both	Pancreatic cancer	2019	10.07	8.06	12.48
Botswana	Both	Pancreatic cancer	2019	7.96	5.90	10.63
Brazil	Both	Pancreatic cancer	2019	6.45	5.94	6.84
Brunei Darussalam	Both	Pancreatic cancer	2019	9.30	8.06	10.61
Bulgaria	Both	Pancreatic cancer	2019	11.09	8.78	13.87
Burkina Faso	Both	Pancreatic cancer	2019	3.64	2.78	4.61
Burundi	Both	Pancreatic cancer	2019	2.31	1.72	3.16
Cabo Verde	Both	Pancreatic cancer	2019	10.82	8.42	13.17
Cambodia	Both	Pancreatic cancer	2019	3.75	3.01	4.41
Cameroon	Both	Pancreatic cancer	2019	7.99	5.69	10.76
Canada	Both	Pancreatic cancer	2019	8.55	7.76	9.35
Central African Republic	Both	Pancreatic cancer	2019	2.19	1.60	2.91
Chad	Both	Pancreatic cancer	2019	2.86	2.31	3.56
Chile	Both	Pancreatic cancer	2019	7.76	7.00	8.53
China	Both	Pancreatic cancer	2019	5.99	5.12	6.93
Colombia	Both	Pancreatic cancer	2019	4.70	3.62	5.98
Comoros	Both	Pancreatic cancer	2019	3.89	3.03	4.89
Congo	Both	Pancreatic cancer	2019	5.48	3.65	7.68
Cook Islands	Both	Pancreatic cancer	2019	4.95	4.17	5.85
Costa Rica	Both	Pancreatic cancer	2019	7.20	5.57	9.08
Côte d'Ivoire	Both	Pancreatic cancer	2019	5.48	4.33	6.79
Croatia	Both	Pancreatic cancer	2019	9.07	7.23	11.36
Cuba	Both	Pancreatic cancer	2019	6.16	5.03	7.54
Cyprus	Both	Pancreatic cancer	2019	8.22	7.13	9.37
Czechia	Both	Pancreatic cancer	2019	11.81	9.57	14.29
Democratic People's Republic of Korea	Both	Pancreatic cancer	2019	3.88	2.98	4.84
Democratic Republic of the Congo	Both	Pancreatic cancer	2019	2.40	1.83	3.10
Denmark	Both	Pancreatic cancer	2019	10.07	8.99	11.09
Djibouti	Both	Pancreatic cancer	2019	4.31	3.12	5.82
Dominica	Both	Pancreatic cancer	2019	8.54	6.83	10.36
Dominican Republic	Both	Pancreatic cancer	2019	3.72	2.56	5.01
Ecuador	Both	Pancreatic cancer	2019	5.83	4.64	7.31
Egypt	Both	Pancreatic cancer	2019	4.43	3.07	6.15
El Salvador	Both	Pancreatic cancer	2019	5.25	3.94	6.76
Equatorial Guinea	Both	Pancreatic cancer	2019	6.36	3.97	9.37
Eritrea	Both	Pancreatic cancer	2019	3.13	2.15	4.29

Estonia	Both	Pancreatic cancer	2019	10.19	7.98	12.86
Eswatini	Both	Pancreatic cancer	2019	8.18	5.65	11.23
Ethiopia	Both	Pancreatic cancer	2019	1.60	1.07	2.32
Fiji	Both	Pancreatic cancer	2019	4.34	3.43	5.37
Finland	Both	Pancreatic cancer	2019	10.71	9.63	11.84
France	Both	Pancreatic cancer	2019	9.76	8.72	10.72
Gabon	Both	Pancreatic cancer	2019	8.18	5.73	11.17
Gambia	Both	Pancreatic cancer	2019	3.52	2.40	4.81
Georgia	Both	Pancreatic cancer	2019	6.28	5.18	7.46
Germany	Both	Pancreatic cancer	2019	10.82	9.86	11.83
Ghana	Both	Pancreatic cancer	2019	9.58	7.34	12.57
Greece	Both	Pancreatic cancer	2019	9.98	9.03	10.84
Greenland	Both	Pancreatic cancer	2019	19.29	15.73	22.84
Grenada	Both	Pancreatic cancer	2019	9.75	8.58	11.03
Guam	Both	Pancreatic cancer	2019	5.26	4.36	6.25
Guatemala	Both	Pancreatic cancer	2019	4.73	3.70	5.89
Guinea	Both	Pancreatic cancer	2019	1.91	1.46	2.38
Guinea-Bissau	Both	Pancreatic cancer	2019	4.72	3.26	6.34
Guyana	Both	Pancreatic cancer	2019	6.14	4.72	7.69
Haiti	Both	Pancreatic cancer	2019	2.54	1.78	3.49
Honduras	Both	Pancreatic cancer	2019	4.61	2.80	6.54
Hungary	Both	Pancreatic cancer	2019	12.02	9.90	14.42
Iceland	Both	Pancreatic cancer	2019	8.12	7.08	9.23
India	Both	Pancreatic cancer	2019	3.13	2.71	3.60
Indonesia	Both	Pancreatic cancer	2019	5.40	3.51	7.49
Iran (Islamic Republic of)	Both	Pancreatic cancer	2019	4.89	4.51	5.32
Iraq	Both	Pancreatic cancer	2019	6.12	4.68	7.47
Ireland	Both	Pancreatic cancer	2019	9.16	8.11	10.17
Israel	Both	Pancreatic cancer	2019	10.73	9.60	11.68
Italy	Both	Pancreatic cancer	2019	9.59	8.70	10.18
Jamaica	Both	Pancreatic cancer	2019	5.14	4.03	6.41
Japan	Both	Pancreatic cancer	2019	9.60	8.40	10.26
Jordan	Both	Pancreatic cancer	2019	5.40	4.54	6.43
Kazakhstan	Both	Pancreatic cancer	2019	6.67	5.72	7.73
Kenya	Both	Pancreatic cancer	2019	3.43	2.67	4.44
Kiribati	Both	Pancreatic cancer	2019	3.00	2.30	3.90
Kuwait	Both	Pancreatic cancer	2019	5.56	4.50	6.79
Kyrgyzstan	Both	Pancreatic cancer	2019	5.30	4.56	6.06
Lao People's Democratic Republic	Both	Pancreatic cancer	2019	3.52	2.78	4.38
Latvia	Both	Pancreatic cancer	2019	10.15	8.42	12.38
Lebanon	Both	Pancreatic cancer	2019	6.61	5.13	8.13
Lesotho	Both	Pancreatic cancer	2019	6.11	4.31	8.13
Liberia	Both	Pancreatic cancer	2019	5.07	3.73	6.67
Libya	Both	Pancreatic cancer	2019	7.28	5.63	9.32
Lithuania	Both	Pancreatic cancer	2019	9.18	7.45	11.31
Luxembourg	Both	Pancreatic cancer	2019	9.07	7.86	10.32
Madagascar	Both	Pancreatic cancer	2019	2.43	1.74	3.22
Malawi	Both	Pancreatic cancer	2019	3.11	2.42	3.87
Malaysia	Both	Pancreatic cancer	2019	3.80	2.98	4.76
Maldives	Both	Pancreatic cancer	2019	4.49	3.69	5.38
Mali	Both	Pancreatic cancer	2019	3.45	2.63	4.42
Malta	Both	Pancreatic cancer	2019	8.68	7.34	9.99
Marshall Islands	Both	Pancreatic cancer	2019	4.01	3.01	5.21
Mauritania	Both	Pancreatic cancer	2019	6.47	4.79	8.33
Mauritius	Both	Pancreatic cancer	2019	5.35	4.29	6.53
Mexico	Both	Pancreatic cancer	2019	6.01	5.16	6.87
Micronesia (Federated States of)	Both	Pancreatic cancer	2019	5.31	3.75	7.09
Monaco	Both	Pancreatic cancer	2019	17.81	14.20	21.23
Mongolia	Both	Pancreatic cancer	2019	6.28	4.99	7.86
Montenegro	Both	Pancreatic cancer	2019	10.89	9.14	12.90
Morocco	Both	Pancreatic cancer	2019	3.96	2.82	5.17

Mozambique	Both	Pancreatic cancer	2019	3.91	2.75	5.48
Myanmar	Both	Pancreatic cancer	2019	3.95	3.11	5.07
Namibia	Both	Pancreatic cancer	2019	4.11	3.23	5.22
Nauru	Both	Pancreatic cancer	2019	6.08	4.26	8.17
Nepal	Both	Pancreatic cancer	2019	2.99	1.79	4.42
Netherlands	Both	Pancreatic cancer	2019	11.10	10.03	12.16
New Zealand	Both	Pancreatic cancer	2019	7.46	6.80	8.11
Nicaragua	Both	Pancreatic cancer	2019	5.97	5.00	6.96
Niger	Both	Pancreatic cancer	2019	2.36	1.58	3.33
Nigeria	Both	Pancreatic cancer	2019	4.27	3.34	5.25
Niue	Both	Pancreatic cancer	2019	6.40	4.89	8.14
North Macedonia	Both	Pancreatic cancer	2019	10.81	8.64	13.42
Northern Mariana Islands	Both	Pancreatic cancer	2019	7.75	6.62	8.96
Norway	Both	Pancreatic cancer	2019	9.02	8.26	9.62
Oman	Both	Pancreatic cancer	2019	6.21	5.43	7.15
Pakistan	Both	Pancreatic cancer	2019	3.10	2.41	3.96
Palau	Both	Pancreatic cancer	2019	11.95	9.23	14.96
Palestine	Both	Pancreatic cancer	2019	7.01	5.84	8.30
Panama	Both	Pancreatic cancer	2019	4.88	3.74	6.27
Papua New Guinea	Both	Pancreatic cancer	2019	1.81	1.28	2.56
Paraguay	Both	Pancreatic cancer	2019	6.26	4.77	7.87
Peru	Both	Pancreatic cancer	2019	5.42	4.09	7.08
Philippines	Both	Pancreatic cancer	2019	4.41	3.64	5.32
Poland	Both	Pancreatic cancer	2019	10.58	8.88	12.52
Portugal	Both	Pancreatic cancer	2019	7.44	6.64	8.12
Puerto Rico	Both	Pancreatic cancer	2019	6.10	4.68	7.78
Qatar	Both	Pancreatic cancer	2019	8.07	6.09	10.50
Republic of Korea	Both	Pancreatic cancer	2019	8.25	7.34	9.20
Republic of Moldova	Both	Pancreatic cancer	2019	7.95	6.90	9.13
Romania	Both	Pancreatic cancer	2019	9.92	8.11	12.17
Russian Federation	Both	Pancreatic cancer	2019	8.23	7.20	9.34
Rwanda	Both	Pancreatic cancer	2019	3.62	2.90	4.58
Saint Kitts and Nevis	Both	Pancreatic cancer	2019	9.49	7.97	11.22
Saint Lucia	Both	Pancreatic cancer	2019	7.73	6.46	9.11
Saint Vincent and the Grenadines	Both	Pancreatic cancer	2019	6.50	5.64	7.45
Samoa	Both	Pancreatic cancer	2019	4.40	3.57	5.59
San Marino	Both	Pancreatic cancer	2019	11.10	7.38	16.00
Sao Tome and Principe	Both	Pancreatic cancer	2019	3.13	2.09	4.31
Saudi Arabia	Both	Pancreatic cancer	2019	5.13	4.16	6.24
Senegal	Both	Pancreatic cancer	2019	4.87	3.98	5.98
Serbia	Both	Pancreatic cancer	2019	10.80	8.55	13.44
Seychelles	Both	Pancreatic cancer	2019	7.50	6.54	8.61
Sierra Leone	Both	Pancreatic cancer	2019	3.74	2.97	4.72
Singapore	Both	Pancreatic cancer	2019	5.59	4.97	6.14
Slovakia	Both	Pancreatic cancer	2019	10.42	8.17	13.03
Slovenia	Both	Pancreatic cancer	2019	9.46	7.43	12.24
Solomon Islands	Both	Pancreatic cancer	2019	3.07	2.18	4.03
Somalia	Both	Pancreatic cancer	2019	1.69	0.92	2.72
South Africa	Both	Pancreatic cancer	2019	6.29	5.64	7.04
South Sudan	Both	Pancreatic cancer	2019	3.00	2.15	4.17
Spain	Both	Pancreatic cancer	2019	8.04	7.25	8.80
Sri Lanka	Both	Pancreatic cancer	2019	2.98	2.25	3.91
Sudan	Both	Pancreatic cancer	2019	3.76	2.60	5.77
Suriname	Both	Pancreatic cancer	2019	7.27	5.99	8.70
Sweden	Both	Pancreatic cancer	2019	9.04	8.31	9.69
Switzerland	Both	Pancreatic cancer	2019	8.73	7.82	9.59
Syrian Arab Republic	Both	Pancreatic cancer	2019	3.75	2.82	5.01
Taiwan (Province of China)	Both	Pancreatic cancer	2019	8.53	6.64	11.08
Tajikistan	Both	Pancreatic cancer	2019	5.62	4.68	6.87
Thailand	Both	Pancreatic cancer	2019	4.11	3.07	5.30
Timor-Leste	Both	Pancreatic cancer	2019	3.07	2.20	3.96

Togo	Both	Pancreatic cancer	2019	4.92	3.64	6.54
Tokelau	Both	Pancreatic cancer	2019	4.46	3.15	5.76
Tonga	Both	Pancreatic cancer	2019	4.49	3.29	5.87
Trinidad and Tobago	Both	Pancreatic cancer	2019	6.00	4.56	7.78
Tunisia	Both	Pancreatic cancer	2019	3.86	2.88	5.07
Turkey	Both	Pancreatic cancer	2019	8.24	6.62	10.08
Turkmenistan	Both	Pancreatic cancer	2019	3.86	3.12	4.80
Tuvalu	Both	Pancreatic cancer	2019	4.02	2.98	5.37
Uganda	Both	Pancreatic cancer	2019	5.00	3.95	6.21
Ukraine	Both	Pancreatic cancer	2019	8.03	6.77	9.42
United Arab Emirates	Both	Pancreatic cancer	2019	17.57	9.45	26.17
United Kingdom	Both	Pancreatic cancer	2019	9.22	8.58	9.66
United Republic of Tanzania	Both	Pancreatic cancer	2019	3.87	3.09	4.90
United States of America	Both	Pancreatic cancer	2019	10.06	9.43	10.52
United States Virgin Islands	Both	Pancreatic cancer	2019	11.30	9.28	13.44
Uruguay	Both	Pancreatic cancer	2019	14.47	13.22	15.74
Uzbekistan	Both	Pancreatic cancer	2019	5.18	4.41	6.03
Vanuatu	Both	Pancreatic cancer	2019	3.11	2.42	4.01
Venezuela (Bolivarian Republic of)	Both	Pancreatic cancer	2019	5.86	4.45	7.55
Viet Nam	Both	Pancreatic cancer	2019	4.53	3.57	5.68
Yemen	Both	Pancreatic cancer	2019	2.23	1.70	2.93
Zambia	Both	Pancreatic cancer	2019	5.09	3.71	6.95
Zimbabwe	Both	Pancreatic cancer	2019	8.06	6.24	10.23
