# Supplementary material for: Global burden of major gastrointestinal cancers and its association with socioeconomics, 1990–2019
Source: Front Oncol. 2022 Nov 1;12:942035. doi: 10.3389/fonc.2022.942035 (PMC9664003; doi:10.3389/fonc.2022.942035)
Supplement: Supplementary file 2 [file DataSheet_2.pdf]

Supplementary Methods

Overview

The GBD 2019 report enumerated approximately 369 diseases and injuries (1). Eighty-four behavioral, environmental and occupational, and metabolic risk factors were also recorded. Furthermore, estimates for sampling error and the 95% uncertainty intervals (UIs) are described in detail by the GBD 2017 Colorectal Cancer Collaborators (2).

Data sources

Based on the GBD definitions, 5 types of cancers were coded as the following: C18–C21.9, D01.0–D01.3, D12–D12.9, D37.3–D37.5 for colon and rectum cancer; C22–C22.9, D13.4 for liver cancer; C16–C16.9, D00.2, D13.1, D37.1 for stomach cancer; C15–C15.9, D00.1, D13.0 for esophageal cancer; and C25–C25.9, D13.6–D13.7 for pancreatic cancer. Data used in this study from GBD 2019, were generated using censuses, household surveys, civil registration, vital statistics, disease registries, health service use, and other sources (1).

Uncertainty analysis

Upon using the cohort-component method of population projection, final population estimates by single year and by single-year age groups were produced (3). The estimates of uncertainty were elucidated in several studies (4, 5). Notably, the estimation of uncertainty in the growth rate of the population using Bootstrap was introduced by Meyer *et al* (6).

Supplementary Figure

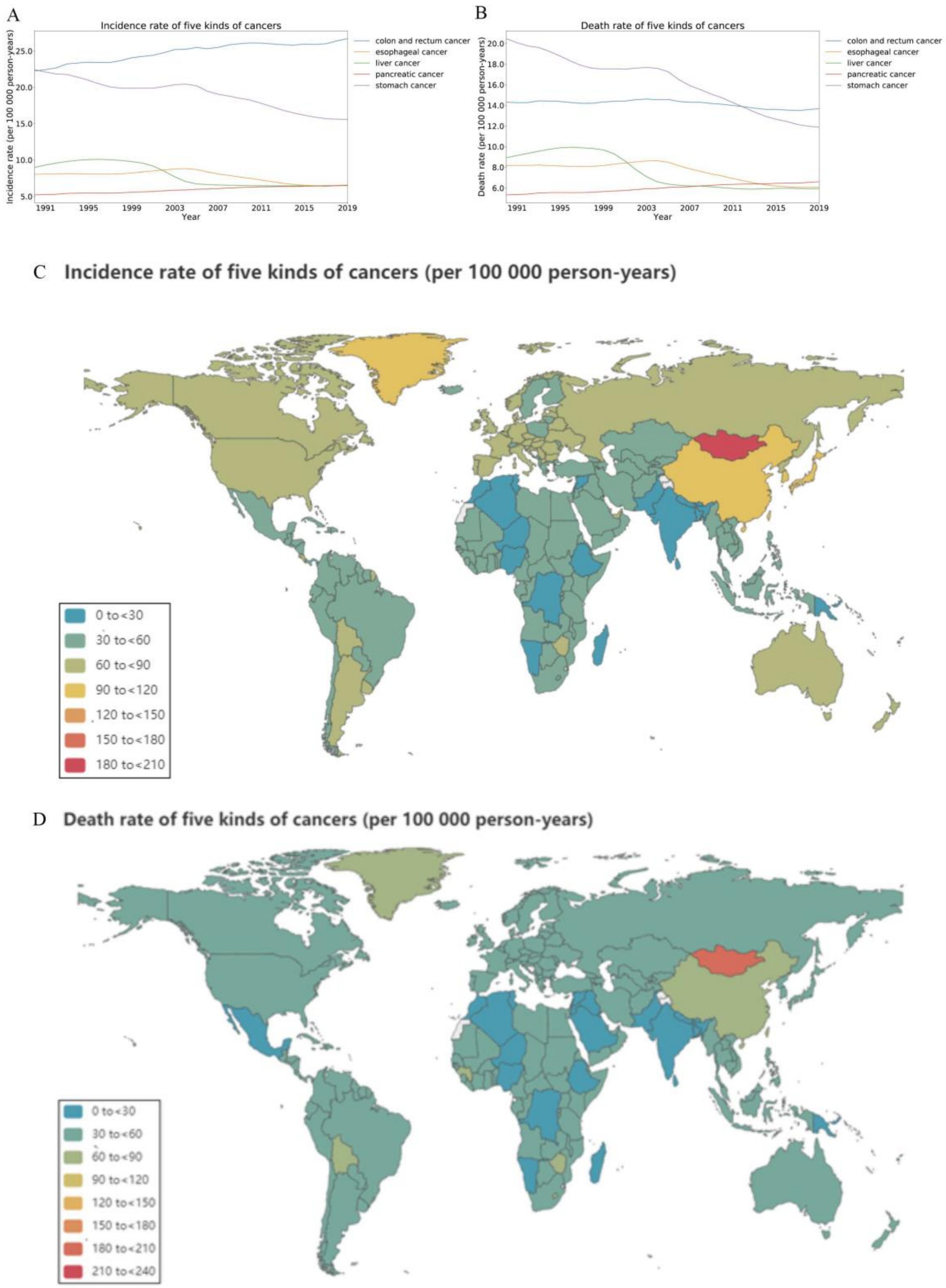

S.Figure 1: Burden of five common cancers of the gastrointestinal tract by country

and territory

Age-standardized incidence (A) and death (B) rate per 100000 person-years of five common cancers of the gastrointestinal tract from 1990 through 2019 by country and territory; age-standardized incidence (C) and death (D) rate of five common cancers of the gastrointestinal tract per 100000 person-years by country and territory, in 2019.

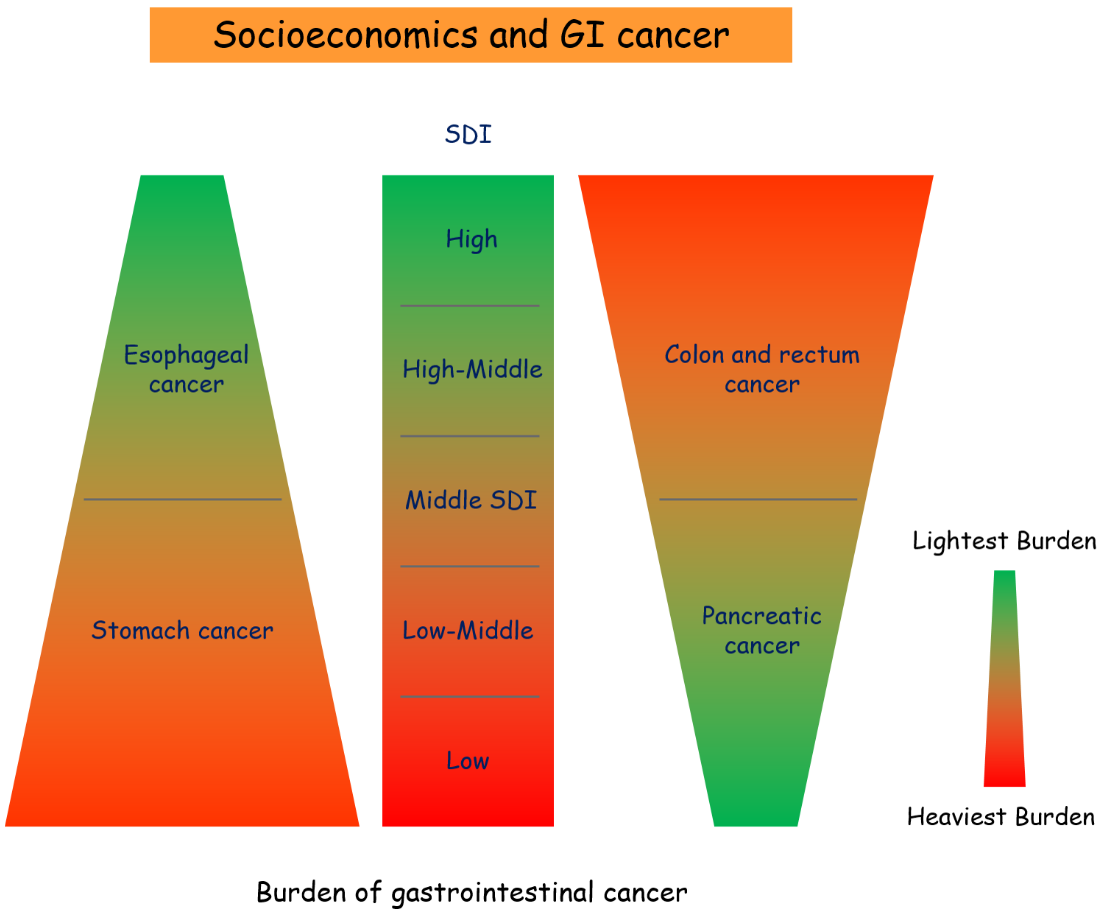

S.Figure 2: The correlation between SDI status and burden of five common cancers of the gastrointestinal tract

References

1. GBD 2019 Diseases and Injuries Collaborators. Global burden of 369 diseases and injuries in 204 countries and territories, 1990-2019: a systematic analysis for the Global Burden of Disease Study 2019. Lancet 2020;396(10258):1204-1222.

2. GBD 2017 Colorectal Cancer Collaborators. The global, regional, and national burden of colorectal cancer and its attributable risk factors in 195 countries and territories, 1990-2017: a systematic analysis for the Global Burden of Disease Study 2017. Lancet Gastroenterol Hepatol 2019;4(12):913-933.

3. Preston SH, Heuveline P, Guillot M. *Demography: measuring and modelling population processes*. Hoboken, NJ, USA: Wiley-Blackwell; 2000.

4. Population GBD, Fertility C. Population and fertility by age and sex for 195 countries and territories, 1950-2017: a systematic analysis for the Global Burden of Disease Study 2017. Lancet 2018;392(10159):1995-2051.

5. Collaborators GBDM. Global, regional, and national age-sex-specific mortality and life expectancy, 1950-2017: a systematic analysis for the Global Burden of Disease Study 2017. Lancet 2018;392(10159):1684-1735.

6. Meyer JS, Ingersoll CG, Mcdonald LL, *et al.* Estimating uncertainty in population growth rates: Jackknife Vs. Bootstrap techniques. Ecology 1986;67(5):1156-1166.
